# Supplementary material for: Parental Phasing Study Identified Lineage-Specific Variants Associated with Gene Expression and Epigenetic Modifications in European–Chinese Hybrid Pigs
Source: Animals (Basel). 2025 May 21;15(10):1494. doi: 10.3390/ani15101494 (PMC12108307; doi:10.3390/ani15101494)
Supplement: Supplementary file 1 [file animals-15-01494-s001.zip › animals-3621255-supplementary.pdf]

Parental Phasing Study Identified Lineage-Specific Variants Associated with Gene Expression and Epigenetic Modifications in European-Chinese Hybrid Pigs

The supplementary information contains:

Supplementary Figures

Supplementary Tables

Supplementary Table S1 Carcass Trait Measurements in All Hybrid Offspring from Three Crossbred Lines

Crossbreed line	Sample ID	Body Weight (kg)	Body Height (cm)	Body Length (cm)	Chest Circumference (cm)	Abdominal Circumference (cm)	Leg Circumference (cm)	Straight Carcass Length(cm)	Oblique Carcass Length(cm)	Total Cervical Length (cm)	Total Thoracic Length (cm)
LWxEHL	LE3	27.50	40.71	66.21	68.57	76.59	10.54	59.12	52.54	11.36	26.32
	LE1	19.35	38.52	63.51	59.51	66.83	11.31	54.30	49.31	7.11	25.51
	LE4	33.52	43.41	76.98	72.53	84.37	13.76	67.50	59,12	10.25	30.34
	LE5	27.45	41.53	69.28	67.85	79.53	12.53	60.21	51.52	10.54	29.87
	LE6	33.54	48.28	78.14	72.62	81.58	15.57	69.23	58.54	11.26	36.67
	LE2	36.21	43.21	74.98	73.48	84.38	13.16	66.12	57.45	10.24	29.26
BerkxGX	BG3	16.13	31.52	58.54	57.89	62.59	10.56	55.52	50.55	11.51	28.89
	BG4	13.38	30.53	54.42	54.76	60.49	12.58	49.21	45.57	10.50	25.54
	BG1	27.82	42.57	73.51	67.56	71.79	13.46	53.52	56.52	4.36	29.85
	BG2	20.95	42.82	62.52	63.59	60.34	12.46	44.51	43.56	NA	29.76
	BG5	20.43	36.83	67.15	63.49	61.71	11.55	50.52	52.78	4.54	29.53
	BG6	23.35	37.42	66.51	66.57	64.42	14.43	54.21	57.53	4.66	29.55
DRCxLG	DL3	30.22	39.12	73.54	66.23	68.38	11.25	44.90	52.12	7.27	28.42
	DL1	29.53	39.44	66.78	68.66	73.31	12.49	44.20	49.24	7.13	27.12
	DL2	31.12	39.73	72.52	70.43	67.53	12.18	45.63	53.57	11.45	27.44
	DL4	17.51	32.21	59.51	56.66	55.37	10.68	38.52	46.93	7.69	24.86
	DL5	23.12	37.12	69.85	63.76	60.97	12.77	40.88	45.85	8.31	26.84
	DL6	24.11	36.43	66.38	62.12	63.32	12.25	40.60	47.83	7.34	29.89

Total Lumbar Length (cm)	Number of Ribs (pairs)	Number of Lumbar Vertebrae (paris)	Skin Thickness at 6th-7th Rib (mm)	Shoulder Fat Thickness(mm)	Fat Thickness at 6th-7th Rib(mm)	Waist Fat Thickness(mm)	Hip Fat Thickness (mm)	Back Fat Thickness (Four-Point Average) (mm)	Eye muscle area(cm2)
15.55	14	7	1.35	15.70	9.06	5.61	8.74	9.78	23.57
14.57	14	6	2.28	16.84	7.46	4.14	6.06	8.63	17.30
19.39	14	7	3.34	13.90	13.70	11.56	10.96	12.53	18.14
15.44	14	7	2.56	19.32	9.48	8.34	6.98	11.03	19.90
14.57	16	5	2.34	17.64	8.14	3.20	5.70	8.67	18.26
19.23	14	7	2.80	18.64	12.98	5.18	8.14	11.24	21.42
15.42	15	6	2.88	13.77	7.33	6.68	4.92	8.18	9.94
12.34	15	6	1.88	13.34	7.13	4.38	10.07	8.73	8.46
19.27	15	7	2.98	15.52	10.86	18.37	10.21	13.71	25.06
19.53	14	8	1.75	14.65	4.01	3.83	8.72	7.80	18.66
17.87	16	7	2.42	6.82	4.68	9.41	5.50	6.60	12.32
16.54	15	7	2.91	14.85	9.20	5.67	2.66	8.10	17.92
14.66	14	6	7.16	33.07	12.95	13.95	12.54	18.13	25.67
14.21	14	7	6.79	27.27	17.71	11.83	7.90	16.18	16.04
16.63	14	6	7.10	25.84	13.87	13.67	10.56	15.99	22.53
12.84	14	6	7.21	24.62	7.76	6.23	5.86	11.12	14.57
13.86	14	6	8.87	30.07	13.00	9.71	7.53	15.08	15.51
12.82	14	5	7.92	21.48	8.72	7.15	8.41	11.44	18.72

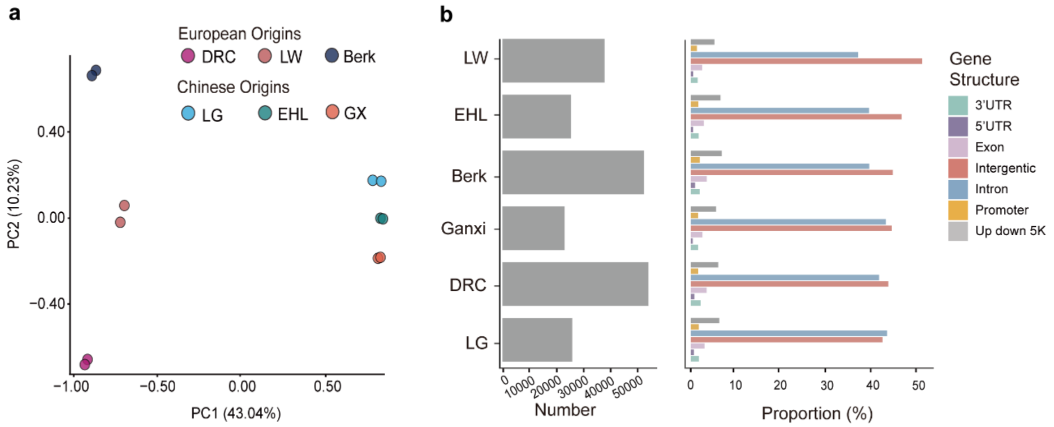

Supplementary Figure S1 Identification Phased SNPs (a) Clustering of SNPs detected based on phases from different lineages. (b) Number of lineage-specific SNPs detected in phases from different lineage (left), their proportions in different gene structures (right).

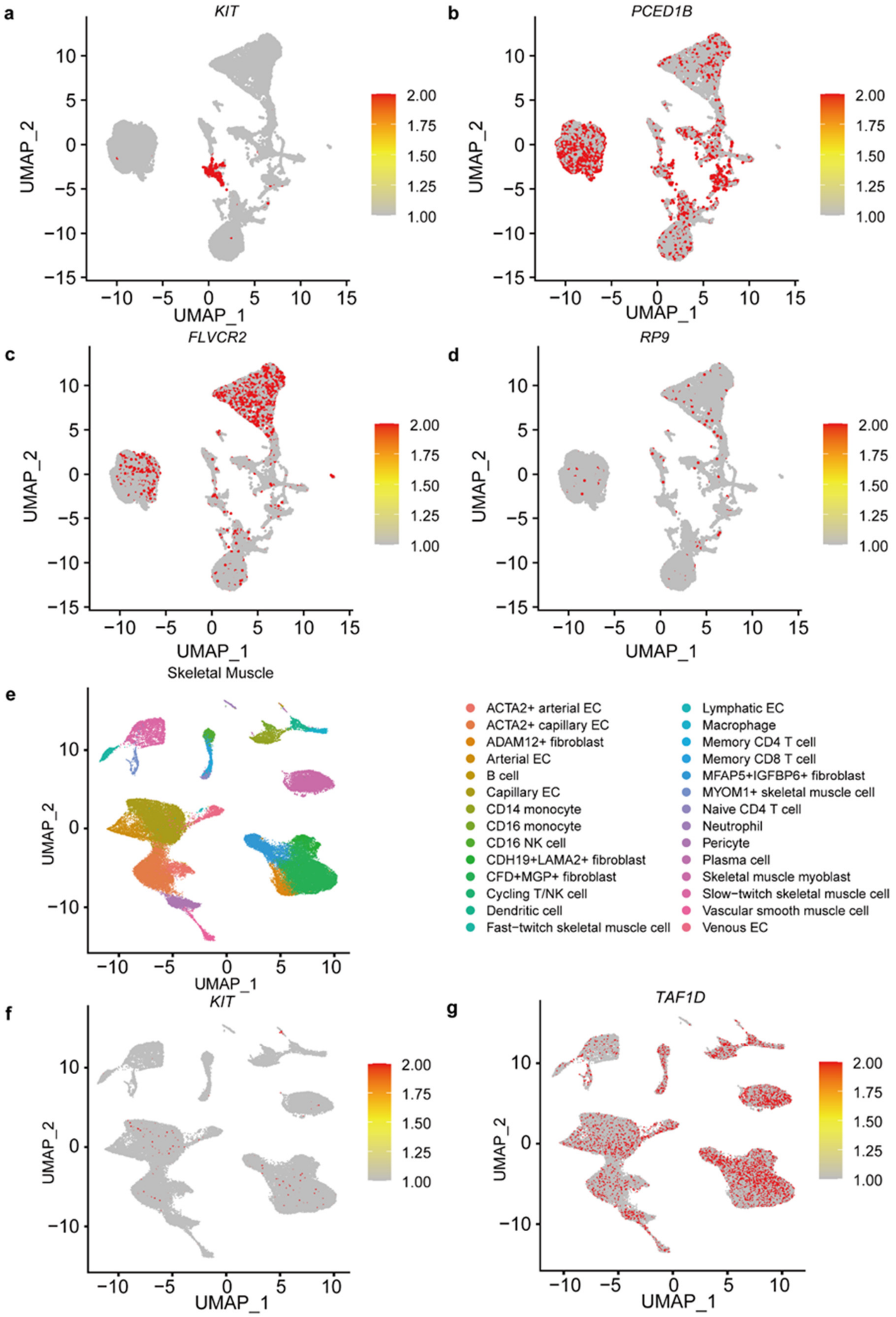
a
KIT
b
PCED1B
c
FLVCR2
d
RP9
e
Skeletal Muscle
f
KIT
g
TAF1D
UMAP_1
UMAP_2
ACTA2+ arterial EC
ACTA2+ capillary EC
ADAM12+ fibroblast
Arterial EC
B cell
Capillary EC
CD14 monocyte
CD16 monocyte
CD16 NK cell
CDH19+LAMA2+ fibroblast
CFD+MGP+ fibroblast
Cycling T/NK cell
Dendritic cell
Fast−twitch skeletal muscle cell
Lymphatic EC
Macrophage
Memory CD4 T cell
Memory CD8 T cell
MFAP5+IGFBP6+ fibroblast
MYOM1+ skeletal muscle cell
Naive CD4 T cell
Neutrophil
Pericyte
Plasma cell
Skeletal muscle myoblast
Slow−twitch skeletal muscle cell
Vascular smooth muscle cell
Venous EC

Supplementary Figure Sp2 Functional annotation of lineage-specific expression significantly associated with lineage-specific genetic variants (a) Major cell type expression of *KIT* in adipose cells. (b) Major cell type expression of *PCED1B* in adipose cells. (c) Major cell type expression of *FLVCR2* in adipose cells. (d) Major cell type expression of *RP9* in adipose cells. (e) UMAP plot depicting the cellular composition of skeletal muscle based on scRNA-seq data. (f) Major cell type expression of *KIT* in skeletal muscle. (g) Major cell type expression of *TAF1D* in skeletal muscle.

Supplementary Table 2 Phased SNPs significantly associated with phased gene expression

SNP region (cluster by window of 1Mb)			Tissue	Gene	beta	t-stat	p-value	FDR
chr1	6873844	6873845	BF	*IGF2R*	22.49048857	8.709741425	5.55E-06	0.019654356
chr1	7221182	7221183	BF	*IGF2R*	22.49048857	8.709741425	5.55E-06	0.019654356
chr1	8355985	8358552	BF	*TAGAP*	-4.110728	-8.25778583	8.91E-06	0.030242245
chr1	14226374	14226374	BF	*ESR1*	-4.93664356	-12.4619645	2.05E-07	0.001227173
chr1	14482510	14498941	BF	*ESR1*	-4.93664356	-12.4619645	2.05E-07	0.001227173
chr1	14638637	14683031	BF	*ESR1*	-4.93664356	-12.4619645	2.05E-07	0.001227173
chr1	141946735	142035676	BF	*SNRPN*	-40.92025	-11.8184998	3.37E-07	0.001341562
chr1	143312332	143412309	BF	*NDN*	-38.57955	-12.8943797	1.48E-07	0.000903454
chr1	268350266	268353701	BF	*DOLK*	3.594185	11.88850014	3.19E-07	0.001341562
chr1	268651190	268651255	BF	*DOLK*	3.594185	11.88850014	3.19E-07	0.001341562
chr1	268821042	268908783	BF	*DOLK*	3.594185	11.88850014	3.19E-07	0.001341562
chr1	269096574	269149568	BF	*DOLK*	3.594185	11.88850014	3.19E-07	0.001341562
chr1	269311767	269603688	BF	*DOLK*	3.594185	11.88850014	3.19E-07	0.001341562
chr1	269841740	269934229	BF	*DOLK*	3.594185	11.88850014	3.19E-07	0.001341562
chr1	269841740	269934229	BF	*DOLK*	-3.594185	-11.8885001	3.19E-07	0.001341562
chr1	273151353	273205572	BF	*FCN1*	-34.9131131	-14.5250497	4.76E-08	0.000903454
chr1	273151353	273205572	BF	*FCN2*	-714.185622	-18.1056666	5.66E-09	0.000512228
chr1	273678324	273682929	BF	*FCN1*	-34.9131131	-14.5250497	4.76E-08	0.000903454
chr1	273678324	273682929	BF	*FCN2*	-714.185622	-18.1056666	5.66E-09	0.000512228
chr1	273812285	273851417	BF	*FCN1*	-34.9131131	-14.5250497	4.76E-08	0.000903454
chr1	273812285	273851417	BF	*FCN2*	-714.185622	-18.1056666	5.66E-09	0.000512228

chr1	273964738	273964739	BF	*FCN1*	-34.9131131	-14.5250497	4.76E-08	0.000903454
chr1	273964738	273964739	BF	*FCN2*	-714.185622	-18.1056666	5.66E-09	0.000512228
chr2	8916593	8918167	BF	*LGALS12*	93.806575	8.565860101	6.44E-06	0.022109223
chr2	9183500	9183500	BF	*LGALS12*	93.806575	8.565860101	6.44E-06	0.022109223
chr2	9419982	9482744	BF	*FADS2*	5.090644714	8.575400193	6.38E-06	0.022001567
chr3	4919803	4919803	BF	*RAC1*	-140.821855	-13.9326131	7.09E-08	0.000903454
chr3	5100694	5209710	BF	*RAC1*	-140.821855	-13.9326131	7.09E-08	0.000903454
chr3	5100694	5209710	BF	*TECPR1*	-1.46732086	-8.62879959	6.03E-06	0.021090526
chr3	5100694	5209710	BF	*TECPR1*	1.467320857	8.628799586	6.03E-06	0.021090526
chr3	6516015	6516016	BF	*CYP3A29*	-195.983331	-9.33955331	2.96E-06	0.010667266
chr3	6664704	6676974	BF	*CYP3A29*	-195.983331	-9.33955331	2.96E-06	0.010667266
chr3	7692810	7692811	BF	*CYP3A29*	-195.983331	-9.33955331	2.96E-06	0.010667266
chr3	17161844	17161845	BF	*ZNF764*	-4.176601	-8.43823418	7.36E-06	0.02521484
chr4	3290413	3339946	BF	*PTP4A3*	-20.0072267	-13.6196544	8.81E-08	0.000903454
chr4	89530182	89530192	BF	*UFC1*	-32.75322	-8.18303971	9.65E-06	0.032481625
chr4	89662788	89662789	BF	*UFC1*	-32.75322	-8.18303971	9.65E-06	0.032481625
chr4	91610300	91645062	BF	*OR6N2*	7.1132148	11.31839479	5.05E-07	0.001976201
chr4	92113040	92127186	BF	*OR6N2*	7.1132148	11.31839479	5.05E-07	0.001976201
chr5	76642700	77382405	BF	*AMIGO2*	-32.306361	-15.6513318	2.32E-08	0.000512228
chr5	77483571	78328698	BF	*AMIGO2*	-32.306361	-15.6513318	2.32E-08	0.000512228
chr5	78347311	78529391	BF	*AMIGO2*	-32.306361	-15.6513318	2.32E-08	0.000512228
chr5	87410174	87410181	BF	*ENSSSCG00000000905*	-12.175553	-8.40063453	7.66E-06	0.026216279
chr5	88173620	88246087	BF	*ENSSSCG00000000905*	-12.175553	-8.40063453	7.66E-06	0.026216279
chr6	1102717	1103994	BF	*APRT*	-13.795183	-9.61028819	2.29E-06	0.008330502
chr6	18863978	18871746	BF	*ENSSSCG00000030300*	-489.295239	-8.89194551	4.61E-06	0.016334225
chr6	19125912	19219123	BF	*ENSSSCG00000030300*	-489.295239	-8.89194551	4.61E-06	0.016334225

chr6	19340591	19344307	BF	*NLRC5*	-19.6376363	-11.38055	4.80E-07	0.001882954
chr6	43001951	43123706	BF	*GPI*	-246.495018	-20.9259623	1.38E-09	0.000250414
chr6	43559541	43707175	BF	*GPI*	-246.495018	-20.9259623	1.38E-09	0.000250414
chr6	44709236	44744128	BF	*GPI*	-246.495018	-20.9259623	1.38E-09	0.000250414
chr6	44709236	44744128	BF	*USF2*	-53.28297	-8.61213511	6.14E-06	0.021414482
chr6	87795327	87795851	BF	*EIF3I*	35.180274	8.316717801	8.37E-06	0.02841913
chr7	23624983	23625490	BF	*ENSSSCG00000001398*	-65.4704	-9.00634846	4.11E-06	0.014669503
chr7	23780665	23901763	BF	*ENSSSCG00000041364*	-91.208435	-8.50022528	6.90E-06	0.023652918
chr7	24080293	24080294	BF	*ENSSSCG00000001398*	-65.4704	-9.00634846	4.11E-06	0.014669503
chr7	24882182	24889959	BF	*ENSSSCG00000001458*	-1.44579667	-11.7420303	3.58E-07	0.001415484
chr7	25002183	25004443	BF	*ENSSSCG00000001458*	-1.44579667	-11.7420303	3.58E-07	0.001415484
chr7	25202040	25202041	BF	*ENSSSCG00000001458*	-1.44579667	-11.7420303	3.58E-07	0.001415484
chr7	98629418	98629754	BF	*FLVCR2*	-20.66231	-8.91691534	4.50E-06	0.015953783
chr7	98750844	98924226	BF	*FLVCR2*	-20.66231	-8.91691534	4.50E-06	0.015953783
chr7	99139177	99141589	BF	*FLVCR2*	-20.66231	-8.91691534	4.50E-06	0.015953783
chr8	40697349	40730833	BF	*KIT*	-47.32528	-14.2420963	5.75E-08	0.000903454
chr8	40940384	40940385	BF	*KIT*	47.32528	14.2420963	5.75E-08	0.000903454
chr8	41431026	41497083	BF	*KIT*	-47.32528	-14.2420963	5.75E-08	0.000903454
chr9	865518	973500	BF	*SCUBE2*	-14.4276206	-11.7930337	3.44E-07	0.001361293
chr9	2551702	2551714	BF	*ENSSSCG00000033089*	-155.230156	-8.26114531	8.88E-06	0.030141532
chr9	8599463	8599463	BF	*PPME1*	-2.62159167	-8.33944387	8.17E-06	0.027762734
chr9	41125319	41750112	BF	*TTC12*	-5.03809953	-11.379447	4.81E-07	0.001882954
chr9	65397286	65397287	BF	*NUAK2*	-1.43554493	-8.26233015	8.87E-06	0.030111056
chr9	123587616	123588211	BF	*RNASEL*	-5.10919563	-9.08058303	3.82E-06	0.01363029
chr9	132444758	132587769	BF	*G0S2*	-65.594082	-10.056254	1.51E-06	0.005599678
chr9	132444758	132587769	BF	*G0S2*	65.594082	10.05625402	1.51E-06	0.005599678

chr9	132699606	132708900	BF	*G0S2*	-65.594082	-10.056254	1.51E-06	0.005599678
chr9	133208682	133311183	BF	*G0S2*	-65.594082	-10.056254	1.51E-06	0.005599678
chr9	133595770	133712807	BF	*G0S2*	-65.594082	-10.056254	1.51E-06	0.005599678
chr9	133983754	134004768	BF	*G0S2*	-65.594082	-10.056254	1.51E-06	0.005599678
chr9	134131366	134218185	BF	*G0S2*	-65.594082	-10.056254	1.51E-06	0.005599678
chr11	24760065	24760066	BF	*KBTBD6*	-4.8021675	-8.60248678	6.20E-06	0.021414482
chr11	25151724	25227259	BF	*KBTBD6*	-4.8021675	-8.60248678	6.20E-06	0.021414482
chr11	25702439	25702439	BF	*KBTBD6*	4.8021675	8.602486776	6.20E-06	0.021414482
chr11	26005085	26005086	BF	*KBTBD6*	4.8021675	8.602486776	6.20E-06	0.021414482
chr11	26240657	26245105	BF	*KBTBD6*	-4.8021675	-8.60248678	6.20E-06	0.021414482
chr11	26240657	26245105	BF	*KBTBD6*	4.8021675	8.602486776	6.20E-06	0.021414482
chr12	39191014	39191015	BF	*ENSBTAG00000025258*	-1296.80003	-8.23741995	9.11E-06	0.030736501
chr12	39946933	39991917	BF	*ENSBTAG00000025258*	-1296.80003	-8.23741995	9.11E-06	0.030736501
chr12	40523974	40568061	BF	*ENSBTAG00000025258*	-1296.80003	-8.23741995	9.11E-06	0.030736501
chr12	51591885	52244598	BF	*DVL2*	-27.088619	-9.93964019	1.68E-06	0.006126836
chr12	52527142	52543234	BF	*DVL2*	-27.088619	-9.93964019	1.68E-06	0.006126836
chr12	52842662	52941312	BF	*DVL2*	-27.088619	-9.93964019	1.68E-06	0.006126836
chr14	11821214	11821215	BF	*CLU*	-46.41071	-8.3338815	8.22E-06	0.027912854
chr14	11997023	11997024	BF	*CLU*	-46.41071	-8.3338815	8.22E-06	0.027912854
chr14	12153966	12153967	BF	*CLU*	-46.41071	-8.3338815	8.22E-06	0.027912854
chr14	20610737	20613185	BF	*DDX60*	-29.974876	-14.5920424	4.56E-08	0.000903454
chr14	21062935	21062936	BF	*DDX60*	-29.974876	-14.5920424	4.56E-08	0.000903454
chr14	100186265	100928651	BF	*IFIT2*	-98.82288	-13.1174664	1.26E-07	0.000903454
chr15	31652080	31660014	BF	*PTPN18*	-51.2045062	-9.24064695	3.26E-06	0.011662495
chr16	23527839	23587280	BF	*C9*	-19.701079	-11.1142152	5.99E-07	0.002288522
chr16	23794378	23794379	BF	*C9*	-19.701079	-11.1142152	5.99E-07	0.002288522

chr16	23904432	24076106	BF	*C9*	-19.701079	-11.1142152	5.99E-07	0.002288522
chr16	24313368	24462616	BF	*C9*	-19.701079	-11.1142152	5.99E-07	0.002288522
chr17	26295072	26329836	BF	*ZNF133*	-10.165323	-11.2720638	5.25E-07	0.002045535
chr17	27051598	27142316	BF	*ZNF133*	-10.165323	-11.2720638	5.25E-07	0.002045535
chr17	27244672	27305113	BF	*ZNF133*	-10.165323	-11.2720638	5.25E-07	0.002045535
chr17	34947111	35042859	BF	*KIF3B*	-13.8261182	-9.53247069	2.46E-06	0.008967524
chr17	38540093	38540094	BF	*RALY*	-93.9442318	-13.4796614	9.72E-08	0.000903454
chr17	46236644	46236645	BF	*PKIG*	-4.71744556	-10.1197946	1.43E-06	0.005409807
chr18	6092201	6092201	BF	*ENSSSCG00000033909*	-32.070071	-9.197376	3.40E-06	0.012166002
chr18	6653385	6654651	BF	*WDR86*	-5.77566125	-10.8287994	7.63E-07	0.002896415
NW_018084833.1	1731919	1755479	BF	*PTGDS*	-77.880621	-10.9748849	6.73E-07	0.00256183
NW_018084833.1	1599578	1617089	BF	*PTGDS*	-77.880621	-10.9748849	6.73E-07	0.00256183
NW_018084833.1	1226353	1331448	BF	*PTGDS*	-77.880621	-10.9748849	6.73E-07	0.00256183
NW_018084833.1	873604	1033319	BF	*PTGDS*	-77.880621	-10.9748849	6.73E-07	0.00256183
NW_018084833.1	760982	760983	BF	*PTGDS*	-77.880621	-10.9748849	6.73E-07	0.00256183
NW_018084979.1	2773614	2901941	BF	*ENSSSCG00000036983*	-22.3764892	-8.38023769	7.82E-06	0.026592598
NW_018084979.1	3289108	3384221	BF	*ENSSSCG00000036983*	-22.3764892	-8.38023769	7.82E-06	0.026592598
chr1	16516308	16528162	LD	*UST*	-3.954299	-10.8724026	7.35E-07	0.002683281
chr1	141946735	142035676	LD	*SNRPN*	-13.87255	-8.7860205	5.13E-06	0.012255747
chr2	1261179	1911690	LD	*IGF2*	-216.949419	-15.3113762	2.87E-08	0.000201002
chr2	2217254	2349801	LD	*IGF2*	-216.949419	-15.3113762	2.87E-08	0.000201002
chr2	3807326	3807327	LD	*CPT1A*	-6.06023688	-8.58123853	6.34E-06	0.014241803
chr2	7045180	7045181	LD	*TRPT1*	2.371567	8.177240291	9.71E-06	0.020925193
chr2	7346938	7346939	LD	*TRPT1*	2.371567	8.177240291	9.71E-06	0.020925193
chr2	7852330	7887560	LD	*TRPT1*	2.371567	8.177240291	9.71E-06	0.020925193
chr2	8079459	8212875	LD	*TRPT1*	2.371567	8.177240291	9.71E-06	0.020925193

chr2	12601035	12655822	LD	*SMTNL1*	-20.4814887	-8.79316118	5.10E-06	0.012255747
chr2	13076216	13076217	LD	*SMTNL1*	-20.4814887	-8.79316118	5.10E-06	0.012255747
chr2	60808398	60881143	LD	*CYP4F8*	-24.5102857	-12.2573624	2.39E-07	0.001482014
chr2	61145543	61145544	LD	*CYP4F8*	-24.5102857	-12.2573624	2.39E-07	0.001482014
chr2	61295472	61295473	LD	*CYP4F8*	-24.5102857	-12.2573624	2.39E-07	0.001482014
chr2	61646216	61646597	LD	*CYP4F8*	-24.5102857	-12.2573624	2.39E-07	0.001482014
chr2	61880938	61886307	LD	*CYP4F8*	-24.5102857	-12.2573624	2.39E-07	0.001482014
chr3	37358853	38155374	LD	*ENSSSCG00000007940*	-2.3232492	-8.71528947	5.52E-06	0.013063949
chr3	38270633	38314844	LD	*ENSSSCG00000007940*	-2.3232492	-8.71528947	5.52E-06	0.013063949
chr3	38492875	38841690	LD	*ENSSSCG00000007940*	-2.3232492	-8.71528947	5.52E-06	0.013063949
chr3	38492875	38841690	LD	*TEDC2*	-2.37935843	-9.2317189	3.29E-06	0.009921906
chr3	39073531	39191417	LD	*TEDC2*	-2.37935843	-9.2317189	3.29E-06	0.009921906
chr3	114473166	114478856	LD	*DNAJC27*	-1.71653952	-11.670566	3.79E-07	0.002339582
chr4	970521	970521	LD	*ADCK5*	-5.16533782	-11.7021698	3.70E-07	0.002289021
chr4	2166361	2167757	LD	*LY6E*	16.36563372	8.769418053	5.22E-06	0.012462278
chr4	31241379	31241380	LD	*ABRA*	227.054675	8.36173839	7.98E-06	0.017514761
chr4	38441196	38455357	LD	*MATN2*	8.61496	8.448219483	7.28E-06	0.016345332
chr4	39909777	39909778	LD	*MATN2*	8.61496	8.448219483	7.28E-06	0.016345332
chr4	116855152	116931599	LD	*RTCA*	-15.0275756	-8.19584583	9.52E-06	0.020585923
chr4	116855152	116931599	LD	*RTCA*	15.02757556	8.195845833	9.52E-06	0.020585923
chr5	46972114	46972835	LD	*TM7SF3*	-2.57761375	-8.23846038	9.10E-06	0.019891065
chr6	35975003	35975004	LD	*ZNF423*	1.87969375	8.718833383	5.50E-06	0.013063949
chr6	39369006	39369695	LD	*UQCRFS1*	-181.578677	-9.47102911	2.61E-06	0.007953742
chr8	40697349	40730833	LD	*KIT*	-47.32528	-14.2420963	5.75E-08	0.000903454
chr8	40940384	40940385	LD	*KIT*	47.32528	14.2420963	5.75E-08	0.000903454
chr8	41431026	41497083	LD	*KIT*	-47.32528	-14.2420963	5.75E-08	0.000903454

chr9	9931352	9954967	LD	*ARRB1*	-2.56320722	-8.32888089	8.26E-06	0.018092294
chr9	13012664	13012770	LD	*THRSP*	-68.967777	-8.27135237	8.78E-06	0.019218446
chr9	25952316	25952316	LD	*TAF1D*	-81.2272	-17.2604734	9.01E-09	7.40E-05
chr9	26550465	26615263	LD	*TAF1D*	-81.2272	-17.2604734	9.01E-09	7.40E-05
chr9	26550465	26615263	LD	*TAF1D*	81.2272	17.26047342	9.01E-09	7.40E-05
chr9	74555808	74605522	LD	*PEG10*	-88.486032	-10.7211881	8.37E-07	0.002683281
chr10	66514466	66514467	LD	*PITRM1*	-7.829875	-8.30037543	8.51E-06	0.018641861
chr10	66891884	66902012	LD	*PITRM1*	-7.829875	-8.30037543	8.51E-06	0.018641861
chr12	19377533	19584490	LD	*PSME3*	-14.7800118	-8.61628619	6.11E-06	0.013735429
chr12	19702952	20479694	LD	*PSME3*	-14.7800118	-8.61628619	6.11E-06	0.013735429
chr12	20612421	20700091	LD	*PSME3*	-14.7800118	-8.61628619	6.11E-06	0.013735429
chr12	20802186	21028529	LD	*PSME3*	-14.7800118	-8.61628619	6.11E-06	0.013735429
chr13	22874152	22874185	LD	*PLCD1*	9.292007143	11.97045204	2.99E-07	0.001851232
chr13	66125188	66125189	LD	*ATP2B2*	-5.54504867	-9.79296229	1.92E-06	0.005878116
chr13	130830746	130921300	LD	*LSG1*	-10.862825	-8.21045111	9.37E-06	0.020361473
chr13	131053889	131053890	LD	*LSG1*	-10.862825	-8.21045111	9.37E-06	0.020361473
chr13	131169759	131440354	LD	*LSG1*	-10.862825	-8.21045111	9.37E-06	0.020361473
chr13	131547611	131632205	LD	*LSG1*	-10.862825	-8.21045111	9.37E-06	0.020361473
chr13	131718872	131720368	LD	*LSG1*	-10.862825	-8.21045111	9.37E-06	0.020361473
chr13	132640475	132640475	LD	*LSG1*	-10.862825	-8.21045111	9.37E-06	0.020361473
chr13	196176256	196176560	LD	*DNAJC28*	-4.45468086	-9.27806245	3.15E-06	0.009582315
chr13	196990696	196990697	LD	*IFNAR1*	-5.98629444	-10.3227272	1.19E-06	0.003626737
chr13	197467403	197467404	LD	*IFNAR1*	-5.98629444	-10.3227272	1.19E-06	0.003626737
chr13	197588864	197588865	LD	*IFNAR1*	-5.98629444	-10.3227272	1.19E-06	0.003626737
chr14	100934220	100934221	LD	*LIPA*	-12.6819955	-8.27642458	8.73E-06	0.019118349
chr14	101600884	101600885	LD	*LIPA*	-12.6819955	-8.27642458	8.73E-06	0.019118349

chr14	101751446	101751447	LD	*LIPA*	-12.6819955	-8.27642458	8.73E-06	0.019118349
chr14	131845886	131920843	LD	*ENSSSCG00000023865*	-2.23461373	-8.42617506	7.45E-06	0.016366392
chr14	132462841	132468730	LD	*ENSSSCG00000023865*	-2.23461373	-8.42617506	7.45E-06	0.016366392
chr15	84256265	84261706	LD	*AGPS*	16.20182356	8.505898921	6.85E-06	0.015393164
chr15	103090533	103090534	LD	*TYW5*	-2.5568315	-9.89421663	1.75E-06	0.005351287
chr16	23221572	23221573	LD	*GDNF*	-10.29879	-8.240677	9.074E-06	0.0198534

Supplementary Table 3 Phased SVs significantly associated with phased gene expression

SV Region			Tissue	SV Type	Gene	Beta	t-stat	P-value	FDR
chr6	44138855	44138856	BF	INS	*GPI*	123.25	20.93	1.38E-09	0.00015
chr5	77568931	77569226	BF	DEL	*AMIGO2*	16.15	15.65	2.32E-08	0.00064
chr3	17155913	17156061	BF	DEL	*FUS*	47.55	15.26	2.95E-08	0.00064
chr3	17195654	17195655	BF	INS	*FUS*	47.55	15.26	2.95E-08	0.00064
chr8	41223208	41783661	BF	DUP	*KIT*	23.66	14.24	5.75E-08	0.00078
chr8	41290153	41294443	BF	DUP	*KIT*	47.33	14.24	5.75E-08	0.00078
chr8	41416313	41416314	BF	INS	*KIT*	23.66	14.24	5.75E-08	0.00078
chr1	14465696	14465697	BF	INS	*ESR1*	2.47	12.46	2.05E-07	0.00236
chr9	385677	385729	BF	DEL	*SCUBE2*	7.21	11.79	3.44E-07	0.00236
chr9	524772	524773	BF	INS	*SCUBE2*	7.21	11.79	3.44E-07	0.00236
chr9	562674	562675	BF	INS	*SCUBE2*	7.21	11.79	3.44E-07	0.00236
chr9	627343	627547	BF	DEL	*SCUBE2*	7.21	11.79	3.44E-07	0.00236
chr16	18285021	18285022	BF	INS	*ENSSSCG00000038326*	12.42	11.78	3.47E-07	0.00236
chr4	91303025	91303026	BF	INS	*OR6N2*	3.56	11.32	5.05E-07	0.00260

chr4	91564864	91564865	BF	INS	*OR6N2*	3.56	11.32	5.05E-07	0.00260
chr4	91629281	91629282	BF	INS	*OR6N2*	3.56	11.32	5.05E-07	0.00260
chr17	26575682	26575683	BF	INS	*ZNF133*	5.08	11.27	5.25E-07	0.00260
chr17	26609373	26609374	BF	INS	*ZNF133*	5.08	11.27	5.25E-07	0.00260
chr17	26616461	26616462	BF	INS	*ZNF133*	5.08	11.27	5.25E-07	0.00260
chr9	133237296	133237932	BF	DEL	*G0S2*	32.80	10.06	1.51E-06	0.00685
chr12	52626428	52626429	BF	INS	*DVL2*	13.54	9.94	1.68E-06	0.00732
chr6	693319	694987	BF	DEL	*APRT*	6.90	9.61	2.29E-06	0.00922
chr6	698250	698251	BF	INS	*APRT*	6.90	9.61	2.29E-06	0.00922
chr11	18172003	18172004	BF	INS	*CAB39L*	4.70	9.44	2.69E-06	0.01010
chr11	18468661	18468662	BF	INS	*CAB39L*	4.70	9.44	2.69E-06	0.01010
chr15	32107959	32107960	BF	INS	*PTPN18*	25.60	9.24	3.26E-06	0.01107
chr16	60186379	60186380	BF	INS	*HMMR*	5.12	9.14	3.61E-06	0.01107
chr16	60403058	60403059	BF	INS	*HMMR*	5.12	9.14	3.61E-06	0.01107
chr7	98771920	98771921	BF	INS	*FLVCR2*	10.33	8.92	4.50E-06	0.01324
chr2	8372545	8372546	BF	INS	*LGALS12*	-46.90	-8.57	6.44E-06	0.01670
chr2	8462410	8462710	BF	DEL	*LGALS12*	-46.90	-8.57	6.44E-06	0.01670
chr2	8524295	8524296	BF	INS	*LGALS12*	-46.90	-8.57	6.44E-06	0.01670
chr2	8530340	8530630	BF	DEL	*LGALS12*	-46.90	-8.57	6.44E-06	0.01670
chr2	89209751	89209752	BF	INS	*FAM151B*	7.68	8.51	6.80E-06	0.01682
chr2	89244034	89244035	BF	INS	*FAM151B*	7.68	8.51	6.80E-06	0.01682
chr3	17798331	17798332	BF	INS	*ZNF764*	2.09	8.44	7.36E-06	0.01742
chr9	8589546	8589547	BF	INS	*PPME1*	-1.31	-8.34	8.17E-06	0.01755
chr14	11329898	11329899	BF	INS	*CLU*	23.21	8.33	8.22E-06	0.01755
chr14	11333615	11333666	BF	DEL	*CLU*	23.21	8.33	8.22E-06	0.01755
chr14	11548158	11548159	BF	INS	*CLU*	23.21	8.33	8.22E-06	0.01755

chr1	471693	471694	BF	INS	*ERMARD*	10.50	8.02	1.15E-05	0.02276
chr1	525720	525721	BF	INS	*ERMARD*	10.50	8.02	1.15E-05	0.02276
chr1	534087	534088	BF	INS	*ERMARD*	10.50	8.02	1.15E-05	0.02276
chr5	77568931	77569226	BF	DEL	*PCED1B*	6.23	8.00	1.18E-05	0.02300
chr5	33687593	33687594	BF	INS	*CCT2*	-35.04	-7.90	1.32E-05	0.02358
chr5	33737700	33737988	BF	DEL	*CCT2*	-35.04	-7.90	1.32E-05	0.02358
chr5	33742773	33742774	BF	INS	*CCT2*	-35.04	-7.90	1.32E-05	0.02358
chr5	33750204	33750205	BF	INS	*CCT2*	-35.04	-7.90	1.32E-05	0.02358
chr5	33853480	33853481	BF	INS	*CCT2*	-35.04	-7.90	1.32E-05	0.02358
chr7	1811808	1811991	BF	DEL	*BPHL*	-9.07	-7.81	1.45E-05	0.02436
chr2	32596021	32596022	BF	INS	*LIN7C*	-14.42	-7.78	1.51E-05	0.02436
chr2	32617465	32617527	BF	DEL	*LIN7C*	-14.42	-7.78	1.51E-05	0.02436
chr5	64244220	64244538	BF	DEL	*TAPBPL*	-4.27	-7.76	1.54E-05	0.02436
chr6	88887946	88887947	BF	INS	*RBBP4*	-65.20	-7.70	1.64E-05	0.02544
chr9	132723713	132723714	BF	INS	*HHAT*	2.39	7.65	1.74E-05	0.02639
chr5	14878005	14878006	BF	INS	*FKBP11*	23.50	7.65	1.74E-05	0.02639
chr18	40229191	40229192	BF	INS	*RP9*	20.92	7.59	1.86E-05	0.02743
chr18	40229201	40229201	BF	BND	*RP9*	41.84	7.59	1.86E-05	0.02743
chr6	75777926	75779607	BF	DEL	*ATP13A2*	-3.19	-7.48	2.11E-05	0.03068
chr7	58930757	58930758	BF	INS	*CLK3*	11.67	7.38	2.38E-05	0.03411
chr9	19413920	19414169	BF	DEL	*TMEM126B*	10.60	7.08	3.36E-05	0.04557
chr9	19469266	19469267	BF	INS	*TMEM126B*	10.60	7.08	3.36E-05	0.04557
chr9	19545416	19545417	BF	INS	*TMEM126B*	10.60	7.08	3.36E-05	0.04557
chr1	29806755	29806756	BF	INS	*ENSSSCG00000043152*	-0.49	-7.08	3.39E-05	0.04557
chr9	26177858	26177859	LD	INS	*TAF1D*	40.61	17.26	9.01E-09	0.00039

chr9	26245864	26245865	LD	INS	*TAF1D*	40.61	17.26	9.01E-09	0.00039
chr3	57293569	57293787	LD	DEL	*IGKV1*	32.88	16.21	1.66E-08	0.00039
chr3	57409472	57409473	LD	INS	*IGKV1*	32.88	16.21	1.66E-08	0.00039
chr3	113617595	113617596	LD	INS	*DNAJC27*	0.87	13.62	8.80E-08	0.00163
chr2	61825111	61825262	LD	DEL	*CYP4F8*	12.36	13.16	1.22E-07	0.00163
chr2	61852282	61852283	LD	INS	*CYP4F8*	12.36	13.16	1.22E-07	0.00163
chr2	61679917	61679918	LD	INS	*CYP4F8*	12.26	12.26	2.39E-07	0.00224
chr2	61687728	61688497	LD	DEL	*CYP4F8*	12.26	12.26	2.39E-07	0.00224
chr2	61750603	61750604	LD	INS	*CYP4F8*	12.26	12.26	2.39E-07	0.00224
chr11	23955908	23955909	LD	INS	*DNAJC15*	34.58	9.89	1.76E-06	0.01267
chr11	24131246	24131247	LD	INS	*DNAJC15*	34.58	9.89	1.76E-06	0.01267
chr12	19950921	19951242	LD	DEL	*PSME3*	7.39	8.62	6.11E-06	0.03320
chr12	20174962	20175050	LD	DEL	*PSME3*	7.39	8.62	6.11E-06	0.03320
chr14	132367009	132367010	LD	INS	*ENSSSCG00000023865*	1.12	8.43	7.45E-06	0.03320
chr14	132500414	132500700	LD	DEL	*ENSSSCG00000023865*	1.12	8.43	7.45E-06	0.03320
chr14	132500882	132501973	LD	DEL	*ENSSSCG00000023865*	1.12	8.43	7.45E-06	0.03320
chr14	132514137	132514327	LD	DEL	*ENSSSCG00000023865*	1.12	8.43	7.45E-06	0.03320
chr2	137162594	137162595	LD	INS	*C5orf24*	-12.06	-8.28	8.68E-06	0.03692
chr13	131533103	131533104	LD	INS	*LSG1*	5.43	8.21	9.37E-06	0.03812
chr12	54525533	54525534	LD	INS	*GAS7*	1.45	7.88	1.35E-05	0.04404
chr8	41223208	41783661	LD	DUP	*KIT*	4.20	7.87	1.37E-05	0.04404
chr8	41290153	41294443	LD	DUP	*KIT*	8.40	7.87	1.37E-05	0.04404

Supplementary Table 4 Phased SNPs significantly associated with phased histone modifications and CTCF signals

SNP Sites			Histone Peaks	beta	t-stat	p-value	FDR	Histone Modification	Tissue
chr10	25885820	25885821	chr10_25885349_25886252	6.0831	12.6900944	1.3985E-06	0.017305224	CTCF	BF
NW_018084861.1	68997	68998	NW_018084861.1_68848_70031	-17.52524	-12.478317	1.5908E-06	0.022081348	CTCF	BF
chr7	135297822	135297823	chr7_135297819_135299247	3.5626678	11.3265722	5.0567E-07	0.002634537	CTCF	BF
chr15	132933952	132933953	chr15_132933680_132934130	-10.028827	-23.815655	1.029E-08	0.000140634	CTCF	BF
chr15	134023357	134023358	chr15_134023394_134023878	-8.63073	-13.479725	8.798E-07	0.000924936	CTCF	BF
chr15	134023395	134023396	chr15_134023394_134023878	-8.63073	-13.479725	8.798E-07	0.000924936	CTCF	BF
chr15	134023459	134023460	chr15_134023394_134023878	-8.63073	-13.479725	8.798E-07	0.000924936	CTCF	BF
chr15	134023462	134023463	chr15_134023394_134023878	-8.63073	-13.479725	8.798E-07	0.000924936	CTCF	BF
chr15	134023472	134023473	chr15_134023394_134023878	-8.63073	-13.479725	8.798E-07	0.000924936	CTCF	BF
chr15	134023475	134023476	chr15_134023394_134023878	-8.63073	-13.479725	8.798E-07	0.000924936	CTCF	BF
chr15	134023495	134023496	chr15_134023394_134023878	-8.63073	-13.479725	8.798E-07	0.000924936	CTCF	BF

chr15	134023656	134023657	chr15_134023394_134023878	-8.63073	-13.479725	8.798E-07	0.000924936	CTCF	BF
chr15	134023703	134023704	chr15_134023394_134023878	-8.63073	-13.479725	8.798E-07	0.000924936	CTCF	BF
chr15	134023929	134023930	chr15_134023394_134023878	-8.63073	-13.479725	8.798E-07	0.000924936	CTCF	BF
chr15	134023944	134023945	chr15_134023394_134023878	-8.63073	-13.479725	8.798E-07	0.000924936	CTCF	BF
chr15	134023960	134023961	chr15_134023394_134023878	-8.63073	-13.479725	8.798E-07	0.000924936	CTCF	BF
chr15	132935447	132935448	chr15_132935443_132935675	-10.863634	-10.817257	4.7079E-06	0.004595922	CTCF	BF
chr18	50487302	50487303	chr18_50487021_50487742	-10.914442	-10.332143	6.6473E-06	0.003813165	CTCF	BF
chr18	50487303	50487304	chr18_50487021_50487742	-10.914442	-10.332143	6.6473E-06	0.003813165	CTCF	BF
chr18	50487346	50487347	chr18_50487021_50487742	-10.914442	-10.332143	6.6473E-06	0.003813165	CTCF	BF
chr18	50487383	50487384	chr18_50487021_50487742	-10.914442	-10.332143	6.6473E-06	0.003813165	CTCF	BF
chr18	50487396	50487397	chr18_50487021_50487742	-10.914442	-10.332143	6.6473E-06	0.003813165	CTCF	BF
chr18	50487515	50487516	chr18_50487021_50487742	-10.914442	-10.332143	6.6473E-06	0.003813165	CTCF	BF
chr18	50487534	50487535	chr18_50487021_50487742	-10.914442	-10.332143	6.6473E-06	0.003813165	CTCF	BF

chr18	50487587	50487588	chr18_50487021_50487742	-10.914442	-10.332143	6.6473E-06	0.003813165	CTCF	BF
chr18	50487628	50487629	chr18_50487021_50487742	-10.914442	-10.332143	6.6473E-06	0.003813165	CTCF	BF
chr18	50487635	50487636	chr18_50487021_50487742	-10.914442	-10.332143	6.6473E-06	0.003813165	CTCF	BF
chr18	50487698	50487699	chr18_50487021_50487742	-10.914442	-10.332143	6.6473E-06	0.003813165	CTCF	BF
chr18	50487721	50487722	chr18_50487021_50487742	-10.914442	-10.332143	6.6473E-06	0.003813165	CTCF	BF
chr18	50487752	50487753	chr18_50487021_50487742	-10.914442	-10.332143	6.6473E-06	0.003813165	CTCF	BF
chr18	50487766	50487767	chr18_50487021_50487742	-10.914442	-10.332143	6.6473E-06	0.003813165	CTCF	BF
chr18	4602648	4602649	chr18_4602452_4602695	-4.8415716	-9.8181381	9.7331E-06	0.004885414	CTCF	BF
chr18	4602729	4602730	chr18_4602452_4602695	-4.8415716	-9.8181381	9.7331E-06	0.004885414	CTCF	BF
chr1	67666953	67666954	chr1_67666918_67667536	-5.2073362	-11.563446	2.842E-06	0.005471967	CTCF	BF
chr1	67666956	67666957	chr1_67666918_67667536	-5.2073362	-11.563446	2.842E-06	0.005471967	CTCF	BF
chr1	67666981	67666982	chr1_67666918_67667536	-5.2073362	-11.563446	2.842E-06	0.005471967	CTCF	BF
chr1	67667109	67667110	chr1_67666918_67667536	-5.2073362	-11.563446	2.842E-06	0.005471967	CTCF	BF

chr1	67667139	67667140	chr1_67666918_67667536	-5.2073362	-11.563446	2.842E-06	0.005471967	CTCF	BF
chr1	67667200	67667201	chr1_67666918_67667536	-5.2073362	-11.563446	2.842E-06	0.005471967	CTCF	BF
chr1	67667207	67667208	chr1_67666918_67667536	-5.2073362	-11.563446	2.842E-06	0.005471967	CTCF	BF
chr1	67667367	67667368	chr1_67666918_67667536	-5.2073362	-11.563446	2.842E-06	0.005471967	CTCF	BF
chr1	67667377	67667378	chr1_67666918_67667536	-5.2073362	-11.563446	2.842E-06	0.005471967	CTCF	BF
chr1	67667388	67667389	chr1_67666918_67667536	-5.2073362	-11.563446	2.842E-06	0.005471967	CTCF	BF
chr1	67667456	67667457	chr1_67666918_67667536	-5.2073362	-11.563446	2.842E-06	0.005471967	CTCF	BF
chr1	67667577	67667578	chr1_67666918_67667536	-5.2073362	-11.563446	2.842E-06	0.005471967	CTCF	BF
chr1	67667598	67667599	chr1_67666918_67667536	-5.2073362	-11.563446	2.842E-06	0.005471967	CTCF	BF
chr1	13685370	13685371	chr1_13685259_13685960	-1.602106	-10.559172	5.6465E-06	0.010095092	CTCF	BF
NW_018085302.1	54383	54384	NW_018085302.1_53617_54636	-14.142168	-12.68577	1.4022E-06	0.009941932	CTCF	BF
NW_018085302.1	54055	54056	NW_018085302.1_53617_54636	-14.142168	-12.68577	1.4022E-06	0.009941932	CTCF	BF
chr2	10282413	10282414	chr2_10282167_10282751	-7.34034	-11.686627	2.6222E-06	0.009941932	CTCF	BF

chr2	12364110	12364111	chr2_12364014_12364703	-3.964064	-11.355021	3.2625E-06	0.009941932	CTCF	BF
chr2	12364201	12364202	chr2_12364014_12364703	-3.964064	-11.355021	3.2625E-06	0.009941932	CTCF	BF
chr2	12364211	12364212	chr2_12364014_12364703	-3.964064	-11.355021	3.2625E-06	0.009941932	CTCF	BF
chr3	94258046	94258047	chr3_94257966_94258426	-11.19148	-15.095272	3.6688E-07	0.006886372	CTCF	BF
chr3	24933230	24933231	chr3_24933018_24933300	-13.632385	-11.783575	2.4625E-06	0.023110806	CTCF	BF
NW_018085169.1	752	753	NW_018085169.1_315_1352	-3.443465	-9.9497184	8.8136E-06	0.02982492	CTCF	BF
NW_018085169.1	234	235	NW_018085169.1_315_1352	-3.443465	-9.9497184	8.8136E-06	0.02982492	CTCF	BF
NW_018085169.1	223	224	NW_018085169.1_315_1352	-3.443465	-9.9497184	8.8136E-06	0.02982492	CTCF	BF
chr3	39750360	39750361	chr3_39750090_39750815	16.62361	9.84544637	9.5338E-06	0.02982492	CTCF	BF
chr6	157501411	157501412	chr6_157501477_157502173	-7.90106	-13.477448	8.8094E-07	0.006059403	CTCF	BF
chr6	157501438	157501439	chr6_157501477_157502173	-7.90106	-13.477448	8.8094E-07	0.006059403	CTCF	BF
chr6	157501472	157501473	chr6_157501477_157502173	-7.90106	-13.477448	8.8094E-07	0.006059403	CTCF	BF
chr6	38979877	38979878	chr6_38978939_38979845	-6.5284264	-9.9507133	8.807E-06	0.045433299	CTCF	BF

chr7	58340343	58340344	chr7_58340187_58340615	-4.944174	-14.319126	5.5211E-07	0.001974978	CTCF	BF
chr7	59080974	59080975	chr7_59080922_59081129	-10.087824	-14.062961	6.3472E-07	0.001974978	CTCF	BF
chr7	59081161	59081162	chr7_59080922_59081129	-10.087824	-14.062961	6.3472E-07	0.001974978	CTCF	BF
chr7	117425078	117425079	chr7_117425122_117425333	-5.7414208	-13.616346	8.1407E-07	0.001974978	CTCF	BF
NW_018085303.1	467467	467468	NW_018085303.1_467089_467382	-4.5943414	-12.22068	1.8657E-06	0.001974978	CTCF	BF
NW_018085303.1	467456	467457	NW_018085303.1_467089_467382	-4.5943414	-12.22068	1.8657E-06	0.001974978	CTCF	BF
NW_018085303.1	467409	467410	NW_018085303.1_467089_467382	-4.5943414	-12.22068	1.8657E-06	0.001974978	CTCF	BF
NW_018085303.1	467356	467357	NW_018085303.1_467089_467382	-4.5943414	-12.22068	1.8657E-06	0.001974978	CTCF	BF
NW_018085303.1	467340	467341	NW_018085303.1_467089_467382	-4.5943414	-12.22068	1.8657E-06	0.001974978	CTCF	BF
NW_018085303.1	467277	467278	NW_018085303.1_467089_467382	-4.5943414	-12.22068	1.8657E-06	0.001974978	CTCF	BF
NW_018085303.1	467262	467263	NW_018085303.1_467089_467382	-4.5943414	-12.22068	1.8657E-06	0.001974978	CTCF	BF
NW_018085303.1	467219	467220	NW_018085303.1_467089_467382	-4.5943414	-12.22068	1.8657E-06	0.001974978	CTCF	BF
NW_018085303.1	467168	467169	NW_018085303.1_467089_467382	-4.5943414	-12.22068	1.8657E-06	0.001974978	CTCF	BF

NW_018085303.1	467162	467163	NW_018085303.1_467089_467382	-4.5943414	-12.22068	1.8657E-06	0.001974978	CTCF	BF
NW_018085303.1	467077	467078	NW_018085303.1_467089_467382	-4.5943414	-12.22068	1.8657E-06	0.001974978	CTCF	BF
NW_018085303.1	467047	467048	NW_018085303.1_467089_467382	-4.5943414	-12.22068	1.8657E-06	0.001974978	CTCF	BF
NW_018085303.1	467023	467024	NW_018085303.1_467089_467382	-4.5943414	-12.22068	1.8657E-06	0.001974978	CTCF	BF
chr1	7142303	7142304	chr1_7142109_7143698	-12.490136	-23.406508	1.1799E-08	0.000579536	H3K27ac	BF
chr1	8446216	8446217	chr1_8446009_8448061	-6.0258088	-15.602855	2.8381E-07	0.003484849	H3K27ac	BF
chr1	8446232	8446233	chr1_8446009_8448061	-6.0258088	-15.602855	2.8381E-07	0.003484849	H3K27ac	BF
chr1	8446299	8446300	chr1_8446009_8448061	-6.0258088	-15.602855	2.8381E-07	0.003484849	H3K27ac	BF
chr1	6377506	6377507	chr1_6376567_6378188	0.668172	12.1553679	1.9436E-06	0.012676786	H3K27ac	BF
chr1	6377945	6377946	chr1_6376567_6378188	0.668172	12.1553679	1.9436E-06	0.012676786	H3K27ac	BF
chr1	206722661	206722662	chr1_206722005_206722716	-7.1816699	-11.517027	2.9301E-06	0.012676786	H3K27ac	BF
chr1	7461783	7461784	chr1_7461631_7462495	5.1468389	11.4331696	3.0972E-06	0.012676786	H3K27ac	BF
chr1	7461865	7461866	chr1_7461631_7462495	5.1468389	11.4331696	3.0972E-06	0.012676786	H3K27ac	BF

chr1	7461929	7461930	chr1_7461631_7462495	5.1468389	11.4331696	3.0972E-06	0.012676786	H3K27ac	BF
chr1	7461937	7461938	chr1_7461631_7462495	5.1468389	11.4331696	3.0972E-06	0.012676786	H3K27ac	BF
chr1	7462348	7462349	chr1_7461631_7462495	5.1468389	11.4331696	3.0972E-06	0.012676786	H3K27ac	BF
chr1	139257019	139257020	chr1_139256166_139257734	-12.787671	-10.915557	4.3974E-06	0.014283876	H3K27ac	BF
chr1	16361430	16361431	chr1_16361499_16362028	-0.7862163	-10.834068	4.6531E-06	0.014283876	H3K27ac	BF
chr1	16362089	16362090	chr1_16361499_16362028	-0.7862163	-10.834068	4.6531E-06	0.014283876	H3K27ac	BF
chr1	16362096	16362097	chr1_16361499_16362028	-0.7862163	-10.834068	4.6531E-06	0.014283876	H3K27ac	BF
chr2	18346567	18346568	chr2_18346492_18347068	-15.556396	-19.540781	4.8894E-08	0.000811354	H3K27ac	BF
chr2	18346605	18346606	chr2_18346492_18347068	-15.556396	-19.540781	4.8894E-08	0.000811354	H3K27ac	BF
chr2	20963903	20963904	chr2_20963095_20964083	-8.6397768	-13.185114	1.0428E-06	0.011244314	H3K27ac	BF
chr2	6393679	6393680	chr2_6393537_6393983	2.584687	12.6371783	1.444E-06	0.011244314	H3K27ac	BF
chr2	24645894	24645895	chr2_24645898_24648669	6.0045578	10.8065163	4.7433E-06	0.011244314	H3K27ac	BF
chr2	24646027	24646028	chr2_24645898_24648669	6.0045578	10.8065163	4.7433E-06	0.011244314	H3K27ac	BF

chr2	24646041	24646042	chr2_24645898_24648669	6.0045578	10.8065163	4.7433E-06	0.011244314	H3K27ac	BF
chr2	24646299	24646300	chr2_24645898_24648669	6.0045578	10.8065163	4.7433E-06	0.011244314	H3K27ac	BF
chr2	24646357	24646358	chr2_24645898_24648669	6.0045578	10.8065163	4.7433E-06	0.011244314	H3K27ac	BF
chr2	24646545	24646546	chr2_24645898_24648669	6.0045578	10.8065163	4.7433E-06	0.011244314	H3K27ac	BF
chr2	24646594	24646595	chr2_24645898_24648669	6.0045578	10.8065163	4.7433E-06	0.011244314	H3K27ac	BF
chr2	24646974	24646975	chr2_24645898_24648669	6.0045578	10.8065163	4.7433E-06	0.011244314	H3K27ac	BF
chr2	24647098	24647099	chr2_24645898_24648669	6.0045578	10.8065163	4.7433E-06	0.011244314	H3K27ac	BF
chr2	24647213	24647214	chr2_24645898_24648669	6.0045578	10.8065163	4.7433E-06	0.011244314	H3K27ac	BF
chr2	17512018	17512019	chr2_17511734_17512156	-4.7398947	-9.9952484	8.5184E-06	0.018847141	H3K27ac	BF
chr3	124428389	124428390	chr3_124428300_124428750	-4.3175773	-12.41323	1.6557E-06	0.057832173	H3K27ac	BF
chr3	106148473	106148474	chr3_106148439_106149389	-8.2467075	-10.104986	7.851E-06	0.058234008	H3K27ac	BF
chr3	26207651	26207652	chr3_26207481_26208042	-9.8434153	-10.024287	8.3358E-06	0.058234008	H3K27ac	BF
chr3	26207887	26207888	chr3_26207481_26208042	-9.8434153	-10.024287	8.3358E-06	0.058234008	H3K27ac	BF

chr3	26208111	26208112	chr3_26207481_26208042	-9.8434153	-10.024287	8.3358E-06	0.058234008	H3K27ac	BF
chr4	35767132	35767133	chr4_35767181_35769406	18.038273	11.856572	2.3495E-06	0.023211265	H3K27ac	BF
chr4	35767365	35767366	chr4_35767181_35769406	18.038273	11.856572	2.3495E-06	0.023211265	H3K27ac	BF
chr4	35767386	35767387	chr4_35767181_35769406	18.038273	11.856572	2.3495E-06	0.023211265	H3K27ac	BF
chr4	108626955	108626956	chr4_108626948_108627560	-7.7101863	-10.732886	4.994E-06	0.024668927	H3K27ac	BF
chr4	108627012	108627013	chr4_108626948_108627560	-7.7101863	-10.732886	4.994E-06	0.024668927	H3K27ac	BF
chr4	108627145	108627146	chr4_108626948_108627560	-7.7101863	-10.732886	4.994E-06	0.024668927	H3K27ac	BF
chr5	60107922	60107923	chr5_60105805_60108574	9.6510688	20.5108409	3.3411E-08	0.000953974	H3K27ac	BF
chr5	4608802	4608803	chr5_4608570_4608989	-7.3589964	-18.925995	6.2836E-08	0.000953974	H3K27ac	BF
chr5	63607336	63607337	chr5_63606077_63608250	-16.337236	-13.634262	8.0587E-07	0.008156478	H3K27ac	BF
chr5	90826513	90826514	chr5_90826589_90827333	-4.36174	-10.735204	4.9859E-06	0.027561607	H3K27ac	BF
chr5	90174593	90174594	chr5_90173088_90175146	-6.4527143	-10.609995	5.4462E-06	0.027561607	H3K27ac	BF
chr5	90174627	90174628	chr5_90173088_90175146	-6.4527143	-10.609995	5.4462E-06	0.027561607	H3K27ac	BF

chr5	77754641	77754642	chr5_77754058 _77755070	-5.1211158	-9.8636152	9.4037E-06	0.03569184	H3K27ac	BF
chr5	77757817	77757818	chr5_77757811_77758325	-5.1211158	-9.8636152	9.4037E-06	0.03569184	H3K27ac	BF
chr10	51685504	51685505	chr10_51684598_51686370	-2.728775	-10.268329	6.9632E-06	0.019846294	H3K27me3	BF
chr10	51685781	51685782	chr10_51684598_51686370	-2.728775	-10.268329	6.9632E-06	0.019846294	H3K27me3	BF
chr10	51686117	51686118	chr10_51684598_51686370	-2.728775	-10.268329	6.9632E-06	0.019846294	H3K27me3	BF
chr10	51686323	51686324	chr10_51684598_51686370	-2.728775	-10.268329	6.9632E-06	0.019846294	H3K27me3	BF
chr10	51686365	51686366	chr10_51684598_51686370	-2.728775	-10.268329	6.9632E-06	0.019846294	H3K27me3	BF
chr10	51686415	51686416	chr10_51684598_51686370	-2.728775	-10.268329	6.9632E-06	0.019846294	H3K27me3	BF
NW_018084861.1	87976	87977	NW_018084861.1_87526_88177	-3.493022	-22.06459	1.88E-08	0.000314379	H3K27me3	BF
chr12	25319329	25319330	chr12_25319200_25320118	-10.448452	-23.347603	1.2036E-08	0.000185843	H3K27me3	BF
chr12	49244943	49244944	chr12_49244240_49245184	-5.270618	-10.656224	5.271E-06	0.013563977	H3K27me3	BF
chr12	49245128	49245129	chr12_49244240_49245184	-5.270618	-10.656224	5.271E-06	0.013563977	H3K27me3	BF
chr12	49244194	49244195	chr12_49244240_49245184	5.270618	10.6562237	5.271E-06	0.013563977	H3K27me3	BF

chr12	49244645	49244646	chr12_49244240_49245184	5.270618	10.6562237	5.271E-06	0.013563977	H3K27me3	BF
chr12	49244815	49244816	chr12_49244240_49245184	5.270618	10.6562237	5.271E-06	0.013563977	H3K27me3	BF
chr14	87350476	87350477	chr14_87350230_87350645	-13.57996	-15.501061	2.9861E-07	0.004065445	H3K27me3	BF
chr14	87350483	87350484	chr14_87350230_87350645	-13.57996	-15.501061	2.9861E-07	0.004065445	H3K27me3	BF
chr15	134023227	134023228	chr15_134023262_134023879	-5.9727702	-11.986699	2.1621E-06	0.003806225	H3K27me3	BF
chr15	134023357	134023358	chr15_134023262_134023879	-5.9727702	-11.986699	2.1621E-06	0.003806225	H3K27me3	BF
chr15	134023395	134023396	chr15_134023262_134023879	-5.9727702	-11.986699	2.1621E-06	0.003806225	H3K27me3	BF
chr15	134023459	134023460	chr15_134023262_134023879	-5.9727702	-11.986699	2.1621E-06	0.003806225	H3K27me3	BF
chr15	134023462	134023463	chr15_134023262_134023879	-5.9727702	-11.986699	2.1621E-06	0.003806225	H3K27me3	BF
chr15	134023472	134023473	chr15_134023262_134023879	-5.9727702	-11.986699	2.1621E-06	0.003806225	H3K27me3	BF
chr15	134023475	134023476	chr15_134023262_134023879	-5.9727702	-11.986699	2.1621E-06	0.003806225	H3K27me3	BF
chr15	134023495	134023496	chr15_134023262_134023879	-5.9727702	-11.986699	2.1621E-06	0.003806225	H3K27me3	BF
chr15	134023656	134023657	chr15_134023262_134023879	-5.9727702	-11.986699	2.1621E-06	0.003806225	H3K27me3	BF

chr15	134023703	134023704	chr15_134023262_134023879	-5.9727702	-11.986699	2.1621E-06	0.003806225	H3K27me3	BF
chr15	134023929	134023930	chr15_134023262_134023879	-5.9727702	-11.986699	2.1621E-06	0.003806225	H3K27me3	BF
chr15	134023944	134023945	chr15_134023262_134023879	-5.9727702	-11.986699	2.1621E-06	0.003806225	H3K27me3	BF
chr15	134023960	134023961	chr15_134023262_134023879	-5.9727702	-11.986699	2.1621E-06	0.003806225	H3K27me3	BF
chr15	110060	110061	chr15_109691_110014	-8.393636	-9.8996493	9.1516E-06	0.013962838	H3K27me3	BF
chr15	110103	110104	chr15_109691_110014	-8.393636	-9.8996493	9.1516E-06	0.013962838	H3K27me3	BF
chr18	839377	839378	chr18_839109_839395	-5.901676	-15.325669	3.262E-07	0.004665295	H3K27me3	BF
chr18	7423633	7423634	chr18_7423385_7423658	-9.149918	-11.58537	2.8014E-06	0.020032984	H3K27me3	BF
chr1	142269745	142269746	chr1_142269835_142270820	-4.952488	-16.506135	1.8315E-07	0.003375288	H3K27me3	BF
chr1	142270730	142270731	chr1_142269835_142270820	-4.952488	-16.506135	1.8315E-07	0.003375288	H3K27me3	BF
chr1	142244555	142244556	chr1_142244437_142245373	-3.806254	-14.115061	6.1686E-07	0.007578913	H3K27me3	BF
chr1	255519996	255519997	chr1_255519859_255520363	-2.7017357	-12.23921	1.8442E-06	0.016993997	H3K27me3	BF
chr1	263719944	263719945	chr1_263719671_263720104	-5.317978	-10.773729	4.8532E-06	0.020249867	H3K27me3	BF

chr1	142279025	142279026	chr1_142279010_142279382	-3.9610558	-10.747106	4.9445E-06	0.020249867	H3K27me3	BF
chr1	142279193	142279194	chr1_142279010_142279382	-3.9610558	-10.747106	4.9445E-06	0.020249867	H3K27me3	BF
chr1	142279314	142279315	chr1_142279010_142279382	-3.9610558	-10.747106	4.9445E-06	0.020249867	H3K27me3	BF
chr1	142279326	142279327	chr1_142279010_142279382	-3.9610558	-10.747106	4.9445E-06	0.020249867	H3K27me3	BF
chr2	13525002	13525003	chr2_13524700_13525204	-13.23816	-16.754441	1.6302E-07	0.00427546	H3K27me3	BF
chr2	71987566	71987567	chr2_71986523_71988691	-3.3487476	-12.579289	1.4956E-06	0.005603738	H3K27me3	BF
chr2	71987758	71987759	chr2_71986523_71988691	-3.3487476	-12.579289	1.4956E-06	0.005603738	H3K27me3	BF
chr2	71987858	71987859	chr2_71986523_71988691	-3.3487476	-12.579289	1.4956E-06	0.005603738	H3K27me3	BF
chr2	71988040	71988041	chr2_71986523_71988691	-3.3487476	-12.579289	1.4956E-06	0.005603738	H3K27me3	BF
chr2	71988041	71988042	chr2_71986523_71988691	-3.3487476	-12.579289	1.4956E-06	0.005603738	H3K27me3	BF
chr2	71988759	71988760	chr2_71986523_71988691	-3.3487476	-12.579289	1.4956E-06	0.005603738	H3K27me3	BF
chr2	10463737	10463738	chr2_10463757_10464017	-3.865664	-10.891835	4.4702E-06	0.01001927	H3K27me3	BF
chr2	12364110	12364111	chr2_12363898_12365147	-3.426553	-10.740835	4.9663E-06	0.01001927	H3K27me3	BF

chr2	12364201	12364202	chr2_12363898_12365147	-3.426553	-10.740835	4.9663E-06	0.01001927	H3K27me3	BF
chr2	12364211	12364212	chr2_12363898_12365147	-3.426553	-10.740835	4.9663E-06	0.01001927	H3K27me3	BF
chr2	12365120	12365121	chr2_12363898_12365147	-3.426553	-10.740835	4.9663E-06	0.01001927	H3K27me3	BF
chr2	12365246	12365247	chr2_12363898_12365147	-3.426553	-10.740835	4.9663E-06	0.01001927	H3K27me3	BF
chr2	72067271	72067272	chr2_72066722_72068090	-3.245026	-10.521197	5.8015E-06	0.010868208	H3K27me3	BF
chr2	71952558	71952559	chr2_71952524_71953399	-3.746612	-10.057025	8.1352E-06	0.012550755	H3K27me3	BF
chr2	71952756	71952757	chr2_71952524_71953399	-3.746612	-10.057025	8.1352E-06	0.012550755	H3K27me3	BF
chr2	71953051	71953052	chr2_71952524_71953399	-3.746612	-10.057025	8.1352E-06	0.012550755	H3K27me3	BF
chr3	57160551	57160552	chr3_57159512_57160931	-3.1031154	-11.634457	2.7129E-06	0.014614831	H3K27me3	BF
chr3	57160586	57160587	chr3_57159512_57160931	-3.1031154	-11.634457	2.7129E-06	0.014614831	H3K27me3	BF
chr3	57160662	57160663	chr3_57159512_57160931	-3.1031154	-11.634457	2.7129E-06	0.014614831	H3K27me3	BF
chr3	57160667	57160668	chr3_57159512_57160931	-3.1031154	-11.634457	2.7129E-06	0.014614831	H3K27me3	BF
NW_018085011.1	62849	62850	NW_018085011.1_62154_63122	-2.865268	-11.017791	4.0985E-06	0.017873157	H3K27me3	BF

chr3	41843932	41843933	chr3_41844032_41844341	-4.915672	-10.20396	7.299E-06	0.017873157	H3K27me3	BF
chr3	41844004	41844005	chr3_41844032_41844341	-4.915672	-10.20396	7.299E-06	0.017873157	H3K27me3	BF
chr3	41844057	41844058	chr3_41844032_41844341	-4.915672	-10.20396	7.299E-06	0.017873157	H3K27me3	BF
chr3	41844149	41844150	chr3_41844032_41844341	-4.915672	-10.20396	7.299E-06	0.017873157	H3K27me3	BF
chr3	41844158	41844159	chr3_41844032_41844341	-4.915672	-10.20396	7.299E-06	0.017873157	H3K27me3	BF
chr4	81222845	81222846	chr4_81222840_81224001	-4.0248	-12.345895	1.7259E-06	0.028922234	H3K27me3	BF
chr4	101211795	101211796	chr4_101210858_101212777	-5.33609	-11.984681	2.1648E-06	0.028922234	H3K27me3	BF
chr4	75756843	75756844	chr4_75756303_75757631	2.4908674	11.2636359	3.4685E-06	0.030892416	H3K27me3	BF
NW_018085127.1	6067	6068	NW_018085127.1_4078_6141	-6.18374	-11.513997	2.936E-06	0.005972343	H3K27me3	BF
NW_018085127.1	5976	5977	NW_018085127.1_4078_6141	-6.18374	-11.513997	2.936E-06	0.005972343	H3K27me3	BF
NW_018085127.1	5851	5852	NW_018085127.1_4078_6141	-6.18374	-11.513997	2.936E-06	0.005972343	H3K27me3	BF
NW_018085127.1	5103	5104	NW_018085127.1_4078_6141	-6.18374	-11.513997	2.936E-06	0.005972343	H3K27me3	BF
NW_018085127.1	4988	4989	NW_018085127.1_4078_6141	-6.18374	-11.513997	2.936E-06	0.005972343	H3K27me3	BF

NW_018085127.1	4572	4573	NW_018085127.1_4078_6141	6.18374	11.5139968	2.936E-06	0.005972343	H3K27me3	BF
NW_018085127.1	4534	4535	NW_018085127.1_4078_6141	-6.18374	-11.513997	2.936E-06	0.005972343	H3K27me3	BF
NW_018085127.1	4481	4482	NW_018085127.1_4078_6141	6.18374	11.5139968	2.936E-06	0.005972343	H3K27me3	BF
NW_018085127.1	4426	4427	NW_018085127.1_4078_6141	6.18374	11.5139968	2.936E-06	0.005972343	H3K27me3	BF
NW_018085127.1	4196	4197	NW_018085127.1_4078_6141	-6.18374	-11.513997	2.936E-06	0.005972343	H3K27me3	BF
NW_018085127.1	4164	4165	NW_018085127.1_4078_6141	-6.18374	-11.513997	2.936E-06	0.005972343	H3K27me3	BF
chr5	61089587	61089588	chr5_61088030_61089801	-4.9063914	-10.136993	7.6676E-06	0.010723095	H3K27me3	BF
chr5	61089451	61089452	chr5_61088030_61089801	-4.9063914	-10.136993	7.6676E-06	0.010723095	H3K27me3	BF
chr5	61089288	61089289	chr5_61088030_61089801	-4.9063914	-10.136993	7.6676E-06	0.010723095	H3K27me3	BF
chr5	61088947	61088948	chr5_61088030_61089801	-4.9063914	-10.136993	7.6676E-06	0.010723095	H3K27me3	BF
chr5	61088323	61088324	chr5_61088030_61089801	-4.9063914	-10.136993	7.6676E-06	0.010723095	H3K27me3	BF
NW_018084880.1	4049	4050	NW_018084880.1_4017_4418	-5.910954	-10.003766	8.4644E-06	0.011141081	H3K27me3	BF
chr6	5659532	5659533	chr6_5659565_5659860	-5.0263202	-14.728042	4.4403E-07	0.001061776	H3K27me3	BF

chr6	5659536	5659537	chr6_5659565_5659860	-5.0263202	-14.728042	4.4403E-07	0.001061776	H3K27me3	BF
chr6	5659609	5659610	chr6_5659565_5659860	-5.0263202	-14.728042	4.4403E-07	0.001061776	H3K27me3	BF
chr6	5659643	5659644	chr6_5659565_5659860	-5.0263202	-14.728042	4.4403E-07	0.001061776	H3K27me3	BF
chr6	5659689	5659690	chr6_5659565_5659860	-5.0263202	-14.728042	4.4403E-07	0.001061776	H3K27me3	BF
chr6	5659789	5659790	chr6_5659565_5659860	-5.0263202	-14.728042	4.4403E-07	0.001061776	H3K27me3	BF
chr6	5659801	5659802	chr6_5659565_5659860	-5.0263202	-14.728042	4.4403E-07	0.001061776	H3K27me3	BF
chr6	5659815	5659816	chr6_5659565_5659860	-5.0263202	-14.728042	4.4403E-07	0.001061776	H3K27me3	BF
chr6	5659959	5659960	chr6_5659565_5659860	-5.0263202	-14.728042	4.4403E-07	0.001061776	H3K27me3	BF
chr6	72892043	72892044	chr6_72892086_72892877	-5.4202436	-13.702334	7.7554E-07	0.001061776	H3K27me3	BF
chr6	72892188	72892189	chr6_72892086_72892877	-5.4202436	-13.702334	7.7554E-07	0.001061776	H3K27me3	BF
chr6	72892195	72892196	chr6_72892086_72892877	-5.4202436	-13.702334	7.7554E-07	0.001061776	H3K27me3	BF
chr6	72892320	72892321	chr6_72892086_72892877	-5.4202436	-13.702334	7.7554E-07	0.001061776	H3K27me3	BF
chr6	72892338	72892339	chr6_72892086_72892877	-5.4202436	-13.702334	7.7554E-07	0.001061776	H3K27me3	BF

chr6	72892419	72892420	chr6_72892086_72892877	-5.4202436	-13.702334	7.7554E-07	0.001061776	H3K27me3	BF
chr6	72892448	72892449	chr6_72892086_72892877	-5.4202436	-13.702334	7.7554E-07	0.001061776	H3K27me3	BF
chr6	72892463	72892464	chr6_72892086_72892877	-5.4202436	-13.702334	7.7554E-07	0.001061776	H3K27me3	BF
chr6	72892481	72892482	chr6_72892086_72892877	-5.4202436	-13.702334	7.7554E-07	0.001061776	H3K27me3	BF
chr6	72892504	72892505	chr6_72892086_72892877	-5.4202436	-13.702334	7.7554E-07	0.001061776	H3K27me3	BF
chr6	72892602	72892603	chr6_72892086_72892877	-5.4202436	-13.702334	7.7554E-07	0.001061776	H3K27me3	BF
chr6	72892610	72892611	chr6_72892086_72892877	-5.4202436	-13.702334	7.7554E-07	0.001061776	H3K27me3	BF
chr6	72892974	72892975	chr6_72892086_72892877	-5.4202436	-13.702334	7.7554E-07	0.001061776	H3K27me3	BF
chr6	72892977	72892978	chr6_72892086_72892877	-5.4202436	-13.702334	7.7554E-07	0.001061776	H3K27me3	BF
chr6	76580739	76580740	chr6_76580623_76582367	-2.327792	-11.112778	3.841E-06	0.00483802	H3K27me3	BF
chr6	76582366	76582367	chr6_76580623_76582367	-2.327792	-11.112778	3.841E-06	0.00483802	H3K27me3	BF
chr6	10694991	10694992	chr6_10694432_10695103	-6.0838536	-10.601392	5.4796E-06	0.00663639	H3K27me3	BF
chr7	109075672	109075673	chr7_109075423_109075725	-1.9053919	-13.736309	7.6088E-07	0.002655917	H3K27me3	BF

chr7	109075726	109075727	chr7_109075423_109075725	-1.9053919	-13.736309	7.6088E-07	0.002655917	H3K27me3	BF
NW_018085303.1	467467	467468	NW_018085303.1_466451_467683	-3.5048082	-11.640638	2.702E-06	0.002655917	H3K27me3	BF
NW_018085303.1	467456	467457	NW_018085303.1_466451_467683	-3.5048082	-11.640638	2.702E-06	0.002655917	H3K27me3	BF
NW_018085303.1	467409	467410	NW_018085303.1_466451_467683	-3.5048082	-11.640638	2.702E-06	0.002655917	H3K27me3	BF
NW_018085303.1	467356	467357	NW_018085303.1_466451_467683	-3.5048082	-11.640638	2.702E-06	0.002655917	H3K27me3	BF
NW_018085303.1	467340	467341	NW_018085303.1_466451_467683	-3.5048082	-11.640638	2.702E-06	0.002655917	H3K27me3	BF
NW_018085303.1	467277	467278	NW_018085303.1_466451_467683	-3.5048082	-11.640638	2.702E-06	0.002655917	H3K27me3	BF
NW_018085303.1	467262	467263	NW_018085303.1_466451_467683	-3.5048082	-11.640638	2.702E-06	0.002655917	H3K27me3	BF
NW_018085303.1	467219	467220	NW_018085303.1_466451_467683	-3.5048082	-11.640638	2.702E-06	0.002655917	H3K27me3	BF
NW_018085303.1	467168	467169	NW_018085303.1_466451_467683	-3.5048082	-11.640638	2.702E-06	0.002655917	H3K27me3	BF
NW_018085303.1	467162	467163	NW_018085303.1_466451_467683	-3.5048082	-11.640638	2.702E-06	0.002655917	H3K27me3	BF
NW_018085303.1	467077	467078	NW_018085303.1_466451_467683	-3.5048082	-11.640638	2.702E-06	0.002655917	H3K27me3	BF
NW_018085303.1	467047	467048	NW_018085303.1_466451_467683	-3.5048082	-11.640638	2.702E-06	0.002655917	H3K27me3	BF

NW_018085303.1	467023	467024	NW_018085303.1_466451_467683	-3.5048082	-11.640638	2.702E-06	0.002655917	H3K27me3	BF
NW_018085303.1	466530	466531	NW_018085303.1_466451_467683	-3.5048082	-11.640638	2.702E-06	0.002655917	H3K27me3	BF
NW_018085303.1	466514	466515	NW_018085303.1_466451_467683	-3.5048082	-11.640638	2.702E-06	0.002655917	H3K27me3	BF
NW_018085303.1	466507	466508	NW_018085303.1_466451_467683	-3.5048082	-11.640638	2.702E-06	0.002655917	H3K27me3	BF
NW_018085303.1	466470	466471	NW_018085303.1_466451_467683	-3.5048082	-11.640638	2.702E-06	0.002655917	H3K27me3	BF
NW_018085303.1	466456	466457	NW_018085303.1_466451_467683	-3.5048082	-11.640638	2.702E-06	0.002655917	H3K27me3	BF
NW_018085303.1	466450	466451	NW_018085303.1_466451_467683	-3.5048082	-11.640638	2.702E-06	0.002655917	H3K27me3	BF
NW_018085303.1	466444	466445	NW_018085303.1_466451_467683	-3.5048082	-11.640638	2.702E-06	0.002655917	H3K27me3	BF
NW_018085303.1	466435	466436	NW_018085303.1_466451_467683	-3.5048082	-11.640638	2.702E-06	0.002655917	H3K27me3	BF
NW_018085303.1	466404	466405	NW_018085303.1_466451_467683	-3.5048082	-11.640638	2.702E-06	0.002655917	H3K27me3	BF
NW_018085303.1	466401	466402	NW_018085303.1_466451_467683	-3.5048082	-11.640638	2.702E-06	0.002655917	H3K27me3	BF
NW_018085303.1	466378	466379	NW_018085303.1_466451_467683	-3.5048082	-11.640638	2.702E-06	0.002655917	H3K27me3	BF
chr7	54235496	54235497	chr7_54235430_54235995	-5.802592	-10.942669	4.3158E-06	0.003803425	H3K27me3	BF

chr7	54235803	54235804	chr7_54235430_54235995	-5.802592	-10.942669	4.3158E-06	0.003803425	H3K27me3	BF
chr7	54235861	54235862	chr7_54235430_54235995	-5.802592	-10.942669	4.3158E-06	0.003803425	H3K27me3	BF
chr8	5364723	5364724	chr8_5364723_5364892	-2.9290067	-11.642401	2.6988E-06	0.020939454	H3K27me3	BF
chr8	5364784	5364785	chr8_5364723_5364892	-2.9290067	-11.642401	2.6988E-06	0.020939454	H3K27me3	BF
chr8	5364946	5364947	chr8_5364723_5364892	-2.9290067	-11.642401	2.6988E-06	0.020939454	H3K27me3	BF
chr9	4607888	4607889	chr9_4607785_4608504	-4.846547	-15.013997	3.8257E-07	0.011173629	H3K27me3	BF
chr10	21569619	21569620	chr10_21569655_21570415	-2.5591114	-14.16043	6.0176E-07	0.00869841	H3K4me1	BF
chr10	21570149	21570150	chr10_21569655_21570415	-2.5591114	-14.16043	6.0176E-07	0.00869841	H3K4me1	BF
chr10	38467761	38467762	chr10_38467547_38468160	-2.9419452	-10.676368	5.1966E-06	0.026657654	H3K4me1	BF
chr10	38468159	38468160	chr10_38467547_38468160	-2.9419452	-10.676368	5.1966E-06	0.026657654	H3K4me1	BF
chr10	17449997	17449998	chr10_17449414_17450795	-0.8741353	-10.372741	6.4546E-06	0.026657654	H3K4me1	BF
chr10	17450110	17450111	chr10_17449414_17450795	-0.8741353	-10.372741	6.4546E-06	0.026657654	H3K4me1	BF
chr10	17450260	17450261	chr10_17449414_17450795	-0.8741353	-10.372741	6.4546E-06	0.026657654	H3K4me1	BF

chr10	9667075	9667076	chr10_9667142_9668379	-2.182879	-9.8125364	9.7746E-06	0.028258274	H3K4me1	BF
chr10	9667739	9667740	chr10_9667142_9668379	-2.182879	-9.8125364	9.7746E-06	0.028258274	H3K4me1	BF
chr10	9668439	9668440	chr10_9667142_9668379	-2.182879	-9.8125364	9.7746E-06	0.028258274	H3K4me1	BF
chr11	20849506	20849507	chr11_20848313_20849743	-1.894369	-21.70293	2.1416E-08	0.000260285	H3K4me1	BF
chr11	5027204	5027205	chr11_5026878_5027747	-3.8814454	-19.670239	4.6424E-08	0.000260285	H3K4me1	BF
chr11	5027326	5027327	chr11_5026878_5027747	-3.8814454	-19.670239	4.6424E-08	0.000260285	H3K4me1	BF
chr11	5027663	5027664	chr11_5026878_5027747	-3.8814454	-19.670239	4.6424E-08	0.000260285	H3K4me1	BF
chr11	20657334	20657335	chr11_20657352_20658291	-3.02505	-12.4531	1.6156E-06	0.002264511	H3K4me1	BF
chr11	20657411	20657412	chr11_20657352_20658291	-3.02505	-12.4531	1.6156E-06	0.002264511	H3K4me1	BF
chr11	20657412	20657413	chr11_20657352_20658291	-3.02505	-12.4531	1.6156E-06	0.002264511	H3K4me1	BF
chr11	20657457	20657458	chr11_20657352_20658291	-3.02505	-12.4531	1.6156E-06	0.002264511	H3K4me1	BF
chr11	20657460	20657461	chr11_20657352_20658291	-3.02505	-12.4531	1.6156E-06	0.002264511	H3K4me1	BF
chr11	20657474	20657475	chr11_20657352_20658291	-3.02505	-12.4531	1.6156E-06	0.002264511	H3K4me1	BF

chr11	20657542	20657543	chr11_20657352_20658291	-3.02505	-12.4531	1.6156E-06	0.002264511	H3K4me1	BF
chr11	20657909	20657910	chr11_20657352_20658291	-3.02505	-12.4531	1.6156E-06	0.002264511	H3K4me1	BF
chr11	20657951	20657952	chr11_20657352_20658291	-3.02505	-12.4531	1.6156E-06	0.002264511	H3K4me1	BF
chr11	20658031	20658032	chr11_20657352_20658291	-3.02505	-12.4531	1.6156E-06	0.002264511	H3K4me1	BF
chr11	20658036	20658037	chr11_20657352_20658291	-3.02505	-12.4531	1.6156E-06	0.002264511	H3K4me1	BF
chr11	20658370	20658371	chr11_20657352_20658291	-3.02505	-12.4531	1.6156E-06	0.002264511	H3K4me1	BF
chr11	48444723	48444724	chr11_48444444_48445266	-1.6307488	-11.291352	3.4045E-06	0.004241817	H3K4me1	BF
chr11	48445027	48445028	chr11_48444444_48445266	-1.6307488	-11.291352	3.4045E-06	0.004241817	H3K4me1	BF
chr11	66308114	66308115	chr11_66307960_66310434	1.3736	10.2878843	6.8646E-06	0.00769767	H3K4me1	BF
chr11	66309972	66309973	chr11_66307960_66310434	1.3736	10.2878843	6.8646E-06	0.00769767	H3K4me1	BF
chr12	25409851	25409852	chr12_25409847_25411030	-3.1364258	-12.303613	1.7718E-06	0.005625554	H3K4me1	BF
chr12	25409861	25409862	chr12_25409847_25411030	-3.1364258	-12.303613	1.7718E-06	0.005625554	H3K4me1	BF
chr12	25409890	25409891	chr12_25409847_25411030	-3.1364258	-12.303613	1.7718E-06	0.005625554	H3K4me1	BF

chr12	25410061	25410062	chr12_25409847_25411030	-3.1364258	-12.303613	1.7718E-06	0.005625554	H3K4me1	BF
chr12	25410211	25410212	chr12_25409847_25411030	-3.1364258	-12.303613	1.7718E-06	0.005625554	H3K4me1	BF
chr12	25410215	25410216	chr12_25409847_25411030	-3.1364258	-12.303613	1.7718E-06	0.005625554	H3K4me1	BF
chr12	25410228	25410229	chr12_25409847_25411030	-3.1364258	-12.303613	1.7718E-06	0.005625554	H3K4me1	BF
chr12	25410234	25410235	chr12_25409847_25411030	-3.1364258	-12.303613	1.7718E-06	0.005625554	H3K4me1	BF
chr12	25410487	25410488	chr12_25409847_25411030	-3.1364258	-12.303613	1.7718E-06	0.005625554	H3K4me1	BF
chr12	15170712	15170713	chr12_15170223_15171088	-2.810786	-10.112352	7.8084E-06	0.018594384	H3K4me1	BF
chr12	15170739	15170740	chr12_15170223_15171088	-2.810786	-10.112352	7.8084E-06	0.018594384	H3K4me1	BF
chr12	15170749	15170750	chr12_15170223_15171088	-2.810786	-10.112352	7.8084E-06	0.018594384	H3K4me1	BF
chr13	132266480	132266481	chr13_132266539_132266883	-5.6073027	-19.198184	5.6177E-08	0.000803368	H3K4me1	BF
chr13	132266494	132266495	chr13_132266539_132266883	-5.6073027	-19.198184	5.6177E-08	0.000803368	H3K4me1	BF
chr13	132266506	132266507	chr13_132266539_132266883	-5.6073027	-19.198184	5.6177E-08	0.000803368	H3K4me1	BF
chr13	10873932	10873933	chr13_10873764_10874463	-3.2017058	-14.184474	5.9392E-07	0.00637013	H3K4me1	BF

chr13	196740259	196740260	chr13_196739497_196740765	-3.0564723	-11.724837	2.5579E-06	0.01271047	H3K4me1	BF
chr13	196740360	196740361	chr13_196739497_196740765	-3.0564723	-11.724837	2.5579E-06	0.01271047	H3K4me1	BF
chr13	196740384	196740385	chr13_196739497_196740765	-3.0564723	-11.724837	2.5579E-06	0.01271047	H3K4me1	BF
chr13	13114249	13114250	chr13_13113780_13114879	-1.6371824	-10.561679	5.6364E-06	0.01271047	H3K4me1	BF
chr13	13114347	13114348	chr13_13113780_13114879	-1.6371824	-10.561679	5.6364E-06	0.01271047	H3K4me1	BF
chr13	13114363	13114364	chr13_13113780_13114879	-1.6371824	-10.561679	5.6364E-06	0.01271047	H3K4me1	BF
chr13	13114379	13114380	chr13_13113780_13114879	-1.6371824	-10.561679	5.6364E-06	0.01271047	H3K4me1	BF
chr13	13114385	13114386	chr13_13113780_13114879	-1.6371824	-10.561679	5.6364E-06	0.01271047	H3K4me1	BF
chr13	13114427	13114428	chr13_13113780_13114879	-1.6371824	-10.561679	5.6364E-06	0.01271047	H3K4me1	BF
chr13	13114903	13114904	chr13_13113780_13114879	-1.6371824	-10.561679	5.6364E-06	0.01271047	H3K4me1	BF
chr13	152347212	152347213	chr13_152347162_152348201	2.5131923	10.3092621	6.7587E-06	0.01271047	H3K4me1	BF
chr13	152347274	152347275	chr13_152347162_152348201	2.5131923	10.3092621	6.7587E-06	0.01271047	H3K4me1	BF
chr13	152347733	152347734	chr13_152347162_152348201	2.5131923	10.3092621	6.7587E-06	0.01271047	H3K4me1	BF

chr13	152347810	152347811	chr13_152347162_152348201	2.5131923	10.3092621	6.7587E-06	0.01271047	H3K4me1	BF
chr13	152347841	152347842	chr13_152347162_152348201	2.5131923	10.3092621	6.7587E-06	0.01271047	H3K4me1	BF
chr13	152347846	152347847	chr13_152347162_152348201	2.5131923	10.3092621	6.7587E-06	0.01271047	H3K4me1	BF
chr13	133107428	133107429	chr13_133107172_133108810	-1.8375113	-10.239688	7.1104E-06	0.01271047	H3K4me1	BF
chr13	133107745	133107746	chr13_133107172_133108810	-1.8375113	-10.239688	7.1104E-06	0.01271047	H3K4me1	BF
chr13	133108373	133108374	chr13_133107172_133108810	-1.8375113	-10.239688	7.1104E-06	0.01271047	H3K4me1	BF
chr13	133108395	133108396	chr13_133107172_133108810	-1.8375113	-10.239688	7.1104E-06	0.01271047	H3K4me1	BF
chr14	109761110	109761111	chr14_109760736_109761084	4.5418133	19.5237304	4.9231E-08	0.001537371	H3K4me1	BF
chr14	58393043	58393044	chr14_58393027_58393846	-2.3676851	-14.528543	4.9347E-07	0.001537371	H3K4me1	BF
chr14	58393304	58393305	chr14_58393027_58393846	-2.3676851	-14.528543	4.9347E-07	0.001537371	H3K4me1	BF
chr14	58393354	58393355	chr14_58393027_58393846	-2.3676851	-14.528543	4.9347E-07	0.001537371	H3K4me1	BF
chr14	58393362	58393363	chr14_58393027_58393846	-2.3676851	-14.528543	4.9347E-07	0.001537371	H3K4me1	BF
chr14	58393391	58393392	chr14_58393027_58393846	-2.3676851	-14.528543	4.9347E-07	0.001537371	H3K4me1	BF

chr14	58393416	58393417	chr14_58393027_58393846	-2.3676851	-14.528543	4.9347E-07	0.001537371	H3K4me1	BF
chr14	58393437	58393438	chr14_58393027_58393846	-2.3676851	-14.528543	4.9347E-07	0.001537371	H3K4me1	BF
chr14	73959706	73959707	chr14_73959429_73960971	-3.6381204	-14.014556	6.5184E-07	0.001537371	H3K4me1	BF
chr14	73959765	73959766	chr14_73959429_73960971	-3.6381204	-14.014556	6.5184E-07	0.001537371	H3K4me1	BF
chr14	73959971	73959972	chr14_73959429_73960971	-3.6381204	-14.014556	6.5184E-07	0.001537371	H3K4me1	BF
chr14	73960010	73960011	chr14_73959429_73960971	-3.6381204	-14.014556	6.5184E-07	0.001537371	H3K4me1	BF
chr14	73960076	73960077	chr14_73959429_73960971	-3.6381204	-14.014556	6.5184E-07	0.001537371	H3K4me1	BF
chr14	73960291	73960292	chr14_73959429_73960971	-3.6381204	-14.014556	6.5184E-07	0.001537371	H3K4me1	BF
chr14	73960387	73960388	chr14_73959429_73960971	-3.6381204	-14.014556	6.5184E-07	0.001537371	H3K4me1	BF
chr14	73960569	73960570	chr14_73959429_73960971	-3.6381204	-14.014556	6.5184E-07	0.001537371	H3K4me1	BF
chr14	91197022	91197023	chr14_91195958_91197547	3.5419692	13.6127557	8.1573E-07	0.001539116	H3K4me1	BF
chr14	91197035	91197036	chr14_91195958_91197547	3.5419692	13.6127557	8.1573E-07	0.001539116	H3K4me1	BF
chr14	91197407	91197408	chr14_91195958_91197547	3.5419692	13.6127557	8.1573E-07	0.001539116	H3K4me1	BF

chr14	91197444	91197445	chr14_91195958_91197547	3.5419692	13.6127557	8.1573E-07	0.001539116	H3K4me1	BF
chr14	123771614	123771615	chr14_123771441_123772561	-4.1154213	-12.69517	1.3942E-06	0.002192207	H3K4me1	BF
chr14	123771841	123771842	chr14_123771441_123772561	-4.1154213	-12.69517	1.3942E-06	0.002192207	H3K4me1	BF
chr14	123771933	123771934	chr14_123771441_123772561	-4.1154213	-12.69517	1.3942E-06	0.002192207	H3K4me1	BF
chr14	123772164	123772165	chr14_123771441_123772561	-4.1154213	-12.69517	1.3942E-06	0.002192207	H3K4me1	BF
chr14	85954900	85954901	chr14_85954777_85955227	-2.7119124	-12.015006	2.1235E-06	0.00296793	H3K4me1	BF
chr14	85954901	85954902	chr14_85954777_85955227	-2.7119124	-12.015006	2.1235E-06	0.00296793	H3K4me1	BF
chr14	85954959	85954960	chr14_85954777_85955227	-2.7119124	-12.015006	2.1235E-06	0.00296793	H3K4me1	BF
chr14	2991773	2991774	chr14_2991793_2992409	3.6647263	11.8701727	2.3291E-06	0.003030671	H3K4me1	BF
chr14	2991775	2991776	chr14_2991793_2992409	3.6647263	11.8701727	2.3291E-06	0.003030671	H3K4me1	BF
chr14	82115127	82115128	chr14_82114175_82116056	4.6455799	11.2601052	3.4767E-06	0.00437323	H3K4me1	BF
chr14	128761240	128761241	chr14_128761274_128762157	-1.9088207	-10.487198	5.9443E-06	0.00451793	H3K4me1	BF
chr14	128761248	128761249	chr14_128761274_128762157	-1.9088207	-10.487198	5.9443E-06	0.00451793	H3K4me1	BF

chr14	128761311	128761312	chr14_128761274_128762157	-1.9088207	-10.487198	5.9443E-06	0.00451793	H3K4me1	BF
chr14	128761332	128761333	chr14_128761274_128762157	-1.9088207	-10.487198	5.9443E-06	0.00451793	H3K4me1	BF
chr14	128761417	128761418	chr14_128761274_128762157	-1.9088207	-10.487198	5.9443E-06	0.00451793	H3K4me1	BF
chr14	128761440	128761441	chr14_128761274_128762157	-1.9088207	-10.487198	5.9443E-06	0.00451793	H3K4me1	BF
chr14	128761452	128761453	chr14_128761274_128762157	-1.9088207	-10.487198	5.9443E-06	0.00451793	H3K4me1	BF
chr14	128761456	128761457	chr14_128761274_128762157	-1.9088207	-10.487198	5.9443E-06	0.00451793	H3K4me1	BF
chr14	128761491	128761492	chr14_128761274_128762157	-1.9088207	-10.487198	5.9443E-06	0.00451793	H3K4me1	BF
chr14	128761517	128761518	chr14_128761274_128762157	-1.9088207	-10.487198	5.9443E-06	0.00451793	H3K4me1	BF
chr14	128761538	128761539	chr14_128761274_128762157	-1.9088207	-10.487198	5.9443E-06	0.00451793	H3K4me1	BF
chr14	128761548	128761549	chr14_128761274_128762157	-1.9088207	-10.487198	5.9443E-06	0.00451793	H3K4me1	BF
chr14	128761604	128761605	chr14_128761274_128762157	-1.9088207	-10.487198	5.9443E-06	0.00451793	H3K4me1	BF
chr14	128761678	128761679	chr14_128761274_128762157	-1.9088207	-10.487198	5.9443E-06	0.00451793	H3K4me1	BF
chr14	128761714	128761715	chr14_128761274_128762157	-1.9088207	-10.487198	5.9443E-06	0.00451793	H3K4me1	BF

chr14	128761922	128761923	chr14_128761274_128762157	-1.9088207	-10.487198	5.9443E-06	0.00451793	H3K4me1	BF
chr14	134044053	134044054	chr14_134043967_134044964	5.0871624	10.3682542	6.4756E-06	0.00451793	H3K4me1	BF
chr14	134044178	134044179	chr14_134043967_134044964	5.0871624	10.3682542	6.4756E-06	0.00451793	H3K4me1	BF
chr14	134044975	134044976	chr14_134043967_134044964	5.0871624	10.3682542	6.4756E-06	0.00451793	H3K4me1	BF
chr14	43353431	43353432	chr14_43352820_43355365	-1.9671333	-10.331709	6.6494E-06	0.00451793	H3K4me1	BF
chr14	43354447	43354448	chr14_43352820_43355365	-1.9671333	-10.331709	6.6494E-06	0.00451793	H3K4me1	BF
chr14	43354693	43354694	chr14_43352820_43355365	-1.9671333	-10.331709	6.6494E-06	0.00451793	H3K4me1	BF
chr14	43354741	43354742	chr14_43352820_43355365	-1.9671333	-10.331709	6.6494E-06	0.00451793	H3K4me1	BF
chr14	73476097	73476098	chr14_73476163_73477002	1.8824217	9.90329574	9.1265E-06	0.00451793	H3K4me1	BF
chr14	73476105	73476106	chr14_73476163_73477002	1.8824217	9.90329574	9.1265E-06	0.00451793	H3K4me1	BF
chr14	73476770	73476771	chr14_73476163_73477002	1.8824217	9.90329574	9.1265E-06	0.00451793	H3K4me1	BF
chr14	73476865	73476866	chr14_73476163_73477002	1.8824217	9.90329574	9.1265E-06	0.00451793	H3K4me1	BF
chr14	73476915	73476916	chr14_73476163_73477002	1.8824217	9.90329574	9.1265E-06	0.00451793	H3K4me1	BF

chr14	73476973	73476974	chr14_73476163_73477002	1.8824217	9.90329574	9.1265E-06	0.00451793	H3K4me1	BF
chr14	141278414	141278415	chr14_141278319_141280329	-6.3895329	-9.8559658	9.4582E-06	0.00451793	H3K4me1	BF
chr14	141278452	141278453	chr14_141278319_141280329	-6.3895329	-9.8559658	9.4582E-06	0.00451793	H3K4me1	BF
chr14	141278554	141278555	chr14_141278319_141280329	-6.3895329	-9.8559658	9.4582E-06	0.00451793	H3K4me1	BF
chr14	141279384	141279385	chr14_141278319_141280329	-6.3895329	-9.8559658	9.4582E-06	0.00451793	H3K4me1	BF
chr14	141279404	141279405	chr14_141278319_141280329	-6.3895329	-9.8559658	9.4582E-06	0.00451793	H3K4me1	BF
chr14	141279482	141279483	chr14_141278319_141280329	-6.3895329	-9.8559658	9.4582E-06	0.00451793	H3K4me1	BF
chr14	141279488	141279489	chr14_141278319_141280329	-6.3895329	-9.8559658	9.4582E-06	0.00451793	H3K4me1	BF
chr14	141279551	141279552	chr14_141278319_141280329	-6.3895329	-9.8559658	9.4582E-06	0.00451793	H3K4me1	BF
chr14	141279723	141279724	chr14_141278319_141280329	-6.3895329	-9.8559658	9.4582E-06	0.00451793	H3K4me1	BF
chr14	141279745	141279746	chr14_141278319_141280329	-6.3895329	-9.8559658	9.4582E-06	0.00451793	H3K4me1	BF
chr14	141279900	141279901	chr14_141278319_141280329	-6.3895329	-9.8559658	9.4582E-06	0.00451793	H3K4me1	BF
chr14	141280176	141280177	chr14_141278319_141280329	-6.3895329	-9.8559658	9.4582E-06	0.00451793	H3K4me1	BF

chr14	141280184	141280185	chr14_141278319_141280329	-6.3895329	-9.8559658	9.4582E-06	0.00451793	H3K4me1	BF
chr14	141280205	141280206	chr14_141278319_141280329	-6.3895329	-9.8559658	9.4582E-06	0.00451793	H3K4me1	BF
chr14	141280238	141280239	chr14_141278319_141280329	-6.3895329	-9.8559658	9.4582E-06	0.00451793	H3K4me1	BF
chr14	141280261	141280262	chr14_141278319_141280329	-6.3895329	-9.8559658	9.4582E-06	0.00451793	H3K4me1	BF
chr14	141280269	141280270	chr14_141278319_141280329	-6.3895329	-9.8559658	9.4582E-06	0.00451793	H3K4me1	BF
chr14	141280329	141280330	chr14_141278319_141280329	-6.3895329	-9.8559658	9.4582E-06	0.00451793	H3K4me1	BF
chr14	141280344	141280345	chr14_141278319_141280329	-6.3895329	-9.8559658	9.4582E-06	0.00451793	H3K4me1	BF
chr14	141280388	141280389	chr14_141278319_141280329	-6.3895329	-9.8559658	9.4582E-06	0.00451793	H3K4me1	BF
chr15	52228045	52228046	chr15_52226497_52228432	-2.3817724	-13.787086	7.3954E-07	0.005543613	H3K4me1	BF
chr15	131571308	131571309	chr15_131570950_131572460	-5.8410529	-13.650586	7.9848E-07	0.005543613	H3K4me1	BF
chr15	131571601	131571602	chr15_131570950_131572460	-5.8410529	-13.650586	7.9848E-07	0.005543613	H3K4me1	BF
chr15	31898317	31898318	chr15_31898402_31899704	-8.878903	-13.376845	9.3322E-07	0.005543613	H3K4me1	BF
chr15	31898400	31898401	chr15_31898402_31899704	-8.878903	-13.376845	9.3322E-07	0.005543613	H3K4me1	BF

chr15	31898864	31898865	chr15_31898402_31899704	-8.878903	-13.376845	9.3322E-07	0.005543613	H3K4me1	BF
chr15	47755105	47755106	chr15_47754544_47755356	-3.0008193	-13.003761	1.1598E-06	0.00565431	H3K4me1	BF
chr15	95740936	95740937	chr15_95740784_95741060	-2.9853107	-12.212071	1.8757E-06	0.00565431	H3K4me1	BF
chr15	66146531	66146532	chr15_66146068_66146961	-1.9555425	-11.942809	2.2233E-06	0.00565431	H3K4me1	BF
chr15	140313483	140313484	chr15_140313572_140313973	-3.2530455	-11.073676	3.9448E-06	0.00565431	H3K4me1	BF
chr15	140313536	140313537	chr15_140313572_140313973	-3.2530455	-11.073676	3.9448E-06	0.00565431	H3K4me1	BF
chr15	140313554	140313555	chr15_140313572_140313973	-3.2530455	-11.073676	3.9448E-06	0.00565431	H3K4me1	BF
chr15	140313578	140313579	chr15_140313572_140313973	-3.2530455	-11.073676	3.9448E-06	0.00565431	H3K4me1	BF
chr15	140313670	140313671	chr15_140313572_140313973	-3.2530455	-11.073676	3.9448E-06	0.00565431	H3K4me1	BF
chr15	140313699	140313700	chr15_140313572_140313973	-3.2530455	-11.073676	3.9448E-06	0.00565431	H3K4me1	BF
chr15	140313776	140313777	chr15_140313572_140313973	-3.2530455	-11.073676	3.9448E-06	0.00565431	H3K4me1	BF
chr15	140313907	140313908	chr15_140313572_140313973	-3.2530455	-11.073676	3.9448E-06	0.00565431	H3K4me1	BF
chr15	140314011	140314012	chr15_140313572_140313973	-3.2530455	-11.073676	3.9448E-06	0.00565431	H3K4me1	BF

chr15	140314033	140314034	chr15_140313572_140313973	-3.2530455	-11.073676	3.9448E-06	0.00565431	H3K4me1	BF
chr15	3501925	3501926	chr15_3501897_3503651	-3.4189163	-10.953628	4.2833E-06	0.00565431	H3K4me1	BF
chr15	3502430	3502431	chr15_3501897_3503651	-3.4189163	-10.953628	4.2833E-06	0.00565431	H3K4me1	BF
chr15	3502670	3502671	chr15_3501897_3503651	-3.4189163	-10.953628	4.2833E-06	0.00565431	H3K4me1	BF
chr15	3502699	3502700	chr15_3501897_3503651	-3.4189163	-10.953628	4.2833E-06	0.00565431	H3K4me1	BF
chr15	3502876	3502877	chr15_3501897_3503651	-3.4189163	-10.953628	4.2833E-06	0.00565431	H3K4me1	BF
chr15	3502943	3502944	chr15_3501897_3503651	-3.4189163	-10.953628	4.2833E-06	0.00565431	H3K4me1	BF
chr15	3503168	3503169	chr15_3501897_3503651	-3.4189163	-10.953628	4.2833E-06	0.00565431	H3K4me1	BF
chr15	3503262	3503263	chr15_3501897_3503651	-3.4189163	-10.953628	4.2833E-06	0.00565431	H3K4me1	BF
chr15	20173688	20173689	chr15_20173393_20174752	3.1720193	10.4792332	5.9783E-06	0.006873479	H3K4me1	BF
chr15	20173802	20173803	chr15_20173393_20174752	3.1720193	10.4792332	5.9783E-06	0.006873479	H3K4me1	BF
chr15	20174151	20174152	chr15_20173393_20174752	3.1720193	10.4792332	5.9783E-06	0.006873479	H3K4me1	BF
chr15	20174255	20174256	chr15_20173393_20174752	3.1720193	10.4792332	5.9783E-06	0.006873479	H3K4me1	BF

chr15	17993762	17993763	chr15_17993673_17995216	-2.2763672	-10.14612	7.6161E-06	0.007755826	H3K4me1	BF
chr15	17994049	17994050	chr15_17993673_17995216	-2.2763672	-10.14612	7.6161E-06	0.007755826	H3K4me1	BF
chr15	17994497	17994498	chr15_17993673_17995216	-2.2763672	-10.14612	7.6161E-06	0.007755826	H3K4me1	BF
chr15	17994513	17994514	chr15_17993673_17995216	-2.2763672	-10.14612	7.6161E-06	0.007755826	H3K4me1	BF
chr16	45164758	45164759	chr16_45164754_45165391	-2.351195	-27.740145	3.0773E-09	7.88648E-06	H3K4me1	BF
chr16	45164947	45164948	chr16_45164754_45165391	-2.351195	-27.740145	3.0773E-09	7.88648E-06	H3K4me1	BF
chr16	45165020	45165021	chr16_45164754_45165391	-2.351195	-27.740145	3.0773E-09	7.88648E-06	H3K4me1	BF
chr16	45165035	45165036	chr16_45164754_45165391	-2.351195	-27.740145	3.0773E-09	7.88648E-06	H3K4me1	BF
chr16	45165246	45165247	chr16_45164754_45165391	-2.351195	-27.740145	3.0773E-09	7.88648E-06	H3K4me1	BF
chr16	45165263	45165264	chr16_45164754_45165391	-2.351195	-27.740145	3.0773E-09	7.88648E-06	H3K4me1	BF
chr16	45165388	45165389	chr16_45164754_45165391	-2.351195	-27.740145	3.0773E-09	7.88648E-06	H3K4me1	BF
chr16	45165394	45165395	chr16_45164754_45165391	-2.351195	-27.740145	3.0773E-09	7.88648E-06	H3K4me1	BF
chr16	75938810	75938811	chr16_75938781_75939594	-2.324183	-14.988266	3.8769E-07	0.000794838	H3K4me1	BF

chr16	75939290	75939291	chr16_75938781_75939594	-2.324183	-14.988266	3.8769E-07	0.000794838	H3K4me1	BF
chr16	63433026	63433027	chr16_63433078_63433368	-3.439768	-12.190164	1.9016E-06	0.002051944	H3K4me1	BF
chr16	63433159	63433160	chr16_63433078_63433368	-3.439768	-12.190164	1.9016E-06	0.002051944	H3K4me1	BF
chr16	63433176	63433177	chr16_63433078_63433368	-3.439768	-12.190164	1.9016E-06	0.002051944	H3K4me1	BF
chr16	63433197	63433198	chr16_63433078_63433368	-3.439768	-12.190164	1.9016E-06	0.002051944	H3K4me1	BF
chr16	63433217	63433218	chr16_63433078_63433368	-3.439768	-12.190164	1.9016E-06	0.002051944	H3K4me1	BF
chr16	63433236	63433237	chr16_63433078_63433368	-3.439768	-12.190164	1.9016E-06	0.002051944	H3K4me1	BF
chr16	63433295	63433296	chr16_63433078_63433368	-3.439768	-12.190164	1.9016E-06	0.002051944	H3K4me1	BF
chr16	63433390	63433391	chr16_63433078_63433368	-3.439768	-12.190164	1.9016E-06	0.002051944	H3K4me1	BF
chr16	63433455	63433456	chr16_63433078_63433368	-3.439768	-12.190164	1.9016E-06	0.002051944	H3K4me1	BF
chr16	71903906	71903907	chr16_71903908_71904756	-1.9123342	-10.494677	5.9125E-06	0.005270364	H3K4me1	BF
chr16	71904480	71904481	chr16_71903908_71904756	-1.9123342	-10.494677	5.9125E-06	0.005270364	H3K4me1	BF
chr16	71904667	71904668	chr16_71903908_71904756	-1.9123342	-10.494677	5.9125E-06	0.005270364	H3K4me1	BF

chr16	71904759	71904760	chr16_71903908_71904756	-1.9123342	-10.494677	5.9125E-06	0.005270364	H3K4me1	BF
chr16	20284402	20284403	chr16_20283506_20284587	-1.2392536	-10.246363	7.0758E-06	0.005425078	H3K4me1	BF
chr16	20284470	20284471	chr16_20283506_20284587	-1.2392536	-10.246363	7.0758E-06	0.005425078	H3K4me1	BF
chr16	67256997	67256998	chr16_67256778_67257597	-2.2383021	-10.183554	7.4091E-06	0.005425078	H3K4me1	BF
chr16	67257336	67257337	chr16_67256778_67257597	-2.2383021	-10.183554	7.4091E-06	0.005425078	H3K4me1	BF
chr16	67257585	67257586	chr16_67256778_67257597	-2.2383021	-10.183554	7.4091E-06	0.005425078	H3K4me1	BF
chr17	58995097	58995098	chr17_58995099_58996309	-3.222618	-17.118776	1.3782E-07	0.003019403	H3K4me1	BF
chr17	880010	880011	chr17_880019_880394	-3.5187098	-14.573833	4.8173E-07	0.003941354	H3K4me1	BF
chr17	41313650	41313651	chr17_41313580_41314634	1.8392683	12.7527493	1.3467E-06	0.003941354	H3K4me1	BF
chr17	32803790	32803791	chr17_32803887_32804469	-4.3799967	-11.664616	2.66E-06	0.003941354	H3K4me1	BF
chr17	32803939	32803940	chr17_32803887_32804469	-4.3799967	-11.664616	2.66E-06	0.003941354	H3K4me1	BF
chr17	32804004	32804005	chr17_32803887_32804469	-4.3799967	-11.664616	2.66E-06	0.003941354	H3K4me1	BF
chr17	32804049	32804050	chr17_32803887_32804469	-4.3799967	-11.664616	2.66E-06	0.003941354	H3K4me1	BF

chr17	32804123	32804124	chr17_32803887_32804469	-4.3799967	-11.664616	2.66E-06	0.003941354	H3K4me1	BF
chr17	32804146	32804147	chr17_32803887_32804469	-4.3799967	-11.664616	2.66E-06	0.003941354	H3K4me1	BF
chr17	32804168	32804169	chr17_32803887_32804469	-4.3799967	-11.664616	2.66E-06	0.003941354	H3K4me1	BF
chr17	32804435	32804436	chr17_32803887_32804469	-4.3799967	-11.664616	2.66E-06	0.003941354	H3K4me1	BF
chr17	32804495	32804496	chr17_32803887_32804469	-4.3799967	-11.664616	2.66E-06	0.003941354	H3K4me1	BF
chr17	32804515	32804516	chr17_32803887_32804469	-4.3799967	-11.664616	2.66E-06	0.003941354	H3K4me1	BF
chr17	20956001	20956002	chr17_20955435_20957303	-2.6628269	-11.642558	2.6986E-06	0.003941354	H3K4me1	BF
chr17	20956086	20956087	chr17_20955435_20957303	-2.6628269	-11.642558	2.6986E-06	0.003941354	H3K4me1	BF
chr17	5011780	5011781	chr17_5011729_5012578	-3.3061023	-11.287021	3.4144E-06	0.004155712	H3K4me1	BF
chr17	5012366	5012367	chr17_5011729_5012578	-3.3061023	-11.287021	3.4144E-06	0.004155712	H3K4me1	BF
chr17	5012584	5012585	chr17_5011729_5012578	-3.3061023	-11.287021	3.4144E-06	0.004155712	H3K4me1	BF
chr17	17218079	17218080	chr17_17216744_17219367	4.416658	10.4658317	6.036E-06	0.006611846	H3K4me1	BF
chr17	17218173	17218174	chr17_17216744_17219367	4.416658	10.4658317	6.036E-06	0.006611846	H3K4me1	BF

chr18	21936870	21936871	chr18_21936662_21937254	-2.1113003	-12.611188	1.4669E-06	0.003606989	H3K4me1	BF
chr18	21936905	21936906	chr18_21936662_21937254	-2.1113003	-12.611188	1.4669E-06	0.003606989	H3K4me1	BF
chr18	21936911	21936912	chr18_21936662_21937254	-2.1113003	-12.611188	1.4669E-06	0.003606989	H3K4me1	BF
chr18	50487302	50487303	chr18_50486492_50487931	-10.875746	-11.223881	3.5626E-06	0.003606989	H3K4me1	BF
chr18	50487303	50487304	chr18_50486492_50487931	-10.875746	-11.223881	3.5626E-06	0.003606989	H3K4me1	BF
chr18	50487346	50487347	chr18_50486492_50487931	-10.875746	-11.223881	3.5626E-06	0.003606989	H3K4me1	BF
chr18	50487383	50487384	chr18_50486492_50487931	-10.875746	-11.223881	3.5626E-06	0.003606989	H3K4me1	BF
chr18	50487396	50487397	chr18_50486492_50487931	-10.875746	-11.223881	3.5626E-06	0.003606989	H3K4me1	BF
chr18	50487515	50487516	chr18_50486492_50487931	-10.875746	-11.223881	3.5626E-06	0.003606989	H3K4me1	BF
chr18	50487534	50487535	chr18_50486492_50487931	-10.875746	-11.223881	3.5626E-06	0.003606989	H3K4me1	BF
chr18	50487587	50487588	chr18_50486492_50487931	-10.875746	-11.223881	3.5626E-06	0.003606989	H3K4me1	BF
chr18	50487628	50487629	chr18_50486492_50487931	-10.875746	-11.223881	3.5626E-06	0.003606989	H3K4me1	BF
chr18	50487635	50487636	chr18_50486492_50487931	-10.875746	-11.223881	3.5626E-06	0.003606989	H3K4me1	BF

chr18	50487698	50487699	chr18_50486492_50487931	-10.875746	-11.223881	3.5626E-06	0.003606989	H3K4me1	BF
chr18	50487721	50487722	chr18_50486492_50487931	-10.875746	-11.223881	3.5626E-06	0.003606989	H3K4me1	BF
chr18	50487752	50487753	chr18_50486492_50487931	-10.875746	-11.223881	3.5626E-06	0.003606989	H3K4me1	BF
chr18	50487766	50487767	chr18_50486492_50487931	-10.875746	-11.223881	3.5626E-06	0.003606989	H3K4me1	BF
chr18	645045	645046	chr18_644646_645650	-2.6553584	-10.645328	5.3117E-06	0.004353577	H3K4me1	BF
chr18	645198	645199	chr18_644646_645650	-2.6553584	-10.645328	5.3117E-06	0.004353577	H3K4me1	BF
chr18	645266	645267	chr18_644646_645650	-2.6553584	-10.645328	5.3117E-06	0.004353577	H3K4me1	BF
chr18	645583	645584	chr18_644646_645650	-2.6553584	-10.645328	5.3117E-06	0.004353577	H3K4me1	BF
chr1	142412685	142412686	chr1_142412536_142414460	-3.1450863	-17.720286	1.0522E-07	0.002243182	H3K4me1	BF
chr1	142414313	142414314	chr1_142412536_142414460	-3.1450863	-17.720286	1.0522E-07	0.002243182	H3K4me1	BF
NW_018085246.1	138205	138206	NW_018085246.1_137104_138121	-4.3671018	-17.607087	1.1063E-07	0.002243182	H3K4me1	BF
chr1	268002541	268002542	chr1_268001165_268002512	-2.7839372	-12.666925	1.4182E-06	0.006939437	H3K4me1	BF
chr1	12999283	12999284	chr1_12999060_12999765	1.4821936	12.5880952	1.4877E-06	0.006939437	H3K4me1	BF

chr1	12999309	12999310	chr1_12999060_12999765	1.4821936	12.5880952	1.4877E-06	0.006939437	H3K4me1	BF
chr1	12999349	12999350	chr1_12999060_12999765	1.4821936	12.5880952	1.4877E-06	0.006939437	H3K4me1	BF
chr1	12999356	12999357	chr1_12999060_12999765	1.4821936	12.5880952	1.4877E-06	0.006939437	H3K4me1	BF
chr1	12999396	12999397	chr1_12999060_12999765	1.4821936	12.5880952	1.4877E-06	0.006939437	H3K4me1	BF
chr1	12999708	12999709	chr1_12999060_12999765	1.4821936	12.5880952	1.4877E-06	0.006939437	H3K4me1	BF
chr1	7422232	7422233	chr1_7419908_7422363	3.711714	11.898795	2.2867E-06	0.006939437	H3K4me1	BF
chr1	98200201	98200202	chr1_98199823_98200558	-3.629054	-11.748952	2.5183E-06	0.006939437	H3K4me1	BF
chr1	98200277	98200278	chr1_98199823_98200558	-3.629054	-11.748952	2.5183E-06	0.006939437	H3K4me1	BF
chr1	98200429	98200430	chr1_98199823_98200558	-3.629054	-11.748952	2.5183E-06	0.006939437	H3K4me1	BF
chr1	8809411	8809412	chr1_8809496_8810462	-5.924945	-11.234767	3.5365E-06	0.006939437	H3K4me1	BF
chr1	8809431	8809432	chr1_8809496_8810462	-5.924945	-11.234767	3.5365E-06	0.006939437	H3K4me1	BF
chr1	8809481	8809482	chr1_8809496_8810462	-5.924945	-11.234767	3.5365E-06	0.006939437	H3K4me1	BF
chr1	8809578	8809579	chr1_8809496_8810462	-5.924945	-11.234767	3.5365E-06	0.006939437	H3K4me1	BF

chr1	8809610	8809611	chr1_8809496_8810462	-5.924945	-11.234767	3.5365E-06	0.006939437	H3K4me1	BF
chr1	8809616	8809617	chr1_8809496_8810462	-5.924945	-11.234767	3.5365E-06	0.006939437	H3K4me1	BF
chr1	8809760	8809761	chr1_8809496_8810462	-5.924945	-11.234767	3.5365E-06	0.006939437	H3K4me1	BF
chr1	8809823	8809824	chr1_8809496_8810462	-5.924945	-11.234767	3.5365E-06	0.006939437	H3K4me1	BF
chr1	8809899	8809900	chr1_8809496_8810462	-5.924945	-11.234767	3.5365E-06	0.006939437	H3K4me1	BF
chr1	8809990	8809991	chr1_8809496_8810462	-5.924945	-11.234767	3.5365E-06	0.006939437	H3K4me1	BF
chr1	8810176	8810177	chr1_8809496_8810462	-5.924945	-11.234767	3.5365E-06	0.006939437	H3K4me1	BF
chr1	8810202	8810203	chr1_8809496_8810462	-5.924945	-11.234767	3.5365E-06	0.006939437	H3K4me1	BF
chr1	8810236	8810237	chr1_8809496_8810462	-5.924945	-11.234767	3.5365E-06	0.006939437	H3K4me1	BF
chr1	8810460	8810461	chr1_8809496_8810462	-5.924945	-11.234767	3.5365E-06	0.006939437	H3K4me1	BF
chr1	8810463	8810464	chr1_8809496_8810462	-5.924945	-11.234767	3.5365E-06	0.006939437	H3K4me1	BF
chr1	8810517	8810518	chr1_8809496_8810462	-5.924945	-11.234767	3.5365E-06	0.006939437	H3K4me1	BF
chr1	8810553	8810554	chr1_8809496_8810462	-5.924945	-11.234767	3.5365E-06	0.006939437	H3K4me1	BF

chr1	271399158	271399159	chr1_271398811_271399223	-5.7818038	-10.884856	4.4918E-06	0.008036294	H3K4me1	BF
chr1	271399199	271399200	chr1_271398811_271399223	-5.7818038	-10.884856	4.4918E-06	0.008036294	H3K4me1	BF
chr1	271399287	271399288	chr1_271398811_271399223	-5.7818038	-10.884856	4.4918E-06	0.008036294	H3K4me1	BF
chr1	142408652	142408653	chr1_142408663_142409648	-3.0419792	-10.532035	5.7567E-06	0.008601274	H3K4me1	BF
chr1	159654733	159654734	chr1_159654558_159655388	-1.6724668	-10.455661	6.0802E-06	0.008601274	H3K4me1	BF
chr1	159654826	159654827	chr1_159654558_159655388	-1.6724668	-10.455661	6.0802E-06	0.008601274	H3K4me1	BF
chr1	159654838	159654839	chr1_159654558_159655388	-1.6724668	-10.455661	6.0802E-06	0.008601274	H3K4me1	BF
chr1	159654984	159654985	chr1_159654558_159655388	-1.6724668	-10.455661	6.0802E-06	0.008601274	H3K4me1	BF
chr1	159655231	159655232	chr1_159654558_159655388	-1.6724668	-10.455661	6.0802E-06	0.008601274	H3K4me1	BF
chr1	159655234	159655235	chr1_159654558_159655388	-1.6724668	-10.455661	6.0802E-06	0.008601274	H3K4me1	BF
chr1	159655255	159655256	chr1_159654558_159655388	-1.6724668	-10.455661	6.0802E-06	0.008601274	H3K4me1	BF
chr1	159655415	159655416	chr1_159654558_159655388	-1.6724668	-10.455661	6.0802E-06	0.008601274	H3K4me1	BF
chr2	24645894	24645895	chr2_24645869_24646862	5.3827477	12.3534789	1.7179E-06	0.007345721	H3K4me1	BF

chr2	24646027	24646028	chr2_24645869_24646862	5.3827477	12.3534789	1.7179E-06	0.007345721	H3K4me1	BF
chr2	24646041	24646042	chr2_24645869_24646862	5.3827477	12.3534789	1.7179E-06	0.007345721	H3K4me1	BF
chr2	24646299	24646300	chr2_24645869_24646862	5.3827477	12.3534789	1.7179E-06	0.007345721	H3K4me1	BF
chr2	24646357	24646358	chr2_24645869_24646862	5.3827477	12.3534789	1.7179E-06	0.007345721	H3K4me1	BF
chr2	24646545	24646546	chr2_24645869_24646862	5.3827477	12.3534789	1.7179E-06	0.007345721	H3K4me1	BF
chr2	24646594	24646595	chr2_24645869_24646862	5.3827477	12.3534789	1.7179E-06	0.007345721	H3K4me1	BF
chr2	138401794	138401795	chr2_138401792_138402951	-2.7346372	-11.44663	3.0697E-06	0.007345721	H3K4me1	BF
chr2	138401854	138401855	chr2_138401792_138402951	-2.7346372	-11.44663	3.0697E-06	0.007345721	H3K4me1	BF
chr2	138402122	138402123	chr2_138401792_138402951	-2.7346372	-11.44663	3.0697E-06	0.007345721	H3K4me1	BF
chr2	138402153	138402154	chr2_138401792_138402951	-2.7346372	-11.44663	3.0697E-06	0.007345721	H3K4me1	BF
chr2	138402195	138402196	chr2_138401792_138402951	-2.7346372	-11.44663	3.0697E-06	0.007345721	H3K4me1	BF
chr2	138402311	138402312	chr2_138401792_138402951	-2.7346372	-11.44663	3.0697E-06	0.007345721	H3K4me1	BF
chr2	138402335	138402336	chr2_138401792_138402951	-2.7346372	-11.44663	3.0697E-06	0.007345721	H3K4me1	BF

chr2	138402612	138402613	chr2_138401792_138402951	-2.7346372	-11.44663	3.0697E-06	0.007345721	H3K4me1	BF
chr2	138402839	138402840	chr2_138401792_138402951	-2.7346372	-11.44663	3.0697E-06	0.007345721	H3K4me1	BF
chr2	138402960	138402961	chr2_138401792_138402951	-2.7346372	-11.44663	3.0697E-06	0.007345721	H3K4me1	BF
chr2	138402996	138402997	chr2_138401792_138402951	-2.7346372	-11.44663	3.0697E-06	0.007345721	H3K4me1	BF
chr2	24645077	24645078	chr2_24644945_24645237	4.9001317	10.9027189	4.4366E-06	0.007962605	H3K4me1	BF
chr2	24645098	24645099	chr2_24644945_24645237	4.9001317	10.9027189	4.4366E-06	0.007962605	H3K4me1	BF
chr2	24645119	24645120	chr2_24644945_24645237	4.9001317	10.9027189	4.4366E-06	0.007962605	H3K4me1	BF
chr2	24645142	24645143	chr2_24644945_24645237	4.9001317	10.9027189	4.4366E-06	0.007962605	H3K4me1	BF
chr2	24645225	24645226	chr2_24644945_24645237	4.9001317	10.9027189	4.4366E-06	0.007962605	H3K4me1	BF
chr2	24645287	24645288	chr2_24644945_24645237	4.9001317	10.9027189	4.4366E-06	0.007962605	H3K4me1	BF
chr2	8451520	8451521	chr2_8451617_8452602	-2.144544	-10.650758	5.2914E-06	0.008766164	H3K4me1	BF
chr2	8452300	8452301	chr2_8451617_8452602	-2.144544	-10.650758	5.2914E-06	0.008766164	H3K4me1	BF
chr2	138970317	138970318	chr2_138970286_138970730	-2.2372518	-10.377069	6.4345E-06	0.009119538	H3K4me1	BF

chr2	138970328	138970329	chr2_138970286_138970730	-2.2372518	-10.377069	6.4345E-06	0.009119538	H3K4me1	BF
chr2	138970814	138970815	chr2_138970286_138970730	-2.2372518	-10.377069	6.4345E-06	0.009119538	H3K4me1	BF
chr2	102423001	102423002	chr2_102422730_102423512	-3.4783177	-10.222884	7.1984E-06	0.009119538	H3K4me1	BF
chr2	102423002	102423003	chr2_102422730_102423512	-3.4783177	-10.222884	7.1984E-06	0.009119538	H3K4me1	BF
chr2	102423078	102423079	chr2_102422730_102423512	-3.4783177	-10.222884	7.1984E-06	0.009119538	H3K4me1	BF
chr2	102423079	102423080	chr2_102422730_102423512	-3.4783177	-10.222884	7.1984E-06	0.009119538	H3K4me1	BF
chr2	102423089	102423090	chr2_102422730_102423512	-3.4783177	-10.222884	7.1984E-06	0.009119538	H3K4me1	BF
chr2	69319020	69319021	chr2_69318819_69322697	-0.93294	-9.9028029	9.1299E-06	0.011235977	H3K4me1	BF
chr3	48556043	48556044	chr3_48554916_48557327	-2.41683	-32.080428	9.7093E-10	3.34851E-06	H3K4me1	BF
chr3	48556227	48556228	chr3_48554916_48557327	-2.41683	-32.080428	9.7093E-10	3.34851E-06	H3K4me1	BF
chr3	48556267	48556268	chr3_48554916_48557327	-2.41683	-32.080428	9.7093E-10	3.34851E-06	H3K4me1	BF
chr3	48556442	48556443	chr3_48554916_48557327	-2.41683	-32.080428	9.7093E-10	3.34851E-06	H3K4me1	BF
chr3	48556597	48556598	chr3_48554916_48557327	-2.41683	-32.080428	9.7093E-10	3.34851E-06	H3K4me1	BF

chr3	48556898	48556899	chr3_48554916_48557327	-2.41683	-32.080428	9.7093E-10	3.34851E-06	H3K4me1	BF
chr3	48556961	48556962	chr3_48554916_48557327	-2.41683	-32.080428	9.7093E-10	3.34851E-06	H3K4me1	BF
chr3	48556986	48556987	chr3_48554916_48557327	-2.41683	-32.080428	9.7093E-10	3.34851E-06	H3K4me1	BF
chr3	48556992	48556993	chr3_48554916_48557327	-2.41683	-32.080428	9.7093E-10	3.34851E-06	H3K4me1	BF
chr3	48557257	48557258	chr3_48554916_48557327	-2.41683	-32.080428	9.7093E-10	3.34851E-06	H3K4me1	BF
chr3	48557392	48557393	chr3_48554916_48557327	-2.41683	-32.080428	9.7093E-10	3.34851E-06	H3K4me1	BF
chr3	48557410	48557411	chr3_48554916_48557327	-2.41683	-32.080428	9.7093E-10	3.34851E-06	H3K4me1	BF
chr3	94258046	94258047	chr3_94257485_94258538	-5.84951	-22.167116	1.8126E-08	5.77021E-05	H3K4me1	BF
chr3	118225846	118225847	chr3_118225739_118227025	-2.8909894	-17.992062	9.341E-08	0.000168078	H3K4me1	BF
chr3	118225861	118225862	chr3_118225739_118227025	-2.8909894	-17.992062	9.341E-08	0.000168078	H3K4me1	BF
chr3	118225875	118225876	chr3_118225739_118227025	-2.8909894	-17.992062	9.341E-08	0.000168078	H3K4me1	BF
chr3	118226255	118226256	chr3_118225739_118227025	-2.8909894	-17.992062	9.341E-08	0.000168078	H3K4me1	BF
chr3	118226259	118226260	chr3_118225739_118227025	-2.8909894	-17.992062	9.341E-08	0.000168078	H3K4me1	BF

chr3	118226293	118226294	chr3_118225739_118227025	-2.8909894	-17.992062	9.341E-08	0.000168078	H3K4me1	BF
chr3	118226300	118226301	chr3_118225739_118227025	-2.8909894	-17.992062	9.341E-08	0.000168078	H3K4me1	BF
chr3	118226406	118226407	chr3_118225739_118227025	-2.8909894	-17.992062	9.341E-08	0.000168078	H3K4me1	BF
chr3	118226624	118226625	chr3_118225739_118227025	-2.8909894	-17.992062	9.341E-08	0.000168078	H3K4me1	BF
chr3	118226713	118226714	chr3_118225739_118227025	-2.8909894	-17.992062	9.341E-08	0.000168078	H3K4me1	BF
chr3	45821055	45821056	chr3_45820906_45821786	-4.2630894	-16.954787	1.4858E-07	0.000256204	H3K4me1	BF
chr3	47379731	47379732	chr3_47379391_47380466	-1.300775	-15.202951	3.472E-07	0.000504893	H3K4me1	BF
chr3	47379784	47379785	chr3_47379391_47380466	-1.300775	-15.202951	3.472E-07	0.000504893	H3K4me1	BF
chr3	47379915	47379916	chr3_47379391_47380466	-1.300775	-15.202951	3.472E-07	0.000504893	H3K4me1	BF
chr3	3791236	3791237	chr3_3791091_3792883	-1.9351967	-14.858126	4.148E-07	0.000504893	H3K4me1	BF
chr3	3791527	3791528	chr3_3791091_3792883	-1.9351967	-14.858126	4.148E-07	0.000504893	H3K4me1	BF
chr3	3791950	3791951	chr3_3791091_3792883	-1.9351967	-14.858126	4.148E-07	0.000504893	H3K4me1	BF
chr3	3792105	3792106	chr3_3791091_3792883	-1.9351967	-14.858126	4.148E-07	0.000504893	H3K4me1	BF

chr3	3792236	3792237	chr3_3791091_3792883	-1.9351967	-14.858126	4.148E-07	0.000504893	H3K4me1	BF
chr3	3792297	3792298	chr3_3791091_3792883	-1.9351967	-14.858126	4.148E-07	0.000504893	H3K4me1	BF
chr3	3792781	3792782	chr3_3791091_3792883	-1.9351967	-14.858126	4.148E-07	0.000504893	H3K4me1	BF
chr3	94305093	94305094	chr3_94304061_94306371	-6.727644	-13.579941	8.3103E-07	0.000918412	H3K4me1	BF
NW_018085111.1	11289	11290	NW_018085111.1_10432_11405	2.579208	13.4209769	9.0987E-07	0.000918412	H3K4me1	BF
NW_018085111.1	11134	11135	NW_018085111.1_10432_11405	2.579208	13.4209769	9.0987E-07	0.000918412	H3K4me1	BF
NW_018085111.1	11005	11006	NW_018085111.1_10432_11405	2.579208	13.4209769	9.0987E-07	0.000918412	H3K4me1	BF
NW_018085111.1	10713	10714	NW_018085111.1_10432_11405	2.579208	13.4209769	9.0987E-07	0.000918412	H3K4me1	BF
NW_018085111.1	10712	10713	NW_018085111.1_10432_11405	2.579208	13.4209769	9.0987E-07	0.000918412	H3K4me1	BF
NW_018085111.1	10364	10365	NW_018085111.1_10432_11405	2.579208	13.4209769	9.0987E-07	0.000918412	H3K4me1	BF
chr3	94254539	94254540	chr3_94254558_94255189	-6.598456	-12.52971	1.5415E-06	0.001449911	H3K4me1	BF
chr3	94254779	94254780	chr3_94254558_94255189	-6.598456	-12.52971	1.5415E-06	0.001449911	H3K4me1	BF
chr3	94256221	94256222	chr3_94254558_94255189	-6.598456	-12.52971	1.5415E-06	0.001449911	H3K4me1	BF

chr3	49395050	49395051	chr3_49394990_49395771	-4.6595389	-12.097647	2.0154E-06	0.001668161	H3K4me1	BF
chr3	49395137	49395138	chr3_49394990_49395771	-4.6595389	-12.097647	2.0154E-06	0.001668161	H3K4me1	BF
chr3	49395233	49395234	chr3_49394990_49395771	-4.6595389	-12.097647	2.0154E-06	0.001668161	H3K4me1	BF
chr3	49395245	49395246	chr3_49394990_49395771	-4.6595389	-12.097647	2.0154E-06	0.001668161	H3K4me1	BF
chr3	49395553	49395554	chr3_49394990_49395771	-4.6595389	-12.097647	2.0154E-06	0.001668161	H3K4me1	BF
chr3	49395673	49395674	chr3_49394990_49395771	-4.6595389	-12.097647	2.0154E-06	0.001668161	H3K4me1	BF
chr3	19341556	19341557	chr3_19341360_19341613	-4.8115119	-11.72344	2.5602E-06	0.002037605	H3K4me1	BF
chr3	19341560	19341561	chr3_19341360_19341613	-4.8115119	-11.72344	2.5602E-06	0.002037605	H3K4me1	BF
chr3	125764879	125764880	chr3_125764489_125765229	2.271963	10.628436	5.3756E-06	0.004128194	H3K4me1	BF
chr3	112150093	112150094	chr3_112150156_112151225	1.6734891	10.5178907	5.8152E-06	0.004128194	H3K4me1	BF
chr3	112150379	112150380	chr3_112150156_112151225	1.6734891	10.5178907	5.8152E-06	0.004128194	H3K4me1	BF
chr3	112150407	112150408	chr3_112150156_112151225	1.6734891	10.5178907	5.8152E-06	0.004128194	H3K4me1	BF
chr3	112150599	112150600	chr3_112150156_112151225	1.6734891	10.5178907	5.8152E-06	0.004128194	H3K4me1	BF

chr3	11898407	11898408	chr3_11898250_11898784	0.9687114	10.3665064	6.4838E-06	0.004128194	H3K4me1	BF
chr3	11898483	11898484	chr3_11898250_11898784	0.9687114	10.3665064	6.4838E-06	0.004128194	H3K4me1	BF
chr3	11898527	11898528	chr3_11898250_11898784	0.9687114	10.3665064	6.4838E-06	0.004128194	H3K4me1	BF
chr3	11898643	11898644	chr3_11898250_11898784	0.9687114	10.3665064	6.4838E-06	0.004128194	H3K4me1	BF
chr3	11898699	11898700	chr3_11898250_11898784	0.9687114	10.3665064	6.4838E-06	0.004128194	H3K4me1	BF
chr3	11898719	11898720	chr3_11898250_11898784	0.9687114	10.3665064	6.4838E-06	0.004128194	H3K4me1	BF
chr3	11898816	11898817	chr3_11898250_11898784	0.9687114	10.3665064	6.4838E-06	0.004128194	H3K4me1	BF
chr3	11898866	11898867	chr3_11898250_11898784	0.9687114	10.3665064	6.4838E-06	0.004128194	H3K4me1	BF
chr3	104979494	104979495	chr3_104979238_104980383	-2.5095256	-10.202095	7.3089E-06	0.004583044	H3K4me1	BF
chr3	89742656	89742657	chr3_89742382_89743496	-2.0972852	-10.02348	8.3408E-06	0.005076252	H3K4me1	BF
chr3	89742807	89742808	chr3_89742382_89743496	-2.0972852	-10.02348	8.3408E-06	0.005076252	H3K4me1	BF
chr3	124668674	124668675	chr3_124668580_124669682	-1.9149918	-9.8394145	9.5774E-06	0.005505029	H3K4me1	BF
chr3	124669145	124669146	chr3_124668580_124669682	-1.9149918	-9.8394145	9.5774E-06	0.005505029	H3K4me1	BF

chr3	124669162	124669163	chr3_124668580_124669682	-1.9149918	-9.8394145	9.5774E-06	0.005505029	H3K4me1	BF
chr3	124669472	124669473	chr3_124668580_124669682	-1.9149918	-9.8394145	9.5774E-06	0.005505029	H3K4me1	BF
chr4	127417137	127417138	chr4_127416669_127418009	-3.5769388	-13.302929	9.7384E-07	0.002932693	H3K4me1	BF
chr4	127417297	127417298	chr4_127416669_127418009	-3.5769388	-13.302929	9.7384E-07	0.002932693	H3K4me1	BF
chr4	127417625	127417626	chr4_127416669_127418009	-3.5769388	-13.302929	9.7384E-07	0.002932693	H3K4me1	BF
chr4	127417800	127417801	chr4_127416669_127418009	-3.5769388	-13.302929	9.7384E-07	0.002932693	H3K4me1	BF
chr4	127417933	127417934	chr4_127416669_127418009	-3.5769388	-13.302929	9.7384E-07	0.002932693	H3K4me1	BF
chr4	80894627	80894628	chr4_80894682_80895703	1.6016756	12.5294773	1.5417E-06	0.002932693	H3K4me1	BF
chr4	80894719	80894720	chr4_80894682_80895703	1.6016756	12.5294773	1.5417E-06	0.002932693	H3K4me1	BF
chr4	80895140	80895141	chr4_80894682_80895703	1.6016756	12.5294773	1.5417E-06	0.002932693	H3K4me1	BF
chr4	80895227	80895228	chr4_80894682_80895703	1.6016756	12.5294773	1.5417E-06	0.002932693	H3K4me1	BF
chr4	80895228	80895229	chr4_80894682_80895703	1.6016756	12.5294773	1.5417E-06	0.002932693	H3K4me1	BF
chr4	80895313	80895314	chr4_80894682_80895703	1.6016756	12.5294773	1.5417E-06	0.002932693	H3K4me1	BF

chr4	80895326	80895327	chr4_80894682_80895703	1.6016756	12.5294773	1.5417E-06	0.002932693	H3K4me1	BF
chr4	80895421	80895422	chr4_80894682_80895703	1.6016756	12.5294773	1.5417E-06	0.002932693	H3K4me1	BF
chr4	80895537	80895538	chr4_80894682_80895703	1.6016756	12.5294773	1.5417E-06	0.002932693	H3K4me1	BF
chr4	80895671	80895672	chr4_80894682_80895703	1.6016756	12.5294773	1.5417E-06	0.002932693	H3K4me1	BF
chr4	90699979	90699980	chr4_90699947_90701996	-1.9019711	-12.516656	1.5539E-06	0.002932693	H3K4me1	BF
chr4	90700525	90700526	chr4_90699947_90701996	-1.9019711	-12.516656	1.5539E-06	0.002932693	H3K4me1	BF
chr4	90701326	90701327	chr4_90699947_90701996	-1.9019711	-12.516656	1.5539E-06	0.002932693	H3K4me1	BF
chr4	90701467	90701468	chr4_90699947_90701996	-1.9019711	-12.516656	1.5539E-06	0.002932693	H3K4me1	BF
chr4	90701473	90701474	chr4_90699947_90701996	-1.9019711	-12.516656	1.5539E-06	0.002932693	H3K4me1	BF
chr4	72463647	72463648	chr4_72463533_72464705	4.7145827	11.2037128	3.6114E-06	0.004717312	H3K4me1	BF
chr4	72463723	72463724	chr4_72463533_72464705	4.7145827	11.2037128	3.6114E-06	0.004717312	H3K4me1	BF
chr4	72463778	72463779	chr4_72463533_72464705	4.7145827	11.2037128	3.6114E-06	0.004717312	H3K4me1	BF
chr4	72463857	72463858	chr4_72463533_72464705	4.7145827	11.2037128	3.6114E-06	0.004717312	H3K4me1	BF

chr4	72464055	72464056	chr4_72463533_72464705	4.7145827	11.2037128	3.6114E-06	0.004717312	H3K4me1	BF
chr4	72464401	72464402	chr4_72463533_72464705	4.7145827	11.2037128	3.6114E-06	0.004717312	H3K4me1	BF
chr4	105015252	105015253	chr4_105014263_105015946	2.616436	11.1484129	3.7492E-06	0.004717312	H3K4me1	BF
chr4	105015404	105015405	chr4_105014263_105015946	2.616436	11.1484129	3.7492E-06	0.004717312	H3K4me1	BF
chr4	105015623	105015624	chr4_105014263_105015946	2.616436	11.1484129	3.7492E-06	0.004717312	H3K4me1	BF
chr4	105015750	105015751	chr4_105014263_105015946	2.616436	11.1484129	3.7492E-06	0.004717312	H3K4me1	BF
chr4	2643936	2643937	chr4_2643362_2644531	3.385892	10.1755853	7.4527E-06	0.008037598	H3K4me1	BF
chr4	2643940	2643941	chr4_2643362_2644531	3.385892	10.1755853	7.4527E-06	0.008037598	H3K4me1	BF
chr4	2644130	2644131	chr4_2643362_2644531	3.385892	10.1755853	7.4527E-06	0.008037598	H3K4me1	BF
chr4	2644222	2644223	chr4_2643362_2644531	3.385892	10.1755853	7.4527E-06	0.008037598	H3K4me1	BF
chr4	2644563	2644564	chr4_2643362_2644531	3.385892	10.1755853	7.4527E-06	0.008037598	H3K4me1	BF
chr4	30182726	30182727	chr4_30182607_30183657	-1.6860962	-9.9366516	8.9004E-06	0.00847403	H3K4me1	BF
chr4	30183209	30183210	chr4_30182607_30183657	-1.6860962	-9.9366516	8.9004E-06	0.00847403	H3K4me1	BF

chr4	30183213	30183214	chr4_30182607_30183657	-1.6860962	-9.9366516	8.9004E-06	0.00847403	H3K4me1	BF
chr4	110840848	110840849	chr4_110840057_110841190	2.6365667	9.9248308	8.9798E-06	0.00847403	H3K4me1	BF
chr4	110840850	110840851	chr4_110840057_110841190	2.6365667	9.9248308	8.9798E-06	0.00847403	H3K4me1	BF
chr5	2702730	2702731	chr5_2702021_2703985	3.1348402	20.0885066	3.935E-08	0.001144553	H3K4me1	BF
chr5	15979651	15979652	chr5_15979307_15981624	4.2970971	15.596379	2.8472E-07	0.001144553	H3K4me1	BF
chr5	15979782	15979783	chr5_15979307_15981624	4.2970971	15.596379	2.8472E-07	0.001144553	H3K4me1	BF
chr5	15979794	15979795	chr5_15979307_15981624	4.2970971	15.596379	2.8472E-07	0.001144553	H3K4me1	BF
chr5	15979819	15979820	chr5_15979307_15981624	4.2970971	15.596379	2.8472E-07	0.001144553	H3K4me1	BF
chr5	15979840	15979841	chr5_15979307_15981624	4.2970971	15.596379	2.8472E-07	0.001144553	H3K4me1	BF
chr5	15980386	15980387	chr5_15979307_15981624	4.2970971	15.596379	2.8472E-07	0.001144553	H3K4me1	BF
chr5	15980489	15980490	chr5_15979307_15981624	4.2970971	15.596379	2.8472E-07	0.001144553	H3K4me1	BF
chr5	15980775	15980776	chr5_15979307_15981624	4.2970971	15.596379	2.8472E-07	0.001144553	H3K4me1	BF
chr5	23724060	23724061	chr5_23722953_23724505	2.1572332	15.116321	3.6294E-07	0.001193704	H3K4me1	BF

chr5	23724286	23724287	chr5_23722953_23724505	2.1572332	15.116321	3.6294E-07	0.001193704	H3K4me1	BF
chr5	7749731	7749732	chr5_7749300_7750228	-4.3707051	-12.463696	1.6051E-06	0.004839204	H3K4me1	BF
chr5	80014180	80014181	chr5_80013760_80014739	-2.2683258	-10.935762	4.3364E-06	0.011206279	H3K4me1	BF
chr5	80014445	80014446	chr5_80013760_80014739	-2.2683258	-10.935762	4.3364E-06	0.011206279	H3K4me1	BF
chr5	19297220	19297221	chr5_19296751_19297594	-4.5421593	-10.550184	5.6827E-06	0.012093864	H3K4me1	BF
chr5	19297232	19297233	chr5_19296751_19297594	-4.5421593	-10.550184	5.6827E-06	0.012093864	H3K4me1	BF
chr5	19297592	19297593	chr5_19296751_19297594	-4.5421593	-10.550184	5.6827E-06	0.012093864	H3K4me1	BF
chr6	2704588	2704589	chr6_2704262_2704855	3.299344	13.8088958	7.3058E-07	0.007357589	H3K4me1	BF
chr6	157238130	157238131	chr6_157238017_157239191	3.843818	12.6777358	1.409E-06	0.007357589	H3K4me1	BF
chr6	157238184	157238185	chr6_157238017_157239191	3.843818	12.6777358	1.409E-06	0.007357589	H3K4me1	BF
chr6	157238262	157238263	chr6_157238017_157239191	3.843818	12.6777358	1.409E-06	0.007357589	H3K4me1	BF
chr6	157238324	157238325	chr6_157238017_157239191	3.843818	12.6777358	1.409E-06	0.007357589	H3K4me1	BF
chr6	157238464	157238465	chr6_157238017_157239191	3.843818	12.6777358	1.409E-06	0.007357589	H3K4me1	BF

chr6	157238525	157238526	chr6_157238017_157239191	3.843818	12.6777358	1.409E-06	0.007357589	H3K4me1	BF
chr6	157238552	157238553	chr6_157238017_157239191	3.843818	12.6777358	1.409E-06	0.007357589	H3K4me1	BF
chr6	157238585	157238586	chr6_157238017_157239191	3.843818	12.6777358	1.409E-06	0.007357589	H3K4me1	BF
chr6	8355815	8355816	chr6_8355655_8356374	-3.1960204	-12.195574	1.8952E-06	0.008097105	H3K4me1	BF
chr6	8355819	8355820	chr6_8355655_8356374	-3.1960204	-12.195574	1.8952E-06	0.008097105	H3K4me1	BF
chr6	134628960	134628961	chr6_134628614_134629054	-2.7315129	-11.558197	2.8518E-06	0.008700898	H3K4me1	BF
chr6	147349887	147349888	chr6_147349982_147351252	-4.106315	-10.491874	5.9244E-06	0.008700898	H3K4me1	BF
chr6	147349934	147349935	chr6_147349982_147351252	-4.106315	-10.491874	5.9244E-06	0.008700898	H3K4me1	BF
chr6	147349949	147349950	chr6_147349982_147351252	-4.106315	-10.491874	5.9244E-06	0.008700898	H3K4me1	BF
chr6	147349953	147349954	chr6_147349982_147351252	-4.106315	-10.491874	5.9244E-06	0.008700898	H3K4me1	BF
chr6	147350032	147350033	chr6_147349982_147351252	-4.106315	-10.491874	5.9244E-06	0.008700898	H3K4me1	BF
chr6	147350156	147350157	chr6_147349982_147351252	-4.106315	-10.491874	5.9244E-06	0.008700898	H3K4me1	BF
chr6	147350210	147350211	chr6_147349982_147351252	-4.106315	-10.491874	5.9244E-06	0.008700898	H3K4me1	BF

chr6	147350318	147350319	chr6_147349982_147351252	-4.106315	-10.491874	5.9244E-06	0.008700898	H3K4me1	BF
chr6	147350342	147350343	chr6_147349982_147351252	-4.106315	-10.491874	5.9244E-06	0.008700898	H3K4me1	BF
chr6	147350385	147350386	chr6_147349982_147351252	-4.106315	-10.491874	5.9244E-06	0.008700898	H3K4me1	BF
chr6	147350457	147350458	chr6_147349982_147351252	-4.106315	-10.491874	5.9244E-06	0.008700898	H3K4me1	BF
chr6	147350541	147350542	chr6_147349982_147351252	-4.106315	-10.491874	5.9244E-06	0.008700898	H3K4me1	BF
chr6	147350589	147350590	chr6_147349982_147351252	-4.106315	-10.491874	5.9244E-06	0.008700898	H3K4me1	BF
chr6	147350653	147350654	chr6_147349982_147351252	-4.106315	-10.491874	5.9244E-06	0.008700898	H3K4me1	BF
chr6	147350655	147350656	chr6_147349982_147351252	-4.106315	-10.491874	5.9244E-06	0.008700898	H3K4me1	BF
chr6	147350815	147350816	chr6_147349982_147351252	-4.106315	-10.491874	5.9244E-06	0.008700898	H3K4me1	BF
chr6	147350826	147350827	chr6_147349982_147351252	-4.106315	-10.491874	5.9244E-06	0.008700898	H3K4me1	BF
chr6	147350896	147350897	chr6_147349982_147351252	-4.106315	-10.491874	5.9244E-06	0.008700898	H3K4me1	BF
chr6	147351088	147351089	chr6_147349982_147351252	-4.106315	-10.491874	5.9244E-06	0.008700898	H3K4me1	BF
chr6	147351201	147351202	chr6_147349982_147351252	-4.106315	-10.491874	5.9244E-06	0.008700898	H3K4me1	BF

chr7	110387577	110387578	chr7_110385939_110389700	-5.3299767	-20.179466	3.7977E-08	0.000232478	H3K4me1	BF
chr7	110388495	110388496	chr7_110385939_110389700	-5.3299767	-20.179466	3.7977E-08	0.000232478	H3K4me1	BF
chr7	112602499	112602500	chr7_112602481_112603522	2.5973026	20.0474926	3.9987E-08	0.000232478	H3K4me1	BF
chr7	112602590	112602591	chr7_112602481_112603522	2.5973026	20.0474926	3.9987E-08	0.000232478	H3K4me1	BF
chr7	112602663	112602664	chr7_112602481_112603522	2.5973026	20.0474926	3.9987E-08	0.000232478	H3K4me1	BF
chr7	112603140	112603141	chr7_112602481_112603522	2.5973026	20.0474926	3.9987E-08	0.000232478	H3K4me1	BF
chr7	112603438	112603439	chr7_112602481_112603522	2.5973026	20.0474926	3.9987E-08	0.000232478	H3K4me1	BF
chr7	8168859	8168860	chr7_8168742_8169102	-6.0746691	-14.573944	4.817E-07	0.00163365	H3K4me1	BF
chr7	8168870	8168871	chr7_8168742_8169102	-6.0746691	-14.573944	4.817E-07	0.00163365	H3K4me1	BF
chr7	8168871	8168872	chr7_8168742_8169102	-6.0746691	-14.573944	4.817E-07	0.00163365	H3K4me1	BF
chr7	8168917	8168918	chr7_8168742_8169102	-6.0746691	-14.573944	4.817E-07	0.00163365	H3K4me1	BF
chr7	8169017	8169018	chr7_8168742_8169102	-6.0746691	-14.573944	4.817E-07	0.00163365	H3K4me1	BF
chr7	41864104	41864105	chr7_41864114_41865009	-2.9130894	-10.752104	4.9272E-06	0.006684075	H3K4me1	BF

chr7	41864114	41864115	chr7_41864114_41865009	-2.9130894	-10.752104	4.9272E-06	0.006684075	H3K4me1	BF
chr7	41864521	41864522	chr7_41864114_41865009	-2.9130894	-10.752104	4.9272E-06	0.006684075	H3K4me1	BF
chr7	41864587	41864588	chr7_41864114_41865009	-2.9130894	-10.752104	4.9272E-06	0.006684075	H3K4me1	BF
chr7	41864628	41864629	chr7_41864114_41865009	-2.9130894	-10.752104	4.9272E-06	0.006684075	H3K4me1	BF
chr7	41864648	41864649	chr7_41864114_41865009	-2.9130894	-10.752104	4.9272E-06	0.006684075	H3K4me1	BF
chr7	41864771	41864772	chr7_41864114_41865009	-2.9130894	-10.752104	4.9272E-06	0.006684075	H3K4me1	BF
chr7	41864839	41864840	chr7_41864114_41865009	-2.9130894	-10.752104	4.9272E-06	0.006684075	H3K4me1	BF
chr7	41864883	41864884	chr7_41864114_41865009	-2.9130894	-10.752104	4.9272E-06	0.006684075	H3K4me1	BF
chr7	41864946	41864947	chr7_41864114_41865009	-2.9130894	-10.752104	4.9272E-06	0.006684075	H3K4me1	BF
chr7	41864959	41864960	chr7_41864114_41865009	-2.9130894	-10.752104	4.9272E-06	0.006684075	H3K4me1	BF
chr7	41864974	41864975	chr7_41864114_41865009	-2.9130894	-10.752104	4.9272E-06	0.006684075	H3K4me1	BF
chr7	41864988	41864989	chr7_41864114_41865009	-2.9130894	-10.752104	4.9272E-06	0.006684075	H3K4me1	BF
chr7	41864994	41864995	chr7_41864114_41865009	-2.9130894	-10.752104	4.9272E-06	0.006684075	H3K4me1	BF

chr7	41864999	41865000	chr7_41864114_41865009	-2.9130894	-10.752104	4.9272E-06	0.006684075	H3K4me1	BF
chr7	41865009	41865010	chr7_41864114_41865009	-2.9130894	-10.752104	4.9272E-06	0.006684075	H3K4me1	BF
chr7	41865035	41865036	chr7_41864114_41865009	-2.9130894	-10.752104	4.9272E-06	0.006684075	H3K4me1	BF
chr7	41865076	41865077	chr7_41864114_41865009	-2.9130894	-10.752104	4.9272E-06	0.006684075	H3K4me1	BF
chr7	90272307	90272308	chr7_90272134_90272654	-2.3366774	-10.114203	7.7977E-06	0.009616473	H3K4me1	BF
chr7	90272330	90272331	chr7_90272134_90272654	-2.3366774	-10.114203	7.7977E-06	0.009616473	H3K4me1	BF
chr7	90272426	90272427	chr7_90272134_90272654	-2.3366774	-10.114203	7.7977E-06	0.009616473	H3K4me1	BF
chr7	91501459	91501460	chr7_91501389_91502073	-2.0560052	-9.7942083	9.9116E-06	0.011204776	H3K4me1	BF
chr7	91501548	91501549	chr7_91501389_91502073	-2.0560052	-9.7942083	9.9116E-06	0.011204776	H3K4me1	BF
chr7	91501592	91501593	chr7_91501389_91502073	-2.0560052	-9.7942083	9.9116E-06	0.011204776	H3K4me1	BF
chr8	130051147	130051148	chr8_130050588_130052311	-2.8090354	-16.179228	2.1403E-07	0.001725787	H3K4me1	BF
chr8	130051424	130051425	chr8_130050588_130052311	-2.8090354	-16.179228	2.1403E-07	0.001725787	H3K4me1	BF
chr8	11208384	11208385	chr8_11208160_11210230	-2.4157455	-15.761824	2.623E-07	0.001725787	H3K4me1	BF

chr8	11208402	11208403	chr8_11208160_11210230	-2.4157455	-15.761824	2.623E-07	0.001725787	H3K4me1	BF
chr8	11208456	11208457	chr8_11208160_11210230	-2.4157455	-15.761824	2.623E-07	0.001725787	H3K4me1	BF
chr8	73775342	73775343	chr8_73774992_73776519	-2.2764589	-14.321466	5.5141E-07	0.0030233	H3K4me1	BF
chr8	108173484	108173485	chr8_108173243_108173767	-1.950556	-11.448839	3.0652E-06	0.004906551	H3K4me1	BF
chr8	79171524	79171525	chr8_79171355_79171724	-2.0903836	-11.305545	3.3723E-06	0.004906551	H3K4me1	BF
chr8	74922463	74922464	chr8_74922533_74924551	1.1786608	10.6373651	5.3417E-06	0.004906551	H3K4me1	BF
chr8	74922514	74922515	chr8_74922533_74924551	1.1786608	10.6373651	5.3417E-06	0.004906551	H3K4me1	BF
chr8	74922550	74922551	chr8_74922533_74924551	1.1786608	10.6373651	5.3417E-06	0.004906551	H3K4me1	BF
chr8	74922604	74922605	chr8_74922533_74924551	1.1786608	10.6373651	5.3417E-06	0.004906551	H3K4me1	BF
chr8	74922807	74922808	chr8_74922533_74924551	1.1786608	10.6373651	5.3417E-06	0.004906551	H3K4me1	BF
chr8	74922855	74922856	chr8_74922533_74924551	1.1786608	10.6373651	5.3417E-06	0.004906551	H3K4me1	BF
chr8	74923061	74923062	chr8_74922533_74924551	1.1786608	10.6373651	5.3417E-06	0.004906551	H3K4me1	BF
chr8	74923431	74923432	chr8_74922533_74924551	1.1786608	10.6373651	5.3417E-06	0.004906551	H3K4me1	BF

chr8	74923450	74923451	chr8_74922533_74924551	1.1786608	10.6373651	5.3417E-06	0.004906551	H3K4me1	BF
chr8	74923666	74923667	chr8_74922533_74924551	1.1786608	10.6373651	5.3417E-06	0.004906551	H3K4me1	BF
chr8	74923802	74923803	chr8_74922533_74924551	1.1786608	10.6373651	5.3417E-06	0.004906551	H3K4me1	BF
chr8	74924217	74924218	chr8_74922533_74924551	1.1786608	10.6373651	5.3417E-06	0.004906551	H3K4me1	BF
chr8	74924320	74924321	chr8_74922533_74924551	1.1786608	10.6373651	5.3417E-06	0.004906551	H3K4me1	BF
chr8	74924567	74924568	chr8_74922533_74924551	1.1786608	10.6373651	5.3417E-06	0.004906551	H3K4me1	BF
chr8	32068833	32068834	chr8_32068698_32069795	-3.239158	-10.553914	5.6677E-06	0.004906551	H3K4me1	BF
chr8	32068865	32068866	chr8_32068698_32069795	-3.239158	-10.553914	5.6677E-06	0.004906551	H3K4me1	BF
chr8	32069128	32069129	chr8_32068698_32069795	-3.239158	-10.553914	5.6677E-06	0.004906551	H3K4me1	BF
chr8	32069145	32069146	chr8_32068698_32069795	-3.239158	-10.553914	5.6677E-06	0.004906551	H3K4me1	BF
chr8	32069166	32069167	chr8_32068698_32069795	-3.239158	-10.553914	5.6677E-06	0.004906551	H3K4me1	BF
chr8	32069199	32069200	chr8_32068698_32069795	-3.239158	-10.553914	5.6677E-06	0.004906551	H3K4me1	BF
chr8	32069208	32069209	chr8_32068698_32069795	-3.239158	-10.553914	5.6677E-06	0.004906551	H3K4me1	BF

chr8	32069246	32069247	chr8_32068698_32069795	-3.239158	-10.553914	5.6677E-06	0.004906551	H3K4me1	BF
chr8	32069252	32069253	chr8_32068698_32069795	-3.239158	-10.553914	5.6677E-06	0.004906551	H3K4me1	BF
chr8	32069271	32069272	chr8_32068698_32069795	-3.239158	-10.553914	5.6677E-06	0.004906551	H3K4me1	BF
chr8	32069322	32069323	chr8_32068698_32069795	-3.239158	-10.553914	5.6677E-06	0.004906551	H3K4me1	BF
chr8	32069344	32069345	chr8_32068698_32069795	-3.239158	-10.553914	5.6677E-06	0.004906551	H3K4me1	BF
chr8	32069389	32069390	chr8_32068698_32069795	-3.239158	-10.553914	5.6677E-06	0.004906551	H3K4me1	BF
chr8	32069789	32069790	chr8_32068698_32069795	-3.239158	-10.553914	5.6677E-06	0.004906551	H3K4me1	BF
chr8	32069866	32069867	chr8_32068698_32069795	-3.239158	-10.553914	5.6677E-06	0.004906551	H3K4me1	BF
chr8	32069895	32069896	chr8_32068698_32069795	-3.239158	-10.553914	5.6677E-06	0.004906551	H3K4me1	BF
chr8	18716589	18716590	chr8_18716219_18717053	-2.8417933	-10.469502	6.0201E-06	0.004951113	H3K4me1	BF
chr8	18716835	18716836	chr8_18716219_18717053	-2.8417933	-10.469502	6.0201E-06	0.004951113	H3K4me1	BF
chr9	43625611	43625612	chr9_43625623_43627459	-3.4423593	-17.382927	1.2228E-07	0.001613338	H3K4me1	BF
chr9	43625632	43625633	chr9_43625623_43627459	-3.4423593	-17.382927	1.2228E-07	0.001613338	H3K4me1	BF

chr9	43627492	43627493	chr9_43625623_43627459	-3.4423593	-17.382927	1.2228E-07	0.001613338	H3K4me1	BF
chr9	115529104	115529105	chr9_115528939_115529496	3.8209495	14.2373064	5.7711E-07	0.002855242	H3K4me1	BF
chr9	115529214	115529215	chr9_115528939_115529496	3.8209495	14.2373064	5.7711E-07	0.002855242	H3K4me1	BF
chr9	115529392	115529393	chr9_115528939_115529496	3.8209495	14.2373064	5.7711E-07	0.002855242	H3K4me1	BF
chr9	115529495	115529496	chr9_115528939_115529496	3.8209495	14.2373064	5.7711E-07	0.002855242	H3K4me1	BF
chr9	115529564	115529565	chr9_115528939_115529496	3.8209495	14.2373064	5.7711E-07	0.002855242	H3K4me1	BF
chr9	71191476	71191477	chr9_71190880_71192807	-2.6434674	-13.348602	9.4851E-07	0.003754203	H3K4me1	BF
chr9	71191584	71191585	chr9_71190880_71192807	-2.6434674	-13.348602	9.4851E-07	0.003754203	H3K4me1	BF
chr9	33397723	33397724	chr9_33395410_33398293	-1.66664	-11.595989	2.782E-06	0.006608778	H3K4me1	BF
chr9	33398133	33398134	chr9_33395410_33398293	-1.66664	-11.595989	2.782E-06	0.006608778	H3K4me1	BF
chr9	33398155	33398156	chr9_33395410_33398293	-1.66664	-11.595989	2.782E-06	0.006608778	H3K4me1	BF
chr9	33398165	33398166	chr9_33395410_33398293	-1.66664	-11.595989	2.782E-06	0.006608778	H3K4me1	BF
chr9	45014673	45014674	chr9_45014673_45016028	3.7403323	11.3970123	3.1725E-06	0.006608778	H3K4me1	BF

chr9	45015209	45015210	chr9_45014673_45016028	3.7403323	11.3970123	3.1725E-06	0.006608778	H3K4me1	BF
chr9	45015579	45015580	chr9_45014673_45016028	3.7403323	11.3970123	3.1725E-06	0.006608778	H3K4me1	BF
chr9	45015988	45015989	chr9_45014673_45016028	3.7403323	11.3970123	3.1725E-06	0.006608778	H3K4me1	BF
chr9	34314812	34314813	chr9_34313353_34315279	3.847124	10.7205742	5.0374E-06	0.009969021	H3K4me1	BF
chr9	10016481	10016482	chr9_10016160_10017199	-3.71755	-10.146093	7.6163E-06	0.01370238	H3K4me1	BF
chr9	10016688	10016689	chr9_10016160_10017199	-3.71755	-10.146093	7.6163E-06	0.01370238	H3K4me1	BF
chr9	57799301	57799302	chr9_57798295_57799321	1.1776355	10.0244454	8.3348E-06	0.014343157	H3K4me1	BF
chr10	65734248	65734249	chr10_65734184_65734655	-0.8984083	-9.8716889	9.3466E-06	0.066304489	H3K4me3	BF
chr11	75640432	75640433	chr11_75640469_75641338	-4.4973366	-13.874745	7.0427E-07	0.000676214	H3K4me3	BF
chr11	75640780	75640781	chr11_75640469_75641338	-4.4973366	-13.874745	7.0427E-07	0.000676214	H3K4me3	BF
chr11	75640994	75640995	chr11_75640469_75641338	-4.4973366	-13.874745	7.0427E-07	0.000676214	H3K4me3	BF
chr11	75641239	75641240	chr11_75640469_75641338	-4.4973366	-13.874745	7.0427E-07	0.000676214	H3K4me3	BF
chr11	75641251	75641252	chr11_75640469_75641338	-4.4973366	-13.874745	7.0427E-07	0.000676214	H3K4me3	BF

chr11	75641261	75641262	chr11_75640469_75641338	-4.4973366	-13.874745	7.0427E-07	0.000676214	H3K4me3	BF
chr11	15951199	15951200	chr11_15950267_15951287	1.503009	10.0166692	8.3833E-06	0.003800031	H3K4me3	BF
chr11	15951275	15951276	chr11_15950267_15951287	1.503009	10.0166692	8.3833E-06	0.003800031	H3K4me3	BF
chr11	352145	352146	chr11_351369_353680	-7.5505505	-9.9863824	8.575E-06	0.003800031	H3K4me3	BF
chr11	352243	352244	chr11_351369_353680	-7.5505505	-9.9863824	8.575E-06	0.003800031	H3K4me3	BF
chr11	352354	352355	chr11_351369_353680	-7.5505505	-9.9863824	8.575E-06	0.003800031	H3K4me3	BF
chr11	352545	352546	chr11_351369_353680	-7.5505505	-9.9863824	8.575E-06	0.003800031	H3K4me3	BF
chr11	352660	352661	chr11_351369_353680	-7.5505505	-9.9863824	8.575E-06	0.003800031	H3K4me3	BF
chr12	59579872	59579873	chr12_59578949_59580392	4.1033167	10.2865898	6.8711E-06	0.010202851	H3K4me3	BF
chr12	59579934	59579935	chr12_59578949_59580392	4.1033167	10.2865898	6.8711E-06	0.010202851	H3K4me3	BF
chr12	59579965	59579966	chr12_59578949_59580392	4.1033167	10.2865898	6.8711E-06	0.010202851	H3K4me3	BF
chr12	59580008	59580009	chr12_59578949_59580392	4.1033167	10.2865898	6.8711E-06	0.010202851	H3K4me3	BF
chr12	59580162	59580163	chr12_59578949_59580392	4.1033167	10.2865898	6.8711E-06	0.010202851	H3K4me3	BF

chr12	59580172	59580173	chr12_59578949_59580392	4.1033167	10.2865898	6.8711E-06	0.010202851	H3K4me3	BF
chr12	59580179	59580180	chr12_59578949_59580392	4.1033167	10.2865898	6.8711E-06	0.010202851	H3K4me3	BF
chr12	59580239	59580240	chr12_59578949_59580392	4.1033167	10.2865898	6.8711E-06	0.010202851	H3K4me3	BF
chr12	59580316	59580317	chr12_59578949_59580392	4.1033167	10.2865898	6.8711E-06	0.010202851	H3K4me3	BF
chr12	20911769	20911770	chr12_20911619_20912319	-6.0289163	-10.099746	7.8816E-06	0.010532907	H3K4me3	BF
chr13	140680793	140680794	chr13_140680037_140681682	-13.16406	-10.264071	6.9849E-06	0.010887093	H3K4me3	BF
chr13	140680841	140680842	chr13_140680037_140681682	-13.16406	-10.264071	6.9849E-06	0.010887093	H3K4me3	BF
chr13	140681207	140681208	chr13_140680037_140681682	-13.16406	-10.264071	6.9849E-06	0.010887093	H3K4me3	BF
chr13	140681300	140681301	chr13_140680037_140681682	-13.16406	-10.264071	6.9849E-06	0.010887093	H3K4me3	BF
chr13	140681503	140681504	chr13_140680037_140681682	-13.16406	-10.264071	6.9849E-06	0.010887093	H3K4me3	BF
chr13	140681569	140681570	chr13_140680037_140681682	-13.16406	-10.264071	6.9849E-06	0.010887093	H3K4me3	BF
chr14	103871416	103871417	chr14_103870399_103872636	-62.36492	-16.775649	1.6142E-07	0.000387925	H3K4me3	BF
chr14	103871456	103871457	chr14_103870399_103872636	-62.36492	-16.775649	1.6142E-07	0.000387925	H3K4me3	BF

chr14	103871460	103871461	chr14_103870399_103872636	-62.36492	-16.775649	1.6142E-07	0.000387925	H3K4me3	BF
chr14	103871526	103871527	chr14_103870399_103872636	-62.36492	-16.775649	1.6142E-07	0.000387925	H3K4me3	BF
chr14	137310600	137310601	chr14_137309371_137310565	-5.89434	-13.812854	7.2897E-07	0.001401519	H3K4me3	BF
chr14	87919048	87919049	chr14_87918856_87919413	-10.101266	-9.9340281	8.918E-06	0.007062566	H3K4me3	BF
chr14	87919094	87919095	chr14_87918856_87919413	-10.101266	-9.9340281	8.918E-06	0.007062566	H3K4me3	BF
chr14	87919137	87919138	chr14_87918856_87919413	-10.101266	-9.9340281	8.918E-06	0.007062566	H3K4me3	BF
chr14	87919253	87919254	chr14_87918856_87919413	-10.101266	-9.9340281	8.918E-06	0.007062566	H3K4me3	BF
chr14	87919377	87919378	chr14_87918856_87919413	-10.101266	-9.9340281	8.918E-06	0.007062566	H3K4me3	BF
chr14	87919387	87919388	chr14_87918856_87919413	-10.101266	-9.9340281	8.918E-06	0.007062566	H3K4me3	BF
chr14	87919471	87919472	chr14_87918856_87919413	-10.101266	-9.9340281	8.918E-06	0.007062566	H3K4me3	BF
chr14	133830605	133830606	chr14_133829344_133830922	-6.8166875	-9.8430711	9.551E-06	0.007062566	H3K4me3	BF
chr15	140348788	140348789	chr15_140348697_140349918	-10.575563	-11.657002	2.6733E-06	0.011412917	H3K4me3	BF
chr15	25301394	25301395	chr15_25301129_25301775	-3.6836642	-11.005944	4.132E-06	0.011412917	H3K4me3	BF

chr15	31865931	31865932	chr15_31865937_31866456	-3.919432	-10.429818	6.1943E-06	0.011412917	H3K4me3	BF
chr15	31866230	31866231	chr15_31865937_31866456	-3.919432	-10.429818	6.1943E-06	0.011412917	H3K4me3	BF
chr16	63086864	63086865	chr16_63086669_63086945	-3.039386	-11.160645	3.7182E-06	0.002785663	H3K4me3	BF
chr16	63086873	63086874	chr16_63086669_63086945	-3.039386	-11.160645	3.7182E-06	0.002785663	H3K4me3	BF
chr16	63086895	63086896	chr16_63086669_63086945	-3.039386	-11.160645	3.7182E-06	0.002785663	H3K4me3	BF
chr16	63086903	63086904	chr16_63086669_63086945	-3.039386	-11.160645	3.7182E-06	0.002785663	H3K4me3	BF
chr16	63087033	63087034	chr16_63086669_63086945	-3.039386	-11.160645	3.7182E-06	0.002785663	H3K4me3	BF
chr18	10919145	10919146	chr18_10919132_10921354	-9.6916006	-14.877561	4.1062E-07	0.001966443	H3K4me3	BF
chr1	142450121	142450122	chr1_142450176_142451699	-18.543234	-23.462982	1.1577E-08	4.59843E-05	H3K4me3	BF
chr1	142451516	142451517	chr1_142450176_142451699	-18.543234	-23.462982	1.1577E-08	4.59843E-05	H3K4me3	BF
chr1	142451778	142451779	chr1_142450176_142451699	-18.543234	-23.462982	1.1577E-08	4.59843E-05	H3K4me3	BF
chr1	7679228	7679229	chr1_7678939_7681347	-11.59625	-20.022506	4.0381E-08	4.59843E-05	H3K4me3	BF
chr1	7679607	7679608	chr1_7678939_7681347	-11.59625	-20.022506	4.0381E-08	4.59843E-05	H3K4me3	BF

chr1	7679629	7679630	chr1_7678939_7681347	-11.59625	-20.022506	4.0381E-08	4.59843E-05	H3K4me3	BF
chr1	7679651	7679652	chr1_7678939_7681347	-11.59625	-20.022506	4.0381E-08	4.59843E-05	H3K4me3	BF
chr1	7679751	7679752	chr1_7678939_7681347	-11.59625	-20.022506	4.0381E-08	4.59843E-05	H3K4me3	BF
chr1	7680015	7680016	chr1_7678939_7681347	-11.59625	-20.022506	4.0381E-08	4.59843E-05	H3K4me3	BF
chr1	7680836	7680837	chr1_7678939_7681347	-11.59625	-20.022506	4.0381E-08	4.59843E-05	H3K4me3	BF
chr1	7680860	7680861	chr1_7678939_7681347	-11.59625	-20.022506	4.0381E-08	4.59843E-05	H3K4me3	BF
chr1	7680981	7680982	chr1_7678939_7681347	-11.59625	-20.022506	4.0381E-08	4.59843E-05	H3K4me3	BF
chr1	7681330	7681331	chr1_7678939_7681347	-11.59625	-20.022506	4.0381E-08	4.59843E-05	H3K4me3	BF
chr1	142412685	142412686	chr1_142412334_142413814	-44.425464	-17.391947	1.2179E-07	0.000128784	H3K4me3	BF
chr1	142500481	142500482	chr1_142499806_142501674	-17.92865	-13.561593	8.3973E-07	0.000605928	H3K4me3	BF
chr1	142500930	142500931	chr1_142499806_142501674	-17.92865	-13.561593	8.3973E-07	0.000605928	H3K4me3	BF
chr1	142501054	142501055	chr1_142499806_142501674	-17.92865	-13.561593	8.3973E-07	0.000605928	H3K4me3	BF
chr1	236946719	236946720	chr1_236946332_236947161	13.481982	13.5205971	8.5953E-07	0.000605928	H3K4me3	BF

chr1	236946870	236946871	chr1_236946332_236947161	13.481982	13.5205971	8.5953E-07	0.000605928	H3K4me3	BF
chr1	236946899	236946900	chr1_236946332_236947161	13.481982	13.5205971	8.5953E-07	0.000605928	H3K4me3	BF
chr1	236946939	236946940	chr1_236946332_236947161	13.481982	13.5205971	8.5953E-07	0.000605928	H3K4me3	BF
chr1	8809760	8809761	chr1_8809746_8810000	-3.2895433	-13.159163	1.0587E-06	0.000626892	H3K4me3	BF
chr1	8809823	8809824	chr1_8809746_8810000	-3.2895433	-13.159163	1.0587E-06	0.000626892	H3K4me3	BF
chr1	8809899	8809900	chr1_8809746_8810000	-3.2895433	-13.159163	1.0587E-06	0.000626892	H3K4me3	BF
chr1	8809990	8809991	chr1_8809746_8810000	-3.2895433	-13.159163	1.0587E-06	0.000626892	H3K4me3	BF
chr3	39750360	39750361	chr3_39750236_39751718	12.5488	12.4728143	1.5961E-06	0.007119324	H3K4me3	BF
chr3	39751373	39751374	chr3_39750236_39751718	12.5488	12.4728143	1.5961E-06	0.007119324	H3K4me3	BF
chr3	17761423	17761424	chr3_17761064_17762514	-14.788275	-11.438424	3.0864E-06	0.007119324	H3K4me3	BF
chr3	17762121	17762122	chr3_17761064_17762514	-14.788275	-11.438424	3.0864E-06	0.007119324	H3K4me3	BF
chr3	17762446	17762447	chr3_17761064_17762514	-14.788275	-11.438424	3.0864E-06	0.007119324	H3K4me3	BF
chr3	17762565	17762566	chr3_17761064_17762514	-14.788275	-11.438424	3.0864E-06	0.007119324	H3K4me3	BF

chr4	104650833	104650834	chr4_104650335_104651024	-7.3841803	-13.179525	1.0462E-06	0.003748015	H3K4me3	BF
chr4	104650838	104650839	chr4_104650335_104651024	-7.3841803	-13.179525	1.0462E-06	0.003748015	H3K4me3	BF
NW_018085290.1	34305	34306	NW_018085290.1_33060_34403	-3.8429797	-10.727186	5.0141E-06	0.003748015	H3K4me3	BF
NW_018085290.1	34273	34274	NW_018085290.1_33060_34403	-3.8429797	-10.727186	5.0141E-06	0.003748015	H3K4me3	BF
NW_018085290.1	34163	34164	NW_018085290.1_33060_34403	-3.8429797	-10.727186	5.0141E-06	0.003748015	H3K4me3	BF
NW_018085290.1	34024	34025	NW_018085290.1_33060_34403	-3.8429797	-10.727186	5.0141E-06	0.003748015	H3K4me3	BF
NW_018085290.1	33997	33998	NW_018085290.1_33060_34403	-3.8429797	-10.727186	5.0141E-06	0.003748015	H3K4me3	BF
NW_018085290.1	33711	33712	NW_018085290.1_33060_34403	-3.8429797	-10.727186	5.0141E-06	0.003748015	H3K4me3	BF
NW_018085290.1	33676	33677	NW_018085290.1_33060_34403	-3.8429797	-10.727186	5.0141E-06	0.003748015	H3K4me3	BF
NW_018085290.1	33659	33660	NW_018085290.1_33060_34403	-3.8429797	-10.727186	5.0141E-06	0.003748015	H3K4me3	BF
NW_018085290.1	33366	33367	NW_018085290.1_33060_34403	-3.8429797	-10.727186	5.0141E-06	0.003748015	H3K4me3	BF
NW_018085290.1	33333	33334	NW_018085290.1_33060_34403	-3.8429797	-10.727186	5.0141E-06	0.003748015	H3K4me3	BF
NW_018085290.1	33322	33323	NW_018085290.1_33060_34403	-3.8429797	-10.727186	5.0141E-06	0.003748015	H3K4me3	BF

NW_018085290.1	33227	33228	NW_018085290.1_33060_34403	-3.8429797	-10.727186	5.0141E-06	0.003748015	H3K4me3	BF
NW_018084968.1	1701770	1701771	NW_018084968.1_1701667_1702012	-2.5056479	-15.704622	2.6982E-07	0.002956704	H3K4me3	BF
chr5	63602041	63602042	chr5_63600532_63601946	-11.647528	-10.294977	6.8293E-06	0.009354408	H3K4me3	BF
chr5	63601115	63601116	chr5_63600532_63601946	-11.647528	-10.294977	6.8293E-06	0.009354408	H3K4me3	BF
chr5	63601655	63601656	chr5_63600532_63601946	-11.647528	-10.294977	6.8293E-06	0.009354408	H3K4me3	BF
chr5	63601683	63601684	chr5_63600532_63601946	-11.647528	-10.294977	6.8293E-06	0.009354408	H3K4me3	BF
chr5	63601968	63601969	chr5_63600532_63601946	-11.647528	-10.294977	6.8293E-06	0.009354408	H3K4me3	BF
chr5	63601993	63601994	chr5_63600532_63601946	-11.647528	-10.294977	6.8293E-06	0.009354408	H3K4me3	BF
chr5	63602043	63602044	chr5_63600532_63601946	-11.647528	-10.294977	6.8293E-06	0.009354408	H3K4me3	BF
chr6	83485389	83485390	chr6_83484620_83486536	-9.9092229	-15.204644	3.469E-07	0.003349889	H3K4me3	BF
chr6	1999937	1999938	chr6_1999935_2001148	-12.63252	-11.709327	2.5838E-06	0.003349889	H3K4me3	BF
chr6	2000097	2000098	chr6_1999935_2001148	-12.63252	-11.709327	2.5838E-06	0.003349889	H3K4me3	BF
chr6	2000143	2000144	chr6_1999935_2001148	-12.63252	-11.709327	2.5838E-06	0.003349889	H3K4me3	BF

chr6	2000251	2000252	chr6_1999935_2001148	-12.63252	-11.709327	2.5838E-06	0.003349889	H3K4me3	BF
chr6	2000256	2000257	chr6_1999935_2001148	-12.63252	-11.709327	2.5838E-06	0.003349889	H3K4me3	BF
chr6	2000300	2000301	chr6_1999935_2001148	-12.63252	-11.709327	2.5838E-06	0.003349889	H3K4me3	BF
chr6	2000341	2000342	chr6_1999935_2001148	-12.63252	-11.709327	2.5838E-06	0.003349889	H3K4me3	BF
chr6	2000343	2000344	chr6_1999935_2001148	-12.63252	-11.709327	2.5838E-06	0.003349889	H3K4me3	BF
chr6	2000502	2000503	chr6_1999935_2001148	-12.63252	-11.709327	2.5838E-06	0.003349889	H3K4me3	BF
chr6	2000720	2000721	chr6_1999935_2001148	-12.63252	-11.709327	2.5838E-06	0.003349889	H3K4me3	BF
chr6	2000964	2000965	chr6_1999935_2001148	-12.63252	-11.709327	2.5838E-06	0.003349889	H3K4me3	BF
chr6	51522284	51522285	chr6_51522207_51524035	-8.4829083	-10.368573	6.4741E-06	0.005595802	H3K4me3	BF
chr6	51522436	51522437	chr6_51522207_51524035	-8.4829083	-10.368573	6.4741E-06	0.005595802	H3K4me3	BF
chr6	51522833	51522834	chr6_51522207_51524035	-8.4829083	-10.368573	6.4741E-06	0.005595802	H3K4me3	BF
chr6	51522835	51522836	chr6_51522207_51524035	-8.4829083	-10.368573	6.4741E-06	0.005595802	H3K4me3	BF
chr6	51523509	51523510	chr6_51522207_51524035	-8.4829083	-10.368573	6.4741E-06	0.005595802	H3K4me3	BF

chr6	51523761	51523762	chr6_51522207_51524035	-8.4829083	-10.368573	6.4741E-06	0.005595802	H3K4me3	BF
chr7	92151214	92151215	chr7_92150449_92151214	-1.6773624	-12.702409	1.3882E-06	0.00143941	H3K4me3	BF
chr7	92151283	92151284	chr7_92150449_92151214	-1.6773624	-12.702409	1.3882E-06	0.00143941	H3K4me3	BF
chr8	130363678	130363679	chr8_130363648_130365422	-16.08578	-15.005027	3.8434E-07	0.00068932	H3K4me3	BF
chr8	130364048	130364049	chr8_130363648_130365422	-16.08578	-15.005027	3.8434E-07	0.00068932	H3K4me3	BF
chr8	130364072	130364073	chr8_130363648_130365422	-16.08578	-15.005027	3.8434E-07	0.00068932	H3K4me3	BF
chr8	130364074	130364075	chr8_130363648_130365422	-16.08578	-15.005027	3.8434E-07	0.00068932	H3K4me3	BF
chr8	135245449	135245450	chr8_135245122_135246006	-9.23503	-10.282675	6.8907E-06	0.008239035	H3K4me3	BF
chr8	135246050	135246051	chr8_135245122_135246006	-9.23503	-10.282675	6.8907E-06	0.008239035	H3K4me3	BF
chr9	135097323	135097324	chr9_135096939_135098043	-12.96307	-18.140209	8.7603E-08	0.000305237	H3K4me3	BF
chr9	135097532	135097533	chr9_135096939_135098043	-12.96307	-18.140209	8.7603E-08	0.000305237	H3K4me3	BF
chr9	135097973	135097974	chr9_135096939_135098043	-12.96307	-18.140209	8.7603E-08	0.000305237	H3K4me3	BF
chr9	9819222	9819223	chr9_9819091_9819515	-2.4837363	-10.843951	4.6212E-06	0.00371582	H3K4me3	BF

chr9	9819327	9819328	chr9_9819091_9819515	-2.4837363	-10.843951	4.6212E-06	0.00371582	H3K4me3	BF
chr9	9819335	9819336	chr9_9819091_9819515	-2.4837363	-10.843951	4.6212E-06	0.00371582	H3K4me3	BF
chr9	9819349	9819350	chr9_9819091_9819515	-2.4837363	-10.843951	4.6212E-06	0.00371582	H3K4me3	BF
chr9	9819359	9819360	chr9_9819091_9819515	-2.4837363	-10.843951	4.6212E-06	0.00371582	H3K4me3	BF
chr9	9819403	9819404	chr9_9819091_9819515	-2.4837363	-10.843951	4.6212E-06	0.00371582	H3K4me3	BF
chr9	9819433	9819434	chr9_9819091_9819515	-2.4837363	-10.843951	4.6212E-06	0.00371582	H3K4me3	BF
chr9	9819449	9819450	chr9_9819091_9819515	-2.4837363	-10.843951	4.6212E-06	0.00371582	H3K4me3	BF
chr9	9819551	9819552	chr9_9819091_9819515	-2.4837363	-10.843951	4.6212E-06	0.00371582	H3K4me3	BF
chr9	9819605	9819606	chr9_9819091_9819515	-2.4837363	-10.843951	4.6212E-06	0.00371582	H3K4me3	BF
chr11	73267210	73267211	chr11_73267210_73267503	-3.5337606	-12.837328	1.2802E-06	0.006281955	CTCF	LD
chr11	73267365	73267366	chr11_73267210_73267503	-3.5337606	-12.837328	1.2802E-06	0.006281955	CTCF	LD
chr11	70637151	70637152	chr11_70637244_70637637	-5.4067133	-11.752555	2.5124E-06	0.008218892	CTCF	LD
chr11	351613	351614	chr11_351266_351568	-4.0135175	-11.30662	3.3698E-06	0.008267874	CTCF	LD

chr11	75512289	75512290	chr11_75511886_75512710	-5.3339443	-9.8396964	9.5754E-06	0.018794573	CTCF	LD
chr12	19713936	19713937	chr12_19713354_19714899	-10.314748	-14.106083	6.199E-07	0.001880019	CTCF	LD
chr12	19713977	19713978	chr12_19713354_19714899	-10.314748	-14.106083	6.199E-07	0.001880019	CTCF	LD
chr12	19714086	19714087	chr12_19713354_19714899	-10.314748	-14.106083	6.199E-07	0.001880019	CTCF	LD
chr12	19714157	19714158	chr12_19713354_19714899	-10.314748	-14.106083	6.199E-07	0.001880019	CTCF	LD
chr12	19714257	19714258	chr12_19713354_19714899	-10.314748	-14.106083	6.199E-07	0.001880019	CTCF	LD
chr14	129660889	129660890	chr14_129660665_129661482	-4.460575	-11.395545	3.1756E-06	0.027889524	CTCF	LD
chr14	129661093	129661094	chr14_129660665_129661482	-4.460575	-11.395545	3.1756E-06	0.027889524	CTCF	LD
chr15	31892598	31892599	chr15_31892329_31892710	-5.748852	-10.347812	6.5722E-06	0.068573973	CTCF	LD
chr15	134582595	134582596	chr15_134582188_134582543	-2.7202989	-9.8833212	9.2649E-06	0.068573973	CTCF	LD
chr17	46622087	46622088	chr17_46621838_46622566	-18.362074	-10.69919	5.1137E-06	0.02477082	CTCF	LD
chr17	46622271	46622272	chr17_46621838_46622566	-18.362074	-10.69919	5.1137E-06	0.02477082	CTCF	LD
chr18	14694718	14694719	chr18_14694778_14696518	-10.200865	-10.960936	4.2618E-06	0.001970615	CTCF	LD

chr18	14694831	14694832	chr18_14694778_14696518	-10.200865	-10.960936	4.2618E-06	0.001970615	CTCF	LD
chr18	14694938	14694939	chr18_14694778_14696518	-10.200865	-10.960936	4.2618E-06	0.001970615	CTCF	LD
chr18	14695080	14695081	chr18_14694778_14696518	-10.200865	-10.960936	4.2618E-06	0.001970615	CTCF	LD
chr18	14695154	14695155	chr18_14694778_14696518	-10.200865	-10.960936	4.2618E-06	0.001970615	CTCF	LD
chr18	14695184	14695185	chr18_14694778_14696518	-10.200865	-10.960936	4.2618E-06	0.001970615	CTCF	LD
chr18	14695204	14695205	chr18_14694778_14696518	-10.200865	-10.960936	4.2618E-06	0.001970615	CTCF	LD
chr18	14695317	14695318	chr18_14694778_14696518	-10.200865	-10.960936	4.2618E-06	0.001970615	CTCF	LD
chr18	14695341	14695342	chr18_14694778_14696518	-10.200865	-10.960936	4.2618E-06	0.001970615	CTCF	LD
chr18	14695350	14695351	chr18_14694778_14696518	-10.200865	-10.960936	4.2618E-06	0.001970615	CTCF	LD
chr18	14695501	14695502	chr18_14694778_14696518	-10.200865	-10.960936	4.2618E-06	0.001970615	CTCF	LD
chr18	14695540	14695541	chr18_14694778_14696518	-10.200865	-10.960936	4.2618E-06	0.001970615	CTCF	LD
chr18	14695573	14695574	chr18_14694778_14696518	-10.200865	-10.960936	4.2618E-06	0.001970615	CTCF	LD
chr18	14695792	14695793	chr18_14694778_14696518	-10.200865	-10.960936	4.2618E-06	0.001970615	CTCF	LD

chr18	14696021	14696022	chr18_14694778_14696518	-10.200865	-10.960936	4.2618E-06	0.001970615	CTCF	LD
chr18	14696091	14696092	chr18_14694778_14696518	-10.200865	-10.960936	4.2618E-06	0.001970615	CTCF	LD
chr18	14696171	14696172	chr18_14694778_14696518	-10.200865	-10.960936	4.2618E-06	0.001970615	CTCF	LD
chr18	14696500	14696501	chr18_14694778_14696518	-10.200865	-10.960936	4.2618E-06	0.001970615	CTCF	LD
chr2	151426087	151426088	chr2_151425262_151426198	-28.203442	-15.06857	3.7195E-07	0.007371012	CTCF	LD
chr2	27258330	27258331	chr2_27257872_27258645	-15.286182	-11.168968	3.6973E-06	0.03206468	CTCF	LD
chr2	149962253	149962254	chr2_149961648_149962160	-17.9697	-10.773451	4.8541E-06	0.03206468	CTCF	LD
chr3	120050004	120050005	chr3_120050028_120051564	-5.4377891	-14.733803	4.4269E-07	0.001130082	CTCF	LD
chr3	120050160	120050161	chr3_120050028_120051564	-5.4377891	-14.733803	4.4269E-07	0.001130082	CTCF	LD
chr3	120050609	120050610	chr3_120050028_120051564	-5.4377891	-14.733803	4.4269E-07	0.001130082	CTCF	LD
chr3	120050715	120050716	chr3_120050028_120051564	-5.4377891	-14.733803	4.4269E-07	0.001130082	CTCF	LD
chr3	120050984	120050985	chr3_120050028_120051564	-5.4377891	-14.733803	4.4269E-07	0.001130082	CTCF	LD
chr3	120050995	120050996	chr3_120050028_120051564	-5.4377891	-14.733803	4.4269E-07	0.001130082	CTCF	LD

chr3	120051115	120051116	chr3_120050028_120051564	-5.4377891	-14.733803	4.4269E-07	0.001130082	CTCF	LD
chr3	120051175	120051176	chr3_120050028_120051564	-5.4377891	-14.733803	4.4269E-07	0.001130082	CTCF	LD
chr3	125496765	125496766	chr3_125496828_125497373	-3.7781725	-13.550795	8.4489E-07	0.001917159	CTCF	LD
chr3	25270779	25270780	chr3_25270874_25271524	4.891858	11.6476586	2.6896E-06	0.003661807	CTCF	LD
chr3	25271020	25271021	chr3_25270874_25271524	4.891858	11.6476586	2.6896E-06	0.003661807	CTCF	LD
chr3	25271071	25271072	chr3_25270874_25271524	4.891858	11.6476586	2.6896E-06	0.003661807	CTCF	LD
chr3	25271511	25271512	chr3_25270874_25271524	4.891858	11.6476586	2.6896E-06	0.003661807	CTCF	LD
chr3	25271595	25271596	chr3_25270874_25271524	4.891858	11.6476586	2.6896E-06	0.003661807	CTCF	LD
chr3	25271611	25271612	chr3_25270874_25271524	4.891858	11.6476586	2.6896E-06	0.003661807	CTCF	LD
chr5	62735447	62735448	chr5_62735538_62735764	-6.918466	-9.9978735	8.5017E-06	0.027037011	CTCF	LD
chr5	62735627	62735628	chr5_62735538_62735764	-6.918466	-9.9978735	8.5017E-06	0.027037011	CTCF	LD
chr5	62735694	62735695	chr5_62735538_62735764	-6.918466	-9.9978735	8.5017E-06	0.027037011	CTCF	LD
chr5	62735757	62735758	chr5_62735538_62735764	-6.918466	-9.9978735	8.5017E-06	0.027037011	CTCF	LD

chr5	62735790	62735791	chr5_62735538_62735764	-6.918466	-9.9978735	8.5017E-06	0.027037011	CTCF	LD
chr8	16772947	16772948	chr8_16772526_16772962	-2.7362633	-10.010824	8.4199E-06	0.116935536	CTCF	LD
chr9	61713174	61713175	chr9_61712981_61713380	-5.9625513	-10.127899	7.7192E-06	0.140582266	CTCF	LD
chr10	61019774	61019775	chr10_61019745_61020615	-6.4064912	-26.222056	4.8061E-09	1.25456E-05	H3K27ac	LD
chr10	61019916	61019917	chr10_61019745_61020615	-6.4064912	-26.222056	4.8061E-09	1.25456E-05	H3K27ac	LD
chr10	61019939	61019940	chr10_61019745_61020615	-6.4064912	-26.222056	4.8061E-09	1.25456E-05	H3K27ac	LD
chr10	61019945	61019946	chr10_61019745_61020615	-6.4064912	-26.222056	4.8061E-09	1.25456E-05	H3K27ac	LD
chr10	61020563	61020564	chr10_61019745_61020615	-6.4064912	-26.222056	4.8061E-09	1.25456E-05	H3K27ac	LD
chr10	61020571	61020572	chr10_61019745_61020615	-6.4064912	-26.222056	4.8061E-09	1.25456E-05	H3K27ac	LD
chr10	57922579	57922580	chr10_57922405_57922781	5.5089317	14.8880669	4.0838E-07	0.000799499	H3K27ac	LD
chr10	57922777	57922778	chr10_57922405_57922781	5.5089317	14.8880669	4.0838E-07	0.000799499	H3K27ac	LD
chr10	28127444	28127445	chr10_28127371_28127599	-2.2323168	-12.518947	1.5517E-06	0.002700293	H3K27ac	LD
chr10	50400875	50400876	chr10_50400922_50401617	-9.5502416	-11.703288	2.5939E-06	0.00406264	H3K27ac	LD

chr11	67948921	67948922	chr11_67947411_67950093	-9.305136	-12.170277	1.9255E-06	0.004685798	H3K27ac	LD
chr11	67947411	67947412	chr11_67947411_67950093	9.305136	12.1702767	1.9255E-06	0.004685798	H3K27ac	LD
chr11	67948319	67948320	chr11_67947411_67950093	9.305136	12.1702767	1.9255E-06	0.004685798	H3K27ac	LD
chr11	67948738	67948739	chr11_67947411_67950093	9.305136	12.1702767	1.9255E-06	0.004685798	H3K27ac	LD
chr11	67949725	67949726	chr11_67947411_67950093	9.305136	12.1702767	1.9255E-06	0.004685798	H3K27ac	LD
chr11	19676689	19676690	chr11_19676705_19678228	-8.0985949	-10.839592	4.6353E-06	0.005213053	H3K27ac	LD
chr11	19676804	19676805	chr11_19676705_19678228	-8.0985949	-10.839592	4.6353E-06	0.005213053	H3K27ac	LD
chr11	19676811	19676812	chr11_19676705_19678228	-8.0985949	-10.839592	4.6353E-06	0.005213053	H3K27ac	LD
chr11	14536191	14536192	chr11_14536222_14536561	-1.6462464	-10.509897	5.8485E-06	0.005213053	H3K27ac	LD
chr11	14536280	14536281	chr11_14536222_14536561	-1.6462464	-10.509897	5.8485E-06	0.005213053	H3K27ac	LD
chr11	14536318	14536319	chr11_14536222_14536561	-1.6462464	-10.509897	5.8485E-06	0.005213053	H3K27ac	LD
chr11	65254793	65254794	chr11_65254837_65255735	-3.94158	-10.289861	6.8548E-06	0.005213053	H3K27ac	LD
chr11	65255038	65255039	chr11_65254837_65255735	-3.94158	-10.289861	6.8548E-06	0.005213053	H3K27ac	LD

chr11	65255088	65255089	chr11_65254837_65255735	-3.94158	-10.289861	6.8548E-06	0.005213053	H3K27ac	LD
chr11	65255485	65255486	chr11_65254837_65255735	-3.94158	-10.289861	6.8548E-06	0.005213053	H3K27ac	LD
chr11	65255487	65255488	chr11_65254837_65255735	-3.94158	-10.289861	6.8548E-06	0.005213053	H3K27ac	LD
chr12	58729307	58729308	chr12_58729207_58730627	10.961343	21.2365964	2.5412E-08	0.000162858	H3K27ac	LD
chr12	58729411	58729412	chr12_58729207_58730627	10.961343	21.2365964	2.5412E-08	0.000162858	H3K27ac	LD
chr12	4460802	4460803	chr12_4460022_4461373	-7.7007884	-19.083219	5.8888E-08	0.000162858	H3K27ac	LD
chr12	4461161	4461162	chr12_4460022_4461373	-7.7007884	-19.083219	5.8888E-08	0.000162858	H3K27ac	LD
chr12	19713936	19713937	chr12_19713381_19714949	-6.798664	-18.393163	7.8599E-08	0.000162858	H3K27ac	LD
chr12	19713977	19713978	chr12_19713381_19714949	-6.798664	-18.393163	7.8599E-08	0.000162858	H3K27ac	LD
chr12	19714086	19714087	chr12_19713381_19714949	-6.798664	-18.393163	7.8599E-08	0.000162858	H3K27ac	LD
chr12	19714157	19714158	chr12_19713381_19714949	-6.798664	-18.393163	7.8599E-08	0.000162858	H3K27ac	LD
chr12	19714257	19714258	chr12_19713381_19714949	-6.798664	-18.393163	7.8599E-08	0.000162858	H3K27ac	LD
chr12	31458653	31458654	chr12_31458504_31459093	-2.252559	-13.438895	9.0058E-07	0.001679394	H3K27ac	LD

chr12	54941922	54941923	chr12_54941883_54942302	1.5137531	13.1846103	1.0431E-06	0.001768265	H3K27ac	LD
chr12	55209879	55209880	chr12_55209441_55210267	-12.025804	-12.397681	1.6716E-06	0.002597662	H3K27ac	LD
chr12	55115328	55115329	chr12_55115398_55116851	-3.8967403	-10.607344	5.4565E-06	0.003634021	H3K27ac	LD
chr12	55115776	55115777	chr12_55115398_55116851	-3.8967403	-10.607344	5.4565E-06	0.003634021	H3K27ac	LD
chr12	55115948	55115949	chr12_55115398_55116851	-3.8967403	-10.607344	5.4565E-06	0.003634021	H3K27ac	LD
chr12	55115987	55115988	chr12_55115398_55116851	-3.8967403	-10.607344	5.4565E-06	0.003634021	H3K27ac	LD
chr12	55116029	55116030	chr12_55115398_55116851	-3.8967403	-10.607344	5.4565E-06	0.003634021	H3K27ac	LD
chr12	55116035	55116036	chr12_55115398_55116851	-3.8967403	-10.607344	5.4565E-06	0.003634021	H3K27ac	LD
chr12	55116079	55116080	chr12_55115398_55116851	-3.8967403	-10.607344	5.4565E-06	0.003634021	H3K27ac	LD
chr12	55116134	55116135	chr12_55115398_55116851	-3.8967403	-10.607344	5.4565E-06	0.003634021	H3K27ac	LD
chr12	55116461	55116462	chr12_55115398_55116851	-3.8967403	-10.607344	5.4565E-06	0.003634021	H3K27ac	LD
chr12	55116479	55116480	chr12_55115398_55116851	-3.8967403	-10.607344	5.4565E-06	0.003634021	H3K27ac	LD
chr12	55116556	55116557	chr12_55115398_55116851	-3.8967403	-10.607344	5.4565E-06	0.003634021	H3K27ac	LD

chr12	55116625	55116626	chr12_55115398_55116851	-3.8967403	-10.607344	5.4565E-06	0.003634021	H3K27ac	LD
chr12	55116857	55116858	chr12_55115398_55116851	-3.8967403	-10.607344	5.4565E-06	0.003634021	H3K27ac	LD
chr12	55116885	55116886	chr12_55115398_55116851	-3.8967403	-10.607344	5.4565E-06	0.003634021	H3K27ac	LD
chr12	55116898	55116899	chr12_55115398_55116851	-3.8967403	-10.607344	5.4565E-06	0.003634021	H3K27ac	LD
chr12	55116916	55116917	chr12_55115398_55116851	-3.8967403	-10.607344	5.4565E-06	0.003634021	H3K27ac	LD
chr13	180892958	180892959	chr13_180891887_180892987	-6.1926586	-25.973231	5.1829E-09	5.55534E-05	H3K27ac	LD
chr13	180893016	180893017	chr13_180891887_180892987	-6.1926586	-25.973231	5.1829E-09	5.55534E-05	H3K27ac	LD
chr13	134113413	134113414	chr13_134112975_134114932	-12.210683	-12.520766	1.55E-06	0.011075576	H3K27ac	LD
chr13	82031255	82031256	chr13_82031036_82032914	-2.9506503	-10.702686	5.1012E-06	0.018225563	H3K27ac	LD
chr13	82031894	82031895	chr13_82031036_82032914	-2.9506503	-10.702686	5.1012E-06	0.018225563	H3K27ac	LD
chr13	82032307	82032308	chr13_82031036_82032914	-2.9506503	-10.702686	5.1012E-06	0.018225563	H3K27ac	LD
chr14	135144981	135144982	chr14_135144837_135145520	-6.8247219	-19.238196	5.5267E-08	0.000621508	H3K27ac	LD
chr14	103871416	103871417	chr14_103870639_103872732	-18.78855	-16.801798	1.5947E-07	0.000621508	H3K27ac	LD

chr14	103871456	103871457	chr14_103870639_103872732	-18.78855	-16.801798	1.5947E-07	0.000621508	H3K27ac	LD
chr14	103871460	103871461	chr14_103870639_103872732	-18.78855	-16.801798	1.5947E-07	0.000621508	H3K27ac	LD
chr14	103871526	103871527	chr14_103870639_103872732	-18.78855	-16.801798	1.5947E-07	0.000621508	H3K27ac	LD
chr14	125176875	125176876	chr14_125176644_125177209	-9.0935274	-15.329218	3.2561E-07	0.000793153	H3K27ac	LD
chr14	125176879	125176880	chr14_125176644_125177209	-9.0935274	-15.329218	3.2561E-07	0.000793153	H3K27ac	LD
chr14	125177175	125177176	chr14_125176644_125177209	-9.0935274	-15.329218	3.2561E-07	0.000793153	H3K27ac	LD
chr14	78612323	78612324	chr14_78612168_78612645	2.1175651	11.904951	2.2778E-06	0.004931846	H3K27ac	LD
chr14	129660535	129660536	chr14_129660619_129661491	-3.3225612	-11.080367	3.9268E-06	0.006376799	H3K27ac	LD
chr14	129660889	129660890	chr14_129660619_129661491	-3.3225612	-11.080367	3.9268E-06	0.006376799	H3K27ac	LD
chr14	129661093	129661094	chr14_129660619_129661491	-3.3225612	-11.080367	3.9268E-06	0.006376799	H3K27ac	LD
chr15	84718456	84718457	chr15_84718527_84719582	-6.0859208	-14.995169	3.8631E-07	0.003455706	H3K27ac	LD
chr15	84718868	84718869	chr15_84718527_84719582	-6.0859208	-14.995169	3.8631E-07	0.003455706	H3K27ac	LD
chr15	84728102	84728103	chr15_84727782_84729284	-6.7632778	-11.41318	3.1386E-06	0.005615203	H3K27ac	LD

chr15	84728448	84728449	chr15_84727782_84729284	-6.7632778	-11.41318	3.1386E-06	0.005615203	H3K27ac	LD
chr15	84728649	84728650	chr15_84727782_84729284	-6.7632778	-11.41318	3.1386E-06	0.005615203	H3K27ac	LD
chr15	84729000	84729001	chr15_84727782_84729284	-6.7632778	-11.41318	3.1386E-06	0.005615203	H3K27ac	LD
chr15	84729090	84729091	chr15_84727782_84729284	-6.7632778	-11.41318	3.1386E-06	0.005615203	H3K27ac	LD
chr15	84729220	84729221	chr15_84727782_84729284	-6.7632778	-11.41318	3.1386E-06	0.005615203	H3K27ac	LD
chr15	84729267	84729268	chr15_84727782_84729284	-6.7632778	-11.41318	3.1386E-06	0.005615203	H3K27ac	LD
chr15	84729379	84729380	chr15_84727782_84729284	-6.7632778	-11.41318	3.1386E-06	0.005615203	H3K27ac	LD
chr16	22397113	22397114	chr16_22396976_22397849	-12.572826	-41.121525	1.3467E-10	1.29732E-06	H3K27ac	LD
chr16	71744842	71744843	chr16_71744709_71746050	-7.037076	-13.22618	1.0181E-06	0.001225955	H3K27ac	LD
chr16	71745097	71745098	chr16_71744709_71746050	-7.037076	-13.22618	1.0181E-06	0.001225955	H3K27ac	LD
chr16	71745132	71745133	chr16_71744709_71746050	-7.037076	-13.22618	1.0181E-06	0.001225955	H3K27ac	LD
chr16	71745174	71745175	chr16_71744709_71746050	-7.037076	-13.22618	1.0181E-06	0.001225955	H3K27ac	LD
chr16	71745944	71745945	chr16_71744709_71746050	-7.037076	-13.22618	1.0181E-06	0.001225955	H3K27ac	LD

chr16	71746024	71746025	chr16_71744709_71746050	-7.037076	-13.22618	1.0181E-06	0.001225955	H3K27ac	LD
chr16	71746135	71746136	chr16_71744709_71746050	-7.037076	-13.22618	1.0181E-06	0.001225955	H3K27ac	LD
chr16	71746896	71746897	chr16_71746684_71747249	-8.2136846	-12.433639	1.635E-06	0.001312493	H3K27ac	LD
chr16	71747129	71747130	chr16_71746684_71747249	-8.2136846	-12.433639	1.635E-06	0.001312493	H3K27ac	LD
chr16	71747262	71747263	chr16_71746684_71747249	-8.2136846	-12.433639	1.635E-06	0.001312493	H3K27ac	LD
chr16	71747263	71747264	chr16_71746684_71747249	-8.2136846	-12.433639	1.635E-06	0.001312493	H3K27ac	LD
chr17	46622087	46622088	chr17_46621853_46622524	-32.156822	-9.8987076	9.1581E-06	0.049522161	H3K27ac	LD
chr17	46622271	46622272	chr17_46621853_46622524	-32.156822	-9.8987076	9.1581E-06	0.049522161	H3K27ac	LD
chr1	142412685	142412686	chr1_142412359_142413520	-5.2371446	-15.280706	3.3372E-07	0.001554408	H3K27ac	LD
chr1	141310203	141310204	chr1_141310104_141311061	-3.0636742	-14.715594	4.4695E-07	0.001554408	H3K27ac	LD
chr1	141310457	141310458	chr1_141310104_141311061	-3.0636742	-14.715594	4.4695E-07	0.001554408	H3K27ac	LD
chr1	141310486	141310487	chr1_141310104_141311061	-3.0636742	-14.715594	4.4695E-07	0.001554408	H3K27ac	LD
chr1	141310547	141310548	chr1_141310104_141311061	-3.0636742	-14.715594	4.4695E-07	0.001554408	H3K27ac	LD

chr1	141310568	141310569	chr1_141310104_141311061	-3.0636742	-14.715594	4.4695E-07	0.001554408	H3K27ac	LD
chr1	141310638	141310639	chr1_141310104_141311061	-3.0636742	-14.715594	4.4695E-07	0.001554408	H3K27ac	LD
chr1	141310651	141310652	chr1_141310104_141311061	-3.0636742	-14.715594	4.4695E-07	0.001554408	H3K27ac	LD
chr1	141310665	141310666	chr1_141310104_141311061	-3.0636742	-14.715594	4.4695E-07	0.001554408	H3K27ac	LD
chr1	141310741	141310742	chr1_141310104_141311061	-3.0636742	-14.715594	4.4695E-07	0.001554408	H3K27ac	LD
chr1	7471732	7471733	chr1_7471301_7473459	8.850418	13.9514152	6.7496E-07	0.002133969	H3K27ac	LD
chr1	222537919	222537920	chr1_222537201_222539259	-4.7379755	-13.190259	1.0396E-06	0.002582587	H3K27ac	LD
chr1	222538336	222538337	chr1_222537201_222539259	-4.7379755	-13.190259	1.0396E-06	0.002582587	H3K27ac	LD
chr1	222538441	222538442	chr1_222537201_222539259	-4.7379755	-13.190259	1.0396E-06	0.002582587	H3K27ac	LD
chr1	96566299	96566300	chr1_96566339_96567337	4.2015413	11.5644229	2.8402E-06	0.003406073	H3K27ac	LD
chr1	96566303	96566304	chr1_96566339_96567337	4.2015413	11.5644229	2.8402E-06	0.003406073	H3K27ac	LD
chr1	96566394	96566395	chr1_96566339_96567337	4.2015413	11.5644229	2.8402E-06	0.003406073	H3K27ac	LD
chr1	96566481	96566482	chr1_96566339_96567337	4.2015413	11.5644229	2.8402E-06	0.003406073	H3K27ac	LD

chr1	96566593	96566594	chr1_96566339_96567337	4.2015413	11.5644229	2.8402E-06	0.003406073	H3K27ac	LD
chr1	96566603	96566604	chr1_96566339_96567337	4.2015413	11.5644229	2.8402E-06	0.003406073	H3K27ac	LD
chr1	96566609	96566610	chr1_96566339_96567337	4.2015413	11.5644229	2.8402E-06	0.003406073	H3K27ac	LD
chr1	96566660	96566661	chr1_96566339_96567337	4.2015413	11.5644229	2.8402E-06	0.003406073	H3K27ac	LD
chr1	96566783	96566784	chr1_96566339_96567337	4.2015413	11.5644229	2.8402E-06	0.003406073	H3K27ac	LD
chr1	96567002	96567003	chr1_96566339_96567337	4.2015413	11.5644229	2.8402E-06	0.003406073	H3K27ac	LD
chr1	96567006	96567007	chr1_96566339_96567337	4.2015413	11.5644229	2.8402E-06	0.003406073	H3K27ac	LD
chr1	96567030	96567031	chr1_96566339_96567337	4.2015413	11.5644229	2.8402E-06	0.003406073	H3K27ac	LD
chr1	96567051	96567052	chr1_96566339_96567337	4.2015413	11.5644229	2.8402E-06	0.003406073	H3K27ac	LD
chr1	96567096	96567097	chr1_96566339_96567337	4.2015413	11.5644229	2.8402E-06	0.003406073	H3K27ac	LD
chr1	96567373	96567374	chr1_96566339_96567337	4.2015413	11.5644229	2.8402E-06	0.003406073	H3K27ac	LD
chr1	33936470	33936471	chr1_33936492_33937684	-3.4291693	-10.384958	6.3979E-06	0.004973933	H3K27ac	LD
chr1	33936746	33936747	chr1_33936492_33937684	-3.4291693	-10.384958	6.3979E-06	0.004973933	H3K27ac	LD

chr1	33936945	33936946	chr1_33936492_33937684	-3.4291693	-10.384958	6.3979E-06	0.004973933	H3K27ac	LD
chr1	33937067	33937068	chr1_33936492_33937684	-3.4291693	-10.384958	6.3979E-06	0.004973933	H3K27ac	LD
chr1	33937090	33937091	chr1_33936492_33937684	-3.4291693	-10.384958	6.3979E-06	0.004973933	H3K27ac	LD
chr1	33937113	33937114	chr1_33936492_33937684	-3.4291693	-10.384958	6.3979E-06	0.004973933	H3K27ac	LD
chr1	33937271	33937272	chr1_33936492_33937684	-3.4291693	-10.384958	6.3979E-06	0.004973933	H3K27ac	LD
chr1	33937293	33937294	chr1_33936492_33937684	-3.4291693	-10.384958	6.3979E-06	0.004973933	H3K27ac	LD
chr1	33937441	33937442	chr1_33936492_33937684	-3.4291693	-10.384958	6.3979E-06	0.004973933	H3K27ac	LD
chr1	33937559	33937560	chr1_33936492_33937684	-3.4291693	-10.384958	6.3979E-06	0.004973933	H3K27ac	LD
chr1	51150083	51150084	chr1_51150092_51150813	-4.5543818	-10.204887	7.294E-06	0.004973933	H3K27ac	LD
chr1	51150107	51150108	chr1_51150092_51150813	-4.5543818	-10.204887	7.294E-06	0.004973933	H3K27ac	LD
chr1	51150151	51150152	chr1_51150092_51150813	-4.5543818	-10.204887	7.294E-06	0.004973933	H3K27ac	LD
chr1	51150174	51150175	chr1_51150092_51150813	-4.5543818	-10.204887	7.294E-06	0.004973933	H3K27ac	LD
chr1	51150306	51150307	chr1_51150092_51150813	-4.5543818	-10.204887	7.294E-06	0.004973933	H3K27ac	LD

chr1	51150355	51150356	chr1_51150092_51150813	-4.5543818	-10.204887	7.294E-06	0.004973933	H3K27ac	LD
chr1	51150357	51150358	chr1_51150092_51150813	-4.5543818	-10.204887	7.294E-06	0.004973933	H3K27ac	LD
chr1	51150513	51150514	chr1_51150092_51150813	-4.5543818	-10.204887	7.294E-06	0.004973933	H3K27ac	LD
chr1	51150561	51150562	chr1_51150092_51150813	-4.5543818	-10.204887	7.294E-06	0.004973933	H3K27ac	LD
chr1	51150824	51150825	chr1_51150092_51150813	-4.5543818	-10.204887	7.294E-06	0.004973933	H3K27ac	LD
chr1	51150865	51150866	chr1_51150092_51150813	-4.5543818	-10.204887	7.294E-06	0.004973933	H3K27ac	LD
chr1	51150901	51150902	chr1_51150092_51150813	-4.5543818	-10.204887	7.294E-06	0.004973933	H3K27ac	LD
chr1	4962554	4962555	chr1_4962510_4963139	1.5726392	9.98803023	8.5644E-06	0.005390923	H3K27ac	LD
chr1	4962741	4962742	chr1_4962510_4963139	1.5726392	9.98803023	8.5644E-06	0.005390923	H3K27ac	LD
chr1	4963109	4963110	chr1_4962510_4963139	1.5726392	9.98803023	8.5644E-06	0.005390923	H3K27ac	LD
chr1	7411824	7411825	chr1_7411849_7413820	10.615812	9.9232406	8.9906E-06	0.005390923	H3K27ac	LD
chr1	7411964	7411965	chr1_7411849_7413820	10.615812	9.9232406	8.9906E-06	0.005390923	H3K27ac	LD
chr1	7412492	7412493	chr1_7411849_7413820	10.615812	9.9232406	8.9906E-06	0.005390923	H3K27ac	LD

chr1	7412673	7412674	chr1_7411849_7413820	10.615812	9.9232406	8.9906E-06	0.005390923	H3K27ac	LD
chr2	23050091	23050092	chr2_23050138_23050542	-15.789969	-15.942665	2.4003E-07	0.000863857	H3K27ac	LD
chr2	23050376	23050377	chr2_23050138_23050542	-15.789969	-15.942665	2.4003E-07	0.000863857	H3K27ac	LD
chr2	23050630	23050631	chr2_23050138_23050542	-15.789969	-15.942665	2.4003E-07	0.000863857	H3K27ac	LD
chr2	23050631	23050632	chr2_23050138_23050542	-15.789969	-15.942665	2.4003E-07	0.000863857	H3K27ac	LD
chr2	137410837	137410838	chr2_137410525_137411089	-2.5090667	-15.459729	3.0487E-07	0.000863857	H3K27ac	LD
chr2	137410870	137410871	chr2_137410525_137411089	-2.5090667	-15.459729	3.0487E-07	0.000863857	H3K27ac	LD
chr2	137410963	137410964	chr2_137410525_137411089	-2.5090667	-15.459729	3.0487E-07	0.000863857	H3K27ac	LD
chr2	89746559	89746560	chr2_89746653_89747015	-3.3823697	-14.681542	4.5504E-07	0.000863857	H3K27ac	LD
chr2	840241	840242	chr2_840199_841616	-3.2716563	-14.469269	5.0933E-07	0.000863857	H3K27ac	LD
chr2	840621	840622	chr2_840199_841616	-3.2716563	-14.469269	5.0933E-07	0.000863857	H3K27ac	LD
chr2	840628	840629	chr2_840199_841616	-3.2716563	-14.469269	5.0933E-07	0.000863857	H3K27ac	LD
chr2	840781	840782	chr2_840199_841616	-3.2716563	-14.469269	5.0933E-07	0.000863857	H3K27ac	LD

chr2	841205	841206	chr2_840199_841616	-3.2716563	-14.469269	5.0933E-07	0.000863857	H3K27ac	LD
chr2	841531	841532	chr2_840199_841616	-3.2716563	-14.469269	5.0933E-07	0.000863857	H3K27ac	LD
chr2	144738641	144738642	chr2_144738494_144739552	5.3641958	10.7402923	4.9682E-06	0.007373067	H3K27ac	LD
chr2	144738842	144738843	chr2_144738494_144739552	5.3641958	10.7402923	4.9682E-06	0.007373067	H3K27ac	LD
chr2	25523373	25523374	chr2_25523225_25523394	-1.9178541	-10.126952	7.7246E-06	0.010789464	H3K27ac	LD
chr3	112786598	112786599	chr3_112786544_112787693	-7.40466	-14.612781	4.7188E-07	0.000831444	H3K27ac	LD
chr3	112787108	112787109	chr3_112786544_112787693	-7.40466	-14.612781	4.7188E-07	0.000831444	H3K27ac	LD
chr3	112787526	112787527	chr3_112786544_112787693	-7.40466	-14.612781	4.7188E-07	0.000831444	H3K27ac	LD
chr3	120049442	120049443	chr3_120048946_120051483	-5.92102	-14.449505	5.1474E-07	0.000831444	H3K27ac	LD
chr3	120049454	120049455	chr3_120048946_120051483	-5.92102	-14.449505	5.1474E-07	0.000831444	H3K27ac	LD
chr3	120049854	120049855	chr3_120048946_120051483	-5.92102	-14.449505	5.1474E-07	0.000831444	H3K27ac	LD
chr3	120049897	120049898	chr3_120048946_120051483	-5.92102	-14.449505	5.1474E-07	0.000831444	H3K27ac	LD
chr3	120050004	120050005	chr3_120048946_120051483	-5.92102	-14.449505	5.1474E-07	0.000831444	H3K27ac	LD

chr3	120050160	120050161	chr3_120048946_120051483	-5.92102	-14.449505	5.1474E-07	0.000831444	H3K27ac	LD
chr3	120050609	120050610	chr3_120048946_120051483	-5.92102	-14.449505	5.1474E-07	0.000831444	H3K27ac	LD
chr3	120050715	120050716	chr3_120048946_120051483	-5.92102	-14.449505	5.1474E-07	0.000831444	H3K27ac	LD
chr3	120050984	120050985	chr3_120048946_120051483	-5.92102	-14.449505	5.1474E-07	0.000831444	H3K27ac	LD
chr3	120050995	120050996	chr3_120048946_120051483	-5.92102	-14.449505	5.1474E-07	0.000831444	H3K27ac	LD
chr3	120051115	120051116	chr3_120048946_120051483	-5.92102	-14.449505	5.1474E-07	0.000831444	H3K27ac	LD
chr3	120051175	120051176	chr3_120048946_120051483	-5.92102	-14.449505	5.1474E-07	0.000831444	H3K27ac	LD
chr3	57374026	57374027	chr3_57374115_57375076	3.564835	12.1607999	1.9369E-06	0.001805003	H3K27ac	LD
chr3	57374066	57374067	chr3_57374115_57375076	3.564835	12.1607999	1.9369E-06	0.001805003	H3K27ac	LD
chr3	57374176	57374177	chr3_57374115_57375076	3.564835	12.1607999	1.9369E-06	0.001805003	H3K27ac	LD
chr3	57374236	57374237	chr3_57374115_57375076	3.564835	12.1607999	1.9369E-06	0.001805003	H3K27ac	LD
chr3	57374332	57374333	chr3_57374115_57375076	3.564835	12.1607999	1.9369E-06	0.001805003	H3K27ac	LD
chr3	57374569	57374570	chr3_57374115_57375076	3.564835	12.1607999	1.9369E-06	0.001805003	H3K27ac	LD

chr3	57374587	57374588	chr3_57374115_57375076	3.564835	12.1607999	1.9369E-06	0.001805003	H3K27ac	LD
chr3	57374875	57374876	chr3_57374115_57375076	3.564835	12.1607999	1.9369E-06	0.001805003	H3K27ac	LD
chr3	57374917	57374918	chr3_57374115_57375076	3.564835	12.1607999	1.9369E-06	0.001805003	H3K27ac	LD
chr3	57374949	57374950	chr3_57374115_57375076	3.564835	12.1607999	1.9369E-06	0.001805003	H3K27ac	LD
chr4	123635733	123635734	chr4_123635655_123637365	8.0211888	17.9238562	9.6228E-08	0.000672283	H3K27ac	LD
chr4	123635756	123635757	chr4_123635655_123637365	8.0211888	17.9238562	9.6228E-08	0.000672283	H3K27ac	LD
chr4	123635927	123635928	chr4_123635655_123637365	8.0211888	17.9238562	9.6228E-08	0.000672283	H3K27ac	LD
chr4	38548195	38548196	chr4_38547918_38548201	-6.4910887	-14.507005	4.9917E-07	0.002615519	H3K27ac	LD
chr4	115750159	115750160	chr4_115749480_115750289	-6.9735734	-13.396288	9.2285E-07	0.003868393	H3K27ac	LD
chr5	72845358	72845359	chr5_72845252_72845511	-5.156225	-26.070639	5.0316E-09	4.91137E-05	H3K27ac	LD
chr5	72845382	72845383	chr5_72845252_72845511	-5.156225	-26.070639	5.0316E-09	4.91137E-05	H3K27ac	LD
chr5	32158226	32158227	chr5_32158206_32158926	-7.5385764	-18.918355	6.3035E-08	0.000307643	H3K27ac	LD
chr5	32158537	32158538	chr5_32158206_32158926	-7.5385764	-18.918355	6.3035E-08	0.000307643	H3K27ac	LD

chr5	91274972	91274973	chr5_91274322_91275534	4.9707613	17.5824901	1.1185E-07	0.000436693	H3K27ac	LD
chr5	5088227	5088228	chr5_5088311_5089276	5.4885713	15.4931759	2.9979E-07	0.000650286	H3K27ac	LD
chr5	5088246	5088247	chr5_5088311_5089276	5.4885713	15.4931759	2.9979E-07	0.000650286	H3K27ac	LD
chr5	5088402	5088403	chr5_5088311_5089276	5.4885713	15.4931759	2.9979E-07	0.000650286	H3K27ac	LD
chr5	5088843	5088844	chr5_5088311_5089276	5.4885713	15.4931759	2.9979E-07	0.000650286	H3K27ac	LD
chr5	28658574	28658575	chr5_28658110_28658936	-8.7614234	-12.422373	1.6464E-06	0.002921851	H3K27ac	LD
chr5	28658674	28658675	chr5_28658110_28658936	-8.7614234	-12.422373	1.6464E-06	0.002921851	H3K27ac	LD
chr5	102128207	102128208	chr5_102127853_102128369	2.9322977	11.3175455	3.3453E-06	0.005023553	H3K27ac	LD
chr5	102128308	102128309	chr5_102127853_102128369	2.9322977	11.3175455	3.3453E-06	0.005023553	H3K27ac	LD
chr5	91288256	91288257	chr5_91288331_91289584	3.9149949	10.8591249	4.5727E-06	0.00637637	H3K27ac	LD
chr5	13823891	13823892	chr5_13823717_13824765	3.7372857	10.1562454	7.5595E-06	0.007767198	H3K27ac	LD
chr5	13823910	13823911	chr5_13823717_13824765	3.7372857	10.1562454	7.5595E-06	0.007767198	H3K27ac	LD
chr5	13823954	13823955	chr5_13823717_13824765	3.7372857	10.1562454	7.5595E-06	0.007767198	H3K27ac	LD

chr5	13824358	13824359	chr5_13823717_13824765	3.7372857	10.1562454	7.5595E-06	0.007767198	H3K27ac	LD
chr5	13824406	13824407	chr5_13823717_13824765	3.7372857	10.1562454	7.5595E-06	0.007767198	H3K27ac	LD
chr6	5686870	5686871	chr6_5686417_5686910	-8.8584178	-20.248805	3.6966E-08	0.000765396	H3K27ac	LD
chr6	5659992	5659993	chr6_5659876_5660064	-3.155476	-14.673745	4.5692E-07	0.000765396	H3K27ac	LD
chr6	5659789	5659790	chr6_5659876_5660064	-3.155476	-14.673745	4.5692E-07	0.000765396	H3K27ac	LD
chr6	5659801	5659802	chr6_5659876_5660064	-3.155476	-14.673745	4.5692E-07	0.000765396	H3K27ac	LD
chr6	5659815	5659816	chr6_5659876_5660064	-3.155476	-14.673745	4.5692E-07	0.000765396	H3K27ac	LD
chr6	5659959	5659960	chr6_5659876_5660064	-3.155476	-14.673745	4.5692E-07	0.000765396	H3K27ac	LD
chr6	5660030	5660031	chr6_5659876_5660064	-3.155476	-14.673745	4.5692E-07	0.000765396	H3K27ac	LD
chr6	5660040	5660041	chr6_5659876_5660064	-3.155476	-14.673745	4.5692E-07	0.000765396	H3K27ac	LD
chr6	5660047	5660048	chr6_5659876_5660064	-3.155476	-14.673745	4.5692E-07	0.000765396	H3K27ac	LD
chr6	5660060	5660061	chr6_5659876_5660064	-3.155476	-14.673745	4.5692E-07	0.000765396	H3K27ac	LD
chr6	5660077	5660078	chr6_5659876_5660064	-3.155476	-14.673745	4.5692E-07	0.000765396	H3K27ac	LD

chr6	5660086	5660087	chr6_5659876_5660064	-3.155476	-14.673745	4.5692E-07	0.000765396	H3K27ac	LD
chr6	5660123	5660124	chr6_5659876_5660064	-3.155476	-14.673745	4.5692E-07	0.000765396	H3K27ac	LD
chr6	5660128	5660129	chr6_5659876_5660064	-3.155476	-14.673745	4.5692E-07	0.000765396	H3K27ac	LD
chr6	5660146	5660147	chr6_5659876_5660064	-3.155476	-14.673745	4.5692E-07	0.000765396	H3K27ac	LD
chr6	97147711	97147712	chr6_97147549_97149075	-6.1874546	-10.439568	6.151E-06	0.005724268	H3K27ac	LD
chr6	97147721	97147722	chr6_97147549_97149075	-6.1874546	-10.439568	6.151E-06	0.005724268	H3K27ac	LD
chr6	97147825	97147826	chr6_97147549_97149075	-6.1874546	-10.439568	6.151E-06	0.005724268	H3K27ac	LD
chr6	97147832	97147833	chr6_97147549_97149075	-6.1874546	-10.439568	6.151E-06	0.005724268	H3K27ac	LD
chr6	97148170	97148171	chr6_97147549_97149075	-6.1874546	-10.439568	6.151E-06	0.005724268	H3K27ac	LD
chr6	97148179	97148180	chr6_97147549_97149075	-6.1874546	-10.439568	6.151E-06	0.005724268	H3K27ac	LD
chr6	97148230	97148231	chr6_97147549_97149075	-6.1874546	-10.439568	6.151E-06	0.005724268	H3K27ac	LD
chr6	97148391	97148392	chr6_97147549_97149075	-6.1874546	-10.439568	6.151E-06	0.005724268	H3K27ac	LD
chr6	97148418	97148419	chr6_97147549_97149075	-6.1874546	-10.439568	6.151E-06	0.005724268	H3K27ac	LD

chr6	97148468	97148469	chr6_97147549_97149075	-6.1874546	-10.439568	6.151E-06	0.005724268	H3K27ac	LD
chr6	97148968	97148969	chr6_97147549_97149075	-6.1874546	-10.439568	6.151E-06	0.005724268	H3K27ac	LD
chr6	97149020	97149021	chr6_97147549_97149075	-6.1874546	-10.439568	6.151E-06	0.005724268	H3K27ac	LD
chr6	4935760	4935761	chr6_4935753_4936375	-2.4082751	-9.8580524	9.4433E-06	0.006839751	H3K27ac	LD
chr6	4935904	4935905	chr6_4935753_4936375	-2.4082751	-9.8580524	9.4433E-06	0.006839751	H3K27ac	LD
chr6	4935977	4935978	chr6_4935753_4936375	-2.4082751	-9.8580524	9.4433E-06	0.006839751	H3K27ac	LD
chr6	4936297	4936298	chr6_4935753_4936375	-2.4082751	-9.8580524	9.4433E-06	0.006839751	H3K27ac	LD
chr6	4936402	4936403	chr6_4935753_4936375	-2.4082751	-9.8580524	9.4433E-06	0.006839751	H3K27ac	LD
chr6	4936469	4936470	chr6_4935753_4936375	-2.4082751	-9.8580524	9.4433E-06	0.006839751	H3K27ac	LD
chr6	64424021	64424022	chr6_64423668_64424438	2.7992224	9.84635498	9.5273E-06	0.006839751	H3K27ac	LD
chr6	64424049	64424050	chr6_64423668_64424438	2.7992224	9.84635498	9.5273E-06	0.006839751	H3K27ac	LD
chr7	9866557	9866558	chr7_9866559_9867297	-6.5713267	-29.558186	1.8601E-09	1.09464E-05	H3K27ac	LD
chr7	9866602	9866603	chr7_9866559_9867297	-6.5713267	-29.558186	1.8601E-09	1.09464E-05	H3K27ac	LD

chr7	9866920	9866921	chr7_9866559_9867297	-6.5713267	-29.558186	1.8601E-09	1.09464E-05	H3K27ac	LD
chr7	9866937	9866938	chr7_9866559_9867297	-6.5713267	-29.558186	1.8601E-09	1.09464E-05	H3K27ac	LD
chr7	35330276	35330277	chr7_35329493_35330649	6.1695193	14.6443773	4.6406E-07	0.001780531	H3K27ac	LD
chr7	35330279	35330280	chr7_35329493_35330649	6.1695193	14.6443773	4.6406E-07	0.001780531	H3K27ac	LD
chr7	58712573	58712574	chr7_58712349_58713389	4.356997	14.1501764	6.0513E-07	0.001780531	H3K27ac	LD
chr7	58712918	58712919	chr7_58712349_58713389	4.356997	14.1501764	6.0513E-07	0.001780531	H3K27ac	LD
chr7	58584829	58584830	chr7_58584834_58585748	2.5214266	12.8009418	1.3084E-06	0.002346935	H3K27ac	LD
chr7	58584991	58584992	chr7_58584834_58585748	2.5214266	12.8009418	1.3084E-06	0.002346935	H3K27ac	LD
chr7	58585070	58585071	chr7_58584834_58585748	2.5214266	12.8009418	1.3084E-06	0.002346935	H3K27ac	LD
chr7	58585829	58585830	chr7_58584834_58585748	2.5214266	12.8009418	1.3084E-06	0.002346935	H3K27ac	LD
chr7	51156075	51156076	chr7_51156073_51156853	-4.0063157	-12.196257	1.8944E-06	0.002346935	H3K27ac	LD
chr7	51156189	51156190	chr7_51156073_51156853	-4.0063157	-12.196257	1.8944E-06	0.002346935	H3K27ac	LD
chr7	51156308	51156309	chr7_51156073_51156853	-4.0063157	-12.196257	1.8944E-06	0.002346935	H3K27ac	LD

chr7	51156404	51156405	chr7_51156073_51156853	-4.0063157	-12.196257	1.8944E-06	0.002346935	H3K27ac	LD
chr7	51156405	51156406	chr7_51156073_51156853	-4.0063157	-12.196257	1.8944E-06	0.002346935	H3K27ac	LD
chr7	51156477	51156478	chr7_51156073_51156853	-4.0063157	-12.196257	1.8944E-06	0.002346935	H3K27ac	LD
chr7	51156478	51156479	chr7_51156073_51156853	-4.0063157	-12.196257	1.8944E-06	0.002346935	H3K27ac	LD
chr7	88483057	88483058	chr7_88483146_88483743	-5.8272008	-10.830001	4.6663E-06	0.004224615	H3K27ac	LD
chr7	88483084	88483085	chr7_88483146_88483743	-5.8272008	-10.830001	4.6663E-06	0.004224615	H3K27ac	LD
chr7	88483099	88483100	chr7_88483146_88483743	-5.8272008	-10.830001	4.6663E-06	0.004224615	H3K27ac	LD
chr7	88483308	88483309	chr7_88483146_88483743	-5.8272008	-10.830001	4.6663E-06	0.004224615	H3K27ac	LD
chr7	88483321	88483322	chr7_88483146_88483743	-5.8272008	-10.830001	4.6663E-06	0.004224615	H3K27ac	LD
chr7	88483336	88483337	chr7_88483146_88483743	-5.8272008	-10.830001	4.6663E-06	0.004224615	H3K27ac	LD
chr7	88483349	88483350	chr7_88483146_88483743	-5.8272008	-10.830001	4.6663E-06	0.004224615	H3K27ac	LD
chr7	120896410	120896411	chr7_120896456_120897213	-3.5077145	-9.788545	9.9544E-06	0.008678358	H3K27ac	LD
chr8	23827862	23827863	chr8_23827512_23828826	2.3673838	21.21351	2.5631E-08	0.000148582	H3K27ac	LD

chr8	23828415	23828416	chr8_23827512_23828826	2.3673838	21.21351	2.5631E-08	0.000148582	H3K27ac	LD
chr8	23828441	23828442	chr8_23827512_23828826	2.3673838	21.21351	2.5631E-08	0.000148582	H3K27ac	LD
chr8	15615337	15615338	chr8_15615311_15615914	-3.2887196	-16.223967	2.0948E-07	0.000364304	H3K27ac	LD
chr8	15615638	15615639	chr8_15615311_15615914	-3.2887196	-16.223967	2.0948E-07	0.000364304	H3K27ac	LD
chr8	15615653	15615654	chr8_15615311_15615914	-3.2887196	-16.223967	2.0948E-07	0.000364304	H3K27ac	LD
chr8	15615715	15615716	chr8_15615311_15615914	-3.2887196	-16.223967	2.0948E-07	0.000364304	H3K27ac	LD
chr8	15615760	15615761	chr8_15615311_15615914	-3.2887196	-16.223967	2.0948E-07	0.000364304	H3K27ac	LD
chr8	15615932	15615933	chr8_15615311_15615914	-3.2887196	-16.223967	2.0948E-07	0.000364304	H3K27ac	LD
chr8	15615985	15615986	chr8_15615311_15615914	-3.2887196	-16.223967	2.0948E-07	0.000364304	H3K27ac	LD
chr8	2685747	2685748	chr8_2684190_2685809	-5.7729033	-11.157437	3.7263E-06	0.005400308	H3K27ac	LD
chr8	2685894	2685895	chr8_2684190_2685809	-5.7729033	-11.157437	3.7263E-06	0.005400308	H3K27ac	LD
chr8	109781167	109781168	chr8_109779641_109781515	1.8301457	9.78551507	9.9773E-06	0.013347362	H3K27ac	LD
chr9	137171172	137171173	chr9_137171211_137172433	-5.9971738	-10.755131	4.9168E-06	0.017390586	H3K27ac	LD

chr9	137171606	137171607	chr9_137171211_137172433	-5.9971738	-10.755131	4.9168E-06	0.017390586	H3K27ac	LD
chr9	137171614	137171615	chr9_137171211_137172433	-5.9971738	-10.755131	4.9168E-06	0.017390586	H3K27ac	LD
chr9	137171705	137171706	chr9_137171211_137172433	-5.9971738	-10.755131	4.9168E-06	0.017390586	H3K27ac	LD
chr9	137171873	137171874	chr9_137171211_137172433	-5.9971738	-10.755131	4.9168E-06	0.017390586	H3K27ac	LD
chr9	137172088	137172089	chr9_137171211_137172433	-5.9971738	-10.755131	4.9168E-06	0.017390586	H3K27ac	LD
chr12	25338489	25338490	chr12_25338363_25339563	-13.881078	-13.182589	1.0443E-06	0.010485675	H3K27me3	LD
chr12	25339364	25339365	chr12_25338363_25339563	-13.881078	-13.182589	1.0443E-06	0.010485675	H3K27me3	LD
chr12	11165248	11165249	chr12_11164504_11165342	-5.7447318	-11.390562	3.1861E-06	0.017062684	H3K27me3	LD
chr12	24662154	24662155	chr12_24661753_24662390	-4.5972078	-11.293936	3.3986E-06	0.017062684	H3K27me3	LD
chr12	39468006	39468007	chr12_39467735_39468923	-7.3030709	-10.043764	8.2158E-06	0.023570032	H3K27me3	LD
chr12	39468569	39468570	chr12_39467735_39468923	-7.3030709	-10.043764	8.2158E-06	0.023570032	H3K27me3	LD
chr12	39468712	39468713	chr12_39467735_39468923	-7.3030709	-10.043764	8.2158E-06	0.023570032	H3K27me3	LD
chr13	24266203	24266204	chr13_24266285_24266611	-10.980608	-17.124562	1.3746E-07	0.000455355	H3K27me3	LD

chr13	24266306	24266307	chr13_24266285_24266611	-10.980608	-17.124562	1.3746E-07	0.000455355	H3K27me3	LD
chr13	24266336	24266337	chr13_24266285_24266611	-10.980608	-17.124562	1.3746E-07	0.000455355	H3K27me3	LD
chr13	24266417	24266418	chr13_24266285_24266611	-10.980608	-17.124562	1.3746E-07	0.000455355	H3K27me3	LD
chr13	24266677	24266678	chr13_24266285_24266611	-10.980608	-17.124562	1.3746E-07	0.000455355	H3K27me3	LD
chr13	24266711	24266712	chr13_24266285_24266611	-10.980608	-17.124562	1.3746E-07	0.000455355	H3K27me3	LD
NW_018085356.1	148300	148301	NW_018085356.1_148064_149601	4.801426	12.4535034	1.6152E-06	0.004012866	H3K27me3	LD
NW_018085356.1	148231	148232	NW_018085356.1_148064_149601	4.801426	12.4535034	1.6152E-06	0.004012866	H3K27me3	LD
chr14	74095938	74095939	chr14_74095560_74097098	-2.2894467	-11.006574	4.1302E-06	0.028966903	H3K27me3	LD
chr14	74096224	74096225	chr14_74095560_74097098	-2.2894467	-11.006574	4.1302E-06	0.028966903	H3K27me3	LD
chr14	74096279	74096280	chr14_74095560_74097098	-2.2894467	-11.006574	4.1302E-06	0.028966903	H3K27me3	LD
chr14	74097043	74097044	chr14_74095560_74097098	-2.2894467	-11.006574	4.1302E-06	0.028966903	H3K27me3	LD
chr14	139812956	139812957	chr14_139811434_139813754	-4.9558625	-10.507621	5.858E-06	0.032868181	H3K27me3	LD
chr15	134018992	134018993	chr15_134019078_134019549	-16.852959	-16.617329	1.7381E-07	0.000294254	H3K27me3	LD

chr15	134018993	134018994	chr15_134019078_134019549	-16.852959	-16.617329	1.7381E-07	0.000294254	H3K27me3	LD
chr15	134019002	134019003	chr15_134019078_134019549	-16.852959	-16.617329	1.7381E-07	0.000294254	H3K27me3	LD
chr15	134019148	134019149	chr15_134019078_134019549	-16.852959	-16.617329	1.7381E-07	0.000294254	H3K27me3	LD
chr15	134019247	134019248	chr15_134019078_134019549	-16.852959	-16.617329	1.7381E-07	0.000294254	H3K27me3	LD
chr15	134019260	134019261	chr15_134019078_134019549	-16.852959	-16.617329	1.7381E-07	0.000294254	H3K27me3	LD
chr15	134019298	134019299	chr15_134019078_134019549	-16.852959	-16.617329	1.7381E-07	0.000294254	H3K27me3	LD
chr15	134019304	134019305	chr15_134019078_134019549	-16.852959	-16.617329	1.7381E-07	0.000294254	H3K27me3	LD
chr15	134019326	134019327	chr15_134019078_134019549	-16.852959	-16.617329	1.7381E-07	0.000294254	H3K27me3	LD
chr15	134019553	134019554	chr15_134019078_134019549	-16.852959	-16.617329	1.7381E-07	0.000294254	H3K27me3	LD
chr15	134019558	134019559	chr15_134019078_134019549	-16.852959	-16.617329	1.7381E-07	0.000294254	H3K27me3	LD
chr17	58992470	58992471	chr17_58991629_58993188	13.416398	19.1297874	5.7772E-08	0.001002004	H3K27me3	LD
chr17	50331243	50331244	chr17_50331030_50331320	-14.92978	-13.126757	1.0789E-06	0.00127904	H3K27me3	LD
chr17	63059960	63059961	chr17_63059763_63060095	-8.379032	-12.778047	1.3264E-06	0.00127904	H3K27me3	LD

chr17	50323750	50323751	chr17_50323827_50325964	-6.566848	-12.776814	1.3274E-06	0.00127904	H3K27me3	LD
chr17	50323949	50323950	chr17_50323827_50325964	-6.566848	-12.776814	1.3274E-06	0.00127904	H3K27me3	LD
chr17	50323973	50323974	chr17_50323827_50325964	-6.566848	-12.776814	1.3274E-06	0.00127904	H3K27me3	LD
chr17	50324051	50324052	chr17_50323827_50325964	-6.566848	-12.776814	1.3274E-06	0.00127904	H3K27me3	LD
chr17	50324124	50324125	chr17_50323827_50325964	-6.566848	-12.776814	1.3274E-06	0.00127904	H3K27me3	LD
chr17	50324154	50324155	chr17_50323827_50325964	-6.566848	-12.776814	1.3274E-06	0.00127904	H3K27me3	LD
chr17	50324168	50324169	chr17_50323827_50325964	-6.566848	-12.776814	1.3274E-06	0.00127904	H3K27me3	LD
chr17	50324313	50324314	chr17_50323827_50325964	-6.566848	-12.776814	1.3274E-06	0.00127904	H3K27me3	LD
chr17	50324593	50324594	chr17_50323827_50325964	-6.566848	-12.776814	1.3274E-06	0.00127904	H3K27me3	LD
chr17	50325292	50325293	chr17_50323827_50325964	-6.566848	-12.776814	1.3274E-06	0.00127904	H3K27me3	LD
chr17	50325364	50325365	chr17_50323827_50325964	-6.566848	-12.776814	1.3274E-06	0.00127904	H3K27me3	LD
chr17	50325428	50325429	chr17_50323827_50325964	-6.566848	-12.776814	1.3274E-06	0.00127904	H3K27me3	LD
chr17	50325439	50325440	chr17_50323827_50325964	-6.566848	-12.776814	1.3274E-06	0.00127904	H3K27me3	LD

chr17	50325631	50325632	chr17_50323827_50325964	-6.566848	-12.776814	1.3274E-06	0.00127904	H3K27me3	LD
chr17	50325670	50325671	chr17_50323827_50325964	-6.566848	-12.776814	1.3274E-06	0.00127904	H3K27me3	LD
chr17	33772938	33772939	chr17_33772410_33772947	-10.013367	-9.938563	8.8877E-06	0.008113049	H3K27me3	LD
chr1	16056863	16056864	chr1_16056729_16059254	-18.425168	-15.652902	2.7683E-07	0.000489711	H3K27me3	LD
chr1	16056912	16056913	chr1_16056729_16059254	-18.425168	-15.652902	2.7683E-07	0.000489711	H3K27me3	LD
chr1	16057099	16057100	chr1_16056729_16059254	-18.425168	-15.652902	2.7683E-07	0.000489711	H3K27me3	LD
chr1	16057393	16057394	chr1_16056729_16059254	-18.425168	-15.652902	2.7683E-07	0.000489711	H3K27me3	LD
chr1	16057397	16057398	chr1_16056729_16059254	-18.425168	-15.652902	2.7683E-07	0.000489711	H3K27me3	LD
chr1	16057792	16057793	chr1_16056729_16059254	-18.425168	-15.652902	2.7683E-07	0.000489711	H3K27me3	LD
chr1	16058088	16058089	chr1_16056729_16059254	-18.425168	-15.652902	2.7683E-07	0.000489711	H3K27me3	LD
chr1	16058093	16058094	chr1_16056729_16059254	-18.425168	-15.652902	2.7683E-07	0.000489711	H3K27me3	LD
chr1	16058374	16058375	chr1_16056729_16059254	-18.425168	-15.652902	2.7683E-07	0.000489711	H3K27me3	LD
chr1	16058634	16058635	chr1_16056729_16059254	-18.425168	-15.652902	2.7683E-07	0.000489711	H3K27me3	LD

chr1	16058677	16058678	chr1_16056729_16059254	-18.425168	-15.652902	2.7683E-07	0.000489711	H3K27me3	LD
chr1	16058693	16058694	chr1_16056729_16059254	-18.425168	-15.652902	2.7683E-07	0.000489711	H3K27me3	LD
chr1	16058712	16058713	chr1_16056729_16059254	-18.425168	-15.652902	2.7683E-07	0.000489711	H3K27me3	LD
chr1	16058717	16058718	chr1_16056729_16059254	-18.425168	-15.652902	2.7683E-07	0.000489711	H3K27me3	LD
chr1	16058961	16058962	chr1_16056729_16059254	-18.425168	-15.652902	2.7683E-07	0.000489711	H3K27me3	LD
chr1	16059047	16059048	chr1_16056729_16059254	-18.425168	-15.652902	2.7683E-07	0.000489711	H3K27me3	LD
chr1	16059236	16059237	chr1_16056729_16059254	-18.425168	-15.652902	2.7683E-07	0.000489711	H3K27me3	LD
chr1	132677896	132677897	chr1_132677751_132679461	4.3062757	15.2185715	3.4444E-07	0.000545183	H3K27me3	LD
chr1	132678559	132678560	chr1_132677751_132679461	4.3062757	15.2185715	3.4444E-07	0.000545183	H3K27me3	LD
chr1	142244555	142244556	chr1_142244315_142245218	-5.0590924	-14.985326	3.8828E-07	0.000578422	H3K27me3	LD
chr1	142270730	142270731	chr1_142269881_142270883	-7.3186672	-14.909199	4.0391E-07	0.000578422	H3K27me3	LD
chr1	7501355	7501356	chr1_7501092_7501663	-5.6120976	-11.528506	2.9081E-06	0.003975188	H3K27me3	LD
chr1	255685206	255685207	chr1_255684931_255685243	-4.3893511	-11.01806	4.0978E-06	0.005357898	H3K27me3	LD

chr2	117311624	117311625	chr2_117311555_117311854	4.5905488	10.9460685	4.3057E-06	0.019886352	H3K27me3	LD
chr2	117311746	117311747	chr2_117311555_117311854	4.5905488	10.9460685	4.3057E-06	0.019886352	H3K27me3	LD
chr2	117311791	117311792	chr2_117311555_117311854	4.5905488	10.9460685	4.3057E-06	0.019886352	H3K27me3	LD
chr2	117311814	117311815	chr2_117311555_117311854	4.5905488	10.9460685	4.3057E-06	0.019886352	H3K27me3	LD
chr2	117311949	117311950	chr2_117311555_117311854	4.5905488	10.9460685	4.3057E-06	0.019886352	H3K27me3	LD
chr3	57978	57979	chr3_57841_58609	-11.705343	-23.192782	1.2686E-08	0.000179641	H3K27me3	LD
chr3	58213	58214	chr3_57841_58609	-11.705343	-23.192782	1.2686E-08	0.000179641	H3K27me3	LD
chr3	46431583	46431584	chr3_46431353_46432085	-6.0861976	-12.33689	1.7356E-06	0.005252276	H3K27me3	LD
chr3	46431585	46431586	chr3_46431353_46432085	-6.0861976	-12.33689	1.7356E-06	0.005252276	H3K27me3	LD
chr3	46431712	46431713	chr3_46431353_46432085	-6.0861976	-12.33689	1.7356E-06	0.005252276	H3K27me3	LD
chr3	46431736	46431737	chr3_46431353_46432085	-6.0861976	-12.33689	1.7356E-06	0.005252276	H3K27me3	LD
chr3	46431799	46431800	chr3_46431353_46432085	-6.0861976	-12.33689	1.7356E-06	0.005252276	H3K27me3	LD
chr3	46431840	46431841	chr3_46431353_46432085	-6.0861976	-12.33689	1.7356E-06	0.005252276	H3K27me3	LD

chr3	8306600	8306601	chr3_8306423_8306930	-6.541994	-11.596137	2.7817E-06	0.005252276	H3K27me3	LD
chr3	8306618	8306619	chr3_8306423_8306930	-6.541994	-11.596137	2.7817E-06	0.005252276	H3K27me3	LD
chr3	8306645	8306646	chr3_8306423_8306930	-6.541994	-11.596137	2.7817E-06	0.005252276	H3K27me3	LD
chr3	8306691	8306692	chr3_8306423_8306930	-6.541994	-11.596137	2.7817E-06	0.005252276	H3K27me3	LD
chr3	8306715	8306716	chr3_8306423_8306930	-6.541994	-11.596137	2.7817E-06	0.005252276	H3K27me3	LD
chr3	8306871	8306872	chr3_8306423_8306930	-6.541994	-11.596137	2.7817E-06	0.005252276	H3K27me3	LD
chr3	8307014	8307015	chr3_8306423_8306930	-6.541994	-11.596137	2.7817E-06	0.005252276	H3K27me3	LD
chr3	94287772	94287773	chr3_94287609_94288006	-4.83993	-11.169702	3.6954E-06	0.005814561	H3K27me3	LD
chr3	94288059	94288060	chr3_94287609_94288006	-4.83993	-11.169702	3.6954E-06	0.005814561	H3K27me3	LD
chr3	94288065	94288066	chr3_94287609_94288006	-4.83993	-11.169702	3.6954E-06	0.005814561	H3K27me3	LD
NW_018085169.1	8494	8495	NW_018085169.1_8431_8800	-9.952308	-10.059324	8.1214E-06	0.012105951	H3K27me3	LD
chr4	2175410	2175411	chr4_2175144_2175983	10.270456	25.9415349	5.2333E-09	4.26897E-05	H3K27me3	LD
chr4	2175445	2175446	chr4_2175144_2175983	10.270456	25.9415349	5.2333E-09	4.26897E-05	H3K27me3	LD

chr4	2175520	2175521	chr4_2175144_2175983	10.270456	25.9415349	5.2333E-09	4.26897E-05	H3K27me3	LD
chr4	108842659	108842660	chr4_108842307_108843459	-5.7675474	-13.944323	6.7761E-07	0.002929823	H3K27me3	LD
chr4	108843374	108843375	chr4_108842307_108843459	-5.7675474	-13.944323	6.7761E-07	0.002929823	H3K27me3	LD
chr4	81237576	81237577	chr4_81237051_81237853	-4.94112	-13.214679	1.025E-06	0.003583254	H3K27me3	LD
chr4	89544667	89544668	chr4_89544585_89545100	-4.637708	-11.686546	2.6223E-06	0.004908949	H3K27me3	LD
chr4	89544721	89544722	chr4_89544585_89545100	-4.637708	-11.686546	2.6223E-06	0.004908949	H3K27me3	LD
chr4	89544843	89544844	chr4_89544585_89545100	-4.637708	-11.686546	2.6223E-06	0.004908949	H3K27me3	LD
chr4	7757282	7757283	chr4_7756894_7757588	2.6147976	9.81011001	9.7926E-06	0.014096715	H3K27me3	LD
chr5	17611849	17611850	chr5_17611545_17613253	-9.7068718	-47.999499	3.9256E-11	8.82669E-07	H3K27me3	LD
chr5	94076165	94076166	chr5_94075455_94076956	5.5987727	12.6696089	1.4159E-06	0.010550069	H3K27me3	LD
chr5	94076589	94076590	chr5_94075455_94076956	5.5987727	12.6696089	1.4159E-06	0.010550069	H3K27me3	LD
chr5	17626497	17626498	chr5_17626250_17626607	-17.430548	-12.211146	1.8768E-06	0.010550069	H3K27me3	LD
chr5	79256210	79256211	chr5_79255877_79256883	-2.1876408	-10.319545	6.7084E-06	0.013712501	H3K27me3	LD

chr5	79256371	79256372	chr5_79255877_79256883	-2.1876408	-10.319545	6.7084E-06	0.013712501	H3K27me3	LD
chr5	79256466	79256467	chr5_79255877_79256883	-2.1876408	-10.319545	6.7084E-06	0.013712501	H3K27me3	LD
chr5	79256676	79256677	chr5_79255877_79256883	-2.1876408	-10.319545	6.7084E-06	0.013712501	H3K27me3	LD
chr5	79256694	79256695	chr5_79255877_79256883	-2.1876408	-10.319545	6.7084E-06	0.013712501	H3K27me3	LD
chr5	79256744	79256745	chr5_79255877_79256883	-2.1876408	-10.319545	6.7084E-06	0.013712501	H3K27me3	LD
chr5	79256765	79256766	chr5_79255877_79256883	-2.1876408	-10.319545	6.7084E-06	0.013712501	H3K27me3	LD
chr6	13946064	13946065	chr6_13944392_13946691	-8.9525295	-14.898764	4.0611E-07	0.002333102	H3K27me3	LD
chr6	13946124	13946125	chr6_13944392_13946691	-8.9525295	-14.898764	4.0611E-07	0.002333102	H3K27me3	LD
chr6	13946211	13946212	chr6_13944392_13946691	-8.9525295	-14.898764	4.0611E-07	0.002333102	H3K27me3	LD
chr6	13946243	13946244	chr6_13944392_13946691	-8.9525295	-14.898764	4.0611E-07	0.002333102	H3K27me3	LD
chr6	13946428	13946429	chr6_13944392_13946691	-8.9525295	-14.898764	4.0611E-07	0.002333102	H3K27me3	LD
chr6	13946439	13946440	chr6_13944392_13946691	-8.9525295	-14.898764	4.0611E-07	0.002333102	H3K27me3	LD
chr6	128579918	128579919	chr6_128579101_128580055	-3.1723379	-10.780737	4.8295E-06	0.022227378	H3K27me3	LD

chr6	49110859	49110860	chr6_49110947_49111286	-7.160174	-9.8367161	9.597E-06	0.022227378	H3K27me3	LD
chr6	49110868	49110869	chr6_49110947_49111286	-7.160174	-9.8367161	9.597E-06	0.022227378	H3K27me3	LD
chr6	49110877	49110878	chr6_49110947_49111286	-7.160174	-9.8367161	9.597E-06	0.022227378	H3K27me3	LD
chr6	49110906	49110907	chr6_49110947_49111286	-7.160174	-9.8367161	9.597E-06	0.022227378	H3K27me3	LD
chr6	49110979	49110980	chr6_49110947_49111286	-7.160174	-9.8367161	9.597E-06	0.022227378	H3K27me3	LD
chr6	49110980	49110981	chr6_49110947_49111286	-7.160174	-9.8367161	9.597E-06	0.022227378	H3K27me3	LD
chr6	49111195	49111196	chr6_49110947_49111286	-7.160174	-9.8367161	9.597E-06	0.022227378	H3K27me3	LD
chr6	144577252	144577253	chr6_144575984_144578245	-7.451706	-9.826378	9.6725E-06	0.022227378	H3K27me3	LD
chr7	7506205	7506206	chr7_7506030_7506292	-5.7060478	-16.037842	2.2917E-07	0.002953499	H3K27me3	LD
chr7	7506220	7506221	chr7_7506030_7506292	-5.7060478	-16.037842	2.2917E-07	0.002953499	H3K27me3	LD
NW_018084979.1	3722638	3722639	NW_018084979.1_3722643_3723687	-8.83263	-10.584695	5.5449E-06	0.010149368	H3K27me3	LD
NW_018084979.1	3722669	3722670	NW_018084979.1_3722643_3723687	-8.83263	-10.584695	5.5449E-06	0.010149368	H3K27me3	LD
NW_018084979.1	3722716	3722717	NW_018084979.1_3722643_3723687	-8.83263	-10.584695	5.5449E-06	0.010149368	H3K27me3	LD

chr7	54235149	54235150	chr7_54234989_54236175	-8.066026	-10.334401	6.6364E-06	0.010149368	H3K27me3	LD
chr7	54235496	54235497	chr7_54234989_54236175	-8.066026	-10.334401	6.6364E-06	0.010149368	H3K27me3	LD
chr7	54235803	54235804	chr7_54234989_54236175	-8.066026	-10.334401	6.6364E-06	0.010149368	H3K27me3	LD
chr7	54235861	54235862	chr7_54234989_54236175	-8.066026	-10.334401	6.6364E-06	0.010149368	H3K27me3	LD
chr7	54276383	54276384	chr7_54276248_54276717	-6.6926242	-10.318351	6.7142E-06	0.010149368	H3K27me3	LD
chr7	54276488	54276489	chr7_54276248_54276717	-6.6926242	-10.318351	6.7142E-06	0.010149368	H3K27me3	LD
chr7	54276489	54276490	chr7_54276248_54276717	-6.6926242	-10.318351	6.7142E-06	0.010149368	H3K27me3	LD
chr7	54276499	54276500	chr7_54276248_54276717	-6.6926242	-10.318351	6.7142E-06	0.010149368	H3K27me3	LD
chr7	54276512	54276513	chr7_54276248_54276717	-6.6926242	-10.318351	6.7142E-06	0.010149368	H3K27me3	LD
chr7	54276526	54276527	chr7_54276248_54276717	-6.6926242	-10.318351	6.7142E-06	0.010149368	H3K27me3	LD
chr7	58340343	58340344	chr7_58339370_58340791	-3.657759	-9.8373213	9.5926E-06	0.010149368	H3K27me3	LD
chr7	121718280	121718281	chr7_121718364_121720792	-5.982066	-9.803238	9.8438E-06	0.010149368	H3K27me3	LD
chr7	121718336	121718337	chr7_121718364_121720792	-5.982066	-9.803238	9.8438E-06	0.010149368	H3K27me3	LD

chr7	121718379	121718380	chr7_121718364_121720792	-5.982066	-9.803238	9.8438E-06	0.010149368	H3K27me3	LD
chr7	121718559	121718560	chr7_121718364_121720792	-5.982066	-9.803238	9.8438E-06	0.010149368	H3K27me3	LD
chr7	121718853	121718854	chr7_121718364_121720792	-5.982066	-9.803238	9.8438E-06	0.010149368	H3K27me3	LD
chr7	121718969	121718970	chr7_121718364_121720792	-5.982066	-9.803238	9.8438E-06	0.010149368	H3K27me3	LD
chr7	121718973	121718974	chr7_121718364_121720792	-5.982066	-9.803238	9.8438E-06	0.010149368	H3K27me3	LD
chr7	121719165	121719166	chr7_121718364_121720792	-5.982066	-9.803238	9.8438E-06	0.010149368	H3K27me3	LD
chr7	121719525	121719526	chr7_121718364_121720792	-5.982066	-9.803238	9.8438E-06	0.010149368	H3K27me3	LD
chr8	136662533	136662534	chr8_136661252_136663304	-5.5913515	-11.308731	3.3651E-06	0.051892682	H3K27me3	LD
chr9	6019669	6019670	chr9_6018977_6019646	-6.0618994	-11.357661	3.2568E-06	0.085669076	H3K27me3	LD
chr10	14549333	14549334	chr10_14549391_14550775	-3.2014174	-12.772989	1.3305E-06	0.001917785	H3K4me1	LD
chr10	14549580	14549581	chr10_14549391_14550775	-3.2014174	-12.772989	1.3305E-06	0.001917785	H3K4me1	LD
chr10	14549745	14549746	chr10_14549391_14550775	-3.2014174	-12.772989	1.3305E-06	0.001917785	H3K4me1	LD
chr10	14549933	14549934	chr10_14549391_14550775	-3.2014174	-12.772989	1.3305E-06	0.001917785	H3K4me1	LD

chr10	14550054	14550055	chr10_14549391_14550775	-3.2014174	-12.772989	1.3305E-06	0.001917785	H3K4me1	LD
chr10	14550147	14550148	chr10_14549391_14550775	-3.2014174	-12.772989	1.3305E-06	0.001917785	H3K4me1	LD
chr10	14550328	14550329	chr10_14549391_14550775	-3.2014174	-12.772989	1.3305E-06	0.001917785	H3K4me1	LD
chr10	14550393	14550394	chr10_14549391_14550775	-3.2014174	-12.772989	1.3305E-06	0.001917785	H3K4me1	LD
chr10	14550611	14550612	chr10_14549391_14550775	-3.2014174	-12.772989	1.3305E-06	0.001917785	H3K4me1	LD
chr10	14550660	14550661	chr10_14549391_14550775	-3.2014174	-12.772989	1.3305E-06	0.001917785	H3K4me1	LD
chr10	14550725	14550726	chr10_14549391_14550775	-3.2014174	-12.772989	1.3305E-06	0.001917785	H3K4me1	LD
chr10	14550760	14550761	chr10_14549391_14550775	-3.2014174	-12.772989	1.3305E-06	0.001917785	H3K4me1	LD
chr10	14550781	14550782	chr10_14549391_14550775	-3.2014174	-12.772989	1.3305E-06	0.001917785	H3K4me1	LD
chr10	14550792	14550793	chr10_14549391_14550775	-3.2014174	-12.772989	1.3305E-06	0.001917785	H3K4me1	LD
chr10	14550793	14550794	chr10_14549391_14550775	-3.2014174	-12.772989	1.3305E-06	0.001917785	H3K4me1	LD
chr10	14550873	14550874	chr10_14549391_14550775	-3.2014174	-12.772989	1.3305E-06	0.001917785	H3K4me1	LD
chr10	8545435	8545436	chr10_8545454_8545998	-3.3543078	-10.46077	6.058E-06	0.007761952	H3K4me1	LD

chr10	8545805	8545806	chr10_8545454_8545998	-3.3543078	-10.46077	6.058E-06	0.007761952	H3K4me1	LD
chr10	10773134	10773135	chr10_10772961_10773295	3.932371	10.1375706	7.6643E-06	0.008056634	H3K4me1	LD
chr10	8956432	8956433	chr10_8956144_8956768	1.6998272	10.073788	8.0346E-06	0.008056634	H3K4me1	LD
chr10	8956808	8956809	chr10_8956144_8956768	1.6998272	10.073788	8.0346E-06	0.008056634	H3K4me1	LD
chr10	8956823	8956824	chr10_8956144_8956768	1.6998272	10.073788	8.0346E-06	0.008056634	H3K4me1	LD
chr10	8956862	8956863	chr10_8956144_8956768	1.6998272	10.073788	8.0346E-06	0.008056634	H3K4me1	LD
chr11	6497379	6497380	chr11_6497183_6499652	-4.8963592	-10.139166	7.6553E-06	0.007151407	H3K4me1	LD
chr11	6497388	6497389	chr11_6497183_6499652	-4.8963592	-10.139166	7.6553E-06	0.007151407	H3K4me1	LD
chr11	6497428	6497429	chr11_6497183_6499652	-4.8963592	-10.139166	7.6553E-06	0.007151407	H3K4me1	LD
chr11	6497606	6497607	chr11_6497183_6499652	-4.8963592	-10.139166	7.6553E-06	0.007151407	H3K4me1	LD
chr11	6497677	6497678	chr11_6497183_6499652	-4.8963592	-10.139166	7.6553E-06	0.007151407	H3K4me1	LD
chr11	6499036	6499037	chr11_6497183_6499652	-4.8963592	-10.139166	7.6553E-06	0.007151407	H3K4me1	LD
chr11	72562178	72562179	chr11_72561330_72563067	-2.4005643	-9.8550863	9.4645E-06	0.007151407	H3K4me1	LD

chr11	72562186	72562187	chr11_72561330_72563067	-2.4005643	-9.8550863	9.4645E-06	0.007151407	H3K4me1	LD
chr11	72562190	72562191	chr11_72561330_72563067	-2.4005643	-9.8550863	9.4645E-06	0.007151407	H3K4me1	LD
chr11	72562207	72562208	chr11_72561330_72563067	-2.4005643	-9.8550863	9.4645E-06	0.007151407	H3K4me1	LD
chr11	72562219	72562220	chr11_72561330_72563067	-2.4005643	-9.8550863	9.4645E-06	0.007151407	H3K4me1	LD
chr11	72562231	72562232	chr11_72561330_72563067	-2.4005643	-9.8550863	9.4645E-06	0.007151407	H3K4me1	LD
chr11	72562316	72562317	chr11_72561330_72563067	-2.4005643	-9.8550863	9.4645E-06	0.007151407	H3K4me1	LD
chr11	72562594	72562595	chr11_72561330_72563067	-2.4005643	-9.8550863	9.4645E-06	0.007151407	H3K4me1	LD
chr11	72562652	72562653	chr11_72561330_72563067	-2.4005643	-9.8550863	9.4645E-06	0.007151407	H3K4me1	LD
chr11	72562888	72562889	chr11_72561330_72563067	-2.4005643	-9.8550863	9.4645E-06	0.007151407	H3K4me1	LD
chr11	72562900	72562901	chr11_72561330_72563067	-2.4005643	-9.8550863	9.4645E-06	0.007151407	H3K4me1	LD
chr11	72562932	72562933	chr11_72561330_72563067	-2.4005643	-9.8550863	9.4645E-06	0.007151407	H3K4me1	LD
chr11	72562957	72562958	chr11_72561330_72563067	-2.4005643	-9.8550863	9.4645E-06	0.007151407	H3K4me1	LD
chr11	72562985	72562986	chr11_72561330_72563067	-2.4005643	-9.8550863	9.4645E-06	0.007151407	H3K4me1	LD

chr11	72563005	72563006	chr11_72561330_72563067	-2.4005643	-9.8550863	9.4645E-06	0.007151407	H3K4me1	LD
chr11	72563009	72563010	chr11_72561330_72563067	-2.4005643	-9.8550863	9.4645E-06	0.007151407	H3K4me1	LD
chr11	72563017	72563018	chr11_72561330_72563067	-2.4005643	-9.8550863	9.4645E-06	0.007151407	H3K4me1	LD
chr11	72563032	72563033	chr11_72561330_72563067	-2.4005643	-9.8550863	9.4645E-06	0.007151407	H3K4me1	LD
chr11	72563159	72563160	chr11_72561330_72563067	-2.4005643	-9.8550863	9.4645E-06	0.007151407	H3K4me1	LD
chr12	4460802	4460803	chr12_4460150_4461574	-6.5629	-15.629097	2.8012E-07	0.000863746	H3K4me1	LD
chr12	4461161	4461162	chr12_4460150_4461574	-6.5629	-15.629097	2.8012E-07	0.000863746	H3K4me1	LD
chr12	13192624	13192625	chr12_13191830_13193116	5.3849063	15.4418767	3.0762E-07	0.000863746	H3K4me1	LD
chr12	55109710	55109711	chr12_55109709_55110900	-2.1398842	-14.73893	4.415E-07	0.000863746	H3K4me1	LD
chr12	55109722	55109723	chr12_55109709_55110900	-2.1398842	-14.73893	4.415E-07	0.000863746	H3K4me1	LD
chr12	55109741	55109742	chr12_55109709_55110900	-2.1398842	-14.73893	4.415E-07	0.000863746	H3K4me1	LD
chr12	55109746	55109747	chr12_55109709_55110900	-2.1398842	-14.73893	4.415E-07	0.000863746	H3K4me1	LD
chr12	55109813	55109814	chr12_55109709_55110900	-2.1398842	-14.73893	4.415E-07	0.000863746	H3K4me1	LD

chr12	55109933	55109934	chr12_55109709_55110900	-2.1398842	-14.73893	4.415E-07	0.000863746	H3K4me1	LD
chr12	55110411	55110412	chr12_55109709_55110900	-2.1398842	-14.73893	4.415E-07	0.000863746	H3K4me1	LD
chr12	55110489	55110490	chr12_55109709_55110900	-2.1398842	-14.73893	4.415E-07	0.000863746	H3K4me1	LD
chr12	55110575	55110576	chr12_55109709_55110900	-2.1398842	-14.73893	4.415E-07	0.000863746	H3K4me1	LD
chr12	55110865	55110866	chr12_55109709_55110900	-2.1398842	-14.73893	4.415E-07	0.000863746	H3K4me1	LD
chr12	55248982	55248983	chr12_55248905_55249646	-6.465386	-12.277276	1.801E-06	0.003271776	H3K4me1	LD
chr12	59359153	59359154	chr12_59358857_59359765	-5.3824925	-11.939078	2.2286E-06	0.003542555	H3K4me1	LD
chr12	59359827	59359828	chr12_59358857_59359765	-5.3824925	-11.939078	2.2286E-06	0.003542555	H3K4me1	LD
chr12	40883260	40883261	chr12_40882502_40883823	-2.3591698	-11.81554	2.4123E-06	0.003608928	H3K4me1	LD
chr12	25441846	25441847	chr12_25441743_25442707	-8.521332	-11.45209	3.0586E-06	0.003704238	H3K4me1	LD
chr12	25441848	25441849	chr12_25441743_25442707	-8.521332	-11.45209	3.0586E-06	0.003704238	H3K4me1	LD
chr12	25441930	25441931	chr12_25441743_25442707	-8.521332	-11.45209	3.0586E-06	0.003704238	H3K4me1	LD
chr12	25442662	25442663	chr12_25441743_25442707	-8.521332	-11.45209	3.0586E-06	0.003704238	H3K4me1	LD

chr12	60970701	60970702	chr12_60969603_60972710	4.6657183	10.2073423	7.2809E-06	0.007966209	H3K4me1	LD
chr12	25453909	25453910	chr12_25453715_25455510	-6.835832	-10.108515	7.8306E-06	0.007966209	H3K4me1	LD
chr12	25453952	25453953	chr12_25453715_25455510	-6.835832	-10.108515	7.8306E-06	0.007966209	H3K4me1	LD
chr12	25455414	25455415	chr12_25453715_25455510	-6.835832	-10.108515	7.8306E-06	0.007966209	H3K4me1	LD
chr13	121329061	121329062	chr13_121328978_121330882	1.8432283	17.5506605	1.1344E-07	0.000950503	H3K4me1	LD
chr13	121329133	121329134	chr13_121328978_121330882	1.8432283	17.5506605	1.1344E-07	0.000950503	H3K4me1	LD
chr13	121329243	121329244	chr13_121328978_121330882	1.8432283	17.5506605	1.1344E-07	0.000950503	H3K4me1	LD
chr13	121329279	121329280	chr13_121328978_121330882	1.8432283	17.5506605	1.1344E-07	0.000950503	H3K4me1	LD
chr13	129944949	129944950	chr13_129944377_129945410	-3.3629792	-12.682888	1.4046E-06	0.004430124	H3K4me1	LD
chr13	70030060	70030061	chr13_70029590_70030976	7.355585	11.5300638	2.9051E-06	0.004430124	H3K4me1	LD
chr13	70030123	70030124	chr13_70029590_70030976	7.355585	11.5300638	2.9051E-06	0.004430124	H3K4me1	LD
chr13	70030432	70030433	chr13_70029590_70030976	7.355585	11.5300638	2.9051E-06	0.004430124	H3K4me1	LD
chr13	70030589	70030590	chr13_70029590_70030976	7.355585	11.5300638	2.9051E-06	0.004430124	H3K4me1	LD

chr13	70030874	70030875	chr13_70029590_70030976	7.355585	11.5300638	2.9051E-06	0.004430124	H3K4me1	LD
chr13	84450318	84450319	chr13_84449910_84451168	3.066546	11.1564843	3.7287E-06	0.004430124	H3K4me1	LD
chr13	84450862	84450863	chr13_84449910_84451168	3.066546	11.1564843	3.7287E-06	0.004430124	H3K4me1	LD
chr13	84451175	84451176	chr13_84449910_84451168	3.066546	11.1564843	3.7287E-06	0.004430124	H3K4me1	LD
chr13	1492142	1492143	chr13_1492079_1493259	-2.936948	-11.018084	4.0977E-06	0.004430124	H3K4me1	LD
chr13	1492168	1492169	chr13_1492079_1493259	-2.936948	-11.018084	4.0977E-06	0.004430124	H3K4me1	LD
chr13	1492234	1492235	chr13_1492079_1493259	-2.936948	-11.018084	4.0977E-06	0.004430124	H3K4me1	LD
chr13	1492308	1492309	chr13_1492079_1493259	-2.936948	-11.018084	4.0977E-06	0.004430124	H3K4me1	LD
chr13	1492354	1492355	chr13_1492079_1493259	-2.936948	-11.018084	4.0977E-06	0.004430124	H3K4me1	LD
chr13	1492366	1492367	chr13_1492079_1493259	-2.936948	-11.018084	4.0977E-06	0.004430124	H3K4me1	LD
chr13	1492466	1492467	chr13_1492079_1493259	-2.936948	-11.018084	4.0977E-06	0.004430124	H3K4me1	LD
chr13	1492479	1492480	chr13_1492079_1493259	-2.936948	-11.018084	4.0977E-06	0.004430124	H3K4me1	LD
chr13	1492516	1492517	chr13_1492079_1493259	-2.936948	-11.018084	4.0977E-06	0.004430124	H3K4me1	LD

chr13	1492764	1492765	chr13_1492079_1493259	-2.936948	-11.018084	4.0977E-06	0.004430124	H3K4me1	LD
chr13	1492776	1492777	chr13_1492079_1493259	-2.936948	-11.018084	4.0977E-06	0.004430124	H3K4me1	LD
chr13	1492784	1492785	chr13_1492079_1493259	-2.936948	-11.018084	4.0977E-06	0.004430124	H3K4me1	LD
chr13	1492839	1492840	chr13_1492079_1493259	-2.936948	-11.018084	4.0977E-06	0.004430124	H3K4me1	LD
chr13	1492951	1492952	chr13_1492079_1493259	-2.936948	-11.018084	4.0977E-06	0.004430124	H3K4me1	LD
chr13	1492973	1492974	chr13_1492079_1493259	-2.936948	-11.018084	4.0977E-06	0.004430124	H3K4me1	LD
chr13	1493066	1493067	chr13_1492079_1493259	-2.936948	-11.018084	4.0977E-06	0.004430124	H3K4me1	LD
chr13	1493158	1493159	chr13_1492079_1493259	-2.936948	-11.018084	4.0977E-06	0.004430124	H3K4me1	LD
chr13	1493178	1493179	chr13_1492079_1493259	-2.936948	-11.018084	4.0977E-06	0.004430124	H3K4me1	LD
chr13	115642792	115642793	chr13_115642559_115643656	-2.4077617	-10.551107	5.679E-06	0.005767629	H3K4me1	LD
chr13	115642952	115642953	chr13_115642559_115643656	-2.4077617	-10.551107	5.679E-06	0.005767629	H3K4me1	LD
chr14	109738220	109738221	chr14_109737889_109738957	-2.9929473	-17.609462	1.1051E-07	0.001047867	H3K4me1	LD
chr14	109738268	109738269	chr14_109737889_109738957	-2.9929473	-17.609462	1.1051E-07	0.001047867	H3K4me1	LD

chr14	109738360	109738361	chr14_109737889_109738957	-2.9929473	-17.609462	1.1051E-07	0.001047867	H3K4me1	LD
chr14	78583347	78583348	chr14_78582820_78583769	-3.2477918	-13.657108	7.9554E-07	0.001047867	H3K4me1	LD
chr14	58514121	58514122	chr14_58514156_58515276	-3.1614244	-13.237414	1.0115E-06	0.001047867	H3K4me1	LD
chr14	58514201	58514202	chr14_58514156_58515276	-3.1614244	-13.237414	1.0115E-06	0.001047867	H3K4me1	LD
chr14	58514219	58514220	chr14_58514156_58515276	-3.1614244	-13.237414	1.0115E-06	0.001047867	H3K4me1	LD
chr14	58514232	58514233	chr14_58514156_58515276	-3.1614244	-13.237414	1.0115E-06	0.001047867	H3K4me1	LD
chr14	58514330	58514331	chr14_58514156_58515276	-3.1614244	-13.237414	1.0115E-06	0.001047867	H3K4me1	LD
chr14	58514331	58514332	chr14_58514156_58515276	-3.1614244	-13.237414	1.0115E-06	0.001047867	H3K4me1	LD
chr14	58514507	58514508	chr14_58514156_58515276	-3.1614244	-13.237414	1.0115E-06	0.001047867	H3K4me1	LD
chr14	58514522	58514523	chr14_58514156_58515276	-3.1614244	-13.237414	1.0115E-06	0.001047867	H3K4me1	LD
chr14	58514658	58514659	chr14_58514156_58515276	-3.1614244	-13.237414	1.0115E-06	0.001047867	H3K4me1	LD
chr14	58514662	58514663	chr14_58514156_58515276	-3.1614244	-13.237414	1.0115E-06	0.001047867	H3K4me1	LD
chr14	58514717	58514718	chr14_58514156_58515276	-3.1614244	-13.237414	1.0115E-06	0.001047867	H3K4me1	LD

chr14	58514721	58514722	chr14_58514156_58515276	-3.1614244	-13.237414	1.0115E-06	0.001047867	H3K4me1	LD
chr14	58514795	58514796	chr14_58514156_58515276	-3.1614244	-13.237414	1.0115E-06	0.001047867	H3K4me1	LD
chr14	58514803	58514804	chr14_58514156_58515276	-3.1614244	-13.237414	1.0115E-06	0.001047867	H3K4me1	LD
chr14	58514806	58514807	chr14_58514156_58515276	-3.1614244	-13.237414	1.0115E-06	0.001047867	H3K4me1	LD
chr14	58514816	58514817	chr14_58514156_58515276	-3.1614244	-13.237414	1.0115E-06	0.001047867	H3K4me1	LD
chr14	58514847	58514848	chr14_58514156_58515276	-3.1614244	-13.237414	1.0115E-06	0.001047867	H3K4me1	LD
chr14	58514884	58514885	chr14_58514156_58515276	-3.1614244	-13.237414	1.0115E-06	0.001047867	H3K4me1	LD
chr14	58514887	58514888	chr14_58514156_58515276	-3.1614244	-13.237414	1.0115E-06	0.001047867	H3K4me1	LD
chr14	58514920	58514921	chr14_58514156_58515276	-3.1614244	-13.237414	1.0115E-06	0.001047867	H3K4me1	LD
chr14	58514926	58514927	chr14_58514156_58515276	-3.1614244	-13.237414	1.0115E-06	0.001047867	H3K4me1	LD
chr14	58514933	58514934	chr14_58514156_58515276	-3.1614244	-13.237414	1.0115E-06	0.001047867	H3K4me1	LD
chr14	58515095	58515096	chr14_58514156_58515276	-3.1614244	-13.237414	1.0115E-06	0.001047867	H3K4me1	LD
chr14	58515137	58515138	chr14_58514156_58515276	-3.1614244	-13.237414	1.0115E-06	0.001047867	H3K4me1	LD

chr14	58515246	58515247	chr14_58514156_58515276	-3.1614244	-13.237414	1.0115E-06	0.001047867	H3K4me1	LD
chr14	16637265	16637266	chr14_16636982_16637716	-3.9174053	-13.040488	1.1349E-06	0.001047867	H3K4me1	LD
chr14	16637270	16637271	chr14_16636982_16637716	-3.9174053	-13.040488	1.1349E-06	0.001047867	H3K4me1	LD
chr14	16637456	16637457	chr14_16636982_16637716	-3.9174053	-13.040488	1.1349E-06	0.001047867	H3K4me1	LD
chr14	16637494	16637495	chr14_16636982_16637716	-3.9174053	-13.040488	1.1349E-06	0.001047867	H3K4me1	LD
chr14	16637573	16637574	chr14_16636982_16637716	-3.9174053	-13.040488	1.1349E-06	0.001047867	H3K4me1	LD
chr14	74150090	74150091	chr14_74150141_74151492	5.4354343	11.2995013	3.3859E-06	0.002872745	H3K4me1	LD
chr14	74150102	74150103	chr14_74150141_74151492	5.4354343	11.2995013	3.3859E-06	0.002872745	H3K4me1	LD
chr14	74150142	74150143	chr14_74150141_74151492	5.4354343	11.2995013	3.3859E-06	0.002872745	H3K4me1	LD
chr14	135144981	135144982	chr14_135144872_135145435	-4.612819	-11.22852	3.5514E-06	0.002933859	H3K4me1	LD
chr14	10206303	10206304	chr14_10205615_10206418	-5.7047933	-11.146685	3.7536E-06	0.003021322	H3K4me1	LD
chr14	129182981	129182982	chr14_129183076_129184065	-3.3423338	-10.784538	4.8166E-06	0.003511309	H3K4me1	LD
chr14	129182982	129182983	chr14_129183076_129184065	-3.3423338	-10.784538	4.8166E-06	0.003511309	H3K4me1	LD

chr14	14751512	14751513	chr14_14751585_14753559	-3.2719665	-10.71236	5.0666E-06	0.003511309	H3K4me1	LD
chr14	14751883	14751884	chr14_14751585_14753559	-3.2719665	-10.71236	5.0666E-06	0.003511309	H3K4me1	LD
chr14	14752001	14752002	chr14_14751585_14753559	-3.2719665	-10.71236	5.0666E-06	0.003511309	H3K4me1	LD
chr14	56730813	56730814	chr14_56730689_56731255	-6.3581697	-10.690452	5.1453E-06	0.003511309	H3K4me1	LD
chr14	56731199	56731200	chr14_56730689_56731255	-6.3581697	-10.690452	5.1453E-06	0.003511309	H3K4me1	LD
chr14	114447576	114447577	chr14_114446620_114447935	-4.4943948	-10.422813	6.2256E-06	0.004158153	H3K4me1	LD
chr14	30966527	30966528	chr14_30966376_30966704	-3.7570405	-10.356207	6.5323E-06	0.004272146	H3K4me1	LD
chr14	58448394	58448395	chr14_58448368_58449736	-2.159536	-10.201866	7.3102E-06	0.004329832	H3K4me1	LD
chr14	58448451	58448452	chr14_58448368_58449736	-2.159536	-10.201866	7.3102E-06	0.004329832	H3K4me1	LD
chr14	58448549	58448550	chr14_58448368_58449736	-2.159536	-10.201866	7.3102E-06	0.004329832	H3K4me1	LD
chr14	58448563	58448564	chr14_58448368_58449736	-2.159536	-10.201866	7.3102E-06	0.004329832	H3K4me1	LD
chr14	58448576	58448577	chr14_58448368_58449736	-2.159536	-10.201866	7.3102E-06	0.004329832	H3K4me1	LD
chr14	10694515	10694516	chr14_10694182_10695009	0.5184637	9.9311267	8.9374E-06	0.005195634	H3K4me1	LD

chr15	31898864	31898865	chr15_31898553_31899396	-4.40129	-18.774144	6.6933E-08	0.000940514	H3K4me1	LD
chr15	138945634	138945635	chr15_138945587_138947451	-3.3318746	-14.230786	5.7915E-07	0.000940514	H3K4me1	LD
chr15	138945698	138945699	chr15_138945587_138947451	-3.3318746	-14.230786	5.7915E-07	0.000940514	H3K4me1	LD
chr15	138945872	138945873	chr15_138945587_138947451	-3.3318746	-14.230786	5.7915E-07	0.000940514	H3K4me1	LD
chr15	138945932	138945933	chr15_138945587_138947451	-3.3318746	-14.230786	5.7915E-07	0.000940514	H3K4me1	LD
chr15	138946019	138946020	chr15_138945587_138947451	-3.3318746	-14.230786	5.7915E-07	0.000940514	H3K4me1	LD
chr15	138946030	138946031	chr15_138945587_138947451	-3.3318746	-14.230786	5.7915E-07	0.000940514	H3K4me1	LD
chr15	138946060	138946061	chr15_138945587_138947451	-3.3318746	-14.230786	5.7915E-07	0.000940514	H3K4me1	LD
chr15	138946069	138946070	chr15_138945587_138947451	-3.3318746	-14.230786	5.7915E-07	0.000940514	H3K4me1	LD
chr15	138946131	138946132	chr15_138945587_138947451	-3.3318746	-14.230786	5.7915E-07	0.000940514	H3K4me1	LD
chr15	138946248	138946249	chr15_138945587_138947451	-3.3318746	-14.230786	5.7915E-07	0.000940514	H3K4me1	LD
chr15	138946297	138946298	chr15_138945587_138947451	-3.3318746	-14.230786	5.7915E-07	0.000940514	H3K4me1	LD
chr15	138946444	138946445	chr15_138945587_138947451	-3.3318746	-14.230786	5.7915E-07	0.000940514	H3K4me1	LD

chr15	138946446	138946447	chr15_138945587_138947451	-3.3318746	-14.230786	5.7915E-07	0.000940514	H3K4me1	LD
chr15	138946519	138946520	chr15_138945587_138947451	-3.3318746	-14.230786	5.7915E-07	0.000940514	H3K4me1	LD
chr15	138947172	138947173	chr15_138945587_138947451	-3.3318746	-14.230786	5.7915E-07	0.000940514	H3K4me1	LD
chr15	138947499	138947500	chr15_138945587_138947451	-3.3318746	-14.230786	5.7915E-07	0.000940514	H3K4me1	LD
chr15	138947527	138947528	chr15_138945587_138947451	-3.3318746	-14.230786	5.7915E-07	0.000940514	H3K4me1	LD
chr15	46183924	46183925	chr15_46184008_46184417	-2.6819666	-11.670711	2.6495E-06	0.002700274	H3K4me1	LD
chr15	46184035	46184036	chr15_46184008_46184417	-2.6819666	-11.670711	2.6495E-06	0.002700274	H3K4me1	LD
chr15	46184046	46184047	chr15_46184008_46184417	-2.6819666	-11.670711	2.6495E-06	0.002700274	H3K4me1	LD
chr15	46184070	46184071	chr15_46184008_46184417	-2.6819666	-11.670711	2.6495E-06	0.002700274	H3K4me1	LD
chr15	46184147	46184148	chr15_46184008_46184417	-2.6819666	-11.670711	2.6495E-06	0.002700274	H3K4me1	LD
chr15	46184176	46184177	chr15_46184008_46184417	-2.6819666	-11.670711	2.6495E-06	0.002700274	H3K4me1	LD
chr15	46184262	46184263	chr15_46184008_46184417	-2.6819666	-11.670711	2.6495E-06	0.002700274	H3K4me1	LD
chr15	46184354	46184355	chr15_46184008_46184417	-2.6819666	-11.670711	2.6495E-06	0.002700274	H3K4me1	LD

chr15	46184428	46184429	chr15_46184008_46184417	-2.6819666	-11.670711	2.6495E-06	0.002700274	H3K4me1	LD
chr15	3502430	3502431	chr15_3502146_3502876	-3.0845203	-11.503664	2.9561E-06	0.002700274	H3K4me1	LD
chr15	3502670	3502671	chr15_3502146_3502876	-3.0845203	-11.503664	2.9561E-06	0.002700274	H3K4me1	LD
chr15	3502699	3502700	chr15_3502146_3502876	-3.0845203	-11.503664	2.9561E-06	0.002700274	H3K4me1	LD
chr15	3502876	3502877	chr15_3502146_3502876	-3.0845203	-11.503664	2.9561E-06	0.002700274	H3K4me1	LD
chr15	3502943	3502944	chr15_3502146_3502876	-3.0845203	-11.503664	2.9561E-06	0.002700274	H3K4me1	LD
chr15	74488680	74488681	chr15_74487914_74489864	-5.0898346	-11.024987	4.0783E-06	0.003213343	H3K4me1	LD
chr15	74488756	74488757	chr15_74487914_74489864	-5.0898346	-11.024987	4.0783E-06	0.003213343	H3K4me1	LD
chr15	74488759	74488760	chr15_74487914_74489864	-5.0898346	-11.024987	4.0783E-06	0.003213343	H3K4me1	LD
chr15	74488783	74488784	chr15_74487914_74489864	-5.0898346	-11.024987	4.0783E-06	0.003213343	H3K4me1	LD
chr15	137611355	137611356	chr15_137611218_137611875	-4.0238214	-10.990045	4.1773E-06	0.003213343	H3K4me1	LD
chr15	137611596	137611597	chr15_137611218_137611875	-4.0238214	-10.990045	4.1773E-06	0.003213343	H3K4me1	LD
chr15	131560031	131560032	chr15_131559745_131560546	-3.6670824	-10.076729	8.0171E-06	0.005449962	H3K4me1	LD

chr15	131560083	131560084	chr15_131559745_131560546	-3.6670824	-10.076729	8.0171E-06	0.005449962	H3K4me1	LD
chr15	131560116	131560117	chr15_131559745_131560546	-3.6670824	-10.076729	8.0171E-06	0.005449962	H3K4me1	LD
chr15	131560531	131560532	chr15_131559745_131560546	-3.6670824	-10.076729	8.0171E-06	0.005449962	H3K4me1	LD
chr15	131560600	131560601	chr15_131559745_131560546	-3.6670824	-10.076729	8.0171E-06	0.005449962	H3K4me1	LD
chr16	52198215	52198216	chr16_52197794_52199096	2.985172	15.7627504	2.6218E-07	0.001258272	H3K4me1	LD
chr16	52198510	52198511	chr16_52197794_52199096	2.985172	15.7627504	2.6218E-07	0.001258272	H3K4me1	LD
chr16	71746896	71746897	chr16_71746874_71747350	-9.4997696	-14.472708	5.0839E-07	0.001258272	H3K4me1	LD
chr16	71747129	71747130	chr16_71746874_71747350	-9.4997696	-14.472708	5.0839E-07	0.001258272	H3K4me1	LD
chr16	71747262	71747263	chr16_71746874_71747350	-9.4997696	-14.472708	5.0839E-07	0.001258272	H3K4me1	LD
chr16	71747263	71747264	chr16_71746874_71747350	-9.4997696	-14.472708	5.0839E-07	0.001258272	H3K4me1	LD
chr16	71745097	71745098	chr16_71744995_71746141	-7.47108	-12.373713	1.6965E-06	0.002099424	H3K4me1	LD
chr16	71745132	71745133	chr16_71744995_71746141	-7.47108	-12.373713	1.6965E-06	0.002099424	H3K4me1	LD
chr16	71745174	71745175	chr16_71744995_71746141	-7.47108	-12.373713	1.6965E-06	0.002099424	H3K4me1	LD

chr16	71745944	71745945	chr16_71744995_71746141	-7.47108	-12.373713	1.6965E-06	0.002099424	H3K4me1	LD
chr16	71746024	71746025	chr16_71744995_71746141	-7.47108	-12.373713	1.6965E-06	0.002099424	H3K4me1	LD
chr16	71746135	71746136	chr16_71744995_71746141	-7.47108	-12.373713	1.6965E-06	0.002099424	H3K4me1	LD
chr16	22397113	22397114	chr16_22396670_22397869	-5.2557382	-11.595272	2.7833E-06	0.003179393	H3K4me1	LD
chr16	48076840	48076841	chr16_48076940_48078363	-1.5670566	-10.262836	6.9912E-06	0.005767715	H3K4me1	LD
chr16	48077509	48077510	chr16_48076940_48078363	-1.5670566	-10.262836	6.9912E-06	0.005767715	H3K4me1	LD
chr16	48077672	48077673	chr16_48076940_48078363	1.5670566	10.2628365	6.9912E-06	0.005767715	H3K4me1	LD
chr16	48077902	48077903	chr16_48076940_48078363	1.5670566	10.2628365	6.9912E-06	0.005767715	H3K4me1	LD
chr16	48077940	48077941	chr16_48076940_48078363	1.5670566	10.2628365	6.9912E-06	0.005767715	H3K4me1	LD
chr17	50659393	50659394	chr17_50659235_50659858	-2.590417	-17.776501	1.0265E-07	0.001702319	H3K4me1	LD
chr17	24983751	24983752	chr17_24983838_24985841	3.370852	11.5281958	2.9087E-06	0.001809549	H3K4me1	LD
chr17	24983778	24983779	chr17_24983838_24985841	3.370852	11.5281958	2.9087E-06	0.001809549	H3K4me1	LD
chr17	24983785	24983786	chr17_24983838_24985841	3.370852	11.5281958	2.9087E-06	0.001809549	H3K4me1	LD

chr17	24984010	24984011	chr17_24983838_24985841	3.370852	11.5281958	2.9087E-06	0.001809549	H3K4me1	LD
chr17	24984286	24984287	chr17_24983838_24985841	3.370852	11.5281958	2.9087E-06	0.001809549	H3K4me1	LD
chr17	24984307	24984308	chr17_24983838_24985841	3.370852	11.5281958	2.9087E-06	0.001809549	H3K4me1	LD
chr17	24984681	24984682	chr17_24983838_24985841	3.370852	11.5281958	2.9087E-06	0.001809549	H3K4me1	LD
chr17	24984718	24984719	chr17_24983838_24985841	3.370852	11.5281958	2.9087E-06	0.001809549	H3K4me1	LD
chr17	24984777	24984778	chr17_24983838_24985841	3.370852	11.5281958	2.9087E-06	0.001809549	H3K4me1	LD
chr17	24984865	24984866	chr17_24983838_24985841	3.370852	11.5281958	2.9087E-06	0.001809549	H3K4me1	LD
chr17	24984919	24984920	chr17_24983838_24985841	3.370852	11.5281958	2.9087E-06	0.001809549	H3K4me1	LD
chr17	24984980	24984981	chr17_24983838_24985841	3.370852	11.5281958	2.9087E-06	0.001809549	H3K4me1	LD
chr17	24985120	24985121	chr17_24983838_24985841	3.370852	11.5281958	2.9087E-06	0.001809549	H3K4me1	LD
chr17	24985222	24985223	chr17_24983838_24985841	3.370852	11.5281958	2.9087E-06	0.001809549	H3K4me1	LD
chr17	24985355	24985356	chr17_24983838_24985841	3.370852	11.5281958	2.9087E-06	0.001809549	H3K4me1	LD
chr17	24985413	24985414	chr17_24983838_24985841	3.370852	11.5281958	2.9087E-06	0.001809549	H3K4me1	LD

chr17	24985417	24985418	chr17_24983838_24985841	3.370852	11.5281958	2.9087E-06	0.001809549	H3K4me1	LD
chr17	51441530	51441531	chr17_51441033_51442801	4.6807798	11.3293169	3.319E-06	0.001809549	H3K4me1	LD
chr17	51441606	51441607	chr17_51441033_51442801	4.6807798	11.3293169	3.319E-06	0.001809549	H3K4me1	LD
chr17	51441727	51441728	chr17_51441033_51442801	4.6807798	11.3293169	3.319E-06	0.001809549	H3K4me1	LD
chr17	51441765	51441766	chr17_51441033_51442801	4.6807798	11.3293169	3.319E-06	0.001809549	H3K4me1	LD
chr17	51441781	51441782	chr17_51441033_51442801	4.6807798	11.3293169	3.319E-06	0.001809549	H3K4me1	LD
chr17	51441794	51441795	chr17_51441033_51442801	4.6807798	11.3293169	3.319E-06	0.001809549	H3K4me1	LD
chr17	51441809	51441810	chr17_51441033_51442801	4.6807798	11.3293169	3.319E-06	0.001809549	H3K4me1	LD
chr17	51441982	51441983	chr17_51441033_51442801	4.6807798	11.3293169	3.319E-06	0.001809549	H3K4me1	LD
chr17	51442125	51442126	chr17_51441033_51442801	4.6807798	11.3293169	3.319E-06	0.001809549	H3K4me1	LD
chr17	51442450	51442451	chr17_51441033_51442801	4.6807798	11.3293169	3.319E-06	0.001809549	H3K4me1	LD
chr17	51442633	51442634	chr17_51441033_51442801	4.6807798	11.3293169	3.319E-06	0.001809549	H3K4me1	LD
chr17	51442650	51442651	chr17_51441033_51442801	4.6807798	11.3293169	3.319E-06	0.001809549	H3K4me1	LD

chr17	14719078	14719079	chr17_14717861_14719946	-3.7815501	-11.12124	3.819E-06	0.001809549	H3K4me1	LD
chr17	14719175	14719176	chr17_14717861_14719946	-3.7815501	-11.12124	3.819E-06	0.001809549	H3K4me1	LD
chr17	14719258	14719259	chr17_14717861_14719946	-3.7815501	-11.12124	3.819E-06	0.001809549	H3K4me1	LD
chr17	14719549	14719550	chr17_14717861_14719946	-3.7815501	-11.12124	3.819E-06	0.001809549	H3K4me1	LD
chr17	14719769	14719770	chr17_14717861_14719946	-3.7815501	-11.12124	3.819E-06	0.001809549	H3K4me1	LD
chr17	38318882	38318883	chr17_38318836_38319623	7.2246604	9.88704297	9.2389E-06	0.003830452	H3K4me1	LD
chr17	38319400	38319401	chr17_38318836_38319623	7.2246604	9.88704297	9.2389E-06	0.003830452	H3K4me1	LD
chr17	38319476	38319477	chr17_38318836_38319623	7.2246604	9.88704297	9.2389E-06	0.003830452	H3K4me1	LD
chr17	38319493	38319494	chr17_38318836_38319623	7.2246604	9.88704297	9.2389E-06	0.003830452	H3K4me1	LD
chr17	38319508	38319509	chr17_38318836_38319623	7.2246604	9.88704297	9.2389E-06	0.003830452	H3K4me1	LD
chr18	25671426	25671427	chr18_25671484_25672834	-3.5436061	-11.01811	4.0976E-06	0.003604129	H3K4me1	LD
chr18	25671502	25671503	chr18_25671484_25672834	-3.5436061	-11.01811	4.0976E-06	0.003604129	H3K4me1	LD
chr18	25671567	25671568	chr18_25671484_25672834	-3.5436061	-11.01811	4.0976E-06	0.003604129	H3K4me1	LD

chr18	25671603	25671604	chr18_25671484_25672834	-3.5436061	-11.01811	4.0976E-06	0.003604129	H3K4me1	LD
chr18	25671618	25671619	chr18_25671484_25672834	-3.5436061	-11.01811	4.0976E-06	0.003604129	H3K4me1	LD
chr18	25671645	25671646	chr18_25671484_25672834	-3.5436061	-11.01811	4.0976E-06	0.003604129	H3K4me1	LD
chr18	25671677	25671678	chr18_25671484_25672834	-3.5436061	-11.01811	4.0976E-06	0.003604129	H3K4me1	LD
chr18	25671763	25671764	chr18_25671484_25672834	-3.5436061	-11.01811	4.0976E-06	0.003604129	H3K4me1	LD
chr18	25671786	25671787	chr18_25671484_25672834	-3.5436061	-11.01811	4.0976E-06	0.003604129	H3K4me1	LD
chr18	25671822	25671823	chr18_25671484_25672834	-3.5436061	-11.01811	4.0976E-06	0.003604129	H3K4me1	LD
chr18	25671903	25671904	chr18_25671484_25672834	-3.5436061	-11.01811	4.0976E-06	0.003604129	H3K4me1	LD
chr18	25672053	25672054	chr18_25671484_25672834	-3.5436061	-11.01811	4.0976E-06	0.003604129	H3K4me1	LD
chr18	25672065	25672066	chr18_25671484_25672834	-3.5436061	-11.01811	4.0976E-06	0.003604129	H3K4me1	LD
chr18	25672254	25672255	chr18_25671484_25672834	-3.5436061	-11.01811	4.0976E-06	0.003604129	H3K4me1	LD
chr18	36366053	36366054	chr18_36365710_36366712	-4.6136181	-10.839712	4.6349E-06	0.003604129	H3K4me1	LD
chr18	1510248	1510249	chr18_1510332_1511375	4.2264522	10.5237421	5.7909E-06	0.003604129	H3K4me1	LD

chr18	1510265	1510266	chr18_1510332_1511375	4.2264522	10.5237421	5.7909E-06	0.003604129	H3K4me1	LD
chr18	1510354	1510355	chr18_1510332_1511375	4.2264522	10.5237421	5.7909E-06	0.003604129	H3K4me1	LD
chr18	1510508	1510509	chr18_1510332_1511375	4.2264522	10.5237421	5.7909E-06	0.003604129	H3K4me1	LD
chr18	1510801	1510802	chr18_1510332_1511375	4.2264522	10.5237421	5.7909E-06	0.003604129	H3K4me1	LD
chr18	1510816	1510817	chr18_1510332_1511375	4.2264522	10.5237421	5.7909E-06	0.003604129	H3K4me1	LD
chr18	1510825	1510826	chr18_1510332_1511375	4.2264522	10.5237421	5.7909E-06	0.003604129	H3K4me1	LD
chr18	1511023	1511024	chr18_1510332_1511375	4.2264522	10.5237421	5.7909E-06	0.003604129	H3K4me1	LD
chr18	1511395	1511396	chr18_1510332_1511375	4.2264522	10.5237421	5.7909E-06	0.003604129	H3K4me1	LD
chr1	231236367	231236368	chr1_231235854_231236803	-3.6679967	-21.559559	2.2564E-08	0.000598148	H3K4me1	LD
chr1	231236432	231236433	chr1_231235854_231236803	-3.6679967	-21.559559	2.2564E-08	0.000598148	H3K4me1	LD
chr1	142412685	142412686	chr1_142412602_142414622	-2.321424	-13.343001	9.5158E-07	0.009576498	H3K4me1	LD
chr1	142414313	142414314	chr1_142412602_142414622	-2.321424	-13.343001	9.5158E-07	0.009576498	H3K4me1	LD
chr1	15880988	15880989	chr1_15880491_15881352	-3.34956	-12.98438	1.1731E-06	0.009576498	H3K4me1	LD

chr1	164170854	164170855	chr1_164170457_164170775	-1.8471299	-12.653901	1.4294E-06	0.009576498	H3K4me1	LD
chr1	183891013	183891014	chr1_183890945_183892076	7.3677848	11.1080237	3.8535E-06	0.009576498	H3K4me1	LD
chr1	183891843	183891844	chr1_183890945_183892076	7.3677848	11.1080237	3.8535E-06	0.009576498	H3K4me1	LD
chr1	9455728	9455729	chr1_9455289_9456690	-2.4036859	-10.966602	4.2452E-06	0.009576498	H3K4me1	LD
chr1	9456013	9456014	chr1_9455289_9456690	-2.4036859	-10.966602	4.2452E-06	0.009576498	H3K4me1	LD
chr1	9456070	9456071	chr1_9455289_9456690	-2.4036859	-10.966602	4.2452E-06	0.009576498	H3K4me1	LD
chr1	9456143	9456144	chr1_9455289_9456690	-2.4036859	-10.966602	4.2452E-06	0.009576498	H3K4me1	LD
chr1	9456279	9456280	chr1_9455289_9456690	-2.4036859	-10.966602	4.2452E-06	0.009576498	H3K4me1	LD
chr1	9456335	9456336	chr1_9455289_9456690	-2.4036859	-10.966602	4.2452E-06	0.009576498	H3K4me1	LD
chr1	9456346	9456347	chr1_9455289_9456690	-2.4036859	-10.966602	4.2452E-06	0.009576498	H3K4me1	LD
chr1	9456426	9456427	chr1_9455289_9456690	-2.4036859	-10.966602	4.2452E-06	0.009576498	H3K4me1	LD
chr1	9456669	9456670	chr1_9455289_9456690	-2.4036859	-10.966602	4.2452E-06	0.009576498	H3K4me1	LD
chr1	9456776	9456777	chr1_9455289_9456690	-2.4036859	-10.966602	4.2452E-06	0.009576498	H3K4me1	LD

chr1	7462119	7462120	chr1_7461709_7463688	9.404932	10.8133892	4.7206E-06	0.009576498	H3K4me1	LD
chr1	7462541	7462542	chr1_7461709_7463688	9.404932	10.8133892	4.7206E-06	0.009576498	H3K4me1	LD
chr1	7431868	7431869	chr1_7431530_7432112	11.125286	10.6757203	5.199E-06	0.009576498	H3K4me1	LD
chr1	247469983	247469984	chr1_247469878_247470244	-3.4455748	-10.432961	6.1803E-06	0.009576498	H3K4me1	LD
chr1	201416910	201416911	chr1_201416118_201417157	-3.048472	-10.408934	6.2881E-06	0.009576498	H3K4me1	LD
chr1	161890861	161890862	chr1_161890618_161892665	-2.755048	-10.288046	6.8638E-06	0.009576498	H3K4me1	LD
chr1	161890899	161890900	chr1_161890618_161892665	-2.755048	-10.288046	6.8638E-06	0.009576498	H3K4me1	LD
chr1	161890974	161890975	chr1_161890618_161892665	-2.755048	-10.288046	6.8638E-06	0.009576498	H3K4me1	LD
chr1	161891152	161891153	chr1_161890618_161892665	-2.755048	-10.288046	6.8638E-06	0.009576498	H3K4me1	LD
chr1	161891310	161891311	chr1_161890618_161892665	-2.755048	-10.288046	6.8638E-06	0.009576498	H3K4me1	LD
chr1	161891358	161891359	chr1_161890618_161892665	-2.755048	-10.288046	6.8638E-06	0.009576498	H3K4me1	LD
chr1	161891453	161891454	chr1_161890618_161892665	-2.755048	-10.288046	6.8638E-06	0.009576498	H3K4me1	LD
chr1	161891715	161891716	chr1_161890618_161892665	-2.755048	-10.288046	6.8638E-06	0.009576498	H3K4me1	LD

chr1	161891770	161891771	chr1_161890618_161892665	-2.755048	-10.288046	6.8638E-06	0.009576498	H3K4me1	LD
chr1	161891979	161891980	chr1_161890618_161892665	-2.755048	-10.288046	6.8638E-06	0.009576498	H3K4me1	LD
chr1	161892264	161892265	chr1_161890618_161892665	-2.755048	-10.288046	6.8638E-06	0.009576498	H3K4me1	LD
chr1	161892269	161892270	chr1_161890618_161892665	-2.755048	-10.288046	6.8638E-06	0.009576498	H3K4me1	LD
chr1	161892341	161892342	chr1_161890618_161892665	-2.755048	-10.288046	6.8638E-06	0.009576498	H3K4me1	LD
chr1	161892575	161892576	chr1_161890618_161892665	-2.755048	-10.288046	6.8638E-06	0.009576498	H3K4me1	LD
chr1	161892602	161892603	chr1_161890618_161892665	-2.755048	-10.288046	6.8638E-06	0.009576498	H3K4me1	LD
chr1	269142999	269143000	chr1_269143098_269144634	-1.9424473	-9.8364904	9.5987E-06	0.012412242	H3K4me1	LD
chr1	269144419	269144420	chr1_269143098_269144634	-1.9424473	-9.8364904	9.5987E-06	0.012412242	H3K4me1	LD
chr1	269144596	269144597	chr1_269143098_269144634	-1.9424473	-9.8364904	9.5987E-06	0.012412242	H3K4me1	LD
chr2	76564936	76564937	chr2_76564501_76566045	-2.4725877	-14.773462	4.3357E-07	0.003169927	H3K4me1	LD
chr2	76565441	76565442	chr2_76564501_76566045	-2.4725877	-14.773462	4.3357E-07	0.003169927	H3K4me1	LD
chr2	76565590	76565591	chr2_76564501_76566045	-2.4725877	-14.773462	4.3357E-07	0.003169927	H3K4me1	LD

chr2	76565605	76565606	chr2_76564501_76566045	-2.4725877	-14.773462	4.3357E-07	0.003169927	H3K4me1	LD
chr2	76565906	76565907	chr2_76564501_76566045	-2.4725877	-14.773462	4.3357E-07	0.003169927	H3K4me1	LD
chr2	2357139	2357140	chr2_2357227_2358369	2.7425828	12.5259924	1.545E-06	0.004034294	H3K4me1	LD
chr2	2357176	2357177	chr2_2357227_2358369	2.7425828	12.5259924	1.545E-06	0.004034294	H3K4me1	LD
chr2	2357226	2357227	chr2_2357227_2358369	2.7425828	12.5259924	1.545E-06	0.004034294	H3K4me1	LD
chr2	2357229	2357230	chr2_2357227_2358369	2.7425828	12.5259924	1.545E-06	0.004034294	H3K4me1	LD
chr2	2357453	2357454	chr2_2357227_2358369	2.7425828	12.5259924	1.545E-06	0.004034294	H3K4me1	LD
chr2	2357471	2357472	chr2_2357227_2358369	2.7425828	12.5259924	1.545E-06	0.004034294	H3K4me1	LD
chr2	2357476	2357477	chr2_2357227_2358369	2.7425828	12.5259924	1.545E-06	0.004034294	H3K4me1	LD
chr2	2357513	2357514	chr2_2357227_2358369	2.7425828	12.5259924	1.545E-06	0.004034294	H3K4me1	LD
chr2	2357756	2357757	chr2_2357227_2358369	2.7425828	12.5259924	1.545E-06	0.004034294	H3K4me1	LD
chr2	88882942	88882943	chr2_88883002_88883493	-5.1366617	-11.406935	3.1516E-06	0.004187054	H3K4me1	LD
chr2	88883079	88883080	chr2_88883002_88883493	-5.1366617	-11.406935	3.1516E-06	0.004187054	H3K4me1	LD

chr2	88883215	88883216	chr2_88883002_88883493	-5.1366617	-11.406935	3.1516E-06	0.004187054	H3K4me1	LD
chr2	88883254	88883255	chr2_88883002_88883493	-5.1366617	-11.406935	3.1516E-06	0.004187054	H3K4me1	LD
chr2	88883270	88883271	chr2_88883002_88883493	-5.1366617	-11.406935	3.1516E-06	0.004187054	H3K4me1	LD
chr2	88883379	88883380	chr2_88883002_88883493	-5.1366617	-11.406935	3.1516E-06	0.004187054	H3K4me1	LD
chr2	88883451	88883452	chr2_88883002_88883493	-5.1366617	-11.406935	3.1516E-06	0.004187054	H3K4me1	LD
chr2	94474163	94474164	chr2_94473978_94475577	-1.5381208	-11.328151	3.3216E-06	0.004187054	H3K4me1	LD
chr2	94474208	94474209	chr2_94473978_94475577	-1.5381208	-11.328151	3.3216E-06	0.004187054	H3K4me1	LD
chr2	94474233	94474234	chr2_94473978_94475577	-1.5381208	-11.328151	3.3216E-06	0.004187054	H3K4me1	LD
chr2	94474370	94474371	chr2_94473978_94475577	-1.5381208	-11.328151	3.3216E-06	0.004187054	H3K4me1	LD
chr2	94474573	94474574	chr2_94473978_94475577	-1.5381208	-11.328151	3.3216E-06	0.004187054	H3K4me1	LD
chr2	94474653	94474654	chr2_94473978_94475577	-1.5381208	-11.328151	3.3216E-06	0.004187054	H3K4me1	LD
chr2	94474770	94474771	chr2_94473978_94475577	-1.5381208	-11.328151	3.3216E-06	0.004187054	H3K4me1	LD
chr2	94474797	94474798	chr2_94473978_94475577	-1.5381208	-11.328151	3.3216E-06	0.004187054	H3K4me1	LD

chr2	26446978	26446979	chr2_26445645_26446927	-3.7466312	-11.203971	3.6108E-06	0.00439983	H3K4me1	LD
chr2	136495026	136495027	chr2_136494942_136495389	-4.7871963	-10.865744	4.5518E-06	0.00504227	H3K4me1	LD
chr2	136495057	136495058	chr2_136494942_136495389	-4.7871963	-10.865744	4.5518E-06	0.00504227	H3K4me1	LD
chr2	136495065	136495066	chr2_136494942_136495389	-4.7871963	-10.865744	4.5518E-06	0.00504227	H3K4me1	LD
chr2	135645701	135645702	chr2_135645133_135647059	-6.2323011	-10.570899	5.5996E-06	0.005848494	H3K4me1	LD
chr2	135645845	135645846	chr2_135645133_135647059	-6.2323011	-10.570899	5.5996E-06	0.005848494	H3K4me1	LD
chr2	32877581	32877582	chr2_32877205_32877910	3.3232642	10.2198849	7.2142E-06	0.007127669	H3K4me1	LD
chr2	32877653	32877654	chr2_32877205_32877910	3.3232642	10.2198849	7.2142E-06	0.007127669	H3K4me1	LD
chr2	134823800	134823801	chr2_134823703_134824757	-2.4947672	-9.8125019	9.7748E-06	0.007940632	H3K4me1	LD
chr2	134823887	134823888	chr2_134823703_134824757	-2.4947672	-9.8125019	9.7748E-06	0.007940632	H3K4me1	LD
chr2	134823896	134823897	chr2_134823703_134824757	-2.4947672	-9.8125019	9.7748E-06	0.007940632	H3K4me1	LD
chr2	134823989	134823990	chr2_134823703_134824757	-2.4947672	-9.8125019	9.7748E-06	0.007940632	H3K4me1	LD
chr2	134824002	134824003	chr2_134823703_134824757	-2.4947672	-9.8125019	9.7748E-06	0.007940632	H3K4me1	LD

chr2	134824127	134824128	chr2_134823703_134824757	-2.4947672	-9.8125019	9.7748E-06	0.007940632	H3K4me1	LD
chr2	134824368	134824369	chr2_134823703_134824757	-2.4947672	-9.8125019	9.7748E-06	0.007940632	H3K4me1	LD
chr2	134824432	134824433	chr2_134823703_134824757	-2.4947672	-9.8125019	9.7748E-06	0.007940632	H3K4me1	LD
chr3	30648312	30648313	chr3_30648322_30649637	-6.932083	-23.88893	1.0043E-08	0.000369052	H3K4me1	LD
chr3	100583744	100583745	chr3_100583782_100584456	-2.2511752	-17.551244	1.1341E-07	0.00208373	H3K4me1	LD
chr3	94305093	94305094	chr3_94304257_94306294	-3.625822	-13.819359	7.2633E-07	0.004812978	H3K4me1	LD
chr3	117758557	117758558	chr3_117758238_117760471	-5.4606508	-12.750253	1.3488E-06	0.004812978	H3K4me1	LD
chr3	1164073	1164074	chr3_1164168_1165613	-2.6841922	-12.640855	1.4408E-06	0.004812978	H3K4me1	LD
chr3	1164109	1164110	chr3_1164168_1165613	-2.6841922	-12.640855	1.4408E-06	0.004812978	H3K4me1	LD
chr3	1164124	1164125	chr3_1164168_1165613	-2.6841922	-12.640855	1.4408E-06	0.004812978	H3K4me1	LD
chr3	1164268	1164269	chr3_1164168_1165613	-2.6841922	-12.640855	1.4408E-06	0.004812978	H3K4me1	LD
chr3	1164329	1164330	chr3_1164168_1165613	-2.6841922	-12.640855	1.4408E-06	0.004812978	H3K4me1	LD
chr3	1164367	1164368	chr3_1164168_1165613	-2.6841922	-12.640855	1.4408E-06	0.004812978	H3K4me1	LD

chr3	1164827	1164828	chr3_1164168_1165613	-2.6841922	-12.640855	1.4408E-06	0.004812978	H3K4me1	LD
chr3	117889820	117889821	chr3_117888846_117890268	-3.0626284	-11.983728	2.1661E-06	0.005416027	H3K4me1	LD
chr3	117890283	117890284	chr3_117888846_117890268	-3.0626284	-11.983728	2.1661E-06	0.005416027	H3K4me1	LD
chr3	109235278	109235279	chr3_109235248_109236939	-2.828344	-11.756715	2.5056E-06	0.005416027	H3K4me1	LD
chr3	109236079	109236080	chr3_109235248_109236939	-2.828344	-11.756715	2.5056E-06	0.005416027	H3K4me1	LD
chr3	109236282	109236283	chr3_109235248_109236939	-2.828344	-11.756715	2.5056E-06	0.005416027	H3K4me1	LD
chr3	109236553	109236554	chr3_109235248_109236939	-2.828344	-11.756715	2.5056E-06	0.005416027	H3K4me1	LD
chr3	121991535	121991536	chr3_121991422_121993307	4.2433218	10.3303545	6.6559E-06	0.006772919	H3K4me1	LD
chr3	121992042	121992043	chr3_121991422_121993307	4.2433218	10.3303545	6.6559E-06	0.006772919	H3K4me1	LD
chr3	121992200	121992201	chr3_121991422_121993307	4.2433218	10.3303545	6.6559E-06	0.006772919	H3K4me1	LD
chr3	121992347	121992348	chr3_121991422_121993307	4.2433218	10.3303545	6.6559E-06	0.006772919	H3K4me1	LD
chr3	121992399	121992400	chr3_121991422_121993307	4.2433218	10.3303545	6.6559E-06	0.006772919	H3K4me1	LD
chr3	121992773	121992774	chr3_121991422_121993307	4.2433218	10.3303545	6.6559E-06	0.006772919	H3K4me1	LD

chr3	121993373	121993374	chr3_121991422_121993307	4.2433218	10.3303545	6.6559E-06	0.006772919	H3K4me1	LD
chr3	69980626	69980627	chr3_69980706_69981722	-2.3382954	-10.271594	6.9466E-06	0.006772919	H3K4me1	LD
chr3	69980959	69980960	chr3_69980706_69981722	-2.3382954	-10.271594	6.9466E-06	0.006772919	H3K4me1	LD
chr3	69981147	69981148	chr3_69980706_69981722	-2.3382954	-10.271594	6.9466E-06	0.006772919	H3K4me1	LD
chr3	69981239	69981240	chr3_69980706_69981722	-2.3382954	-10.271594	6.9466E-06	0.006772919	H3K4me1	LD
chr3	69981335	69981336	chr3_69980706_69981722	-2.3382954	-10.271594	6.9466E-06	0.006772919	H3K4me1	LD
chr3	69981403	69981404	chr3_69980706_69981722	-2.3382954	-10.271594	6.9466E-06	0.006772919	H3K4me1	LD
chr3	69981414	69981415	chr3_69980706_69981722	-2.3382954	-10.271594	6.9466E-06	0.006772919	H3K4me1	LD
chr3	69981442	69981443	chr3_69980706_69981722	-2.3382954	-10.271594	6.9466E-06	0.006772919	H3K4me1	LD
chr3	69981456	69981457	chr3_69980706_69981722	-2.3382954	-10.271594	6.9466E-06	0.006772919	H3K4me1	LD
chr3	69981468	69981469	chr3_69980706_69981722	-2.3382954	-10.271594	6.9466E-06	0.006772919	H3K4me1	LD
chr3	11432646	11432647	chr3_11431947_11432998	3.4914858	10.020493	8.3594E-06	0.006772919	H3K4me1	LD
chr3	11432709	11432710	chr3_11431947_11432998	3.4914858	10.020493	8.3594E-06	0.006772919	H3K4me1	LD

chr3	11433049	11433050	chr3_11431947_11432998	3.4914858	10.020493	8.3594E-06	0.006772919	H3K4me1	LD
chr3	11433073	11433074	chr3_11431947_11432998	3.4914858	10.020493	8.3594E-06	0.006772919	H3K4me1	LD
chr3	2128903	2128904	chr3_2128895_2130946	-2.066292	-9.8641149	9.4002E-06	0.006772919	H3K4me1	LD
chr3	2129214	2129215	chr3_2128895_2130946	-2.066292	-9.8641149	9.4002E-06	0.006772919	H3K4me1	LD
chr3	2129345	2129346	chr3_2128895_2130946	-2.066292	-9.8641149	9.4002E-06	0.006772919	H3K4me1	LD
chr3	2129579	2129580	chr3_2128895_2130946	-2.066292	-9.8641149	9.4002E-06	0.006772919	H3K4me1	LD
chr3	2129585	2129586	chr3_2128895_2130946	-2.066292	-9.8641149	9.4002E-06	0.006772919	H3K4me1	LD
chr3	2129600	2129601	chr3_2128895_2130946	-2.066292	-9.8641149	9.4002E-06	0.006772919	H3K4me1	LD
chr3	2129635	2129636	chr3_2128895_2130946	-2.066292	-9.8641149	9.4002E-06	0.006772919	H3K4me1	LD
chr3	2129821	2129822	chr3_2128895_2130946	-2.066292	-9.8641149	9.4002E-06	0.006772919	H3K4me1	LD
chr3	2129864	2129865	chr3_2128895_2130946	-2.066292	-9.8641149	9.4002E-06	0.006772919	H3K4me1	LD
chr3	2129890	2129891	chr3_2128895_2130946	-2.066292	-9.8641149	9.4002E-06	0.006772919	H3K4me1	LD
chr3	2129906	2129907	chr3_2128895_2130946	-2.066292	-9.8641149	9.4002E-06	0.006772919	H3K4me1	LD

chr3	2130075	2130076	chr3_2128895_2130946	-2.066292	-9.8641149	9.4002E-06	0.006772919	H3K4me1	LD
chr3	2130366	2130367	chr3_2128895_2130946	-2.066292	-9.8641149	9.4002E-06	0.006772919	H3K4me1	LD
chr4	3479035	3479036	chr4_3478357_3479136	-3.7305108	-17.077796	1.4043E-07	0.000374545	H3K4me1	LD
chr4	3479108	3479109	chr4_3478357_3479136	-3.7305108	-17.077796	1.4043E-07	0.000374545	H3K4me1	LD
chr4	38209906	38209907	chr4_38209531_38211112	-1.6440748	-14.566498	4.8361E-07	0.000374545	H3K4me1	LD
chr4	38209911	38209912	chr4_38209531_38211112	-1.6440748	-14.566498	4.8361E-07	0.000374545	H3K4me1	LD
chr4	38210031	38210032	chr4_38209531_38211112	-1.6440748	-14.566498	4.8361E-07	0.000374545	H3K4me1	LD
chr4	38210113	38210114	chr4_38209531_38211112	-1.6440748	-14.566498	4.8361E-07	0.000374545	H3K4me1	LD
chr4	38210160	38210161	chr4_38209531_38211112	-1.6440748	-14.566498	4.8361E-07	0.000374545	H3K4me1	LD
chr4	38210270	38210271	chr4_38209531_38211112	-1.6440748	-14.566498	4.8361E-07	0.000374545	H3K4me1	LD
chr4	38210564	38210565	chr4_38209531_38211112	-1.6440748	-14.566498	4.8361E-07	0.000374545	H3K4me1	LD
chr4	38210714	38210715	chr4_38209531_38211112	-1.6440748	-14.566498	4.8361E-07	0.000374545	H3K4me1	LD
chr4	38210722	38210723	chr4_38209531_38211112	-1.6440748	-14.566498	4.8361E-07	0.000374545	H3K4me1	LD

chr4	38210855	38210856	chr4_38209531_38211112	-1.6440748	-14.566498	4.8361E-07	0.000374545	H3K4me1	LD
chr4	38211199	38211200	chr4_38209531_38211112	-1.6440748	-14.566498	4.8361E-07	0.000374545	H3K4me1	LD
chr4	30770871	30770872	chr4_30770918_30773413	3.1937244	14.4223593	5.2228E-07	0.000374545	H3K4me1	LD
chr4	30771027	30771028	chr4_30770918_30773413	3.1937244	14.4223593	5.2228E-07	0.000374545	H3K4me1	LD
chr4	30771108	30771109	chr4_30770918_30773413	3.1937244	14.4223593	5.2228E-07	0.000374545	H3K4me1	LD
chr4	30771244	30771245	chr4_30770918_30773413	3.1937244	14.4223593	5.2228E-07	0.000374545	H3K4me1	LD
chr4	30771362	30771363	chr4_30770918_30773413	3.1937244	14.4223593	5.2228E-07	0.000374545	H3K4me1	LD
chr4	30771372	30771373	chr4_30770918_30773413	3.1937244	14.4223593	5.2228E-07	0.000374545	H3K4me1	LD
chr4	30771383	30771384	chr4_30770918_30773413	3.1937244	14.4223593	5.2228E-07	0.000374545	H3K4me1	LD
chr4	30771508	30771509	chr4_30770918_30773413	3.1937244	14.4223593	5.2228E-07	0.000374545	H3K4me1	LD
chr4	30771553	30771554	chr4_30770918_30773413	3.1937244	14.4223593	5.2228E-07	0.000374545	H3K4me1	LD
chr4	30771606	30771607	chr4_30770918_30773413	3.1937244	14.4223593	5.2228E-07	0.000374545	H3K4me1	LD
chr4	30771638	30771639	chr4_30770918_30773413	3.1937244	14.4223593	5.2228E-07	0.000374545	H3K4me1	LD

chr4	30771668	30771669	chr4_30770918_30773413	3.1937244	14.4223593	5.2228E-07	0.000374545	H3K4me1	LD
chr4	30771699	30771700	chr4_30770918_30773413	3.1937244	14.4223593	5.2228E-07	0.000374545	H3K4me1	LD
chr4	30771746	30771747	chr4_30770918_30773413	3.1937244	14.4223593	5.2228E-07	0.000374545	H3K4me1	LD
chr4	30772069	30772070	chr4_30770918_30773413	3.1937244	14.4223593	5.2228E-07	0.000374545	H3K4me1	LD
chr4	30772142	30772143	chr4_30770918_30773413	3.1937244	14.4223593	5.2228E-07	0.000374545	H3K4me1	LD
chr4	30772150	30772151	chr4_30770918_30773413	3.1937244	14.4223593	5.2228E-07	0.000374545	H3K4me1	LD
chr4	30772248	30772249	chr4_30770918_30773413	3.1937244	14.4223593	5.2228E-07	0.000374545	H3K4me1	LD
chr4	30772348	30772349	chr4_30770918_30773413	3.1937244	14.4223593	5.2228E-07	0.000374545	H3K4me1	LD
chr4	30772429	30772430	chr4_30770918_30773413	3.1937244	14.4223593	5.2228E-07	0.000374545	H3K4me1	LD
chr4	30772483	30772484	chr4_30770918_30773413	3.1937244	14.4223593	5.2228E-07	0.000374545	H3K4me1	LD
chr4	30772584	30772585	chr4_30770918_30773413	3.1937244	14.4223593	5.2228E-07	0.000374545	H3K4me1	LD
chr4	30772724	30772725	chr4_30770918_30773413	3.1937244	14.4223593	5.2228E-07	0.000374545	H3K4me1	LD
chr4	30772917	30772918	chr4_30770918_30773413	3.1937244	14.4223593	5.2228E-07	0.000374545	H3K4me1	LD

chr4	30773027	30773028	chr4_30770918_30773413	3.1937244	14.4223593	5.2228E-07	0.000374545	H3K4me1	LD
chr4	30773030	30773031	chr4_30770918_30773413	3.1937244	14.4223593	5.2228E-07	0.000374545	H3K4me1	LD
chr4	30773072	30773073	chr4_30770918_30773413	3.1937244	14.4223593	5.2228E-07	0.000374545	H3K4me1	LD
chr4	30773276	30773277	chr4_30770918_30773413	3.1937244	14.4223593	5.2228E-07	0.000374545	H3K4me1	LD
chr4	30773330	30773331	chr4_30770918_30773413	3.1937244	14.4223593	5.2228E-07	0.000374545	H3K4me1	LD
chr4	30773399	30773400	chr4_30770918_30773413	3.1937244	14.4223593	5.2228E-07	0.000374545	H3K4me1	LD
chr4	30773418	30773419	chr4_30770918_30773413	3.1937244	14.4223593	5.2228E-07	0.000374545	H3K4me1	LD
chr4	30773494	30773495	chr4_30770918_30773413	3.1937244	14.4223593	5.2228E-07	0.000374545	H3K4me1	LD
chr4	79985541	79985542	chr4_79985334_79986402	2.4637058	13.9429581	6.7812E-07	0.000437674	H3K4me1	LD
chr4	79985653	79985654	chr4_79985334_79986402	2.4637058	13.9429581	6.7812E-07	0.000437674	H3K4me1	LD
chr4	79985846	79985847	chr4_79985334_79986402	2.4637058	13.9429581	6.7812E-07	0.000437674	H3K4me1	LD
chr4	79985908	79985909	chr4_79985334_79986402	2.4637058	13.9429581	6.7812E-07	0.000437674	H3K4me1	LD
chr4	79986353	79986354	chr4_79985334_79986402	2.4637058	13.9429581	6.7812E-07	0.000437674	H3K4me1	LD

chr4	73104192	73104193	chr4_73104258_73105954	2.036462	11.2101611	3.5957E-06	0.001845882	H3K4me1	LD
chr4	73104268	73104269	chr4_73104258_73105954	2.036462	11.2101611	3.5957E-06	0.001845882	H3K4me1	LD
chr4	73104353	73104354	chr4_73104258_73105954	2.036462	11.2101611	3.5957E-06	0.001845882	H3K4me1	LD
chr4	73104381	73104382	chr4_73104258_73105954	2.036462	11.2101611	3.5957E-06	0.001845882	H3K4me1	LD
chr4	73104426	73104427	chr4_73104258_73105954	2.036462	11.2101611	3.5957E-06	0.001845882	H3K4me1	LD
chr4	73104453	73104454	chr4_73104258_73105954	2.036462	11.2101611	3.5957E-06	0.001845882	H3K4me1	LD
chr4	73104462	73104463	chr4_73104258_73105954	2.036462	11.2101611	3.5957E-06	0.001845882	H3K4me1	LD
chr4	73104470	73104471	chr4_73104258_73105954	2.036462	11.2101611	3.5957E-06	0.001845882	H3K4me1	LD
chr4	73104513	73104514	chr4_73104258_73105954	2.036462	11.2101611	3.5957E-06	0.001845882	H3K4me1	LD
chr4	130503505	130503506	chr4_130503005_130504514	-3.6454455	-11.160733	3.718E-06	0.001845882	H3K4me1	LD
chr4	130503506	130503507	chr4_130503005_130504514	-3.6454455	-11.160733	3.718E-06	0.001845882	H3K4me1	LD
chr4	130503568	130503569	chr4_130503005_130504514	-3.6454455	-11.160733	3.718E-06	0.001845882	H3K4me1	LD
chr4	130503620	130503621	chr4_130503005_130504514	-3.6454455	-11.160733	3.718E-06	0.001845882	H3K4me1	LD

chr4	130503806	130503807	chr4_130503005_130504514	-3.6454455	-11.160733	3.718E-06	0.001845882	H3K4me1	LD
chr4	130504133	130504134	chr4_130503005_130504514	-3.6454455	-11.160733	3.718E-06	0.001845882	H3K4me1	LD
chr4	106679440	106679441	chr4_106679028_106680593	-5.7721016	-10.464485	6.0418E-06	0.002437205	H3K4me1	LD
chr4	106679620	106679621	chr4_106679028_106680593	-5.7721016	-10.464485	6.0418E-06	0.002437205	H3K4me1	LD
chr4	106679645	106679646	chr4_106679028_106680593	-5.7721016	-10.464485	6.0418E-06	0.002437205	H3K4me1	LD
chr4	106679708	106679709	chr4_106679028_106680593	-5.7721016	-10.464485	6.0418E-06	0.002437205	H3K4me1	LD
chr4	106679722	106679723	chr4_106679028_106680593	-5.7721016	-10.464485	6.0418E-06	0.002437205	H3K4me1	LD
chr4	106679881	106679882	chr4_106679028_106680593	-5.7721016	-10.464485	6.0418E-06	0.002437205	H3K4me1	LD
chr4	106679937	106679938	chr4_106679028_106680593	-5.7721016	-10.464485	6.0418E-06	0.002437205	H3K4me1	LD
chr4	106679946	106679947	chr4_106679028_106680593	-5.7721016	-10.464485	6.0418E-06	0.002437205	H3K4me1	LD
chr4	106680053	106680054	chr4_106679028_106680593	-5.7721016	-10.464485	6.0418E-06	0.002437205	H3K4me1	LD
chr4	106680165	106680166	chr4_106679028_106680593	-5.7721016	-10.464485	6.0418E-06	0.002437205	H3K4me1	LD
chr4	106680247	106680248	chr4_106679028_106680593	-5.7721016	-10.464485	6.0418E-06	0.002437205	H3K4me1	LD

chr4	106680260	106680261	chr4_106679028_106680593	-5.7721016	-10.464485	6.0418E-06	0.002437205	H3K4me1	LD
chr4	106680315	106680316	chr4_106679028_106680593	-5.7721016	-10.464485	6.0418E-06	0.002437205	H3K4me1	LD
chr4	106680470	106680471	chr4_106679028_106680593	-5.7721016	-10.464485	6.0418E-06	0.002437205	H3K4me1	LD
chr4	106680506	106680507	chr4_106679028_106680593	-5.7721016	-10.464485	6.0418E-06	0.002437205	H3K4me1	LD
chr5	1347172	1347173	chr5_1346805_1347750	-3.8059926	-20.627964	3.1948E-08	0.000320863	H3K4me1	LD
chr5	1347194	1347195	chr5_1346805_1347750	-3.8059926	-20.627964	3.1948E-08	0.000320863	H3K4me1	LD
chr5	1347377	1347378	chr5_1346805_1347750	-3.8059926	-20.627964	3.1948E-08	0.000320863	H3K4me1	LD
chr5	5045793	5045794	chr5_5045567_5047110	3.385269	15.7426551	2.648E-07	0.001329937	H3K4me1	LD
chr5	22306398	22306399	chr5_22305798_22308196	-7.2662194	-15.433088	3.0898E-07	0.001329937	H3K4me1	LD
chr5	22307139	22307140	chr5_22305798_22308196	-7.2662194	-15.433088	3.0898E-07	0.001329937	H3K4me1	LD
chr5	22307696	22307697	chr5_22305798_22308196	-7.2662194	-15.433088	3.0898E-07	0.001329937	H3K4me1	LD
chr5	91274972	91274973	chr5_91274261_91275687	3.8074165	14.2672151	5.6783E-07	0.001900954	H3K4me1	LD
chr5	91275737	91275738	chr5_91274261_91275687	3.8074165	14.2672151	5.6783E-07	0.001900954	H3K4me1	LD

chr5	72845358	72845359	chr5_72845104_72845529	-2.9555513	-12.830729	1.2853E-06	0.003520441	H3K4me1	LD
chr5	72845382	72845383	chr5_72845104_72845529	-2.9555513	-12.830729	1.2853E-06	0.003520441	H3K4me1	LD
chr5	28658574	28658575	chr5_28658036_28658896	-5.2336036	-11.545993	2.8748E-06	0.006662886	H3K4me1	LD
chr5	28658674	28658675	chr5_28658036_28658896	-5.2336036	-11.545993	2.8748E-06	0.006662886	H3K4me1	LD
chr5	91283870	91283871	chr5_91283512_91284030	1.8846066	10.1414471	7.6424E-06	0.011075897	H3K4me1	LD
chr5	91283885	91283886	chr5_91283512_91284030	1.8846066	10.1414471	7.6424E-06	0.011075897	H3K4me1	LD
chr5	66694891	66694892	chr5_66694616_66694920	-3.8656716	-10.006683	8.4459E-06	0.011075897	H3K4me1	LD
chr5	66695017	66695018	chr5_66694616_66694920	-3.8656716	-10.006683	8.4459E-06	0.011075897	H3K4me1	LD
chr5	17270877	17270878	chr5_17270905_17271883	-3.661364	-9.8940719	9.1901E-06	0.011075897	H3K4me1	LD
chr5	17270888	17270889	chr5_17270905_17271883	-3.661364	-9.8940719	9.1901E-06	0.011075897	H3K4me1	LD
chr5	17271215	17271216	chr5_17270905_17271883	-3.661364	-9.8940719	9.1901E-06	0.011075897	H3K4me1	LD
chr5	17271270	17271271	chr5_17270905_17271883	-3.661364	-9.8940719	9.1901E-06	0.011075897	H3K4me1	LD
chr5	17271379	17271380	chr5_17270905_17271883	-3.661364	-9.8940719	9.1901E-06	0.011075897	H3K4me1	LD

chr5	17271385	17271386	chr5_17270905_17271883	-3.661364	-9.8940719	9.1901E-06	0.011075897	H3K4me1	LD
chr5	17271511	17271512	chr5_17270905_17271883	-3.661364	-9.8940719	9.1901E-06	0.011075897	H3K4me1	LD
chr5	17271658	17271659	chr5_17270905_17271883	-3.661364	-9.8940719	9.1901E-06	0.011075897	H3K4me1	LD
chr6	28932729	28932730	chr6_28932529_28933665	-3.103464	-13.729749	7.6368E-07	0.002422169	H3K4me1	LD
chr6	28933320	28933321	chr6_28932529_28933665	-3.103464	-13.729749	7.6368E-07	0.002422169	H3K4me1	LD
chr6	28933432	28933433	chr6_28932529_28933665	-3.103464	-13.729749	7.6368E-07	0.002422169	H3K4me1	LD
chr6	2271589	2271590	chr6_2271631_2273152	1.9925748	13.3317693	9.5776E-07	0.002422169	H3K4me1	LD
chr6	2271721	2271722	chr6_2271631_2273152	1.9925748	13.3317693	9.5776E-07	0.002422169	H3K4me1	LD
chr6	2272244	2272245	chr6_2271631_2273152	1.9925748	13.3317693	9.5776E-07	0.002422169	H3K4me1	LD
chr6	2272251	2272252	chr6_2271631_2273152	1.9925748	13.3317693	9.5776E-07	0.002422169	H3K4me1	LD
chr6	2272592	2272593	chr6_2271631_2273152	1.9925748	13.3317693	9.5776E-07	0.002422169	H3K4me1	LD
chr6	2272863	2272864	chr6_2271631_2273152	1.9925748	13.3317693	9.5776E-07	0.002422169	H3K4me1	LD
chr6	2273187	2273188	chr6_2271631_2273152	1.9925748	13.3317693	9.5776E-07	0.002422169	H3K4me1	LD

chr6	152870485	152870486	chr6_152869776_152871567	-4.2124048	-13.195579	1.0364E-06	0.002422169	H3K4me1	LD
chr6	152870486	152870487	chr6_152869776_152871567	-4.2124048	-13.195579	1.0364E-06	0.002422169	H3K4me1	LD
chr6	152870690	152870691	chr6_152869776_152871567	-4.2124048	-13.195579	1.0364E-06	0.002422169	H3K4me1	LD
chr6	80083337	80083338	chr6_80082379_80083809	-4.0197267	-12.655422	1.4281E-06	0.002422169	H3K4me1	LD
chr6	80083522	80083523	chr6_80082379_80083809	-4.0197267	-12.655422	1.4281E-06	0.002422169	H3K4me1	LD
chr6	80083601	80083602	chr6_80082379_80083809	-4.0197267	-12.655422	1.4281E-06	0.002422169	H3K4me1	LD
chr6	80083840	80083841	chr6_80082379_80083809	-4.0197267	-12.655422	1.4281E-06	0.002422169	H3K4me1	LD
chr6	42996277	42996278	chr6_42996140_42997149	-4.6283859	-12.439451	1.6292E-06	0.002422169	H3K4me1	LD
chr6	42996474	42996475	chr6_42996140_42997149	-4.6283859	-12.439451	1.6292E-06	0.002422169	H3K4me1	LD
chr6	42997203	42997204	chr6_42996140_42997149	-4.6283859	-12.439451	1.6292E-06	0.002422169	H3K4me1	LD
chr6	161278128	161278129	chr6_161278203_161280636	2.452265	11.7080447	2.5859E-06	0.002422169	H3K4me1	LD
chr6	161278192	161278193	chr6_161278203_161280636	2.452265	11.7080447	2.5859E-06	0.002422169	H3K4me1	LD
chr6	161278237	161278238	chr6_161278203_161280636	2.452265	11.7080447	2.5859E-06	0.002422169	H3K4me1	LD

chr6	161278320	161278321	chr6_161278203_161280636	2.452265	11.7080447	2.5859E-06	0.002422169	H3K4me1	LD
chr6	161278450	161278451	chr6_161278203_161280636	2.452265	11.7080447	2.5859E-06	0.002422169	H3K4me1	LD
chr6	161278494	161278495	chr6_161278203_161280636	2.452265	11.7080447	2.5859E-06	0.002422169	H3K4me1	LD
chr6	161278802	161278803	chr6_161278203_161280636	2.452265	11.7080447	2.5859E-06	0.002422169	H3K4me1	LD
chr6	161278835	161278836	chr6_161278203_161280636	2.452265	11.7080447	2.5859E-06	0.002422169	H3K4me1	LD
chr6	161278854	161278855	chr6_161278203_161280636	2.452265	11.7080447	2.5859E-06	0.002422169	H3K4me1	LD
chr6	161278963	161278964	chr6_161278203_161280636	2.452265	11.7080447	2.5859E-06	0.002422169	H3K4me1	LD
chr6	161279153	161279154	chr6_161278203_161280636	2.452265	11.7080447	2.5859E-06	0.002422169	H3K4me1	LD
chr6	161279287	161279288	chr6_161278203_161280636	2.452265	11.7080447	2.5859E-06	0.002422169	H3K4me1	LD
chr6	161279467	161279468	chr6_161278203_161280636	2.452265	11.7080447	2.5859E-06	0.002422169	H3K4me1	LD
chr6	161280006	161280007	chr6_161278203_161280636	2.452265	11.7080447	2.5859E-06	0.002422169	H3K4me1	LD
chr6	161280194	161280195	chr6_161278203_161280636	2.452265	11.7080447	2.5859E-06	0.002422169	H3K4me1	LD
chr6	161280240	161280241	chr6_161278203_161280636	2.452265	11.7080447	2.5859E-06	0.002422169	H3K4me1	LD

chr6	161280389	161280390	chr6_161278203_161280636	2.452265	11.7080447	2.5859E-06	0.002422169	H3K4me1	LD
chr6	161280596	161280597	chr6_161278203_161280636	2.452265	11.7080447	2.5859E-06	0.002422169	H3K4me1	LD
chr6	161280638	161280639	chr6_161278203_161280636	2.452265	11.7080447	2.5859E-06	0.002422169	H3K4me1	LD
chr6	161280687	161280688	chr6_161278203_161280636	2.452265	11.7080447	2.5859E-06	0.002422169	H3K4me1	LD
chr6	161279553	161279554	chr6_161278203_161280636	2.452265	11.7080447	2.5859E-06	0.002422169	H3K4me1	LD
chr6	161279563	161279564	chr6_161278203_161280636	2.452265	11.7080447	2.5859E-06	0.002422169	H3K4me1	LD
chr6	65062184	65062185	chr6_65062062_65063265	-5.5657181	-11.344691	3.2851E-06	0.002481694	H3K4me1	LD
chr6	65062455	65062456	chr6_65062062_65063265	-5.5657181	-11.344691	3.2851E-06	0.002481694	H3K4me1	LD
chr6	65062539	65062540	chr6_65062062_65063265	-5.5657181	-11.344691	3.2851E-06	0.002481694	H3K4me1	LD
chr6	65062718	65062719	chr6_65062062_65063265	-5.5657181	-11.344691	3.2851E-06	0.002481694	H3K4me1	LD
chr6	65062885	65062886	chr6_65062062_65063265	-5.5657181	-11.344691	3.2851E-06	0.002481694	H3K4me1	LD
chr6	162788768	162788769	chr6_162788854_162790248	-1.5908958	-11.28001	3.4305E-06	0.002481694	H3K4me1	LD
chr6	162788774	162788775	chr6_162788854_162790248	-1.5908958	-11.28001	3.4305E-06	0.002481694	H3K4me1	LD

chr6	5188588	5188589	chr6_5188676_5189199	-3.3622518	-11.263157	3.4696E-06	0.002481694	H3K4me1	LD
chr6	5188634	5188635	chr6_5188676_5189199	-3.3622518	-11.263157	3.4696E-06	0.002481694	H3K4me1	LD
chr6	5188641	5188642	chr6_5188676_5189199	-3.3622518	-11.263157	3.4696E-06	0.002481694	H3K4me1	LD
chr6	5189027	5189028	chr6_5188676_5189199	-3.3622518	-11.263157	3.4696E-06	0.002481694	H3K4me1	LD
chr6	5189094	5189095	chr6_5188676_5189199	-3.3622518	-11.263157	3.4696E-06	0.002481694	H3K4me1	LD
chr6	5189150	5189151	chr6_5188676_5189199	-3.3622518	-11.263157	3.4696E-06	0.002481694	H3K4me1	LD
chr6	128226465	128226466	chr6_128226028_128227104	-2.4988767	-10.831065	4.6628E-06	0.002879199	H3K4me1	LD
chr6	13557174	13557175	chr6_13557271_13558305	-3.131265	-10.802322	4.7572E-06	0.002879199	H3K4me1	LD
chr6	13557212	13557213	chr6_13557271_13558305	-3.131265	-10.802322	4.7572E-06	0.002879199	H3K4me1	LD
chr6	13557634	13557635	chr6_13557271_13558305	-3.131265	-10.802322	4.7572E-06	0.002879199	H3K4me1	LD
chr6	13557658	13557659	chr6_13557271_13558305	-3.131265	-10.802322	4.7572E-06	0.002879199	H3K4me1	LD
chr6	13557736	13557737	chr6_13557271_13558305	-3.131265	-10.802322	4.7572E-06	0.002879199	H3K4me1	LD
chr6	13557740	13557741	chr6_13557271_13558305	-3.131265	-10.802322	4.7572E-06	0.002879199	H3K4me1	LD

chr6	13557868	13557869	chr6_13557271_13558305	-3.131265	-10.802322	4.7572E-06	0.002879199	H3K4me1	LD
chr6	13557996	13557997	chr6_13557271_13558305	-3.131265	-10.802322	4.7572E-06	0.002879199	H3K4me1	LD
chr6	13558256	13558257	chr6_13557271_13558305	-3.131265	-10.802322	4.7572E-06	0.002879199	H3K4me1	LD
chr6	167989603	167989604	chr6_167989652_167989984	5.4988202	10.7293254	5.0065E-06	0.002982456	H3K4me1	LD
chr6	36275816	36275817	chr6_36275341_36276560	-2.5466248	-10.687703	5.1552E-06	0.002982456	H3K4me1	LD
chr6	36276058	36276059	chr6_36275341_36276560	-2.5466248	-10.687703	5.1552E-06	0.002982456	H3K4me1	LD
chr6	120040756	120040757	chr6_120040630_120042443	3.6921825	10.3625255	6.5025E-06	0.003602944	H3K4me1	LD
chr6	120041470	120041471	chr6_120040630_120042443	3.6921825	10.3625255	6.5025E-06	0.003602944	H3K4me1	LD
chr6	120041472	120041473	chr6_120040630_120042443	3.6921825	10.3625255	6.5025E-06	0.003602944	H3K4me1	LD
chr6	51742638	51742639	chr6_51742250_51744281	-4.3627608	-9.9014046	9.1395E-06	0.004551224	H3K4me1	LD
chr6	51742927	51742928	chr6_51742250_51744281	-4.3627608	-9.9014046	9.1395E-06	0.004551224	H3K4me1	LD
chr6	51743825	51743826	chr6_51742250_51744281	-4.3627608	-9.9014046	9.1395E-06	0.004551224	H3K4me1	LD
chr6	51743900	51743901	chr6_51742250_51744281	-4.3627608	-9.9014046	9.1395E-06	0.004551224	H3K4me1	LD

chr6	51744162	51744163	chr6_51742250_51744281	-4.3627608	-9.9014046	9.1395E-06	0.004551224	H3K4me1	LD
chr7	58712573	58712574	chr7_58711124_58713732	6.3412574	15.4428007	3.0747E-07	0.000794881	H3K4me1	LD
chr7	58712918	58712919	chr7_58711124_58713732	6.3412574	15.4428007	3.0747E-07	0.000794881	H3K4me1	LD
chr7	102560043	102560044	chr7_102559980_102561602	-4.8379578	-14.60072	4.7491E-07	0.000794881	H3K4me1	LD
chr7	102560105	102560106	chr7_102559980_102561602	-4.8379578	-14.60072	4.7491E-07	0.000794881	H3K4me1	LD
chr7	102560229	102560230	chr7_102559980_102561602	-4.8379578	-14.60072	4.7491E-07	0.000794881	H3K4me1	LD
chr7	102560368	102560369	chr7_102559980_102561602	-4.8379578	-14.60072	4.7491E-07	0.000794881	H3K4me1	LD
chr7	102560454	102560455	chr7_102559980_102561602	-4.8379578	-14.60072	4.7491E-07	0.000794881	H3K4me1	LD
chr7	102560515	102560516	chr7_102559980_102561602	-4.8379578	-14.60072	4.7491E-07	0.000794881	H3K4me1	LD
chr7	102560602	102560603	chr7_102559980_102561602	-4.8379578	-14.60072	4.7491E-07	0.000794881	H3K4me1	LD
chr7	102560619	102560620	chr7_102559980_102561602	-4.8379578	-14.60072	4.7491E-07	0.000794881	H3K4me1	LD
chr7	102560648	102560649	chr7_102559980_102561602	-4.8379578	-14.60072	4.7491E-07	0.000794881	H3K4me1	LD
chr7	102560894	102560895	chr7_102559980_102561602	-4.8379578	-14.60072	4.7491E-07	0.000794881	H3K4me1	LD

chr7	102560935	102560936	chr7_102559980_102561602	-4.8379578	-14.60072	4.7491E-07	0.000794881	H3K4me1	LD
chr7	102561098	102561099	chr7_102559980_102561602	-4.8379578	-14.60072	4.7491E-07	0.000794881	H3K4me1	LD
chr7	102561131	102561132	chr7_102559980_102561602	-4.8379578	-14.60072	4.7491E-07	0.000794881	H3K4me1	LD
chr7	102561144	102561145	chr7_102559980_102561602	-4.8379578	-14.60072	4.7491E-07	0.000794881	H3K4me1	LD
chr7	102561285	102561286	chr7_102559980_102561602	-4.8379578	-14.60072	4.7491E-07	0.000794881	H3K4me1	LD
chr7	102561380	102561381	chr7_102559980_102561602	-4.8379578	-14.60072	4.7491E-07	0.000794881	H3K4me1	LD
chr7	102561548	102561549	chr7_102559980_102561602	-4.8379578	-14.60072	4.7491E-07	0.000794881	H3K4me1	LD
chr7	102561585	102561586	chr7_102559980_102561602	-4.8379578	-14.60072	4.7491E-07	0.000794881	H3K4me1	LD
chr7	102561667	102561668	chr7_102559980_102561602	-4.8379578	-14.60072	4.7491E-07	0.000794881	H3K4me1	LD
chr7	64530562	64530563	chr7_64530515_64530934	4.449199	13.3603565	9.4211E-07	0.00132457	H3K4me1	LD
chr7	64530691	64530692	chr7_64530515_64530934	4.449199	13.3603565	9.4211E-07	0.00132457	H3K4me1	LD
chr7	64530713	64530714	chr7_64530515_64530934	4.449199	13.3603565	9.4211E-07	0.00132457	H3K4me1	LD
chr7	64530886	64530887	chr7_64530515_64530934	4.449199	13.3603565	9.4211E-07	0.00132457	H3K4me1	LD

chr7	54234785	54234786	chr7_54234844_54235897	-2.3723732	-12.343587	1.7284E-06	0.00181715	H3K4me1	LD
chr7	54235149	54235150	chr7_54234844_54235897	-2.3723732	-12.343587	1.7284E-06	0.00181715	H3K4me1	LD
chr7	54235496	54235497	chr7_54234844_54235897	-2.3723732	-12.343587	1.7284E-06	0.00181715	H3K4me1	LD
chr7	54235803	54235804	chr7_54234844_54235897	-2.3723732	-12.343587	1.7284E-06	0.00181715	H3K4me1	LD
chr7	54235861	54235862	chr7_54234844_54235897	-2.3723732	-12.343587	1.7284E-06	0.00181715	H3K4me1	LD
chr7	97813136	97813137	chr7_97813068_97813953	-7.6470658	-12.068818	2.0524E-06	0.00181715	H3K4me1	LD
chr7	97813332	97813333	chr7_97813068_97813953	-7.6470658	-12.068818	2.0524E-06	0.00181715	H3K4me1	LD
chr7	97813724	97813725	chr7_97813068_97813953	-7.6470658	-12.068818	2.0524E-06	0.00181715	H3K4me1	LD
chr7	97813737	97813738	chr7_97813068_97813953	-7.6470658	-12.068818	2.0524E-06	0.00181715	H3K4me1	LD
chr7	97813764	97813765	chr7_97813068_97813953	-7.6470658	-12.068818	2.0524E-06	0.00181715	H3K4me1	LD
chr7	97813777	97813778	chr7_97813068_97813953	-7.6470658	-12.068818	2.0524E-06	0.00181715	H3K4me1	LD
chr7	97813809	97813810	chr7_97813068_97813953	-7.6470658	-12.068818	2.0524E-06	0.00181715	H3K4me1	LD
chr7	97813820	97813821	chr7_97813068_97813953	-7.6470658	-12.068818	2.0524E-06	0.00181715	H3K4me1	LD

chr7	97813829	97813830	chr7_97813068_97813953	-7.6470658	-12.068818	2.0524E-06	0.00181715	H3K4me1	LD
chr7	102551873	102551874	chr7_102551149_102552237	-2.7612692	-11.979967	2.1713E-06	0.00181715	H3K4me1	LD
chr7	102551980	102551981	chr7_102551149_102552237	-2.7612692	-11.979967	2.1713E-06	0.00181715	H3K4me1	LD
chr7	102552105	102552106	chr7_102551149_102552237	-2.7612692	-11.979967	2.1713E-06	0.00181715	H3K4me1	LD
chr7	76838380	76838381	chr7_76838014_76838841	-2.488535	-11.702632	2.5951E-06	0.002121245	H3K4me1	LD
chr7	86955963	86955964	chr7_86955762_86956634	4.8668012	11.1830473	3.6622E-06	0.002563152	H3K4me1	LD
chr7	86955989	86955990	chr7_86955762_86956634	4.8668012	11.1830473	3.6622E-06	0.002563152	H3K4me1	LD
chr7	86956056	86956057	chr7_86955762_86956634	4.8668012	11.1830473	3.6622E-06	0.002563152	H3K4me1	LD
chr7	86956246	86956247	chr7_86955762_86956634	4.8668012	11.1830473	3.6622E-06	0.002563152	H3K4me1	LD
chr7	15392516	15392517	chr7_15392063_15392726	-3.4526292	-10.999686	4.1497E-06	0.002563152	H3K4me1	LD
chr7	15392638	15392639	chr7_15392063_15392726	-3.4526292	-10.999686	4.1497E-06	0.002563152	H3K4me1	LD
chr7	15392687	15392688	chr7_15392063_15392726	-3.4526292	-10.999686	4.1497E-06	0.002563152	H3K4me1	LD
NW_018084979.1	1240219	1240220	NW_018084979.1_1240170_1240628	6.2751256	10.8524754	4.5939E-06	0.002563152	H3K4me1	LD

chr7	80334717	80334718	chr7_80334683_80337389	-2.2596692	-10.743004	4.9587E-06	0.002563152	H3K4me1	LD
chr7	80334765	80334766	chr7_80334683_80337389	-2.2596692	-10.743004	4.9587E-06	0.002563152	H3K4me1	LD
chr7	80334894	80334895	chr7_80334683_80337389	-2.2596692	-10.743004	4.9587E-06	0.002563152	H3K4me1	LD
chr7	80334972	80334973	chr7_80334683_80337389	-2.2596692	-10.743004	4.9587E-06	0.002563152	H3K4me1	LD
chr7	80335400	80335401	chr7_80334683_80337389	-2.2596692	-10.743004	4.9587E-06	0.002563152	H3K4me1	LD
chr7	80335528	80335529	chr7_80334683_80337389	-2.2596692	-10.743004	4.9587E-06	0.002563152	H3K4me1	LD
chr7	80335633	80335634	chr7_80334683_80337389	-2.2596692	-10.743004	4.9587E-06	0.002563152	H3K4me1	LD
chr7	80335886	80335887	chr7_80334683_80337389	-2.2596692	-10.743004	4.9587E-06	0.002563152	H3K4me1	LD
chr7	80335905	80335906	chr7_80334683_80337389	-2.2596692	-10.743004	4.9587E-06	0.002563152	H3K4me1	LD
chr7	80336005	80336006	chr7_80334683_80337389	-2.2596692	-10.743004	4.9587E-06	0.002563152	H3K4me1	LD
chr7	80336114	80336115	chr7_80334683_80337389	-2.2596692	-10.743004	4.9587E-06	0.002563152	H3K4me1	LD
chr7	80336191	80336192	chr7_80334683_80337389	-2.2596692	-10.743004	4.9587E-06	0.002563152	H3K4me1	LD
chr7	80336388	80336389	chr7_80334683_80337389	-2.2596692	-10.743004	4.9587E-06	0.002563152	H3K4me1	LD

chr7	80336510	80336511	chr7_80334683_80337389	-2.2596692	-10.743004	4.9587E-06	0.002563152	H3K4me1	LD
chr7	80336549	80336550	chr7_80334683_80337389	-2.2596692	-10.743004	4.9587E-06	0.002563152	H3K4me1	LD
chr7	80336703	80336704	chr7_80334683_80337389	-2.2596692	-10.743004	4.9587E-06	0.002563152	H3K4me1	LD
chr7	80337070	80337071	chr7_80334683_80337389	-2.2596692	-10.743004	4.9587E-06	0.002563152	H3K4me1	LD
chr7	60050743	60050744	chr7_60050127_60052708	-4.4428762	-10.293798	6.8351E-06	0.003336787	H3K4me1	LD
chr7	60051513	60051514	chr7_60050127_60052708	-4.4428762	-10.293798	6.8351E-06	0.003336787	H3K4me1	LD
chr7	60051670	60051671	chr7_60050127_60052708	-4.4428762	-10.293798	6.8351E-06	0.003336787	H3K4me1	LD
chr7	60052671	60052672	chr7_60050127_60052708	-4.4428762	-10.293798	6.8351E-06	0.003336787	H3K4me1	LD
chr7	80239432	80239433	chr7_80239174_80239495	-4.8721589	-9.9935173	8.5294E-06	0.004106836	H3K4me1	LD
chr8	32068833	32068834	chr8_32068784_32070081	-3.6289486	-17.281189	1.2802E-07	0.000175232	H3K4me1	LD
chr8	32068865	32068866	chr8_32068784_32070081	-3.6289486	-17.281189	1.2802E-07	0.000175232	H3K4me1	LD
chr8	32069128	32069129	chr8_32068784_32070081	-3.6289486	-17.281189	1.2802E-07	0.000175232	H3K4me1	LD
chr8	32069145	32069146	chr8_32068784_32070081	-3.6289486	-17.281189	1.2802E-07	0.000175232	H3K4me1	LD

chr8	32069166	32069167	chr8_32068784_32070081	-3.6289486	-17.281189	1.2802E-07	0.000175232	H3K4me1	LD
chr8	32069199	32069200	chr8_32068784_32070081	-3.6289486	-17.281189	1.2802E-07	0.000175232	H3K4me1	LD
chr8	32069208	32069209	chr8_32068784_32070081	-3.6289486	-17.281189	1.2802E-07	0.000175232	H3K4me1	LD
chr8	32069246	32069247	chr8_32068784_32070081	-3.6289486	-17.281189	1.2802E-07	0.000175232	H3K4me1	LD
chr8	32069252	32069253	chr8_32068784_32070081	-3.6289486	-17.281189	1.2802E-07	0.000175232	H3K4me1	LD
chr8	32069271	32069272	chr8_32068784_32070081	-3.6289486	-17.281189	1.2802E-07	0.000175232	H3K4me1	LD
chr8	32069322	32069323	chr8_32068784_32070081	-3.6289486	-17.281189	1.2802E-07	0.000175232	H3K4me1	LD
chr8	32069344	32069345	chr8_32068784_32070081	-3.6289486	-17.281189	1.2802E-07	0.000175232	H3K4me1	LD
chr8	32069389	32069390	chr8_32068784_32070081	-3.6289486	-17.281189	1.2802E-07	0.000175232	H3K4me1	LD
chr8	32069789	32069790	chr8_32068784_32070081	-3.6289486	-17.281189	1.2802E-07	0.000175232	H3K4me1	LD
chr8	32069866	32069867	chr8_32068784_32070081	-3.6289486	-17.281189	1.2802E-07	0.000175232	H3K4me1	LD
chr8	32069895	32069896	chr8_32068784_32070081	-3.6289486	-17.281189	1.2802E-07	0.000175232	H3K4me1	LD
chr8	32069942	32069943	chr8_32068784_32070081	-3.6289486	-17.281189	1.2802E-07	0.000175232	H3K4me1	LD

chr8	32069982	32069983	chr8_32068784_32070081	-3.6289486	-17.281189	1.2802E-07	0.000175232	H3K4me1	LD
chr8	32069998	32069999	chr8_32068784_32070081	-3.6289486	-17.281189	1.2802E-07	0.000175232	H3K4me1	LD
chr8	32070131	32070132	chr8_32068784_32070081	-3.6289486	-17.281189	1.2802E-07	0.000175232	H3K4me1	LD
chr8	31992454	31992455	chr8_31992415_31992754	-3.1714575	-15.843422	2.5198E-07	0.000299908	H3K4me1	LD
chr8	31992632	31992633	chr8_31992415_31992754	-3.1714575	-15.843422	2.5198E-07	0.000299908	H3K4me1	LD
chr8	31992687	31992688	chr8_31992415_31992754	-3.1714575	-15.843422	2.5198E-07	0.000299908	H3K4me1	LD
chr8	11005973	11005974	chr8_11005138_11006544	1.869511	15.2175316	3.4463E-07	0.00039309	H3K4me1	LD
chr8	19079166	19079167	chr8_19078306_19079581	-1.9011483	-10.986412	4.1878E-06	0.003422888	H3K4me1	LD
chr8	19079358	19079359	chr8_19078306_19079581	-1.9011483	-10.986412	4.1878E-06	0.003422888	H3K4me1	LD
chr8	19079415	19079416	chr8_19078306_19079581	-1.9011483	-10.986412	4.1878E-06	0.003422888	H3K4me1	LD
chr8	32002900	32002901	chr8_32002868_32003686	-1.9737463	-10.922513	4.3763E-06	0.003422888	H3K4me1	LD
chr8	32002901	32002902	chr8_32002868_32003686	-1.9737463	-10.922513	4.3763E-06	0.003422888	H3K4me1	LD
chr8	32003049	32003050	chr8_32002868_32003686	-1.9737463	-10.922513	4.3763E-06	0.003422888	H3K4me1	LD

chr8	32003055	32003056	chr8_32002868_32003686	-1.9737463	-10.922513	4.3763E-06	0.003422888	H3K4me1	LD
chr8	32003513	32003514	chr8_32002868_32003686	-1.9737463	-10.922513	4.3763E-06	0.003422888	H3K4me1	LD
chr8	32003713	32003714	chr8_32002868_32003686	-1.9737463	-10.922513	4.3763E-06	0.003422888	H3K4me1	LD
chr8	32003780	32003781	chr8_32002868_32003686	-1.9737463	-10.922513	4.3763E-06	0.003422888	H3K4me1	LD
chr8	32003784	32003785	chr8_32002868_32003686	-1.9737463	-10.922513	4.3763E-06	0.003422888	H3K4me1	LD
chr8	17554343	17554344	chr8_17553552_17555509	-3.7085342	-10.121492	7.7558E-06	0.005587255	H3K4me1	LD
chr8	17554781	17554782	chr8_17553552_17555509	-3.7085342	-10.121492	7.7558E-06	0.005587255	H3K4me1	LD
chr8	17555456	17555457	chr8_17553552_17555509	-3.7085342	-10.121492	7.7558E-06	0.005587255	H3K4me1	LD
chr8	15615638	15615639	chr8_15615624_15615984	-3.5877836	-9.9193877	9.0166E-06	0.00560978	H3K4me1	LD
chr8	15615653	15615654	chr8_15615624_15615984	-3.5877836	-9.9193877	9.0166E-06	0.00560978	H3K4me1	LD
chr8	15615715	15615716	chr8_15615624_15615984	-3.5877836	-9.9193877	9.0166E-06	0.00560978	H3K4me1	LD
chr8	15615760	15615761	chr8_15615624_15615984	-3.5877836	-9.9193877	9.0166E-06	0.00560978	H3K4me1	LD
chr8	15615932	15615933	chr8_15615624_15615984	-3.5877836	-9.9193877	9.0166E-06	0.00560978	H3K4me1	LD

chr8	15615985	15615986	chr8_15615624_15615984	-3.5877836	-9.9193877	9.0166E-06	0.00560978	H3K4me1	LD
chr9	8363209	8363210	chr9_8363263_8363974	-6.1051493	-18.829393	6.5409E-08	0.000732607	H3K4me1	LD
chr9	8363475	8363476	chr9_8363263_8363974	-6.1051493	-18.829393	6.5409E-08	0.000732607	H3K4me1	LD
chr9	134457155	134457156	chr9_134457217_134458604	-4.0424544	-14.918024	4.0206E-07	0.000732607	H3K4me1	LD
chr9	134457299	134457300	chr9_134457217_134458604	-4.0424544	-14.918024	4.0206E-07	0.000732607	H3K4me1	LD
chr9	134457312	134457313	chr9_134457217_134458604	-4.0424544	-14.918024	4.0206E-07	0.000732607	H3K4me1	LD
chr9	134457321	134457322	chr9_134457217_134458604	-4.0424544	-14.918024	4.0206E-07	0.000732607	H3K4me1	LD
chr9	134457389	134457390	chr9_134457217_134458604	-4.0424544	-14.918024	4.0206E-07	0.000732607	H3K4me1	LD
chr9	134457390	134457391	chr9_134457217_134458604	-4.0424544	-14.918024	4.0206E-07	0.000732607	H3K4me1	LD
chr9	134457409	134457410	chr9_134457217_134458604	-4.0424544	-14.918024	4.0206E-07	0.000732607	H3K4me1	LD
chr9	134457426	134457427	chr9_134457217_134458604	-4.0424544	-14.918024	4.0206E-07	0.000732607	H3K4me1	LD
chr9	134457431	134457432	chr9_134457217_134458604	-4.0424544	-14.918024	4.0206E-07	0.000732607	H3K4me1	LD
chr9	134457445	134457446	chr9_134457217_134458604	-4.0424544	-14.918024	4.0206E-07	0.000732607	H3K4me1	LD

chr9	134457683	134457684	chr9_134457217_134458604	-4.0424544	-14.918024	4.0206E-07	0.000732607	H3K4me1	LD
chr9	134457769	134457770	chr9_134457217_134458604	-4.0424544	-14.918024	4.0206E-07	0.000732607	H3K4me1	LD
chr9	119707376	119707377	chr9_119707403_119709150	-3.2263025	-13.793277	7.3699E-07	0.000732607	H3K4me1	LD
chr9	119707422	119707423	chr9_119707403_119709150	-3.2263025	-13.793277	7.3699E-07	0.000732607	H3K4me1	LD
chr9	119707456	119707457	chr9_119707403_119709150	-3.2263025	-13.793277	7.3699E-07	0.000732607	H3K4me1	LD
chr9	119707524	119707525	chr9_119707403_119709150	-3.2263025	-13.793277	7.3699E-07	0.000732607	H3K4me1	LD
chr9	119707617	119707618	chr9_119707403_119709150	-3.2263025	-13.793277	7.3699E-07	0.000732607	H3K4me1	LD
chr9	119708067	119708068	chr9_119707403_119709150	-3.2263025	-13.793277	7.3699E-07	0.000732607	H3K4me1	LD
chr9	119708387	119708388	chr9_119707403_119709150	-3.2263025	-13.793277	7.3699E-07	0.000732607	H3K4me1	LD
chr9	119708615	119708616	chr9_119707403_119709150	-3.2263025	-13.793277	7.3699E-07	0.000732607	H3K4me1	LD
chr9	119708635	119708636	chr9_119707403_119709150	-3.2263025	-13.793277	7.3699E-07	0.000732607	H3K4me1	LD
chr9	119708701	119708702	chr9_119707403_119709150	-3.2263025	-13.793277	7.3699E-07	0.000732607	H3K4me1	LD
chr9	119708776	119708777	chr9_119707403_119709150	-3.2263025	-13.793277	7.3699E-07	0.000732607	H3K4me1	LD

chr9	119708834	119708835	chr9_119707403_119709150	-3.2263025	-13.793277	7.3699E-07	0.000732607	H3K4me1	LD
chr9	119708853	119708854	chr9_119707403_119709150	-3.2263025	-13.793277	7.3699E-07	0.000732607	H3K4me1	LD
chr9	119708946	119708947	chr9_119707403_119709150	-3.2263025	-13.793277	7.3699E-07	0.000732607	H3K4me1	LD
chr9	35614949	35614950	chr9_35614978_35615685	-4.4535742	-13.791689	7.3764E-07	0.000732607	H3K4me1	LD
chr9	35615363	35615364	chr9_35614978_35615685	-4.4535742	-13.791689	7.3764E-07	0.000732607	H3K4me1	LD
chr9	35615389	35615390	chr9_35614978_35615685	-4.4535742	-13.791689	7.3764E-07	0.000732607	H3K4me1	LD
chr9	35615412	35615413	chr9_35614978_35615685	-4.4535742	-13.791689	7.3764E-07	0.000732607	H3K4me1	LD
chr9	35615437	35615438	chr9_35614978_35615685	-4.4535742	-13.791689	7.3764E-07	0.000732607	H3K4me1	LD
chr9	35615579	35615580	chr9_35614978_35615685	-4.4535742	-13.791689	7.3764E-07	0.000732607	H3K4me1	LD
chr9	47680271	47680272	chr9_47679905_47680615	3.7637458	11.8092611	2.4221E-06	0.00233681	H3K4me1	LD
chr9	13197358	13197359	chr9_13197005_13197416	-2.0478933	-11.51383	2.9363E-06	0.002754265	H3K4me1	LD
chr9	9493150	9493151	chr9_9493220_9493751	3.7355664	10.9395014	4.3253E-06	0.00331944	H3K4me1	LD
chr9	9493178	9493179	chr9_9493220_9493751	3.7355664	10.9395014	4.3253E-06	0.00331944	H3K4me1	LD

chr9	9493265	9493266	chr9_9493220_9493751	3.7355664	10.9395014	4.3253E-06	0.00331944	H3K4me1	LD
chr9	9493297	9493298	chr9_9493220_9493751	3.7355664	10.9395014	4.3253E-06	0.00331944	H3K4me1	LD
chr9	9493391	9493392	chr9_9493220_9493751	3.7355664	10.9395014	4.3253E-06	0.00331944	H3K4me1	LD
chr9	9493406	9493407	chr9_9493220_9493751	3.7355664	10.9395014	4.3253E-06	0.00331944	H3K4me1	LD
chr9	9493506	9493507	chr9_9493220_9493751	3.7355664	10.9395014	4.3253E-06	0.00331944	H3K4me1	LD
chr9	9493610	9493611	chr9_9493220_9493751	3.7355664	10.9395014	4.3253E-06	0.00331944	H3K4me1	LD
chr9	9243685	9243686	chr9_9243684_9244061	4.9553033	10.7321985	4.9965E-06	0.003589794	H3K4me1	LD
chr9	9243804	9243805	chr9_9243684_9244061	4.9553033	10.7321985	4.9965E-06	0.003589794	H3K4me1	LD
chr9	9243969	9243970	chr9_9243684_9244061	4.9553033	10.7321985	4.9965E-06	0.003589794	H3K4me1	LD
chr9	47960633	47960634	chr9_47960087_47961294	2.8412461	9.83554407	9.6055E-06	0.006757501	H3K4me1	LD
chr11	67947411	67947412	chr11_67947319_67949849	7.629414	15.643759	2.7809E-07	0.000284041	H3K4me3	LD
chr11	67948319	67948320	chr11_67947319_67949849	7.629414	15.643759	2.7809E-07	0.000284041	H3K4me3	LD
chr11	67948738	67948739	chr11_67947319_67949849	7.629414	15.643759	2.7809E-07	0.000284041	H3K4me3	LD

chr11	67948921	67948922	chr11_67947319_67949849	-7.629414	-15.643759	2.7809E-07	0.000284041	H3K4me3	LD
chr11	67949725	67949726	chr11_67947319_67949849	7.629414	15.643759	2.7809E-07	0.000284041	H3K4me3	LD
chr11	12309342	12309343	chr11_12309128_12310276	9.6089057	11.0949306	3.888E-06	0.001654669	H3K4me3	LD
chr11	12309529	12309530	chr11_12309128_12310276	9.6089057	11.0949306	3.888E-06	0.001654669	H3K4me3	LD
chr11	12309598	12309599	chr11_12309128_12310276	9.6089057	11.0949306	3.888E-06	0.001654669	H3K4me3	LD
chr11	12309718	12309719	chr11_12309128_12310276	9.6089057	11.0949306	3.888E-06	0.001654669	H3K4me3	LD
chr11	12310211	12310212	chr11_12309128_12310276	9.6089057	11.0949306	3.888E-06	0.001654669	H3K4me3	LD
chr11	12310272	12310273	chr11_12309128_12310276	9.6089057	11.0949306	3.888E-06	0.001654669	H3K4me3	LD
chr11	12310323	12310324	chr11_12309128_12310276	9.6089057	11.0949306	3.888E-06	0.001654669	H3K4me3	LD
chr12	58938185	58938186	chr12_58938077_58940051	22.962358	11.6455424	2.6933E-06	0.004273991	H3K4me3	LD
chr12	58939876	58939877	chr12_58938077_58940051	22.962358	11.6455424	2.6933E-06	0.004273991	H3K4me3	LD
chr12	25409851	25409852	chr12_25409212_25410900	-8.1522176	-10.689255	5.1496E-06	0.004273991	H3K4me3	LD
chr12	25409861	25409862	chr12_25409212_25410900	-8.1522176	-10.689255	5.1496E-06	0.004273991	H3K4me3	LD

chr12	25409890	25409891	chr12_25409212_25410900	-8.1522176	-10.689255	5.1496E-06	0.004273991	H3K4me3	LD
chr12	25410061	25410062	chr12_25409212_25410900	-8.1522176	-10.689255	5.1496E-06	0.004273991	H3K4me3	LD
chr12	25410211	25410212	chr12_25409212_25410900	-8.1522176	-10.689255	5.1496E-06	0.004273991	H3K4me3	LD
chr12	25410215	25410216	chr12_25409212_25410900	-8.1522176	-10.689255	5.1496E-06	0.004273991	H3K4me3	LD
chr12	25410228	25410229	chr12_25409212_25410900	-8.1522176	-10.689255	5.1496E-06	0.004273991	H3K4me3	LD
chr12	25410234	25410235	chr12_25409212_25410900	-8.1522176	-10.689255	5.1496E-06	0.004273991	H3K4me3	LD
chr12	25410487	25410488	chr12_25409212_25410900	-8.1522176	-10.689255	5.1496E-06	0.004273991	H3K4me3	LD
chr12	5430369	5430370	chr12_5430169_5430980	4.3900986	9.9785786	8.6252E-06	0.004273991	H3K4me3	LD
chr12	5430375	5430376	chr12_5430169_5430980	4.3900986	9.9785786	8.6252E-06	0.004273991	H3K4me3	LD
chr12	5430387	5430388	chr12_5430169_5430980	4.3900986	9.9785786	8.6252E-06	0.004273991	H3K4me3	LD
chr12	5430522	5430523	chr12_5430169_5430980	4.3900986	9.9785786	8.6252E-06	0.004273991	H3K4me3	LD
chr12	5430753	5430754	chr12_5430169_5430980	4.3900986	9.9785786	8.6252E-06	0.004273991	H3K4me3	LD
chr12	5430879	5430880	chr12_5430169_5430980	4.3900986	9.9785786	8.6252E-06	0.004273991	H3K4me3	LD

chr12	5430926	5430927	chr12_5430169_5430980	4.3900986	9.9785786	8.6252E-06	0.004273991	H3K4me3	LD
chr12	5430941	5430942	chr12_5430169_5430980	4.3900986	9.9785786	8.6252E-06	0.004273991	H3K4me3	LD
chr13	31917470	31917471	chr13_31916315_31917993	27.5246	18.584778	7.2469E-08	0.000570549	H3K4me3	LD
chr13	200487780	200487781	chr13_200487488_200488625	-4.30023	-12.852336	1.2688E-06	0.002237819	H3K4me3	LD
chr13	200324509	200324510	chr13_200324319_200325867	-8.35426	-10.698467	5.1163E-06	0.002237819	H3K4me3	LD
chr13	200324617	200324618	chr13_200324319_200325867	-8.35426	-10.698467	5.1163E-06	0.002237819	H3K4me3	LD
chr13	200324664	200324665	chr13_200324319_200325867	-8.35426	-10.698467	5.1163E-06	0.002237819	H3K4me3	LD
chr13	200324919	200324920	chr13_200324319_200325867	-8.35426	-10.698467	5.1163E-06	0.002237819	H3K4me3	LD
chr13	200325018	200325019	chr13_200324319_200325867	-8.35426	-10.698467	5.1163E-06	0.002237819	H3K4me3	LD
chr13	200325044	200325045	chr13_200324319_200325867	-8.35426	-10.698467	5.1163E-06	0.002237819	H3K4me3	LD
chr13	200325103	200325104	chr13_200324319_200325867	-8.35426	-10.698467	5.1163E-06	0.002237819	H3K4me3	LD
chr13	200325191	200325192	chr13_200324319_200325867	-8.35426	-10.698467	5.1163E-06	0.002237819	H3K4me3	LD
chr13	200325200	200325201	chr13_200324319_200325867	-8.35426	-10.698467	5.1163E-06	0.002237819	H3K4me3	LD

chr13	200325239	200325240	chr13_200324319_200325867	-8.35426	-10.698467	5.1163E-06	0.002237819	H3K4me3	LD
chr13	200325250	200325251	chr13_200324319_200325867	-8.35426	-10.698467	5.1163E-06	0.002237819	H3K4me3	LD
chr13	200325268	200325269	chr13_200324319_200325867	-8.35426	-10.698467	5.1163E-06	0.002237819	H3K4me3	LD
chr13	200325308	200325309	chr13_200324319_200325867	-8.35426	-10.698467	5.1163E-06	0.002237819	H3K4me3	LD
chr13	200325432	200325433	chr13_200324319_200325867	-8.35426	-10.698467	5.1163E-06	0.002237819	H3K4me3	LD
chr13	200325433	200325434	chr13_200324319_200325867	-8.35426	-10.698467	5.1163E-06	0.002237819	H3K4me3	LD
chr13	200325777	200325778	chr13_200324319_200325867	-8.35426	-10.698467	5.1163E-06	0.002237819	H3K4me3	LD
chr14	103871416	103871417	chr14_103870486_103872766	-64.73108	-18.922148	6.2936E-08	9.79786E-05	H3K4me3	LD
chr14	103871456	103871457	chr14_103870486_103872766	-64.73108	-18.922148	6.2936E-08	9.79786E-05	H3K4me3	LD
chr14	103871460	103871461	chr14_103870486_103872766	-64.73108	-18.922148	6.2936E-08	9.79786E-05	H3K4me3	LD
chr14	103871526	103871527	chr14_103870486_103872766	-64.73108	-18.922148	6.2936E-08	9.79786E-05	H3K4me3	LD
chr14	5795944	5795945	chr14_5795114_5796004	-1.7163558	-18.908172	6.3302E-08	9.79786E-05	H3K4me3	LD
chr14	129660535	129660536	chr14_129660563_129661785	-21.234739	-12.210348	1.8778E-06	0.001816495	H3K4me3	LD

chr14	129660889	129660890	chr14_129660563_129661785	-21.234739	-12.210348	1.8778E-06	0.001816495	H3K4me3	LD
chr14	129661093	129661094	chr14_129660563_129661785	-21.234739	-12.210348	1.8778E-06	0.001816495	H3K4me3	LD
chr15	25301394	25301395	chr15_25301210_25301736	-9.6437536	-17.379051	1.225E-07	0.000818284	H3K4me3	LD
chr15	71496490	71496491	chr15_71496456_71498375	-15.83812	-11.936576	2.2322E-06	0.002982214	H3K4me3	LD
chr15	71497362	71497363	chr15_71496456_71498375	-15.83812	-11.936576	2.2322E-06	0.002982214	H3K4me3	LD
chr15	71497367	71497368	chr15_71496456_71498375	-15.83812	-11.936576	2.2322E-06	0.002982214	H3K4me3	LD
chr15	71498105	71498106	chr15_71496456_71498375	-15.83812	-11.936576	2.2322E-06	0.002982214	H3K4me3	LD
chr15	84718868	84718869	chr15_84718776_84719181	-3.2157279	-10.722211	5.0316E-06	0.005601864	H3K4me3	LD
chr16	66704986	66704987	chr16_66704996_66706920	-3.7515179	-17.215597	1.3188E-07	0.000451436	H3K4me3	LD
chr17	46622087	46622088	chr17_46622025_46622303	-7.7626564	-17.233289	1.3083E-07	0.000274153	H3K4me3	LD
chr17	46622271	46622272	chr17_46622025_46622303	-7.7626564	-17.233289	1.3083E-07	0.000274153	H3K4me3	LD
chr18	20664589	20664590	chr18_20664654_20665040	-4.2150767	-17.989822	9.3501E-08	0.000384384	H3K4me3	LD
chr18	5525971	5525972	chr18_5526032_5527404	2.1163981	12.5258155	1.5452E-06	0.002117435	H3K4me3	LD

chr18	5526004	5526005	chr18_5526032_5527404	2.1163981	12.5258155	1.5452E-06	0.002117435	H3K4me3	LD
chr18	2906083	2906084	chr18_2906059_2907447	-12.377799	-9.9626619	8.7285E-06	0.005980495	H3K4me3	LD
chr18	2906258	2906259	chr18_2906059_2907447	-12.377799	-9.9626619	8.7285E-06	0.005980495	H3K4me3	LD
chr18	2906918	2906919	chr18_2906059_2907447	-12.377799	-9.9626619	8.7285E-06	0.005980495	H3K4me3	LD
chr1	142412685	142412686	chr1_142412344_142413972	-52.095687	-19.828773	4.3587E-08	0.000547667	H3K4me3	LD
chr1	235835013	235835014	chr1_235834587_235836017	-21.274302	-13.958257	6.7241E-07	0.000844882	H3K4me3	LD
chr1	235835126	235835127	chr1_235834587_235836017	-21.274302	-13.958257	6.7241E-07	0.000844882	H3K4me3	LD
chr1	235835171	235835172	chr1_235834587_235836017	-21.274302	-13.958257	6.7241E-07	0.000844882	H3K4me3	LD
chr1	235835340	235835341	chr1_235834587_235836017	-21.274302	-13.958257	6.7241E-07	0.000844882	H3K4me3	LD
chr1	235835600	235835601	chr1_235834587_235836017	-21.274302	-13.958257	6.7241E-07	0.000844882	H3K4me3	LD
chr1	235835685	235835686	chr1_235834587_235836017	-21.274302	-13.958257	6.7241E-07	0.000844882	H3K4me3	LD
chr1	235835777	235835778	chr1_235834587_235836017	-21.274302	-13.958257	6.7241E-07	0.000844882	H3K4me3	LD
chr1	235835807	235835808	chr1_235834587_235836017	-21.274302	-13.958257	6.7241E-07	0.000844882	H3K4me3	LD

chr1	235835993	235835994	chr1_235834587_235836017	-21.274302	-13.958257	6.7241E-07	0.000844882	H3K4me3	LD
chr1	142256580	142256581	chr1_142256550_142257340	-9.333228	-10.57019	5.6024E-06	0.005414914	H3K4me3	LD
chr1	142256961	142256962	chr1_142256550_142257340	-9.333228	-10.57019	5.6024E-06	0.005414914	H3K4me3	LD
chr1	142257365	142257366	chr1_142256550_142257340	-9.333228	-10.57019	5.6024E-06	0.005414914	H3K4me3	LD
chr1	207493929	207493930	chr1_207493588_207494565	-5.0102229	-9.8291668	9.6521E-06	0.008662729	H3K4me3	LD
chr2	1483817	1483818	chr2_1482968_1483769	-25.482553	-28.821003	2.2728E-09	2.47961E-05	H3K4me3	LD
chr2	58412083	58412084	chr2_58411633_58412260	16.6782	12.8077686	1.303E-06	0.001821086	H3K4me3	LD
chr2	840021	840022	chr2_839970_841616	-13.279739	-12.400019	1.6692E-06	0.001821086	H3K4me3	LD
chr2	840053	840054	chr2_839970_841616	-13.279739	-12.400019	1.6692E-06	0.001821086	H3K4me3	LD
chr2	840241	840242	chr2_839970_841616	-13.279739	-12.400019	1.6692E-06	0.001821086	H3K4me3	LD
chr2	840621	840622	chr2_839970_841616	-13.279739	-12.400019	1.6692E-06	0.001821086	H3K4me3	LD
chr2	840628	840629	chr2_839970_841616	-13.279739	-12.400019	1.6692E-06	0.001821086	H3K4me3	LD
chr2	840781	840782	chr2_839970_841616	-13.279739	-12.400019	1.6692E-06	0.001821086	H3K4me3	LD

chr2	841205	841206	chr2_839970_841616	-13.279739	-12.400019	1.6692E-06	0.001821086	H3K4me3	LD
chr2	841531	841532	chr2_839970_841616	-13.279739	-12.400019	1.6692E-06	0.001821086	H3K4me3	LD
chr2	19153493	19153494	chr2_19153590_19154893	-3.2359172	-10.468763	6.0233E-06	0.005054962	H3K4me3	LD
chr2	19154355	19154356	chr2_19153590_19154893	-3.2359172	-10.468763	6.0233E-06	0.005054962	H3K4me3	LD
chr2	19154372	19154373	chr2_19153590_19154893	-3.2359172	-10.468763	6.0233E-06	0.005054962	H3K4me3	LD
chr3	4154504	4154505	chr3_4153523_4154780	-4.3879412	-22.958202	1.3745E-08	2.87995E-05	H3K4me3	LD
chr3	4154506	4154507	chr3_4153523_4154780	-4.3879412	-22.958202	1.3745E-08	2.87995E-05	H3K4me3	LD
chr3	4154523	4154524	chr3_4153523_4154780	-4.3879412	-22.958202	1.3745E-08	2.87995E-05	H3K4me3	LD
chr3	4154532	4154533	chr3_4153523_4154780	-4.3879412	-22.958202	1.3745E-08	2.87995E-05	H3K4me3	LD
chr3	4154590	4154591	chr3_4153523_4154780	-4.3879412	-22.958202	1.3745E-08	2.87995E-05	H3K4me3	LD
chr3	126704357	126704358	chr3_126704427_126706065	-10.869754	-19.239049	5.5247E-08	8.26816E-05	H3K4me3	LD
chr3	126704370	126704371	chr3_126704427_126706065	-10.869754	-19.239049	5.5247E-08	8.26816E-05	H3K4me3	LD
chr3	2089402	2089403	chr3_2089042_2091520	-12.967176	-13.228017	1.017E-06	0.000710303	H3K4me3	LD

chr3	2090575	2090576	chr3_2089042_2091520	-12.967176	-13.228017	1.017E-06	0.000710303	H3K4me3	LD
chr3	2090580	2090581	chr3_2089042_2091520	-12.967176	-13.228017	1.017E-06	0.000710303	H3K4me3	LD
chr3	2090660	2090661	chr3_2089042_2091520	-12.967176	-13.228017	1.017E-06	0.000710303	H3K4me3	LD
chr3	2090884	2090885	chr3_2089042_2091520	-12.967176	-13.228017	1.017E-06	0.000710303	H3K4me3	LD
chr3	2091141	2091142	chr3_2089042_2091520	-12.967176	-13.228017	1.017E-06	0.000710303	H3K4me3	LD
chr3	2091368	2091369	chr3_2089042_2091520	-12.967176	-13.228017	1.017E-06	0.000710303	H3K4me3	LD
chr3	2091504	2091505	chr3_2089042_2091520	-12.967176	-13.228017	1.017E-06	0.000710303	H3K4me3	LD
chr3	1996879	1996880	chr3_1996680_1997608	-3.0474949	-10.800228	4.7641E-06	0.002626802	H3K4me3	LD
chr3	1996949	1996950	chr3_1996680_1997608	-3.0474949	-10.800228	4.7641E-06	0.002626802	H3K4me3	LD
chr3	1997094	1997095	chr3_1996680_1997608	-3.0474949	-10.800228	4.7641E-06	0.002626802	H3K4me3	LD
chr3	1997107	1997108	chr3_1996680_1997608	-3.0474949	-10.800228	4.7641E-06	0.002626802	H3K4me3	LD
chr3	94253344	94253345	chr3_94251735_94255135	-49.055073	-9.9472996	8.8296E-06	0.004021696	H3K4me3	LD
chr3	94254539	94254540	chr3_94251735_94255135	-49.055073	-9.9472996	8.8296E-06	0.004021696	H3K4me3	LD

chr3	94254779	94254780	chr3_94251735_94255135	-49.055073	-9.9472996	8.8296E-06	0.004021696	H3K4me3	LD
chr3	94256221	94256222	chr3_94251735_94255135	-49.055073	-9.9472996	8.8296E-06	0.004021696	H3K4me3	LD
chr4	82385829	82385830	chr4_82384835_82386537	-12.743447	-22.383123	1.6791E-08	0.00014652	H3K4me3	LD
chr4	84165130	84165131	chr4_84164975_84165789	-6.806608	-10.613118	5.4342E-06	0.015806272	H3K4me3	LD
chr4	84165587	84165588	chr4_84164975_84165789	-6.806608	-10.613118	5.4342E-06	0.015806272	H3K4me3	LD
chr4	115750159	115750160	chr4_115749600_115750289	-4.4835391	-9.9152325	9.0449E-06	0.019731344	H3K4me3	LD
chr5	87629309	87629310	chr5_87629140_87629869	-4.5896515	-11.391098	3.185E-06	0.00125884	H3K4me3	LD
chr5	87629735	87629736	chr5_87629140_87629869	-4.5896515	-11.391098	3.185E-06	0.00125884	H3K4me3	LD
chr5	87629911	87629912	chr5_87629140_87629869	-4.5896515	-11.391098	3.185E-06	0.00125884	H3K4me3	LD
chr5	87629942	87629943	chr5_87629140_87629869	-4.5896515	-11.391098	3.185E-06	0.00125884	H3K4me3	LD
chr5	7009801	7009802	chr5_7009870_7011841	-8.549072	-10.638188	5.3386E-06	0.00125884	H3K4me3	LD
chr5	7009889	7009890	chr5_7009870_7011841	-8.549072	-10.638188	5.3386E-06	0.00125884	H3K4me3	LD
chr5	7010167	7010168	chr5_7009870_7011841	-8.549072	-10.638188	5.3386E-06	0.00125884	H3K4me3	LD

chr5	7010179	7010180	chr5_7009870_7011841	-8.549072	-10.638188	5.3386E-06	0.00125884	H3K4me3	LD
chr5	7010214	7010215	chr5_7009870_7011841	-8.549072	-10.638188	5.3386E-06	0.00125884	H3K4me3	LD
chr5	7010220	7010221	chr5_7009870_7011841	-8.549072	-10.638188	5.3386E-06	0.00125884	H3K4me3	LD
chr5	7010239	7010240	chr5_7009870_7011841	-8.549072	-10.638188	5.3386E-06	0.00125884	H3K4me3	LD
chr5	7010542	7010543	chr5_7009870_7011841	-8.549072	-10.638188	5.3386E-06	0.00125884	H3K4me3	LD
chr5	7010578	7010579	chr5_7009870_7011841	-8.549072	-10.638188	5.3386E-06	0.00125884	H3K4me3	LD
chr5	7010583	7010584	chr5_7009870_7011841	-8.549072	-10.638188	5.3386E-06	0.00125884	H3K4me3	LD
chr5	7010653	7010654	chr5_7009870_7011841	-8.549072	-10.638188	5.3386E-06	0.00125884	H3K4me3	LD
chr5	7010667	7010668	chr5_7009870_7011841	-8.549072	-10.638188	5.3386E-06	0.00125884	H3K4me3	LD
chr5	7010687	7010688	chr5_7009870_7011841	-8.549072	-10.638188	5.3386E-06	0.00125884	H3K4me3	LD
chr5	7011128	7011129	chr5_7009870_7011841	-8.549072	-10.638188	5.3386E-06	0.00125884	H3K4me3	LD
chr5	7011150	7011151	chr5_7009870_7011841	-8.549072	-10.638188	5.3386E-06	0.00125884	H3K4me3	LD
chr5	7011257	7011258	chr5_7009870_7011841	-8.549072	-10.638188	5.3386E-06	0.00125884	H3K4me3	LD

chr5	7011278	7011279	chr5_7009870_7011841	-8.549072	-10.638188	5.3386E-06	0.00125884	H3K4me3	LD
chr5	7011344	7011345	chr5_7009870_7011841	-8.549072	-10.638188	5.3386E-06	0.00125884	H3K4me3	LD
chr5	7011386	7011387	chr5_7009870_7011841	-8.549072	-10.638188	5.3386E-06	0.00125884	H3K4me3	LD
chr5	7011415	7011416	chr5_7009870_7011841	-8.549072	-10.638188	5.3386E-06	0.00125884	H3K4me3	LD
chr5	7011469	7011470	chr5_7009870_7011841	-8.549072	-10.638188	5.3386E-06	0.00125884	H3K4me3	LD
chr5	7011489	7011490	chr5_7009870_7011841	-8.549072	-10.638188	5.3386E-06	0.00125884	H3K4me3	LD
chr5	7011552	7011553	chr5_7009870_7011841	-8.549072	-10.638188	5.3386E-06	0.00125884	H3K4me3	LD
chr5	7011556	7011557	chr5_7009870_7011841	-8.549072	-10.638188	5.3386E-06	0.00125884	H3K4me3	LD
chr5	7011563	7011564	chr5_7009870_7011841	-8.549072	-10.638188	5.3386E-06	0.00125884	H3K4me3	LD
chr5	7011584	7011585	chr5_7009870_7011841	-8.549072	-10.638188	5.3386E-06	0.00125884	H3K4me3	LD
chr5	7011597	7011598	chr5_7009870_7011841	-8.549072	-10.638188	5.3386E-06	0.00125884	H3K4me3	LD
chr5	7011639	7011640	chr5_7009870_7011841	-8.549072	-10.638188	5.3386E-06	0.00125884	H3K4me3	LD
chr5	7011763	7011764	chr5_7009870_7011841	-8.549072	-10.638188	5.3386E-06	0.00125884	H3K4me3	LD

chr5	7011832	7011833	chr5_7009870_7011841	-8.549072	-10.638188	5.3386E-06	0.00125884	H3K4me3	LD
chr5	7011914	7011915	chr5_7009870_7011841	-8.549072	-10.638188	5.3386E-06	0.00125884	H3K4me3	LD
chr6	80467869	80467870	chr6_80467930_80468911	-6.0876378	-21.964992	1.9483E-08	4.56796E-05	H3K4me3	LD
chr6	80468117	80468118	chr6_80467930_80468911	-6.0876378	-21.964992	1.9483E-08	4.56796E-05	H3K4me3	LD
chr6	80468235	80468236	chr6_80467930_80468911	-6.0876378	-21.964992	1.9483E-08	4.56796E-05	H3K4me3	LD
chr6	80468387	80468388	chr6_80467930_80468911	-6.0876378	-21.964992	1.9483E-08	4.56796E-05	H3K4me3	LD
chr6	80468890	80468891	chr6_80467930_80468911	-6.0876378	-21.964992	1.9483E-08	4.56796E-05	H3K4me3	LD
chr6	37723904	37723905	chr6_37723752_37724778	-8.7932352	-12.378435	1.6916E-06	0.001322014	H3K4me3	LD
chr6	37723907	37723908	chr6_37723752_37724778	-8.7932352	-12.378435	1.6916E-06	0.001322014	H3K4me3	LD
chr6	37724021	37724022	chr6_37723752_37724778	-8.7932352	-12.378435	1.6916E-06	0.001322014	H3K4me3	LD
chr6	37724129	37724130	chr6_37723752_37724778	-8.7932352	-12.378435	1.6916E-06	0.001322014	H3K4me3	LD
chr6	37724131	37724132	chr6_37723752_37724778	-8.7932352	-12.378435	1.6916E-06	0.001322014	H3K4me3	LD
chr6	37724185	37724186	chr6_37723752_37724778	-8.7932352	-12.378435	1.6916E-06	0.001322014	H3K4me3	LD

chr6	37724350	37724351	chr6_37723752_37724778	-8.7932352	-12.378435	1.6916E-06	0.001322014	H3K4me3	LD
chr6	37724594	37724595	chr6_37723752_37724778	-8.7932352	-12.378435	1.6916E-06	0.001322014	H3K4me3	LD
chr6	37724596	37724597	chr6_37723752_37724778	-8.7932352	-12.378435	1.6916E-06	0.001322014	H3K4me3	LD
chr6	37724684	37724685	chr6_37723752_37724778	-8.7932352	-12.378435	1.6916E-06	0.001322014	H3K4me3	LD
chr8	130364048	130364049	chr8_130363938_130365652	-23.253373	-12.881909	1.2466E-06	0.002906291	H3K4me3	LD
chr8	130364072	130364073	chr8_130363938_130365652	-23.253373	-12.881909	1.2466E-06	0.002906291	H3K4me3	LD
chr8	130364074	130364075	chr8_130363938_130365652	-23.253373	-12.881909	1.2466E-06	0.002906291	H3K4me3	LD
chr9	130481506	130481507	chr9_130479550_130481668	-17.736721	-10.59933	5.4876E-06	0.02360762	H3K4me3	LD
chr9	130481597	130481598	chr9_130479550_130481668	-17.736721	-10.59933	5.4876E-06	0.02360762	H3K4me3	LD

Supplementary Table 5 Phased SVs significantly associated with phased histone modifications and CTCF signals

SV Region			SV Type	Histone Peaks	beta	t-stat	p-value	FDR	Histone Modification	Tissue
chr15	132594245	132594246	INS	chr15_132933680_132934130	-5.0144135	-23.815655	1.03E-08	0.02631321	CTCF	BF

chr17	7404741	7404742	INS	chr17_7452934_7453627	3.55952613	20.2586785	3.68E-08	0.04708346	CTCF	BF
NW_018084956.1	31272	31273	INS	NW_018084956.1_36294_36638	21.3507888	20.8778038	2.91E-08	0.04708346	CTCF	BF
chr5	62540128	62540433	DEL	chr5_62914084_62914990	3.38639638	23.8771643	1.01E-08	0.00010303	H3K27ac	BF
chr4	18227611	18227612	INS	chr4_18390534_18391190	9.720785	23.7503311	1.05E-08	0.00010672	H3K27ac	BF
chr1	20834452	20834453	INS	chr1_20583468_20584418	4.69098188	23.7564094	1.05E-08	0.00010672	H3K27ac	BF
chr1	11626419	11626420	INS	chr1_12029242_12030384	5.21703	23.5656652	1.12E-08	0.00011235	H3K27ac	BF
chr4	99069475	99069639	DEL	chr4_98650684_98651198	8.41497294	23.3326018	1.21E-08	0.00011791	H3K27ac	BF
chr4	98299041	98299042	INS	chr4_98650684_98651198	8.41497294	23.3326018	1.21E-08	0.00011791	H3K27ac	BF
chr4	98512489	98512490	INS	chr4_98650684_98651198	8.41497294	23.3326018	1.21E-08	0.00011791	H3K27ac	BF
chr4	98731523	98731524	INS	chr4_98650684_98651198	8.41497294	23.3326018	1.21E-08	0.00011791	H3K27ac	BF
chr4	98803258	98803259	INS	chr4_98650684_98651198	8.41497294	23.3326018	1.21E-08	0.00011791	H3K27ac	BF
chr1	6460049	6460050	INS	chr1_6771600_6773792	14.626355	23.3583456	1.20E-08	0.00011791	H3K27ac	BF
chr1	107526690	107526691	INS	chr1_107361691_107361861	5.67715531	23.3479091	1.20E-08	0.00011791	H3K27ac	BF
chr1	6643056	6643057	INS	chr1_6771600_6773792	14.626355	23.3583456	1.20E-08	0.00011791	H3K27ac	BF
chr1	6643056	6643057	INS	chr1_7142109_7143698	6.24506813	23.4065076	1.18E-08	0.00011791	H3K27ac	BF
chr1	104052039	104052552	DEL	chr1_104452478_104453229	16.0187816	23.1569271	1.28E-08	0.00012459	H3K27ac	BF
chr1	8356436	8356437	INS	chr1_8595461_8595739	9.52687581	23.1499401	1.29E-08	0.00012459	H3K27ac	BF
chr1	20834452	20834453	INS	chr1_20897681_20898052	4.06396881	23.1415086	1.29E-08	0.00012459	H3K27ac	BF
chr5	62540128	62540433	DEL	chr5_62602255_62602587	5.95608131	23.0253058	1.34E-08	0.00012879	H3K27ac	BF
chr5	73979409	73979468	DEL	chr5_73636495_73637054	8.01150563	23.0219933	1.34E-08	0.00012879	H3K27ac	BF
chr1	20834452	20834453	INS	chr1_20690675_20690880	8.58127525	23.0160588	1.35E-08	0.00012879	H3K27ac	BF
chr3	121657515	121657516	INS	chr3_122143953_122144206	4.529872	22.9688074	1.37E-08	0.00012963	H3K27ac	BF
chr3	122636402	122636403	INS	chr3_122143953_122144206	4.529872	22.9688074	1.37E-08	0.00012963	H3K27ac	BF
chr1	20834452	20834453	INS	chr1_20952222_20952830	7.25044363	22.950876	1.38E-08	0.00013002	H3K27ac	BF
chr1	20834452	20834453	INS	chr1_20765073_20765492	10.6729838	22.9185739	1.39E-08	0.00013105	H3K27ac	BF

chr1	20834452	20834453	INS	chr1_20895770_20896451	9.22047956	22.8885015	1.41E-08	0.000132	H3K27ac	BF
chr1	20834452	20834453	INS	chr1_20531021_20531367	3.42626981	22.8433189	1.43E-08	0.00013365	H3K27ac	BF
chr1	8356436	8356437	INS	chr1_8151888_8152850	5.34817706	22.81638	1.44E-08	0.00013447	H3K27ac	BF
chr3	68144528	68144998	DEL	chr3_68151655_68152005	4.35951881	22.7801578	1.46E-08	0.00013532	H3K27ac	BF
chr3	68141059	68142597	INV	chr3_68151655_68152005	8.71903763	22.7801578	1.46E-08	0.00013532	H3K27ac	BF
chr3	121657515	121657516	INS	chr3_121673268_121673681	7.9391765	22.731176	1.49E-08	0.0001372	H3K27ac	BF
chr4	122536980	122537187	DEL	chr4_122948660_122948937	9.60159239	22.6588596	1.52E-08	0.00013811	H3K27ac	BF
chr4	122853009	122853218	DEL	chr4_122948660_122948937	9.60159239	22.6588596	1.52E-08	0.00013811	H3K27ac	BF
chr4	122888381	122890004	DEL	chr4_122948660_122948937	9.60159239	22.6588596	1.52E-08	0.00013811	H3K27ac	BF
chr4	122750310	122750311	INS	chr4_122948660_122948937	9.60159239	22.6588596	1.52E-08	0.00013811	H3K27ac	BF
chr4	123025234	123025235	INS	chr4_122948660_122948937	9.60159239	22.6588596	1.52E-08	0.00013811	H3K27ac	BF
chr4	123447592	123447593	INS	chr4_122948660_122948937	9.60159239	22.6588596	1.52E-08	0.00013811	H3K27ac	BF
chr5	7581505	7581559	DEL	chr5_7325056_7325539	1.917425	22.6217289	1.54E-08	0.00013905	H3K27ac	BF
chr5	7579345	7579621	DEL	chr5_7325056_7325539	1.917425	22.6217289	1.54E-08	0.00013905	H3K27ac	BF
chr1	11790648	11790649	INS	chr1_12074910_12075305	6.59786556	22.4814784	1.62E-08	0.00014472	H3K27ac	BF
chr1	12305780	12305781	INS	chr1_12074910_12075305	6.59786556	22.4814784	1.62E-08	0.00014472	H3K27ac	BF
chr1	6460049	6460050	INS	chr1_6766244_6766464	9.11988288	22.4457158	1.64E-08	0.00014567	H3K27ac	BF
chr1	6643056	6643057	INS	chr1_6766244_6766464	9.11988288	22.4457158	1.64E-08	0.00014567	H3K27ac	BF
chr4	18955154	18955155	INS	chr4_18892252_18892416	25.6580643	22.3004509	1.73E-08	0.00015286	H3K27ac	BF
chr2	48297275	48297336	DEL	chr2_47840369_47841379	4.21715738	22.243537	1.76E-08	0.00015551	H3K27ac	BF
chr1	8356436	8356437	INS	chr1_8189416_8190998	13.4343694	22.2157311	1.78E-08	0.00015658	H3K27ac	BF
chr1	11626419	11626420	INS	chr1_11963625_11964294	3.79766863	22.1719297	1.81E-08	0.00015763	H3K27ac	BF
chr2	115158984	115158985	INS	chr2_115218287_115219617	6.55492817	22.1311158	1.84E-08	0.000159	H3K27ac	BF
chr2	115657114	115657115	INS	chr2_115218287_115219617	6.55492817	22.1311158	1.84E-08	0.000159	H3K27ac	BF
chr1	205959403	205959404	INS	chr1_206181813_206182735	10.4252729	22.1088521	1.85E-08	0.0001598	H3K27ac	BF
chr1	108733962	108734028	DEL	chr1_108835221_108835815	12.1237722	21.8638729	2.02E-08	0.00017346	H3K27ac	BF

chr1	6643056	6643057	INS	chr1_6976650_6976954	6.42476119	21.7793563	2.08E-08	0.00017831	H3K27ac	BF
chr1	8356436	8356437	INS	chr1_8080421_8081176	4.62218919	21.6524759	2.18E-08	0.00018618	H3K27ac	BF
chr5	4189079	4189168	DEL	chr5_3943375_3945233	12.6404817	21.5978328	2.23E-08	0.00018721	H3K27ac	BF
chr5	4360466	4360685	DEL	chr5_3943375_3945233	12.6404817	21.5978328	2.23E-08	0.00018721	H3K27ac	BF
chr5	4388727	4388728	INS	chr5_3943375_3945233	12.6404817	21.5978328	2.23E-08	0.00018721	H3K27ac	BF
chr5	4439469	4439470	INS	chr5_3943375_3945233	12.6404817	21.5978328	2.23E-08	0.00018721	H3K27ac	BF
chr1	8356436	8356437	INS	chr1_8587053_8587398	4.82781106	21.6107436	2.21E-08	0.00018721	H3K27ac	BF
chr2	13122273	13123683	DEL	chr2_13437062_13437539	17.5002797	21.5439434	2.27E-08	0.00019039	H3K27ac	BF
chr3	55449949	55450016	DEL	chr3_55729071_55730067	3.90118438	21.4939476	2.31E-08	0.00019166	H3K27ac	BF
chr3	55300034	55300035	INS	chr3_55729071_55730067	3.90118438	21.4939476	2.31E-08	0.00019166	H3K27ac	BF
chr1	11626419	11626420	INS	chr1_12028579_12028930	7.34842181	21.4872578	2.32E-08	0.00019166	H3K27ac	BF
chr5	11021536	11021537	INS	chr5_11403360_11404175	21.7585839	21.3870778	2.40E-08	0.00019665	H3K27ac	BF
chr5	11050179	11050180	INS	chr5_11403360_11404175	21.7585839	21.3870778	2.40E-08	0.00019665	H3K27ac	BF
chr5	11265336	11265337	INS	chr5_11403360_11404175	21.7585839	21.3870778	2.40E-08	0.00019665	H3K27ac	BF
chr1	20834452	20834453	INS	chr1_20956752_20957600	12.3494688	21.4014651	2.39E-08	0.00019665	H3K27ac	BF
chr1	127851484	127851485	INS	chr1_127793484_127793759	10.7077428	21.3492826	2.44E-08	0.00019885	H3K27ac	BF
chr1	71451857	71459227	DEL	chr1_71601949_71602686	3.28633156	21.317929	2.47E-08	0.00019988	H3K27ac	BF
chr1	71347317	71347318	INS	chr1_71601949_71602686	3.28633156	21.317929	2.47E-08	0.00019988	H3K27ac	BF
chr5	23006530	23006531	INS	chr5_22767001_22767580	5.23466278	21.3129662	2.47E-08	0.00019988	H3K27ac	BF
chr4	62680082	62680083	INS	chr4_62510763_62511942	11.5581463	21.2842805	2.50E-08	0.00020146	H3K27ac	BF
chr5	47270892	47270956	DEL	chr5_46967936_46970992	10.1109489	21.1965871	2.58E-08	0.00020699	H3K27ac	BF
chr1	129061546	129061547	INS	chr1_129119221_129119627	-7.195679	-21.198791	2.58E-08	0.00020699	H3K27ac	BF
chr5	44693415	44693597	DEL	chr5_44196072_44196331	13.2816181	21.0916249	2.68E-08	0.00021465	H3K27ac	BF
chr1	103658379	103658467	DEL	chr1_103678372_103678619	6.50651517	21.0529567	2.72E-08	0.0002166	H3K27ac	BF
chr1	103354885	103354886	INS	chr1_103678372_103678619	6.50651517	21.0529567	2.72E-08	0.0002166	H3K27ac	BF
chr1	205959403	205959404	INS	chr1_205703754_205704263	1.95637669	21.0147139	2.76E-08	0.00021914	H3K27ac	BF

chr2	129370678	129370828	DEL	chr2_129583786_129584130	5.096025	20.9672944	2.81E-08	0.00022186	H3K27ac	BF
chr2	129690948	129690949	INS	chr2_129583786_129584130	5.096025	20.9672944	2.81E-08	0.00022186	H3K27ac	BF
chr5	13941115	13941116	INS	chr5_14435802_14436857	6.67602111	20.9391976	2.84E-08	0.00022186	H3K27ac	BF
chr5	14116104	14116105	INS	chr5_14435802_14436857	6.67602111	20.9391976	2.84E-08	0.00022186	H3K27ac	BF
chr5	14186300	14186301	INS	chr5_14435802_14436857	6.67602111	20.9391976	2.84E-08	0.00022186	H3K27ac	BF
chr5	14416409	14416410	INS	chr5_14435802_14436857	6.67602111	20.9391976	2.84E-08	0.00022186	H3K27ac	BF
chr1	8356436	8356437	INS	chr1_8423252_8424046	4.68238188	20.8161072	2.97E-08	0.00023178	H3K27ac	BF
chr1	169103127	169103652	DEL	chr1_169338006_169339554	4.94082594	20.7577783	3.04E-08	0.00023266	H3K27ac	BF
chr1	169811358	169811510	DEL	chr1_169338006_169339554	4.94082594	20.7577783	3.04E-08	0.00023266	H3K27ac	BF
chr1	169824231	169824532	DEL	chr1_169338006_169339554	4.94082594	20.7577783	3.0409E-08	0.00023266	H3K27ac	BF
chr5	31001378	31001673	DEL	chr5_30542599_30543884	6.7226775	20.7639158	3.0339E-08	0.00023266	H3K27ac	BF
chr1	168946938	168946939	INS	chr1_169338006_169339554	4.94082594	20.7577783	3.0409E-08	0.00023266	H3K27ac	BF
chr1	169267306	169267307	INS	chr1_169338006_169339554	4.94082594	20.7577783	3.0409E-08	0.00023266	H3K27ac	BF
chr1	169593891	169593892	INS	chr1_169338006_169339554	4.94082594	20.7577783	3.0409E-08	0.00023266	H3K27ac	BF
chr2	3399845	3399897	DEL	chr2_3100486_3100734	7.10899772	20.710756	3.0957E-08	0.00023562	H3K27ac	BF
chr2	2823175	2823176	INS	chr2_3100486_3100734	7.10899772	20.710756	3.0957E-08	0.00023562	H3K27ac	BF
chr4	117978801	117978916	DEL	chr4_117680527_117681521	9.53050833	20.472828	3.3902E-08	0.00024844	H3K27ac	BF

chr4	118001729	118001880	DEL	chr4_117680527_117681521	9.53050833	20.472828	3.3902E-08	0.00024844	H3K27ac	BF
chr4	117999909	118000198	DEL	chr4_117680527_117681521	9.53050833	20.472828	3.3902E-08	0.00024844	H3K27ac	BF
chr5	7581505	7581559	DEL	chr5_8080948_8081627	6.17302938	20.5155555	3.3351E-08	0.00024844	H3K27ac	BF
chr2	48297275	48297336	DEL	chr2_48425406_48426454	3.991163	20.5233049	3.3252E-08	0.00024844	H3K27ac	BF
chr4	117473079	117473080	INS	chr4_117680527_117681521	9.53050833	20.472828	3.3902E-08	0.00024844	H3K27ac	BF
chr4	117746392	117746393	INS	chr4_117680527_117681521	9.53050833	20.472828	3.3902E-08	0.00024844	H3K27ac	BF
chr4	117945046	117945047	INS	chr4_117680527_117681521	9.53050833	20.472828	3.3902E-08	0.00024844	H3K27ac	BF
chr4	118002624	118002625	INS	chr4_117680527_117681521	9.53050833	20.472828	3.3902E-08	0.00024844	H3K27ac	BF
chr4	118005675	118005676	INS	chr4_117680527_117681521	9.53050833	20.472828	3.3902E-08	0.00024844	H3K27ac	BF
chr4	118083331	118083332	INS	chr4_117680527_117681521	9.53050833	20.472828	3.3902E-08	0.00024844	H3K27ac	BF
chr4	118131010	118131011	INS	chr4_117680527_117681521	9.53050833	20.472828	3.3902E-08	0.00024844	H3K27ac	BF
chr1	6643056	6643057	INS	chr1_6965289_6965908	14.6935194	20.4852765	3.3741E-08	0.00024844	H3K27ac	BF
chr1	129061546	129061547	INS	chr1_129111800_129112265	-6.7889288	-20.481068	3.3795E-08	0.00024844	H3K27ac	BF

chr5	73979409	73979468	DEL	chr5_74230518_74230964	4.09828044	20.3708579	3.526E-08	0.00025714	H3K27ac	BF
chr2	134080328	134081531	DEL	chr2_134339180_134339456	12.9616521	20.3706238	3.5263E-08	0.00025714	H3K27ac	BF
chr1	14465696	14465697	INS	chr1_14943521_14943890	6.62905644	20.2538391	3.6894E-08	0.00026446	H3K27ac	BF
chr1	14854631	14854632	INS	chr1_14943521_14943890	6.62905644	20.2538391	3.6894E-08	0.00026446	H3K27ac	BF
chr1	14957571	14957572	INS	chr1_14943521_14943890	6.62905644	20.2538391	3.6894E-08	0.00026446	H3K27ac	BF
chr1	15227869	15227870	INS	chr1_14943521_14943890	6.62905644	20.2538391	3.6894E-08	0.00026446	H3K27ac	BF
chr1	15287464	15287465	INS	chr1_14943521_14943890	6.62905644	20.2538391	3.6894E-08	0.00026446	H3K27ac	BF
chr1	15289209	15289210	INS	chr1_14943521_14943890	6.62905644	20.2538391	3.6894E-08	0.00026446	H3K27ac	BF
chr4	71017558	71017841	DEL	chr4_71040918_71041585	8.06495081	20.2368905	3.7138E-08	0.00026556	H3K27ac	BF
chr1	96099461	96100904	DEL	chr1_95744594_95745150	2.32779561	20.1452833	3.8486E-08	0.00027256	H3K27ac	BF
chr1	95497423	95497424	INS	chr1_95744594_95745150	2.32779561	20.1452833	3.8486E-08	0.00027256	H3K27ac	BF
chr1	96017447	96017448	INS	chr1_95744594_95745150	2.32779561	20.1452833	3.8486E-08	0.00027256	H3K27ac	BF
chr1	96026418	96026419	INS	chr1_95744594_95745150	2.32779561	20.1452833	3.8486E-08	0.00027256	H3K27ac	BF

chr1	7056618	7056619	INS	chr1_6682109_6682569	10.4087495	20.1226554	3.8828E-08	0.00027367	H3K27ac	BF
chr2	117010214	117013747	DEL	chr2_117047977_117048507	5.4701665	20.1032195	3.9124E-08	0.00027445	H3K27ac	BF
chr2	117057518	117057519	INS	chr2_117047977_117048507	5.4701665	20.1032195	3.9124E-08	0.00027445	H3K27ac	BF
chr2	48297275	48297336	DEL	chr2_47864949_47865278	12.7573777	20.0499053	3.9949E-08	0.00027957	H3K27ac	BF
chr5	77783286	77783287	INS	chr5_77487718_77488058	4.09368719	20.0117829	4.0551E-08	0.00028311	H3K27ac	BF
chr2	143620924	143620983	DEL	chr2_143730327_143731618	6.33075611	19.9514918	4.1524E-08	0.00028854	H3K27ac	BF
chr2	143620660	143620661	INS	chr2_143730327_143731618	6.33075611	19.9514918	4.1524E-08	0.00028854	H3K27ac	BF
chr2	132195622	132195703	DEL	chr2_132585635_132586308	5.63532494	19.9232755	4.1988E-08	0.00028905	H3K27ac	BF
chr2	132813015	132813092	DEL	chr2_132585635_132586308	5.63532494	19.9232755	4.1988E-08	0.00028905	H3K27ac	BF
chr2	132846886	132847667	DEL	chr2_132585635_132586308	5.63532494	19.9232755	4.1988E-08	0.00028905	H3K27ac	BF
chr2	132420954	132420955	INS	chr2_132585635_132586308	5.63532494	19.9232755	4.1988E-08	0.00028905	H3K27ac	BF
chr4	116597067	116597068	INS	chr4_116959695_116962349	13.7212622	19.8093819	4.3923E-08	0.00030096	H3K27ac	BF
chr4	116604576	116604577	INS	chr4_116959695_116962349	13.7212622	19.8093819	4.3923E-08	0.00030096	H3K27ac	BF

chr1	11626419	11626420	INS	chr1_12102719_12103019	8.51102813	19.7861341	4.433E-08	0.00030165	H3K27ac	BF
chr5	7581505	7581559	DEL	chr5_7848565_7848874	4.67560313	19.7286951	4.5354E-08	0.00030455	H3K27ac	BF
chr5	7579345	7579621	DEL	chr5_7848565_7848874	4.67560313	19.7286951	4.5354E-08	0.00030455	H3K27ac	BF
chr1	6460049	6460050	INS	chr1_6804149_6804849	7.19538906	19.7367767	4.5208E-08	0.00030455	H3K27ac	BF
chr1	6643056	6643057	INS	chr1_6804149_6804849	7.19538906	19.7367767	4.5208E-08	0.00030455	H3K27ac	BF
chr5	10454542	10454543	INS	chr5_10703224_10703592	5.79279644	19.7218633	4.5478E-08	0.00030455	H3K27ac	BF
chr5	11021536	11021537	INS	chr5_10703224_10703592	5.79279644	19.7218633	4.5478E-08	0.00030455	H3K27ac	BF
chr5	11050179	11050180	INS	chr5_10703224_10703592	5.79279644	19.7218633	4.5478E-08	0.00030455	H3K27ac	BF
chr5	73979409	73979468	DEL	chr5_73902416_73903294	10.5095331	19.6988413	4.5897E-08	0.00030662	H3K27ac	BF
chr3	105682570	105682767	DEL	chr3_106168340_106170060	2.21273	19.6822275	4.6202E-08	0.00030662	H3K27ac	BF
chr3	105713637	105713638	INS	chr3_106168340_106170060	2.21273	19.6822275	4.6202E-08	0.00030662	H3K27ac	BF
chr3	106398685	106398686	INS	chr3_106168340_106170060	2.21273	19.6822275	4.6202E-08	0.00030662	H3K27ac	BF
chr3	1350909	1351109	DEL	chr3_1149037_1149713	13.717429	19.5528004	4.8659E-08	0.00031792	H3K27ac	BF

chr3	1477380	1477559	DEL	chr3_1149037_1149713	13.717429	19.5528004	4.8659E-08	0.00031792	H3K27ac	BF
chr3	1506478	1506548	DEL	chr3_1149037_1149713	13.717429	19.5528004	4.8659E-08	0.00031792	H3K27ac	BF
chr3	1365025	1365026	INS	chr3_1149037_1149713	13.717429	19.5528004	4.8659E-08	0.00031792	H3K27ac	BF
chr3	1491930	1491931	INS	chr3_1149037_1149713	13.717429	19.5528004	4.8659E-08	0.00031792	H3K27ac	BF
chr3	19687944	19687945	INS	chr3_19503807_19504103	7.00451131	19.4610934	5.0489E-08	0.00032842	H3K27ac	BF
chr4	103324840	103325032	DEL	chr4_103083356_103083695	8.15499406	19.3218479	5.3416E-08	0.00034219	H3K27ac	BF
chr5	7581505	7581559	DEL	chr5_7582527_7584370	9.3042375	19.3428296	5.2963E-08	0.00034219	H3K27ac	BF
chr5	7579345	7579621	DEL	chr5_7582527_7584370	9.3042375	19.3428296	5.2963E-08	0.00034219	H3K27ac	BF
chr4	102782001	102782002	INS	chr4_103083356_103083695	8.15499406	19.3218479	5.3416E-08	0.00034219	H3K27ac	BF
chr4	103323208	103323209	INS	chr4_103083356_103083695	8.15499406	19.3218479	5.3416E-08	0.00034219	H3K27ac	BF
chr1	114488786	114488787	INS	chr1_114434985_114435206	4.59657478	19.2693508	5.4569E-08	0.00034732	H3K27ac	BF
chr1	114620312	114620313	INS	chr1_114434985_114435206	4.59657478	19.2693508	5.4569E-08	0.00034732	H3K27ac	BF
chr1	114614128	114614129	INS	chr1_114434985_114435206	4.59657478	19.2693508	5.4569E-08	0.00034732	H3K27ac	BF

chr2	126596215	126596496	DEL	chr2_126633867_126635540	12.5446289	19.2526301	5.4942E-08	0.00034894	H3K27ac	BF
chr1	129061546	129061547	INS	chr1_128918538_128920412	-16.831884	-19.233011	5.5384E-08	0.00035099	H3K27ac	BF
chr5	54104163	54104164	INS	chr5_54285482_54285992	5.27240844	19.219894	5.5681E-08	0.00035212	H3K27ac	BF
chr5	16740917	16741233	DEL	chr5_16246500_16246873	7.45459178	19.2046588	5.6029E-08	0.00035345	H3K27ac	BF
chr1	79764713	79764714	INS	chr1_79545438_79545724	15.6698134	19.194953	5.6251E-08	0.00035345	H3K27ac	BF
chr1	79765548	79765549	INS	chr1_79545438_79545724	15.6698134	19.194953	5.6251E-08	0.00035345	H3K27ac	BF
chr2	134080328	134081531	DEL	chr2_134359325_134359707	17.4322371	19.1718438	5.6785E-08	0.00035605	H3K27ac	BF
chr2	48297275	48297336	DEL	chr2_47841930_47842877	4.29985369	19.1541907	5.7197E-08	0.00035788	H3K27ac	BF
chr8	41223208	41783661	DUP	chr8_41401817_41403400	23.6634665	14.2467895	5.7535E-08	0.00078348	H3K27ac	BF
chr5	7581505	7581559	DEL	chr5_8051395_8052237	5.23888438	19.1044406	5.8376E-08	0.00036371	H3K27ac	BF
chr5	7579345	7579621	DEL	chr5_8051395_8052237	5.23888438	19.1044406	5.8376E-08	0.00036371	H3K27ac	BF
chr5	7581505	7581559	DEL	chr5_7514295_7515937	-5.8439548	-19.021562	6.0402E-08	0.00037475	H3K27ac	BF
chr5	7579345	7579621	DEL	chr5_7514295_7515937	-5.8439548	-19.021562	6.0402E-08	0.00037475	H3K27ac	BF

chr1	79764713	79764714	INS	chr1_79424969_79426158	15.9480647	18.9619992	6.1906E-08	0.00038248	H3K27ac	BF
chr1	79765548	79765549	INS	chr1_79424969_79426158	15.9480647	18.9619992	6.1906E-08	0.00038248	H3K27ac	BF
chr2	143620924	143620983	DEL	chr2_143475358_143476173	7.14787833	18.8924666	6.3716E-08	0.00039202	H3K27ac	BF
chr2	143620660	143620661	INS	chr2_143475358_143476173	7.14787833	18.8924666	6.3716E-08	0.00039202	H3K27ac	BF
chr3	68144528	68144998	DEL	chr3_68359161_68360535	10.4288581	18.7477866	6.7674E-08	0.00041464	H3K27ac	BF
chr3	68141059	68142597	INV	chr3_68359161_68360535	20.8577161	18.7477866	6.7674E-08	0.00041464	H3K27ac	BF
chr3	76926501	76926709	DEL	chr3_76772123_76772470	5.24349322	18.709702	6.8762E-08	0.00042043	H3K27ac	BF
chr5	7581505	7581559	DEL	chr5_7338885_7339913	4.03870619	18.6494409	7.0523E-08	0.00042942	H3K27ac	BF
chr5	7579345	7579621	DEL	chr5_7338885_7339913	4.03870619	18.6494409	7.0523E-08	0.00042942	H3K27ac	BF
chr1	107526690	107526691	INS	chr1_107834989_107835701	5.80776444	18.6031662	7.191E-08	0.00043697	H3K27ac	BF
chr4	116550806	116550807	INS	chr4_116635114_116635569	4.71483688	18.5937187	7.2196E-08	0.00043781	H3K27ac	BF
chr1	129061546	129061547	INS	chr1_128880119_128880834	-19.289711	-18.568318	7.2974E-08	0.00044162	H3K27ac	BF
chr4	71017558	71017841	DEL	chr4_70833641_70835012	10.4930594	18.5595413	7.3245E-08	0.00044235	H3K27ac	BF

chr3	19383684	19383685	INS	chr3_19503807_19504103	6.95371617	18.5279439	7.4229E-08	0.00044738	H3K27ac	BF
chr1	8356436	8356437	INS	chr1_8090070_8090759	8.6582715	18.4926756	7.5346E-08	0.00045319	H3K27ac	BF
chr1	8356436	8356437	INS	chr1_8412941_8413420	8.02982563	18.4388874	7.7085E-08	0.0004627	H3K27ac	BF
chr1	21471187	21471247	DEL	chr1_21825432_21826248	9.9643645	18.3371748	8.0499E-08	0.00048221	H3K27ac	BF
chr3	6483195	6484571	DEL	chr3_6131339_6131696	19.2620767	18.2427633	8.382E-08	0.00048997	H3K27ac	BF
chr5	7581505	7581559	DEL	chr5_8075599_8077228	2.3897225	18.2437832	8.3783E-08	0.00048997	H3K27ac	BF
chr5	7579345	7579621	DEL	chr5_8075599_8077228	2.3897225	18.2437832	8.3783E-08	0.00048997	H3K27ac	BF
chr1	248582916	248583799	DEL	chr1_248245692_248246238	5.15717689	18.2697048	8.2857E-08	0.00048997	H3K27ac	BF
chr1	1511558	1511813	DEL	chr1_1827946_1828127	4.77528822	18.2391305	8.3951E-08	0.00048997	H3K27ac	BF
chr1	1697599	1697690	DEL	chr1_1827946_1828127	4.77528822	18.2391305	8.3951E-08	0.00048997	H3K27ac	BF
chr1	2307186	2307261	DEL	chr1_1827946_1828127	4.77528822	18.2391305	8.3951E-08	0.00048997	H3K27ac	BF
chr2	48297275	48297336	DEL	chr2_48201620_48202939	2.168418	18.2599804	8.3203E-08	0.00048997	H3K27ac	BF
chr3	5686302	5686303	INS	chr3_6131339_6131696	19.2620767	18.2427633	8.382E-08	0.00048997	H3K27ac	BF

chr1	190223373	190223374	INS	chr1_190717200_190717771	4.45198713	18.2606831	8.3178E-08	0.00048997	H3K27ac	BF
chr1	1779044	1779045	INS	chr1_1827946_1828127	4.77528822	18.2391305	8.3951E-08	0.00048997	H3K27ac	BF
chr5	28909531	28909532	INS	chr5_28574474_28575170	4.18545644	18.2710784	8.2808E-08	0.00048997	H3K27ac	BF
chr4	123447592	123447593	INS	chr4_123537713_123539769	9.00543	18.2212301	8.4599E-08	0.0004919	H3K27ac	BF
chr1	268866273	268866274	INS	chr1_268474301_268476790	21.148995	18.2208143	8.4614E-08	0.0004919	H3K27ac	BF
chr1	261708365	261709520	DEL	chr1_261717266_261718658	6.77864	18.2115704	8.4951E-08	0.00049289	H3K27ac	BF
chr1	188641399	188641400	INS	chr1_188565487_188565870	2.593702	18.1596609	8.6871E-08	0.00050304	H3K27ac	BF
chr1	17287767	17287945	DEL	chr1_16957092_16957830	7.3826385	18.100067	8.9135E-08	0.00051514	H3K27ac	BF
chr5	31001378	31001673	DEL	chr5_31063964_31064399	5.02988369	18.0593379	9.0721E-08	0.00052328	H3K27ac	BF
chr5	16740917	16741233	DEL	chr5_16455003_16455316	11.4385617	18.040375	9.147E-08	0.00052657	H3K27ac	BF
chr4	70506310	70506311	INS	chr4_70894632_70895799	2.92423222	17.9577633	9.4816E-08	0.00054266	H3K27ac	BF
chr4	70522463	70522464	INS	chr4_70894632_70895799	2.92423222	17.9577633	9.4816E-08	0.00054266	H3K27ac	BF
chr4	70549506	70549507	INS	chr4_70894632_70895799	2.92423222	17.9577633	9.4816E-08	0.00054266	H3K27ac	BF

chr5	7581505	7581559	DEL	chr5_7685860_7686492	3.80486906	17.9451927	9.5337E-08	0.00054354	H3K27ac	BF
chr5	7579345	7579621	DEL	chr5_7685860_7686492	3.80486906	17.9451927	9.5337E-08	0.00054354	H3K27ac	BF
chr2	13122273	13123683	DEL	NW_018085198.1_15208_16770	11.89749	17.9336195	9.5819E-08	0.00054523	H3K27ac	BF
chr1	6643056	6643057	INS	chr1_7118003_7118294	5.23199063	17.8540458	9.9212E-08	0.00056345	H3K27ac	BF
chr2	88716448	88716528	DEL	chr2_88933496_88933679	3.49489294	17.8055733	1.0134E-07	0.00056684	H3K27ac	BF
chr2	88713249	88713539	DEL	chr2_88933496_88933679	3.49489294	17.8055733	1.0134E-07	0.00056684	H3K27ac	BF
chr2	88810819	88810944	DEL	chr2_88933496_88933679	3.49489294	17.8055733	1.0134E-07	0.00056684	H3K27ac	BF
chr2	88807058	88807258	DEL	chr2_88933496_88933679	3.49489294	17.8055733	1.0134E-07	0.00056684	H3K27ac	BF
chr2	89340281	89340351	DEL	chr2_88933496_88933679	3.49489294	17.8055733	1.0134E-07	0.00056684	H3K27ac	BF
chr2	89364651	89364734	DEL	chr2_88933496_88933679	3.49489294	17.8055733	1.0134E-07	0.00056684	H3K27ac	BF
chr2	89390169	89390170	INS	chr2_88933496_88933679	3.49489294	17.8055733	1.0134E-07	0.00056684	H3K27ac	BF
chr3	39342385	39342688	DEL	chr3_39584250_39584836	10.9995525	17.7788583	1.0254E-07	0.00057246	H3K27ac	BF
chr1	159111344	159111408	DEL	chr1_159418628_159419161	2.21039756	17.7197385	1.0525E-07	0.00057989	H3K27ac	BF

chr1	159363666	159363968	DEL	chr1_159418628_159419161	2.21039756	17.7197385	1.0525E-07	0.00057989	H3K27ac	BF
chr1	104001593	104001594	INS	chr1_103678372_103678619	6.52346144	17.7378304	1.0441E-07	0.00057989	H3K27ac	BF
chr1	159204093	159204094	INS	chr1_159418628_159419161	2.21039756	17.7197385	1.0525E-07	0.00057989	H3K27ac	BF
chr1	159297611	159297612	INS	chr1_159418628_159419161	2.21039756	17.7197385	1.0525E-07	0.00057989	H3K27ac	BF
chr1	159628709	159628710	INS	chr1_159418628_159419161	2.21039756	17.7197385	1.0525E-07	0.00057989	H3K27ac	BF
chr1	205959403	205959404	INS	chr1_205983779_205984045	3.64114238	17.7346393	1.0456E-07	0.00057989	H3K27ac	BF
chr3	55268565	55269399	DEL	chr3_55159387_55160661	3.28483456	17.6502393	1.0853E-07	0.00058277	H3K27ac	BF
chr3	55381566	55381715	DEL	chr3_55159387_55160661	3.28483456	17.6502393	1.0853E-07	0.00058277	H3K27ac	BF
chr3	54799284	54799285	INS	chr3_55159387_55160661	3.28483456	17.6502393	1.0853E-07	0.00058277	H3K27ac	BF
chr3	54915210	54915211	INS	chr3_55159387_55160661	3.28483456	17.6502393	1.0853E-07	0.00058277	H3K27ac	BF
chr3	55155937	55155938	INS	chr3_55159387_55160661	3.28483456	17.6502393	1.0853E-07	0.00058277	H3K27ac	BF
chr3	55161940	55161941	INS	chr3_55159387_55160661	3.28483456	17.6502393	1.0853E-07	0.00058277	H3K27ac	BF
chr4	110485626	110485627	INS	chr4_110793412_110793647	7.63988056	17.6522822	1.0844E-07	0.00058277	H3K27ac	BF

chr4	111094333	111094334	INS	chr4_110793412_110793647	7.63988056	17.6522822	1.0844E-07	0.00058277	H3K27ac	BF
chr4	111155923	111155924	INS	chr4_110793412_110793647	7.63988056	17.6522822	1.0844E-07	0.00058277	H3K27ac	BF
chr4	111158124	111158125	INS	chr4_110793412_110793647	7.63988056	17.6522822	1.0844E-07	0.00058277	H3K27ac	BF
chr1	8356436	8356437	INS	chr1_8562270_8562597	6.65752625	17.6786096	1.0718E-07	0.00058277	H3K27ac	BF
chr1	129061546	129061547	INS	chr1_129149666_129149942	-10.216882	-17.616077	1.1019E-07	0.00059059	H3K27ac	BF
chr3	68144528	68144998	DEL	chr3_68292584_68293407	8.99052806	17.607735	1.106E-07	0.00059064	H3K27ac	BF
chr3	68141059	68142597	INV	chr3_68292584_68293407	17.9810561	17.607735	1.106E-07	0.00059064	H3K27ac	BF
chr1	205959403	205959404	INS	chr1_206010132_206010396	6.98040869	17.5837735	1.1178E-07	0.00059588	H3K27ac	BF
chr1	6460049	6460050	INS	chr1_6812204_6813846	3.67421	17.5528677	1.1333E-07	0.00060196	H3K27ac	BF
chr1	6643056	6643057	INS	chr1_6812204_6813846	3.67421	17.5528677	1.1333E-07	0.00060196	H3K27ac	BF
chr1	33513968	33513969	INS	chr1_33946473_33947079	5.5387755	17.3956101	1.2159E-07	0.0006378	H3K27ac	BF
chr1	33893966	33893967	INS	chr1_33946473_33947079	5.5387755	17.3956101	1.2159E-07	0.0006378	H3K27ac	BF
chr1	33910107	33910108	INS	chr1_33946473_33947079	5.5387755	17.3956101	1.2159E-07	0.0006378	H3K27ac	BF

chr1	34395298	34395299	INS	chr1_33946473_33947079	5.5387755	17.3956101	1.2159E-07	0.0006378	H3K27ac	BF
chr1	205959403	205959404	INS	chr1_205912907_205913993	5.08457306	17.3787923	1.2251E-07	0.0006415	H3K27ac	BF
chr2	60887144	60889402	DEL	chr2_61128503_61129206	6.17657811	17.3621956	1.2343E-07	0.00064289	H3K27ac	BF
chr1	209501134	209505204	DEL	chr1_209223806_209224341	11.2455027	17.3494092	1.2414E-07	0.00064546	H3K27ac	BF
chr3	53936080	53936081	INS	chr3_54347845_54348037	4.21457872	17.3089772	1.2643E-07	0.00065274	H3K27ac	BF
chr3	54034033	54034034	INS	chr3_54347845_54348037	4.21457872	17.3089772	1.2643E-07	0.00065274	H3K27ac	BF
chr3	54284726	54284727	INS	chr3_54347845_54348037	4.21457872	17.3089772	1.2643E-07	0.00065274	H3K27ac	BF
chr3	54635397	54635398	INS	chr3_54347845_54348037	4.21457872	17.3089772	1.2643E-07	0.00065274	H3K27ac	BF
chr2	4023620	4023700	DEL	chr2_4296555_4296816	4.45753667	17.2624814	1.2911E-07	0.00065961	H3K27ac	BF
chr5	62540128	62540433	DEL	chr5_62601188_62601728	5.74592194	17.261343	1.2918E-07	0.00065961	H3K27ac	BF
chr4	103998094	103998095	INS	chr4_104469834_104470424	3.75885422	17.255081	1.2954E-07	0.00065961	H3K27ac	BF
chr4	104094809	104094810	INS	chr4_104469834_104470424	3.75885422	17.255081	1.2954E-07	0.00065961	H3K27ac	BF
chr4	104290539	104290540	INS	chr4_104469834_104470424	3.75885422	17.255081	1.2954E-07	0.00065961	H3K27ac	BF

chr4	104532089	104532090	INS	chr4_104469834_104470424	3.75885422	17.255081	1.2954E-07	0.00065961	H3K27ac	BF
chr5	70607550	70607551	INS	chr5_70173540_70174691	6.93040563	17.2471011	1.3001E-07	0.00066086	H3K27ac	BF
chr1	129061546	129061547	INS	chr1_128847177_128847395	-21.464276	-17.213696	1.32E-07	0.00066864	H3K27ac	BF
chr4	18227611	18227612	INS	chr4_18308959_18309310	5.71670361	17.2045933	1.3254E-07	0.00067026	H3K27ac	BF
chr1	205959403	205959404	INS	chr1_206136049_206136293	3.26280863	17.178271	1.3414E-07	0.00067716	H3K27ac	BF
chr1	7056618	7056619	INS	chr1_6782308_6783075	5.62295889	17.1695486	1.3467E-07	0.00067754	H3K27ac	BF
chr5	62540128	62540433	DEL	chr5_62051471_62052020	6.98037294	17.0966875	1.3922E-07	0.00069804	H3K27ac	BF
chr4	34580266	34581338	DEL	chr4_34324655_34325267	5.02898367	17.0817571	1.4017E-07	0.00069808	H3K27ac	BF
chr4	34582051	34582221	DEL	chr4_34324655_34325267	5.02898367	17.0817571	1.4017E-07	0.00069808	H3K27ac	BF
chr1	8356436	8356437	INS	chr1_8100755_8100923	5.174224	17.0916396	1.3954E-07	0.00069808	H3K27ac	BF
chr4	34565438	34565439	INS	chr4_34324655_34325267	5.02898367	17.0817571	1.4017E-07	0.00069808	H3K27ac	BF
chr5	73979409	73979468	DEL	chr5_73953932_73954397	8.17085813	16.9932214	1.4597E-07	0.00072575	H3K27ac	BF
chr2	87378442	87378532	DEL	chr2_87524390_87524797	3.8554825	16.9656013	1.4784E-07	0.00073133	H3K27ac	BF

chr2	87847374	87847375	INS	chr2_87524390_87524797	3.8554825	16.9656013	1.4784E-07	0.00073133	H3K27ac	BF
chr1	8356436	8356437	INS	chr1_8531406_8531898	3.93844956	16.971762	1.4742E-07	0.00073133	H3K27ac	BF
chr3	69924536	69924680	DEL	chr3_69980259_69980522	9.06207667	16.9121524	1.5153E-07	0.00073601	H3K27ac	BF
chr3	69609170	69609171	INS	chr3_69980259_69980522	9.06207667	16.9121524	1.5153E-07	0.00073601	H3K27ac	BF
chr3	69764216	69764217	INS	chr3_69980259_69980522	9.06207667	16.9121524	1.5153E-07	0.00073601	H3K27ac	BF
chr3	69734939	69734940	INS	chr3_69980259_69980522	9.06207667	16.9121524	1.5153E-07	0.00073601	H3K27ac	BF
chr3	69913224	69913225	INS	chr3_69980259_69980522	9.06207667	16.9121524	1.5153E-07	0.00073601	H3K27ac	BF
chr3	70189105	70189106	INS	chr3_69980259_69980522	9.06207667	16.9121524	1.5153E-07	0.00073601	H3K27ac	BF
chr3	70329262	70329263	INS	chr3_69980259_69980522	9.06207667	16.9121524	1.5153E-07	0.00073601	H3K27ac	BF
chr3	70318457	70318458	INS	chr3_69980259_69980522	9.06207667	16.9121524	1.5153E-07	0.00073601	H3K27ac	BF
chr1	188641399	188641400	INS	chr1_188560100_188560856	10.2572494	16.9466272	1.4914E-07	0.00073601	H3K27ac	BF
chr3	9073155	9073156	INS	chr3_9514868_9515224	2.95481033	16.9225277	1.508E-07	0.00073601	H3K27ac	BF
chr3	17187797	17187798	INS	chr3_17420192_17421151	9.73756722	16.9241976	1.5069E-07	0.00073601	H3K27ac	BF

chr2	6971934	6972779	DEL	chr2_7188132_7188789	23.2619567	16.8778674	1.5394E-07	0.00074166	H3K27ac	BF
chr2	6973113	6973278	DEL	chr2_7188132_7188789	23.2619567	16.8778674	1.5394E-07	0.00074166	H3K27ac	BF
chr2	7089639	7089857	DEL	chr2_7188132_7188789	23.2619567	16.8778674	1.5394E-07	0.00074166	H3K27ac	BF
chr2	7105946	7105947	INS	chr2_7188132_7188789	23.2619567	16.8778674	1.5394E-07	0.00074166	H3K27ac	BF
chr1	129061546	129061547	INS	chr1_129115404_129115799	-7.7198874	-16.883454	1.5355E-07	0.00074166	H3K27ac	BF
chr5	31001378	31001673	DEL	chr5_30582199_30582402	6.74691263	16.840402	1.5664E-07	0.00075296	H3K27ac	BF
chr1	129061546	129061547	INS	chr1_128774407_128775815	-10.813895	-16.83814	1.568E-07	0.00075296	H3K27ac	BF
chr4	71017558	71017841	DEL	chr4_71014830_71015378	2.28736156	16.672007	1.6941E-07	0.00081219	H3K27ac	BF
chr4	106250741	106250900	DEL	chr4_105872078_105872326	25.9337542	16.6036629	1.7492E-07	0.00082958	H3K27ac	BF
chr4	106254708	106254779	DEL	chr4_105872078_105872326	25.9337542	16.6036629	1.7492E-07	0.00082958	H3K27ac	BF
chr4	106253272	106253544	DEL	chr4_105872078_105872326	25.9337542	16.6036629	1.7492E-07	0.00082958	H3K27ac	BF
chr4	106049394	106049395	INS	chr4_105872078_105872326	25.9337542	16.6036629	1.7492E-07	0.00082958	H3K27ac	BF
chr4	106281804	106281805	INS	chr4_105872078_105872326	25.9337542	16.6036629	1.7492E-07	0.00082958	H3K27ac	BF

chr4	106257961	106257962	INS	chr4_105872078_105872326	25.9337542	16.6036629	1.7492E-07	0.00082958	H3K27ac	BF
chr3	55449949	55450016	DEL	chr3_55733417_55733734	4.31098	16.55819	1.7871E-07	0.00084135	H3K27ac	BF
chr1	17287767	17287945	DEL	chr1_17492252_17493234	12.0985989	16.5632984	1.7828E-07	0.00084135	H3K27ac	BF
chr3	55300034	55300035	INS	chr3_55733417_55733734	4.31098	16.55819	1.7871E-07	0.00084135	H3K27ac	BF
chr1	6460049	6460050	INS	chr1_6832915_6833153	7.62323025	16.5557313	1.7891E-07	0.00084135	H3K27ac	BF
chr1	6643056	6643057	INS	chr1_6832915_6833153	7.62323025	16.5557313	1.7891E-07	0.00084135	H3K27ac	BF
chr4	96057885	96057886	INS	chr4_95825917_95826450	34.7522722	16.4911037	1.8445E-07	0.00086465	H3K27ac	BF
chr4	96106669	96106670	INS	chr4_95825917_95826450	34.7522722	16.4911037	1.8445E-07	0.00086465	H3K27ac	BF
chr2	13122273	13123683	DEL	chr2_13166775_13167086	35.8013187	16.441215	1.8886E-07	0.0008839	H3K27ac	BF
chr1	144694030	144694320	DEL	chr1_145025738_145026007	5.05604299	16.3584798	1.9643E-07	0.00091211	H3K27ac	BF
chr1	144696466	144696517	DEL	chr1_145025738_145026007	5.05604299	16.3584798	1.9643E-07	0.00091211	H3K27ac	BF
chr1	144695107	144695391	DEL	chr1_145025738_145026007	5.05604299	16.3584798	1.9643E-07	0.00091211	H3K27ac	BF
chr1	144812554	144812555	INS	chr1_145025738_145026007	5.05604299	16.3584798	1.9643E-07	0.00091211	H3K27ac	BF

chr1	144855887	144855888	INS	chr1_145025738_145026007	5.05604299	16.3584798	1.9643E-07	0.00091211	H3K27ac	BF
chr5	62540128	62540433	DEL	chr5_62220372_62220662	4.1432975	16.2961718	2.0236E-07	0.0009137	H3K27ac	BF
chr1	108049450	108049630	DEL	chr1_107834989_107835701	5.75219894	16.3023153	2.0176E-07	0.0009137	H3K27ac	BF
chr5	8178977	8180239	DEL	chr5_7846175_7847272	19.0653243	16.3192966	2.0013E-07	0.0009137	H3K27ac	BF
chr5	8301274	8301364	DEL	chr5_7846175_7847272	19.0653243	16.3192966	2.0013E-07	0.0009137	H3K27ac	BF
chr1	254733161	254733240	DEL	chr1_255030270_255030746	5.47267406	16.3069915	2.0131E-07	0.0009137	H3K27ac	BF
chr1	108037908	108037909	INS	chr1_107834989_107835701	5.75219894	16.3023153	2.0176E-07	0.0009137	H3K27ac	BF
chr1	108035411	108035412	INS	chr1_107834989_107835701	5.75219894	16.3023153	2.0176E-07	0.0009137	H3K27ac	BF
chr5	7636706	7636707	INS	chr5_7846175_7847272	19.0653243	16.3192966	2.0013E-07	0.0009137	H3K27ac	BF
chr1	254888379	254888380	INS	chr1_255030270_255030746	5.47267406	16.3069915	2.0131E-07	0.0009137	H3K27ac	BF
chr1	255363534	255363535	INS	chr1_255030270_255030746	5.47267406	16.3069915	2.0131E-07	0.0009137	H3K27ac	BF
chr1	20183207	20183208	INS	chr1_20595700_20596024	16.7730763	16.2867736	2.0327E-07	0.0009137	H3K27ac	BF
chr1	20326834	20326835	INS	chr1_20595700_20596024	16.7730763	16.2867736	2.0327E-07	0.0009137	H3K27ac	BF

chr1	20468195	20468196	INS	chr1_20595700_20596024	16.7730763	16.2867736	2.0327E-07	0.0009137	H3K27ac	BF
chr5	33699385	33699386	INS	chr5_33810548_33811626	3.31319311	16.3126084	2.0077E-07	0.0009137	H3K27ac	BF
chr5	33691306	33691307	INS	chr5_33810548_33811626	3.31319311	16.3126084	2.0077E-07	0.0009137	H3K27ac	BF
chr5	33889049	33889050	INS	chr5_33810548_33811626	3.31319311	16.3126084	2.0077E-07	0.0009137	H3K27ac	BF
chr5	33887412	33887413	INS	chr5_33810548_33811626	3.31319311	16.3126084	2.0077E-07	0.0009137	H3K27ac	BF
chr5	34010981	34010982	INS	chr5_33810548_33811626	3.31319311	16.3126084	2.0077E-07	0.0009137	H3K27ac	BF
chr5	34024212	34024213	INS	chr5_33810548_33811626	3.31319311	16.3126084	2.0077E-07	0.0009137	H3K27ac	BF
chr5	34178708	34178709	INS	chr5_33810548_33811626	3.31319311	16.3126084	2.0077E-07	0.0009137	H3K27ac	BF
chr5	7581505	7581559	DEL	chr5_7573610_7574728	3.44244813	16.2785993	2.0407E-07	0.00091449	H3K27ac	BF
chr5	7579345	7579621	DEL	chr5_7573610_7574728	3.44244813	16.2785993	2.0407E-07	0.00091449	H3K27ac	BF
chr1	6460049	6460050	INS	chr1_6900874_6901080	3.7500795	16.2599651	2.0589E-07	0.0009199	H3K27ac	BF
chr1	6643056	6643057	INS	chr1_6900874_6901080	3.7500795	16.2599651	2.0589E-07	0.0009199	H3K27ac	BF
chr1	128326093	128326094	INS	chr1_128652842_128653709	5.89479444	16.2123975	2.1065E-07	0.00093547	H3K27ac	BF

chr1	128588065	128588066	INS	chr1_128652842_128653709	5.89479444	16.2123975	2.1065E-07	0.00093547	H3K27ac	BF
chr1	129032172	129032173	INS	chr1_128652842_128653709	5.89479444	16.2123975	2.1065E-07	0.00093547	H3K27ac	BF
chr1	129072436	129072437	INS	chr1_128652842_128653709	5.89479444	16.2123975	2.1065E-07	0.00093547	H3K27ac	BF
chr5	73979409	73979468	DEL	chr5_73598567_73599392	7.91764494	16.1855755	2.1338E-07	0.00094618	H3K27ac	BF
chr3	96511189	96511475	DEL	chr3_96666273_96666465	4.796667	16.1316083	2.19E-07	0.00095533	H3K27ac	BF
chr3	96525948	96526127	DEL	chr3_96666273_96666465	4.796667	16.1316083	2.19E-07	0.00095533	H3K27ac	BF
chr3	96711570	96711720	DEL	chr3_96666273_96666465	4.796667	16.1316083	2.19E-07	0.00095533	H3K27ac	BF
chr3	96716275	96716522	DEL	chr3_96666273_96666465	4.796667	16.1316083	2.19E-07	0.00095533	H3K27ac	BF
chr3	96714242	96714783	DEL	chr3_96666273_96666465	9.593334	16.1316083	2.19E-07	0.00095533	H3K27ac	BF
chr3	96721584	96721698	DEL	chr3_96666273_96666465	4.796667	16.1316083	2.19E-07	0.00095533	H3K27ac	BF
chr5	43766319	43766320	INS	chr5_44196072_44196331	13.1176475	16.1405537	2.1806E-07	0.00095533	H3K27ac	BF
chr5	44652564	44652565	INS	chr5_44196072_44196331	13.1176475	16.1405537	2.1806E-07	0.00095533	H3K27ac	BF
chr3	96181371	96181372	INS	chr3_96666273_96666465	4.796667	16.1316083	2.19E-07	0.00095533	H3K27ac	BF
chr3	96459393	96459394	INS	chr3_96666273_96666465	4.796667	16.1316083	2.19E-07	0.00095533	H3K27ac	BF
chr3	96690622	96690623	INS	chr3_96666273_96666465	4.796667	16.1316083	2.19E-07	0.00095533	H3K27ac	BF
chr5	62540128	62540433	DEL	chr5_62961321_62961843	4.25339125	16.1210012	2.2012E-07	0.00095882	H3K27ac	BF
chr2	64442080	64442080	BND	chr2_64620878_64621430	20.2120075	16.1051725	2.2181E-07	0.00096051	H3K27ac	BF

chr2	64442064	64442065	INS	chr2_64620878_64621430	20.2120075	16.1051725	2.2181E-07	0.00096051	H3K27ac	BF
chr1	205959403	205959404	INS	chr1_206268695_206269790	3.61601369	16.1130786	2.2097E-07	0.00096051	H3K27ac	BF
chr1	97157313	97157554	DEL	chr1_97346607_97347410	4.49274344	16.0878111	2.2368E-07	0.00096156	H3K27ac	BF
chr1	97377635	97377742	DEL	chr1_97346607_97347410	4.49274344	16.0878111	2.2368E-07	0.00096156	H3K27ac	BF
chr1	97132849	97132850	INS	chr1_97346607_97347410	4.49274344	16.0878111	2.2368E-07	0.00096156	H3K27ac	BF
chr1	97212360	97212361	INS	chr1_97346607_97347410	4.49274344	16.0878111	2.2368E-07	0.00096156	H3K27ac	BF
chr1	121518626	121518627	INS	chr1_121691839_121693372	5.49674544	16.0904252	2.234E-07	0.00096156	H3K27ac	BF
chr1	113331480	113331481	INS	chr1_113569584_113570337	43.7529458	16.0675829	2.2589E-07	0.00096961	H3K27ac	BF
chr4	107389870	107389871	INS	chr4_107472759_107473224	6.74213722	16.0444508	2.2843E-07	0.00097832	H3K27ac	BF
chr1	27373450	27373510	DEL	chr1_27727351_27727923	5.51525439	15.9665819	2.3725E-07	0.00100812	H3K27ac	BF
chr1	27392872	27392873	INS	chr1_27727351_27727923	5.51525439	15.9665819	2.3725E-07	0.00100812	H3K27ac	BF
chr1	27395063	27395064	INS	chr1_27727351_27727923	5.51525439	15.9665819	2.3725E-07	0.00100812	H3K27ac	BF
chr1	27832462	27832463	INS	chr1_27727351_27727923	5.51525439	15.9665819	2.3725E-07	0.00100812	H3K27ac	BF

chr1	27829816	27829817	INS	chr1_27727351_27727923	5.51525439	15.9665819	2.3725E-07	0.00100812	H3K27ac	BF
chr5	70607550	70607551	INS	chr5_70200936_70201300	6.77203319	15.9112152	2.4375E-07	0.00102834	H3K27ac	BF
chr1	8547604	8547605	INS	chr1_8809486_8810370	4.43435208	15.9118001	2.4368E-07	0.00102834	H3K27ac	BF
chr1	9055614	9055615	INS	chr1_8809486_8810370	4.43435208	15.9118001	2.4368E-07	0.00102834	H3K27ac	BF
chr1	9293106	9293107	INS	chr1_8809486_8810370	4.43435208	15.9118001	2.4368E-07	0.00102834	H3K27ac	BF
chr1	9307398	9307399	INS	chr1_8809486_8810370	4.43435208	15.9118001	2.4368E-07	0.00102834	H3K27ac	BF
chr1	104052039	104052552	DEL	chr1_103860730_103861992	10.7304625	15.8635938	2.495E-07	0.00104902	H3K27ac	BF
chr1	23192903	23194104	DEL	chr1_22696443_22697157	10.4838537	15.8618204	2.4971E-07	0.00104902	H3K27ac	BF
chr1	22795548	22795549	INS	chr1_22696443_22697157	10.4838537	15.8618204	2.4971E-07	0.00104902	H3K27ac	BF
chr1	20834452	20834453	INS	chr1_20958900_20959239	5.61453563	15.8298149	2.5367E-07	0.00106412	H3K27ac	BF
chr5	77356348	77356349	INS	chr5_77277990_77279676	6.80100611	15.8087525	2.5631E-07	0.00107063	H3K27ac	BF
chr5	77545855	77545856	INS	chr5_77277990_77279676	6.80100611	15.8087525	2.5631E-07	0.00107063	H3K27ac	BF
chr3	68144528	68144998	DEL	chr3_68137449_68137983	5.514122	15.7882269	2.5891E-07	0.00107845	H3K27ac	BF

chr3	68141059	68142597	INV	chr3_68137449_68137983	11.028244	15.7882269	2.5891E-07	0.00107845	H3K27ac	BF
chr1	37492757	37493579	DEL	chr1_37159980_37160664	6.03781861	15.7536251	2.6336E-07	0.00108932	H3K27ac	BF
chr2	48397735	48398192	DEL	chr2_48868176_48869733	11.08313	15.7558364	2.6308E-07	0.00108932	H3K27ac	BF
chr1	36712573	36712574	INS	chr1_37159980_37160664	6.03781861	15.7536251	2.6336E-07	0.00108932	H3K27ac	BF
chr1	36681746	36681747	INS	chr1_37159980_37160664	6.03781861	15.7536251	2.6336E-07	0.00108932	H3K27ac	BF
chr2	48399146	48399147	INS	chr2_48868176_48869733	11.08313	15.7558364	2.6308E-07	0.00108932	H3K27ac	BF
chr1	17287767	17287945	DEL	chr1_17553885_17554059	9.96262889	15.662559	2.7551E-07	0.00113795	H3K27ac	BF
chr1	8356436	8356437	INS	chr1_8194400_8194644	10.29719	15.6591474	2.7597E-07	0.00113828	H3K27ac	BF
chr1	106923248	106923249	INS	chr1_107361691_107361861	5.59239472	15.6276643	2.8032E-07	0.00115301	H3K27ac	BF
chr1	106956004	106956005	INS	chr1_107361691_107361861	5.59239472	15.6276643	2.8032E-07	0.00115301	H3K27ac	BF
chr1	8356436	8356437	INS	chr1_8446009_8448061	3.01290438	15.6028547	2.8381E-07	0.00116571	H3K27ac	BF
chr3	114296996	114297171	DEL	chr3_114178118_114178409	12.2984613	15.5787001	2.8724E-07	0.00116804	H3K27ac	BF
chr3	114290863	114291151	DEL	chr3_114178118_114178409	12.2984613	15.5787001	2.8724E-07	0.00116804	H3K27ac	BF

chr3	114499500	114499743	DEL	chr3_114178118_114178409	12.2984613	15.5787001	2.8724E-07	0.00116804	H3K27ac	BF
chr3	114598599	114598668	DEL	chr3_114178118_114178409	12.2984613	15.5787001	2.8724E-07	0.00116804	H3K27ac	BF
chr2	16281871	16282146	DEL	chr2_16304164_16304643	7.42222506	15.5629025	2.8952E-07	0.00116804	H3K27ac	BF
chr2	16376309	16376363	DEL	chr2_16304164_16304643	7.42222506	15.5629025	2.8952E-07	0.00116804	H3K27ac	BF
chr2	16576696	16576757	DEL	chr2_16304164_16304643	7.42222506	15.5629025	2.8952E-07	0.00116804	H3K27ac	BF
chr2	16680046	16680331	DEL	chr2_16304164_16304643	7.42222506	15.5629025	2.8952E-07	0.00116804	H3K27ac	BF
chr1	6460049	6460050	INS	chr1_6852208_6852383	17.952925	15.5792257	2.8717E-07	0.00116804	H3K27ac	BF
chr1	6643056	6643057	INS	chr1_6852208_6852383	17.952925	15.5792257	2.8717E-07	0.00116804	H3K27ac	BF
chr2	15833138	15833139	INS	chr2_16304164_16304643	7.42222506	15.5629025	2.8952E-07	0.00116804	H3K27ac	BF
chr2	16104627	16104628	INS	chr2_16304164_16304643	7.42222506	15.5629025	2.8952E-07	0.00116804	H3K27ac	BF
chr2	16406615	16406616	INS	chr2_16304164_16304643	7.42222506	15.5629025	2.8952E-07	0.00116804	H3K27ac	BF
chr4	38643842	38644096	DEL	chr4_39022489_39024607	6.39921222	15.546651	2.9188E-07	0.00116958	H3K27ac	BF
chr4	38666874	38666982	DEL	chr4_39022489_39024607	6.39921222	15.546651	2.9188E-07	0.00116958	H3K27ac	BF

chr4	38595995	38595996	INS	chr4_39022489_39024607	6.39921222	15.546651	2.9188E-07	0.00116958	H3K27ac	BF
chr4	39302295	39302296	INS	chr4_39022489_39024607	6.39921222	15.546651	2.9188E-07	0.00116958	H3K27ac	BF
chr1	8336791	8336792	INS	chr1_8589040_8589449	3.74408389	15.534947	2.9359E-07	0.00117167	H3K27ac	BF
chr1	8426315	8426316	INS	chr1_8589040_8589449	3.74408389	15.534947	2.9359E-07	0.00117167	H3K27ac	BF
chr1	8455438	8455439	INS	chr1_8589040_8589449	3.74408389	15.534947	2.9359E-07	0.00117167	H3K27ac	BF
chr5	44101704	44101705	INS	chr5_44034864_44035243	11.9523587	15.525859	2.9493E-07	0.00117384	H3K27ac	BF
chr5	44155068	44155069	INS	chr5_44034864_44035243	11.9523587	15.525859	2.9493E-07	0.00117384	H3K27ac	BF
chr5	7485632	7486913	DEL	chr5_7842240_7842587	8.94865507	15.5041362	2.9815E-07	0.00117732	H3K27ac	BF
chr5	7486932	7487883	DEL	chr5_7842240_7842587	8.94865507	15.5041362	2.9815E-07	0.00117732	H3K27ac	BF
chr5	7679240	7679241	INS	chr5_7842240_7842587	8.94865507	15.5041362	2.9815E-07	0.00117732	H3K27ac	BF
chr1	11626419	11626420	INS	chr1_11952140_11952668	4.39074594	15.5038586	2.9819E-07	0.00117732	H3K27ac	BF
chr1	8356436	8356437	INS	chr1_7967489_7967756	3.01859381	15.4973057	2.9917E-07	0.00117961	H3K27ac	BF
chr2	13122273	13123683	DEL	chr2_13148650_13148901	7.16009143	15.480826	3.0166E-07	0.00118782	H3K27ac	BF

chr1	129061546	129061547	INS	chr1_129131372_129131616	-8.4187303	-15.467956	3.0361E-07	0.00119391	H3K27ac	BF
chr1	26088493	26088670	DEL	chr1_26465749_26466242	4.830755	15.4469006	3.0684E-07	0.00120182	H3K27ac	BF
chr1	26539658	26539718	DEL	chr1_26465749_26466242	4.830755	15.4469006	3.0684E-07	0.00120182	H3K27ac	BF
chr1	6460049	6460050	INS	chr1_6829823_6830451	7.31295888	15.4056837	3.1327E-07	0.00122377	H3K27ac	BF
chr1	6643056	6643057	INS	chr1_6829823_6830451	7.31295888	15.4056837	3.1327E-07	0.00122377	H3K27ac	BF
chr1	205959403	205959404	INS	chr1_206191416_206192791	2.691011	15.4021743	3.1383E-07	0.00122432	H3K27ac	BF
chr4	61776445	61776499	DEL	chr4_61949506_61951713	13.0477872	15.396697	3.147E-07	0.00122608	H3K27ac	BF
chr1	111875980	111875981	INS	chr1_111414820_111415428	3.66010289	15.3758213	3.1803E-07	0.00123744	H3K27ac	BF
chr2	116712319	116712320	INS	chr2_116619444_116620186	8.67086778	15.3599069	3.206E-07	0.00124415	H3K27ac	BF
chr2	116856506	116856507	INS	chr2_116619444_116620186	8.67086778	15.3599069	3.206E-07	0.00124415	H3K27ac	BF
chr1	20834452	20834453	INS	chr1_20898345_20898990	7.38842931	15.3525634	3.2179E-07	0.00124714	H3K27ac	BF
chr4	103530187	103530358	DEL	chr4_103083356_103083695	8.15586144	15.3446177	3.2308E-07	0.00125052	H3K27ac	BF
chr5	7581505	7581559	DEL	chr5_7566340_7566737	3.02024388	15.3279051	3.2583E-07	0.0012562	H3K27ac	BF

chr5	7579345	7579621	DEL	chr5_7566340_7566737	3.02024388	15.3279051	3.2583E-07	0.0012562	H3K27ac	BF
chr2	13122273	13123683	DEL	chr2_13370980_13371500	10.829478	15.3289315	3.2566E-07	0.0012562	H3K27ac	BF
chr1	21471187	21471247	DEL	chr1_21708214_21710215	9.5324245	15.3148353	3.2799E-07	0.0012629	H3K27ac	BF
chr5	7581505	7581559	DEL	chr5_7498143_7499104	2.84248919	15.2922532	3.3177E-07	0.00127413	H3K27ac	BF
chr5	7579345	7579621	DEL	chr5_7498143_7499104	2.84248919	15.2922532	3.3177E-07	0.00127413	H3K27ac	BF
chr4	38643842	38644096	DEL	chr4_39029426_39029716	4.29840283	15.2248219	3.4335E-07	0.00129999	H3K27ac	BF
chr4	38666874	38666982	DEL	chr4_39029426_39029716	4.29840283	15.2248219	3.4335E-07	0.00129999	H3K27ac	BF
chr1	86136311	86136312	INS	chr1_86109920_86110223	7.09524861	15.2248466	3.4334E-07	0.00129999	H3K27ac	BF
chr1	86279573	86279574	INS	chr1_86109920_86110223	7.09524861	15.2248466	3.4334E-07	0.00129999	H3K27ac	BF
chr1	86508701	86508702	INS	chr1_86109920_86110223	7.09524861	15.2248466	3.4334E-07	0.00129999	H3K27ac	BF
chr1	86606023	86606024	INS	chr1_86109920_86110223	7.09524861	15.2248466	3.4334E-07	0.00129999	H3K27ac	BF
chr4	38595995	38595996	INS	chr4_39029426_39029716	4.29840283	15.2248219	3.4335E-07	0.00129999	H3K27ac	BF
chr4	39302295	39302296	INS	chr4_39029426_39029716	4.29840283	15.2248219	3.4335E-07	0.00129999	H3K27ac	BF

chr5	62540128	62540433	DEL	chr5_62866386_62867610	8.09890925	15.2138971	3.4527E-07	0.00130557	H3K27ac	BF
chr2	13122273	13123683	DEL	chr2_13125597_13125762	46.0868	15.2032903	3.4714E-07	0.00131098	H3K27ac	BF
chr1	119077652	119077653	INS	chr1_119003076_119003771	7.74708783	15.1438704	3.5785E-07	0.00134797	H3K27ac	BF
chr1	119166234	119166235	INS	chr1_119003076_119003771	7.74708783	15.1438704	3.5785E-07	0.00134797	H3K27ac	BF
chr1	79767443	79767752	DEL	chr1_79424969_79426158	15.751175	15.1126008	3.6363E-07	0.00136627	H3K27ac	BF
chr1	79156622	79156623	INS	chr1_79424969_79426158	15.751175	15.1126008	3.6363E-07	0.00136627	H3K27ac	BF
chr3	68144528	68144998	DEL	chr3_68276429_68277461	3.57647288	15.0993233	3.6612E-07	0.00137213	H3K27ac	BF
chr3	68141059	68142597	INV	chr3_68276429_68277461	7.15294575	15.0993233	3.6612E-07	0.00137213	H3K27ac	BF
chr4	71413024	71413025	INS	chr4_71895768_71897536	15.6904706	15.0670573	3.7224E-07	0.00139155	H3K27ac	BF
chr4	71809050	71809051	INS	chr4_71895768_71897536	15.6904706	15.0670573	3.7224E-07	0.00139155	H3K27ac	BF
chr1	104052039	104052552	DEL	chr1_104465072_104466635	11.0188677	15.0324158	3.7895E-07	0.00141356	H3K27ac	BF
chr1	6460049	6460050	INS	chr1_6793244_6794156	3.19791056	15.0292388	3.7957E-07	0.00141356	H3K27ac	BF
chr1	6643056	6643057	INS	chr1_6793244_6794156	3.19791056	15.0292388	3.7957E-07	0.00141356	H3K27ac	BF

chr5	62540128	62540433	DEL	chr5_62167526_62168325	2.71770563	14.9767119	3.9001E-07	0.00145063	H3K27ac	BF
chr2	13122273	13123683	DEL	chr2_13431466_13432187	10.8181997	14.9674333	3.9189E-07	0.00145578	H3K27ac	BF
chr3	113573874	113573875	INS	chr3_113715285_113715898	16.7820854	14.9527753	3.9488E-07	0.00146504	H3K27ac	BF
chr4	15488020	15488021	INS	chr4_15271331_15271874	5.26595944	14.9312251	3.9932E-07	0.00147965	H3K27ac	BF
chr1	27832462	27832463	INS	chr1_28037512_28038061	10.7124769	14.9238643	4.0085E-07	0.00148159	H3K27ac	BF
chr1	27829816	27829817	INS	chr1_28037512_28038061	10.7124769	14.9238643	4.0085E-07	0.00148159	H3K27ac	BF
chr5	67816692	67816693	INS	chr5_67388081_67388547	6.74797611	14.9117816	4.0337E-07	0.00148906	H3K27ac	BF
chr2	4023620	4023700	DEL	chr2_3860219_3860491	3.24037017	14.8913702	4.0768E-07	0.00149746	H3K27ac	BF
chr2	3399845	3399897	DEL	chr2_3860219_3860491	3.24037017	14.8913702	4.0768E-07	0.00149746	H3K27ac	BF
chr2	13122273	13123683	DEL	chr2_13378913_13379238	18.1089567	14.8382942	4.1911E-07	0.00153375	H3K27ac	BF
chr2	103594433	103594434	INS	chr2_103624208_103624817	27.0604944	14.8399623	4.1875E-07	0.00153375	H3K27ac	BF
chr2	103590216	103590217	INS	chr2_103624208_103624817	27.0604944	14.8399623	4.1875E-07	0.00153375	H3K27ac	BF
chr1	169811358	169811510	DEL	chr1_169615553_169616299	5.64034283	14.8273182	4.2152E-07	0.00153496	H3K27ac	BF

chr1	169824231	169824532	DEL	chr1_169615553_169616299	5.64034283	14.8273182	4.2152E-07	0.00153496	H3K27ac	BF
chr1	169267306	169267307	INS	chr1_169615553_169616299	5.64034283	14.8273182	4.2152E-07	0.00153496	H3K27ac	BF
chr1	169593891	169593892	INS	chr1_169615553_169616299	5.64034283	14.8273182	4.2152E-07	0.00153496	H3K27ac	BF
chr1	240699894	240699895	INS	chr1_241021332_241021921	5.64293622	14.8214119	4.2282E-07	0.0015378	H3K27ac	BF
chr4	61982070	61982071	INS	chr4_62327163_62327480	13.6530477	14.8065911	4.2611E-07	0.00154406	H3K27ac	BF
chr4	61978257	61978258	INS	chr4_62327163_62327480	13.6530477	14.8065911	4.2611E-07	0.00154406	H3K27ac	BF
chr4	62624794	62624795	INS	chr4_62327163_62327480	13.6530477	14.8065911	4.2611E-07	0.00154406	H3K27ac	BF
chr2	126596215	126596496	DEL	chr2_126983811_126984222	2.70474706	14.801739	4.272E-07	0.00154609	H3K27ac	BF
chr5	73979409	73979468	DEL	chr5_73919939_73920172	3.15116638	14.7625799	4.3605E-07	0.00156491	H3K27ac	BF
chr4	102884126	102885737	DEL	chr4_103083356_103083695	8.14499581	14.756837	4.3737E-07	0.00156491	H3K27ac	BF
chr1	37492757	37493579	DEL	chr1_37880004_37880180	14.8127689	14.7659085	4.3529E-07	0.00156491	H3K27ac	BF
chr4	116597067	116597068	INS	chr4_116845806_116847261	9.82232111	14.7583026	4.3703E-07	0.00156491	H3K27ac	BF
chr4	116604576	116604577	INS	chr4_116845806_116847261	9.82232111	14.7583026	4.3703E-07	0.00156491	H3K27ac	BF

chr1	129061546	129061547	INS	chr1_128984505_128985161	-5.3337505	-14.755415	4.377E-07	0.00156491	H3K27ac	BF
chr1	37718951	37718952	INS	chr1_37880004_37880180	14.8127689	14.7659085	4.3529E-07	0.00156491	H3K27ac	BF
chr1	38023171	38023172	INS	chr1_37880004_37880180	14.8127689	14.7659085	4.3529E-07	0.00156491	H3K27ac	BF
chr1	38125169	38125170	INS	chr1_37880004_37880180	14.8127689	14.7659085	4.3529E-07	0.00156491	H3K27ac	BF
chr1	38094609	38094610	INS	chr1_37880004_37880180	14.8127689	14.7659085	4.3529E-07	0.00156491	H3K27ac	BF
chr2	69705683	69705684	INS	chr2_70176236_70176655	3.60146111	14.7142274	4.4727E-07	0.00159363	H3K27ac	BF
chr4	19914280	19914281	INS	chr4_20412424_20412945	5.20782083	14.7139101	4.4735E-07	0.00159363	H3K27ac	BF
chr4	20751112	20751113	INS	chr4_20412424_20412945	5.20782083	14.7139101	4.4735E-07	0.00159363	H3K27ac	BF
chr1	1511558	1511813	DEL	chr1_1823136_1823431	11.349181	14.690444	4.5291E-07	0.0016057	H3K27ac	BF
chr1	1697599	1697690	DEL	chr1_1823136_1823431	11.349181	14.690444	4.5291E-07	0.0016057	H3K27ac	BF
chr1	2307186	2307261	DEL	chr1_1823136_1823431	11.349181	14.690444	4.5291E-07	0.0016057	H3K27ac	BF
chr1	1779044	1779045	INS	chr1_1823136_1823431	11.349181	14.690444	4.5291E-07	0.0016057	H3K27ac	BF
chr3	68144528	68144998	DEL	chr3_68167479_68168122	5.87209438	14.6618419	4.598E-07	0.0016195	H3K27ac	BF

chr5	7485632	7486913	DEL	chr5_7589153_7589723	6.42835778	14.6606257	4.6009E-07	0.0016195	H3K27ac	BF
chr5	7486932	7487883	DEL	chr5_7589153_7589723	6.42835778	14.6606257	4.6009E-07	0.0016195	H3K27ac	BF
chr5	7679240	7679241	INS	chr5_7589153_7589723	6.42835778	14.6606257	4.6009E-07	0.0016195	H3K27ac	BF
chr3	68141059	68142597	INV	chr3_68167479_68168122	11.7441888	14.6618419	4.598E-07	0.0016195	H3K27ac	BF
chr1	221143081	221143082	INS	chr1_221009648_221010042	5.31445611	14.6315901	4.6721E-07	0.00163868	H3K27ac	BF
chr1	221269364	221269365	INS	chr1_221009648_221010042	5.31445611	14.6315901	4.6721E-07	0.00163868	H3K27ac	BF
chr1	221358635	221358636	INS	chr1_221009648_221010042	5.31445611	14.6315901	4.6721E-07	0.00163868	H3K27ac	BF
chr1	218661436	218662998	DEL	chr1_219093672_219094498	4.7621935	14.6136625	4.7166E-07	0.00164843	H3K27ac	BF
chr1	218904154	218904461	DEL	chr1_219093672_219094498	4.7621935	14.6136625	4.7166E-07	0.00164843	H3K27ac	BF
chr1	218904946	218904947	INS	chr1_219093672_219094498	4.7621935	14.6136625	4.7166E-07	0.00164843	H3K27ac	BF
chr1	228937749	228938016	DEL	chr1_229038086_229039061	4.15416422	14.5293444	4.9326E-07	0.0017158	H3K27ac	BF
chr1	228953944	228954011	DEL	chr1_229038086_229039061	4.15416422	14.5293444	4.9326E-07	0.0017158	H3K27ac	BF
chr1	229398592	229398593	INS	chr1_229038086_229039061	4.15416422	14.5293444	4.9326E-07	0.0017158	H3K27ac	BF

chr1	229396062	229396063	INS	chr1_229038086_229039061	4.15416422	14.5293444	4.9326E-07	0.0017158	H3K27ac	BF
chr2	13122273	13123683	DEL	chr2_13121320_13121741	32.497117	14.5215145	4.9532E-07	0.00172095	H3K27ac	BF
chr1	33121969	33122174	DEL	chr1_33304785_33305645	4.98749228	14.5008046	5.0082E-07	0.0017319	H3K27ac	BF
chr1	33122896	33123201	DEL	chr1_33304785_33305645	4.98749228	14.5008046	5.0082E-07	0.0017319	H3K27ac	BF
chr1	33083035	33083036	INS	chr1_33304785_33305645	4.98749228	14.5008046	5.0082E-07	0.0017319	H3K27ac	BF
chr1	33513968	33513969	INS	chr1_33304785_33305645	4.98749228	14.5008046	5.0082E-07	0.0017319	H3K27ac	BF
chr2	13122273	13123683	DEL	chr2_13379425_13379659	17.1693091	14.4917758	5.0324E-07	0.00173823	H3K27ac	BF
chr3	53202776	53202837	DEL	chr3_52830583_52830996	6.90731132	14.4683683	5.0957E-07	0.00175191	H3K27ac	BF
chr3	53192935	53192936	INS	chr3_52830583_52830996	6.90731132	14.4683683	5.0957E-07	0.00175191	H3K27ac	BF
chr3	53196048	53196049	INS	chr3_52830583_52830996	6.90731132	14.4683683	5.0957E-07	0.00175191	H3K27ac	BF
chr1	20834452	20834453	INS	chr1_20981229_20983015	-4.1984544	-14.469479	5.0927E-07	0.00175191	H3K27ac	BF
chr2	6971934	6972779	DEL	chr2_7115661_7115979	4.68972861	14.4314594	5.1974E-07	0.00177858	H3K27ac	BF
chr2	6973113	6973278	DEL	chr2_7115661_7115979	4.68972861	14.4314594	5.1974E-07	0.00177858	H3K27ac	BF

chr2	7089639	7089857	DEL	chr2_7115661_7115979	4.68972861	14.4314594	5.1974E-07	0.00177858	H3K27ac	BF
chr2	7105946	7105947	INS	chr2_7115661_7115979	4.68972861	14.4314594	5.1974E-07	0.00177858	H3K27ac	BF
chr1	20183207	20183208	INS	chr1_20591068_20591234	9.64159771	14.4191834	5.2317E-07	0.00178001	H3K27ac	BF
chr1	20326834	20326835	INS	chr1_20591068_20591234	9.64159771	14.4191834	5.2317E-07	0.00178001	H3K27ac	BF
chr1	20468195	20468196	INS	chr1_20591068_20591234	9.64159771	14.4191834	5.2317E-07	0.00178001	H3K27ac	BF
chr1	20834452	20834453	INS	chr1_20938401_20938646	4.08679813	14.4211983	5.2261E-07	0.00178001	H3K27ac	BF
chr5	73979409	73979468	DEL	chr5_74143235_74143818	2.35749756	14.4036602	5.2755E-07	0.00178814	H3K27ac	BF
chr3	93195340	93195413	DEL	chr3_93001209_93002221	6.30072	14.3999845	5.2859E-07	0.00178814	H3K27ac	BF
chr3	93423836	93423892	DEL	chr3_93001209_93002221	6.30072	14.3999845	5.2859E-07	0.00178814	H3K27ac	BF
chr1	201384714	201385082	DEL	chr1_200909012_200909731	4.54638195	14.407054	5.2659E-07	0.00178814	H3K27ac	BF
chr3	92761333	92761334	INS	chr3_93001209_93002221	6.30072	14.3999845	5.2859E-07	0.00178814	H3K27ac	BF
chr4	107389870	107389871	INS	chr4_107781333_107781566	9.47069097	14.3960235	5.2972E-07	0.0017899	H3K27ac	BF
chr2	132195622	132195703	DEL	chr2_131843521_131843826	8.21529611	14.379453	5.3445E-07	0.00180179	H3K27ac	BF

chr2	132071320	132071321	INS	chr2_131843521_131843826	8.21529611	14.379453	5.3445E-07	0.00180179	H3K27ac	BF
chr3	102554990	102555055	DEL	chr3_102172219_102174201	6.69549556	14.3411529	5.4559E-07	0.00183723	H3K27ac	BF
chr1	77125236	77125237	INS	chr1_77363230_77363660	7.52637375	14.3326166	5.481E-07	0.0018415	H3K27ac	BF
chr1	77786939	77786940	INS	chr1_77363230_77363660	7.52637375	14.3326166	5.481E-07	0.0018415	H3K27ac	BF
chr2	13122273	13123683	DEL	chr2_13498181_13499692	13.2416014	14.3052402	5.5626E-07	0.00186389	H3K27ac	BF
chr5	7636706	7636707	INS	chr5_7570543_7570845	3.40115456	14.3079927	5.5544E-07	0.00186389	H3K27ac	BF
chr1	268866273	268866274	INS	chr1_268601449_268601619	8.64831625	14.3039191	5.5666E-07	0.00186389	H3K27ac	BF
chr2	114530061	114532178	DEL	chr2_114365559_114366419	2.90283506	14.2888797	5.6121E-07	0.00187486	H3K27ac	BF
chr2	114533753	114533754	INS	chr2_114365559_114366419	2.90283506	14.2888797	5.6121E-07	0.00187486	H3K27ac	BF
chr1	270587310	270587487	DEL	chr1_270800792_270801972	18.3216194	14.2728989	5.6608E-07	0.00188687	H3K27ac	BF
chr1	271070524	271070525	INS	chr1_270800792_270801972	18.3216194	14.2728989	5.6608E-07	0.00188687	H3K27ac	BF
chr3	29069145	29069396	DEL	chr3_28927121_28927705	8.22910411	14.240488	5.7611E-07	0.00190953	H3K27ac	BF
chr3	29103576	29104180	DEL	chr3_28927121_28927705	8.22910411	14.240488	5.7611E-07	0.00190953	H3K27ac	BF

chr3	29104564	29107552	DEL	chr3_28927121_28927705	8.22910411	14.240488	5.7611E-07	0.00190953	H3K27ac	BF
chr3	28900623	28900624	INS	chr3_28927121_28927705	8.22910411	14.240488	5.7611E-07	0.00190953	H3K27ac	BF
chr3	28843114	28843115	INS	chr3_28927121_28927705	8.22910411	14.240488	5.7611E-07	0.00190953	H3K27ac	BF
chr3	68144528	68144998	DEL	chr3_68175780_68178037	4.08868625	14.2351456	5.7779E-07	0.00191079	H3K27ac	BF
chr3	68141059	68142597	INV	chr3_68175780_68178037	8.1773725	14.2351456	5.7779E-07	0.00191079	H3K27ac	BF
chr1	188108680	188109925	DEL	chr1_188050736_188051034	5.93829106	14.2108692	5.8545E-07	0.00193183	H3K27ac	BF
chr1	187775529	187775530	INS	chr1_188050736_188051034	5.93829106	14.2108692	5.8545E-07	0.00193183	H3K27ac	BF
chr2	13122273	13123683	DEL	chr2_13152222_13152408	29.2216491	14.1815452	5.9487E-07	0.00196071	H3K27ac	BF
chr2	48297275	48297336	DEL	chr2_48611190_48611461	3.41681375	14.1672706	5.9952E-07	0.00197382	H3K27ac	BF
chr1	11891912	11892497	DEL	chr1_11761921_11762147	10.5579903	14.1507013	6.0496E-07	0.00197974	H3K27ac	BF
chr5	19305696	19305895	DEL	chr5_18822390_18823217	5.14316572	14.1590423	6.0221E-07	0.00197974	H3K27ac	BF
chr1	86136311	86136312	INS	chr1_85915690_85916006	8.11042771	14.1495589	6.0534E-07	0.00197974	H3K27ac	BF
chr1	86279573	86279574	INS	chr1_85915690_85916006	8.11042771	14.1495589	6.0534E-07	0.00197974	H3K27ac	BF

chr5	31001378	31001673	DEL	chr5_30601003_30601780	6.59982838	14.1171696	6.1615E-07	0.00201286	H3K27ac	BF
chr1	108733962	108734028	DEL	chr1_108780123_108781090	3.84556111	14.1041841	6.2054E-07	0.00202497	H3K27ac	BF
chr1	26882689	26882987	DEL	chr1_26465749_26466242	4.84493063	14.0837215	6.2754E-07	0.00204554	H3K27ac	BF
chr4	112216586	112216758	DEL	chr4_112709616_112709904	5.03344456	14.0678367	6.3303E-07	0.00204986	H3K27ac	BF
chr4	112447773	112447774	INS	chr4_112709616_112709904	5.03344456	14.0678367	6.3303E-07	0.00204986	H3K27ac	BF
chr4	112779625	112779626	INS	chr4_112709616_112709904	5.03344456	14.0678367	6.3303E-07	0.00204986	H3K27ac	BF
chr4	112829187	112829188	INS	chr4_112709616_112709904	5.03344456	14.0678367	6.3303E-07	0.00204986	H3K27ac	BF
chr1	129061546	129061547	INS	chr1_129437130_129437383	-13.198535	-14.068731	6.3272E-07	0.00204986	H3K27ac	BF
chr2	129199779	129199780	INS	chr2_129322830_129323009	4.38712106	14.0608214	6.3547E-07	0.00205552	H3K27ac	BF
chr5	73979409	73979468	DEL	chr5_74156077_74157835	2.62070063	14.0267645	6.4748E-07	0.00208978	H3K27ac	BF
chr3	50906019	50906020	INS	chr3_51365880_51366206	5.77979044	14.027046	6.4738E-07	0.00208978	H3K27ac	BF
chr1	205959403	205959404	INS	chr1_206139743_206140339	3.39794619	14.0222184	6.491E-07	0.00209273	H3K27ac	BF
chr1	201822017	201822018	INS	chr1_201707408_201707745	9.17114917	14.0117539	6.5285E-07	0.00210253	H3K27ac	BF

chr3	88586939	88587058	DEL	chr3_88517148_88518266	2.71063622	13.9903567	6.606E-07	0.00212053	H3K27ac	BF
chr3	88253606	88253607	INS	chr3_88517148_88518266	2.71063622	13.9903567	6.606E-07	0.00212053	H3K27ac	BF
chr3	88285211	88285212	INS	chr3_88517148_88518266	2.71063622	13.9903567	6.606E-07	0.00212053	H3K27ac	BF
chr3	55268565	55269399	DEL	chr3_55704423_55705557	2.53517333	13.9684396	6.6864E-07	0.00213705	H3K27ac	BF
chr3	55381566	55381715	DEL	chr3_55704423_55705557	2.53517333	13.9684396	6.6864E-07	0.00213705	H3K27ac	BF
chr5	31001378	31001673	DEL	chr5_30593149_30593427	5.19597106	13.9731063	6.6691E-07	0.00213705	H3K27ac	BF
chr3	56133996	56133997	INS	chr3_55704423_55705557	2.53517333	13.9684396	6.6864E-07	0.00213705	H3K27ac	BF
chr2	136461573	136461574	INS	chr2_136360148_136361296	2.21341244	13.9581693	6.7244E-07	0.00213902	H3K27ac	BF
chr2	136621710	136621711	INS	chr2_136360148_136361296	2.21341244	13.9581693	6.7244E-07	0.00213902	H3K27ac	BF
chr1	14854631	14854632	INS	chr1_15322320_15322557	5.40108922	13.9531239	6.7432E-07	0.00213902	H3K27ac	BF
chr1	14957571	14957572	INS	chr1_15322320_15322557	5.40108922	13.9531239	6.7432E-07	0.00213902	H3K27ac	BF
chr1	15227869	15227870	INS	chr1_15322320_15322557	5.40108922	13.9531239	6.7432E-07	0.00213902	H3K27ac	BF
chr1	15287464	15287465	INS	chr1_15322320_15322557	5.40108922	13.9531239	6.7432E-07	0.00213902	H3K27ac	BF

chr1	15289209	15289210	INS	chr1_15322320_15322557	5.40108922	13.9531239	6.7432E-07	0.00213902	H3K27ac	BF
chr2	48397735	48398192	DEL	chr2_48833146_48833956	4.06963321	13.9441953	6.7766E-07	0.002145	H3K27ac	BF
chr2	48399146	48399147	INS	chr2_48833146_48833956	4.06963321	13.9441953	6.7766E-07	0.002145	H3K27ac	BF
chr1	190223373	190223374	INS	chr1_190460755_190461356	3.56684794	13.9362757	6.8063E-07	0.00215067	H3K27ac	BF
chr1	190223373	190223374	INS	chr1_190651269_190651534	4.42186563	13.9355579	6.8091E-07	0.00215067	H3K27ac	BF
chr4	1354038	1355196	DEL	chr4_1775020_1775257	4.86977789	13.9219282	6.8607E-07	0.00216465	H3K27ac	BF
chr4	68096511	68096512	INS	chr4_68422454_68423250	7.33178368	13.9079207	6.9141E-07	0.00217919	H3K27ac	BF
chr1	116096314	116096445	DEL	chr1_115930079_115932782	3.52212111	13.8980464	6.9521E-07	0.0021865	H3K27ac	BF
chr1	115974133	115974134	INS	chr1_115930079_115932782	3.52212111	13.8980464	6.9521E-07	0.0021865	H3K27ac	BF
chr2	80662404	80662616	DEL	chr2_80988218_80988640	8.26540944	13.8603149	7.0994E-07	0.00222684	H3K27ac	BF
chr2	81219789	81219866	DEL	chr2_80988218_80988640	8.26540944	13.8603149	7.0994E-07	0.00222684	H3K27ac	BF
chr1	6643056	6643057	INS	chr1_7105913_7107527	8.98935188	13.8594113	7.103E-07	0.00222684	H3K27ac	BF
chr1	104052039	104052552	DEL	chr1_104451718_104452146	6.39256063	13.83907	7.1839E-07	0.00224981	H3K27ac	BF

chr2	85776209	85776607	DEL	chr2_85980446_85982052	2.29611413	13.8274646	7.2305E-07	0.00225962	H3K27ac	BF
chr2	85694887	85694888	INS	chr2_85980446_85982052	2.29611413	13.8274646	7.2305E-07	0.00225962	H3K27ac	BF
chr1	7746518	7747818	DEL	chr1_8165473_8166074	9.68852111	13.7955549	7.3605E-07	0.00228813	H3K27ac	BF
chr1	8336791	8336792	INS	chr1_8165473_8166074	9.68852111	13.7955549	7.3605E-07	0.00228813	H3K27ac	BF
chr1	8426315	8426316	INS	chr1_8165473_8166074	9.68852111	13.7955549	7.3605E-07	0.00228813	H3K27ac	BF
chr1	8455438	8455439	INS	chr1_8165473_8166074	9.68852111	13.7955549	7.3605E-07	0.00228813	H3K27ac	BF
chr1	190223373	190223374	INS	chr1_190311228_190311818	4.28450531	13.7961393	7.3581E-07	0.00228813	H3K27ac	BF
chr3	17369660	17369661	INS	chr3_17692851_17693658	3.96181478	13.792118	7.3746E-07	0.00229012	H3K27ac	BF
chr4	103530187	103530358	DEL	chr4_103178412_103179963	5.708805	13.7839732	7.4083E-07	0.00229815	H3K27ac	BF
chr2	83200498	83200570	DEL	chr2_82878303_82878920	5.29791083	13.7592907	7.5113E-07	0.00232037	H3K27ac	BF
chr2	83198806	83199203	DEL	chr2_82878303_82878920	5.29791083	13.7592907	7.5113E-07	0.00232037	H3K27ac	BF
chr2	82918749	82918750	INS	chr2_82878303_82878920	5.29791083	13.7592907	7.5113E-07	0.00232037	H3K27ac	BF
chr2	83067882	83067883	INS	chr2_82878303_82878920	5.29791083	13.7592907	7.5113E-07	0.00232037	H3K27ac	BF

chr3	76926501	76926709	DEL	chr3_76566933_76567570	3.1331015	13.7107272	7.7188E-07	0.00236532	H3K27ac	BF
chr2	133701012	133701064	DEL	chr2_134083127_134083509	10.1083771	13.7028861	7.7529E-07	0.00236532	H3K27ac	BF
chr2	133899189	133902056	DEL	chr2_134083127_134083509	10.1083771	13.7028861	7.7529E-07	0.00236532	H3K27ac	BF
chr2	133904101	133904172	DEL	chr2_134083127_134083509	10.1083771	13.7028861	7.7529E-07	0.00236532	H3K27ac	BF
chr1	158903207	158903407	DEL	chr1_158456102_158456347	5.90612222	13.707461	7.733E-07	0.00236532	H3K27ac	BF
chr2	133913002	133913003	INS	chr2_134083127_134083509	10.1083771	13.7028861	7.7529E-07	0.00236532	H3K27ac	BF
chr2	133907109	133907110	INS	chr2_134083127_134083509	10.1083771	13.7028861	7.7529E-07	0.00236532	H3K27ac	BF
chr2	133904244	133904245	INS	chr2_134083127_134083509	10.1083771	13.7028861	7.7529E-07	0.00236532	H3K27ac	BF
chr2	134416866	134416867	INS	chr2_134083127_134083509	10.1083771	13.7028861	7.7529E-07	0.00236532	H3K27ac	BF
chr2	134503933	134503934	INS	chr2_134083127_134083509	10.1083771	13.7028861	7.7529E-07	0.00236532	H3K27ac	BF
chr1	158586885	158586886	INS	chr1_158456102_158456347	5.90612222	13.707461	7.733E-07	0.00236532	H3K27ac	BF
chr1	158815069	158815070	INS	chr1_158456102_158456347	5.90612222	13.707461	7.733E-07	0.00236532	H3K27ac	BF
chr1	32231956	32231957	INS	chr1_32184830_32185199	2.61589506	13.6866799	7.824E-07	0.00237716	H3K27ac	BF

chr1	32549279	32549280	INS	chr1_32184830_32185199	2.61589506	13.6866799	7.824E-07	0.00237716	H3K27ac	BF
chr1	32658817	32658818	INS	chr1_32184830_32185199	2.61589506	13.6866799	7.824E-07	0.00237716	H3K27ac	BF
chr1	32661405	32661406	INS	chr1_32184830_32185199	2.61589506	13.6866799	7.824E-07	0.00237716	H3K27ac	BF
chr1	72225691	72225692	INS	chr1_71742842_71743867	3.12251939	13.681507	7.8468E-07	0.00238164	H3K27ac	BF
chr5	35738926	35739036	DEL	chr5_35491843_35492175	2.91980303	13.6592962	7.9456E-07	0.00239931	H3K27ac	BF
chr5	35734081	35734206	DEL	chr5_35491843_35492175	2.91980303	13.6592962	7.9456E-07	0.00239931	H3K27ac	BF
chr5	35864767	35865108	DEL	chr5_35491843_35492175	2.91980303	13.6592962	7.9456E-07	0.00239931	H3K27ac	BF
chr5	35456206	35456207	INS	chr5_35491843_35492175	2.91980303	13.6592962	7.9456E-07	0.00239931	H3K27ac	BF
chr5	28909531	28909532	INS	chr5_28538033_28538199	5.77018625	13.6570339	7.9558E-07	0.00239992	H3K27ac	BF
chr5	7581505	7581559	DEL	chr5_7210117_7211833	5.34351375	13.6239358	8.1059E-07	0.00244022	H3K27ac	BF
chr5	7579345	7579621	DEL	chr5_7210117_7211833	5.34351375	13.6239358	8.1059E-07	0.00244022	H3K27ac	BF
chr1	268866273	268866274	INS	chr1_269273447_269273711	4.26001344	13.6126369	8.1578E-07	0.00245336	H3K27ac	BF
chr3	79564678	79564743	DEL	chr3_79337738_79340398	6.29648778	13.5951981	8.2388E-07	0.00245768	H3K27ac	BF

chr5	70684409	70684410	INS	chr5_71152941_71154212	7.05817222	13.5963229	8.2335E-07	0.00245768	H3K27ac	BF
chr5	70678854	70678855	INS	chr5_71152941_71154212	7.05817222	13.5963229	8.2335E-07	0.00245768	H3K27ac	BF
chr5	70942608	70942609	INS	chr5_71152941_71154212	7.05817222	13.5963229	8.2335E-07	0.00245768	H3K27ac	BF
chr3	78913907	78913908	INS	chr3_79337738_79340398	6.29648778	13.5951981	8.2388E-07	0.00245768	H3K27ac	BF
chr3	79773741	79773742	INS	chr3_79337738_79340398	6.29648778	13.5951981	8.2388E-07	0.00245768	H3K27ac	BF
chr5	77362281	77363238	DEL	chr5_77718772_77719136	6.12858521	13.5436883	8.4831E-07	0.00252547	H3K27ac	BF
chr5	77806006	77806007	INS	chr5_77718772_77719136	6.12858521	13.5436883	8.4831E-07	0.00252547	H3K27ac	BF
chr2	11307422	11307423	INS	chr2_10853118_10853940	16.2609722	13.5260151	8.5688E-07	0.00254842	H3K27ac	BF
chr2	13122273	13123683	DEL	chr2_13459624_13459912	16.9466024	13.5161487	8.6171E-07	0.00255698	H3K27ac	BF
chr3	56270077	56270078	INS	chr3_56676876_56677565	8.56340694	13.5113066	8.6409E-07	0.00255698	H3K27ac	BF
chr3	56635856	56635857	INS	chr3_56676876_56677565	8.56340694	13.5113066	8.6409E-07	0.00255698	H3K27ac	BF
chr3	56766242	56766243	INS	chr3_56676876_56677565	8.56340694	13.5113066	8.6409E-07	0.00255698	H3K27ac	BF
chr1	26247780	26247781	INS	chr1_26301307_26301464	5.30981585	13.5158332	8.6187E-07	0.00255698	H3K27ac	BF

chr1	11626419	11626420	INS	chr1_11944714_11945394	3.29365963	13.4843739	8.7746E-07	0.00258877	H3K27ac	BF
chr3	101852520	101852521	INS	chr3_102241523_102241860	5.78121292	13.4678831	8.8577E-07	0.00260466	H3K27ac	BF
chr3	101899978	101899979	INS	chr3_102241523_102241860	5.78121292	13.4678831	8.8577E-07	0.00260466	H3K27ac	BF
chr3	121657515	121657516	INS	chr3_121781242_121781798	3.7435555	13.4676141	8.859E-07	0.00260466	H3K27ac	BF
chr1	129061546	129061547	INS	chr1_128962033_128964313	-14.431375	-13.466677	8.8638E-07	0.00260466	H3K27ac	BF
chr2	132195622	132195703	DEL	chr2_131882112_131882401	15.8944088	13.4410475	8.9947E-07	0.00263787	H3K27ac	BF
chr2	132071320	132071321	INS	chr2_131882112_131882401	15.8944088	13.4410475	8.9947E-07	0.00263787	H3K27ac	BF
chr1	1511558	1511813	DEL	chr1_1834438_1836074	8.63621667	13.4320666	9.041E-07	0.00264098	H3K27ac	BF
chr1	1697599	1697690	DEL	chr1_1834438_1836074	8.63621667	13.4320666	9.041E-07	0.00264098	H3K27ac	BF
chr1	2307186	2307261	DEL	chr1_1834438_1836074	8.63621667	13.4320666	9.041E-07	0.00264098	H3K27ac	BF
chr1	1779044	1779045	INS	chr1_1834438_1836074	8.63621667	13.4320666	9.041E-07	0.00264098	H3K27ac	BF
chr1	184341764	184341765	INS	chr1_184093897_184094164	8.91222521	13.4174341	9.1172E-07	0.00265272	H3K27ac	BF
chr1	184417738	184417739	INS	chr1_184093897_184094164	8.91222521	13.4174341	9.1172E-07	0.00265272	H3K27ac	BF

chr1	184444660	184444661	INS	chr1_184093897_184094164	8.91222521	13.4174341	9.1172E-07	0.00265272	H3K27ac	BF
chr1	8356436	8356437	INS	chr1_8408822_8409290	7.23320125	13.3962957	9.2284E-07	0.00268245	H3K27ac	BF
chr5	74417165	74417166	INS	chr5_74650027_74650271	4.23489306	13.3862394	9.2819E-07	0.00269269	H3K27ac	BF
chr5	74865495	74865496	INS	chr5_74650027_74650271	4.23489306	13.3862394	9.2819E-07	0.00269269	H3K27ac	BF
chr3	100624231	100624292	DEL	chr3_100316260_100316624	8.62790125	13.3632016	9.4057E-07	0.00272593	H3K27ac	BF
chr5	10454542	10454543	INS	chr5_10789631_10790253	3.60439011	13.3546061	9.4524E-07	0.00272607	H3K27ac	BF
chr5	11021536	11021537	INS	chr5_10789631_10790253	3.60439011	13.3546061	9.4524E-07	0.00272607	H3K27ac	BF
chr5	11050179	11050180	INS	chr5_10789631_10790253	3.60439011	13.3546061	9.4524E-07	0.00272607	H3K27ac	BF
chr5	11265336	11265337	INS	chr5_10789631_10790253	3.60439011	13.3546061	9.4524E-07	0.00272607	H3K27ac	BF
chr1	20834452	20834453	INS	chr1_20891450_20892436	7.53560244	13.3611728	9.4167E-07	0.00272607	H3K27ac	BF
chr3	16621069	16621070	INS	chr3_16357402_16358317	4.50872	13.342222	9.52E-07	0.00273491	H3K27ac	BF
chr3	16611489	16611490	INS	chr3_16357402_16358317	4.50872	13.342222	9.52E-07	0.00273491	H3K27ac	BF
chr3	16854353	16854354	INS	chr3_16357402_16358317	4.50872	13.342222	9.52E-07	0.00273491	H3K27ac	BF
chr1	188641399	188641400	INS	chr1_188183095_188183615	4.28164669	13.3399693	9.5324E-07	0.0027358	H3K27ac	BF

chr1	190223373	190223374	INS	chr1_190543387_190543737	6.20204563	13.3309246	9.5822E-07	0.00274744	H3K27ac	BF
chr1	54189483	54189539	DEL	chr1_54436752_54437680	11.3148635	13.3263122	9.6078E-07	0.00274942	H3K27ac	BF
chr1	54560788	54560789	INS	chr1_54436752_54437680	11.3148635	13.3263122	9.6078E-07	0.00274942	H3K27ac	BF
chr4	71017558	71017841	DEL	chr4_70909201_70910192	6.03403394	13.2984542	9.7636E-07	0.00278861	H3K27ac	BF
chr1	189872667	189874527	DEL	chr1_190311228_190311818	4.27752406	13.2692613	9.93E-07	0.00283338	H3K27ac	BF
chr5	5629447	5632537	DEL	chr5_6017698_6017934	8.3375084	13.2592999	9.9875E-07	0.00283882	H3K27ac	BF
chr5	5937136	5937631	DEL	chr5_6017698_6017934	8.3375084	13.2592999	9.9875E-07	0.00283882	H3K27ac	BF
chr5	5794458	5794459	INS	chr5_6017698_6017934	8.3375084	13.2592999	9.9875E-07	0.00283882	H3K27ac	BF
chr3	18608956	18609099	DEL	chr3_18877373_18877665	5.85434389	13.220049	1.0218E-06	0.0028848	H3K27ac	BF
chr2	129760003	129760004	INS	chr2_129583786_129584130	5.05971875	13.2203171	1.0216E-06	0.0028848	H3K27ac	BF
chr3	18601690	18601691	INS	chr3_18877373_18877665	5.85434389	13.220049	1.0218E-06	0.0028848	H3K27ac	BF
chr1	43898539	43898540	INS	chr1_44335164_44335818	3.98449007	13.2201376	1.0217E-06	0.0028848	H3K27ac	BF
chr1	44087119	44087120	INS	chr1_44335164_44335818	3.98449007	13.2201376	1.0217E-06	0.0028848	H3K27ac	BF

chr1	44132637	44132638	INS	chr1_44335164_44335818	3.98449007	13.2201376	1.0217E-06	0.0028848	H3K27ac	BF
chr2	113083728	113084743	DEL	chr2_113528245_113529949	1.63561396	13.2159474	1.0242E-06	0.00288617	H3K27ac	BF
chr2	113461690	113461691	INS	chr2_113528245_113529949	1.63561396	13.2159474	1.0242E-06	0.00288617	H3K27ac	BF
chr1	104052039	104052552	DEL	chr1_103853847_103854701	8.0036325	13.2101069	1.0277E-06	0.0028898	H3K27ac	BF
chr3	124835193	124835194	INS	chr3_124375288_124375621	5.64299317	13.2077741	1.0291E-06	0.0028898	H3K27ac	BF
chr1	20834452	20834453	INS	chr1_20673253_20673490	3.26557525	13.2076721	1.0291E-06	0.0028898	H3K27ac	BF
chr5	28909531	28909532	INS	chr5_28600861_28601108	2.3162775	13.1975923	1.0352E-06	0.00290331	H3K27ac	BF
chr3	124312874	124312875	INS	chr3_123978828_123979529	1.93135706	13.1646819	1.0552E-06	0.00295393	H3K27ac	BF
chr3	124462415	124462416	INS	chr3_123978828_123979529	1.93135706	13.1646819	1.0552E-06	0.00295393	H3K27ac	BF
chr1	8356436	8356437	INS	chr1_8574711_8575394	4.89371563	13.1587471	1.0589E-06	0.00296137	H3K27ac	BF
chr4	123880440	123881468	DEL	chr4_123394461_123395467	12.0256417	13.141583	1.0696E-06	0.00297877	H3K27ac	BF
chr1	41269681	41269890	DEL	chr1_40884336_40885201	8.75081806	13.1390184	1.0712E-06	0.00297877	H3K27ac	BF
chr4	123879499	123879500	INS	chr4_123394461_123395467	12.0256417	13.141583	1.0696E-06	0.00297877	H3K27ac	BF

chr1	40572342	40572343	INS	chr1_40884336_40885201	8.75081806	13.1390184	1.0712E-06	0.00297877	H3K27ac	BF
chr1	40963751	40963752	INS	chr1_40884336_40885201	8.75081806	13.1390184	1.0712E-06	0.00297877	H3K27ac	BF
chr1	40970666	40970667	INS	chr1_40884336_40885201	8.75081806	13.1390184	1.0712E-06	0.00297877	H3K27ac	BF
chr1	188641399	188641400	INS	chr1_188182380_188182707	2.87228369	13.1258724	1.0795E-06	0.00299894	H3K27ac	BF
chr4	71017558	71017841	DEL	chr4_71044923_71045736	4.43044819	13.0998049	1.0961E-06	0.00304221	H3K27ac	BF
chr1	104052039	104052552	DEL	chr1_103735908_103736534	4.12177513	13.0206563	1.1483E-06	0.00318411	H3K27ac	BF
chr1	240699894	240699895	INS	chr1_240675211_240675461	5.52785889	13.013149	1.1534E-06	0.00319523	H3K27ac	BF
chr2	13122273	13123683	DEL	chr2_13420212_13421463	12.8066243	13.0047502	1.1591E-06	0.0032081	H3K27ac	BF
chr1	104052039	104052552	DEL	chr1_103856527_103857338	9.45089438	12.9932721	1.167E-06	0.00322688	H3K27ac	BF
chr3	93195340	93195413	DEL	chr3_93371304_93373642	-3.4460622	-12.985525	1.1723E-06	0.00323563	H3K27ac	BF
chr3	93423836	93423892	DEL	chr3_93371304_93373642	-3.4460622	-12.985525	1.1723E-06	0.00323563	H3K27ac	BF
chr2	13122273	13123683	DEL	chr2_13142613_13143125	29.7956764	12.9802541	1.176E-06	0.00324269	H3K27ac	BF
chr1	26247780	26247781	INS	chr1_26465749_26466242	4.82833563	12.9782549	1.1774E-06	0.0032435	H3K27ac	BF

chr2	12789310	12789615	DEL	chr2_12487920_12490492	19.2657354	12.9344044	1.2083E-06	0.00331337	H3K27ac	BF
chr2	12116201	12116202	INS	chr2_12487920_12490492	19.2657354	12.9344044	1.2083E-06	0.00331337	H3K27ac	BF
chr2	12126050	12126051	INS	chr2_12487920_12490492	19.2657354	12.9344044	1.2083E-06	0.00331337	H3K27ac	BF
chr2	12327115	12327116	INS	chr2_12487920_12490492	19.2657354	12.9344044	1.2083E-06	0.00331337	H3K27ac	BF
chr2	12747947	12747948	INS	chr2_12487920_12490492	19.2657354	12.9344044	1.2083E-06	0.00331337	H3K27ac	BF
chr2	135094132	135094133	INS	chr2_134677018_134678702	-5.1460656	-12.931617	1.2103E-06	0.00331577	H3K27ac	BF
chr3	112749795	112749890	DEL	chr3_113114306_113114464	4.89744444	12.9180924	1.2201E-06	0.00333322	H3K27ac	BF
chr3	112950366	112950367	INS	chr3_113114306_113114464	4.89744444	12.9180924	1.2201E-06	0.00333322	H3K27ac	BF
chr3	113202306	113202307	INS	chr3_113114306_113114464	4.89744444	12.9180924	1.2201E-06	0.00333322	H3K27ac	BF
chr3	101852520	101852521	INS	chr3_102103617_102103905	4.09603889	12.9126221	1.2241E-06	0.00333789	H3K27ac	BF
chr3	101899978	101899979	INS	chr3_102103617_102103905	4.09603889	12.9126221	1.2241E-06	0.00333789	H3K27ac	BF
chr1	96099461	96100904	DEL	chr1_96462561_96463297	7.70451667	12.8704304	1.2552E-06	0.00341279	H3K27ac	BF
chr1	96017447	96017448	INS	chr1_96462561_96463297	7.70451667	12.8704304	1.2552E-06	0.00341279	H3K27ac	BF

chr1	96026418	96026419	INS	chr1_96462561_96463297	7.70451667	12.8704304	1.2552E-06	0.00341279	H3K27ac	BF
chr1	112842193	112842194	INS	chr1_112865120_112865567	4.43181356	12.8691264	1.2561E-06	0.00341279	H3K27ac	BF
chr3	60627776	60627777	INS	chr3_60665491_60665936	1.4720871	12.8572784	1.2651E-06	0.00343381	H3K27ac	BF
chr2	134080328	134081531	DEL	chr2_133717757_133718428	6.26308288	12.8502773	1.2703E-06	0.00344502	H3K27ac	BF
chr1	168716660	168716661	INS	chr1_168434619_168434909	4.14557944	12.8036204	1.3063E-06	0.00353594	H3K27ac	BF
chr1	8356436	8356437	INS	chr1_8438164_8439010	5.2373425	12.7921351	1.3153E-06	0.00355708	H3K27ac	BF
chr3	96511189	96511475	DEL	chr3_96783663_96784144	8.50792061	12.7764865	1.3277E-06	0.00356449	H3K27ac	BF
chr3	96525948	96526127	DEL	chr3_96783663_96784144	8.50792061	12.7764865	1.3277E-06	0.00356449	H3K27ac	BF
chr3	96711570	96711720	DEL	chr3_96783663_96784144	8.50792061	12.7764865	1.3277E-06	0.00356449	H3K27ac	BF
chr3	96716275	96716522	DEL	chr3_96783663_96784144	8.50792061	12.7764865	1.3277E-06	0.00356449	H3K27ac	BF
chr3	96714242	96714783	DEL	chr3_96783663_96784144	17.0158412	12.7764865	1.3277E-06	0.00356449	H3K27ac	BF
chr3	96721584	96721698	DEL	chr3_96783663_96784144	8.50792061	12.7764865	1.3277E-06	0.00356449	H3K27ac	BF
chr3	96459393	96459394	INS	chr3_96783663_96784144	8.50792061	12.7764865	1.3277E-06	0.00356449	H3K27ac	BF

chr3	96690622	96690623	INS	chr3_96783663_96784144	8.50792061	12.7764865	1.3277E-06	0.00356449	H3K27ac	BF
chr2	87378442	87378532	DEL	chr2_87513333_87514438	5.5269865	12.7674575	1.3349E-06	0.00357411	H3K27ac	BF
chr2	87847374	87847375	INS	chr2_87513333_87514438	5.5269865	12.7674575	1.3349E-06	0.00357411	H3K27ac	BF
chr1	129061546	129061547	INS	chr1_129026268_129028230	-7.1704875	-12.767886	1.3345E-06	0.00357411	H3K27ac	BF
chr1	265047123	265047124	INS	chr1_265539175_265539745	3.72852111	12.763443	1.3381E-06	0.00357625	H3K27ac	BF
chr1	265730426	265730427	INS	chr1_265539175_265539745	3.72852111	12.763443	1.3381E-06	0.00357625	H3K27ac	BF
chr4	39225700	39225776	DEL	chr4_39049795_39049958	5.64428456	12.7490811	1.3497E-06	0.00359747	H3K27ac	BF
chr4	38737338	38737339	INS	chr4_39049795_39049958	5.64428456	12.7490811	1.3497E-06	0.00359747	H3K27ac	BF
chr4	39152606	39152607	INS	chr4_39049795_39049958	5.64428456	12.7490811	1.3497E-06	0.00359747	H3K27ac	BF
chr2	13122273	13123683	DEL	chr2_13497448_13497733	22.6220079	12.7236396	1.3705E-06	0.00364965	H3K27ac	BF
chr2	68602732	68602856	DEL	chr2_68948216_68948586	4.46374514	12.7064026	1.3848E-06	0.0036679	H3K27ac	BF
chr2	68611697	68611857	DEL	chr2_68948216_68948586	4.46374514	12.7064026	1.3848E-06	0.0036679	H3K27ac	BF
chr2	69376163	69376442	DEL	chr2_68948216_68948586	4.46374514	12.7064026	1.3848E-06	0.0036679	H3K27ac	BF

chr2	68889784	68889785	INS	chr2_68948216_68948586	4.46374514	12.7064026	1.3848E-06	0.0036679	H3K27ac	BF
chr2	69408537	69408538	INS	chr2_68948216_68948586	4.46374514	12.7064026	1.3848E-06	0.0036679	H3K27ac	BF
chr1	134075402	134075403	INS	chr1_134044710_134045422	5.11778014	12.7092363	1.3825E-06	0.0036679	H3K27ac	BF
chr1	114488786	114488787	INS	chr1_114357410_114358205	4.00332661	12.6628816	1.4217E-06	0.00375546	H3K27ac	BF
chr1	114620312	114620313	INS	chr1_114357410_114358205	4.00332661	12.6628816	1.4217E-06	0.00375546	H3K27ac	BF
chr1	114614128	114614129	INS	chr1_114357410_114358205	4.00332661	12.6628816	1.4217E-06	0.00375546	H3K27ac	BF
chr2	6011831	6011832	INS	chr2_6393537_6393983	1.2923435	12.6371783	1.444E-06	0.00380753	H3K27ac	BF
chr2	6556735	6556736	INS	chr2_6393537_6393983	1.2923435	12.6371783	1.444E-06	0.00380753	H3K27ac	BF
chr1	179531942	179532100	DEL	chr1_179730245_179730497	5.22981622	12.6220529	1.4573E-06	0.00383575	H3K27ac	BF
chr1	179437275	179437276	INS	chr1_179730245_179730497	5.22981622	12.6220529	1.4573E-06	0.00383575	H3K27ac	BF
chr3	39342385	39342688	DEL	chr3_39253891_39254396	7.8555745	12.6015077	1.4756E-06	0.00388043	H3K27ac	BF
chr1	86702062	86702063	INS	chr1_87123622_87124721	3.248845	12.5958545	1.4807E-06	0.0038834	H3K27ac	BF
chr1	86756615	86756616	INS	chr1_87123622_87124721	3.248845	12.5958545	1.4807E-06	0.0038834	H3K27ac	BF

chr1	87556573	87556574	INS	chr1_87123622_87124721	3.248845	12.5958545	1.4807E-06	0.0038834	H3K27ac	BF
chr3	93195340	93195413	DEL	chr3_93416985_93418597	7.19636761	12.5469095	1.5254E-06	0.00399376	H3K27ac	BF
chr3	93423836	93423892	DEL	chr3_93416985_93418597	7.19636761	12.5469095	1.5254E-06	0.00399376	H3K27ac	BF
chr5	31001378	31001673	DEL	chr5_30576918_30577323	3.10404688	12.5420167	1.53E-06	0.00400214	H3K27ac	BF
chr3	44031071	44031129	DEL	chr3_43949518_43950241	4.23766833	12.5358374	1.5358E-06	0.00401016	H3K27ac	BF
chr3	44035533	44035599	DEL	chr3_43949518_43950241	4.23766833	12.5358374	1.5358E-06	0.00401016	H3K27ac	BF
chr5	3596904	3597581	DEL	chr5_3715397_3715690	4.92681535	12.5171613	1.5534E-06	0.004049	H3K27ac	BF
chr5	3651763	3651814	DEL	chr5_3715397_3715690	4.92681535	12.5171613	1.5534E-06	0.004049	H3K27ac	BF
chr1	104052039	104052552	DEL	chr1_103727969_103730188	4.17094288	12.5018148	1.568E-06	0.00408357	H3K27ac	BF
chr4	71017558	71017841	DEL	chr4_71008188_71009184	2.2831475	12.484834	1.5844E-06	0.00411242	H3K27ac	BF
chr1	188108680	188109925	DEL	chr1_188233304_188233594	6.00932819	12.4845647	1.5847E-06	0.00411242	H3K27ac	BF
chr2	13122273	13123683	DEL	chr2_13204063_13204283	20.0914257	12.4874854	1.5819E-06	0.00411242	H3K27ac	BF
chr1	187775529	187775530	INS	chr1_188233304_188233594	6.00932819	12.4845647	1.5847E-06	0.00411242	H3K27ac	BF

chr4	61434992	61437571	DEL	chr4_61336955_61337291	6.7306184	12.4701079	1.5988E-06	0.00411647	H3K27ac	BF
chr4	61466393	61466462	DEL	chr4_61336955_61337291	6.7306184	12.4701079	1.5988E-06	0.00411647	H3K27ac	BF
chr4	60869108	60869109	INS	chr4_61336955_61337291	6.7306184	12.4701079	1.5988E-06	0.00411647	H3K27ac	BF
chr4	61369809	61369810	INS	chr4_61336955_61337291	6.7306184	12.4701079	1.5988E-06	0.00411647	H3K27ac	BF
chr4	61433125	61433126	INS	chr4_61336955_61337291	6.7306184	12.4701079	1.5988E-06	0.00411647	H3K27ac	BF
chr4	61454319	61454320	INS	chr4_61336955_61337291	6.7306184	12.4701079	1.5988E-06	0.00411647	H3K27ac	BF
chr4	61614516	61614517	INS	chr4_61336955_61337291	6.7306184	12.4701079	1.5988E-06	0.00411647	H3K27ac	BF
chr4	61811174	61811175	INS	chr4_61336955_61337291	6.7306184	12.4701079	1.5988E-06	0.00411647	H3K27ac	BF
chr5	62540128	62540433	DEL	chr5_62751955_62752859	3.95973669	12.4636685	1.6051E-06	0.00412916	H3K27ac	BF
chr5	41398825	41398826	INS	chr5_41741507_41742488	24.1223836	12.4458026	1.6228E-06	0.004153	H3K27ac	BF
chr5	41406216	41406217	INS	chr5_41741507_41742488	24.1223836	12.4458026	1.6228E-06	0.004153	H3K27ac	BF
chr5	41446260	41446261	INS	chr5_41741507_41742488	24.1223836	12.4458026	1.6228E-06	0.004153	H3K27ac	BF
chr5	41571403	41571404	INS	chr5_41741507_41742488	24.1223836	12.4458026	1.6228E-06	0.004153	H3K27ac	BF

chr5	41851424	41851425	INS	chr5_41741507_41742488	24.1223836	12.4458026	1.6228E-06	0.004153	H3K27ac	BF
chr5	42116965	42116966	INS	chr5_41741507_41742488	24.1223836	12.4458026	1.6228E-06	0.004153	H3K27ac	BF
chr5	23006530	23006531	INS	chr5_22620671_22620925	4.63825861	12.4426762	1.6259E-06	0.00415738	H3K27ac	BF
chr1	20834452	20834453	INS	chr1_20855151_20856044	3.35786825	12.4384773	1.6301E-06	0.00416452	H3K27ac	BF
chr3	126604486	126604487	INS	chr3_126127152_126127469	8.56339	12.4336474	1.635E-06	0.00416969	H3K27ac	BF
chr2	140666739	140666978	DEL	chr2_141126160_141126565	4.00552549	12.4232826	1.6454E-06	0.00417832	H3K27ac	BF
chr2	140915494	140917530	DEL	chr2_141126160_141126565	4.00552549	12.4232826	1.6454E-06	0.00417832	H3K27ac	BF
chr1	80588498	80588499	INS	chr1_80370374_80371642	2.09267333	12.4261158	1.6426E-06	0.00417832	H3K27ac	BF
chr2	140729157	140729158	INS	chr2_141126160_141126565	4.00552549	12.4232826	1.6454E-06	0.00417832	H3K27ac	BF
chr2	141254237	141254238	INS	chr2_141126160_141126565	4.00552549	12.4232826	1.6454E-06	0.00417832	H3K27ac	BF
chr5	70601089	70601090	INS	chr5_70650405_70651501	3.21967006	12.3912536	1.6782E-06	0.00424334	H3K27ac	BF
chr5	70684409	70684410	INS	chr5_70650405_70651501	3.21967006	12.3912536	1.6782E-06	0.00424334	H3K27ac	BF
chr5	70678854	70678855	INS	chr5_70650405_70651501	3.21967006	12.3912536	1.6782E-06	0.00424334	H3K27ac	BF

chr5	70942608	70942609	INS	chr5_70650405_70651501	3.21967006	12.3912536	1.6782E-06	0.00424334	H3K27ac	BF
chr5	7581505	7581559	DEL	chr5_7637454_7638620	3.72238563	12.3660178	1.7046E-06	0.0043026	H3K27ac	BF
chr5	7579345	7579621	DEL	chr5_7637454_7638620	3.72238563	12.3660178	1.7046E-06	0.0043026	H3K27ac	BF
chr1	91556900	91557060	DEL	chr1_91250123_91252359	2.03964167	12.3028323	1.7726E-06	0.00446359	H3K27ac	BF
chr1	11626419	11626420	INS	chr1_12078744_12079198	2.80562019	12.3012066	1.7744E-06	0.00446359	H3K27ac	BF
chr2	143620924	143620983	DEL	chr2_144036758_144037162	3.60649278	12.2907243	1.786E-06	0.00448313	H3K27ac	BF
chr1	129061546	129061547	INS	chr1_129267901_129268479	-47.108643	-12.2887	1.7883E-06	0.00448313	H3K27ac	BF
chr2	143620660	143620661	INS	chr2_144036758_144037162	3.60649278	12.2907243	1.786E-06	0.00448313	H3K27ac	BF
chr3	22347726	22347727	INS	chr3_22720331_22720726	2.84413822	12.2891884	1.7877E-06	0.00448313	H3K27ac	BF
chr1	23192903	23194104	DEL	chr1_22727365_22728912	3.36992299	12.2804676	1.7974E-06	0.0044985	H3K27ac	BF
chr1	22795548	22795549	INS	chr1_22727365_22728912	3.36992299	12.2804676	1.7974E-06	0.0044985	H3K27ac	BF
chr2	40550891	40550892	INS	chr2_40886298_40886513	5.72670056	12.2548573	1.8263E-06	0.00456305	H3K27ac	BF
chr2	41117465	41117466	INS	chr2_40886298_40886513	5.72670056	12.2548573	1.8263E-06	0.00456305	H3K27ac	BF

chr1	75066219	75066220	INS	chr1_75136648_75137228	27.3981239	12.2308334	1.8539E-06	0.0046202	H3K27ac	BF
chr1	75534021	75534022	INS	chr1_75136648_75137228	27.3981239	12.2308334	1.8539E-06	0.0046202	H3K27ac	BF
chr1	75587668	75587669	INS	chr1_75136648_75137228	27.3981239	12.2308334	1.8539E-06	0.0046202	H3K27ac	BF
chr4	109954653	109954654	INS	chr4_110003520_110003723	6.79175007	12.2167412	1.8703E-06	0.00464927	H3K27ac	BF
chr4	110485626	110485627	INS	chr4_110003520_110003723	6.79175007	12.2167412	1.8703E-06	0.00464927	H3K27ac	BF
chr2	3874759	3874824	DEL	chr2_4186860_4187252	1.20899594	12.1886936	1.9034E-06	0.00472361	H3K27ac	BF
chr2	6971934	6972779	DEL	chr2_7050999_7051710	1.7088665	12.165618	1.9311E-06	0.00477635	H3K27ac	BF
chr2	6973113	6973278	DEL	chr2_7050999_7051710	1.7088665	12.165618	1.9311E-06	0.00477635	H3K27ac	BF
chr2	7089639	7089857	DEL	chr2_7050999_7051710	1.7088665	12.165618	1.9311E-06	0.00477635	H3K27ac	BF
chr2	7105946	7105947	INS	chr2_7050999_7051710	1.7088665	12.165618	1.9311E-06	0.00477635	H3K27ac	BF
chr3	93195340	93195413	DEL	chr3_92764654_92765392	7.57926306	12.1601104	1.9378E-06	0.00478087	H3K27ac	BF
chr5	54104163	54104164	INS	chr5_54281504_54282801	3.61079238	12.1625758	1.9348E-06	0.00478087	H3K27ac	BF
chr3	92761333	92761334	INS	chr3_92764654_92765392	7.57926306	12.1601104	1.9378E-06	0.00478087	H3K27ac	BF

chr2	134080328	134081531	DEL	chr2_133628851_133629235	3.03596177	12.1397848	1.9627E-06	0.00483824	H3K27ac	BF
chr5	3596904	3597581	DEL	chr5_3894131_3894555	7.84938111	12.1020382	2.0098E-06	0.00493396	H3K27ac	BF
chr5	3651763	3651814	DEL	chr5_3894131_3894555	7.84938111	12.1020382	2.0098E-06	0.00493396	H3K27ac	BF
chr3	101852520	101852521	INS	chr3_102155748_102156116	3.37642972	12.1025658	2.0092E-06	0.00493396	H3K27ac	BF
chr3	101899978	101899979	INS	chr3_102155748_102156116	3.37642972	12.1025658	2.0092E-06	0.00493396	H3K27ac	BF
chr3	44031071	44031129	DEL	chr3_44365589_44366292	6.90644667	12.0960761	2.0174E-06	0.00494431	H3K27ac	BF
chr3	44035533	44035599	DEL	chr3_44365589_44366292	6.90644667	12.0960761	2.0174E-06	0.00494431	H3K27ac	BF
chr4	67430927	67431504	DEL	chr4_66974732_66975184	1.55035425	12.0850186	2.0315E-06	0.00494608	H3K27ac	BF
chr4	67441371	67442086	DEL	chr4_66974732_66975184	1.55035425	12.0850186	2.0315E-06	0.00494608	H3K27ac	BF
chr4	67352065	67352066	INS	chr4_66974732_66975184	1.55035425	12.0850186	2.0315E-06	0.00494608	H3K27ac	BF
chr2	64300537	64300538	INS	chr2_64620878_64621430	20.1062088	12.0888623	2.0266E-06	0.00494608	H3K27ac	BF
chr4	67430930	67431664	INV	chr4_66974732_66975184	3.1007085	12.0850186	2.0315E-06	0.00494608	H3K27ac	BF
chr2	13122273	13123683	DEL	chr2_13629675_13633613	-15.327045	-12.066635	2.0553E-06	0.00499971	H3K27ac	BF

chr4	111529407	111529590	DEL	chr4_111480885_111481162	5.43499183	12.052096	2.0742E-06	0.00500057	H3K27ac	BF
chr2	13122273	13123683	DEL	chr2_12985972_12986270	15.6780001	12.0635274	2.0593E-06	0.00500057	H3K27ac	BF
chr4	111094333	111094334	INS	chr4_111480885_111481162	5.43499183	12.052096	2.0742E-06	0.00500057	H3K27ac	BF
chr4	111155923	111155924	INS	chr4_111480885_111481162	5.43499183	12.052096	2.0742E-06	0.00500057	H3K27ac	BF
chr4	111158124	111158125	INS	chr4_111480885_111481162	5.43499183	12.052096	2.0742E-06	0.00500057	H3K27ac	BF
chr4	111482116	111482117	INS	chr4_111480885_111481162	5.43499183	12.052096	2.0742E-06	0.00500057	H3K27ac	BF
chr4	111524672	111524673	INS	chr4_111480885_111481162	5.43499183	12.052096	2.0742E-06	0.00500057	H3K27ac	BF
chr4	111876285	111876286	INS	chr4_111480885_111481162	5.43499183	12.052096	2.0742E-06	0.00500057	H3K27ac	BF
chr4	111869970	111869971	INS	chr4_111480885_111481162	5.43499183	12.052096	2.0742E-06	0.00500057	H3K27ac	BF
chr3	68152030	68152724	DEL	chr3_68387202_68387581	2.569984	12.0396642	2.0906E-06	0.00502366	H3K27ac	BF
chr3	68092589	68092590	INS	chr3_68387202_68387581	2.569984	12.0396642	2.0906E-06	0.00502366	H3K27ac	BF
chr3	68209516	68209517	INS	chr3_68387202_68387581	2.569984	12.0396642	2.0906E-06	0.00502366	H3K27ac	BF
chr3	68278870	68278871	INS	chr3_68387202_68387581	2.569984	12.0396642	2.0906E-06	0.00502366	H3K27ac	BF

chr2	13122273	13123683	DEL	chr2_13505511_13506028	10.6011949	11.9972313	2.1476E-06	0.00515649	H3K27ac	BF
chr2	112711763	112711882	DEL	chr2_112803786_112803988	4.37922931	11.9941166	2.1519E-06	0.00515831	H3K27ac	BF
chr2	112796879	112796880	INS	chr2_112803786_112803988	4.37922931	11.9941166	2.1519E-06	0.00515831	H3K27ac	BF
chr1	165770335	165770336	INS	chr1_166207368_166207882	3.698519	11.9855685	2.1636E-06	0.00518221	H3K27ac	BF
chr3	15789866	15789867	INS	chr3_16269082_16269513	6.62123472	11.9694845	2.1859E-06	0.00522279	H3K27ac	BF
chr3	15847597	15847598	INS	chr3_16269082_16269513	6.62123472	11.9694845	2.1859E-06	0.00522279	H3K27ac	BF
chr3	16268136	16268137	INS	chr3_16269082_16269513	6.62123472	11.9694845	2.1859E-06	0.00522279	H3K27ac	BF
chr4	99418220	99418221	INS	chr4_99807120_99807906	4.47924438	11.9427746	2.2234E-06	0.00528252	H3K27ac	BF
chr4	99792968	99792969	INS	chr4_99807120_99807906	4.47924438	11.9427746	2.2234E-06	0.00528252	H3K27ac	BF
chr4	100136603	100136604	INS	chr4_99807120_99807906	4.47924438	11.9427746	2.2234E-06	0.00528252	H3K27ac	BF
chr4	100168005	100168006	INS	chr4_99807120_99807906	4.47924438	11.9427746	2.2234E-06	0.00528252	H3K27ac	BF
chr4	100245877	100245878	INS	chr4_99807120_99807906	4.47924438	11.9427746	2.2234E-06	0.00528252	H3K27ac	BF
chr4	100217377	100217378	INS	chr4_99807120_99807906	4.47924438	11.9427746	2.2234E-06	0.00528252	H3K27ac	BF

chr4	100225175	100225176	INS	chr4_99807120_99807906	4.47924438	11.9427746	2.2234E-06	0.00528252	H3K27ac	BF
chr2	13122273	13123683	DEL	chr2_13331067_13331382	14.2580586	11.935695	2.2335E-06	0.00530217	H3K27ac	BF
chr3	103416473	103416474	INS	chr3_103468686_103470418	6.10169438	11.9313039	2.2397E-06	0.00531278	H3K27ac	BF
chr4	80372998	80372999	INS	chr4_80554138_80554972	6.37981833	11.9133225	2.2656E-06	0.00536124	H3K27ac	BF
chr4	80415815	80415816	INS	chr4_80554138_80554972	6.37981833	11.9133225	2.2656E-06	0.00536124	H3K27ac	BF
chr4	80728915	80728916	INS	chr4_80554138_80554972	6.37981833	11.9133225	2.2656E-06	0.00536124	H3K27ac	BF
chr5	62540128	62540433	DEL	chr5_62700962_62701428	4.40996188	11.8904948	2.2989E-06	0.00542965	H3K27ac	BF
chr5	7581505	7581559	DEL	chr5_7185240_7185502	9.68494688	11.8897516	2.3E-06	0.00542965	H3K27ac	BF
chr5	7579345	7579621	DEL	chr5_7185240_7185502	9.68494688	11.8897516	2.3E-06	0.00542965	H3K27ac	BF
chr2	126596215	126596496	DEL	chr2_126285428_126285725	2.52707488	11.8812774	2.3125E-06	0.00543361	H3K27ac	BF
chr5	64363334	64363335	INS	chr5_64587820_64588308	20.2376643	11.8859177	2.3057E-06	0.00543361	H3K27ac	BF
chr5	64367802	64367803	INS	chr5_64587820_64588308	20.2376643	11.8859177	2.3057E-06	0.00543361	H3K27ac	BF
chr5	74875100	74875101	INS	chr5_74991007_74991630	9.80162756	11.8811444	2.3127E-06	0.00543361	H3K27ac	BF
chr5	4189079	4189168	DEL	chr5_3775187_3775351	19.7180313	11.875786	2.3207E-06	0.00544797	H3K27ac	BF

chr1	26088493	26088670	DEL	chr1_26301307_26301464	5.24426257	11.8636007	2.3389E-06	0.005482	H3K27ac	BF
chr1	26539658	26539718	DEL	chr1_26301307_26301464	5.24426257	11.8636007	2.3389E-06	0.005482	H3K27ac	BF
chr1	34116497	34118117	DEL	chr1_33711583_33712265	3.10894756	11.8489952	2.3609E-06	0.00550742	H3K27ac	BF
chr1	33650404	33650405	INS	chr1_33711583_33712265	3.10894756	11.8489952	2.3609E-06	0.00550742	H3K27ac	BF
chr1	33639163	33639164	INS	chr1_33711583_33712265	3.10894756	11.8489952	2.3609E-06	0.00550742	H3K27ac	BF
chr5	62540128	62540433	DEL	chr5_62596276_62597773	4.76517344	11.8379431	2.3778E-06	0.0055423	H3K27ac	BF
chr3	121657515	121657516	INS	chr3_121672562_121672850	6.55471344	11.8317903	2.3872E-06	0.00555988	H3K27ac	BF
chr5	73979409	73979468	DEL	chr5_73704214_73705256	2.57095538	11.8244263	2.3985E-06	0.00558189	H3K27ac	BF
chr3	106398685	106398686	INS	chr3_106324972_106325755	2.38619994	11.8033433	2.4313E-06	0.00565375	H3K27ac	BF
chr5	70607550	70607551	INS	chr5_70163997_70165363	3.2850975	11.783058	2.4633E-06	0.00571472	H3K27ac	BF
chr5	73936605	73937297	DEL	chr5_73745622_73745971	2.33993811	11.7724521	2.4803E-06	0.00572254	H3K27ac	BF
chr5	73971208	73971315	DEL	chr5_73745622_73745971	2.33993811	11.7724521	2.4803E-06	0.00572254	H3K27ac	BF
chr5	73805971	73805972	INS	chr5_73745622_73745971	2.33993811	11.7724521	2.4803E-06	0.00572254	H3K27ac	BF

chr5	73924522	73924523	INS	chr5_73745622_73745971	2.33993811	11.7724521	2.4803E-06	0.00572254	H3K27ac	BF
chr5	73961834	73961835	INS	chr5_73745622_73745971	2.33993811	11.7724521	2.4803E-06	0.00572254	H3K27ac	BF
chr5	73947094	73947095	INS	chr5_73745622_73745971	2.33993811	11.7724521	2.4803E-06	0.00572254	H3K27ac	BF
chr5	73979409	73979468	DEL	chr5_73562066_73563412	5.29254313	11.7617269	2.4975E-06	0.00575785	H3K27ac	BF
chr1	8356436	8356437	INS	chr1_8429935_8430663	1.95754938	11.7492221	2.5178E-06	0.00580009	H3K27ac	BF
chr2	126596215	126596496	DEL	chr2_127030053_127030827	7.19195238	11.7467753	2.5218E-06	0.00580476	H3K27ac	BF
chr1	86136311	86136312	INS	chr1_86057846_86058600	4.18964183	11.7364481	2.5387E-06	0.00582103	H3K27ac	BF
chr1	86279573	86279574	INS	chr1_86057846_86058600	4.18964183	11.7364481	2.5387E-06	0.00582103	H3K27ac	BF
chr1	86508701	86508702	INS	chr1_86057846_86058600	4.18964183	11.7364481	2.5387E-06	0.00582103	H3K27ac	BF
chr2	90129340	90130737	DEL	chr2_90193613_90194470	9.59776139	11.7294715	2.5502E-06	0.00584288	H3K27ac	BF
chr4	71017558	71017841	DEL	chr4_70619492_70620718	5.61487481	11.7084586	2.5853E-06	0.00588945	H3K27ac	BF
chr4	77827678	77827800	DEL	chr4_78092234_78092757	3.37211701	11.7094028	2.5837E-06	0.00588945	H3K27ac	BF
chr4	117978801	117978916	DEL	chr4_117618738_117620141	2.75918667	11.7005939	2.5985E-06	0.00588945	H3K27ac	BF

chr4	118001729	118001880	DEL	chr4_117618738_117620141	2.75918667	11.7005939	2.5985E-06	0.00588945	H3K27ac	BF
chr4	117999909	118000198	DEL	chr4_117618738_117620141	2.75918667	11.7005939	2.5985E-06	0.00588945	H3K27ac	BF
chr5	7581505	7581559	DEL	chr5_7603113_7603396	2.24033888	11.7096525	2.5832E-06	0.00588945	H3K27ac	BF
chr5	7579345	7579621	DEL	chr5_7603113_7603396	2.24033888	11.7096525	2.5832E-06	0.00588945	H3K27ac	BF
chr4	117473079	117473080	INS	chr4_117618738_117620141	2.75918667	11.7005939	2.5985E-06	0.00588945	H3K27ac	BF
chr4	117746392	117746393	INS	chr4_117618738_117620141	2.75918667	11.7005939	2.5985E-06	0.00588945	H3K27ac	BF
chr4	117945046	117945047	INS	chr4_117618738_117620141	2.75918667	11.7005939	2.5985E-06	0.00588945	H3K27ac	BF
chr4	118002624	118002625	INS	chr4_117618738_117620141	2.75918667	11.7005939	2.5985E-06	0.00588945	H3K27ac	BF
chr4	118005675	118005676	INS	chr4_117618738_117620141	2.75918667	11.7005939	2.5985E-06	0.00588945	H3K27ac	BF
chr4	118083331	118083332	INS	chr4_117618738_117620141	2.75918667	11.7005939	2.5985E-06	0.00588945	H3K27ac	BF
chr1	8356436	8356437	INS	chr1_8202749_8204142	5.80197313	11.7043987	2.5921E-06	0.00588945	H3K27ac	BF
chr1	11626419	11626420	INS	chr1_12055701_12056117	3.16795169	11.6938371	2.6099E-06	0.00590178	H3K27ac	BF
chr5	52398267	52398268	INS	chr5_51977059_51978517	1.12724939	11.6778701	2.6372E-06	0.00595883	H3K27ac	BF

chr5	68656687	68656775	DEL	chr5_68721264_68721869	1.54333958	11.6375112	2.7075E-06	0.00610367	H3K27ac	BF
chr5	68242387	68242388	INS	chr5_68721264_68721869	1.54333958	11.6375112	2.7075E-06	0.00610367	H3K27ac	BF
chr5	68747078	68747079	INS	chr5_68721264_68721869	1.54333958	11.6375112	2.7075E-06	0.00610367	H3K27ac	BF
chr5	62540128	62540433	DEL	chr5_62201377_62202042	5.83620813	11.6133409	2.7506E-06	0.00619611	H3K27ac	BF
chr1	104052039	104052552	DEL	chr1_103793767_103795435	-5.1684425	-11.612042	2.7529E-06	0.00619665	H3K27ac	BF
chr5	63962538	63962539	INS	chr5_64159499_64159883	1.96172728	11.6106943	2.7553E-06	0.0061974	H3K27ac	BF
chr1	6669966	6669967	INS	chr1_6961724_6963549	3.58489167	11.606967	2.7621E-06	0.0062078	H3K27ac	BF
chr5	47270892	47270956	DEL	chr5_47699295_47699700	8.15497222	11.5867549	2.7989E-06	0.00628097	H3K27ac	BF
chr5	47503686	47503687	INS	chr5_47699295_47699700	8.15497222	11.5867549	2.7989E-06	0.00628097	H3K27ac	BF
chr5	73979409	73979468	DEL	chr5_74090759_74091501	2.74251219	11.5832979	2.8052E-06	0.00629044	H3K27ac	BF
chr5	40186785	40186834	DEL	chr5_40189937_40191046	4.38691601	11.5628026	2.8432E-06	0.00635149	H3K27ac	BF
chr5	40194281	40194330	DEL	chr5_40189937_40191046	4.38691601	11.5628026	2.8432E-06	0.00635149	H3K27ac	BF
chr1	108733962	108734028	DEL	chr1_108617012_108617284	2.216149	11.5657596	2.8377E-06	0.00635149	H3K27ac	BF

chr5	40181920	40181921	INS	chr5_40189937_40191046	4.38691601	11.5628026	2.8432E-06	0.00635149	H3K27ac	BF
chr2	48397735	48398192	DEL	chr2_48425406_48426454	3.96890964	11.5572745	2.8536E-06	0.00636016	H3K27ac	BF
chr1	205959403	205959404	INS	chr1_205705103_205707014	2.81177238	11.5590627	2.8502E-06	0.00636016	H3K27ac	BF
chr2	48399146	48399147	INS	chr2_48425406_48426454	3.96890964	11.5572745	2.8536E-06	0.00636016	H3K27ac	BF
chr1	252516114	252516183	DEL	chr1_252244453_252244912	4.65749514	11.5399551	2.8862E-06	0.00641846	H3K27ac	BF
chr1	252456578	252456579	INS	chr1_252244453_252244912	4.65749514	11.5399551	2.8862E-06	0.00641846	H3K27ac	BF
chr1	252514025	252514026	INS	chr1_252244453_252244912	4.65749514	11.5399551	2.8862E-06	0.00641846	H3K27ac	BF
chr2	48397735	48398192	DEL	chr2_48703452_48704634	1.06122493	11.5267741	2.9114E-06	0.00646465	H3K27ac	BF
chr2	48399146	48399147	INS	chr2_48703452_48704634	1.06122493	11.5267741	2.9114E-06	0.00646465	H3K27ac	BF
chr4	34144107	34144326	DEL	chr4_34452169_34453278	1.58268939	11.5209043	2.9227E-06	0.00646539	H3K27ac	BF
chr4	34183722	34183833	DEL	chr4_34452169_34453278	1.58268939	11.5209043	2.9227E-06	0.00646539	H3K27ac	BF
chr4	34189745	34190024	DEL	chr4_34452169_34453278	1.58268939	11.5209043	2.9227E-06	0.00646539	H3K27ac	BF
chr4	34178074	34178075	INS	chr4_34452169_34453278	1.58268939	11.5209043	2.9227E-06	0.00646539	H3K27ac	BF

chr5	7581505	7581559	DEL	chr5_8055216_8055936	5.58054063	11.5068176	2.9499E-06	0.00651107	H3K27ac	BF
chr5	7579345	7579621	DEL	chr5_8055216_8055936	5.58054063	11.5068176	2.9499E-06	0.00651107	H3K27ac	BF
chr4	103324840	103325032	DEL	chr4_103178412_103179963	5.62350889	11.4802717	3.0021E-06	0.00661137	H3K27ac	BF
chr4	102782001	102782002	INS	chr4_103178412_103179963	5.62350889	11.4802717	3.0021E-06	0.00661137	H3K27ac	BF
chr4	103323208	103323209	INS	chr4_103178412_103179963	5.62350889	11.4802717	3.0021E-06	0.00661137	H3K27ac	BF
chr5	73979409	73979468	DEL	chr5_73486084_73486635	5.31636938	11.4761665	3.0102E-06	0.0066244	H3K27ac	BF
chr1	53410251	53410252	INS	chr1_53441820_53442797	5.30617611	11.4727251	3.0171E-06	0.00663454	H3K27ac	BF
chr3	80224606	80224607	INS	chr3_80311309_80311568	4.08407069	11.4656282	3.0313E-06	0.00664597	H3K27ac	BF
chr3	80379357	80379358	INS	chr3_80311309_80311568	4.08407069	11.4656282	3.0313E-06	0.00664597	H3K27ac	BF
chr3	80479690	80479691	INS	chr3_80311309_80311568	4.08407069	11.4656282	3.0313E-06	0.00664597	H3K27ac	BF
chr3	80799187	80799188	INS	chr3_80311309_80311568	4.08407069	11.4656282	3.0313E-06	0.00664597	H3K27ac	BF
chr4	107389870	107389871	INS	chr4_107786499_107786762	4.82032319	11.4390914	3.085E-06	0.00675528	H3K27ac	BF
chr3	101852520	101852521	INS	chr3_102098144_102099356	5.81921875	11.4376411	3.088E-06	0.00675528	H3K27ac	BF

chr3	101899978	101899979	INS	chr3_102098144_102099356	5.81921875	11.4376411	3.088E-06	0.00675528	H3K27ac	BF
chr1	100773249	100773316	DEL	chr1_100689802_100690212	6.80882106	11.4042017	3.1573E-06	0.00687132	H3K27ac	BF
chr1	100904519	100905589	DEL	chr1_100689802_100690212	6.80882106	11.4042017	3.1573E-06	0.00687132	H3K27ac	BF
chr1	110888425	110888588	DEL	chr1_110892967_110893892	3.35174633	11.404935	3.1558E-06	0.00687132	H3K27ac	BF
chr1	100545021	100545022	INS	chr1_100689802_100690212	6.80882106	11.4042017	3.1573E-06	0.00687132	H3K27ac	BF
chr1	100619569	100619570	INS	chr1_100689802_100690212	6.80882106	11.4042017	3.1573E-06	0.00687132	H3K27ac	BF
chr1	100773516	100773517	INS	chr1_100689802_100690212	6.80882106	11.4042017	3.1573E-06	0.00687132	H3K27ac	BF
chr1	100875848	100875849	INS	chr1_100689802_100690212	6.80882106	11.4042017	3.1573E-06	0.00687132	H3K27ac	BF
chr1	126387564	126398406	DEL	chr1_126431928_126432363	2.59646017	11.3941118	3.1786E-06	0.0069125	H3K27ac	BF
chr3	22347726	22347727	INS	chr3_22725092_22725795	3.40351625	11.3913867	3.1844E-06	0.00691995	H3K27ac	BF
chr5	31001378	31001673	DEL	chr5_30644805_30647007	5.5204375	11.3891456	3.1891E-06	0.00692518	H3K27ac	BF
chr1	9470258	9470259	INS	chr1_9186219_9186927	7.45068444	11.3388023	3.298E-06	0.00715115	H3K27ac	BF
chr1	9600521	9600522	INS	chr1_9186219_9186927	7.45068444	11.3388023	3.298E-06	0.00715115	H3K27ac	BF

chr1	82466104	82466408	DEL	chr1_82901893_82902773	2.68801944	11.3266277	3.325E-06	0.0071675	H3K27ac	BF
chr1	82873127	82873198	DEL	chr1_82901893_82902773	2.68801944	11.3266277	3.325E-06	0.0071675	H3K27ac	BF
chr1	83337752	83337829	DEL	chr1_82901893_82902773	2.68801944	11.3266277	3.325E-06	0.0071675	H3K27ac	BF
chr2	138042221	138042510	DEL	chr2_137588305_137588584	7.73263833	11.3285847	3.3206E-06	0.0071675	H3K27ac	BF
chr1	82429574	82429575	INS	chr1_82901893_82902773	2.68801944	11.3266277	3.325E-06	0.0071675	H3K27ac	BF
chr1	82684939	82684940	INS	chr1_82901893_82902773	2.68801944	11.3266277	3.325E-06	0.0071675	H3K27ac	BF
chr1	82769443	82769444	INS	chr1_82901893_82902773	2.68801944	11.3266277	3.325E-06	0.0071675	H3K27ac	BF
chr2	137795374	137795375	INS	chr2_137588305_137588584	7.73263833	11.3285847	3.3206E-06	0.0071675	H3K27ac	BF
chr3	106915037	106915038	INS	chr3_107234043_107234805	3.797288	11.3200847	3.3396E-06	0.0071937	H3K27ac	BF
chr1	71451857	71459227	DEL	chr1_71620504_71620777	3.97709383	11.2748195	3.4425E-06	0.00740457	H3K27ac	BF
chr1	71347317	71347318	INS	chr1_71620504_71620777	3.97709383	11.2748195	3.4425E-06	0.00740457	H3K27ac	BF
chr3	57861689	57861761	DEL	chr3_58028680_58029035	7.81127667	11.2376929	3.5295E-06	0.00754239	H3K27ac	BF
chr3	57865269	57865748	DEL	chr3_58028680_58029035	7.81127667	11.2376929	3.5295E-06	0.00754239	H3K27ac	BF

chr2	59887809	59887858	DEL	chr2_59507610_59509587	5.85193819	11.2377858	3.5293E-06	0.00754239	H3K27ac	BF
chr2	77609952	77610227	DEL	chr2_77926269_77927367	3.09194583	11.2390912	3.5262E-06	0.00754239	H3K27ac	BF
chr2	77762098	77762170	DEL	chr2_77926269_77927367	3.09194583	11.2390912	3.5262E-06	0.00754239	H3K27ac	BF
chr2	59855238	59855239	INS	chr2_59507610_59509587	5.85193819	11.2377858	3.5293E-06	0.00754239	H3K27ac	BF
chr2	78113963	78113964	INS	chr2_77926269_77927367	3.09194583	11.2390912	3.5262E-06	0.00754239	H3K27ac	BF
chr1	8356436	8356437	INS	chr1_8178803_8179352	5.66584363	11.2424874	3.5182E-06	0.00754239	H3K27ac	BF
chr1	25372708	25372709	INS	chr1_25323499_25323895	3.31314563	11.2403478	3.5232E-06	0.00754239	H3K27ac	BF
chr1	268866273	268866274	INS	chr1_268773192_268775090	-2.4522306	-11.232014	3.5431E-06	0.00756583	H3K27ac	BF
chr1	112963658	112964123	DEL	chr1_113138113_113138604	3.31933894	11.2058094	3.6063E-06	0.00767304	H3K27ac	BF
chr1	112943872	112943873	INS	chr1_113138113_113138604	3.31933894	11.2058094	3.6063E-06	0.00767304	H3K27ac	BF
chr1	113331480	113331481	INS	chr1_113138113_113138604	3.31933894	11.2058094	3.6063E-06	0.00767304	H3K27ac	BF
chr5	20991401	20991402	INS	chr5_21388430_21389644	5.13636806	11.2063342	3.605E-06	0.00767304	H3K27ac	BF
chr5	21229418	21229419	INS	chr5_21388430_21389644	5.13636806	11.2063342	3.605E-06	0.00767304	H3K27ac	BF

chr5	74409749	74409890	DEL	chr5_74866965_74867491	3.78456561	11.1779788	3.6748E-06	0.00779073	H3K27ac	BF
chr5	74875100	74875101	INS	chr5_74866965_74867491	3.78456561	11.1779788	3.6748E-06	0.00779073	H3K27ac	BF
chr4	107389870	107389871	INS	chr4_107791960_107792216	7.77266661	11.1781713	3.6743E-06	0.00779073	H3K27ac	BF
chr2	87378442	87378532	DEL	chr2_87509297_87510196	16.0778039	11.1588748	3.7226E-06	0.00788089	H3K27ac	BF
chr2	87847374	87847375	INS	chr2_87509297_87510196	16.0778039	11.1588748	3.7226E-06	0.00788089	H3K27ac	BF
chr4	71017558	71017841	DEL	chr4_70881528_70882540	1.83083688	11.1575453	3.726E-06	0.00788235	H3K27ac	BF
chr5	27104409	27104563	DEL	chr5_27401330_27401567	3.3813075	11.1512304	3.742E-06	0.00789555	H3K27ac	BF
chr5	27105631	27105911	DEL	chr5_27401330_27401567	3.3813075	11.1512304	3.742E-06	0.00789555	H3K27ac	BF
chr1	11626419	11626420	INS	chr1_11984137_11984518	4.04346688	11.1487553	3.7483E-06	0.00789555	H3K27ac	BF
chr5	26959570	26959571	INS	chr5_27401330_27401567	3.3813075	11.1512304	3.742E-06	0.00789555	H3K27ac	BF
chr1	32231956	32231957	INS	chr1_32396711_32397119	1.91334806	11.137356	3.7774E-06	0.00793424	H3K27ac	BF
chr1	32549279	32549280	INS	chr1_32396711_32397119	1.91334806	11.137356	3.7774E-06	0.00793424	H3K27ac	BF
chr1	32658817	32658818	INS	chr1_32396711_32397119	1.91334806	11.137356	3.7774E-06	0.00793424	H3K27ac	BF

chr1	32661405	32661406	INS	chr1_32396711_32397119	1.91334806	11.137356	3.7774E-06	0.00793424	H3K27ac	BF
chr2	109334690	109334691	INS	chr2_109014671_109015453	3.48087813	11.1127795	3.841E-06	0.00806217	H3K27ac	BF
chr5	64965531	64965602	DEL	chr5_64992534_64993679	4.95873806	11.0881755	3.9059E-06	0.00818097	H3K27ac	BF
chr5	65256809	65256899	DEL	chr5_64992534_64993679	4.95873806	11.0881755	3.9059E-06	0.00818097	H3K27ac	BF
chr5	65423082	65423303	DEL	chr5_64992534_64993679	4.95873806	11.0881755	3.9059E-06	0.00818097	H3K27ac	BF
chr4	111529407	111529590	DEL	chr4_111585150_111585591	3.96938722	11.0648278	3.9687E-06	0.00825382	H3K27ac	BF
chr4	111094333	111094334	INS	chr4_111585150_111585591	3.96938722	11.0648278	3.9687E-06	0.00825382	H3K27ac	BF
chr4	111155923	111155924	INS	chr4_111585150_111585591	3.96938722	11.0648278	3.9687E-06	0.00825382	H3K27ac	BF
chr4	111158124	111158125	INS	chr4_111585150_111585591	3.96938722	11.0648278	3.9687E-06	0.00825382	H3K27ac	BF
chr4	111482116	111482117	INS	chr4_111585150_111585591	3.96938722	11.0648278	3.9687E-06	0.00825382	H3K27ac	BF
chr4	111524672	111524673	INS	chr4_111585150_111585591	3.96938722	11.0648278	3.9687E-06	0.00825382	H3K27ac	BF
chr4	111876285	111876286	INS	chr4_111585150_111585591	3.96938722	11.0648278	3.9687E-06	0.00825382	H3K27ac	BF
chr4	111869970	111869971	INS	chr4_111585150_111585591	3.96938722	11.0648278	3.9687E-06	0.00825382	H3K27ac	BF

chr4	22058694	22058695	INS	chr4_21968304_21969142	4.47282889	11.0225646	4.0851E-06	0.00849001	H3K27ac	BF
chr1	100095611	100096160	DEL	chr1_100595072_100595472	3.2227615	10.9962862	4.1594E-06	0.00859608	H3K27ac	BF
chr1	100773249	100773316	DEL	chr1_100595072_100595472	3.2227615	10.9962862	4.1594E-06	0.00859608	H3K27ac	BF
chr1	100904519	100905589	DEL	chr1_100595072_100595472	3.2227615	10.9962862	4.1594E-06	0.00859608	H3K27ac	BF
chr1	100545021	100545022	INS	chr1_100595072_100595472	3.2227615	10.9962862	4.1594E-06	0.00859608	H3K27ac	BF
chr1	100619569	100619570	INS	chr1_100595072_100595472	3.2227615	10.9962862	4.1594E-06	0.00859608	H3K27ac	BF
chr1	100773516	100773517	INS	chr1_100595072_100595472	3.2227615	10.9962862	4.1594E-06	0.00859608	H3K27ac	BF
chr1	100875848	100875849	INS	chr1_100595072_100595472	3.2227615	10.9962862	4.1594E-06	0.00859608	H3K27ac	BF
chr2	126596215	126596496	DEL	chr2_127011191_127011769	2.20085088	10.9923481	4.1707E-06	0.00861333	H3K27ac	BF
chr4	122853009	122853218	DEL	chr4_123160759_123161485	6.35259056	10.9868722	4.1864E-06	0.00861568	H3K27ac	BF
chr4	122888381	122890004	DEL	chr4_123160759_123161485	6.35259056	10.9868722	4.1864E-06	0.00861568	H3K27ac	BF
chr4	122750310	122750311	INS	chr4_123160759_123161485	6.35259056	10.9868722	4.1864E-06	0.00861568	H3K27ac	BF
chr4	123025234	123025235	INS	chr4_123160759_123161485	6.35259056	10.9868722	4.1864E-06	0.00861568	H3K27ac	BF

chr4	123447592	123447593	INS	chr4_123160759_123161485	6.35259056	10.9868722	4.1864E-06	0.00861568	H3K27ac	BF
chr3	74463678	74464416	DEL	chr3_74277621_74278783	3.55851478	10.9765254	4.2163E-06	0.00864707	H3K27ac	BF
chr3	79773741	79773742	INS	chr3_80269352_80270988	4.50401222	10.9773325	4.214E-06	0.00864707	H3K27ac	BF
chr3	80224606	80224607	INS	chr3_80269352_80270988	4.50401222	10.9773325	4.214E-06	0.00864707	H3K27ac	BF
chr3	80379357	80379358	INS	chr3_80269352_80270988	4.50401222	10.9773325	4.214E-06	0.00864707	H3K27ac	BF
chr3	80479690	80479691	INS	chr3_80269352_80270988	4.50401222	10.9773325	4.214E-06	0.00864707	H3K27ac	BF
chr1	26014847	26014848	INS	chr1_25915692_25916159	1.14702856	10.9711779	4.2319E-06	0.00866091	H3K27ac	BF
chr1	26040898	26040899	INS	chr1_25915692_25916159	1.14702856	10.9711779	4.2319E-06	0.00866091	H3K27ac	BF
chr4	111529407	111529590	DEL	chr4_111462576_111462927	8.37301594	10.9506657	4.2921E-06	0.0087117	H3K27ac	BF
chr4	111094333	111094334	INS	chr4_111462576_111462927	8.37301594	10.9506657	4.2921E-06	0.0087117	H3K27ac	BF
chr4	111155923	111155924	INS	chr4_111462576_111462927	8.37301594	10.9506657	4.2921E-06	0.0087117	H3K27ac	BF
chr4	111158124	111158125	INS	chr4_111462576_111462927	8.37301594	10.9506657	4.2921E-06	0.0087117	H3K27ac	BF
chr4	111482116	111482117	INS	chr4_111462576_111462927	8.37301594	10.9506657	4.2921E-06	0.0087117	H3K27ac	BF

chr4	111524672	111524673	INS	chr4_111462576_111462927	8.37301594	10.9506657	4.2921E-06	0.0087117	H3K27ac	BF
chr4	111876285	111876286	INS	chr4_111462576_111462927	8.37301594	10.9506657	4.2921E-06	0.0087117	H3K27ac	BF
chr4	111869970	111869971	INS	chr4_111462576_111462927	8.37301594	10.9506657	4.2921E-06	0.0087117	H3K27ac	BF
chr1	99271613	99271614	INS	chr1_99317044_99317733	4.06993294	10.9541309	4.2818E-06	0.0087117	H3K27ac	BF
chr1	8356436	8356437	INS	chr1_8059480_8059787	7.709899	10.9574615	4.272E-06	0.0087117	H3K27ac	BF
chr4	102076552	102076552	BND	chr4_101868346_101869211	6.69862639	10.9420577	4.3176E-06	0.00875755	H3K27ac	BF
chr5	73979409	73979468	DEL	chr5_74454040_74454572	2.63125556	10.9281047	4.3594E-06	0.00883623	H3K27ac	BF
chr5	62540128	62540433	DEL	chr5_62405579_62406261	1.50483413	10.925916	4.366E-06	0.00883642	H3K27ac	BF
chr5	74409749	74409890	DEL	chr5_74843380_74843622	6.16821678	10.9231152	4.3745E-06	0.00883642	H3K27ac	BF
chr5	74875100	74875101	INS	chr5_74843380_74843622	6.16821678	10.9231152	4.3745E-06	0.00883642	H3K27ac	BF
chr5	7581505	7581559	DEL	chr5_7867461_7868005	14.0674164	10.9174894	4.3915E-06	0.00885872	H3K27ac	BF
chr5	7579345	7579621	DEL	chr5_7867461_7868005	14.0674164	10.9174894	4.3915E-06	0.00885872	H3K27ac	BF
chr4	102884126	102885737	DEL	chr4_103178412_103179963	5.64130188	10.9113529	4.4102E-06	0.00888425	H3K27ac	BF

chr2	140930545	140930734	DEL	chr2_140853296_140854016	2.563569	10.895918	4.4575E-06	0.00893532	H3K27ac	BF
chr4	15771261	15771351	DEL	chr4_16131567_16132896	5.28345347	10.897795	4.4518E-06	0.00893532	H3K27ac	BF
chr4	16500467	16500559	DEL	chr4_16131567_16132896	5.28345347	10.897795	4.4518E-06	0.00893532	H3K27ac	BF
chr4	32209579	32210486	DEL	chr4_31875525_31876964	3.70422361	10.895207	4.4597E-06	0.00893532	H3K27ac	BF
chr4	15978130	15978131	INS	chr4_16131567_16132896	5.28345347	10.897795	4.4518E-06	0.00893532	H3K27ac	BF
chr4	15997390	15997391	INS	chr4_16131567_16132896	5.28345347	10.897795	4.4518E-06	0.00893532	H3K27ac	BF
chr4	31529406	31529407	INS	chr4_31875525_31876964	3.70422361	10.895207	4.4597E-06	0.00893532	H3K27ac	BF
chr4	31823934	31823935	INS	chr4_31875525_31876964	3.70422361	10.895207	4.4597E-06	0.00893532	H3K27ac	BF
chr2	89340281	89340351	DEL	chr2_89596736_89597118	3.91412979	10.8770658	4.5162E-06	0.00902387	H3K27ac	BF
chr2	89364651	89364734	DEL	chr2_89596736_89597118	3.91412979	10.8770658	4.5162E-06	0.00902387	H3K27ac	BF
chr2	89390169	89390170	INS	chr2_89596736_89597118	3.91412979	10.8770658	4.5162E-06	0.00902387	H3K27ac	BF
chr2	89461252	89461253	INS	chr2_89596736_89597118	3.91412979	10.8770658	4.5162E-06	0.00902387	H3K27ac	BF
chr1	129061546	129061547	INS	chr1_128701435_128702149	-26.79485	-10.872502	4.5305E-06	0.009029	H3K27ac	BF

chr1	11626419	11626420	INS	chr1_11982950_11983303	3.93541016	10.8713783	4.534E-06	0.009029	H3K27ac	BF
chr1	19331363	19331364	INS	chr1_19081248_19082495	3.77259556	10.8714354	4.5338E-06	0.009029	H3K27ac	BF
chr2	5033423	5033552	DEL	chr2_4545060_4546034	4.14435833	10.8674344	4.5464E-06	0.00903547	H3K27ac	BF
chr5	70607550	70607551	INS	chr5_70658508_70660792	-2.2441413	-10.863036	4.5603E-06	0.00905092	H3K27ac	BF
chr5	71142449	71142450	INS	chr5_70658508_70660792	-2.2441413	-10.863036	4.5603E-06	0.00905092	H3K27ac	BF
chr1	6460049	6460050	INS	chr1_6842857_6843233	3.52836094	10.8539731	4.5891E-06	0.00909584	H3K27ac	BF
chr1	6643056	6643057	INS	chr1_6842857_6843233	3.52836094	10.8539731	4.5891E-06	0.00909584	H3K27ac	BF
chr2	48397735	48398192	DEL	chr2_48201620_48202939	2.15343429	10.8498457	4.6023E-06	0.00910974	H3K27ac	BF
chr2	48399146	48399147	INS	chr2_48201620_48202939	2.15343429	10.8498457	4.6023E-06	0.00910974	H3K27ac	BF
chr2	91361216	91361217	INS	chr2_91716348_91716650	6.29697167	10.8469555	4.6116E-06	0.00911584	H3K27ac	BF
chr2	92213697	92213698	INS	chr2_91716348_91716650	6.29697167	10.8469555	4.6116E-06	0.00911584	H3K27ac	BF
chr1	126387564	126398406	DEL	chr1_126541098_126543573	9.89812444	10.8458759	4.615E-06	0.00911659	H3K27ac	BF
chr5	7581505	7581559	DEL	chr5_7862160_7862982	11.4502874	10.8432286	4.6235E-06	0.00912118	H3K27ac	BF

chr5	7579345	7579621	DEL	chr5_7862160_7862982	11.4502874	10.8432286	4.6235E-06	0.00912118	H3K27ac	BF
chr1	129061546	129061547	INS	chr1_128908304_128908518	-24.988435	-10.840154	4.6334E-06	0.00913461	H3K27ac	BF
chr5	10662712	10662713	INS	chr5_10827642_10827988	5.2070259	10.8276919	4.6738E-06	0.00920189	H3K27ac	BF
chr5	10966160	10966161	INS	chr5_10827642_10827988	5.2070259	10.8276919	4.6738E-06	0.00920189	H3K27ac	BF
chr1	129061546	129061547	INS	chr1_129266489_129267158	-50.925872	-10.824168	4.6853E-06	0.00921836	H3K27ac	BF
chr1	205959403	205959404	INS	chr1_205634692_205634994	5.41813664	10.8088478	4.7356E-06	0.00931111	H3K27ac	BF
chr2	24518579	24518663	DEL	chr2_24645898_24648669	3.00227892	10.8065163	4.7433E-06	0.00931386	H3K27ac	BF
chr2	24784421	24784422	INS	chr2_24645898_24648669	3.00227892	10.8065163	4.7433E-06	0.00931386	H3K27ac	BF
chr1	20834452	20834453	INS	chr1_20706583_20707009	7.61895547	10.8047585	4.7491E-06	0.00931506	H3K27ac	BF
chr1	24236404	24236405	INS	chr1_23762501_23763012	4.8823591	10.8044264	4.7502E-06	0.00931506	H3K27ac	BF
chr5	73979409	73979468	DEL	chr5_73546091_73546768	11.7804568	10.8003066	4.7639E-06	0.00931713	H3K27ac	BF
chr1	18874985	18875191	DEL	chr1_19081248_19082495	3.79451375	10.80039	4.7636E-06	0.00931713	H3K27ac	BF
chr1	15339093	15339094	INS	chr1_15319616_15320030	3.19777133	10.8017143	4.7592E-06	0.00931713	H3K27ac	BF

chr2	18359642	18359643	INS	chr2_18128002_18128355	5.632968	10.8012097	4.7609E-06	0.00931713	H3K27ac	BF
chr3	58943129	58943333	DEL	chr3_58921622_58921897	2.90412389	10.7863841	4.8104E-06	0.00932177	H3K27ac	BF
chr2	126596215	126596496	DEL	chr2_126911114_126911961	10.7075098	10.7961614	4.7777E-06	0.00932177	H3K27ac	BF
chr4	858640	858787	DEL	chr4_1325143_1325499	0.70895007	10.7854391	4.8136E-06	0.00932177	H3K27ac	BF
chr4	1045729	1045874	DEL	chr4_1325143_1325499	0.70895007	10.7854391	4.8136E-06	0.00932177	H3K27ac	BF
chr4	1078391	1078566	DEL	chr4_1325143_1325499	0.70895007	10.7854391	4.8136E-06	0.00932177	H3K27ac	BF
chr4	1604180	1604246	DEL	chr4_1325143_1325499	0.70895007	10.7854391	4.8136E-06	0.00932177	H3K27ac	BF
chr4	1606541	1606624	DEL	chr4_1325143_1325499	0.70895007	10.7854391	4.8136E-06	0.00932177	H3K27ac	BF
chr3	59038914	59038915	INS	chr3_58921622_58921897	2.90412389	10.7863841	4.8104E-06	0.00932177	H3K27ac	BF
chr4	1125076	1125077	INS	chr4_1325143_1325499	0.70895007	10.7854391	4.8136E-06	0.00932177	H3K27ac	BF
chr4	1099047	1099048	INS	chr4_1325143_1325499	0.70895007	10.7854391	4.8136E-06	0.00932177	H3K27ac	BF
chr4	1191085	1191086	INS	chr4_1325143_1325499	0.70895007	10.7854391	4.8136E-06	0.00932177	H3K27ac	BF
chr4	1350135	1350136	INS	chr4_1325143_1325499	0.70895007	10.7854391	4.8136E-06	0.00932177	H3K27ac	BF

chr1	20834452	20834453	INS	chr1_20719924_20720168	12.5870477	10.7889525	4.8018E-06	0.00932177	H3K27ac	BF
chr1	20834452	20834453	INS	chr1_20771081_20771578	10.3174901	10.7925432	4.7898E-06	0.00932177	H3K27ac	BF
chr1	11626419	11626420	INS	chr1_12066026_12066188	17.5863592	10.7734838	4.854E-06	0.00938155	H3K27ac	BF
chr3	53936080	53936081	INS	chr3_54377397_54378374	3.00150406	10.7671465	4.8756E-06	0.00939863	H3K27ac	BF
chr3	54034033	54034034	INS	chr3_54377397_54378374	3.00150406	10.7671465	4.8756E-06	0.00939863	H3K27ac	BF
chr3	54284726	54284727	INS	chr3_54377397_54378374	3.00150406	10.7671465	4.8756E-06	0.00939863	H3K27ac	BF
chr3	54635397	54635398	INS	chr3_54377397_54378374	3.00150406	10.7671465	4.8756E-06	0.00939863	H3K27ac	BF
chr1	20834452	20834453	INS	chr1_20795526_20795849	14.4578722	10.7659382	4.8797E-06	0.00940045	H3K27ac	BF
chr5	62540128	62540433	DEL	chr5_62148856_62150723	2.887325	10.7576891	4.908E-06	0.00941938	H3K27ac	BF
chr5	67677034	67677243	DEL	chr5_68011352_68011721	7.60495956	10.7548325	4.9178E-06	0.00941938	H3K27ac	BF
chr2	114530061	114532178	DEL	chr2_114699831_114700551	2.34329822	10.7579579	4.907E-06	0.00941938	H3K27ac	BF
chr1	9913068	9913215	DEL	chr1_10073192_10073783	7.74881	10.7547035	4.9182E-06	0.00941938	H3K27ac	BF
chr5	67641407	67641408	INS	chr5_68011352_68011721	15.2099191	10.7548325	4.9178E-06	0.00941938	H3K27ac	BF

chr5	67741227	67741228	INS	chr5_68011352_68011721	7.60495956	10.7548325	4.9178E-06	0.00941938	H3K27ac	BF
chr5	68212673	68212674	INS	chr5_68011352_68011721	7.60495956	10.7548325	4.9178E-06	0.00941938	H3K27ac	BF
chr2	114533753	114533754	INS	chr2_114699831_114700551	2.34329822	10.7579579	4.907E-06	0.00941938	H3K27ac	BF
chr1	8356436	8356437	INS	chr1_8266739_8267925	7.08777344	10.758594	4.9049E-06	0.00941938	H3K27ac	BF
chr1	129061546	129061547	INS	chr1_128700664_128700922	-30.716216	-10.746002	4.9483E-06	0.00947085	H3K27ac	BF
chr1	252516114	252516183	DEL	chr1_252138156_252139384	4.51201889	10.740831	4.9663E-06	0.00948681	H3K27ac	BF
chr1	252456578	252456579	INS	chr1_252138156_252139384	4.51201889	10.740831	4.9663E-06	0.00948681	H3K27ac	BF
chr1	252514025	252514026	INS	chr1_252138156_252139384	4.51201889	10.740831	4.9663E-06	0.00948681	H3K27ac	BF
chr4	108969013	108969014	INS	chr4_108626948_108627560	3.85509317	10.7328857	4.994E-06	0.00953367	H3K27ac	BF
chr1	11626419	11626420	INS	chr1_11939202_11940590	3.55008594	10.7248132	5.0224E-06	0.00956929	H3K27ac	BF
chr5	13941115	13941116	INS	chr5_14076206_14076718	5.56413611	10.7119297	5.0681E-06	0.00962526	H3K27ac	BF
chr5	14116104	14116105	INS	chr5_14076206_14076718	5.56413611	10.7119297	5.0681E-06	0.00962526	H3K27ac	BF
chr5	14186300	14186301	INS	chr5_14076206_14076718	5.56413611	10.7119297	5.0681E-06	0.00962526	H3K27ac	BF

chr5	14416409	14416410	INS	chr5_14076206_14076718	5.56413611	10.7119297	5.0681E-06	0.00962526	H3K27ac	BF
chr1	8356436	8356437	INS	chr1_8767126_8767285	15.6982774	10.7086661	5.0797E-06	0.00962859	H3K27ac	BF
chr1	11626419	11626420	INS	chr1_11675919_11676837	7.00131797	10.7077825	5.0829E-06	0.00962859	H3K27ac	BF
chr1	107526690	107526691	INS	chr1_107360297_107360886	13.8161175	10.6991213	5.114E-06	0.00968123	H3K27ac	BF
chr4	20751112	20751113	INS	chr4_20905689_20906274	9.68315994	10.6939804	5.1325E-06	0.0097101	H3K27ac	BF
chr2	65760781	65760782	INS	chr2_65636013_65637751	1.97651208	10.6930593	5.1358E-06	0.00971018	H3K27ac	BF
chr1	11626419	11626420	INS	chr1_11835362_11835895	12.4368873	10.6847678	5.1659E-06	0.00974833	H3K27ac	BF
chr1	188641399	188641400	INS	chr1_188277381_188277681	3.71978078	10.6778151	5.1913E-06	0.00978995	H3K27ac	BF
chr1	37492757	37493579	DEL	chr1_37654816_37655076	4.33468806	10.6481386	5.3012E-06	0.00995902	H3K27ac	BF
chr2	48297275	48297336	DEL	chr2_48624336_48626471	-5.233105	-10.648273	5.3007E-06	0.00995902	H3K27ac	BF
chr1	37718951	37718952	INS	chr1_37654816_37655076	4.33468806	10.6481386	5.3012E-06	0.00995902	H3K27ac	BF
chr1	38023171	38023172	INS	chr1_37654816_37655076	4.33468806	10.6481386	5.3012E-06	0.00995902	H3K27ac	BF
chr1	38125169	38125170	INS	chr1_37654816_37655076	4.33468806	10.6481386	5.3012E-06	0.00995902	H3K27ac	BF

chr1	38094609	38094610	INS	chr1_37654816_37655076	4.33468806	10.6481386	5.3012E-06	0.00995902	H3K27ac	BF
chr2	13583454	13583455	INS	chr2_13205793_13207626	3.52322083	10.6410625	5.3278E-06	0.0100026	H3K27ac	BF
chr1	20834452	20834453	INS	chr1_20605949_20607180	7.94329992	10.6348978	5.351E-06	0.01003993	H3K27ac	BF
chr2	77609952	77610227	DEL	chr2_77861180_77861994	9.91825833	10.6313584	5.3644E-06	0.01004595	H3K27ac	BF
chr2	77762098	77762170	DEL	chr2_77861180_77861994	9.91825833	10.6313584	5.3644E-06	0.01004595	H3K27ac	BF
chr2	78113963	78113964	INS	chr2_77861180_77861994	9.91825833	10.6313584	5.3644E-06	0.01004595	H3K27ac	BF
chr5	27104409	27104563	DEL	chr5_27408572_27408932	3.67445972	10.6262276	5.384E-06	0.010057	H3K27ac	BF
chr5	27105631	27105911	DEL	chr5_27408572_27408932	3.67445972	10.6262276	5.384E-06	0.010057	H3K27ac	BF
chr1	20834452	20834453	INS	chr1_21083748_21084003	10.0340422	10.628344	5.3759E-06	0.010057	H3K27ac	BF
chr5	26959570	26959571	INS	chr5_27408572_27408932	3.67445972	10.6262276	5.384E-06	0.010057	H3K27ac	BF
chr1	133443332	133443332	BND	chr1_132968398_132968744	2.308078	10.6242892	5.3914E-06	0.01006444	H3K27ac	BF
chr1	240699894	240699895	INS	chr1_240640847_240641257	13.9141461	10.6205278	5.4057E-06	0.01008491	H3K27ac	BF
chr2	5033423	5033552	DEL	chr2_5036737_5037204	4.91041056	10.615152	5.4264E-06	0.01010404	H3K27ac	BF

chr1	205959403	205959404	INS	chr1_205593205_205593625	13.5459957	10.6162329	5.4222E-06	0.01010404	H3K27ac	BF
chr1	11626419	11626420	INS	chr1_11841526_11841808	6.54362344	10.6134021	5.4331E-06	0.01010404	H3K27ac	BF
chr3	68144528	68144998	DEL	chr3_68174786_68175659	3.23703313	10.6010257	5.481E-06	0.01018028	H3K27ac	BF
chr3	68141059	68142597	INV	chr3_68174786_68175659	6.47406625	10.6010257	5.481E-06	0.01018028	H3K27ac	BF
chr1	8356436	8356437	INS	chr1_8573776_8574034	3.27362869	10.5963005	5.4994E-06	0.01020806	H3K27ac	BF
chr2	48297275	48297336	DEL	chr2_48284069_48284715	14.5684284	10.5881624	5.5313E-06	0.01023504	H3K27ac	BF
NW_018085268.1	50420	50755	DEL	NW_018085268.1_57758_59393	18.4444599	10.5885708	5.5297E-06	0.01023504	H3K27ac	BF
chr1	11626419	11626420	INS	chr1_11608452_11608855	7.45470914	10.5893866	5.5265E-06	0.01023504	H3K27ac	BF
chr4	92757233	92759201	DEL	chr4_93105433_93106033	2.59969067	10.5745318	5.5851E-06	0.01030239	H3K27ac	BF
chr4	92855965	92856268	DEL	chr4_93105433_93106033	2.59969067	10.5745318	5.5851E-06	0.01030239	H3K27ac	BF
chr4	93472430	93472693	DEL	chr4_93105433_93106033	2.59969067	10.5745318	5.5851E-06	0.01030239	H3K27ac	BF
chr4	92728057	92728058	INS	chr4_93105433_93106033	2.59969067	10.5745318	5.5851E-06	0.01030239	H3K27ac	BF
chr4	116550806	116550807	INS	chr4_116942362_116942612	6.12940213	10.5779465	5.5716E-06	0.01030239	H3K27ac	BF

chr2	140930545	140930734	DEL	chr2_140835279_140835808	3.52013417	10.5703029	5.6019E-06	0.01032027	H3K27ac	BF
chr1	11626419	11626420	INS	chr1_12050601_12051013	9.32262844	10.5685846	5.6088E-06	0.01032027	H3K27ac	BF
chr5	7581505	7581559	DEL	chr5_7154318_7155610	1.96481938	10.5636231	5.6286E-06	0.01033744	H3K27ac	BF
chr5	7579345	7579621	DEL	chr5_7154318_7155610	1.96481938	10.5636231	5.6286E-06	0.01033744	H3K27ac	BF
chr3	116548269	116548270	INS	chr3_116480177_116480496	24.9499226	10.5648294	5.6238E-06	0.01033744	H3K27ac	BF
chr1	11626419	11626420	INS	chr1_11585538_11585895	5.15595266	10.560504	5.6411E-06	0.01034113	H3K27ac	BF
chr4	107947139	107947485	DEL	chr4_108374795_108375137	0.99089958	10.556169	5.6586E-06	0.01036023	H3K27ac	BF
chr4	108421866	108422013	DEL	chr4_108374795_108375137	0.99089958	10.556169	5.6586E-06	0.01036023	H3K27ac	BF
chr1	165174699	165175474	DEL	chr1_164801837_164802469	3.30201356	10.5507881	5.6803E-06	0.01036788	H3K27ac	BF
chr5	70607550	70607551	INS	chr5_70222303_70223067	8.236754	10.5527898	5.6722E-06	0.01036788	H3K27ac	BF
chr1	33893966	33893967	INS	chr1_34245080_34245707	2.11140319	10.5510037	5.6794E-06	0.01036788	H3K27ac	BF
chr1	33910107	33910108	INS	chr1_34245080_34245707	2.11140319	10.5510037	5.6794E-06	0.01036788	H3K27ac	BF
chr1	34395298	34395299	INS	chr1_34245080_34245707	2.11140319	10.5510037	5.6794E-06	0.01036788	H3K27ac	BF

chr2	48397735	48398192	DEL	chr2_48611190_48611461	3.41306786	10.5469735	5.6958E-06	0.01038326	H3K27ac	BF
chr2	48399146	48399147	INS	chr2_48611190_48611461	3.41306786	10.5469735	5.6958E-06	0.01038326	H3K27ac	BF
chr5	7581505	7581559	DEL	chr5_7868429_7868665	14.2868547	10.5442144	5.707E-06	0.01039087	H3K27ac	BF
chr5	7579345	7579621	DEL	chr5_7868429_7868665	14.2868547	10.5442144	5.707E-06	0.01039087	H3K27ac	BF
chr1	11626419	11626420	INS	chr1_11849471_11850495	16.4729641	10.5345243	5.7465E-06	0.0104436	H3K27ac	BF
chr2	48297275	48297336	DEL	chr2_47931936_47933267	2.75196805	10.530757	5.762E-06	0.01046527	H3K27ac	BF
chr5	73979409	73979468	DEL	chr5_73710054_73710730	5.26146094	10.5142383	5.8304E-06	0.01058295	H3K27ac	BF
chr1	8356436	8356437	INS	chr1_7969057_7969429	1.93491613	10.5039036	5.8736E-06	0.01065488	H3K27ac	BF
chr3	121657515	121657516	INS	chr3_121805571_121805991	6.21395742	10.4994332	5.8924E-06	0.01067589	H3K27ac	BF
chr5	65414148	65414149	INS	chr5_64992534_64993679	4.63016963	10.4960528	5.9067E-06	0.01069519	H3K27ac	BF
chr1	11626419	11626420	INS	chr1_11787494_11787784	8.11558047	10.4924834	5.9218E-06	0.01070286	H3K27ac	BF
chr4	111529407	111529590	DEL	chr4_111894153_111894881	9.42323911	10.4840445	5.9577E-06	0.01072835	H3K27ac	BF
chr4	112216586	112216758	DEL	chr4_111894153_111894881	9.42323911	10.4840445	5.9577E-06	0.01072835	H3K27ac	BF

chr4	111482116	111482117	INS	chr4_111894153_111894881	9.42323911	10.4840445	5.9577E-06	0.01072835	H3K27ac	BF
chr4	111524672	111524673	INS	chr4_111894153_111894881	9.42323911	10.4840445	5.9577E-06	0.01072835	H3K27ac	BF
chr4	111876285	111876286	INS	chr4_111894153_111894881	9.42323911	10.4840445	5.9577E-06	0.01072835	H3K27ac	BF
chr4	111869970	111869971	INS	chr4_111894153_111894881	9.42323911	10.4840445	5.9577E-06	0.01072835	H3K27ac	BF
chr2	126596215	126596496	DEL	chr2_126861607_126862179	12.0811659	10.4809262	5.971E-06	0.01074579	H3K27ac	BF
chr1	188641399	188641400	INS	chr1_188239905_188240633	5.48600156	10.4765496	5.9898E-06	0.01077299	H3K27ac	BF
chr3	68144528	68144998	DEL	chr3_68258442_68258850	9.15310438	10.4691972	6.0215E-06	0.01081019	H3K27ac	BF
chr3	68141059	68142597	INV	chr3_68258442_68258850	18.3062088	10.4691972	6.0215E-06	0.01081019	H3K27ac	BF
chr3	112152071	112153111	DEL	chr3_112145603_112146079	2.84422444	10.4603338	6.0599E-06	0.01084304	H3K27ac	BF
chr3	112154560	112154639	DEL	chr3_112145603_112146079	2.84422444	10.4603338	6.0599E-06	0.01084304	H3K27ac	BF
chr5	76263409	76263410	INS	chr5_76737764_76738045	2.79626813	10.4565244	6.0765E-06	0.01084304	H3K27ac	BF
chr5	76526152	76526153	INS	chr5_76737764_76738045	2.79626813	10.4565244	6.0765E-06	0.01084304	H3K27ac	BF
chr5	76568601	76568602	INS	chr5_76737764_76738045	2.79626813	10.4565244	6.0765E-06	0.01084304	H3K27ac	BF

chr5	76662168	76662169	INS	chr5_76737764_76738045	2.79626813	10.4565244	6.0765E-06	0.01084304	H3K27ac	BF
chr3	112202635	112202636	INS	chr3_112145603_112146079	2.84422444	10.4603338	6.0599E-06	0.01084304	H3K27ac	BF
chr1	11626419	11626420	INS	chr1_12025001_12025205	6.1963718	10.4593301	6.0642E-06	0.01084304	H3K27ac	BF
chr1	71451857	71459227	DEL	chr1_71779304_71780724	4.12231389	10.4486417	6.111E-06	0.01089145	H3K27ac	BF
chr1	71347317	71347318	INS	chr1_71779304_71780724	4.12231389	10.4486417	6.111E-06	0.01089145	H3K27ac	BF
chr4	71017558	71017841	DEL	chr4_70908679_70909043	5.63706981	10.4412231	6.1436E-06	0.01090082	H3K27ac	BF
chr5	59582012	59582096	DEL	chr5_59932513_59933440	4.29994722	10.4365382	6.1644E-06	0.01090082	H3K27ac	BF
chr5	59945771	59945857	DEL	chr5_59932513_59933440	4.29994722	10.4365382	6.1644E-06	0.01090082	H3K27ac	BF
chr5	76180669	76180826	DEL	chr5_76261383_76261620	2.41502451	10.434337	6.1741E-06	0.01090082	H3K27ac	BF
chr3	5128715	5128915	DEL	chr3_5361004_5362042	1.6590295	10.4239036	6.2207E-06	0.01090082	H3K27ac	BF
chr1	130147620	130147936	DEL	chr1_130004311_130004790	2.18923367	10.4225022	6.227E-06	0.01090082	H3K27ac	BF
chr1	130257701	130257880	DEL	chr1_130004311_130004790	2.18923367	10.4225022	6.227E-06	0.01090082	H3K27ac	BF
chr3	5631002	5631076	DEL	chr3_5361004_5362042	1.6590295	10.4239036	6.2207E-06	0.01090082	H3K27ac	BF

chr5	31001378	31001673	DEL	chr5_30525677_30527552	-2.9184094	-10.434696	6.1726E-06	0.01090082	H3K27ac	BF
chr5	59653873	59653874	INS	chr5_59932513_59933440	4.29994722	10.4365382	6.1644E-06	0.01090082	H3K27ac	BF
chr5	60298343	60298344	INS	chr5_59932513_59933440	4.29994722	10.4365382	6.1644E-06	0.01090082	H3K27ac	BF
chr5	73979058	73979059	INS	chr5_74084510_74084749	1.66673869	10.4413211	6.1432E-06	0.01090082	H3K27ac	BF
chr5	76263409	76263410	INS	chr5_76261383_76261620	2.41502451	10.434337	6.1741E-06	0.01090082	H3K27ac	BF
chr5	76526152	76526153	INS	chr5_76261383_76261620	2.41502451	10.434337	6.1741E-06	0.01090082	H3K27ac	BF
chr5	76568601	76568602	INS	chr5_76261383_76261620	2.41502451	10.434337	6.1741E-06	0.01090082	H3K27ac	BF
chr5	76662168	76662169	INS	chr5_76261383_76261620	2.41502451	10.434337	6.1741E-06	0.01090082	H3K27ac	BF
chr3	5059861	5059862	INS	chr3_5361004_5362042	1.6590295	10.4239036	6.2207E-06	0.01090082	H3K27ac	BF
chr3	5050518	5050519	INS	chr3_5361004_5362042	1.6590295	10.4239036	6.2207E-06	0.01090082	H3K27ac	BF
chr3	5280099	5280100	INS	chr3_5361004_5362042	1.6590295	10.4239036	6.2207E-06	0.01090082	H3K27ac	BF
chr3	5311847	5311848	INS	chr3_5361004_5362042	1.6590295	10.4239036	6.2207E-06	0.01090082	H3K27ac	BF
chr3	5318752	5318753	INS	chr3_5361004_5362042	1.6590295	10.4239036	6.2207E-06	0.01090082	H3K27ac	BF

chr1	129061546	129061547	INS	chr1_129251326_129251839	-21.662729	-10.446062	6.1223E-06	0.01090082	H3K27ac	BF
chr1	129945218	129945219	INS	chr1_130004311_130004790	2.18923367	10.4225022	6.227E-06	0.01090082	H3K27ac	BF
chr3	5549799	5549800	INS	chr3_5361004_5362042	1.6590295	10.4239036	6.2207E-06	0.01090082	H3K27ac	BF
chr3	5492452	5492453	INS	chr3_5361004_5362042	1.6590295	10.4239036	6.2207E-06	0.01090082	H3K27ac	BF
chr3	5495403	5495404	INS	chr3_5361004_5362042	1.6590295	10.4239036	6.2207E-06	0.01090082	H3K27ac	BF
chr3	5503659	5503660	INS	chr3_5361004_5362042	1.6590295	10.4239036	6.2207E-06	0.01090082	H3K27ac	BF
chr3	5841180	5841181	INS	chr3_5361004_5362042	1.6590295	10.4239036	6.2207E-06	0.01090082	H3K27ac	BF
chr3	4922220	4922527	INV	chr3_5361004_5362042	3.318059	10.4239036	6.2207E-06	0.01090082	H3K27ac	BF
chr1	194561673	194561674	INS	chr1_194459620_194460332	4.19027406	10.4163544	6.2546E-06	0.01094271	H3K27ac	BF
chr3	39342385	39342688	DEL	chr3_39672682_39673038	3.606681	10.4108338	6.2795E-06	0.0109715	H3K27ac	BF
chr1	11626419	11626420	INS	chr1_11634678_11634902	4.52525578	10.4082089	6.2914E-06	0.0109715	H3K27ac	BF
chr1	11626419	11626420	INS	chr1_11687096_11688107	2.50627852	10.4069674	6.2971E-06	0.0109715	H3K27ac	BF
chr5	73979409	73979468	DEL	chr5_73941027_73941642	4.09031484	10.3935194	6.3585E-06	0.01106701	H3K27ac	BF

chr3	105019346	105019408	DEL	chr3_105336614_105337044	2.15460125	10.391688	6.3669E-06	0.01106701	H3K27ac	BF
chr3	105570276	105570322	DEL	chr3_105336614_105337044	2.15460125	10.391688	6.3669E-06	0.01106701	H3K27ac	BF
chr3	105048262	105048263	INS	chr3_105336614_105337044	2.15460125	10.391688	6.3669E-06	0.01106701	H3K27ac	BF
chr1	11626419	11626420	INS	chr1_11881769_11882804	9.76535078	10.3859374	6.3934E-06	0.01109348	H3K27ac	BF
chr1	129061546	129061547	INS	chr1_129329145_129329610	-26.902734	-10.379134	6.4249E-06	0.0111416	H3K27ac	BF
chr3	9073155	9073156	INS	chr3_9411664_9412342	1.73082844	10.3780383	6.43E-06	0.01114389	H3K27ac	BF
chr4	71017558	71017841	DEL	chr4_70917675_70917912	12.1008044	10.3704754	6.4652E-06	0.01116465	H3K27ac	BF
chr2	133701012	133701064	DEL	chr2_133617336_133617797	2.53809194	10.365761	6.4873E-06	0.01116465	H3K27ac	BF
chr2	133899189	133902056	DEL	chr2_133617336_133617797	2.53809194	10.365761	6.4873E-06	0.01116465	H3K27ac	BF
chr2	133904101	133904172	DEL	chr2_133617336_133617797	2.53809194	10.365761	6.4873E-06	0.01116465	H3K27ac	BF
chr1	1511558	1511813	DEL	chr1_1817176_1818521	7.7887641	10.3669408	6.4818E-06	0.01116465	H3K27ac	BF
chr1	1697599	1697690	DEL	chr1_1817176_1818521	7.7887641	10.3669408	6.4818E-06	0.01116465	H3K27ac	BF
chr1	2307186	2307261	DEL	chr1_1817176_1818521	7.7887641	10.3669408	6.4818E-06	0.01116465	H3K27ac	BF

chr5	77783286	77783287	INS	chr5_77333586_77333963	3.02619644	10.3694283	6.4701E-06	0.01116465	H3K27ac	BF
chr2	133913002	133913003	INS	chr2_133617336_133617797	2.53809194	10.365761	6.4873E-06	0.01116465	H3K27ac	BF
chr2	133907109	133907110	INS	chr2_133617336_133617797	2.53809194	10.365761	6.4873E-06	0.01116465	H3K27ac	BF
chr2	133904244	133904245	INS	chr2_133617336_133617797	2.53809194	10.365761	6.4873E-06	0.01116465	H3K27ac	BF
chr1	1779044	1779045	INS	chr1_1817176_1818521	7.7887641	10.3669408	6.4818E-06	0.01116465	H3K27ac	BF
chr1	129061546	129061547	INS	chr1_129472604_129473670	-13.656837	-10.346822	6.5769E-06	0.01130565	H3K27ac	BF
chr1	8356436	8356437	INS	chr1_8831786_8832074	7.53924063	10.3469989	6.5761E-06	0.01130565	H3K27ac	BF
NW_018085268.1	50420	50755	DEL	NW_018085268.1_109742_110050	22.0755536	10.3446313	6.5873E-06	0.01131255	H3K27ac	BF
chr1	11626419	11626420	INS	chr1_11873564_11873800	9.54098039	10.3427724	6.5962E-06	0.01131255	H3K27ac	BF
chr5	73979409	73979468	DEL	chr5_74115270_74115441	1.69229125	10.3410677	6.6044E-06	0.01131996	H3K27ac	BF
chr3	38117442	38117516	DEL	chr3_38075710_38075933	5.13594128	10.3230982	6.6911E-06	0.01145403	H3K27ac	BF
chr1	11626419	11626420	INS	chr1_11760753_11761563	4.33581797	10.3208509	6.702E-06	0.01145403	H3K27ac	BF
chr3	38554703	38554704	INS	chr3_38075710_38075933	5.13594128	10.3230982	6.6911E-06	0.01145403	H3K27ac	BF

chr5	73979409	73979468	DEL	chr5_74437314_74438158	2.56364819	10.3129377	6.7406E-06	0.01151341	H3K27ac	BF
chr4	116550806	116550807	INS	chr4_116886603_116887290	3.65666894	10.3070784	6.7694E-06	0.01155588	H3K27ac	BF
chr2	48297275	48297336	DEL	chr2_48778739_48779007	3.9796482	10.2968713	6.8199E-06	0.01163528	H3K27ac	BF
chr4	126924760	126925063	DEL	chr4_127392558_127393162	1.37594124	10.2958153	6.8251E-06	0.01163751	H3K27ac	BF
chr2	59887809	59887858	DEL	chr2_59988009_59988635	10.9071063	10.288455	6.8618E-06	0.01168012	H3K27ac	BF
chr4	122853009	122853218	DEL	chr4_123130865_123131308	1.70506333	10.2828915	6.8897E-06	0.01168012	H3K27ac	BF
chr4	122888381	122890004	DEL	chr4_123130865_123131308	1.70506333	10.2828915	6.8897E-06	0.01168012	H3K27ac	BF
chr2	59855238	59855239	INS	chr2_59988009_59988635	10.9071063	10.288455	6.8618E-06	0.01168012	H3K27ac	BF
chr4	122750310	122750311	INS	chr4_123130865_123131308	1.70506333	10.2828915	6.8897E-06	0.01168012	H3K27ac	BF
chr4	123025234	123025235	INS	chr4_123130865_123131308	1.70506333	10.2828915	6.8897E-06	0.01168012	H3K27ac	BF
chr4	123447592	123447593	INS	chr4_123130865_123131308	1.70506333	10.2828915	6.8897E-06	0.01168012	H3K27ac	BF
chr1	11626419	11626420	INS	chr1_11893800_11894211	6.63460313	10.2835539	6.8863E-06	0.01168012	H3K27ac	BF
chr1	20834452	20834453	INS	chr1_20583149_20583333	5.00735938	10.2814974	6.8967E-06	0.01168529	H3K27ac	BF

chr4	70856707	70856708	INS	chr4_71153867_71154236	0.96561146	10.272979	6.9396E-06	0.01173788	H3K27ac	BF
chr4	71085769	71085770	INS	chr4_71153867_71154236	0.96561146	10.272979	6.9396E-06	0.01173788	H3K27ac	BF
chr4	71282290	71282291	INS	chr4_71153867_71154236	0.96561146	10.272979	6.9396E-06	0.01173788	H3K27ac	BF
chr1	230548938	230548939	INS	chr1_230884523_230884950	5.34287778	10.2682824	6.9634E-06	0.01177142	H3K27ac	BF
chr4	9697334	9697335	INS	chr4_10137374_10137957	6.1556315	10.2555673	7.0284E-06	0.01187439	H3K27ac	BF
chr5	51380539	51380540	INS	chr5_51596560_51596873	3.03346622	10.2527054	7.0431E-06	0.01189243	H3K27ac	BF
chr1	205959403	205959404	INS	chr1_205803087_205803682	10.5318638	10.2454181	7.0807E-06	0.0119491	H3K27ac	BF
chr4	69763557	69763558	INS	chr4_69385250_69385642	4.01846083	10.2389935	7.114E-06	0.01199166	H3K27ac	BF
chr4	69772223	69772224	INS	chr4_69385250_69385642	4.01846083	10.2389935	7.114E-06	0.01199166	H3K27ac	BF
chr1	129061546	129061547	INS	chr1_129464167_129464412	-16.891158	-10.219077	7.2185E-06	0.01216084	H3K27ac	BF
chr2	12410503	12410504	INS	chr2_12487920_12490492	19.1246492	10.2177042	7.2258E-06	0.01216613	H3K27ac	BF
chr1	190223373	190223374	INS	chr1_190587465_190587737	4.16921727	10.2006164	7.3169E-06	0.01231252	H3K27ac	BF
chr1	269907677	269907830	DEL	chr1_270400910_270401295	1.9539	10.1875688	7.3873E-06	0.01240982	H3K27ac	BF

chr1	270007327	270007521	DEL	chr1_270400910_270401295	1.9539	10.1875688	7.3873E-06	0.01240982	H3K27ac	BF
chr1	270587310	270587487	DEL	chr1_270400910_270401295	1.9539	10.1875688	7.3873E-06	0.01240982	H3K27ac	BF
chr5	23006530	23006531	INS	chr5_22514038_22514555	4.42246181	10.180852	7.4239E-06	0.01246413	H3K27ac	BF
chr1	82284385	82284471	DEL	chr1_82043058_82043870	1.55754861	10.1656028	7.5076E-06	0.01251931	H3K27ac	BF
chr1	82393824	82394514	DEL	chr1_82043058_82043870	1.55754861	10.1656028	7.5076E-06	0.01251931	H3K27ac	BF
chr1	82466104	82466408	DEL	chr1_82043058_82043870	1.55754861	10.1656028	7.5076E-06	0.01251931	H3K27ac	BF
chr1	81760214	81760215	INS	chr1_82043058_82043870	1.55754861	10.1656028	7.5076E-06	0.01251931	H3K27ac	BF
chr1	81834343	81834344	INS	chr1_82043058_82043870	1.55754861	10.1656028	7.5076E-06	0.01251931	H3K27ac	BF
chr1	81854676	81854677	INS	chr1_82043058_82043870	1.55754861	10.1656028	7.5076E-06	0.01251931	H3K27ac	BF
chr1	81830283	81830284	INS	chr1_82043058_82043870	1.55754861	10.1656028	7.5076E-06	0.01251931	H3K27ac	BF
chr1	81936658	81936659	INS	chr1_82043058_82043870	1.55754861	10.1656028	7.5076E-06	0.01251931	H3K27ac	BF
chr1	82222210	82222211	INS	chr1_82043058_82043870	1.55754861	10.1656028	7.5076E-06	0.01251931	H3K27ac	BF
chr1	82396020	82396021	INS	chr1_82043058_82043870	1.55754861	10.1656028	7.5076E-06	0.01251931	H3K27ac	BF

chr1	82429574	82429575	INS	chr1_82043058_82043870	1.55754861	10.1656028	7.5076E-06	0.01251931	H3K27ac	BF
chr1	112842193	112842194	INS	chr1_113017480_113018892	3.59707639	10.1691888	7.4878E-06	0.01251931	H3K27ac	BF
chr5	7581505	7581559	DEL	chr5_7178244_7179138	4.45313438	10.1617115	7.5291E-06	0.01254106	H3K27ac	BF
chr5	7579345	7579621	DEL	chr5_7178244_7179138	4.45313438	10.1617115	7.5291E-06	0.01254106	H3K27ac	BF
chr2	127144929	127144930	INS	chr2_126732834_126733635	3.40174294	10.147796	7.6067E-06	0.01264889	H3K27ac	BF
chr2	127156523	127156524	INS	chr2_126732834_126733635	3.40174294	10.147796	7.6067E-06	0.01264889	H3K27ac	BF
chr5	68763881	68763943	DEL	chr5_69106948_69107115	3.01267986	10.1378121	7.6629E-06	0.01269234	H3K27ac	BF
chr5	69066214	69066419	DEL	chr5_69106948_69107115	3.01267986	10.1378121	7.6629E-06	0.01269234	H3K27ac	BF
chr5	68692912	68692913	INS	chr5_69106948_69107115	3.01267986	10.1378121	7.6629E-06	0.01269234	H3K27ac	BF
chr5	68899638	68899639	INS	chr5_69106948_69107115	3.01267986	10.1378121	7.6629E-06	0.01269234	H3K27ac	BF
chr5	69097478	69097479	INS	chr5_69106948_69107115	3.01267986	10.1378121	7.6629E-06	0.01269234	H3K27ac	BF
chr5	69277585	69277586	INS	chr5_69106948_69107115	3.01267986	10.1378121	7.6629E-06	0.01269234	H3K27ac	BF
chr5	69320646	69320647	INS	chr5_69106948_69107115	3.01267986	10.1378121	7.6629E-06	0.01269234	H3K27ac	BF

chr1	8356436	8356437	INS	chr1_8566769_8567103	2.05724813	10.1284034	7.7163E-06	0.01277362	H3K27ac	BF
chr5	73979409	73979468	DEL	chr5_73620931_73621747	2.94816813	10.1266391	7.7264E-06	0.0127831	H3K27ac	BF
chr1	20834452	20834453	INS	chr1_20848379_20848553	4.7108575	10.1243459	7.7395E-06	0.0127976	H3K27ac	BF
chr1	129061546	129061547	INS	chr1_129558223_129560150	-6.5315075	-10.123398	7.7449E-06	0.0127994	H3K27ac	BF
chr1	58158194	58158195	INS	chr1_57807238_57807601	3.38882344	10.1226047	7.7495E-06	0.01279974	H3K27ac	BF
chr2	5033423	5033552	DEL	chr2_4752890_4753286	4.95536111	10.1204173	7.762E-06	0.01281328	H3K27ac	BF
chr4	42828063	42829444	DEL	chr4_42973701_42974919	2.38208056	10.1179375	7.7762E-06	0.01282961	H3K27ac	BF
chr3	110348309	110348598	DEL	chr3_110328180_110328689	3.72049322	10.1066991	7.8411E-06	0.0128791	H3K27ac	BF
chr3	110403509	110403559	DEL	chr3_110328180_110328689	3.72049322	10.1066991	7.8411E-06	0.0128791	H3K27ac	BF
chr3	110754806	110756967	DEL	chr3_110328180_110328689	3.72049322	10.1066991	7.8411E-06	0.0128791	H3K27ac	BF
chr3	110756851	110757132	DEL	chr3_110328180_110328689	3.72049322	10.1066991	7.8411E-06	0.0128791	H3K27ac	BF
chr3	110101328	110101329	INS	chr3_110328180_110328689	3.72049322	10.1066991	7.8411E-06	0.0128791	H3K27ac	BF
chr3	110206678	110206679	INS	chr3_110328180_110328689	3.72049322	10.1066991	7.8411E-06	0.0128791	H3K27ac	BF

chr3	110739096	110739097	INS	chr3_110328180_110328689	3.72049322	10.1066991	7.8411E-06	0.0128791	H3K27ac	BF
chr3	110698177	110698178	INS	chr3_110328180_110328689	3.72049322	10.1066991	7.8411E-06	0.0128791	H3K27ac	BF
chr3	68144528	68144998	DEL	chr3_68395099_68395599	2.32234141	10.0984729	7.889E-06	0.01292355	H3K27ac	BF
chr2	145329737	145330157	DEL	chr2_144876414_144877170	8.68298944	10.0996078	7.8824E-06	0.01292355	H3K27ac	BF
chr1	9913068	9913215	DEL	chr1_10078702_10078868	5.86455469	10.0982902	7.8901E-06	0.01292355	H3K27ac	BF
chr3	68141059	68142597	INV	chr3_68395099_68395599	4.64468281	10.0984729	7.889E-06	0.01292355	H3K27ac	BF
chr1	205959403	205959404	INS	chr1_206145816_206146424	3.20191675	10.0942356	7.9138E-06	0.01295522	H3K27ac	BF
chr5	71142449	71142450	INS	chr5_71179328_71180989	5.07047188	10.0829421	7.9803E-06	0.01305684	H3K27ac	BF
chr5	77783286	77783287	INS	chr5_77397915_77399567	2.71128945	10.0775904	8.012E-06	0.01310149	H3K27ac	BF
chr5	73979409	73979468	DEL	chr5_74154864_74155044	3.24957383	10.0726063	8.0417E-06	0.01314274	H3K27ac	BF
chr1	254733161	254733240	DEL	chr1_254624374_254624655	3.2274475	10.0697381	8.0588E-06	0.01315618	H3K27ac	BF
chr1	254888379	254888380	INS	chr1_254624374_254624655	3.2274475	10.0697381	8.0588E-06	0.01315618	H3K27ac	BF
chr1	8356436	8356437	INS	chr1_8424194_8424833	4.67031094	10.0629315	8.0996E-06	0.01321552	H3K27ac	BF

chr4	19914280	19914281	INS	chr4_20409167_20410006	3.7144945	10.0449341	8.2087E-06	0.01337867	H3K27ac	BF
chr4	20751112	20751113	INS	chr4_20409167_20410006	3.7144945	10.0449341	8.2087E-06	0.01337867	H3K27ac	BF
chr3	122636402	122636403	INS	chr3_122175920_122176117	2.87820406	10.0402641	8.2372E-06	0.01340303	H3K27ac	BF
chr1	129061546	129061547	INS	chr1_128881259_128881822	-11.019805	-10.040775	8.2341E-06	0.01340303	H3K27ac	BF
chr5	7581505	7581559	DEL	chr5_7347414_7347780	3.1108615	10.0360064	8.2634E-06	0.01343077	H3K27ac	BF
chr5	7579345	7579621	DEL	chr5_7347414_7347780	3.1108615	10.0360064	8.2634E-06	0.01343077	H3K27ac	BF
chr3	72474120	72474121	INS	chr3_72379056_72381806	-7.5891139	-10.034002	8.2757E-06	0.01343603	H3K27ac	BF
chr3	72467711	72467712	INS	chr3_72379056_72381806	-7.5891139	-10.034002	8.2757E-06	0.01343603	H3K27ac	BF
chr4	122979779	122979780	INS	chr4_122688126_122688562	3.43086833	10.0272935	8.3172E-06	0.01349135	H3K27ac	BF
chr1	58158194	58158195	INS	chr1_58191701_58192022	6.25399028	10.0270079	8.3189E-06	0.01349135	H3K27ac	BF
chr3	25804206	25804207	INS	chr3_26207481_26208042	4.92170764	10.0242869	8.3358E-06	0.01351131	H3K27ac	BF
NW_018085018.1	59529	59640	DEL	NW_018085018.1_416155_417711	4.83445389	10.0222697	8.3484E-06	0.01352421	H3K27ac	BF
chr5	62540128	62540433	DEL	chr5_62704590_62705549	2.61302375	10.0185615	8.3715E-06	0.0135542	H3K27ac	BF

chr2	13583454	13583455	INS	chr2_13150797_13151171	1.91703578	10.0161322	8.3866E-06	0.01357133	H3K27ac	BF
chr2	48297275	48297336	DEL	chr2_47894327_47895021	3.54631805	10.0091495	8.4304E-06	0.01363472	H3K27ac	BF
chr5	31001378	31001673	DEL	chr5_30651835_30652368	1.16088144	10.0076899	8.4396E-06	0.0136421	H3K27ac	BF
chr4	61434992	61437571	DEL	chr4_61123568_61124236	2.17638458	9.99445663	8.5234E-06	0.01371	H3K27ac	BF
chr4	61466393	61466462	DEL	chr4_61123568_61124236	2.17638458	9.99445663	8.5234E-06	0.01371	H3K27ac	BF
chr4	60813329	60813330	INS	chr4_61123568_61124236	2.17638458	9.99445663	8.5234E-06	0.01371	H3K27ac	BF
chr4	60869108	60869109	INS	chr4_61123568_61124236	2.17638458	9.99445663	8.5234E-06	0.01371	H3K27ac	BF
chr4	61369809	61369810	INS	chr4_61123568_61124236	2.17638458	9.99445663	8.5234E-06	0.01371	H3K27ac	BF
chr4	61433125	61433126	INS	chr4_61123568_61124236	2.17638458	9.99445663	8.5234E-06	0.01371	H3K27ac	BF
chr4	61454319	61454320	INS	chr4_61123568_61124236	2.17638458	9.99445663	8.5234E-06	0.01371	H3K27ac	BF
chr4	61614516	61614517	INS	chr4_61123568_61124236	2.17638458	9.99445663	8.5234E-06	0.01371	H3K27ac	BF
chr5	7581505	7581559	DEL	chr5_7847462_7847795	5.99571852	9.99147415	8.5424E-06	0.01371378	H3K27ac	BF
chr5	7579345	7579621	DEL	chr5_7847462_7847795	5.99571852	9.99147415	8.5424E-06	0.01371378	H3K27ac	BF

chr1	184341764	184341765	INS	chr1_184634610_184635134	3.68962167	9.99044734	8.549E-06	0.01371378	H3K27ac	BF
chr1	184417738	184417739	INS	chr1_184634610_184635134	3.68962167	9.99044734	8.549E-06	0.01371378	H3K27ac	BF
chr1	184444660	184444661	INS	chr1_184634610_184635134	3.68962167	9.99044734	8.549E-06	0.01371378	H3K27ac	BF
NW_018085018.1	59529	59640	DEL	NW_018085018.1_428348_430308	3.96619722	9.97911904	8.6217E-06	0.01381541	H3K27ac	BF
chr1	74342560	74342561	INS	chr1_74807759_74808187	2.71322691	9.97258847	8.6639E-06	0.01387419	H3K27ac	BF
chr5	77783286	77783287	INS	chr5_77440476_77441236	2.97768234	9.9719943	8.6677E-06	0.01387419	H3K27ac	BF
chr1	105707049	105707330	DEL	chr1_106046764_106048791	13.2512326	9.95668829	8.7677E-06	0.01401136	H3K27ac	BF
chr1	105639354	105639355	INS	chr1_106046764_106048791	13.2512326	9.95668829	8.7677E-06	0.01401136	H3K27ac	BF
chr1	106461104	106461105	INS	chr1_106046764_106048791	13.2512326	9.95668829	8.7677E-06	0.01401136	H3K27ac	BF
chr5	33889049	33889050	INS	chr5_34297306_34297824	4.29857451	9.95177693	8.8E-06	0.01402509	H3K27ac	BF
chr5	33887412	33887413	INS	chr5_34297306_34297824	4.29857451	9.95177693	8.8E-06	0.01402509	H3K27ac	BF
chr5	34010981	34010982	INS	chr5_34297306_34297824	4.29857451	9.95177693	8.8E-06	0.01402509	H3K27ac	BF
chr5	34024212	34024213	INS	chr5_34297306_34297824	4.29857451	9.95177693	8.8E-06	0.01402509	H3K27ac	BF
chr5	34178708	34178709	INS	chr5_34297306_34297824	4.29857451	9.95177693	8.8E-06	0.01402509	H3K27ac	BF
chr3	119996455	119996730	DEL	chr3_120003611_120004041	0.75513791	9.94792619	8.8255E-06	0.01405806	H3K27ac	BF

chr4	62680082	62680083	INS	chr4_62297986_62298440	1.91158194	9.94153229	8.8679E-06	0.0141141	H3K27ac	BF
chr1	229398592	229398593	INS	chr1_229531968_229533648	1.84914528	9.93975092	8.8798E-06	0.0141141	H3K27ac	BF
chr1	229396062	229396063	INS	chr1_229531968_229533648	1.84914528	9.93975092	8.8798E-06	0.0141141	H3K27ac	BF
chr1	239709046	239709186	DEL	chr1_239400296_239400865	1.84056822	9.93112639	8.9374E-06	0.01419052	H3K27ac	BF
chr1	239722010	239722358	DEL	chr1_239400296_239400865	1.84056822	9.93112639	8.9374E-06	0.01419052	H3K27ac	BF
chr3	22347726	22347727	INS	chr3_22731886_22732361	3.45691493	9.92991661	8.9456E-06	0.01419578	H3K27ac	BF
chr3	121657515	121657516	INS	chr3_121820237_121820401	3.32914516	9.91775366	9.0277E-06	0.01431077	H3K27ac	BF
chr1	18325395	18325396	INS	chr1_18504157_18504482	7.71528984	9.91652615	9.0361E-06	0.01431601	H3K27ac	BF
chr1	26247780	26247781	INS	chr1_26469359_26469700	3.280902	9.91583972	9.0407E-06	0.01431601	H3K27ac	BF
chr1	7746518	7747818	DEL	chr1_7643683_7644099	5.23627444	9.90334507	9.1261E-06	0.01443576	H3K27ac	BF
chr1	7491242	7491243	INS	chr1_7643683_7644099	5.23627444	9.90334507	9.1261E-06	0.01443576	H3K27ac	BF
chr5	7581505	7581559	DEL	chr5_7445096_7445431	1.5582325	9.89589582	9.1775E-06	0.01446276	H3K27ac	BF
chr5	7579345	7579621	DEL	chr5_7445096_7445431	1.5582325	9.89589582	9.1775E-06	0.01446276	H3K27ac	BF

chr4	107269662	107269663	INS	chr4_107374331_107374687	2.51096453	9.89840341	9.1601E-06	0.01446276	H3K27ac	BF
chr1	11626419	11626420	INS	chr1_12115021_12115220	5.05317727	9.89692836	9.1703E-06	0.01446276	H3K27ac	BF
chr3	72982792	72982851	DEL	chr3_73018267_73018628	2.48944958	9.87976369	9.2898E-06	0.01460855	H3K27ac	BF
chr3	73139546	73139547	INS	chr3_73018267_73018628	2.48944958	9.87976369	9.2898E-06	0.01460855	H3K27ac	BF
chr3	73402609	73402610	INS	chr3_73018267_73018628	2.48944958	9.87976369	9.2898E-06	0.01460855	H3K27ac	BF
chr1	188641399	188641400	INS	chr1_189110024_189111671	-1.6167263	-9.8802097	9.2866E-06	0.01460855	H3K27ac	BF
chr5	77568931	77569226	DEL	chr5_77754058_77755070	2.56055789	9.86361524	9.4037E-06	0.01477202	H3K27ac	BF
chr5	77568931	77569226	DEL	chr5_77757811_77758325	2.56055789	9.86361524	9.4037E-06	0.01477202	H3K27ac	BF
chr4	85322163	85322278	DEL	chr4_84981504_84981836	2.38959806	9.86177777	9.4168E-06	0.01477682	H3K27ac	BF
chr4	85224475	85224476	INS	chr4_84981504_84981836	2.38959806	9.86177777	9.4168E-06	0.01477682	H3K27ac	BF
chr1	26088493	26088670	DEL	chr1_26469359_26469700	3.25899289	9.85199887	9.4867E-06	0.01486277	H3K27ac	BF
chr1	26539658	26539718	DEL	chr1_26469359_26469700	3.25899289	9.85199887	9.4867E-06	0.01486277	H3K27ac	BF
chr2	132195622	132195703	DEL	chr2_132195140_132196207	6.18908983	9.8359203	9.6028E-06	0.01502083	H3K27ac	BF

chr2	132071320	132071321	INS	chr2_132195140_132196207	6.18908983	9.8359203	9.6028E-06	0.01502083	H3K27ac	BF
chr2	132420954	132420955	INS	chr2_132195140_132196207	6.18908983	9.8359203	9.6028E-06	0.01502083	H3K27ac	BF
chr1	20834452	20834453	INS	chr1_20899666_20900297	2.95984125	9.82034542	9.7168E-06	0.01519114	H3K27ac	BF
chr3	127204794	127207673	DEL	chr3_127251754_127252271	2.60740544	9.8168035	9.743E-06	0.0152159	H3K27ac	BF
chr3	126820716	126820717	INS	chr3_127251754_127252271	2.60740544	9.8168035	9.743E-06	0.0152159	H3K27ac	BF
chr1	223159591	223159885	DEL	chr1_223648913_223649118	4.05396031	9.8046629	9.8332E-06	0.01534865	H3K27ac	BF
chr3	114598599	114598668	DEL	chr3_115019261_115019606	4.29298783	9.79571499	9.9002E-06	0.01536694	H3K27ac	BF
chr1	34116497	34118117	DEL	chr1_33931082_33931344	1.42378431	9.79546495	9.9021E-06	0.01536694	H3K27ac	BF
chr2	65438528	65438529	INS	chr2_65636013_65637751	1.95211069	9.79754296	9.8865E-06	0.01536694	H3K27ac	BF
chr3	114749286	114749287	INS	chr3_115019261_115019606	4.29298783	9.79571499	9.9002E-06	0.01536694	H3K27ac	BF
chr3	114840061	114840062	INS	chr3_115019261_115019606	4.29298783	9.79571499	9.9002E-06	0.01536694	H3K27ac	BF
chr3	114970527	114970528	INS	chr3_115019261_115019606	4.29298783	9.79571499	9.9002E-06	0.01536694	H3K27ac	BF
chr1	33650404	33650405	INS	chr1_33931082_33931344	1.42378431	9.79546495	9.9021E-06	0.01536694	H3K27ac	BF

chr1	33639163	33639164	INS	chr1_33931082_33931344	1.42378431	9.79546495	9.9021E-06	0.01536694	H3K27ac	BF
chr1	58158194	58158195	INS	chr1_57713412_57714314	1.04452694	9.79230857	9.9259E-06	0.01539576	H3K27ac	BF
chr5	47270892	47270956	DEL	chr5_47248325_47249361	2.89431458	9.78470992	9.9834E-06	0.01546063	H3K27ac	BF
chr5	47503686	47503687	INS	chr5_47248325_47249361	2.89431458	9.78470992	9.9834E-06	0.01546063	H3K27ac	BF
chr1	43925947	43925948	INS	chr1_44335164_44335818	3.93881933	9.78564734	9.9763E-06	0.01546063	H3K27ac	BF
chr1	129061546	129061547	INS	chr1_128860682_128861045	-48.022884	-80.800994	6.1372E-13	1.0069E-07	H3K27ac	BF
chr5	66188135	66188136	INS	chr5_66430044_66430506	10.0345288	35.4204699	4.4182E-10	1.0356E-05	H3K27ac	BF
chr5	66644134	66644135	INS	chr5_66430044_66430506	10.0345288	35.4204699	4.4182E-10	1.0356E-05	H3K27ac	BF
chr1	20834452	20834453	INS	chr1_20914140_20914754	8.88080819	35.4452871	4.3937E-10	1.0356E-05	H3K27ac	BF
chr1	6460049	6460050	INS	chr1_6883688_6884563	18.5353549	51.4777295	2.2467E-11	1.0532E-06	H3K27ac	BF
chr1	6643056	6643057	INS	chr1_6883688_6884563	18.5353549	51.4777295	2.2467E-11	1.0532E-06	H3K27ac	BF
chr4	12348698	12348881	DEL	chr4_12059697_12060283	9.36178433	35.1363546	4.7105E-10	1.0539E-05	H3K27ac	BF
chr1	8356436	8356437	INS	chr1_8753122_8753442	3.14378231	35.1695274	4.6753E-10	1.0539E-05	H3K27ac	BF

chr4	11764208	11764209	INS	chr4_12059697_12060283	9.36178433	35.1363546	4.7105E-10	1.0539E-05	H3K27ac	BF
chr4	12168744	12168745	INS	chr4_12059697_12060283	9.36178433	35.1363546	4.7105E-10	1.0539E-05	H3K27ac	BF
chr4	12354380	12354381	INS	chr4_12059697_12060283	9.36178433	35.1363546	4.7105E-10	1.0539E-05	H3K27ac	BF
chr1	20834452	20834453	INS	chr1_20872835_20873103	15.2231675	35.1946032	4.6488E-10	1.0539E-05	H3K27ac	BF
chr4	125695336	125695337	INS	chr4_125495702_125496351	4.12223661	34.7509314	5.1424E-10	1.1249E-05	H3K27ac	BF
chr5	6640570	6640571	INS	chr5_6699673_6701380	12.4290078	34.7552437	5.1373E-10	1.1249E-05	H3K27ac	BF
chr5	6626341	6626342	INS	chr5_6699673_6701380	12.4290078	34.7552437	5.1373E-10	1.1249E-05	H3K27ac	BF
chr4	117978801	117978916	DEL	chr4_117978278_117978721	13.0484862	34.2868797	5.7226E-10	1.1575E-05	H3K27ac	BF
chr4	118001729	118001880	DEL	chr4_117978278_117978721	13.0484862	34.2868797	5.7226E-10	1.1575E-05	H3K27ac	BF
chr4	117999909	118000198	DEL	chr4_117978278_117978721	13.0484862	34.2868797	5.7226E-10	1.1575E-05	H3K27ac	BF
chr2	126596215	126596496	DEL	chr2_126678179_126679757	7.627225	34.3192649	5.6798E-10	1.1575E-05	H3K27ac	BF
chr4	117746392	117746393	INS	chr4_117978278_117978721	13.0484862	34.2868797	5.7226E-10	1.1575E-05	H3K27ac	BF
chr4	117945046	117945047	INS	chr4_117978278_117978721	13.0484862	34.2868797	5.7226E-10	1.1575E-05	H3K27ac	BF

chr4	118002624	118002625	INS	chr4_117978278_117978721	13.0484862	34.2868797	5.7226E-10	1.1575E-05	H3K27ac	BF
chr4	118005675	118005676	INS	chr4_117978278_117978721	13.0484862	34.2868797	5.7226E-10	1.1575E-05	H3K27ac	BF
chr4	118083331	118083332	INS	chr4_117978278_117978721	13.0484862	34.2868797	5.7226E-10	1.1575E-05	H3K27ac	BF
chr4	118131010	118131011	INS	chr4_117978278_117978721	13.0484862	34.2868797	5.7226E-10	1.1575E-05	H3K27ac	BF
chr5	3596904	3597581	DEL	chr5_4072087_4072450	16.2884296	50.3819371	2.6675E-11	1.2119E-06	H3K27ac	BF
chr5	3651763	3651814	DEL	chr5_4072087_4072450	16.2884296	50.3819371	2.6675E-11	1.2119E-06	H3K27ac	BF
chr5	62540128	62540433	DEL	chr5_62759644_62760284	4.46127538	50.0916825	2.7933E-11	1.2499E-06	H3K27ac	BF
chr2	139980016	139980293	DEL	chr2_140295060_140295588	7.97063372	33.5263424	6.8397E-10	1.3466E-05	H3K27ac	BF
chr2	139984127	139984399	DEL	chr2_140295060_140295588	7.97063372	33.5263424	6.8397E-10	1.3466E-05	H3K27ac	BF
chr4	62680082	62680083	INS	chr4_62756264_62756679	17.6031126	33.5984048	6.724E-10	1.3466E-05	H3K27ac	BF
chr2	139983889	139983890	INS	chr2_140295060_140295588	7.97063372	33.5263424	6.8397E-10	1.3466E-05	H3K27ac	BF
chr5	70607550	70607551	INS	chr5_70239300_70239820	9.61371244	33.3891106	7.0664E-10	1.3678E-05	H3K27ac	BF
chr1	6265442	6265443	INS	chr1_6756206_6756955	6.09717594	33.3227492	7.1791E-10	1.3678E-05	H3K27ac	BF

chr4	116896255	116896256	INS	chr4_117134200_117135570	12.5357156	33.3337019	7.1603E-10	1.3678E-05	H3K27ac	BF
chr4	117473079	117473080	INS	chr4_117134200_117135570	12.5357156	33.3337019	7.1603E-10	1.3678E-05	H3K27ac	BF
chr1	6669966	6669967	INS	chr1_6756206_6756955	6.09717594	33.3227492	7.1791E-10	1.3678E-05	H3K27ac	BF
chr1	201038608	201038676	DEL	chr1_201258251_201258900	13.6413761	33.1484508	7.4846E-10	1.3884E-05	H3K27ac	BF
chr5	7581505	7581559	DEL	chr5_7856888_7857242	7.92348344	33.1847041	7.4199E-10	1.3884E-05	H3K27ac	BF
chr5	7579345	7579621	DEL	chr5_7856888_7857242	7.92348344	33.1847041	7.4199E-10	1.3884E-05	H3K27ac	BF
chr4	116550806	116550807	INS	chr4_116829719_116830288	3.64139988	33.178847	7.4303E-10	1.3884E-05	H3K27ac	BF
chr1	9401021	9401022	INS	chr1_9529983_9531711	13.4505059	33.1275255	7.5223E-10	1.3884E-05	H3K27ac	BF
chr5	5937136	5937631	DEL	chr5_6413512_6414010	7.6806125	33.078279	7.6117E-10	1.3962E-05	H3K27ac	BF
chr2	143620924	143620983	DEL	chr2_143727114_143727766	7.89642333	32.7545593	8.2305E-10	1.4912E-05	H3K27ac	BF
chr2	143620660	143620661	INS	chr2_143727114_143727766	7.89642333	32.7545593	8.2305E-10	1.4912E-05	H3K27ac	BF
chr1	205959403	205959404	INS	chr1_206002160_206002841	8.46597613	48.6917989	3.5019E-11	1.5436E-06	H3K27ac	BF
chr1	13769208	13769209	INS	chr1_13935436_13936414	10.0363268	32.349369	9.0862E-10	1.6165E-05	H3K27ac	BF

chr1	13781480	13781481	INS	chr1_13935436_13936414	10.0363268	32.349369	9.0862E-10	1.6165E-05	H3K27ac	BF
chr1	104052039	104052552	DEL	chr1_103787062_103787306	12.2572894	32.2909172	9.2177E-10	1.6301E-05	H3K27ac	BF
chr5	73979409	73979468	DEL	chr5_73568690_73569812	10.8661839	32.1910981	9.4473E-10	1.6607E-05	H3K27ac	BF
chr5	11265336	11265337	INS	chr5_11578819_11579534	14.3930439	32.0817207	9.7062E-10	1.6961E-05	H3K27ac	BF
chr3	68144528	68144998	DEL	chr3_68314170_68314744	9.27632419	31.8129545	1.0377E-09	1.7131E-05	H3K27ac	BF
chr4	117978801	117978916	DEL	chr4_118451239_118451607	11.3550078	31.7220952	1.0616E-09	1.7131E-05	H3K27ac	BF
chr4	118001729	118001880	DEL	chr4_118451239_118451607	11.3550078	31.7220952	1.0616E-09	1.7131E-05	H3K27ac	BF
chr4	117999909	118000198	DEL	chr4_118451239_118451607	11.3550078	31.7220952	1.0616E-09	1.7131E-05	H3K27ac	BF
chr4	118834697	118835149	DEL	chr4_118451239_118451607	11.3550078	31.7220952	1.0616E-09	1.7131E-05	H3K27ac	BF
chr1	6460049	6460050	INS	chr1_6927802_6928270	6.60846819	31.9914051	9.9261E-10	1.7131E-05	H3K27ac	BF
chr4	118002624	118002625	INS	chr4_118451239_118451607	11.3550078	31.7220952	1.0616E-09	1.7131E-05	H3K27ac	BF
chr4	118005675	118005676	INS	chr4_118451239_118451607	11.3550078	31.7220952	1.0616E-09	1.7131E-05	H3K27ac	BF
chr4	118083331	118083332	INS	chr4_118451239_118451607	11.3550078	31.7220952	1.0616E-09	1.7131E-05	H3K27ac	BF

chr4	118131010	118131011	INS	chr4_118451239_118451607	11.3550078	31.7220952	1.0616E-09	1.7131E-05	H3K27ac	BF
chr4	118860341	118860342	INS	chr4_118451239_118451607	11.3550078	31.7220952	1.0616E-09	1.7131E-05	H3K27ac	BF
chr1	6643056	6643057	INS	chr1_6927802_6928270	6.60846819	31.9914051	9.9261E-10	1.7131E-05	H3K27ac	BF
chr1	20834452	20834453	INS	chr1_20739300_20739805	6.64533638	31.7805867	1.0461E-09	1.7131E-05	H3K27ac	BF
chr3	68141059	68142597	INV	chr3_68314170_68314744	18.5526484	31.8129545	1.0377E-09	1.7131E-05	H3K27ac	BF
chr2	126596215	126596496	DEL	chr2_126749917_126750722	4.4564115	31.6215963	1.0886E-09	1.7473E-05	H3K27ac	BF
chr1	11626419	11626420	INS	chr1_12062396_12062729	8.42088313	31.3276321	1.1725E-09	1.8517E-05	H3K27ac	BF
chr1	6669966	6669967	INS	chr1_6955893_6956296	9.54505328	46.978721	4.6596E-11	2.0237E-06	H3K27ac	BF
chr1	6643056	6643057	INS	chr1_7092501_7092786	13.8747626	46.7921598	4.8098E-11	2.0586E-06	H3K27ac	BF
chr2	140666739	140666978	DEL	chr2_140282508_140283227	8.57233906	30.629209	1.4023E-09	2.1912E-05	H3K27ac	BF
chr2	140729157	140729158	INS	chr2_140282508_140283227	8.57233906	30.629209	1.4023E-09	2.1912E-05	H3K27ac	BF
chr2	126596215	126596496	DEL	chr2_127071622_127071927	12.1280928	30.4722817	1.4607E-09	2.2704E-05	H3K27ac	BF
chr1	129061546	129061547	INS	chr1_129135639_129136495	-9.2238855	-30.400089	1.4885E-09	2.3015E-05	H3K27ac	BF

chr5	73979409	73979468	DEL	chr5_74464366_74465300	7.60877181	30.3167481	1.5213E-09	2.3158E-05	H3K27ac	BF
chr1	8356436	8356437	INS	chr1_8589555_8589824	13.5275326	30.3197833	1.5201E-09	2.3158E-05	H3K27ac	BF
chr1	205959403	205959404	INS	chr1_206107971_206108558	10.4065426	30.3362418	1.5135E-09	2.3158E-05	H3K27ac	BF
chr3	13581410	13581411	INS	chr3_13332522_13332856	7.03480206	30.2344177	1.5545E-09	2.3542E-05	H3K27ac	BF
chr3	68144528	68144998	DEL	chr3_68289551_68290903	12.2057338	29.9450912	1.6777E-09	2.5151E-05	H3K27ac	BF
chr3	68141059	68142597	INV	chr3_68289551_68290903	24.4114675	29.9450912	1.6777E-09	2.5151E-05	H3K27ac	BF
chr5	7581505	7581559	DEL	chr5_7602528_7602798	7.96364044	29.8476739	1.7217E-09	2.5551E-05	H3K27ac	BF
chr5	7579345	7579621	DEL	chr5_7602528_7602798	7.96364044	29.8476739	1.7217E-09	2.5551E-05	H3K27ac	BF
chr4	62680082	62680083	INS	chr4_62526100_62526833	16.4399156	71.3247561	1.6628E-12	2.567E-07	H3K27ac	BF
chr1	6643056	6643057	INS	chr1_6971979_6972623	12.16314	70.9287557	1.7384E-12	2.567E-07	H3K27ac	BF
chr5	40186785	40186834	DEL	chr5_40182658_40182876	20.5522286	44.3192532	7.4152E-11	2.7763E-06	H3K27ac	BF
chr5	40194281	40194330	DEL	chr5_40182658_40182876	20.5522286	44.3192532	7.4152E-11	2.7763E-06	H3K27ac	BF
chr5	40181920	40181921	INS	chr5_40182658_40182876	20.5522286	44.3192532	7.4152E-11	2.7763E-06	H3K27ac	BF

chr4	62680082	62680083	INS	chr4_62830536_62831880	12.7031438	44.4861761	7.1963E-11	2.7763E-06	H3K27ac	BF
chr3	70787884	70787885	INS	chr3_70834609_70835357	9.49145933	44.7823588	6.8256E-11	2.7763E-06	H3K27ac	BF
chr3	70846907	70846908	INS	chr3_70834609_70835357	9.49145933	44.7823588	6.8256E-11	2.7763E-06	H3K27ac	BF
chr5	77356348	77356349	INS	chr5_77685467_77685704	19.0978942	44.310581	7.4268E-11	2.7763E-06	H3K27ac	BF
chr5	77545855	77545856	INS	chr5_77685467_77685704	19.0978942	44.310581	7.4268E-11	2.7763E-06	H3K27ac	BF
chr5	78067627	78067628	INS	chr5_77685467_77685704	19.0978942	44.310581	7.4268E-11	2.7763E-06	H3K27ac	BF
chr1	20834452	20834453	INS	chr1_20881392_20882256	7.67536375	29.504739	1.887E-09	2.7864E-05	H3K27ac	BF
chr1	86136311	86136312	INS	chr1_86394196_86395303	11.3555534	29.2819238	2.004E-09	2.8591E-05	H3K27ac	BF
chr1	86279573	86279574	INS	chr1_86394196_86395303	11.3555534	29.2819238	2.004E-09	2.8591E-05	H3K27ac	BF
chr1	86508701	86508702	INS	chr1_86394196_86395303	11.3555534	29.2819238	2.004E-09	2.8591E-05	H3K27ac	BF
chr1	86606023	86606024	INS	chr1_86394196_86395303	11.3555534	29.2819238	2.004E-09	2.8591E-05	H3K27ac	BF
chr1	86702062	86702063	INS	chr1_86394196_86395303	11.3555534	29.2819238	2.004E-09	2.8591E-05	H3K27ac	BF
chr1	86756615	86756616	INS	chr1_86394196_86395303	11.3555534	29.2819238	2.004E-09	2.8591E-05	H3K27ac	BF

chr1	205959403	205959404	INS	chr1_206040195_206042007	3.25910563	29.3122093	1.9876E-09	2.8591E-05	H3K27ac	BF
chr3	54799284	54799285	INS	chr3_54508349_54509098	18.6010536	69.0809271	2.1465E-12	2.8814E-07	H3K27ac	BF
chr3	54915210	54915211	INS	chr3_54508349_54509098	18.6010536	69.0809271	2.1465E-12	2.8814E-07	H3K27ac	BF
chr3	126980653	126980722	DEL	chr3_127384249_127384885	12.4102272	29.1553007	2.0741E-09	2.903E-05	H3K27ac	BF
chr3	127215039	127215413	DEL	chr3_127384249_127384885	12.4102272	29.1553007	2.0741E-09	2.903E-05	H3K27ac	BF
chr3	127165473	127165474	INS	chr3_127384249_127384885	12.4102272	29.1553007	2.0741E-09	2.903E-05	H3K27ac	BF
chr3	127701685	127701686	INS	chr3_127384249_127384885	12.4102272	29.1553007	2.0741E-09	2.903E-05	H3K27ac	BF
chr5	7581505	7581559	DEL	chr5_7761346_7762067	9.65287769	29.1032125	2.1037E-09	2.9168E-05	H3K27ac	BF
chr5	7579345	7579621	DEL	chr5_7761346_7762067	9.65287769	29.1032125	2.1037E-09	2.9168E-05	H3K27ac	BF
chr1	104052039	104052552	DEL	chr1_104445118_104445373	6.73953694	28.810029	2.2797E-09	3.146E-05	H3K27ac	BF
chr1	252516114	252516183	DEL	chr1_252608972_252609726	16.2803541	67.100595	2.7079E-12	3.1542E-07	H3K27ac	BF
chr1	6460049	6460050	INS	chr1_6863431_6863696	15.8766003	66.5740149	2.8838E-12	3.1542E-07	H3K27ac	BF
chr1	6643056	6643057	INS	chr1_6863431_6863696	15.8766003	66.5740149	2.8838E-12	3.1542E-07	H3K27ac	BF

chr1	252456578	252456579	INS	chr1_252608972_252609726	16.2803541	67.100595	2.7079E-12	3.1542E-07	H3K27ac	BF
chr1	252514025	252514026	INS	chr1_252608972_252609726	16.2803541	67.100595	2.7079E-12	3.1542E-07	H3K27ac	BF
chr1	104052039	104052552	DEL	chr1_104352418_104352940	4.15855656	28.7638438	2.3089E-09	3.1714E-05	H3K27ac	BF
chr1	205959403	205959404	INS	chr1_206049786_206050072	16.0079288	28.6627731	2.3742E-09	3.2312E-05	H3K27ac	BF
chr1	129061546	129061547	INS	chr1_128912094_128913275	-16.419818	-65.792576	3.169E-12	3.3424E-07	H3K27ac	BF
chr5	73979409	73979468	DEL	chr5_73578669_73579799	6.75815669	42.6458833	1.0077E-10	3.3562E-06	H3K27ac	BF
chr2	133701012	133701064	DEL	chr2_134061630_134062519	9.37587622	42.5663707	1.0228E-10	3.3562E-06	H3K27ac	BF
chr2	133899189	133902056	DEL	chr2_134061630_134062519	9.37587622	42.5663707	1.0228E-10	3.3562E-06	H3K27ac	BF
chr2	133904101	133904172	DEL	chr2_134061630_134062519	9.37587622	42.5663707	1.0228E-10	3.3562E-06	H3K27ac	BF
chr1	129061546	129061547	INS	chr1_128869895_128871310	-22.428973	-42.851027	9.6991E-11	3.3562E-06	H3K27ac	BF
chr2	133913002	133913003	INS	chr2_134061630_134062519	9.37587622	42.5663707	1.0228E-10	3.3562E-06	H3K27ac	BF
chr2	133907109	133907110	INS	chr2_134061630_134062519	9.37587622	42.5663707	1.0228E-10	3.3562E-06	H3K27ac	BF
chr2	133904244	133904245	INS	chr2_134061630_134062519	9.37587622	42.5663707	1.0228E-10	3.3562E-06	H3K27ac	BF

chr2	134416866	134416867	INS	chr2_134061630_134062519	9.37587622	42.5663707	1.0228E-10	3.3562E-06	H3K27ac	BF
chr2	134503933	134503934	INS	chr2_134061630_134062519	9.37587622	42.5663707	1.0228E-10	3.3562E-06	H3K27ac	BF
chr1	29844104	29844105	INS	chr1_29858342_29859448	11.6825509	42.7717199	9.8433E-11	3.3562E-06	H3K27ac	BF
chr1	6460049	6460050	INS	chr1_6850053_6851505	9.63406706	28.4094888	2.5473E-09	3.4195E-05	H3K27ac	BF
chr1	6643056	6643057	INS	chr1_6850053_6851505	9.63406706	28.4094888	2.5473E-09	3.4195E-05	H3K27ac	BF
chr4	125695336	125695337	INS	chr4_125343169_125344130	11.9941956	28.2976184	2.6283E-09	3.4964E-05	H3K27ac	BF
chr1	129061546	129061547	INS	chr1_128699359_128700003	-28.46475	-28.303862	2.6237E-09	3.4964E-05	H3K27ac	BF
chr5	15414449	15414695	DEL	chr5_14973818_14975111	5.25572667	28.2329657	2.6764E-09	3.5129E-05	H3K27ac	BF
chr5	15113608	15113609	INS	chr5_14973818_14975111	5.25572667	28.2329657	2.6764E-09	3.5129E-05	H3K27ac	BF
chr5	15367044	15367045	INS	chr5_14973818_14975111	5.25572667	28.2329657	2.6764E-09	3.5129E-05	H3K27ac	BF
chr5	77783286	77783287	INS	chr5_77403616_77404203	9.88001719	64.6687217	3.6364E-12	3.7032E-07	H3K27ac	BF
chr4	116550806	116550807	INS	chr4_116919329_116919846	6.36764944	27.9904152	2.8659E-09	3.745E-05	H3K27ac	BF
chr5	73979409	73979468	DEL	chr5_73610216_73610927	6.039685	27.9710972	2.8816E-09	3.7489E-05	H3K27ac	BF

chr1	97160666	97161813	DEL	chr1_97384660_97384887	7.05914456	41.6871585	1.2079E-10	3.8773E-06	H3K27ac	BF
chr1	97151189	97151190	INS	chr1_97384660_97384887	7.05914456	41.6871585	1.2079E-10	3.8773E-06	H3K27ac	BF
chr2	126596215	126596496	DEL	chr2_126953231_126954297	3.16694069	27.8007836	3.0245E-09	3.9176E-05	H3K27ac	BF
chr4	116550806	116550807	INS	chr4_116453064_116454392	17.0747734	41.4224431	1.2707E-10	4.0353E-06	H3K27ac	BF
chr1	221596935	221596936	INS	chr1_221800995_221801551	17.5815047	27.6400877	3.1668E-09	4.0839E-05	H3K27ac	BF
chr1	6460049	6460050	INS	chr1_6761599_6761989	5.69392919	41.152584	1.3387E-10	4.1615E-06	H3K27ac	BF
chr1	6643056	6643057	INS	chr1_6761599_6761989	5.69392919	41.152584	1.3387E-10	4.1615E-06	H3K27ac	BF
chr4	123025234	123025235	INS	chr4_123449900_123450400	28.3170776	93.0645512	1.9838E-13	4.1848E-08	H3K27ac	BF
chr4	123447592	123447593	INS	chr4_123449900_123450400	28.3170776	93.0645512	1.9838E-13	4.1848E-08	H3K27ac	BF
chr1	11626419	11626420	INS	chr1_12082013_12082229	5.79356925	27.4126937	3.381E-09	4.3038E-05	H3K27ac	BF
chr1	129061546	129061547	INS	chr1_128853448_128854639	-8.2893001	-40.824076	1.4269E-10	4.3896E-06	H3K27ac	BF
chr4	104290539	104290540	INS	chr4_104613415_104613849	14.5257722	27.1932269	3.6034E-09	4.5283E-05	H3K27ac	BF
chr4	104532089	104532090	INS	chr4_104613415_104613849	14.5257722	27.1932269	3.6034E-09	4.5283E-05	H3K27ac	BF

chr5	73979409	73979468	DEL	chr5_73585200_73585755	11.8833739	27.1666562	3.6314E-09	4.5442E-05	H3K27ac	BF
chr1	11626419	11626420	INS	chr1_12070177_12070760	4.16372006	27.0841232	3.72E-09	4.5966E-05	H3K27ac	BF
chr5	14116104	14116105	INS	chr5_14503174_14503608	21.2452908	61.9459883	5.1269E-12	4.7316E-07	H3K27ac	BF
chr5	14186300	14186301	INS	chr5_14503174_14503608	21.2452908	61.9459883	5.1269E-12	4.7316E-07	H3K27ac	BF
chr5	14416409	14416410	INS	chr5_14503174_14503608	21.2452908	61.9459883	5.1269E-12	4.7316E-07	H3K27ac	BF
chr3	68144528	68144998	DEL	chr3_68459621_68460824	15.8251256	59.9079457	6.6967E-12	4.7602E-07	H3K27ac	BF
chr5	41398825	41398826	INS	chr5_41746664_41747777	21.5302023	59.1487493	7.4145E-12	4.7602E-07	H3K27ac	BF
chr5	41406216	41406217	INS	chr5_41746664_41747777	21.5302023	59.1487493	7.4145E-12	4.7602E-07	H3K27ac	BF
chr5	41446260	41446261	INS	chr5_41746664_41747777	21.5302023	59.1487493	7.4145E-12	4.7602E-07	H3K27ac	BF
chr5	41571403	41571404	INS	chr5_41746664_41747777	21.5302023	59.1487493	7.4145E-12	4.7602E-07	H3K27ac	BF
chr5	41851424	41851425	INS	chr5_41746664_41747777	21.5302023	59.1487493	7.4145E-12	4.7602E-07	H3K27ac	BF
chr5	42116965	42116966	INS	chr5_41746664_41747777	21.5302023	59.1487493	7.4145E-12	4.7602E-07	H3K27ac	BF
chr5	68212673	68212674	INS	chr5_68252044_68252682	18.7572742	60.9468117	5.8378E-12	4.7602E-07	H3K27ac	BF

chr5	68692912	68692913	INS	chr5_68252044_68252682	18.7572742	60.9468117	5.8378E-12	4.7602E-07	H3K27ac	BF
chr1	129061546	129061547	INS	chr1_129155306_129155865	-16.904718	-60.955647	5.831E-12	4.7602E-07	H3K27ac	BF
chr1	11626419	11626420	INS	chr1_11962503_11963220	4.622515	59.1921921	7.3712E-12	4.7602E-07	H3K27ac	BF
chr3	68141059	68142597	INV	chr3_68459621_68460824	31.6502513	59.9079457	6.6967E-12	4.7602E-07	H3K27ac	BF
chr3	90070561	90070562	INS	chr3_89875868_89877020	22.1481361	58.4166334	8.1896E-12	4.9359E-07	H3K27ac	BF
chr3	90133194	90133195	INS	chr3_89875868_89877020	22.1481361	58.4166334	8.1896E-12	4.9359E-07	H3K27ac	BF
chr1	205959403	205959404	INS	chr1_206061309_206061492	10.8925569	40.1647776	1.6245E-10	4.9459E-06	H3K27ac	BF
chr3	53202776	53202837	DEL	chr3_53275069_53276205	9.50440083	39.7485855	1.765E-10	4.9643E-06	H3K27ac	BF
chr3	68144528	68144998	DEL	chr3_67721565_67722543	9.17538675	39.9883431	1.6825E-10	4.9643E-06	H3K27ac	BF
chr5	7581505	7581559	DEL	chr5_7247498_7248632	9.972715	39.9200796	1.7055E-10	4.9643E-06	H3K27ac	BF
chr5	7579345	7579621	DEL	chr5_7247498_7248632	9.972715	39.9200796	1.7055E-10	4.9643E-06	H3K27ac	BF
chr3	53192935	53192936	INS	chr3_53275069_53276205	9.50440083	39.7485855	1.765E-10	4.9643E-06	H3K27ac	BF
chr3	53196048	53196049	INS	chr3_53275069_53276205	9.50440083	39.7485855	1.765E-10	4.9643E-06	H3K27ac	BF

chr3	53605055	53605056	INS	chr3_53275069_53276205	9.50440083	39.7485855	1.765E-10	4.9643E-06	H3K27ac	BF
chr3	68141059	68142597	INV	chr3_67721565_67722543	18.3507735	39.9883431	1.6825E-10	4.9643E-06	H3K27ac	BF
chr2	13122273	13123683	DEL	chr2_13456126_13456389	5.99149771	26.7401936	4.1163E-09	5.0651E-05	H3K27ac	BF
chr5	31001378	31001673	DEL	chr5_30602857_30603142	7.1516795	26.7000781	4.1655E-09	5.1045E-05	H3K27ac	BF
chr5	15414449	15414695	DEL	chr5_15434049_15434230	21.0888184	57.436376	9.3742E-12	5.3239E-07	H3K27ac	BF
chr5	15113608	15113609	INS	chr5_15434049_15434230	21.0888184	57.436376	9.3742E-12	5.3239E-07	H3K27ac	BF
chr5	15367044	15367045	INS	chr5_15434049_15434230	21.0888184	57.436376	9.3742E-12	5.3239E-07	H3K27ac	BF
chr2	101635023	101635024	INS	chr2_102024177_102024605	9.38732878	26.4072195	4.5457E-09	5.5245E-05	H3K27ac	BF
chr2	102343986	102343987	INS	chr2_102024177_102024605	9.38732878	26.4072195	4.5457E-09	5.5245E-05	H3K27ac	BF
chr1	205959403	205959404	INS	chr1_206119822_206121335	12.6534606	39.1510612	1.9913E-10	5.5478E-06	H3K27ac	BF
chr1	8356436	8356437	INS	chr1_8761157_8762041	2.33085631	26.3737379	4.5916E-09	5.5574E-05	H3K27ac	BF
chr3	72292473	72293698	DEL	chr3_72634356_72634811	14.5530311	56.9110999	1.0088E-11	5.5583E-07	H3K27ac	BF
chr5	66188135	66188136	INS	chr5_66251599_66251843	24.4708275	56.7271789	1.0352E-11	5.5583E-07	H3K27ac	BF

chr5	66644134	66644135	INS	chr5_66251599_66251843	24.4708275	56.7271789	1.0352E-11	5.5583E-07	H3K27ac	BF
chr1	79767443	79767752	DEL	chr1_79545438_79545724	15.6995591	38.9292433	2.0834E-10	5.6448E-06	H3K27ac	BF
chr1	79156622	79156623	INS	chr1_79545438_79545724	15.6995591	38.9292433	2.0834E-10	5.6448E-06	H3K27ac	BF
chr5	62540128	62540433	DEL	chr5_62409453_62411235	6.924585	26.2957081	4.7006E-09	5.6661E-05	H3K27ac	BF
chr1	119880398	119880458	DEL	chr1_119785418_119785711	10.7667627	26.2188446	4.8108E-09	5.7288E-05	H3K27ac	BF
chr1	120002818	120002895	DEL	chr1_119785418_119785711	10.7667627	26.2188446	4.8108E-09	5.7288E-05	H3K27ac	BF
chr1	119938480	119938481	INS	chr1_119785418_119785711	10.7667627	26.2188446	4.8108E-09	5.7288E-05	H3K27ac	BF
chr4	68096511	68096512	INS	chr4_68525788_68527452	8.44109072	26.0318576	5.0913E-09	6.0143E-05	H3K27ac	BF
chr5	31001378	31001673	DEL	chr5_30539295_30540325	4.79166188	25.9878695	5.1599E-09	6.0711E-05	H3K27ac	BF
chr3	113573874	113573875	INS	chr3_113490520_113492966	9.87736278	25.9429846	5.231E-09	6.1303E-05	H3K27ac	BF
chr4	62680082	62680083	INS	chr4_62610607_62612062	10.3392294	25.8393335	5.3994E-09	6.3026E-05	H3K27ac	BF
chr5	62540128	62540433	DEL	chr5_62202797_62205150	7.11681875	25.8199813	5.4315E-09	6.3151E-05	H3K27ac	BF
chr3	4171395	4171752	DEL	chr3_3875349_3876161	10.152295	38.1736802	2.4353E-10	6.4213E-06	H3K27ac	BF

chr3	4188412	4188413	INS	chr3_3875349_3876161	10.152295	38.1736802	2.4353E-10	6.4213E-06	H3K27ac	BF
chr4	116550806	116550807	INS	chr4_116934258_116935033	15.9154158	55.1199317	1.3021E-11	6.8667E-07	H3K27ac	BF
chr1	11626419	11626420	INS	chr1_11996478_11997642	4.11258938	25.5049267	5.9857E-09	6.8782E-05	H3K27ac	BF
chr1	6460049	6460050	INS	chr1_6760974_6761213	7.26861244	25.202832	6.5774E-09	7.4998E-05	H3K27ac	BF
chr1	6643056	6643057	INS	chr1_6760974_6761213	7.26861244	25.202832	6.5774E-09	7.4998E-05	H3K27ac	BF
chr1	8356436	8356437	INS	chr1_7950392_7950639	9.37268921	25.1545653	6.6779E-09	7.5561E-05	H3K27ac	BF
chr1	8356436	8356437	INS	chr1_8416988_8417689	6.63172125	25.1565166	6.6738E-09	7.5561E-05	H3K27ac	BF
chr1	20834452	20834453	INS	chr1_20581056_20581349	9.57490269	37.2176724	2.98E-10	7.7199E-06	H3K27ac	BF
chr1	141594721	141594722	INS	chr1_141665415_141666180	5.31919656	25.0431064	6.9166E-09	7.7252E-05	H3K27ac	BF
chr1	11626419	11626420	INS	chr1_12063848_12064306	9.514058	25.0133111	6.982E-09	7.7252E-05	H3K27ac	BF
chr1	18325395	18325396	INS	chr1_18288255_18289311	6.72503119	25.0483609	6.9051E-09	7.7252E-05	H3K27ac	BF
chr1	20834452	20834453	INS	chr1_20714004_20714874	6.63121906	25.0122847	6.9843E-09	7.7252E-05	H3K27ac	BF
chr1	268866273	268866274	INS	chr1_268432236_268432721	14.0723289	24.9351974	7.1569E-09	7.8866E-05	H3K27ac	BF

chr4	71017558	71017841	DEL	chr4_70902373_70902789	7.66039344	24.9226102	7.1855E-09	7.8887E-05	H3K27ac	BF
chr3	12956131	12956132	INS	chr3_13332522_13332856	6.97681183	24.875107	7.2948E-09	7.9495E-05	H3K27ac	BF
chr3	13085123	13085124	INS	chr3_13332522_13332856	6.97681183	24.875107	7.2948E-09	7.9495E-05	H3K27ac	BF
chr3	118208700	118208701	INS	chr3_118685202_118685473	9.00937283	24.8358555	7.3864E-09	7.9904E-05	H3K27ac	BF
chr3	118224570	118224571	INS	chr3_118685202_118685473	9.00937283	24.8358555	7.3864E-09	7.9904E-05	H3K27ac	BF
chr1	8356436	8356437	INS	chr1_8530724_8531095	20.6533738	53.8807172	1.5612E-11	8.0886E-07	H3K27ac	BF
chr5	73936605	73937297	DEL	chr5_73570294_73570756	29.8109714	116.243635	3.3523E-14	8.25E-09	H3K27ac	BF
chr5	73971208	73971315	DEL	chr5_73570294_73570756	29.8109714	116.243635	3.3523E-14	8.25E-09	H3K27ac	BF
chr2	126596215	126596496	DEL	chr2_126605561_126606664	10.9246923	137.492521	8.7564E-15	8.25E-09	H3K27ac	BF
chr5	73149236	73149237	INS	chr5_73570294_73570756	29.8109714	116.243635	3.3523E-14	8.25E-09	H3K27ac	BF
chr5	73805971	73805972	INS	chr5_73570294_73570756	29.8109714	116.243635	3.3523E-14	8.25E-09	H3K27ac	BF
chr5	73924522	73924523	INS	chr5_73570294_73570756	29.8109714	116.243635	3.3523E-14	8.25E-09	H3K27ac	BF
chr5	73961834	73961835	INS	chr5_73570294_73570756	29.8109714	116.243635	3.3523E-14	8.25E-09	H3K27ac	BF

chr5	73947094	73947095	INS	chr5_73570294_73570756	29.8109714	116.243635	3.3523E-14	8.25E-09	H3K27ac	BF
chr1	11626419	11626420	INS	chr1_12056570_12057415	14.5026931	125.426707	1.8252E-14	8.25E-09	H3K27ac	BF
chr2	126596215	126596496	DEL	chr2_127095018_127095259	8.64585581	36.7559643	3.2913E-10	8.4521E-06	H3K27ac	BF
chr1	205959403	205959404	INS	chr1_206282904_206283196	9.80701119	53.4540716	1.6634E-11	8.4697E-07	H3K27ac	BF
chr2	6850528	6850529	INS	chr2_7009500_7009765	6.89126983	36.6052587	3.4007E-10	8.5837E-06	H3K27ac	BF
chr2	6875000	6875001	INS	chr2_7009500_7009765	6.89126983	36.6052587	3.4007E-10	8.5837E-06	H3K27ac	BF
chr5	31074385	31074674	DEL	chr5_30839292_30840124	7.318457	24.5638402	8.0585E-09	8.5915E-05	H3K27ac	BF
chr5	31256984	31257057	DEL	chr5_30839292_30840124	7.318457	24.5638402	8.0585E-09	8.5915E-05	H3K27ac	BF
chr5	31336204	31336292	DEL	chr5_30839292_30840124	7.318457	24.5638402	8.0585E-09	8.5915E-05	H3K27ac	BF
chr5	30347610	30347611	INS	chr5_30839292_30840124	7.318457	24.5638402	8.0585E-09	8.5915E-05	H3K27ac	BF
chr1	20834452	20834453	INS	chr1_20965972_20967351	6.1463575	36.3870307	3.5664E-10	8.7907E-06	H3K27ac	BF
chr2	126596215	126596496	DEL	chr2_126922473_126922683	8.85767381	36.300986	3.6342E-10	8.8699E-06	H3K27ac	BF
chr1	268866273	268866274	INS	chr1_268919661_268920125	25.6305256	83.6979624	4.6314E-13	9.0248E-08	H3K27ac	BF

chr4	15488020	15488021	INS	chr4_15158289_15159114	11.8782117	36.1251677	3.7773E-10	9.1437E-06	H3K27ac	BF
chr1	129061546	129061547	INS	chr1_129497186_129498842	-16.965226	-36.044792	3.8449E-10	9.2315E-06	H3K27ac	BF
chr2	5508744	5508946	DEL	chr2_5927773_5928332	14.3653468	24.2518191	8.9153E-09	9.2383E-05	H3K27ac	BF
chr2	6040838	6041097	DEL	chr2_5927773_5928332	14.3653468	24.2518191	8.9153E-09	9.2383E-05	H3K27ac	BF
chr2	6388855	6388951	DEL	chr2_5927773_5928332	14.3653468	24.2518191	8.9153E-09	9.2383E-05	H3K27ac	BF
chr4	123880440	123881468	DEL	chr4_123468159_123468460	13.1306682	24.2528072	8.9125E-09	9.2383E-05	H3K27ac	BF
chr5	31001378	31001673	DEL	chr5_30582944_30583123	6.0820625	24.256217	8.9026E-09	9.2383E-05	H3K27ac	BF
chr2	5661857	5661858	INS	chr2_5927773_5928332	14.3653468	24.2518191	8.9153E-09	9.2383E-05	H3K27ac	BF
chr2	6040580	6040581	INS	chr2_5927773_5928332	14.3653468	24.2518191	8.9153E-09	9.2383E-05	H3K27ac	BF
chr4	123879499	123879500	INS	chr4_123468159_123468460	13.1306682	24.2528072	8.9125E-09	9.2383E-05	H3K27ac	BF
chr4	71017558	71017841	DEL	chr4_71035535_71035817	12.0518428	52.6056087	1.8899E-11	9.2764E-07	H3K27ac	BF
chr3	8236218	8236219	INS	chr3_8638275_8638881	21.0222537	52.5151329	1.9161E-11	9.2764E-07	H3K27ac	BF
chr1	29844104	29844105	INS	chr1_29919726_29921342	18.413155	52.6787327	1.8691E-11	9.2764E-07	H3K27ac	BF

chr5	31001378	31001673	DEL	chr5_30610400_30610571	6.28115369	24.1934687	9.0867E-09	9.3829E-05	H3K27ac	BF
chr1	6643056	6643057	INS	chr1_6995693_6997695	9.46615063	24.1663373	9.1676E-09	9.4335E-05	H3K27ac	BF
chr1	104052039	104052552	DEL	chr1_103688372_103689128	17.2880534	81.9886565	5.4617E-13	9.4881E-08	H3K27ac	BF
chr1	205959403	205959404	INS	chr1_205992110_205993680	8.13926725	24.098676	9.373E-09	9.6114E-05	H3K27ac	BF
chr2	45579565	45579883	DEL	chr2_45606164_45608115	3.72858644	19.8956818	4.2448E-08	0.00577398	H3K27me3	BF
chr2	45586949	45587222	DEL	chr2_45606164_45608115	3.72858644	19.8956818	4.2448E-08	0.00577398	H3K27me3	BF
chr2	45588150	45589844	DEL	chr2_45606164_45608115	3.72858644	19.8956818	4.2448E-08	0.00577398	H3K27me3	BF
chr2	45595976	45596276	DEL	chr2_45606164_45608115	3.72858644	19.8956818	4.2448E-08	0.00577398	H3K27me3	BF
chr2	45588150	45588287	DEL	chr2_45606164_45608115	3.72858644	19.8956818	4.2448E-08	0.00577398	H3K27me3	BF
chr2	45605878	45608297	DUP	chr2_45606164_45608115	7.45717289	19.8956818	4.2448E-08	0.00577398	H3K27me3	BF
chr5	39989084	39989085	INS	chr5_40184235_40184912	5.07706761	23.3350076	1.2088E-08	0.00577398	H3K27me3	BF
chr2	44387292	44387293	INS	chr2_44251831_44252444	-3.576954	-24.783504	7.5107E-09	0.00577398	H3K27me3	BF
chr2	45555215	45555216	INS	chr2_45606164_45608115	3.72858644	19.8956818	4.2448E-08	0.00577398	H3K27me3	BF

chr2	45548726	45548727	INS	chr2_45606164_45608115	3.72858644	19.8956818	4.2448E-08	0.00577398	H3K27me3	BF
chr2	45570410	45570411	INS	chr2_45606164_45608115	3.72858644	19.8956818	4.2448E-08	0.00577398	H3K27me3	BF
chr2	45565638	45565639	INS	chr2_45606164_45608115	3.72858644	19.8956818	4.2448E-08	0.00577398	H3K27me3	BF
chr2	45591258	45591259	INS	chr2_45606164_45608115	3.72858644	19.8956818	4.2448E-08	0.00577398	H3K27me3	BF
chr2	45650548	45650549	INS	chr2_45606164_45608115	3.72858644	19.8956818	4.2448E-08	0.00577398	H3K27me3	BF
chr2	45616793	45616794	INS	chr2_45606164_45608115	3.72858644	19.8956818	4.2448E-08	0.00577398	H3K27me3	BF
chr2	45717532	45717533	INS	chr2_45606164_45608115	3.72858644	19.8956818	4.2448E-08	0.00577398	H3K27me3	BF
chr2	45721112	45721113	INS	chr2_45606164_45608115	3.72858644	19.8956818	4.2448E-08	0.00577398	H3K27me3	BF
chr2	45719661	45719662	INS	chr2_45606164_45608115	3.72858644	19.8956818	4.2448E-08	0.00577398	H3K27me3	BF
chr2	45802510	45802511	INS	chr2_45606164_45608115	3.72858644	19.8956818	4.2448E-08	0.00577398	H3K27me3	BF
chr8	130493532	130493824	DEL	chr8_130529078_130529532	2.41329494	18.9923622	6.1134E-08	0.00708376	H3K27me3	BF
chr8	130343584	130343585	INS	chr8_130529078_130529532	2.41329494	18.9923622	6.1134E-08	0.00708376	H3K27me3	BF
chr8	130490911	130490912	INS	chr8_130529078_130529532	2.41329494	18.9923622	6.1134E-08	0.00708376	H3K27me3	BF

chr8	130613881	130613882	INS	chr8_130529078_130529532	2.41329494	18.9923622	6.1134E-08	0.00708376	H3K27me3	BF
chr16	71107204	71107204	BND	chr16_71105932_71107092	4.49529813	17.5749938	1.1222E-07	0.01210643	H3K27me3	BF
chr16	70996251	70996252	INS	chr16_71105932_71107092	2.24764906	17.5749938	1.1222E-07	0.01210643	H3K27me3	BF
chr1	142151990	142152063	DEL	chr1_142269835_142270820	-2.476244	-16.506135	1.8315E-07	0.01909942	H3K27me3	BF
chr7	78694627	78694896	DEL	chr7_78646710_78647125	-1.4946505	-15.574577	2.8783E-07	0.02904859	H3K27me3	BF
chr9	51715367	51715419	DEL	chr9_51803321_51804110	-2.1092663	-13.733029	7.6228E-07	0.04332693	H3K27me3	BF
chr9	51762931	51763191	DEL	chr9_51803321_51804110	-2.1092663	-13.733029	7.6228E-07	0.04332693	H3K27me3	BF
chr18	49127000	49127110	DEL	chr18_49261651_49262017	4.20435133	13.8486467	7.1457E-07	0.04332693	H3K27me3	BF
chr18	49357122	49357300	DEL	chr18_49261651_49262017	2.10217567	13.8486467	7.1457E-07	0.04332693	H3K27me3	BF
chr6	72699732	72703660	DEL	chr6_72892086_72892877	-2.7101218	-13.702334	7.7554E-07	0.04332693	H3K27me3	BF
chr6	72718969	72719283	DEL	chr6_72892086_72892877	-2.7101218	-13.702334	7.7554E-07	0.04332693	H3K27me3	BF
chr2	58016681	58024078	DEL	chr2_58165525_58166607	2.44747062	13.9371151	6.8032E-07	0.04332693	H3K27me3	BF
chr1	142151990	142152063	DEL	chr1_142244437_142245373	-1.903127	-14.115061	6.1686E-07	0.04332693	H3K27me3	BF

chr18	49125075	49125076	INS	chr18_49261651_49262017	2.10217567	13.8486467	7.1457E-07	0.04332693	H3K27me3	BF
chr18	49355645	49355646	INS	chr18_49261651_49262017	2.10217567	13.8486467	7.1457E-07	0.04332693	H3K27me3	BF
chr6	72760073	72760074	INS	chr6_72892086_72892877	-5.4202436	-13.702334	7.7554E-07	0.04332693	H3K27me3	BF
chr6	72847306	72847307	INS	chr6_72892086_72892877	-2.7101218	-13.702334	7.7554E-07	0.04332693	H3K27me3	BF
chr6	72945988	72945989	INS	chr6_72892086_72892877	2.7101218	13.7023337	7.7554E-07	0.04332693	H3K27me3	BF
chr10	33973565	33973566	INS	chr10_34114120_34114415	-1.3224619	-13.98248	6.6347E-07	0.04332693	H3K27me3	BF
chr15	113631840	113631841	INS	chr15_113498548_113498829	1.76441	14.25575	5.7136E-07	0.04332693	H3K27me3	BF
chr15	123842296	123842297	INS	chr15_123656845_123657192	2.44043067	13.9197341	6.869E-07	0.04332693	H3K27me3	BF
chr7	109110416	109110417	INS	chr7_109075423_109075725	-0.952696	-13.736309	7.6088E-07	0.04332693	H3K27me3	BF
chr1	240979836	240979837	INS	chr1_241027323_241027684	-0.6814936	-13.884275	7.0055E-07	0.04332693	H3K27me3	BF
chr14	16620363	16620364	INS	chr14_16619696_16620322	1.73533167	14.0408438	6.4248E-07	0.04332693	H3K27me3	BF
chr17	40726869	40726970	DEL	chr17_40529084_40530257	1.68682063	13.5260477	8.5687E-07	0.04622004	H3K27me3	BF
chr7	54408762	54408763	INS	chr7_54276368_54276816	-2.1279607	-13.545945	8.4723E-07	0.04622004	H3K27me3	BF

chr3	58398188	58398189	INS	chr3_58477654_58478373	3.53391213	35.4639966	4.3752E-10	0.00054291	H3K4me1	BF
chr3	58568874	58568875	INS	chr3_58477654_58478373	3.53391213	35.4639966	4.3752E-10	0.00054291	H3K4me1	BF
chr9	90870107	90870108	INS	chr9_90916256_90917330	1.87067044	28.4440531	2.5229E-09	0.00234794	H3K4me1	BF
chr2	4682489	4682545	DEL	chr2_4544962_4546039	4.20634975	23.8085628	1.0314E-08	0.0042662	H3K4me1	BF
chr2	4696179	4696361	DEL	chr2_4544962_4546039	4.20634975	23.8085628	1.0314E-08	0.0042662	H3K4me1	BF
chr2	4411235	4411236	INS	chr2_4544962_4546039	4.20634975	23.8085628	1.0314E-08	0.0042662	H3K4me1	BF
chr2	4544282	4544283	INS	chr2_4544962_4546039	4.20634975	23.8085628	1.0314E-08	0.0042662	H3K4me1	BF
chr2	4581207	4581208	INS	chr2_4544962_4546039	4.20634975	23.8085628	1.0314E-08	0.0042662	H3K4me1	BF
chr11	20770920	20771218	DEL	chr11_20848313_20849743	0.94718452	21.7029299	2.1416E-08	0.00797224	H3K4me1	BF
chr7	110443691	110443947	DEL	chr7_110385939_110389700	-2.6649883	-20.179466	3.7977E-08	0.00826969	H3K4me1	BF
chr15	25474114	25474411	DEL	chr15_25471768_25472450	1.5880735	20.0892354	3.9338E-08	0.00826969	H3K4me1	BF
chr15	25485211	25485601	DEL	chr15_25471768_25472450	1.5880735	20.0892354	3.9338E-08	0.00826969	H3K4me1	BF
chr15	25530577	25530957	DEL	chr15_25471768_25472450	1.5880735	20.0892354	3.9338E-08	0.00826969	H3K4me1	BF

chr2	6491451	6491452	INS	chr2_6531383_6531792	3.342583	20.5579581	3.2814E-08	0.00826969	H3K4me1	BF
chr2	6493181	6493182	INS	chr2_6531383_6531792	3.342583	20.5579581	3.2814E-08	0.00826969	H3K4me1	BF
chr7	112536843	112536844	INS	chr7_112602481_112603522	1.29865129	20.0474926	3.9987E-08	0.00826969	H3K4me1	BF
chr15	25451700	25451701	INS	chr15_25471768_25472450	1.5880735	20.0892354	3.9338E-08	0.00826969	H3K4me1	BF
chr11	5033796	5033797	INS	chr11_5026878_5027747	1.94072269	19.6702389	4.6424E-08	0.00864079	H3K4me1	BF
chr2	143513144	143513145	INS	chr2_143584821_143585587	1.87659175	19.3495746	5.2818E-08	0.00936292	H3K4me1	BF
chr3	19587087	19587182	DEL	chr3_19395360_19395784	1.1170432	18.9717633	6.1657E-08	0.00997923	H3K4me1	BF
chr3	108228150	108228151	INS	chr3_108422640_108423123	2.51246156	18.9738443	6.1604E-08	0.00997923	H3K4me1	BF
chr3	79569783	79571355	DEL	chr3_79477828_79478668	0.91573444	18.1532796	8.711E-08	0.01158124	H3K4me1	BF
chr3	79656298	79656348	DEL	chr3_79477828_79478668	0.91573444	18.1532796	8.711E-08	0.01158124	H3K4me1	BF
chr3	79387484	79387485	INS	chr3_79477828_79478668	0.91573444	18.1532796	8.711E-08	0.01158124	H3K4me1	BF
chr3	79466100	79466101	INS	chr3_79477828_79478668	0.91573444	18.1532796	8.711E-08	0.01158124	H3K4me1	BF
chr8	41416313	41416314	INS	chr8_41545536_41547450	5.29827125	18.4509784	7.669E-08	0.01158124	H3K4me1	BF

chr3	118226827	118227098	DEL	chr3_118225739_118227025	1.44549469	17.9920617	9.341E-08	0.01199061	H3K4me1	BF
NW_018085246.1	75482	75532	DEL	NW_018085246.1_137104_138121	-2.1835509	-17.607087	1.1063E-07	0.0121127	H3K4me1	BF
NW_018085246.1	224079	224366	DEL	NW_018085246.1_137104_138121	-2.1835509	-17.607087	1.1063E-07	0.0121127	H3K4me1	BF
NW_018085246.1	294074	294137	DEL	NW_018085246.1_137104_138121	-2.1835509	-17.607087	1.1063E-07	0.0121127	H3K4me1	BF
NW_018085246.1	328807	329087	DEL	NW_018085246.1_137104_138121	-2.1835509	-17.607087	1.1063E-07	0.0121127	H3K4me1	BF
NW_018085246.1	305762	305763	INS	NW_018085246.1_137104_138121	-2.1835509	-17.607087	1.1063E-07	0.0121127	H3K4me1	BF
chr1	5225520	5225521	INS	chr1_5364105_5364911	1.43531572	17.3629102	1.2339E-07	0.01241435	H3K4me1	BF
chr1	5232649	5232650	INS	chr1_5364105_5364911	1.43531572	17.3629102	1.2339E-07	0.01241435	H3K4me1	BF
chr1	5401874	5401875	INS	chr1_5364105_5364911	1.43531572	17.3629102	1.2339E-07	0.01241435	H3K4me1	BF
chr2	143472503	143472504	INS	chr2_143584821_143585587	1.88329597	17.2823084	1.2796E-07	0.01253517	H3K4me1	BF
chr8	41416313	41416314	INS	chr8_41491375_41493127	1.69415438	17.1386867	1.3658E-07	0.01303636	H3K4me1	BF
chr3	49247217	49248009	DEL	chr3_49155976_49157466	4.23658389	16.2357944	2.0829E-07	0.01833937	H3K4me1	BF
chr3	49249961	49250068	DEL	chr3_49155976_49157466	4.23658389	16.2357944	2.0829E-07	0.01833937	H3K4me1	BF

chr13	25722752	25722818	DEL	chr13_25560972_25561698	2.41232478	16.0432033	2.2857E-07	0.01833937	H3K4me1	BF
chr6	163225816	163226069	DEL	chr6_163212586_163213509	1.03849194	16.0165689	2.3155E-07	0.01833937	H3K4me1	BF
chr6	163224661	163224886	DEL	chr6_163212586_163213509	1.03849194	16.0165689	2.3155E-07	0.01833937	H3K4me1	BF
chr13	25435743	25435744	INS	chr13_25560972_25561698	2.41232478	16.0432033	2.2857E-07	0.01833937	H3K4me1	BF
chr3	58398188	58398189	INS	chr3_58476361_58477411	3.78369075	15.9011077	2.4495E-07	0.01860946	H3K4me1	BF
chr3	58568874	58568875	INS	chr3_58476361_58477411	3.78369075	15.9011077	2.4495E-07	0.01860946	H3K4me1	BF
chr10	56205692	56205693	INS	chr10_56080785_56081460	-0.4884495	-15.691784	2.7154E-07	0.02021679	H3K4me1	BF
chr13	21894931	21895379	DEL	chr13_22058948_22059209	2.79322494	15.3218311	3.2683E-07	0.02047074	H3K4me1	BF
chr3	112451069	112451465	DEL	chr3_112484898_112485464	1.408193	15.5146043	2.9659E-07	0.02047074	H3K4me1	BF
chr13	195429968	195430247	DEL	chr13_195616150_195617025	1.41524656	15.161599	3.5461E-07	0.02047074	H3K4me1	BF
chr13	195428830	195429690	DEL	chr13_195616150_195617025	1.41524656	15.161599	3.5461E-07	0.02047074	H3K4me1	BF
chr13	195510602	195510927	DEL	chr13_195616150_195617025	1.41524656	15.161599	3.5461E-07	0.02047074	H3K4me1	BF
chr13	195768403	195768833	DEL	chr13_195616150_195617025	1.41524656	15.161599	3.5461E-07	0.02047074	H3K4me1	BF

chr8	19195001	19195309	DEL	chr8_19330694_19331048	1.41382661	15.3700229	3.1896E-07	0.02047074	H3K4me1	BF
chr5	23783563	23783669	DEL	chr5_23722953_23724505	1.0786166	15.116321	3.6294E-07	0.02047074	H3K4me1	BF
chr13	22066560	22066561	INS	chr13_22058948_22059209	2.79322494	15.3218311	3.2683E-07	0.02047074	H3K4me1	BF
chr13	22070807	22070808	INS	chr13_22058948_22059209	2.79322494	15.3218311	3.2683E-07	0.02047074	H3K4me1	BF
chr8	120258664	120258665	INS	chr8_120253103_120254093	2.53408105	15.2350779	3.4156E-07	0.02047074	H3K4me1	BF
chr8	120383219	120383220	INS	chr8_120253103_120254093	2.53408105	15.2350779	3.4156E-07	0.02047074	H3K4me1	BF
chr2	130946052	130946053	INS	chr2_130938753_130939726	1.92685672	15.4509484	3.0622E-07	0.02047074	H3K4me1	BF
chr2	130947217	130947218	INS	chr2_130938753_130939726	1.92685672	15.4509484	3.0622E-07	0.02047074	H3K4me1	BF
chr13	195516967	195516968	INS	chr13_195616150_195617025	1.41524656	15.161599	3.5461E-07	0.02047074	H3K4me1	BF
chr5	23722702	23722703	INS	chr5_23722953_23724505	1.0786166	15.116321	3.6294E-07	0.02047074	H3K4me1	BF
chr14	29845588	29845589	INS	chr14_29818764_29819723	3.77823375	15.0514727	3.7524E-07	0.02054228	H3K4me1	BF
chr6	30762592	30762592	BND	chr6_30716382_30716739	1.73983179	14.7236423	4.4506E-07	0.02096616	H3K4me1	BF
chr10	54843220	54843684	DEL	chr10_54831184_54831552	2.90445528	14.6946519	4.5191E-07	0.02096616	H3K4me1	BF

chr6	578524	582296	DEL	chr6_578685_580305	-4.1705178	-14.676704	4.562E-07	0.02096616	H3K4me1	BF
chr6	657188	657537	DEL	chr6_578685_580305	-4.1705178	-14.676704	4.562E-07	0.02096616	H3K4me1	BF
chr8	41290153	41294443	DUP	chr8_41421537_41422519	3.4234725	14.6963478	4.515E-07	0.02096616	H3K4me1	BF
chr7	43571868	43571869	INS	chr7_43574534_43575492	1.09791056	14.7314927	4.4323E-07	0.02096616	H3K4me1	BF
chr16	6651299	6651300	INS	chr16_6787210_6789299	1.11974367	14.8581889	4.1478E-07	0.02096616	H3K4me1	BF
chr16	6793483	6793484	INS	chr16_6787210_6789299	1.11974367	14.8581889	4.1478E-07	0.02096616	H3K4me1	BF
chr6	527354	527355	INS	chr6_578685_580305	-4.1705178	-14.676704	4.562E-07	0.02096616	H3K4me1	BF
chr6	586825	586826	INS	chr6_578685_580305	-4.1705178	-14.676704	4.562E-07	0.02096616	H3K4me1	BF
chr6	30899618	30899619	INS	chr6_30716382_30716739	1.73983179	14.7236423	4.4506E-07	0.02096616	H3K4me1	BF
chr8	41416313	41416314	INS	chr8_41421537_41422519	1.71173625	14.6963478	4.515E-07	0.02096616	H3K4me1	BF
chr11	2741070	2741252	DEL	chr11_2701838_2702541	1.19442089	14.2050146	5.8732E-07	0.02267796	H3K4me1	BF
chr11	2743815	2743868	DEL	chr11_2701838_2702541	1.19442089	14.2050146	5.8732E-07	0.02267796	H3K4me1	BF
chr11	2789307	2789469	DEL	chr11_2701838_2702541	1.19442089	14.2050146	5.8732E-07	0.02267796	H3K4me1	BF

chr14	74047398	74047812	DEL	chr14_73959429_73960971	-1.8190602	-14.014556	6.5184E-07	0.02267796	H3K4me1	BF
chr3	119771892	119772203	DEL	chr3_119934248_119934700	1.83642493	14.0301033	6.4629E-07	0.02267796	H3K4me1	BF
chr3	119901923	119902228	DEL	chr3_119934248_119934700	1.83642493	14.0301033	6.4629E-07	0.02267796	H3K4me1	BF
chr13	10917768	10917821	DEL	chr13_10873764_10874463	1.60085292	14.1844737	5.9392E-07	0.02267796	H3K4me1	BF
chr13	10944020	10944291	DEL	chr13_10873764_10874463	1.60085292	14.1844737	5.9392E-07	0.02267796	H3K4me1	BF
chr11	2744435	2744436	INS	chr11_2701838_2702541	1.19442089	14.2050146	5.8732E-07	0.02267796	H3K4me1	BF
chr8	73786360	73786361	INS	chr8_73774992_73776519	1.13822943	14.321466	5.5141E-07	0.02267796	H3K4me1	BF
chr14	73764356	73764357	INS	chr14_73959429_73960971	-1.8190602	-14.014556	6.5184E-07	0.02267796	H3K4me1	BF
chr14	73822571	73822572	INS	chr14_73959429_73960971	-1.8190602	-14.014556	6.5184E-07	0.02267796	H3K4me1	BF
chr14	74043768	74043769	INS	chr14_73959429_73960971	-1.8190602	-14.014556	6.5184E-07	0.02267796	H3K4me1	BF
chr3	108228150	108228151	INS	chr3_108423443_108423761	2.29779144	14.1389114	6.0887E-07	0.02267796	H3K4me1	BF
chr9	115485709	115485710	INS	chr9_115528939_115529496	1.91047475	14.2373064	5.7711E-07	0.02267796	H3K4me1	BF
chr9	115625232	115625233	INS	chr9_115528939_115529496	1.91047475	14.2373064	5.7711E-07	0.02267796	H3K4me1	BF

chr9	115628862	115628863	INS	chr9_115528939_115529496	1.91047475	14.2373064	5.7711E-07	0.02267796	H3K4me1	BF
chr14	9793129	9793130	INS	chr14_9609569_9611604	0.79487375	14.1639452	6.0061E-07	0.02267796	H3K4me1	BF
chr3	30667296	30667297	INS	chr3_30603740_30604904	3.09898089	14.0588901	6.3614E-07	0.02267796	H3K4me1	BF
chr3	30747394	30747395	INS	chr3_30603740_30604904	3.09898089	14.0588901	6.3614E-07	0.02267796	H3K4me1	BF
chr18	36293554	36293555	INS	chr18_36373259_36373730	2.14898519	14.3222924	5.5117E-07	0.02267796	H3K4me1	BF
chr13	10882771	10882772	INS	chr13_10873764_10874463	1.60085292	14.1844737	5.9392E-07	0.02267796	H3K4me1	BF
chr14	91492330	91495425	DEL	chr14_91463048_91463801	1.28473911	13.90824	6.9129E-07	0.02369389	H3K4me1	BF
chr9	15948993	15949202	DEL	chr9_15865992_15866676	1.24257479	13.7738623	7.4503E-07	0.02369389	H3K4me1	BF
chr6	2695235	2695294	DEL	chr6_2704262_2704855	1.649672	13.8088958	7.3058E-07	0.02369389	H3K4me1	BF
chr6	2702923	2703210	DEL	chr6_2704262_2704855	1.649672	13.8088958	7.3058E-07	0.02369389	H3K4me1	BF
chr5	43127408	43127409	INS	chr5_43307543_43308113	2.19157736	13.7444165	7.5742E-07	0.02369389	H3K4me1	BF
chr9	15976644	15976645	INS	chr9_15865992_15866676	1.24257479	13.7738623	7.4503E-07	0.02369389	H3K4me1	BF
chr8	41416313	41416314	INS	chr8_41395429_41395950	1.07636413	13.7594088	7.5109E-07	0.02369389	H3K4me1	BF

chr15	52261103	52261104	INS	chr15_52226497_52228432	-1.1908862	-13.787086	7.3954E-07	0.02369389	H3K4me1	BF
chr15	131706025	131706092	DEL	chr15_131570950_131572460	2.92052644	13.6505864	7.9848E-07	0.02476997	H3K4me1	BF
chr13	17797768	17798071	DEL	chr13_17832326_17832656	1.34015875	13.3727901	9.3539E-07	0.02483017	H3K4me1	BF
chr13	17832979	17833124	DEL	chr13_17832326_17832656	1.34015875	13.3727901	9.3539E-07	0.02483017	H3K4me1	BF
chr13	17865917	17866227	DEL	chr13_17832326_17832656	1.34015875	13.3727901	9.3539E-07	0.02483017	H3K4me1	BF
chr13	17892939	17893235	DEL	chr13_17832326_17832656	1.34015875	13.3727901	9.3539E-07	0.02483017	H3K4me1	BF
chr13	17952563	17952934	DEL	chr13_17832326_17832656	1.34015875	13.3727901	9.3539E-07	0.02483017	H3K4me1	BF
chr13	17953833	17954148	DEL	chr13_17832326_17832656	1.34015875	13.3727901	9.3539E-07	0.02483017	H3K4me1	BF
chr7	80984705	80984879	DEL	chr7_81173979_81174339	1.58127549	13.4394831	9.0027E-07	0.02483017	H3K4me1	BF
chr7	81186681	81186743	DEL	chr7_81173979_81174339	1.58127549	13.4394831	9.0027E-07	0.02483017	H3K4me1	BF
chr4	127348711	127348987	DEL	chr4_127416669_127418009	1.78846942	13.3029286	9.7384E-07	0.02483017	H3K4me1	BF
chr4	127413705	127413774	DEL	chr4_127416669_127418009	1.78846942	13.3029286	9.7384E-07	0.02483017	H3K4me1	BF
chr4	127432274	127432554	DEL	chr4_127416669_127418009	1.78846942	13.3029286	9.7384E-07	0.02483017	H3K4me1	BF

chr4	127494914	127495246	DEL	chr4_127416669_127418009	1.78846942	13.3029286	9.7384E-07	0.02483017	H3K4me1	BF
chr13	17815254	17815255	INS	chr13_17832326_17832656	1.34015875	13.3727901	9.3539E-07	0.02483017	H3K4me1	BF
chr13	17826440	17826441	INS	chr13_17832326_17832656	1.34015875	13.3727901	9.3539E-07	0.02483017	H3K4me1	BF
chr13	17899311	17899312	INS	chr13_17832326_17832656	1.34015875	13.3727901	9.3539E-07	0.02483017	H3K4me1	BF
chr13	17915770	17915771	INS	chr13_17832326_17832656	1.34015875	13.3727901	9.3539E-07	0.02483017	H3K4me1	BF
chr13	18005338	18005339	INS	chr13_17832326_17832656	1.34015875	13.3727901	9.3539E-07	0.02483017	H3K4me1	BF
chr9	64167836	64167837	INS	chr9_64253845_64255202	1.26704611	13.3141825	9.6753E-07	0.02483017	H3K4me1	BF
chr9	64221136	64221137	INS	chr9_64253845_64255202	1.26704611	13.3141825	9.6753E-07	0.02483017	H3K4me1	BF
chr7	80981485	80981486	INS	chr7_81173979_81174339	1.58127549	13.4394831	9.0027E-07	0.02483017	H3K4me1	BF
chr2	128802593	128802594	INS	chr2_128873627_128873929	2.25268889	13.3227839	9.6274E-07	0.02483017	H3K4me1	BF
chr13	185374893	185374893	BND	chr13_185366282_185367063	0.93658117	13.2087279	1.0285E-06	0.02569614	H3K4me1	BF
chr13	185374836	185374836	BND	chr13_185366282_185367063	0.93658117	13.2087279	1.0285E-06	0.02569614	H3K4me1	BF
chr6	42173511	42173563	DEL	chr6_42349541_42350891	0.553371	13.2316043	1.0149E-06	0.02569614	H3K4me1	BF

chr13	40200565	40200566	INS	chr13_40305547_40306870	1.92325278	13.097188	1.0977E-06	0.02707231	H3K4me1	BF
chr3	5257375	5257376	INS	chr3_5337563_5337977	1.49239667	13.0965695	1.0981E-06	0.02707231	H3K4me1	BF
chr5	35738926	35739036	DEL	chr5_35770370_35770726	1.98713944	12.988046	1.1706E-06	0.02811323	H3K4me1	BF
chr5	35734081	35734206	DEL	chr5_35770370_35770726	1.98713944	12.988046	1.1706E-06	0.02811323	H3K4me1	BF
chr5	35864767	35865108	DEL	chr5_35770370_35770726	1.98713944	12.988046	1.1706E-06	0.02811323	H3K4me1	BF
chr13	1038184	1038185	INS	chr13_946949_947566	1.9055035	13.0007259	1.1618E-06	0.02811323	H3K4me1	BF
chr6	156626994	156627055	DEL	chr6_156824528_156825793	1.77192625	12.8873308	1.2426E-06	0.02894702	H3K4me1	BF
chr12	54521446	54521447	INS	chr12_54660686_54661232	1.18361617	12.8852188	1.2442E-06	0.02894702	H3K4me1	BF
chr12	54525533	54525534	INS	chr12_54660686_54661232	1.18361617	12.8852188	1.2442E-06	0.02894702	H3K4me1	BF
chr9	133884982	133884983	INS	chr9_133761427_133763275	1.44650323	12.9006697	1.2328E-06	0.02894702	H3K4me1	BF
chr1	71663643	71663721	DEL	chr1_71598772_71599196	1.82467139	12.8242286	1.2903E-06	0.02946693	H3K4me1	BF
chr1	71648947	71648948	INS	chr1_71598772_71599196	1.82467139	12.8242286	1.2903E-06	0.02946693	H3K4me1	BF
chr4	80885079	80885361	DEL	chr4_80894682_80895703	0.8008378	12.5294773	1.5417E-06	0.03165297	H3K4me1	BF

chr4	80874391	80874669	DEL	chr4_80894682_80895703	0.8008378	12.5294773	1.5417E-06	0.03165297	H3K4me1	BF
chr13	39728296	39728605	DEL	chr13_39853261_39853749	1.94679444	12.6592044	1.4249E-06	0.03165297	H3K4me1	BF
chr6	157435437	157435708	DEL	chr6_157238017_157239191	1.921909	12.6777358	1.409E-06	0.03165297	H3K4me1	BF
chr1	12993725	12993779	DEL	chr1_12999060_12999765	0.7410968	12.5880952	1.4877E-06	0.03165297	H3K4me1	BF
chr1	13032285	13032589	DEL	chr1_12999060_12999765	0.7410968	12.5880952	1.4877E-06	0.03165297	H3K4me1	BF
chr11	20537295	20537348	DEL	chr11_20657352_20658291	1.512525	12.4531001	1.6156E-06	0.03165297	H3K4me1	BF
chr11	20603666	20603863	DEL	chr11_20657352_20658291	-1.512525	-12.4531	1.6156E-06	0.03165297	H3K4me1	BF
chr11	20654803	20655098	DEL	chr11_20657352_20658291	1.512525	12.4531001	1.6156E-06	0.03165297	H3K4me1	BF
chr11	20685274	20685324	DEL	chr11_20657352_20658291	-1.512525	-12.4531	1.6156E-06	0.03165297	H3K4me1	BF
chr11	20705070	20705394	DEL	chr11_20657352_20658291	1.512525	12.4531001	1.6156E-06	0.03165297	H3K4me1	BF
chr11	20761366	20761648	DEL	chr11_20657352_20658291	1.512525	12.4531001	1.6156E-06	0.03165297	H3K4me1	BF
chr18	21887681	21887974	DEL	chr18_21936662_21937254	1.05565017	12.6111878	1.4669E-06	0.03165297	H3K4me1	BF
chr8	4225136	4225137	INS	chr8_4250671_4251284	0.87899106	12.523851	1.5471E-06	0.03165297	H3K4me1	BF

chr4	80856801	80856802	INS	chr4_80894682_80895703	0.8008378	12.5294773	1.5417E-06	0.03165297	H3K4me1	BF
chr7	4327186	4327187	INS	chr7_4391748_4392064	3.56718049	12.6299208	1.4504E-06	0.03165297	H3K4me1	BF
chr6	157265863	157265864	INS	chr6_157238017_157239191	1.921909	12.6777358	1.409E-06	0.03165297	H3K4me1	BF
chr5	7725285	7725286	INS	chr5_7749300_7750228	2.18535257	12.4636964	1.6051E-06	0.03165297	H3K4me1	BF
chr5	7791185	7791186	INS	chr5_7749300_7750228	2.18535257	12.4636964	1.6051E-06	0.03165297	H3K4me1	BF
chr11	20654151	20654152	INS	chr11_20657352_20658291	1.512525	12.4531001	1.6156E-06	0.03165297	H3K4me1	BF
chr17	11702847	11702848	INS	chr17_11704381_11705261	1.61388108	12.5559015	1.5171E-06	0.03165297	H3K4me1	BF
chr3	2632066	2632067	INS	chr3_2834452_2835889	1.39471406	12.5344695	1.5371E-06	0.03165297	H3K4me1	BF
chr3	2759063	2759064	INS	chr3_2834452_2835889	1.39471406	12.5344695	1.5371E-06	0.03165297	H3K4me1	BF
chr3	2847790	2847791	INS	chr3_2834452_2835889	1.39471406	12.5344695	1.5371E-06	0.03165297	H3K4me1	BF
chr3	2940616	2940617	INS	chr3_2834452_2835889	1.39471406	12.5344695	1.5371E-06	0.03165297	H3K4me1	BF
NW_018085246.1	216603	216604	INS	NW_018085246.1_140380_141331	1.91683807	12.4103669	1.6586E-06	0.03232571	H3K4me1	BF
chr10	66704707	66704708	INS	chr10_66601366_66602381	0.93933306	12.3789902	1.691E-06	0.03261574	H3K4me1	BF

chr17	7800055	7800056	INS	chr17_7933176_7933672	0.9625455	12.3810633	1.6888E-06	0.03261574	H3K4me1	BF
chr2	24518579	24518663	DEL	chr2_24645869_24646862	2.69137383	12.3534789	1.7179E-06	0.03262684	H3K4me1	BF
chr13	129827922	129827923	INS	chr13_129841519_129841804	1.47789181	12.3549213	1.7163E-06	0.03262684	H3K4me1	BF
chr2	24784421	24784422	INS	chr2_24645869_24646862	2.69137383	12.3534789	1.7179E-06	0.03262684	H3K4me1	BF
chr18	40773993	40773994	INS	chr18_40676163_40676830	2.29487489	12.3246411	1.7488E-06	0.03304606	H3K4me1	BF
chr14	16014685	16014686	INS	chr14_15824265_15824614	1.44935311	12.2929093	1.7836E-06	0.03353304	H3K4me1	BF
chr8	41290153	41294443	DUP	chr8_41394814_41395116	2.86985938	12.2466957	1.8356E-06	0.03399643	H3K4me1	BF
chr8	41416313	41416314	INS	chr8_41394814_41395116	1.43492969	12.2466957	1.8356E-06	0.03399643	H3K4me1	BF
chr1	18874985	18875191	DEL	chr1_19042310_19042783	2.30549313	12.1829288	1.9103E-06	0.03502996	H3K4me1	BF
chr10	1425099	1425100	INS	chr10_1483967_1484567	1.00277556	12.1905496	1.9012E-06	0.03502996	H3K4me1	BF
chr6	145333125	145333201	DEL	chr6_145345708_145346528	1.70841556	12.0514289	2.0751E-06	0.03670253	H3K4me1	BF
chr1	243956235	243957710	DEL	chr1_243907521_243909276	-1.4326973	-12.025161	2.1099E-06	0.03670253	H3K4me1	BF
chr6	145322959	145322960	INS	chr6_145345708_145346528	1.70841556	12.0514289	2.0751E-06	0.03670253	H3K4me1	BF

chr6	145344252	145344253	INS	chr6_145345708_145346528	1.70841556	12.0514289	2.0751E-06	0.03670253	H3K4me1	BF
chr1	243952071	243952072	INS	chr1_243907521_243909276	-1.4326973	-12.025161	2.1099E-06	0.03670253	H3K4me1	BF
chr1	243962224	243962225	INS	chr1_243907521_243909276	-1.4326973	-12.025161	2.1099E-06	0.03670253	H3K4me1	BF
chr8	14288158	14288159	INS	chr8_14238544_14241292	0.74405905	12.0316325	2.1013E-06	0.03670253	H3K4me1	BF
chr7	115743049	115743050	INS	chr7_115687181_115687726	1.46679683	11.9826683	2.1676E-06	0.03753077	H3K4me1	BF
chr11	65521017	65521345	DEL	chr11_65414307_65415341	0.84027475	11.8943563	2.2932E-06	0.03795002	H3K4me1	BF
chr3	120022205	120022472	DEL	chr3_120022673_120024304	1.199375	11.6680219	2.6541E-06	0.03795002	H3K4me1	BF
chr3	5357277	5361074	DEL	chr3_5337563_5337977	1.49393313	11.7906291	2.4513E-06	0.03795002	H3K4me1	BF
chr11	6394079	6395867	DEL	chr11_6501325_6502453	1.49296938	11.7021012	2.5959E-06	0.03795002	H3K4me1	BF
chr14	2991326	2991602	DEL	chr14_2991793_2992409	1.83236313	11.8701727	2.3291E-06	0.03795002	H3K4me1	BF
chr1	260621493	260621612	DEL	chr1_260626149_260627531	-1.7960196	-11.881333	2.3125E-06	0.03795002	H3K4me1	BF
chr15	23603461	23603733	DEL	chr15_23761519_23763114	1.01540611	11.6772842	2.6382E-06	0.03795002	H3K4me1	BF
chr14	20922171	20923458	DEL	chr14_20991694_20992238	1.30038222	11.8192642	2.4065E-06	0.03795002	H3K4me1	BF

chr14	21137248	21137308	DEL	chr14_20991694_20992238	1.30038222	11.8192642	2.4065E-06	0.03795002	H3K4me1	BF
chr17	20990368	20990650	DEL	chr17_20955435_20957303	1.33141345	11.6425577	2.6986E-06	0.03795002	H3K4me1	BF
chr17	21021488	21021607	DEL	chr17_20955435_20957303	1.33141345	11.6425577	2.6986E-06	0.03795002	H3K4me1	BF
chr16	54027939	54028136	DEL	chr16_54029139_54030077	0.83420361	11.7521476	2.5131E-06	0.03795002	H3K4me1	BF
chr16	54162114	54162213	DEL	chr16_54029139_54030077	0.83420361	11.7521476	2.5131E-06	0.03795002	H3K4me1	BF
chr11	65436639	65436640	INS	chr11_65414307_65415341	0.84027475	11.8943563	2.2932E-06	0.03795002	H3K4me1	BF
chr6	92549517	92549518	INS	chr6_92586193_92586498	2.37807017	11.7730363	2.4793E-06	0.03795002	H3K4me1	BF
chr6	92659971	92659972	INS	chr6_92586193_92586498	2.37807017	11.7730363	2.4793E-06	0.03795002	H3K4me1	BF
chr6	92661435	92661436	INS	chr6_92586193_92586498	2.37807017	11.7730363	2.4793E-06	0.03795002	H3K4me1	BF
chr3	76043857	76043858	INS	chr3_76078555_76079990	0.94938875	11.7836449	2.4624E-06	0.03795002	H3K4me1	BF
chr3	86835949	86835950	INS	chr3_86796984_86797410	1.7538625	11.8966229	2.2899E-06	0.03795002	H3K4me1	BF
chr15	112892312	112892313	INS	chr15_112752102_112752492	1.69430721	11.8154919	2.4124E-06	0.03795002	H3K4me1	BF
chr3	86842526	86842527	INS	chr3_86796984_86797410	1.7538625	11.8966229	2.2899E-06	0.03795002	H3K4me1	BF

chr1	98223171	98223172	INS	chr1_98199823_98200558	-1.814527	-11.748952	2.5183E-06	0.03795002	H3K4me1	BF
chr3	119848403	119848404	INS	chr3_120022673_120024304	1.199375	11.6680219	2.6541E-06	0.03795002	H3K4me1	BF
chr3	120086208	120086209	INS	chr3_120022673_120024304	1.199375	11.6680219	2.6541E-06	0.03795002	H3K4me1	BF
chr5	100369269	100369270	INS	chr5_100386899_100387954	2.009781	11.8373528	2.3787E-06	0.03795002	H3K4me1	BF
chr5	100361622	100361623	INS	chr5_100386899_100387954	2.009781	11.8373528	2.3787E-06	0.03795002	H3K4me1	BF
chr5	100582690	100582691	INS	chr5_100386899_100387954	2.009781	11.8373528	2.3787E-06	0.03795002	H3K4me1	BF
chr9	5527723	5527724	INS	chr9_5681041_5681978	2.81073511	11.6350985	2.7117E-06	0.03795002	H3K4me1	BF
chr9	5553999	5554000	INS	chr9_5681041_5681978	2.81073511	11.6350985	2.7117E-06	0.03795002	H3K4me1	BF
chr9	5680674	5680675	INS	chr9_5681041_5681978	2.81073511	11.6350985	2.7117E-06	0.03795002	H3K4me1	BF
chr9	5691805	5691806	INS	chr9_5681041_5681978	2.81073511	11.6350985	2.7117E-06	0.03795002	H3K4me1	BF
chr9	5840411	5840412	INS	chr9_5681041_5681978	2.81073511	11.6350985	2.7117E-06	0.03795002	H3K4me1	BF
chr1	260474112	260474113	INS	chr1_260626149_260627531	-1.7960196	-11.881333	2.3125E-06	0.03795002	H3K4me1	BF
chr1	260621212	260621213	INS	chr1_260626149_260627531	-1.7960196	-11.881333	2.3125E-06	0.03795002	H3K4me1	BF

chr15	23605616	23605617	INS	chr15_23761519_23763114	1.01540611	11.6772842	2.6382E-06	0.03795002	H3K4me1	BF
chr15	23604008	23604009	INS	chr15_23761519_23763114	1.01540611	11.6772842	2.6382E-06	0.03795002	H3K4me1	BF
chr14	20810527	20810528	INS	chr14_20991694_20992238	1.30038222	11.8192642	2.4065E-06	0.03795002	H3K4me1	BF
chr14	20825521	20825522	INS	chr14_20991694_20992238	1.30038222	11.8192642	2.4065E-06	0.03795002	H3K4me1	BF
chr14	20977070	20977071	INS	chr14_20991694_20992238	1.30038222	11.8192642	2.4065E-06	0.03795002	H3K4me1	BF
chr14	21135909	21135910	INS	chr14_20991694_20992238	1.30038222	11.8192642	2.4065E-06	0.03795002	H3K4me1	BF
chr7	24928677	24928678	INS	chr7_25002149_25003325	-1.1558938	-11.667533	2.655E-06	0.03795002	H3K4me1	BF
chr7	24940061	24940062	INS	chr7_25002149_25003325	-1.1558938	-11.667533	2.655E-06	0.03795002	H3K4me1	BF
chr7	24946429	24946430	INS	chr7_25002149_25003325	-1.1558938	-11.667533	2.655E-06	0.03795002	H3K4me1	BF
chr7	25048818	25048819	INS	chr7_25002149_25003325	-1.1558938	-11.667533	2.655E-06	0.03795002	H3K4me1	BF
chr2	3115224	3115225	INS	chr2_3126995_3127697	1.28297611	11.9227461	2.252E-06	0.03795002	H3K4me1	BF
chr2	3157413	3157414	INS	chr2_3126995_3127697	1.28297611	11.9227461	2.252E-06	0.03795002	H3K4me1	BF
chr9	5524752	5531905	INV	chr9_5681041_5681978	2.81073511	11.6350985	2.7117E-06	0.03795002	H3K4me1	BF

chr7	80984705	80984879	DEL	chr7_81176162_81176468	2.19891042	11.5909545	2.7912E-06	0.03848314	H3K4me1	BF
chr7	81186681	81186743	DEL	chr7_81176162_81176468	2.19891042	11.5909545	2.7912E-06	0.03848314	H3K4me1	BF
chr9	33391970	33392269	DEL	chr9_33395410_33398293	-0.83332	-11.595989	2.782E-06	0.03848314	H3K4me1	BF
chr7	80981485	80981486	INS	chr7_81176162_81176468	2.19891042	11.5909545	2.7912E-06	0.03848314	H3K4me1	BF
chr1	268975962	268976235	DEL	chr1_269076953_269077975	1.2137825	11.5586255	2.851E-06	0.03916304	H3K4me1	BF
chr3	58398188	58398189	INS	chr3_58470297_58472135	3.24420688	11.5044257	2.9546E-06	0.0401412	H3K4me1	BF
chr3	58568874	58568875	INS	chr3_58470297_58472135	3.24420688	11.5044257	2.9546E-06	0.0401412	H3K4me1	BF
chr5	101800527	101800528	INS	chr5_101914313_101916342	0.96486356	11.5150054	2.934E-06	0.0401412	H3K4me1	BF
chr9	136034948	136035029	DEL	chr9_136198609_136199656	1.38992773	11.4802939	3.002E-06	0.04049039	H3K4me1	BF
chr9	136051138	136051139	INS	chr9_136198609_136199656	1.38992773	11.4802939	3.002E-06	0.04049039	H3K4me1	BF
chr7	58967003	58967004	INS	chr7_59126636_59127562	2.846795	11.4262347	3.1115E-06	0.04174693	H3K4me1	BF
chr15	108717764	108717765	INS	chr15_108569662_108570373	0.96769417	11.4232565	3.1176E-06	0.04174693	H3K4me1	BF
chr6	136913322	136913323	INS	chr6_136944898_136946395	1.13372078	11.3945817	3.1776E-06	0.04239766	H3K4me1	BF

chr15	86623650	86624175	DEL	chr15_86791985_86792341	1.18895706	11.3753768	3.2185E-06	0.04263769	H3K4me1	BF
chr15	86627595	86627596	INS	chr15_86791985_86792341	1.18895706	11.3753768	3.2185E-06	0.04263769	H3K4me1	BF
chr18	50334620	50334741	DEL	chr18_50486492_50487931	-5.4378732	-11.223881	3.5626E-06	0.04325905	H3K4me1	BF
chr18	50412657	50414366	DEL	chr18_50486492_50487931	-5.4378732	-11.223881	3.5626E-06	0.04325905	H3K4me1	BF
chr11	48279936	48280225	DEL	chr11_48444444_48445266	-0.8153744	-11.291352	3.4045E-06	0.04325905	H3K4me1	BF
chr14	82100623	82101119	DEL	chr14_82114175_82116056	-2.3227899	-11.260105	3.4767E-06	0.04325905	H3K4me1	BF
chr14	82157378	82157686	DEL	chr14_82114175_82116056	-2.3227899	-11.260105	3.4767E-06	0.04325905	H3K4me1	BF
chr6	148597915	148597994	DEL	chr6_148646263_148646591	1.73768282	11.2169849	3.5792E-06	0.04325905	H3K4me1	BF
chr1	8807134	8808692	DEL	chr1_8809496_8810462	2.9624725	11.2347671	3.5365E-06	0.04325905	H3K4me1	BF
chr1	8817246	8817423	DEL	chr1_8809496_8810462	2.9624725	11.2347671	3.5365E-06	0.04325905	H3K4me1	BF
chr1	8818772	8818834	DEL	chr1_8809496_8810462	2.9624725	11.2347671	3.5365E-06	0.04325905	H3K4me1	BF
chr1	8827722	8828022	DEL	chr1_8809496_8810462	2.9624725	11.2347671	3.5365E-06	0.04325905	H3K4me1	BF
chr7	22807665	22807798	DEL	chr7_22612687_22614210	0.77437438	11.2512006	3.4976E-06	0.04325905	H3K4me1	BF

chr18	50352302	50352303	INS	chr18_50486492_50487931	-5.4378732	-11.223881	3.5626E-06	0.04325905	H3K4me1	BF
chr18	50578194	50578195	INS	chr18_50486492_50487931	-5.4378732	-11.223881	3.5626E-06	0.04325905	H3K4me1	BF
chr18	50681374	50681375	INS	chr18_50486492_50487931	-5.4378732	-11.223881	3.5626E-06	0.04325905	H3K4me1	BF
chr14	82099622	82099623	INS	chr14_82114175_82116056	-2.3227899	-11.260105	3.4767E-06	0.04325905	H3K4me1	BF
chr2	93339574	93339575	INS	chr2_93458467_93458903	0.85258161	11.2389778	3.5265E-06	0.04325905	H3K4me1	BF
chr5	88716728	88716729	INS	chr5_88535507_88535711	0.67654075	11.2990616	3.3869E-06	0.04325905	H3K4me1	BF
chr4	125691956	125691957	INS	chr4_125496134_125496639	1.15592594	11.2569421	3.4841E-06	0.04325905	H3K4me1	BF
chr6	148712317	148712318	INS	chr6_148646263_148646591	1.73768282	11.2169849	3.5792E-06	0.04325905	H3K4me1	BF
chr6	148763290	148763291	INS	chr6_148646263_148646591	1.73768282	11.2169849	3.5792E-06	0.04325905	H3K4me1	BF
chr1	8818417	8818418	INS	chr1_8809496_8810462	2.9624725	11.2347671	3.5365E-06	0.04325905	H3K4me1	BF
chr17	5088777	5088778	INS	chr17_5011729_5012578	1.65305113	11.2870208	3.4144E-06	0.04325905	H3K4me1	BF
chr14	24059356	24059357	INS	chr14_24071452_24071747	1.73975806	11.2474624	3.5064E-06	0.04325905	H3K4me1	BF
chr1	44087119	44087120	INS	chr1_44263726_44264436	1.24820667	11.3289005	3.3199E-06	0.04325905	H3K4me1	BF

chr1	44132637	44132638	INS	chr1_44263726_44264436	1.24820667	11.3289005	3.3199E-06	0.04325905	H3K4me1	BF
chr1	59755857	59755858	INS	chr1_59579572_59579931	1.83565117	11.3099629	3.3623E-06	0.04325905	H3K4me1	BF
chr1	59739801	59739802	INS	chr1_59579572_59579931	1.83565117	11.3099629	3.3623E-06	0.04325905	H3K4me1	BF
chr6	136149575	136149576	INS	chr6_136257887_136258575	2.27237839	11.1367656	3.7789E-06	0.0452326	H3K4me1	BF
chr2	2300873	2300873	BND	chr2_2155859_2157623	1.74633846	11.1223878	3.816E-06	0.04541289	H3K4me1	BF
chr18	6803274	6803404	DEL	chr18_6648241_6648966	0.78583933	11.1106582	3.8466E-06	0.04541289	H3K4me1	BF
chr10	44800205	44800206	INS	chr10_44602617_44603281	1.14806367	11.0889926	3.9038E-06	0.04541289	H3K4me1	BF
chr18	14074088	14074089	INS	chr18_14271543_14271831	0.51494681	11.0976365	3.8808E-06	0.04541289	H3K4me1	BF
chr4	108977508	108977509	INS	chr4_108954264_108954697	1.50385267	11.0730251	3.9465E-06	0.04576724	H3K4me1	BF
chr5	88495960	88496236	DEL	chr5_88392164_88392966	1.12144136	11.0114187	4.1165E-06	0.04744232	H3K4me1	BF
chr5	88420855	88420856	INS	chr5_88392164_88392966	1.12144136	11.0114187	4.1165E-06	0.04744232	H3K4me1	BF
chr8	112167699	112169435	DEL	chr8_112167366_112168492	-1.5719159	-10.976458	4.2165E-06	0.04756473	H3K4me1	BF
chr13	127922896	127923186	DEL	chr13_127851836_127852127	1.11182679	10.9818699	4.2009E-06	0.04756473	H3K4me1	BF

chr2	6491451	6491452	INS	chr2_6526037_6527711	2.1889175	10.9825504	4.1989E-06	0.04756473	H3K4me1	BF
chr2	6493181	6493182	INS	chr2_6526037_6527711	2.1889175	10.9825504	4.1989E-06	0.04756473	H3K4me1	BF
chr13	127840816	127840817	INS	chr13_127851836_127852127	1.11182679	10.9818699	4.2009E-06	0.04756473	H3K4me1	BF
chr13	127852373	127852374	INS	chr13_127851836_127852127	1.11182679	10.9818699	4.2009E-06	0.04756473	H3K4me1	BF
chr7	92805141	92805213	DEL	chr7_92823824_92824510	0.90962413	10.9316693	4.3487E-06	0.04794625	H3K4me1	BF
NW_018085246.1	75482	75532	DEL	NW_018085246.1_140380_141331	-1.7953732	-10.911401	4.41E-06	0.04794625	H3K4me1	BF
NW_018085246.1	224079	224366	DEL	NW_018085246.1_140380_141331	-1.7953732	-10.911401	4.41E-06	0.04794625	H3K4me1	BF
NW_018085246.1	294074	294137	DEL	NW_018085246.1_140380_141331	-1.7953732	-10.911401	4.41E-06	0.04794625	H3K4me1	BF
NW_018085246.1	328807	329087	DEL	NW_018085246.1_140380_141331	-1.7953732	-10.911401	4.41E-06	0.04794625	H3K4me1	BF
chr9	16985678	16985955	DEL	chr9_16860553_16861244	1.31021067	10.8962836	4.4564E-06	0.04794625	H3K4me1	BF
chr2	24518579	24518663	DEL	chr2_24644945_24645237	2.45006583	10.9027189	4.4366E-06	0.04794625	H3K4me1	BF
chr7	92764982	92764983	INS	chr7_92823824_92824510	0.90962413	10.9316693	4.3487E-06	0.04794625	H3K4me1	BF
chr7	92809599	92809600	INS	chr7_92823824_92824510	0.90962413	10.9316693	4.3487E-06	0.04794625	H3K4me1	BF

chr4	126986938	126986939	INS	chr4_127113274_127113780	3.00725347	10.9436526	4.3129E-06	0.04794625	H3K4me1	BF
chr4	127303393	127303394	INS	chr4_127113274_127113780	3.00725347	10.9436526	4.3129E-06	0.04794625	H3K4me1	BF
chr3	5328850	5328851	INS	chr3_5343730_5343989	1.26596214	10.903705	4.4336E-06	0.04794625	H3K4me1	BF
NW_018085246.1	305762	305763	INS	NW_018085246.1_140380_141331	-1.7953732	-10.911401	4.41E-06	0.04794625	H3K4me1	BF
chr9	16713644	16713645	INS	chr9_16860553_16861244	1.31021067	10.8962836	4.4564E-06	0.04794625	H3K4me1	BF
chr9	16994089	16994090	INS	chr9_16860553_16861244	1.31021067	10.8962836	4.4564E-06	0.04794625	H3K4me1	BF
chr2	24784421	24784422	INS	chr2_24644945_24645237	2.45006583	10.9027189	4.4366E-06	0.04794625	H3K4me1	BF
chr1	221894303	221894304	INS	chr1_221826229_221827106	3.11123061	10.8804245	4.5057E-06	0.0482747	H3K4me1	BF
chr3	40045289	40045290	INS	chr3_39942043_39942333	5.15291111	10.878114	4.5129E-06	0.0482747	H3K4me1	BF
chr14	61051438	61051439	INS	chr14_60936663_60937397	2.31474144	10.8642151	4.5566E-06	0.04860284	H3K4me1	BF
chr16	32419559	32419560	INS	chr16_32350069_32350378	3.51785878	10.8210286	4.6955E-06	0.04979939	H3K4me1	BF
chr5	100885004	100885004	BND	chr5_100763071_100766086	5.25268375	34.4379474	5.526E-10	0.00012426	H3K4me3	BF
chr6	27072003	27072310	DEL	chr6_27508712_27509849	8.60077444	32.2407204	9.3324E-10	0.00012426	H3K4me3	BF

chr6	27156306	27156375	DEL	chr6_27508712_27509849	8.60077444	32.2407204	9.3324E-10	0.00012426	H3K4me3	BF
chr6	27614341	27614930	DEL	chr6_27508712_27509849	8.60077444	32.2407204	9.3324E-10	0.00012426	H3K4me3	BF
chr6	27671069	27671242	DEL	chr6_27508712_27509849	8.60077444	32.2407204	9.3324E-10	0.00012426	H3K4me3	BF
chr6	27821965	27822250	DEL	chr6_27508712_27509849	8.60077444	32.2407204	9.3324E-10	0.00012426	H3K4me3	BF
chr6	110756053	110756054	INS	chr6_110679335_110680788	2.65305922	32.9560681	7.839E-10	0.00012426	H3K4me3	BF
chr6	27499402	27499403	INS	chr6_27508712_27509849	8.60077444	32.2407204	9.3324E-10	0.00012426	H3K4me3	BF
chr11	25436047	25436048	INS	chr11_25705251_25707052	-8.6333475	-33.274606	7.262E-10	0.00012426	H3K4me3	BF
NW_018085211.1	29713	29714	INS	chr11_25705251_25707052	-8.6333475	-33.274606	7.262E-10	0.00012426	H3K4me3	BF
chr11	2766927	2767100	DEL	chr11_2441602_2442716	2.50974988	28.2478124	2.6653E-09	0.00033946	H3K4me3	BF
chr14	138967024	138967474	DEL	chr14_138968086_138968740	1.67278194	25.8465923	5.3874E-09	0.00065757	H3K4me3	BF
chr17	10893104	10893105	INS	chr17_10758661_10758831	3.76924144	25.1167984	6.7577E-09	0.00079183	H3K4me3	BF
chr7	26538363	26538364	INS	chr7_26791373_26793188	2.48996075	23.9361276	9.8879E-09	0.00111405	H3K4me3	BF
chr1	142151990	142152063	DEL	chr1_142450176_142451699	-9.2716169	-23.462982	1.1577E-08	0.00125604	H3K4me3	BF

chr7	53574936	53574986	DEL	chr7_53666271_53667725	2.6601835	22.4672963	1.6301E-08	0.00170545	H3K4me3	BF
chr7	53911834	53912138	DEL	chr7_53665670_53665864	3.34366483	21.2253756	2.5518E-08	0.00178212	H3K4me3	BF
chr10	65558869	65559714	DEL	chr10_65548799_65550607	6.04766183	21.4408541	2.3567E-08	0.00178212	H3K4me3	BF
chr10	65748216	65749559	DEL	chr10_65548799_65550607	6.04766183	21.4408541	2.3567E-08	0.00178212	H3K4me3	BF
chr7	53994934	53994935	INS	chr7_53665670_53665864	3.34366483	21.2253756	2.5518E-08	0.00178212	H3K4me3	BF
chr10	65246062	65246063	INS	chr10_65548799_65550607	6.04766183	21.4408541	2.3567E-08	0.00178212	H3K4me3	BF
chr10	65248466	65248467	INS	chr10_65548799_65550607	6.04766183	21.4408541	2.3567E-08	0.00178212	H3K4me3	BF
chr10	65558502	65558503	INS	chr10_65548799_65550607	6.04766183	21.4408541	2.3567E-08	0.00178212	H3K4me3	BF
chr10	65702783	65702784	INS	chr10_65548799_65550607	6.04766183	21.4408541	2.3567E-08	0.00178212	H3K4me3	BF
chr10	65801565	65801566	INS	chr10_65548799_65550607	6.04766183	21.4408541	2.3567E-08	0.00178212	H3K4me3	BF
chr10	65957118	65957119	INS	chr10_65548799_65550607	6.04766183	21.4408541	2.3567E-08	0.00178212	H3K4me3	BF
chr9	40043727	40043728	INS	chr9_40005941_40006844	5.496165	21.2218909	2.5551E-08	0.00178212	H3K4me3	BF
chr9	39887950	39888158	INV	chr9_40005941_40006844	5.496165	21.2218909	2.5551E-08	0.00178212	H3K4me3	BF

chr11	1323114	1323179	DEL	chr11_1422536_1423236	3.31155328	20.6854828	3.1256E-08	0.00179527	H3K4me3	BF
chr11	1324176	1324348	DEL	chr11_1422536_1423236	3.31155328	20.6854828	3.1256E-08	0.00179527	H3K4me3	BF
chr11	1859558	1859780	DEL	chr11_1422536_1423236	3.31155328	20.6854828	3.1256E-08	0.00179527	H3K4me3	BF
chr11	1860535	1860720	DEL	chr11_1422536_1423236	3.31155328	20.6854828	3.1256E-08	0.00179527	H3K4me3	BF
chr11	1862349	1862737	DEL	chr11_1422536_1423236	3.31155328	20.6854828	3.1256E-08	0.00179527	H3K4me3	BF
chr11	1318281	1318282	INS	chr11_1422536_1423236	3.31155328	20.6854828	3.1256E-08	0.00179527	H3K4me3	BF
chr11	1744077	1744078	INS	chr11_1422536_1423236	3.31155328	20.6854828	3.1256E-08	0.00179527	H3K4me3	BF
chr11	1811146	1811147	INS	chr11_1422536_1423236	3.31155328	20.6854828	3.1256E-08	0.00179527	H3K4me3	BF
chr11	1847050	1847051	INS	chr11_1422536_1423236	3.31155328	20.6854828	3.1256E-08	0.00179527	H3K4me3	BF
chr7	53911834	53912138	DEL	chr7_53666271_53667725	2.63756478	19.7119017	4.5658E-08	0.00186364	H3K4me3	BF
chr2	148119463	148119776	DEL	chr2_148519138_148519907	2.02022461	19.6692375	4.6442E-08	0.00186364	H3K4me3	BF
chr7	2070266	2070544	DEL	chr7_1925802_1926079	4.65022739	20.0975409	3.9211E-08	0.00186364	H3K4me3	BF
chr17	40108721	40108722	INS	chr17_40537252_40539302	9.70518817	19.8957074	4.2448E-08	0.00186364	H3K4me3	BF

chr17	40950025	40950026	INS	chr17_40537252_40539302	9.70518817	19.8957074	4.2448E-08	0.00186364	H3K4me3	BF
chr7	53994934	53994935	INS	chr7_53666271_53667725	2.63756478	19.7119017	4.5658E-08	0.00186364	H3K4me3	BF
chr5	77783286	77783287	INS	chr5_77486786_77489173	2.23240125	19.72126	4.5489E-08	0.00186364	H3K4me3	BF
chr1	7220531	7220532	INS	chr1_7678939_7681347	5.798125	20.0225064	4.0381E-08	0.00186364	H3K4me3	BF
chr1	7607934	7607935	INS	chr1_7678939_7681347	5.798125	20.0225064	4.0381E-08	0.00186364	H3K4me3	BF
chr1	7679742	7679743	INS	chr1_7678939_7681347	5.798125	20.0225064	4.0381E-08	0.00186364	H3K4me3	BF
chr1	8031907	8031908	INS	chr1_7678939_7681347	5.798125	20.0225064	4.0381E-08	0.00186364	H3K4me3	BF
chr2	148121124	148121125	INS	chr2_148519138_148519907	2.02022461	19.6692375	4.6442E-08	0.00186364	H3K4me3	BF
chr2	148556528	148556529	INS	chr2_148519138_148519907	2.02022461	19.6692375	4.6442E-08	0.00186364	H3K4me3	BF
chr2	148806350	148806351	INS	chr2_148519138_148519907	2.02022461	19.6692375	4.6442E-08	0.00186364	H3K4me3	BF
chr2	148890161	148890162	INS	chr2_148519138_148519907	2.02022461	19.6692375	4.6442E-08	0.00186364	H3K4me3	BF
chr7	1465919	1465920	INS	chr7_1925802_1926079	4.65022739	20.0975409	3.9211E-08	0.00186364	H3K4me3	BF
chr7	2085290	2085291	INS	chr7_1925802_1926079	4.65022739	20.0975409	3.9211E-08	0.00186364	H3K4me3	BF

chr7	2297683	2297684	INS	chr7_1925802_1926079	4.65022739	20.0975409	3.9211E-08	0.00186364	H3K4me3	BF
chr17	10893104	10893105	INS	chr17_10760917_10762495	3.228524	20.0345394	4.019E-08	0.00186364	H3K4me3	BF
chr4	93338145	93338146	INS	chr4_93383994_93384750	15.4132187	19.4721247	5.0264E-08	0.00193741	H3K4me3	BF
chr4	93629793	93629794	INS	chr4_93383994_93384750	15.4132187	19.4721247	5.0264E-08	0.00193741	H3K4me3	BF
chr14	1125326	1125619	DEL	chr14_994593_995876	1.51620375	18.7829254	6.6688E-08	0.0021706	H3K4me3	BF
chr14	1335137	1335303	DEL	chr14_994593_995876	1.51620375	18.7829254	6.6688E-08	0.0021706	H3K4me3	BF
chr3	49595300	49595301	INS	chr3_50091520_50091836	1.64008367	18.9370634	6.2548E-08	0.0021706	H3K4me3	BF
chr14	749171	749172	INS	chr14_994593_995876	1.51620375	18.7829254	6.6688E-08	0.0021706	H3K4me3	BF
chr14	740838	740839	INS	chr14_994593_995876	1.51620375	18.7829254	6.6688E-08	0.0021706	H3K4me3	BF
chr14	861435	861436	INS	chr14_994593_995876	1.51620375	18.7829254	6.6688E-08	0.0021706	H3K4me3	BF
chr14	912821	912822	INS	chr14_994593_995876	1.51620375	18.7829254	6.6688E-08	0.0021706	H3K4me3	BF
chr14	956293	956294	INS	chr14_994593_995876	1.51620375	18.7829254	6.6688E-08	0.0021706	H3K4me3	BF
chr14	1131376	1131377	INS	chr14_994593_995876	1.51620375	18.7829254	6.6688E-08	0.0021706	H3K4me3	BF

chr14	1354846	1354847	INS	chr14_994593_995876	1.51620375	18.7829254	6.6688E-08	0.0021706	H3K4me3	BF
chr14	1414040	1414041	INS	chr14_994593_995876	1.51620375	18.7829254	6.6688E-08	0.0021706	H3K4me3	BF
NW_018084866.1	139421	139422	INS	NW_018084866.1_138909_139246	2.03887283	18.9198463	6.2996E-08	0.0021706	H3K4me3	BF
NW_018084866.1	120236	120237	INS	NW_018084866.1_138909_139246	2.03887283	18.9198463	6.2996E-08	0.0021706	H3K4me3	BF
chr3	49883104	49886365	INV	chr3_50091520_50091836	1.64008367	18.9370634	6.2548E-08	0.0021706	H3K4me3	BF
chr5	26377270	26377271	INS	chr5_26266170_26266445	2.19972067	18.6063788	7.1812E-08	0.00228657	H3K4me3	BF
chr18	49457013	49457014	INS	chr18_49577676_49577947	2.14554694	18.2200914	8.464E-08	0.00263768	H3K4me3	BF
chr18	50042739	50042740	INS	chr18_49577676_49577947	2.14554694	18.2200914	8.464E-08	0.00263768	H3K4me3	BF
chr9	134888654	134888655	INS	chr9_135096939_135098043	-6.481535	-18.140209	8.7603E-08	0.00267313	H3K4me3	BF
chr9	135076250	135076251	INS	chr9_135096939_135098043	-6.481535	-18.140209	8.7603E-08	0.00267313	H3K4me3	BF
chr5	88338289	88338498	DEL	chr5_88433389_88434396	2.667774	17.5784516	1.1205E-07	0.00325645	H3K4me3	BF
chr8	124961592	124963864	DEL	chr8_124702983_124703296	2.04952111	17.6309057	1.0947E-07	0.00325645	H3K4me3	BF
chr14	20240682	20240742	DEL	chr14_20575677_20576485	1.83092006	17.4867409	1.1672E-07	0.00325645	H3K4me3	BF

chr14	20922171	20923458	DEL	chr14_20575677_20576485	1.83092006	17.4867409	1.1672E-07	0.00325645	H3K4me3	BF
chr3	3924276	3924277	INS	chr3_4362340_4363950	-16.215358	-17.625947	1.0971E-07	0.00325645	H3K4me3	BF
chr14	20235664	20235665	INS	chr14_20575677_20576485	1.83092006	17.4867409	1.1672E-07	0.00325645	H3K4me3	BF
chr14	20810527	20810528	INS	chr14_20575677_20576485	1.83092006	17.4867409	1.1672E-07	0.00325645	H3K4me3	BF
chr14	20825521	20825522	INS	chr14_20575677_20576485	1.83092006	17.4867409	1.1672E-07	0.00325645	H3K4me3	BF
chr14	20977070	20977071	INS	chr14_20575677_20576485	1.83092006	17.4867409	1.1672E-07	0.00325645	H3K4me3	BF
chr1	142151990	142152063	DEL	chr1_142412334_142413814	-22.212732	-17.391947	1.2179E-07	0.00336572	H3K4me3	BF
chr7	8781874	8782518	DEL	chr7_8641549_8641919	3.17584088	17.0847686	1.3998E-07	0.00376191	H3K4me3	BF
chr7	8876101	8876371	DEL	chr7_8641549_8641919	3.17584088	17.0847686	1.3998E-07	0.00376191	H3K4me3	BF
chr7	8856362	8856363	INS	chr7_8641549_8641919	3.17584088	17.0847686	1.3998E-07	0.00376191	H3K4me3	BF
chr5	51731254	51731454	DEL	chr5_52037014_52038078	1.51989481	16.3882023	1.9367E-07	0.00493334	H3K4me3	BF
chr5	51847714	51850721	DEL	chr5_52037014_52038078	1.51989481	16.3882023	1.9367E-07	0.00493334	H3K4me3	BF
chr5	52371516	52372821	DEL	chr5_52037014_52038078	3.03978961	16.3882023	1.9367E-07	0.00493334	H3K4me3	BF

chr5	51900391	51900392	INS	chr5_52037014_52038078	1.51989481	16.3882023	1.9367E-07	0.00493334	H3K4me3	BF
chr5	51997025	51997026	INS	chr5_52037014_52038078	1.51989481	16.3882023	1.9367E-07	0.00493334	H3K4me3	BF
chr6	146886904	146887223	DEL	chr6_147234640_147235510	0.86601092	16.1175595	2.2049E-07	0.00556808	H3K4me3	BF
chr3	1869051	1869811	DEL	chr3_1381908_1383341	8.65827889	15.8085002	2.5634E-07	0.00636365	H3K4me3	BF
chr3	887966	887967	INS	chr3_1381908_1383341	8.65827889	15.8085002	2.5634E-07	0.00636365	H3K4me3	BF
chr7	53574936	53574986	DEL	chr7_53665670_53665864	3.34071975	15.7385096	2.6534E-07	0.00642606	H3K4me3	BF
NW_018084968.1	1411493	1411813	DEL	NW_018084968.1_1701667_1702012	1.25282394	15.7046225	2.6982E-07	0.00642606	H3K4me3	BF
NW_018084968.1	1599681	1599682	INS	NW_018084968.1_1701667_1702012	1.25282394	15.7046225	2.6982E-07	0.00642606	H3K4me3	BF
NW_018084968.1	1355603	1355604	INS	NW_018084968.1_1701667_1702012	1.25282394	15.7046225	2.6982E-07	0.00642606	H3K4me3	BF
NW_018084968.1	1681017	1683006	INV	NW_018084968.1_1701667_1702012	2.50564789	15.7046225	2.6982E-07	0.00642606	H3K4me3	BF
NW_018085018.1	220604	220889	DEL	NW_018085018.1_126936_127427	2.62824733	15.4293667	3.0956E-07	0.00692223	H3K4me3	BF
chr5	16623010	16623011	INS	NW_018085018.1_126936_127427	2.62824733	15.4293667	3.0956E-07	0.00692223	H3K4me3	BF
NW_018085018.1	382448	382449	INS	NW_018085018.1_126936_127427	2.62824733	15.4293667	3.0956E-07	0.00692223	H3K4me3	BF

NW_018085018.1	157153	157154	INS	NW_018085018.1_126936_127427	2.62824733	15.4293667	3.0956E-07	0.00692223	H3K4me3	BF
chr5	16849636	16849637	INS	NW_018085018.1_126936_127427	2.62824733	15.4293667	3.0956E-07	0.00692223	H3K4me3	BF
chr5	16963728	16963729	INS	NW_018085018.1_126936_127427	2.62824733	15.4293667	3.0956E-07	0.00692223	H3K4me3	BF
NW_018084866.1	437391	437392	INS	NW_018084866.1_138909_139246	2.04055975	15.4724279	3.0293E-07	0.00692223	H3K4me3	BF
chr5	40186785	40186834	DEL	chr5_40182743_40184026	7.46356654	15.0700961	3.7166E-07	0.00756065	H3K4me3	BF
chr5	40194281	40194330	DEL	chr5_40182743_40184026	7.46356654	15.0700961	3.7166E-07	0.00756065	H3K4me3	BF
chr11	4444689	4444883	DEL	chr11_4857993_4858799	5.020255	15.0848503	3.6885E-07	0.00756065	H3K4me3	BF
chr11	4445071	4446258	DEL	chr11_4857993_4858799	5.020255	15.0848503	3.6885E-07	0.00756065	H3K4me3	BF
chr11	4858012	4858123	DEL	chr11_4857993_4858799	5.020255	15.0848503	3.6885E-07	0.00756065	H3K4me3	BF
chr11	4871624	4871839	DEL	chr11_4857993_4858799	5.020255	15.0848503	3.6885E-07	0.00756065	H3K4me3	BF
chr5	40181920	40181921	INS	chr5_40182743_40184026	7.46356654	15.0700961	3.7166E-07	0.00756065	H3K4me3	BF
chr11	4500270	4500271	INS	chr11_4857993_4858799	5.020255	15.0848503	3.6885E-07	0.00756065	H3K4me3	BF
chr13	196401490	196401491	INS	chr13_196232105_196233067	1.18641374	15.1832454	3.5071E-07	0.00756065	H3K4me3	BF

chr11	365457	365583	DEL	chr11_735407_735930	2.01698674	14.9225144	4.0113E-07	0.00790719	H3K4me3	BF
chr18	10719297	10719969	DEL	chr18_10919132_10921354	4.8458003	14.8775608	4.1062E-07	0.00790719	H3K4me3	BF
chr3	1350909	1351109	DEL	chr3_1269807_1270433	3.60181569	14.8170554	4.2379E-07	0.00790719	H3K4me3	BF
chr3	1477380	1477559	DEL	chr3_1269807_1270433	3.60181569	14.8170554	4.2379E-07	0.00790719	H3K4me3	BF
chr3	1506478	1506548	DEL	chr3_1269807_1270433	3.60181569	14.8170554	4.2379E-07	0.00790719	H3K4me3	BF
chr6	37985703	37985785	DEL	chr6_38317135_38319173	1.10970519	14.9464645	3.9617E-07	0.00790719	H3K4me3	BF
chr6	38066684	38066753	DEL	chr6_38317135_38319173	1.10970519	14.9464645	3.9617E-07	0.00790719	H3K4me3	BF
chr11	732646	732647	INS	chr11_735407_735930	2.01698674	14.9225144	4.0113E-07	0.00790719	H3K4me3	BF
chr18	10666841	10666842	INS	chr18_10919132_10921354	4.8458003	14.8775608	4.1062E-07	0.00790719	H3K4me3	BF
chr3	1365025	1365026	INS	chr3_1269807_1270433	3.60181569	14.8170554	4.2379E-07	0.00790719	H3K4me3	BF
chr3	1491930	1491931	INS	chr3_1269807_1270433	3.60181569	14.8170554	4.2379E-07	0.00790719	H3K4me3	BF
chr18	10720533	10720821	INV	chr18_10919132_10921354	-9.6916006	-14.877561	4.1062E-07	0.00790719	H3K4me3	BF
chr1	262738472	262738472	BND	chr1_262938037_262938332	5.01784903	14.6357362	4.6618E-07	0.0083263	H3K4me3	BF

chr18	48464563	48464650	DEL	chr18_48605608_48606334	0.86610669	14.6160761	4.7106E-07	0.0083263	H3K4me3	BF
chr15	134149340	134149410	DEL	chr15_134567755_134568361	3.27295967	14.6129824	4.7183E-07	0.0083263	H3K4me3	BF
chr1	263157004	263157122	DEL	chr1_262938037_262938332	2.50892451	14.6357362	4.6618E-07	0.0083263	H3K4me3	BF
chr18	48903896	48903897	INS	chr18_48605608_48606334	0.86610669	14.6160761	4.7106E-07	0.0083263	H3K4me3	BF
chr15	134336590	134336591	INS	chr15_134567755_134568361	1.63647983	14.6129824	4.7183E-07	0.0083263	H3K4me3	BF
chr15	134532036	134532037	INS	chr15_134567755_134568361	1.63647983	14.6129824	4.7183E-07	0.0083263	H3K4me3	BF
chr18	48238756	48238757	INS	chr18_48605608_48606334	0.86610669	14.6160761	4.7106E-07	0.0083263	H3K4me3	BF
chr3	11920833	11920898	DEL	chr3_11942567_11942989	3.128188	14.5051018	4.9968E-07	0.00861019	H3K4me3	BF
chr14	134284616	134284617	INS	chr14_133829344_133830922	-3.5302714	-14.525903	4.9417E-07	0.00861019	H3K4me3	BF
chr3	11637049	11637050	INS	chr3_11942567_11942989	3.128188	14.5051018	4.9968E-07	0.00861019	H3K4me3	BF
chr3	11921302	11921303	INS	chr3_11942567_11942989	3.128188	14.5051018	4.9968E-07	0.00861019	H3K4me3	BF
chr3	125236510	125237128	DEL	chr3_125190519_125190982	1.604995	14.2824377	5.6317E-07	0.00901485	H3K4me3	BF
chr3	125534678	125534905	DEL	chr3_125190519_125190982	1.604995	14.2824377	5.6317E-07	0.00901485	H3K4me3	BF

chr3	124835193	124835194	INS	chr3_125190519_125190982	1.604995	14.2824377	5.6317E-07	0.00901485	H3K4me3	BF
chr3	125038991	125038992	INS	chr3_125190519_125190982	1.604995	14.2824377	5.6317E-07	0.00901485	H3K4me3	BF
chr3	125045994	125045995	INS	chr3_125190519_125190982	1.604995	14.2824377	5.6317E-07	0.00901485	H3K4me3	BF
chr14	133989957	133990007	DEL	chr14_134361125_134361814	3.94036011	14.0735945	6.3103E-07	0.00983241	H3K4me3	BF
chr12	39146270	39146525	DEL	chr12_39354345_39354680	2.27130528	14.0427104	6.4182E-07	0.00983241	H3K4me3	BF
chr12	39434784	39435036	DEL	chr12_39354345_39354680	2.27130528	14.0427104	6.4182E-07	0.00983241	H3K4me3	BF
chr14	134361753	134361754	INS	chr14_134361125_134361814	3.94036011	14.0735945	6.3103E-07	0.00983241	H3K4me3	BF
chr12	39327407	39327408	INS	chr12_39354345_39354680	2.27130528	14.0427104	6.4182E-07	0.00983241	H3K4me3	BF
chr12	39467253	39467254	INS	chr12_39354345_39354680	2.27130528	14.0427104	6.4182E-07	0.00983241	H3K4me3	BF
chr1	33764836	33764899	DEL	chr1_33435279_33435574	2.0869805	13.9936408	6.594E-07	0.00995682	H3K4me3	BF
chr1	33792542	33792621	DEL	chr1_33435279_33435574	2.0869805	13.9936408	6.594E-07	0.00995682	H3K4me3	BF
chr14	136992649	136993899	DEL	chr14_137309371_137310565	2.94717	13.8128543	7.2897E-07	0.01051931	H3K4me3	BF
chr14	137145946	137146018	DEL	chr14_137309371_137310565	2.94717	13.8128543	7.2897E-07	0.01051931	H3K4me3	BF

chr14	137473604	137473913	DEL	chr14_137309371_137310565	2.94717	13.8128543	7.2897E-07	0.01051931	H3K4me3	BF
chr14	136949443	136949444	INS	chr14_137309371_137310565	2.94717	13.8128543	7.2897E-07	0.01051931	H3K4me3	BF
chr14	137059027	137059028	INS	chr14_137309371_137310565	2.94717	13.8128543	7.2897E-07	0.01051931	H3K4me3	BF
chr14	137765463	137765464	INS	chr14_137309371_137310565	2.94717	13.8128543	7.2897E-07	0.01051931	H3K4me3	BF
chr9	40043727	40043728	INS	chr9_39872445_39873489	2.80983117	13.82227	7.2515E-07	0.01051931	H3K4me3	BF
chr9	39887950	39888158	INV	chr9_39872445_39873489	2.80983117	13.82227	7.2515E-07	0.01051931	H3K4me3	BF
chr9	13483314	13483744	DEL	chr9_13330563_13331523	1.41211406	13.8008356	7.3388E-07	0.01053824	H3K4me3	BF
chr8	4771499	4771786	DEL	chr8_4413088_4413627	2.30607306	13.6074553	8.1818E-07	0.01134673	H3K4me3	BF
chr15	136972779	136972949	DEL	chr15_137065595_137066773	2.72119783	13.5518032	8.4441E-07	0.01134673	H3K4me3	BF
chr15	137458503	137458775	DEL	chr15_137065595_137066773	2.72119783	13.5518032	8.4441E-07	0.01134673	H3K4me3	BF
chr15	137457498	137457825	DEL	chr15_137065595_137066773	2.72119783	13.5518032	8.4441E-07	0.01134673	H3K4me3	BF
chr1	142151990	142152063	DEL	chr1_142499806_142501674	-8.9643248	-13.561593	8.3973E-07	0.01134673	H3K4me3	BF
chr6	26940139	26940221	DEL	chr6_26988808_26988965	2.59496306	13.5843519	8.2896E-07	0.01134673	H3K4me3	BF

chr7	3874884	3874885	INS	chr7_4190369_4192699	3.31647125	13.5799955	8.3101E-07	0.01134673	H3K4me3	BF
chr2	68261696	68261697	INS	NW_018085086.1_74579_74994	2.54351694	13.6313041	8.0722E-07	0.01134673	H3K4me3	BF
chr7	4448957	4448958	INS	chr7_4190369_4192699	3.31647125	13.5799955	8.3101E-07	0.01134673	H3K4me3	BF
chr15	137255433	137255434	INS	chr15_137065595_137066773	2.72119783	13.5518032	8.4441E-07	0.01134673	H3K4me3	BF
chr15	137289706	137289707	INS	chr15_137065595_137066773	2.72119783	13.5518032	8.4441E-07	0.01134673	H3K4me3	BF
chr15	137471795	137471796	INS	chr15_137065595_137066773	2.72119783	13.5518032	8.4441E-07	0.01134673	H3K4me3	BF
chr15	137480507	137480508	INS	chr15_137065595_137066773	2.72119783	13.5518032	8.4441E-07	0.01134673	H3K4me3	BF
chr6	27082427	27082428	INS	chr6_26988808_26988965	2.59496306	13.5843519	8.2896E-07	0.01134673	H3K4me3	BF
chr9	81252044	81252465	DEL	chr9_80888758_80890437	1.7388374	13.519238	8.602E-07	0.01140194	H3K4me3	BF
chr9	80658520	80658521	INS	chr9_80888758_80890437	1.7388374	13.519238	8.602E-07	0.01140194	H3K4me3	BF
chr9	81161238	81161239	INS	chr9_80888758_80890437	1.7388374	13.519238	8.602E-07	0.01140194	H3K4me3	BF
chr10	58147556	58149393	DEL	chr10_58316644_58316904	1.937664	13.4663685	8.8653E-07	0.01149107	H3K4me3	BF
chr10	58510003	58510106	DEL	chr10_58316644_58316904	1.937664	13.4663685	8.8653E-07	0.01149107	H3K4me3	BF

chr10	58217606	58217607	INS	chr10_58316644_58316904	1.937664	13.4663685	8.8653E-07	0.01149107	H3K4me3	BF
chr10	58309000	58309001	INS	chr10_58316644_58316904	1.937664	13.4663685	8.8653E-07	0.01149107	H3K4me3	BF
chr11	4930743	4930833	DEL	chr11_5067149_5068286	5.50303911	13.412375	9.1437E-07	0.01164571	H3K4me3	BF
chr11	5289386	5289387	INS	chr11_5067149_5068286	5.50303911	13.412375	9.1437E-07	0.01164571	H3K4me3	BF
chr11	5441144	5441145	INS	chr11_5067149_5068286	5.50303911	13.412375	9.1437E-07	0.01164571	H3K4me3	BF
chr11	5445254	5445255	INS	chr11_5067149_5068286	5.50303911	13.412375	9.1437E-07	0.01164571	H3K4me3	BF
chr9	79075913	79087471	DEL	chr9_79188577_79189065	2.64593267	13.3254615	9.6125E-07	0.01193158	H3K4me3	BF
chr9	79400857	79401079	DEL	chr9_79188577_79189065	2.64593267	13.3254615	9.6125E-07	0.01193158	H3K4me3	BF
chr9	79420256	79420350	DEL	chr9_79188577_79189065	2.64593267	13.3254615	9.6125E-07	0.01193158	H3K4me3	BF
chr9	78739039	78739040	INS	chr9_79188577_79189065	2.64593267	13.3254615	9.6125E-07	0.01193158	H3K4me3	BF
chr9	79649168	79649169	INS	chr9_79188577_79189065	2.64593267	13.3254615	9.6125E-07	0.01193158	H3K4me3	BF
chr9	45458817	45458818	INS	chr9_45642617_45644335	-2.1048763	-13.331002	9.5818E-07	0.01193158	H3K4me3	BF
chr6	132359782	132359783	INS	chr6_132277909_132278733	2.6715466	13.2719031	9.9148E-07	0.01220337	H3K4me3	BF

chr6	132560221	132560222	INS	chr6_132277909_132278733	2.6715466	13.2719031	9.9148E-07	0.01220337	H3K4me3	BF
chr7	8333524	8333525	INS	chr7_8641549_8641919	3.16762436	13.2375077	1.0115E-06	0.01239713	H3K4me3	BF
chr4	105030310	105030626	DEL	chr4_104650335_104651024	3.69209017	13.1795245	1.0462E-06	0.01250476	H3K4me3	BF
chr1	8807134	8808692	DEL	chr1_8809746_8810000	1.64477163	13.159163	1.0587E-06	0.01250476	H3K4me3	BF
chr1	8817246	8817423	DEL	chr1_8809746_8810000	1.64477163	13.159163	1.0587E-06	0.01250476	H3K4me3	BF
chr1	8818772	8818834	DEL	chr1_8809746_8810000	1.64477163	13.159163	1.0587E-06	0.01250476	H3K4me3	BF
chr1	8827722	8828022	DEL	chr1_8809746_8810000	1.64477163	13.159163	1.0587E-06	0.01250476	H3K4me3	BF
chr4	104638086	104638087	INS	chr4_104650335_104651024	3.69209017	13.1795245	1.0462E-06	0.01250476	H3K4me3	BF
chr1	8818417	8818418	INS	chr1_8809746_8810000	1.64477163	13.159163	1.0587E-06	0.01250476	H3K4me3	BF
chr1	9041769	9041770	INS	chr1_8809746_8810000	1.64477163	13.159163	1.0587E-06	0.01250476	H3K4me3	BF
chr7	2070266	2070544	DEL	chr7_2552392_2552938	3.96356222	13.1070636	1.0914E-06	0.012687	H3K4me3	BF
chr7	2085290	2085291	INS	chr7_2552392_2552938	3.96356222	13.1070636	1.0914E-06	0.012687	H3K4me3	BF
chr7	2297683	2297684	INS	chr7_2552392_2552938	3.96356222	13.1070636	1.0914E-06	0.012687	H3K4me3	BF

chr7	2577152	2577153	INS	chr7_2552392_2552938	3.96356222	13.1070636	1.0914E-06	0.012687	H3K4me3	BF
chr10	54179640	54179641	INS	chr10_54056512_54056685	1.556165	13.0658035	1.1182E-06	0.01294652	H3K4me3	BF
chr13	202667554	202667555	INS	chr13_202936774_202937081	2.81004544	13.0388549	1.136E-06	0.01305019	H3K4me3	BF
chr13	202669454	202669455	INS	chr13_202936774_202937081	2.81004544	13.0388549	1.136E-06	0.01305019	H3K4me3	BF
chr2	1729822	1729823	INS	chr2_1483955_1485535	10.7345439	13.0246641	1.1455E-06	0.01310833	H3K4me3	BF
chr3	54993668	54993951	DEL	chr3_55009199_55009935	1.76969525	12.9747967	1.1798E-06	0.01344044	H3K4me3	BF
chr3	124834252	124834490	DEL	chr3_125059361_125060465	1.42408333	12.9495833	1.1975E-06	0.01344044	H3K4me3	BF
chr3	124951564	124951614	DEL	chr3_125059361_125060465	1.42408333	12.9495833	1.1975E-06	0.01344044	H3K4me3	BF
chr3	125103576	125103577	INS	chr3_125059361_125060465	1.42408333	12.9495833	1.1975E-06	0.01344044	H3K4me3	BF
chr3	125330075	125330076	INS	chr3_125059361_125060465	1.42408333	12.9495833	1.1975E-06	0.01344044	H3K4me3	BF
chr15	133027548	133027549	INS	chr15_133446026_133446625	0.91501725	12.9341321	1.2085E-06	0.01351233	H3K4me3	BF
chr8	71578731	71578975	DEL	chr8_71193268_71193530	3.06475104	12.8335989	1.2831E-06	0.01412986	H3K4me3	BF
chr8	70929158	70929159	INS	chr8_71193268_71193530	3.06475104	12.8335989	1.2831E-06	0.01412986	H3K4me3	BF

chr8	71515478	71515479	INS	chr8_71193268_71193530	3.06475104	12.8335989	1.2831E-06	0.01412986	H3K4me3	BF
chr5	20991401	20991402	INS	NW_018084856.1_18342_18595	2.87870514	12.7660706	1.336E-06	0.01460312	H3K4me3	BF
chr5	21229418	21229419	INS	NW_018084856.1_18342_18595	2.87870514	12.7660706	1.336E-06	0.01460312	H3K4me3	BF
chr9	9664853	9664947	DEL	chr9_9664641_9665443	2.493632	12.7343952	1.3617E-06	0.01482842	H3K4me3	BF
chr9	61709845	61709845	BND	chr9_61591457_61592632	5.49566319	12.5861187	1.4894E-06	0.01487954	H3K4me3	BF
chr9	61900187	61900187	BND	chr9_61591457_61592632	5.49566319	12.5861187	1.4894E-06	0.01487954	H3K4me3	BF
chr8	35588068	35588068	BND	chr8_35159645_35160883	2.22549426	12.6063989	1.4712E-06	0.01487954	H3K4me3	BF
chr9	61888077	61888266	DEL	chr9_61591457_61592632	2.7478316	12.5861187	1.4894E-06	0.01487954	H3K4me3	BF
chr10	40078567	40081101	DEL	chr10_40200841_40201353	2.19312667	12.5590033	1.5142E-06	0.01487954	H3K4me3	BF
chr10	40091807	40091876	DEL	chr10_40200841_40201353	2.19312667	12.5590033	1.5142E-06	0.01487954	H3K4me3	BF
chr3	79564678	79564743	DEL	chr3_79773570_79773757	2.76421056	12.5895972	1.4863E-06	0.01487954	H3K4me3	BF
chr1	126597098	126597285	DEL	chr1_126815641_126816513	3.18720806	12.5541072	1.5188E-06	0.01487954	H3K4me3	BF
chr1	126731163	126731339	DEL	chr1_126815641_126816513	3.18720806	12.5541072	1.5188E-06	0.01487954	H3K4me3	BF

chr1	127248406	127248641	DEL	chr1_126815641_126816513	3.18720806	12.5541072	1.5188E-06	0.01487954	H3K4me3	BF
chr11	1105864	1106138	DEL	chr11_1380644_1380917	1.40150083	12.6508462	1.4321E-06	0.01487954	H3K4me3	BF
chr9	61709832	61709833	INS	chr9_61591457_61592632	2.7478316	12.5861187	1.4894E-06	0.01487954	H3K4me3	BF
chr9	61900182	61900183	INS	chr9_61591457_61592632	2.7478316	12.5861187	1.4894E-06	0.01487954	H3K4me3	BF
chr9	61954683	61954684	INS	chr9_61591457_61592632	2.7478316	12.5861187	1.4894E-06	0.01487954	H3K4me3	BF
chr14	99055992	99055993	INS	chr14_99483711_99484100	2.594793	12.5957488	1.4807E-06	0.01487954	H3K4me3	BF
chr14	99438057	99438058	INS	chr14_99483711_99484100	2.594793	12.5957488	1.4807E-06	0.01487954	H3K4me3	BF
chr14	99528180	99528181	INS	chr14_99483711_99484100	2.594793	12.5957488	1.4807E-06	0.01487954	H3K4me3	BF
chr10	49975475	49975476	INS	chr10_50257728_50258105	1.38334194	12.6895045	1.399E-06	0.01487954	H3K4me3	BF
chr10	50363015	50363016	INS	chr10_50257728_50258105	1.38334194	12.6895045	1.399E-06	0.01487954	H3K4me3	BF
chr10	50616433	50616434	INS	chr10_50257728_50258105	1.38334194	12.6895045	1.399E-06	0.01487954	H3K4me3	BF
chr10	50614640	50614641	INS	chr10_50257728_50258105	1.38334194	12.6895045	1.399E-06	0.01487954	H3K4me3	BF
chr10	50683636	50683637	INS	chr10_50257728_50258105	1.38334194	12.6895045	1.399E-06	0.01487954	H3K4me3	BF

chr3	79773741	79773742	INS	chr3_79773570_79773757	2.76421056	12.5895972	1.4863E-06	0.01487954	H3K4me3	BF
chr3	80224606	80224607	INS	chr3_79773570_79773757	2.76421056	12.5895972	1.4863E-06	0.01487954	H3K4me3	BF
chr1	126654721	126654722	INS	chr1_126815641_126816513	3.18720806	12.5541072	1.5188E-06	0.01487954	H3K4me3	BF
chr1	126744736	126744737	INS	chr1_126815641_126816513	3.18720806	12.5541072	1.5188E-06	0.01487954	H3K4me3	BF
chr1	126815645	126815646	INS	chr1_126815641_126816513	3.18720806	12.5541072	1.5188E-06	0.01487954	H3K4me3	BF
chr1	127184733	127184734	INS	chr1_126815641_126816513	3.18720806	12.5541072	1.5188E-06	0.01487954	H3K4me3	BF
chr11	1101079	1101080	INS	chr11_1380644_1380917	1.40150083	12.6508462	1.4321E-06	0.01487954	H3K4me3	BF
chr7	9085629	9085705	DEL	chr7_8641549_8641919	3.15943518	12.5144889	1.5559E-06	0.0151316	H3K4me3	BF
chr3	39974207	39974508	DEL	chr3_39750236_39751718	6.2744	12.4728143	1.5961E-06	0.0151316	H3K4me3	BF
chr3	40212156	40212203	DEL	chr3_39750236_39751718	6.2744	12.4728143	1.5961E-06	0.0151316	H3K4me3	BF
chr5	9130563	9130564	INS	chr5_8996885_8997677	1.16976813	12.5123141	1.558E-06	0.0151316	H3K4me3	BF
chr3	39711704	39711705	INS	chr3_39750236_39751718	6.2744	12.4728143	1.5961E-06	0.0151316	H3K4me3	BF
chr3	40082054	40082055	INS	chr3_39750236_39751718	6.2744	12.4728143	1.5961E-06	0.0151316	H3K4me3	BF

chr3	40061797	40061798	INS	chr3_39750236_39751718	6.2744	12.4728143	1.5961E-06	0.0151316	H3K4me3	BF
chr3	40210026	40210027	INS	chr3_39750236_39751718	6.2744	12.4728143	1.5961E-06	0.0151316	H3K4me3	BF
chr3	39338356	39342676	INV	chr3_39750236_39751718	12.5488	12.4728143	1.5961E-06	0.0151316	H3K4me3	BF
chr3	39353571	39356592	INV	chr3_39750236_39751718	12.5488	12.4728143	1.5961E-06	0.0151316	H3K4me3	BF
chr1	302214	302287	DEL	chr1_738414_739200	1.97378806	12.2737348	1.805E-06	0.01662713	H3K4me3	BF
chr1	657560	657698	DEL	chr1_738414_739200	1.97378806	12.2737348	1.805E-06	0.01662713	H3K4me3	BF
chr1	705974	706043	DEL	chr1_738414_739200	1.97378806	12.2737348	1.805E-06	0.01662713	H3K4me3	BF
chr3	11920833	11920898	DEL	chr3_11943862_11944422	2.25994833	12.2779195	1.8003E-06	0.01662713	H3K4me3	BF
chr1	211950844	211950845	INS	chr1_211832172_211832854	3.96894881	12.2830834	1.7945E-06	0.01662713	H3K4me3	BF
chr3	11637049	11637050	INS	chr3_11943862_11944422	2.25994833	12.2779195	1.8003E-06	0.01662713	H3K4me3	BF
chr3	11921302	11921303	INS	chr3_11943862_11944422	2.25994833	12.2779195	1.8003E-06	0.01662713	H3K4me3	BF
chr2	68261696	68261697	INS	chr2_67843822_67844784	2.36057675	12.2470524	1.8352E-06	0.01685275	H3K4me3	BF
chr16	54407505	54407816	DEL	chr16_54905194_54906025	1.51972994	12.2140267	1.8734E-06	0.01704348	H3K4me3	BF

chr5	11719758	11719759	INS	chr5_11530977_11531363	2.25574338	12.2184042	1.8683E-06	0.01704348	H3K4me3	BF
chr10	19991009	19991010	INS	chr10_20164424_20164892	1.89003163	12.1489275	1.9514E-06	0.01769797	H3K4me3	BF
chr4	93338145	93338146	INS	chr4_93385105_93389803	8.62846188	12.0770685	2.0418E-06	0.01834677	H3K4me3	BF
chr4	93629793	93629794	INS	chr4_93385105_93389803	8.62846188	12.0770685	2.0418E-06	0.01834677	H3K4me3	BF
chr6	38700268	38700269	INS	chr6_38317135_38319173	1.1063539	12.0471408	2.0807E-06	0.01863996	H3K4me3	BF
chr17	26151214	26153068	DEL	chr17_26412284_26412662	2.22282889	11.8991771	2.2862E-06	0.02035583	H3K4me3	BF
chr11	2929403	2929403	BND	chr11_3038687_3039403	2.37519833	11.7217857	2.563E-06	0.02066782	H3K4me3	BF
chr8	3889090	3889288	DEL	chr8_4314265_4314575	2.27052456	11.6893507	2.6176E-06	0.02066782	H3K4me3	BF
chr8	60307686	60309970	DEL	chr8_59862732_59863270	3.17036783	11.8318104	2.3872E-06	0.02066782	H3K4me3	BF
chr8	60355985	60356287	DEL	chr8_59862732_59863270	3.17036783	11.8318104	2.3872E-06	0.02066782	H3K4me3	BF
chr11	2796284	2796586	DEL	chr11_3038687_3039403	2.37519833	11.7217857	2.563E-06	0.02066782	H3K4me3	BF
chr8	4072359	4072647	DEL	chr8_4314265_4314575	2.27052456	11.6893507	2.6176E-06	0.02066782	H3K4me3	BF
chr11	3028105	3028695	DEL	chr11_3038687_3039403	2.37519833	11.7217857	2.563E-06	0.02066782	H3K4me3	BF

chr8	4429295	4429569	DEL	chr8_4314265_4314575	2.27052456	11.6893507	2.6176E-06	0.02066782	H3K4me3	BF
chr11	3465357	3469944	DEL	chr11_3038687_3039403	2.37519833	11.7217857	2.563E-06	0.02066782	H3K4me3	BF
chr17	49851500	49851725	DEL	chr17_50306564_50306722	2.53280493	11.8329909	2.3853E-06	0.02066782	H3K4me3	BF
chr17	50705701	50705802	DEL	chr17_50306564_50306722	2.53280493	11.8329909	2.3853E-06	0.02066782	H3K4me3	BF
chr1	168690699	168691007	DEL	chr1_168501355_168501885	-1.873119	-11.748668	2.5187E-06	0.02066782	H3K4me3	BF
chr1	168685196	168685569	DEL	chr1_168501355_168501885	-1.873119	-11.748668	2.5187E-06	0.02066782	H3K4me3	BF
chr1	168729675	168729948	DEL	chr1_168501355_168501885	-1.873119	-11.748668	2.5187E-06	0.02066782	H3K4me3	BF
chr1	168739741	168741684	DEL	chr1_168501355_168501885	-1.873119	-11.748668	2.5187E-06	0.02066782	H3K4me3	BF
chr11	15394609	15394913	DEL	chr11_15897263_15897697	3.020965	11.7697562	2.4846E-06	0.02066782	H3K4me3	BF
chr11	16253211	16253371	DEL	chr11_15897263_15897697	3.020965	11.7697562	2.4846E-06	0.02066782	H3K4me3	BF
chr11	16300589	16300773	DEL	chr11_15897263_15897697	3.020965	11.7697562	2.4846E-06	0.02066782	H3K4me3	BF
chr11	16351503	16352329	DEL	chr11_15897263_15897697	3.020965	11.7697562	2.4846E-06	0.02066782	H3K4me3	BF
chr6	1560434	1560484	DEL	chr6_1999935_2001148	-6.31626	-11.709327	2.5838E-06	0.02066782	H3K4me3	BF

chr6	1923538	1923911	DEL	chr6_1999935_2001148	-6.31626	-11.709327	2.5838E-06	0.02066782	H3K4me3	BF
chr8	3897800	3897801	INS	chr8_4314265_4314575	2.27052456	11.6893507	2.6176E-06	0.02066782	H3K4me3	BF
chr8	3920185	3920186	INS	chr8_4314265_4314575	2.27052456	11.6893507	2.6176E-06	0.02066782	H3K4me3	BF
chr11	2929402	2929403	INS	chr11_3038687_3039403	2.37519833	11.7217857	2.563E-06	0.02066782	H3K4me3	BF
chr8	4659755	4659756	INS	chr8_4314265_4314575	2.27052456	11.6893507	2.6176E-06	0.02066782	H3K4me3	BF
chr8	4709162	4709163	INS	chr8_4314265_4314575	2.27052456	11.6893507	2.6176E-06	0.02066782	H3K4me3	BF
chr17	50692337	50692338	INS	chr17_50306564_50306722	2.53280493	11.8329909	2.3853E-06	0.02066782	H3K4me3	BF
chr1	168268277	168268278	INS	chr1_168501355_168501885	-1.873119	-11.748668	2.5187E-06	0.02066782	H3K4me3	BF
chr1	168618598	168618599	INS	chr1_168501355_168501885	-1.873119	-11.748668	2.5187E-06	0.02066782	H3K4me3	BF
chr1	168621924	168621925	INS	chr1_168501355_168501885	-1.873119	-11.748668	2.5187E-06	0.02066782	H3K4me3	BF
chr1	168729432	168729433	INS	chr1_168501355_168501885	-1.873119	-11.748668	2.5187E-06	0.02066782	H3K4me3	BF
chr3	6415806	6415807	INS	chr3_6547135_6548717	2.68424343	11.8681432	2.3321E-06	0.02066782	H3K4me3	BF
chr11	15813356	15813357	INS	chr11_15897263_15897697	3.020965	11.7697562	2.4846E-06	0.02066782	H3K4me3	BF

chr11	16041318	16041319	INS	chr11_15897263_15897697	3.020965	11.7697562	2.4846E-06	0.02066782	H3K4me3	BF
chr11	16263489	16263490	INS	chr11_15897263_15897697	3.020965	11.7697562	2.4846E-06	0.02066782	H3K4me3	BF
chr11	16253742	16253743	INS	chr11_15897263_15897697	3.020965	11.7697562	2.4846E-06	0.02066782	H3K4me3	BF
chr11	16299574	16299575	INS	chr11_15897263_15897697	3.020965	11.7697562	2.4846E-06	0.02066782	H3K4me3	BF
chr6	1798228	1798229	INS	chr6_1999935_2001148	-6.31626	-11.709327	2.5838E-06	0.02066782	H3K4me3	BF
chr6	2001179	2001180	INS	chr6_1999935_2001148	-6.31626	-11.709327	2.5838E-06	0.02066782	H3K4me3	BF
chr6	2164640	2164641	INS	chr6_1999935_2001148	-6.31626	-11.709327	2.5838E-06	0.02066782	H3K4me3	BF
chr8	3856003	3856004	INS	chr8_4314265_4314575	2.27052456	11.6893507	2.6176E-06	0.02066782	H3K4me3	BF
chr2	61687728	61688497	DEL	chr2_61744977_61745640	1.71428739	11.660584	2.667E-06	0.0208896	H3K4me3	BF
chr2	61679917	61679918	INS	chr2_61744977_61745640	1.71428739	11.660584	2.667E-06	0.0208896	H3K4me3	BF
chr2	61750603	61750604	INS	chr2_61744977_61745640	1.71428739	11.660584	2.667E-06	0.0208896	H3K4me3	BF
chr15	46100976	46102492	DEL	chr15_45850228_45851604	6.46362889	11.6367326	2.7089E-06	0.02102828	H3K4me3	BF
chr1	9401021	9401022	INS	chr1_9092593_9093104	-0.6707006	-11.634135	2.7135E-06	0.02102828	H3K4me3	BF

chr15	45465782	45465783	INS	chr15_45850228_45851604	6.46362889	11.6367326	2.7089E-06	0.02102828	H3K4me3	BF
chr15	45476744	45476745	INS	chr15_45850228_45851604	6.46362889	11.6367326	2.7089E-06	0.02102828	H3K4me3	BF
chr1	142751907	142759959	DEL	chr1_142499806_142501674	9.42915312	11.5739864	2.8224E-06	0.02181501	H3K4me3	BF
chr16	47085242	47085536	DEL	chr16_47468615_47469862	2.94701561	11.5330991	2.8993E-06	0.02200273	H3K4me3	BF
chr16	47001071	47001072	INS	chr16_47468615_47469862	2.94701561	11.5330991	2.8993E-06	0.02200273	H3K4me3	BF
chr16	46999984	46999985	INS	chr16_47468615_47469862	2.94701561	11.5330991	2.8993E-06	0.02200273	H3K4me3	BF
chr16	47271630	47271631	INS	chr16_47468615_47469862	2.94701561	11.5330991	2.8993E-06	0.02200273	H3K4me3	BF
chr16	47282696	47282697	INS	chr16_47468615_47469862	2.94701561	11.5330991	2.8993E-06	0.02200273	H3K4me3	BF
chr16	47000000	47000179	INV	chr16_47468615_47469862	2.94701561	11.5330991	2.8993E-06	0.02200273	H3K4me3	BF
chr14	111014067	111062178	DUP	chr14_111047936_111048498	3.37094788	11.483563	2.9956E-06	0.0226162	H3K4me3	BF
chr14	111449975	111449976	INS	chr14_111047936_111048498	1.68547394	11.483563	2.9956E-06	0.0226162	H3K4me3	BF
chr14	11004537	11004538	INS	chr14_11351891_11353001	2.50489278	11.474875	3.0128E-06	0.02268798	H3K4me3	BF
chr3	17907851	17907852	INS	chr3_17761064_17762514	-7.3941375	-11.438424	3.0864E-06	0.02318263	H3K4me3	BF

chr4	117762163	117762439	DEL	chr4_117467808_117468948	2.04592417	11.3258642	3.3267E-06	0.02430197	H3K4me3	BF
chr4	117808292	117808580	DEL	chr4_117467808_117468948	2.04592417	11.3258642	3.3267E-06	0.02430197	H3K4me3	BF
chr4	117827588	117827878	DEL	chr4_117467808_117468948	2.04592417	11.3258642	3.3267E-06	0.02430197	H3K4me3	BF
chr4	117242280	117242281	INS	chr4_117467808_117468948	2.04592417	11.3258642	3.3267E-06	0.02430197	H3K4me3	BF
chr4	117308749	117308750	INS	chr4_117467808_117468948	2.04592417	11.3258642	3.3267E-06	0.02430197	H3K4me3	BF
chr4	117686611	117686612	INS	chr4_117467808_117468948	2.04592417	11.3258642	3.3267E-06	0.02430197	H3K4me3	BF
chr4	117688344	117688345	INS	chr4_117467808_117468948	2.04592417	11.3258642	3.3267E-06	0.02430197	H3K4me3	BF
chr4	117892771	117892772	INS	chr4_117467808_117468948	2.04592417	11.3258642	3.3267E-06	0.02430197	H3K4me3	BF
chr4	117947416	117947417	INS	chr4_117467808_117468948	2.04592417	11.3258642	3.3267E-06	0.02430197	H3K4me3	BF
chr3	19844572	19853848	DEL	chr3_19756673_19757034	2.2408535	11.3159076	3.3489E-06	0.02434303	H3K4me3	BF
chr3	19846188	19847874	DEL	chr3_19756673_19757034	2.2408535	11.3159076	3.3489E-06	0.02434303	H3K4me3	BF
chr7	8907373	8907672	DEL	chr7_8818453_8818697	1.79386989	11.2978388	3.3897E-06	0.02451785	H3K4me3	BF
chr7	8908245	8908246	INS	chr7_8818453_8818697	1.79386989	11.2978388	3.3897E-06	0.02451785	H3K4me3	BF

chr6	34292950	34293005	DEL	chr6_34214137_34214514	1.64436667	11.2496792	3.5012E-06	0.02489374	H3K4me3	BF
chr6	34460914	34461140	DEL	chr6_34214137_34214514	1.64436667	11.2496792	3.5012E-06	0.02489374	H3K4me3	BF
chr4	147865	147866	INS	chr4_134466_135081	9.3878	11.2599874	3.477E-06	0.02489374	H3K4me3	BF
NW_018084889.1	7338	7339	INS	chr4_134466_135081	4.6939	11.2599874	3.477E-06	0.02489374	H3K4me3	BF
chr6	1253963	1253964	INS	chr6_1422732_1423667	0.73967952	11.266657	3.4614E-06	0.02489374	H3K4me3	BF
chr6	34115332	34115333	INS	chr6_34214137_34214514	1.64436667	11.2496792	3.5012E-06	0.02489374	H3K4me3	BF
chr6	34388154	34388155	INS	chr6_34214137_34214514	1.64436667	11.2496792	3.5012E-06	0.02489374	H3K4me3	BF
chr6	148411777	148412074	DEL	chr6_148886527_148887486	1.44668977	11.2143496	3.5855E-06	0.02543189	H3K4me3	BF
chr12	26292233	26292234	INS	chr12_26395493_26398376	-7.7630813	-11.209689	3.5968E-06	0.0254504	H3K4me3	BF
chr8	40246268	40246269	INS	chr8_40421673_40421928	2.12799097	11.1890633	3.6473E-06	0.02574542	H3K4me3	BF
chr16	62942507	62942578	DEL	chr16_63086669_63086945	-1.519693	-11.160645	3.7182E-06	0.02574921	H3K4me3	BF
chr16	63168831	63169119	DEL	chr16_63086669_63086945	-1.519693	-11.160645	3.7182E-06	0.02574921	H3K4me3	BF
chr16	62774663	62774664	INS	chr16_63086669_63086945	-1.519693	-11.160645	3.7182E-06	0.02574921	H3K4me3	BF

chr16	62776057	62776058	INS	chr16_63086669_63086945	-1.519693	-11.160645	3.7182E-06	0.02574921	H3K4me3	BF
chr16	63169746	63169747	INS	chr16_63086669_63086945	-1.519693	-11.160645	3.7182E-06	0.02574921	H3K4me3	BF
chr16	63184245	63184246	INS	chr16_63086669_63086945	1.519693	11.1606451	3.7182E-06	0.02574921	H3K4me3	BF
chr16	63182747	63182748	INS	chr16_63086669_63086945	-1.519693	-11.160645	3.7182E-06	0.02574921	H3K4me3	BF
chr16	63296374	63296375	INS	chr16_63086669_63086945	-1.519693	-11.160645	3.7182E-06	0.02574921	H3K4me3	BF
chr2	13122273	13123683	DEL	chr2_13382755_13383813	3.52397129	11.0902712	3.9004E-06	0.02648441	H3K4me3	BF
chr7	29169904	29171283	DEL	chr7_28919210_28921105	8.10742381	11.0885909	3.9048E-06	0.02648441	H3K4me3	BF
chr6	56033943	56034408	DEL	chr6_58714220_58715873	2.91248403	11.0872428	3.9084E-06	0.02648441	H3K4me3	BF
chr17	28730835	28730893	DEL	chr17_28720800_28721055	1.30529328	11.0747507	3.9419E-06	0.02648441	H3K4me3	BF
chr17	11500050	11500051	INS	chr17_11309086_11309485	0.85019514	11.1019182	3.8695E-06	0.02648441	H3K4me3	BF
chr17	28439584	28439585	INS	chr17_28720800_28721055	1.30529328	11.0747507	3.9419E-06	0.02648441	H3K4me3	BF
chr17	28863709	28863710	INS	chr17_28720800_28721055	1.30529328	11.0747507	3.9419E-06	0.02648441	H3K4me3	BF
chr17	29148816	29148817	INS	chr17_28720800_28721055	1.30529328	11.0747507	3.9419E-06	0.02648441	H3K4me3	BF

chr17	29127048	29127049	INS	chr17_28720800_28721055	1.30529328	11.0747507	3.9419E-06	0.02648441	H3K4me3	BF
chr5	6513867	6513868	INS	chr5_6086575_6086848	5.55095063	11.0575396	3.9885E-06	0.02673625	H3K4me3	BF
chr6	117786152	117789097	DEL	chr6_118087465_118088041	2.46637278	11.0202116	4.0917E-06	0.02689776	H3K4me3	BF
chr6	117782261	117782534	DEL	chr6_118087465_118088041	2.46637278	11.0202116	4.0917E-06	0.02689776	H3K4me3	BF
chr6	117783560	117783667	DEL	chr6_118087465_118088041	2.46637278	11.0202116	4.0917E-06	0.02689776	H3K4me3	BF
chr15	25462007	25462315	DEL	chr15_25301129_25301775	1.84183208	11.0059437	4.132E-06	0.02689776	H3K4me3	BF
chr6	117811373	117811374	INS	chr6_118087465_118088041	2.46637278	11.0202116	4.0917E-06	0.02689776	H3K4me3	BF
chr6	118485182	118485183	INS	chr6_118087465_118088041	2.46637278	11.0202116	4.0917E-06	0.02689776	H3K4me3	BF
NW_018085005.1	88084	88085	INS	chr6_29948184_29949739	1.09087438	11.0380904	4.0419E-06	0.02689776	H3K4me3	BF
NW_018085005.1	84771	84772	INS	chr6_29948184_29949739	1.09087438	11.0380904	4.0419E-06	0.02689776	H3K4me3	BF
chr9	133396180	133396253	DEL	chr9_133424623_133425332	1.98299918	10.9093552	4.4163E-06	0.02868495	H3K4me3	BF
chr14	23513062	23513371	DEL	chr14_23139260_23139648	0.97960831	10.9058856	4.4269E-06	0.02869026	H3K4me3	BF
chr4	10945005	10945567	DEL	chr4_10655454_10655679	2.19694339	10.8866101	4.4864E-06	0.02894768	H3K4me3	BF

chr2	10318470	10318470	BND	chr2_10034606_10035116	0.57823745	10.8711343	4.5348E-06	0.02903737	H3K4me3	BF
chr7	59198366	59198662	DEL	chr7_59055727_59056312	0.72340875	10.8478279	4.6088E-06	0.02903737	H3K4me3	BF
chr14	88753970	88754285	DEL	chr14_88559383_88559619	1.23397994	10.8387955	4.6378E-06	0.02903737	H3K4me3	BF
chr12	26953276	26953559	DEL	chr12_27078366_27078545	2.46968611	10.8384142	4.6391E-06	0.02903737	H3K4me3	BF
chr12	27037542	27037834	DEL	chr12_27078366_27078545	2.46968611	10.8384142	4.6391E-06	0.02903737	H3K4me3	BF
chr9	9906114	9906115	INS	chr9_9819091_9819515	1.24186814	10.8439508	4.6212E-06	0.02903737	H3K4me3	BF
chr12	27009586	27009587	INS	chr12_27078366_27078545	2.46968611	10.8384142	4.6391E-06	0.02903737	H3K4me3	BF
chr12	27012031	27012032	INS	chr12_27078366_27078545	2.46968611	10.8384142	4.6391E-06	0.02903737	H3K4me3	BF
chr12	27207300	27207301	INS	chr12_27078366_27078545	2.46968611	10.8384142	4.6391E-06	0.02903737	H3K4me3	BF
chr12	27517498	27517499	INS	chr12_27078366_27078545	2.46968611	10.8384142	4.6391E-06	0.02903737	H3K4me3	BF
chr3	112302343	112302588	DEL	chr3_112434708_112435100	2.10505183	10.8127632	4.7227E-06	0.02949773	H3K4me3	BF
chr2	61825111	61825262	DEL	chr2_61744977_61745640	1.71677494	10.7701542	4.8653E-06	0.02950788	H3K4me3	BF
chr9	16985678	16985955	DEL	chr9_16854003_16854495	2.07439589	10.7997803	4.7656E-06	0.02950788	H3K4me3	BF

chr2	16281871	16282146	DEL	chr2_16239874_16240082	2.14043556	10.77068	4.8635E-06	0.02950788	H3K4me3	BF
chr2	16376309	16376363	DEL	chr2_16239874_16240082	2.14043556	10.77068	4.8635E-06	0.02950788	H3K4me3	BF
chr2	16576696	16576757	DEL	chr2_16239874_16240082	2.14043556	10.77068	4.8635E-06	0.02950788	H3K4me3	BF
chr2	16680046	16680331	DEL	chr2_16239874_16240082	2.14043556	10.77068	4.8635E-06	0.02950788	H3K4me3	BF
chr9	17233315	17233579	DEL	chr9_16854003_16854495	2.07439589	10.7997803	4.7656E-06	0.02950788	H3K4me3	BF
chr2	61852282	61852283	INS	chr2_61744977_61745640	1.71677494	10.7701542	4.8653E-06	0.02950788	H3K4me3	BF
chr9	133125553	133125554	INS	chr9_133425534_133425884	2.16125739	10.7786019	4.8367E-06	0.02950788	H3K4me3	BF
chr9	16713644	16713645	INS	chr9_16854003_16854495	2.07439589	10.7997803	4.7656E-06	0.02950788	H3K4me3	BF
chr2	15833138	15833139	INS	chr2_16239874_16240082	2.14043556	10.77068	4.8635E-06	0.02950788	H3K4me3	BF
chr9	16994089	16994090	INS	chr9_16854003_16854495	2.07439589	10.7997803	4.7656E-06	0.02950788	H3K4me3	BF
chr2	16104627	16104628	INS	chr2_16239874_16240082	2.14043556	10.77068	4.8635E-06	0.02950788	H3K4me3	BF
chr2	16406615	16406616	INS	chr2_16239874_16240082	2.14043556	10.77068	4.8635E-06	0.02950788	H3K4me3	BF
chr1	94493508	94493629	DEL	chr1_94358865_94359228	1.57253172	10.7555658	4.9153E-06	0.02968784	H3K4me3	BF

chr1	94227790	94227791	INS	chr1_94358865_94359228	1.57253172	10.7555658	4.9153E-06	0.02968784	H3K4me3	BF
chr14	112157414	112157415	INS	chr14_111803213_111803557	1.46843111	10.7279538	5.0114E-06	0.02995942	H3K4me3	BF
chr15	38843250	38843251	INS	chr15_38731704_38733279	2.83058021	10.728618	5.009E-06	0.02995942	H3K4me3	BF
chr10	442684	442685	INS	chr10_858031_858211	1.6916729	10.7315945	4.9986E-06	0.02995942	H3K4me3	BF
chr5	77806006	77806007	INS	chr5_77935630_77935792	2.86607521	10.6879749	5.1543E-06	0.03068837	H3K4me3	BF
chr10	442684	442685	INS	chr10_395122_395748	1.38646785	10.6059454	5.4619E-06	0.03238846	H3K4me3	BF
chr2	88716448	88716528	DEL	chr2_88620795_88621372	1.11507708	10.5840335	5.5475E-06	0.03247421	H3K4me3	BF
chr2	88713249	88713539	DEL	chr2_88620795_88621372	1.11507708	10.5840335	5.5475E-06	0.03247421	H3K4me3	BF
chr2	88810819	88810944	DEL	chr2_88620795_88621372	1.11507708	10.5840335	5.5475E-06	0.03247421	H3K4me3	BF
chr2	88807058	88807258	DEL	chr2_88620795_88621372	1.11507708	10.5840335	5.5475E-06	0.03247421	H3K4me3	BF
chr1	6949592	6949593	INS	chr1_7446488_7448419	-6.9903169	-10.579593	5.565E-06	0.03247421	H3K4me3	BF
chr1	129061546	129061547	INS	chr1_129009964_129010198	-6.0142453	-10.560599	5.6407E-06	0.03285048	H3K4me3	BF
chr5	76107599	76107600	INS	chr5_76162049_76162896	1.51042688	10.5575654	5.6529E-06	0.03285619	H3K4me3	BF

chr8	41223208	41783661	DUP	chr8_41401921_41403769	23.6634665	14.2467895	5.7535E-08	0.00078348	H3K4me3	BF
chr5	39976409	39977636	DEL	chr5_40184380_40184682	8.02081644	10.5133633	5.834E-06	0.03377467	H3K4me3	BF
chr5	39989084	39989085	INS	chr5_40184380_40184682	8.02081644	10.5133633	5.834E-06	0.03377467	H3K4me3	BF
chr1	27720430	27720552	DEL	chr1_27625497_27626056	1.246332	10.5036822	5.8745E-06	0.03387535	H3K4me3	BF
chr1	27656614	27656615	INS	chr1_27625497_27626056	1.246332	10.5036822	5.8745E-06	0.03387535	H3K4me3	BF
chr12	8798	8799	INS	chr12_185815_186919	-4.5157014	-10.481983	5.9665E-06	0.03427077	H3K4me3	BF
chr10	35640297	35640298	INS	NW_018085157.1_50036_50219	2.77766069	10.4784488	5.9816E-06	0.03429044	H3K4me3	BF
chr6	83665068	83665200	DEL	chr6_83179299_83179766	0.91938056	10.4425116	6.138E-06	0.03498116	H3K4me3	BF
chr6	82737125	82737126	INS	chr6_83179299_83179766	0.91938056	10.4425116	6.138E-06	0.03498116	H3K4me3	BF
chr6	83135923	83135924	INS	chr6_83179299_83179766	0.91938056	10.4425116	6.138E-06	0.03498116	H3K4me3	BF
chr9	65764218	65764500	DEL	chr9_65501599_65503153	4.70013611	10.372273	6.4568E-06	0.0364013	H3K4me3	BF
chr9	65945345	65945394	DEL	chr9_65501599_65503153	4.70013611	10.372273	6.4568E-06	0.0364013	H3K4me3	BF
chr6	51447108	51447109	INS	chr6_51522207_51524035	4.24145417	10.3685731	6.4741E-06	0.0364013	H3K4me3	BF

chr6	51473377	51473378	INS	chr6_51522207_51524035	4.24145417	10.3685731	6.4741E-06	0.0364013	H3K4me3	BF
chr6	51619227	51619228	INS	chr6_51522207_51524035	4.24145417	10.3685731	6.4741E-06	0.0364013	H3K4me3	BF
chr6	51626418	51626419	INS	chr6_51522207_51524035	4.24145417	10.3685731	6.4741E-06	0.0364013	H3K4me3	BF
chr6	51910594	51910595	INS	chr6_51522207_51524035	4.24145417	10.3685731	6.4741E-06	0.0364013	H3K4me3	BF
chr1	35599420	35599471	DEL	chr1_35751190_35751690	1.17629028	10.3342453	6.6371E-06	0.03717521	H3K4me3	BF
chr1	35789203	35789204	INS	chr1_35751190_35751690	1.17629028	10.3342453	6.6371E-06	0.03717521	H3K4me3	BF
chr7	45849844	45850579	DEL	chr7_46122634_46123082	2.86160021	10.2843813	6.8822E-06	0.03747292	H3K4me3	BF
chr7	46147406	46148107	DEL	chr7_46122634_46123082	2.86160021	10.2843813	6.8822E-06	0.03747292	H3K4me3	BF
chr7	46570269	46570512	DEL	chr7_46122634_46123082	2.86160021	10.2843813	6.8822E-06	0.03747292	H3K4me3	BF
chr7	45865492	45865493	INS	chr7_46122634_46123082	2.86160021	10.2843813	6.8822E-06	0.03747292	H3K4me3	BF
chr7	46169442	46169443	INS	chr7_46122634_46123082	2.86160021	10.2843813	6.8822E-06	0.03747292	H3K4me3	BF
chr7	46557513	46557514	INS	chr7_46122634_46123082	2.86160021	10.2843813	6.8822E-06	0.03747292	H3K4me3	BF
chr7	46569824	46569825	INS	chr7_46122634_46123082	2.86160021	10.2843813	6.8822E-06	0.03747292	H3K4me3	BF

chr12	59555836	59555837	INS	chr12_59578949_59580392	2.05165833	10.2865898	6.8711E-06	0.03747292	H3K4me3	BF
chr12	59690569	59690570	INS	chr12_59578949_59580392	2.05165833	10.2865898	6.8711E-06	0.03747292	H3K4me3	BF
chr12	59889842	59889843	INS	chr12_59578949_59580392	2.05165833	10.2865898	6.8711E-06	0.03747292	H3K4me3	BF
chr12	60052560	60052561	INS	chr12_59578949_59580392	2.05165833	10.2865898	6.8711E-06	0.03747292	H3K4me3	BF
chr5	63589295	63589296	INS	chr5_63600532_63601946	5.82376389	10.2949775	6.8293E-06	0.03747292	H3K4me3	BF
chr13	140513603	140513715	DEL	chr13_140680037_140681682	-6.58203	-10.264071	6.9849E-06	0.03768186	H3K4me3	BF
chr13	140687166	140687486	DEL	chr13_140680037_140681682	-6.58203	-10.264071	6.9849E-06	0.03768186	H3K4me3	BF
chr13	140726091	140726379	DEL	chr13_140680037_140681682	-6.58203	-10.264071	6.9849E-06	0.03768186	H3K4me3	BF
chr13	140553763	140553764	INS	chr13_140680037_140681682	-6.58203	-10.264071	6.9849E-06	0.03768186	H3K4me3	BF
chr5	40186785	40186834	DEL	chr5_40189922_40191046	10.5342284	10.2276979	7.1731E-06	0.03769352	H3K4me3	BF
chr5	40194281	40194330	DEL	chr5_40189922_40191046	10.5342284	10.2276979	7.1731E-06	0.03769352	H3K4me3	BF
chr15	122812232	122812695	DEL	chr15_122382906_122383210	2.04065771	10.2500631	7.0567E-06	0.03769352	H3K4me3	BF
chr10	19755373	19755427	DEL	chr10_20164424_20164892	1.85890033	10.2360478	7.1294E-06	0.03769352	H3K4me3	BF

chr10	20188162	20188225	DEL	chr10_20164424_20164892	1.85890033	10.2360478	7.1294E-06	0.03769352	H3K4me3	BF
chr10	20562979	20563521	DEL	chr10_20164424_20164892	1.85890033	10.2360478	7.1294E-06	0.03769352	H3K4me3	BF
chr5	40181920	40181921	INS	chr5_40189922_40191046	10.5342284	10.2276979	7.1731E-06	0.03769352	H3K4me3	BF
chr15	122486346	122486347	INS	chr15_122382906_122383210	2.04065771	10.2500631	7.0567E-06	0.03769352	H3K4me3	BF
chr14	132376317	132376318	INS	chr14_132531556_132532882	-7.9496333	-10.243709	7.0895E-06	0.03769352	H3K4me3	BF
chr14	132532104	132532105	INS	chr14_132531556_132532882	-7.9496333	-10.243709	7.0895E-06	0.03769352	H3K4me3	BF
chr14	132933909	132933910	INS	chr14_132531556_132532882	-7.9496333	-10.243709	7.0895E-06	0.03769352	H3K4me3	BF
chr10	19831437	19831438	INS	chr10_20164424_20164892	1.85890033	10.2360478	7.1294E-06	0.03769352	H3K4me3	BF
chr10	19923680	19923681	INS	chr10_20164424_20164892	1.85890033	10.2360478	7.1294E-06	0.03769352	H3K4me3	BF
chr8	38855538	38855539	INS	chr8_39253764_39254009	2.19850736	10.2263713	7.18E-06	0.03769352	H3K4me3	BF
chr4	41652060	41652293	DEL	chr4_41915665_41916610	2.18756431	10.1910667	7.3684E-06	0.03833857	H3K4me3	BF
chr8	125877743	125877744	INS	chr8_126232838_126233691	2.56634393	10.194827	7.348E-06	0.03833857	H3K4me3	BF
chr8	126021258	126021259	INS	chr8_126232838_126233691	2.56634393	10.194827	7.348E-06	0.03833857	H3K4me3	BF

chr8	126284960	126284961	INS	chr8_126232838_126233691	2.56634393	10.194827	7.348E-06	0.03833857	H3K4me3	BF
chr4	41799446	41799447	INS	chr4_41915665_41916610	2.18756431	10.1910667	7.3684E-06	0.03833857	H3K4me3	BF
chr3	39359873	39359925	DEL	chr3_39776224_39777862	4.32988947	10.1721437	7.4716E-06	0.0388066	H3K4me3	BF
chr14	20240682	20240742	DEL	chr14_20577928_20578360	1.47955833	10.1536428	7.574E-06	0.03892467	H3K4me3	BF
chr14	20922171	20923458	DEL	chr14_20577928_20578360	1.47955833	10.1536428	7.574E-06	0.03892467	H3K4me3	BF
chr14	20235664	20235665	INS	chr14_20577928_20578360	1.47955833	10.1536428	7.574E-06	0.03892467	H3K4me3	BF
chr14	20810527	20810528	INS	chr14_20577928_20578360	1.47955833	10.1536428	7.574E-06	0.03892467	H3K4me3	BF
chr14	20825521	20825522	INS	chr14_20577928_20578360	1.47955833	10.1536428	7.574E-06	0.03892467	H3K4me3	BF
chr14	20977070	20977071	INS	chr14_20577928_20578360	1.47955833	10.1536428	7.574E-06	0.03892467	H3K4me3	BF
chr1	268975962	268976235	DEL	chr1_269068920_269070874	4.00225625	10.1468119	7.6122E-06	0.03898434	H3K4me3	BF
chr17	43499019	43499020	INS	chr17_43661487_43662189	-0.5600117	-10.147074	7.6108E-06	0.03898434	H3K4me3	BF
chr2	13122273	13123683	DEL	chr2_13384161_13384870	4.80896486	10.1015215	7.8712E-06	0.040017	H3K4me3	BF
chr10	34130108	34130109	INS	chr10_33938474_33939611	1.66841944	10.1070508	7.8391E-06	0.040017	H3K4me3	BF

chr15	14013891	14013892	INS	chr15_14303400_14303699	2.35842742	10.0975613	7.8943E-06	0.040017	H3K4me3	BF
chr9	20218091	20218092	INS	chr9_20482136_20482377	1.62567014	10.0972981	7.8958E-06	0.040017	H3K4me3	BF
chr9	20247169	20247170	INS	chr9_20482136_20482377	1.62567014	10.0972981	7.8958E-06	0.040017	H3K4me3	BF
chr3	76963668	76964067	DEL	chr3_77281354_77282621	1.64618903	10.0897049	7.9404E-06	0.04002698	H3K4me3	BF
chr3	76969386	76969678	DEL	chr3_77281354_77282621	1.64618903	10.0897049	7.9404E-06	0.04002698	H3K4me3	BF
chr3	76987891	76987892	INS	chr3_77281354_77282621	1.64618903	10.0897049	7.9404E-06	0.04002698	H3K4me3	BF
NW_018085246.1	216603	216604	INS	NW_018085246.1_140787_142796	21.5835177	10.0876499	7.9525E-06	0.04002698	H3K4me3	BF
chr6	164185738	164185925	DEL	chr6_164388434_164388985	-1.2344118	-10.060659	8.1133E-06	0.04072935	H3K4me3	BF
chr1	15219617	15219898	DEL	chr1_14958327_14958861	0.535929	10.0595775	8.1198E-06	0.04072935	H3K4me3	BF
chr11	16188456	16188457	INS	chr11_15950267_15951287	0.75150448	10.0166692	8.3833E-06	0.04183589	H3K4me3	BF
chr7	32942705	32942706	INS	chr7_32871879_32872428	1.2695625	10.018213	8.3736E-06	0.04183589	H3K4me3	BF
chr17	26737201	26737496	DEL	chr17_26330351_26330596	1.51338178	9.99731315	8.5052E-06	0.04222867	H3K4me3	BF
chr15	135756484	135756485	INS	chr15_135656347_135656853	1.72010826	10.0006867	8.4838E-06	0.04222867	H3K4me3	BF

chr17	26331780	26331781	INS	chr17_26330351_26330596	1.51338178	9.99731315	8.5052E-06	0.04222867	H3K4me3	BF
chr11	315319	315629	DEL	chr11_351369_353680	3.77527524	9.98638245	8.575E-06	0.04235956	H3K4me3	BF
chr11	324042	324350	DEL	chr11_351369_353680	3.77527524	9.98638245	8.575E-06	0.04235956	H3K4me3	BF
chr11	331968	332277	DEL	chr11_351369_353680	3.77527524	9.98638245	8.575E-06	0.04235956	H3K4me3	BF
chr9	133908469	133908759	DEL	chr9_133424623_133425332	1.95851771	9.96523199	8.7117E-06	0.04267537	H3K4me3	BF
chr9	133600442	133600443	INS	chr9_133424623_133425332	1.95851771	9.96523199	8.7117E-06	0.04267537	H3K4me3	BF
chr9	133785174	133785175	INS	chr9_133424623_133425332	1.95851771	9.96523199	8.7117E-06	0.04267537	H3K4me3	BF
chr9	133871898	133871899	INS	chr9_133424623_133425332	1.95851771	9.96523199	8.7117E-06	0.04267537	H3K4me3	BF
chr9	133906238	133906239	INS	chr9_133424623_133425332	1.95851771	9.96523199	8.7117E-06	0.04267537	H3K4me3	BF
chr1	104269475	104269547	DEL	chr1_104669728_104670410	3.51469444	9.95790111	8.7597E-06	0.04276733	H3K4me3	BF
chr1	104703759	104703760	INS	chr1_104669728_104670410	3.51469444	9.95790111	8.7597E-06	0.04276733	H3K4me3	BF
chr5	16302359	16302520	DEL	chr5_15983427_15984052	6.2496	9.93079048	8.9397E-06	0.04321394	H3K4me3	BF
chr5	16360179	16360340	DEL	chr5_15983427_15984052	6.2496	9.93079048	8.9397E-06	0.04321394	H3K4me3	BF

chr5	16359539	16359540	INS	chr5_15983427_15984052	6.2496	9.93079048	8.9397E-06	0.04321394	H3K4me3	BF
chr5	16423396	16423397	INS	chr5_15983427_15984052	6.2496	9.93079048	8.9397E-06	0.04321394	H3K4me3	BF
chr5	16480999	16481000	INS	chr5_15983427_15984052	6.2496	9.93079048	8.9397E-06	0.04321394	H3K4me3	BF
chr5	16483092	16483093	INS	chr5_15983427_15984052	6.2496	9.93079048	8.9397E-06	0.04321394	H3K4me3	BF
chr6	149220910	149220911	INS	chr6_149398134_149398949	0.51576528	9.91073631	9.0755E-06	0.04358269	H3K4me3	BF
chr6	149241422	149241423	INS	chr6_149398134_149398949	0.51576528	9.91073631	9.0755E-06	0.04358269	H3K4me3	BF
chr6	149814386	149814387	INS	chr6_149398134_149398949	0.51576528	9.91073631	9.0755E-06	0.04358269	H3K4me3	BF
chr6	149813143	149813144	INS	chr6_149398134_149398949	0.51576528	9.91073631	9.0755E-06	0.04358269	H3K4me3	BF
chr14	133835731	133835999	DEL	chr14_133829344_133830922	-3.4083438	-9.8430711	9.551E-06	0.04520533	H3K4me3	BF
chr13	132584898	132585177	DEL	chr13_132486727_132489220	-1.59475	-9.8368768	9.5959E-06	0.04520533	H3K4me3	BF
chr13	132587660	132588197	DEL	chr13_132486727_132489220	-1.59475	-9.8368768	9.5959E-06	0.04520533	H3K4me3	BF
chr9	7344319	7344506	DEL	chr9_7155039_7156325	0.54259563	9.8365033	9.5986E-06	0.04520533	H3K4me3	BF
chr9	7414588	7415520	DEL	chr9_7155039_7156325	0.54259563	9.8365033	9.5986E-06	0.04520533	H3K4me3	BF

chr14	133534685	133534686	INS	chr14_133829344_133830922	-3.4083438	-9.8430711	9.551E-06	0.04520533	H3K4me3	BF
chr14	133576793	133576794	INS	chr14_133829344_133830922	-3.4083438	-9.8430711	9.551E-06	0.04520533	H3K4me3	BF
chr14	133614433	133614434	INS	chr14_133829344_133830922	-3.4083438	-9.8430711	9.551E-06	0.04520533	H3K4me3	BF
chr9	10764404	10764405	INS	chr9_10286036_10286506	1.69815144	9.8590838	9.436E-06	0.04520533	H3K4me3	BF
NW_018084989.1	122610	122611	INS	NW_018084856.1_18342_18595	5.70292656	9.84169293	9.5609E-06	0.04520533	H3K4me3	BF
chr10	50056284	50056285	INS	chr10_50257728_50258105	1.37053444	9.83186268	9.6324E-06	0.04529169	H3K4me3	BF
chr7	2711337	2711338	INS	chr7_2463233_2463777	1.64880162	9.8271206	9.667E-06	0.0453819	H3K4me3	BF
chr9	43009181	43009182	INS	chr9_43379824_43380970	0.95656725	9.80428794	9.836E-06	0.04602739	H3K4me3	BF
chr9	43544462	43544463	INS	chr9_43379824_43380970	0.95656725	9.80428794	9.836E-06	0.04602739	H3K4me3	BF
NW_018085246.1	224079	224366	DEL	NW_018085246.1_140787_142796	-21.096708	-40.901189	1.4056E-10	4.5751E-05	H3K4me3	BF
NW_018085246.1	294074	294137	DEL	NW_018085246.1_140787_142796	-21.096708	-40.901189	1.4056E-10	4.5751E-05	H3K4me3	BF
NW_018085246.1	328807	329087	DEL	NW_018085246.1_140787_142796	-21.096708	-40.901189	1.4056E-10	4.5751E-05	H3K4me3	BF
NW_018085246.1	357496	357556	DEL	NW_018085246.1_140787_142796	-21.096708	-40.901189	1.4056E-10	4.5751E-05	H3K4me3	BF

NW_018085246.1	305762	305763	INS	NW_018085246.1_140787_142796	-21.096708	-40.901189	1.4056E-10	4.5751E-05	H3K4me3	BF
NW_018085246.1	356177	356178	INS	NW_018085246.1_140787_142796	-21.096708	-40.901189	1.4056E-10	4.5751E-05	H3K4me3	BF
NW_018085246.1	351928	351929	INS	NW_018085246.1_140787_142796	-21.096708	-40.901189	1.4056E-10	4.5751E-05	H3K4me3	BF
chr2	8433866	8433934	DEL	chr2_8448639_8449604	7.68754889	23.546073	1.1258E-08	0.00655339	CTCF	LD
chr2	8923036	8923292	DEL	chr2_8448639_8449604	7.68754889	23.546073	1.1258E-08	0.00655339	CTCF	LD
chr2	8959315	8959613	DEL	chr2_8448639_8449604	7.68754889	23.546073	1.1258E-08	0.00655339	CTCF	LD
chr2	8846105	8846106	INS	chr2_8448639_8449604	7.68754889	23.546073	1.1258E-08	0.00655339	CTCF	LD
chr16	21515209	21515321	DEL	chr16_21220712_21220959	3.639169	21.9430796	1.9637E-08	0.00935229	CTCF	LD
chr16	21546365	21546534	DEL	chr16_21220712_21220959	3.639169	21.9430796	1.9637E-08	0.00935229	CTCF	LD
chr18	6455560	6455560	BND	chr18_6260090_6260985	13.1341778	18.6491658	7.0531E-08	0.0094605	CTCF	LD
chr13	22539950	22540231	DEL	chr13_22846117_22847436	17.281355	20.0994973	3.9181E-08	0.0094605	CTCF	LD
chr13	22542122	22542373	DEL	chr13_22846117_22847436	17.281355	20.0994973	3.9181E-08	0.0094605	CTCF	LD
chr13	22543169	22543600	DEL	chr13_22846117_22847436	17.281355	20.0994973	3.9181E-08	0.0094605	CTCF	LD

chr16	71239536	71239703	DEL	chr16_71719519_71719753	3.67453367	19.065143	5.9327E-08	0.0094605	CTCF	LD
chr16	72129299	72129653	DEL	chr16_71719519_71719753	3.67453367	19.065143	5.9327E-08	0.0094605	CTCF	LD
chr14	98404751	98404813	DEL	chr14_97944483_97944755	3.45651578	18.4771207	7.5844E-08	0.0094605	CTCF	LD
chr1	154126426	154126717	DEL	chr1_153806238_153806501	3.57933444	18.830279	6.5385E-08	0.0094605	CTCF	LD
chr9	8588494	8589789	DEL	chr9_8437835_8438586	4.65070144	18.514229	7.4661E-08	0.0094605	CTCF	LD
chr9	8905074	8905473	DEL	chr9_8437835_8438586	4.65070144	18.514229	7.4661E-08	0.0094605	CTCF	LD
chr18	5859059	5861902	DEL	chr18_6260090_6260985	6.56708889	18.6491658	7.0531E-08	0.0094605	CTCF	LD
chr16	54865063	54865121	DEL	chr16_55302278_55302532	5.30934233	19.1803698	5.6588E-08	0.0094605	CTCF	LD
chr16	55316767	55316890	DEL	chr16_55302278_55302532	5.30934233	19.1803698	5.6588E-08	0.0094605	CTCF	LD
chr16	55731010	55731086	DEL	chr16_55302278_55302532	5.30934233	19.1803698	5.6588E-08	0.0094605	CTCF	LD
chr16	71684815	71684816	INS	chr16_71719519_71719753	3.67453367	19.065143	5.9327E-08	0.0094605	CTCF	LD
chr16	71966713	71966714	INS	chr16_71719519_71719753	3.67453367	19.065143	5.9327E-08	0.0094605	CTCF	LD
chr14	97795546	97795547	INS	chr14_97944483_97944755	3.45651578	18.4771207	7.5844E-08	0.0094605	CTCF	LD

chr14	98113612	98113613	INS	chr14_97944483_97944755	3.45651578	18.4771207	7.5844E-08	0.0094605	CTCF	LD
chr9	8092921	8092922	INS	chr9_8437835_8438586	4.65070144	18.514229	7.4661E-08	0.0094605	CTCF	LD
chr9	8666806	8666807	INS	chr9_8437835_8438586	4.65070144	18.514229	7.4661E-08	0.0094605	CTCF	LD
chr9	8866106	8866107	INS	chr9_8437835_8438586	4.65070144	18.514229	7.4661E-08	0.0094605	CTCF	LD
chr9	8865150	8865151	INS	chr9_8437835_8438586	4.65070144	18.514229	7.4661E-08	0.0094605	CTCF	LD
chr18	5823385	5823386	INS	chr18_6260090_6260985	6.56708889	18.6491658	7.0531E-08	0.0094605	CTCF	LD
chr18	6287987	6287988	INS	chr18_6260090_6260985	6.56708889	18.6491658	7.0531E-08	0.0094605	CTCF	LD
chr18	6455560	6455561	INS	chr18_6260090_6260985	6.56708889	18.6491658	7.0531E-08	0.0094605	CTCF	LD
chr2	1845529	1845530	INS	chr2_1754293_1754831	2.68490056	21.0131216	2.762E-08	0.0094605	CTCF	LD
chr16	55244188	55244189	INS	chr16_55302278_55302532	5.30934233	19.1803698	5.6588E-08	0.0094605	CTCF	LD
chr16	55315380	55315381	INS	chr16_55302278_55302532	5.30934233	19.1803698	5.6588E-08	0.0094605	CTCF	LD
chr16	55481723	55481724	INS	chr16_55302278_55302532	5.30934233	19.1803698	5.6588E-08	0.0094605	CTCF	LD
chr6	1283794	1283869	DEL	chr6_1261738_1262638	-3.0980917	-17.879957	9.8092E-08	0.01141992	CTCF	LD

chr5	39976409	39977636	DEL	chr5_40183426_40184692	6.51337711	15.9538766	2.3872E-07	0.0182069	CTCF	LD
chr9	67446707	67446810	DEL	chr9_67561706_67561964	5.66245656	16.3447969	1.9771E-07	0.0182069	CTCF	LD
chr10	50867019	50867161	DEL	chr10_51120622_51121655	2.77309333	16.0579817	2.2694E-07	0.0182069	CTCF	LD
chr10	50873614	50873789	DEL	chr10_51120622_51121655	2.77309333	16.0579817	2.2694E-07	0.0182069	CTCF	LD
chr18	294693	294879	DEL	chr18_392540_392813	5.20260861	16.5650302	1.7813E-07	0.0182069	CTCF	LD
chr18	728314	728721	DEL	chr18_392540_392813	5.20260861	16.5650302	1.7813E-07	0.0182069	CTCF	LD
chr18	12364209	12364373	DEL	chr18_12137285_12137728	3.42060667	16.2623586	2.0566E-07	0.0182069	CTCF	LD
chr18	22963980	22964034	DEL	chr18_23123158_23123401	2.56919519	16.0565934	2.2709E-07	0.0182069	CTCF	LD
chr4	40934082	40934450	DEL	chr4_40603175_40603670	2.73640589	16.1853728	2.134E-07	0.0182069	CTCF	LD
chr5	39989084	39989085	INS	chr5_40183426_40184692	6.51337711	15.9538766	2.3872E-07	0.0182069	CTCF	LD
chr9	67181333	67181334	INS	chr9_67561706_67561964	5.66245656	16.3447969	1.9771E-07	0.0182069	CTCF	LD
chr9	67224531	67224532	INS	chr9_67561706_67561964	5.66245656	16.3447969	1.9771E-07	0.0182069	CTCF	LD
chr10	50683636	50683637	INS	chr10_51120622_51121655	2.77309333	16.0579817	2.2694E-07	0.0182069	CTCF	LD

chr10	50951655	50951656	INS	chr10_51120622_51121655	2.77309333	16.0579817	2.2694E-07	0.0182069	CTCF	LD
chr10	51021953	51021954	INS	chr10_51120622_51121655	2.77309333	16.0579817	2.2694E-07	0.0182069	CTCF	LD
chr10	51413283	51413284	INS	chr10_51120622_51121655	2.77309333	16.0579817	2.2694E-07	0.0182069	CTCF	LD
chr8	5924680	5924681	INS	chr8_5750584_5750863	5.8286145	16.0845808	2.2403E-07	0.0182069	CTCF	LD
chr18	574490	574491	INS	chr18_392540_392813	5.20260861	16.5650302	1.7813E-07	0.0182069	CTCF	LD
chr18	861327	861328	INS	chr18_392540_392813	5.20260861	16.5650302	1.7813E-07	0.0182069	CTCF	LD
chr4	40193840	40193841	INS	chr4_40603175_40603670	2.73640589	16.1853728	2.134E-07	0.0182069	CTCF	LD
chr4	40176880	40176881	INS	chr4_40603175_40603670	2.73640589	16.1853728	2.134E-07	0.0182069	CTCF	LD
chr8	24187889	24188150	DEL	chr8_24104160_24104399	1.77133481	15.3733863	3.1842E-07	0.02349539	CTCF	LD
NW_018085136.1	167830	167831	INS	NW_018085136.1_169864_170157	3.28081231	15.259029	3.3742E-07	0.02455157	CTCF	LD
chr13	1632642	1633456	DEL	chr13_1234624_1234877	4.05338294	15.1124012	3.6367E-07	0.02609897	CTCF	LD
chr1	144722048	144722229	DEL	chr1_145207244_145207539	3.82064826	14.7120355	4.4779E-07	0.03165717	CTCF	LD
chr13	204670254	204670309	DEL	chr13_204605864_204606234	3.02684288	14.6892169	4.532E-07	0.03165717	CTCF	LD

chr2	8301951	8301952	INS	chr2_8448639_8449604	7.64979375	14.6502582	4.6262E-07	0.03188959	CTCF	LD
chr13	17842761	17844390	DEL	chr13_17862001_17862437	3.36604806	14.1240637	6.1383E-07	0.03278841	CTCF	LD
chr13	18144382	18144499	DEL	chr13_17862001_17862437	3.36604806	14.1240637	6.1383E-07	0.03278841	CTCF	LD
chr9	65004411	65004744	DEL	chr9_65240430_65240701	4.26064022	14.2669867	5.679E-07	0.03278841	CTCF	LD
chr1	119912026	119912167	DEL	chr1_120258784_120259015	3.48826456	14.4114076	5.2536E-07	0.03278841	CTCF	LD
chr1	119962358	119962442	DEL	chr1_120258784_120259015	3.48826456	14.4114076	5.2536E-07	0.03278841	CTCF	LD
chr6	56033943	56034408	DEL	chr6_58714933_58715383	6.5813616	14.3072161	5.5567E-07	0.03278841	CTCF	LD
chr15	54856720	54856826	DEL	chr15_55327482_55328558	5.19348667	14.4979823	5.0158E-07	0.03278841	CTCF	LD
chr15	54870552	54871365	DEL	chr15_55327482_55328558	5.19348667	14.4979823	5.0158E-07	0.03278841	CTCF	LD
chr13	17546713	17546714	INS	chr13_17862001_17862437	3.36604806	14.1240637	6.1383E-07	0.03278841	CTCF	LD
chr13	17562813	17562814	INS	chr13_17862001_17862437	3.36604806	14.1240637	6.1383E-07	0.03278841	CTCF	LD
chr13	17851219	17851220	INS	chr13_17862001_17862437	3.36604806	14.1240637	6.1383E-07	0.03278841	CTCF	LD
chr13	18051750	18051751	INS	chr13_17862001_17862437	3.36604806	14.1240637	6.1383E-07	0.03278841	CTCF	LD

chr13	18113494	18113495	INS	chr13_17862001_17862437	3.36604806	14.1240637	6.1383E-07	0.03278841	CTCF	LD
chr13	18143759	18143760	INS	chr13_17862001_17862437	3.36604806	14.1240637	6.1383E-07	0.03278841	CTCF	LD
chr9	64995317	64995318	INS	chr9_65240430_65240701	4.26064022	14.2669867	5.679E-07	0.03278841	CTCF	LD
chr9	65535826	65535827	INS	chr9_65240430_65240701	4.26064022	14.2669867	5.679E-07	0.03278841	CTCF	LD
chr11	75424096	75424097	INS	chr11_74934249_74934717	1.68532919	14.2031333	5.8792E-07	0.03278841	CTCF	LD
chr8	125760372	125760373	INS	chr8_126024886_126025150	4.27637083	14.07044	6.3212E-07	0.03278841	CTCF	LD
chr8	126509788	126509789	INS	chr8_126024886_126025150	4.27637083	14.07044	6.3212E-07	0.03278841	CTCF	LD
chr1	119912736	119912737	INS	chr1_120258784_120259015	3.48826456	14.4114076	5.2536E-07	0.03278841	CTCF	LD
chr6	153170681	153170682	INS	chr6_152676298_152676561	3.37889161	14.190678	5.9192E-07	0.03278841	CTCF	LD
chr12	19713772	19713773	INS	chr12_19713354_19714899	-5.1573739	-14.106083	6.199E-07	0.03278841	CTCF	LD
chr3	36732246	36732247	INS	chr3_36730504_36730924	4.194419	14.1191362	6.1548E-07	0.03278841	CTCF	LD
chr15	54855217	54855218	INS	chr15_55327482_55328558	5.19348667	14.4979823	5.0158E-07	0.03278841	CTCF	LD
chr3	122340330	122341368	DEL	chr3_122300897_122301195	4.29078452	14.0240465	6.4845E-07	0.03330532	CTCF	LD

chr15	66391402	66391403	INS	chr15_66821318_66821636	2.47207813	13.9638517	6.7033E-07	0.03348094	CTCF	LD
chr14	107798048	107798049	INS	chr14_107642847_107643241	2.24859039	13.9619495	6.7104E-07	0.03348094	CTCF	LD
chr14	107818358	107818359	INS	chr14_107642847_107643241	2.24859039	13.9619495	6.7104E-07	0.03348094	CTCF	LD
chr11	70716201	70716292	DEL	chr11_70799933_70800167	3.36028261	13.8146766	7.2823E-07	0.03388364	CTCF	LD
chr9	126502338	126503142	DEL	chr9_126644042_126644538	3.498775	13.6352602	8.0542E-07	0.03388364	CTCF	LD
chr1	242737904	242738840	DEL	chr1_243198717_243199664	1.43321389	13.5593441	8.408E-07	0.03388364	CTCF	LD
chr1	243236545	243239086	DEL	chr1_243198717_243199664	1.43321389	13.5593441	8.408E-07	0.03388364	CTCF	LD
chr5	10762104	10762578	DEL	chr5_10520539_10520940	1.33599294	13.9014542	6.939E-07	0.03388364	CTCF	LD
chr7	43047159	43047160	INS	chr7_43470449_43470700	4.06094111	13.5768217	8.325E-07	0.03388364	CTCF	LD
chr7	43144232	43144233	INS	chr7_43470449_43470700	4.06094111	13.5768217	8.325E-07	0.03388364	CTCF	LD
chr3	58398188	58398189	INS	chr3_58476439_58478348	3.74659688	13.8022617	7.3329E-07	0.03388364	CTCF	LD
chr3	58568874	58568875	INS	chr3_58476439_58478348	3.74659688	13.8022617	7.3329E-07	0.03388364	CTCF	LD
chr13	34385671	34385672	INS	chr13_34370632_34370972	-4.5323433	-13.567559	8.3689E-07	0.03388364	CTCF	LD

chr11	70717636	70717637	INS	chr11_70799933_70800167	3.36028261	13.8146766	7.2823E-07	0.03388364	CTCF	LD
chr3	118236305	118236306	INS	chr3_118661999_118662294	4.11155194	13.7231642	7.6651E-07	0.03388364	CTCF	LD
chr3	119159860	119159861	INS	chr3_118661999_118662294	4.11155194	13.7231642	7.6651E-07	0.03388364	CTCF	LD
chr1	153350677	153350678	INS	chr1_153806238_153806501	3.56667625	13.6867705	7.8236E-07	0.03388364	CTCF	LD
chr1	202360136	202360137	INS	chr1_202800902_202801163	4.21336514	13.8299173	7.2206E-07	0.03388364	CTCF	LD
chr1	242866604	242866605	INS	chr1_243198717_243199664	1.43321389	13.5593441	8.408E-07	0.03388364	CTCF	LD
chr1	243249032	243249033	INS	chr1_243198717_243199664	1.43321389	13.5593441	8.408E-07	0.03388364	CTCF	LD
chr8	13147905	13147906	INS	chr8_13243100_13243413	3.75312656	13.5797263	8.3113E-07	0.03388364	CTCF	LD
chr8	13376387	13376388	INS	chr8_13243100_13243413	3.75312656	13.5797263	8.3113E-07	0.03388364	CTCF	LD
NW_018084925.1	18517	18518	INS	NW_018085302.1_30150_30498	4.44454638	13.5877601	8.2736E-07	0.03388364	CTCF	LD
NW_018084884.1	61346	61347	INS	NW_018084884.1_78705_79458	29.6907025	13.6995807	7.7674E-07	0.03388364	CTCF	LD
NW_018084804.1	10283	10284	INS	NW_018085270.1_76729_77113	9.83295422	13.5339007	8.5305E-07	0.03411475	CTCF	LD
chr5	31176745	31176838	DEL	chr5_31256539_31256811	2.02907794	13.4926678	8.7332E-07	0.03424136	CTCF	LD

chr10	51341558	51341559	INS	chr10_51120622_51121655	2.7730525	13.4926956	8.7331E-07	0.03424136	CTCF	LD
chr6	14361557	14361558	INS	chr6_14414188_14414571	2.05552344	13.4746327	8.8236E-07	0.03424136	CTCF	LD
chr6	57400733	57400881	DEL	chr6_57626469_57626747	4.29243868	13.4446103	8.9763E-07	0.03432564	CTCF	LD
chr6	57394067	57396486	DEL	chr6_57626469_57626747	4.29243868	13.4446103	8.9763E-07	0.03432564	CTCF	LD
chr10	66704707	66704708	INS	chr10_67136077_67136378	4.30622244	13.4063317	9.1754E-07	0.0348327	CTCF	LD
chr5	71998093	71998165	DEL	chr5_72470963_72471381	2.85057167	13.276949	9.8859E-07	0.03547329	CTCF	LD
chr1	157303656	157304850	DEL	chr1_157753474_157753805	2.66591372	13.3129845	9.682E-07	0.03547329	CTCF	LD
chr5	72235550	72235551	INS	chr5_72470963_72471381	2.85057167	13.276949	9.8859E-07	0.03547329	CTCF	LD
chr1	157291153	157291154	INS	chr1_157753474_157753805	2.66591372	13.3129845	9.682E-07	0.03547329	CTCF	LD
chr1	158089942	158089943	INS	chr1_157753474_157753805	2.66591372	13.3129845	9.682E-07	0.03547329	CTCF	LD
chr1	158271940	158271941	INS	chr1_157753474_157753805	2.66591372	13.3129845	9.682E-07	0.03547329	CTCF	LD
chr2	39953038	39953039	INS	chr2_39570675_39571304	6.32897278	13.2797233	9.87E-07	0.03547329	CTCF	LD
chr7	39100485	39100486	INS	chr7_38794769_38795006	2.76284333	13.2386611	1.0108E-06	0.03602279	CTCF	LD

chr9	11304012	11304013	INS	chr9_11214280_11214935	6.06746875	13.1541116	1.0618E-06	0.03758484	CTCF	LD
chr4	89638135	89638135	BND	chr4_89633598_89633868	7.66830833	12.9438434	1.2016E-06	0.03787051	CTCF	LD
chr4	71324234	71324356	DEL	chr4_71414092_71414469	2.6457095	12.8625937	1.261E-06	0.03787051	CTCF	LD
chr4	71327213	71327715	DEL	chr4_71414092_71414469	2.6457095	12.8625937	1.261E-06	0.03787051	CTCF	LD
chr4	4173223	4173296	DEL	chr4_4342092_4342527	2.19034988	12.6822511	1.4052E-06	0.03787051	CTCF	LD
chr15	101897244	101897369	DEL	chr15_102387879_102388272	5.45002711	12.9631359	1.1879E-06	0.03787051	CTCF	LD
chr15	121967787	121968344	DEL	chr15_122305497_122305744	3.46731494	12.7902281	1.3168E-06	0.03787051	CTCF	LD
chr10	66356476	66356538	DEL	chr10_66829225_66829484	2.77517639	12.9317401	1.2102E-06	0.03787051	CTCF	LD
chr1	121241297	121241455	DEL	chr1_120821716_120821982	4.66510792	12.8820615	1.2465E-06	0.03787051	CTCF	LD
chr5	100073548	100073855	DEL	chr5_100357411_100357792	2.66277117	12.6434484	1.4385E-06	0.03787051	CTCF	LD
chr6	152842565	152842887	DEL	chr6_152676298_152676561	3.38331244	12.6523884	1.4308E-06	0.03787051	CTCF	LD
chr1	212249098	212249267	DEL	chr1_212482680_212483503	1.84822594	13.0724391	1.1138E-06	0.03787051	CTCF	LD
chr14	9465618	9467565	DEL	chr14_8996093_8996379	3.76470924	12.997207	1.1643E-06	0.03787051	CTCF	LD

chr14	11134315	11134397	DEL	chr14_11026304_11026576	3.00576167	12.9267675	1.2138E-06	0.03787051	CTCF	LD
chr14	11125158	11126042	DEL	chr14_11026304_11026576	3.00576167	12.9267675	1.2138E-06	0.03787051	CTCF	LD
chr14	11242835	11243130	DEL	chr14_11026304_11026576	3.00576167	12.9267675	1.2138E-06	0.03787051	CTCF	LD
chr17	13692495	13692775	DEL	chr17_14040829_14041111	2.88162283	12.6590088	1.425E-06	0.03787051	CTCF	LD
chr17	13675972	13676286	DEL	chr17_14040829_14041111	2.88162283	12.6590088	1.425E-06	0.03787051	CTCF	LD
chr17	13812403	13812678	DEL	chr17_14040829_14041111	2.88162283	12.6590088	1.425E-06	0.03787051	CTCF	LD
chr9	46124508	46124784	DEL	chr9_46235793_46236616	9.18640556	12.8389819	1.2789E-06	0.03787051	CTCF	LD
chr9	46425783	46426818	DEL	chr9_46235793_46236616	9.18640556	12.8389819	1.2789E-06	0.03787051	CTCF	LD
chr15	101933234	101933235	INS	chr15_102387879_102388272	5.45002711	12.9631359	1.1879E-06	0.03787051	CTCF	LD
chr15	102246166	102246167	INS	chr15_102387879_102388272	5.45002711	12.9631359	1.1879E-06	0.03787051	CTCF	LD
chr9	78612058	78612059	INS	chr9_79109231_79109496	2.80895153	13.069107	1.116E-06	0.03787051	CTCF	LD
chr9	79328433	79328434	INS	chr9_79109231_79109496	2.80895153	13.069107	1.116E-06	0.03787051	CTCF	LD
chr15	122507157	122507158	INS	chr15_122305497_122305744	3.46731494	12.7902281	1.3168E-06	0.03787051	CTCF	LD

chr15	122780520	122780521	INS	chr15_122305497_122305744	3.46731494	12.7902281	1.3168E-06	0.03787051	CTCF	LD
chr15	122775984	122775985	INS	chr15_122305497_122305744	3.46731494	12.7902281	1.3168E-06	0.03787051	CTCF	LD
chr10	66704707	66704708	INS	chr10_66829225_66829484	2.77517639	12.9317401	1.2102E-06	0.03787051	CTCF	LD
chr5	99977601	99977602	INS	chr5_100357411_100357792	2.66277117	12.6434484	1.4385E-06	0.03787051	CTCF	LD
chr5	99976240	99976241	INS	chr5_100357411_100357792	2.66277117	12.6434484	1.4385E-06	0.03787051	CTCF	LD
chr5	100072687	100072688	INS	chr5_100357411_100357792	2.66277117	12.6434484	1.4385E-06	0.03787051	CTCF	LD
chr5	100054588	100054589	INS	chr5_100357411_100357792	2.66277117	12.6434484	1.4385E-06	0.03787051	CTCF	LD
chr5	100160811	100160812	INS	chr5_100357411_100357792	2.66277117	12.6434484	1.4385E-06	0.03787051	CTCF	LD
chr5	100543669	100543670	INS	chr5_100357411_100357792	2.66277117	12.6434484	1.4385E-06	0.03787051	CTCF	LD
chr5	6513867	6513868	INS	chr5_6127280_6128623	5.07676875	13.1219809	1.0819E-06	0.03787051	CTCF	LD
chr10	11698833	11698834	INS	chr10_11945253_11945601	3.34710411	12.9554985	1.1933E-06	0.03787051	CTCF	LD
chr10	11979215	11979216	INS	chr10_11945253_11945601	3.34710411	12.9554985	1.1933E-06	0.03787051	CTCF	LD
chr10	12030922	12030923	INS	chr10_11945253_11945601	3.34710411	12.9554985	1.1933E-06	0.03787051	CTCF	LD

chr10	12138054	12138055	INS	chr10_11945253_11945601	3.34710411	12.9554985	1.1933E-06	0.03787051	CTCF	LD
chr14	11244352	11244353	INS	chr14_11026304_11026576	3.00576167	12.9267675	1.2138E-06	0.03787051	CTCF	LD
chr17	13677448	13677449	INS	chr17_14040829_14041111	2.88162283	12.6590088	1.425E-06	0.03787051	CTCF	LD
chr17	13673625	13673626	INS	chr17_14040829_14041111	2.88162283	12.6590088	1.425E-06	0.03787051	CTCF	LD
chr17	13680492	13680493	INS	chr17_14040829_14041111	2.88162283	12.6590088	1.425E-06	0.03787051	CTCF	LD
chr17	13681354	13681355	INS	chr17_14040829_14041111	2.88162283	12.6590088	1.425E-06	0.03787051	CTCF	LD
chr17	14166713	14166714	INS	chr17_14040829_14041111	2.88162283	12.6590088	1.425E-06	0.03787051	CTCF	LD
chr17	14236471	14236472	INS	chr17_14040829_14041111	2.88162283	12.6590088	1.425E-06	0.03787051	CTCF	LD
chr9	38551880	38551881	INS	chr9_38547315_38547603	3.81566438	12.86035	1.2627E-06	0.03787051	CTCF	LD
chr9	45871117	45871118	INS	chr9_46235793_46236616	9.18640556	12.8389819	1.2789E-06	0.03787051	CTCF	LD
chr9	45882749	45882750	INS	chr9_46235793_46236616	9.18640556	12.8389819	1.2789E-06	0.03787051	CTCF	LD
NW_018085293.1	591799	591867	DEL	NW_018085293.1_623988_624240	6.76759206	12.6227131	1.4567E-06	0.03796782	CTCF	LD
chr2	112903250	112903986	DEL	chr2_112960372_112960604	3.95761711	12.5296405	1.5416E-06	0.03887349	CTCF	LD

chr2	112661189	112661190	INS	chr2_112960372_112960604	3.95761711	12.5296405	1.5416E-06	0.03887349	CTCF	LD
chr2	113011958	113011959	INS	chr2_112960372_112960604	3.95761711	12.5296405	1.5416E-06	0.03887349	CTCF	LD
chr2	113173367	113173368	INS	chr2_112960372_112960604	3.95761711	12.5296405	1.5416E-06	0.03887349	CTCF	LD
chr2	113136658	113136659	INS	chr2_112960372_112960604	3.95761711	12.5296405	1.5416E-06	0.03887349	CTCF	LD
chr1	258230645	258230646	INS	chr1_258568389_258568744	2.64391956	12.546917	1.5254E-06	0.03887349	CTCF	LD
chr7	16574529	16574530	INS	chr7_16468812_16469278	1.00175167	12.527727	1.5434E-06	0.03887349	CTCF	LD
chr10	26541639	26541779	DEL	chr10_26294333_26294612	2.83944688	12.3849997	1.6847E-06	0.039665	CTCF	LD
chr11	47340496	47341795	DEL	chr11_46953682_46954073	2.56570722	12.4100245	1.6589E-06	0.039665	CTCF	LD
chr8	77121665	77121731	DEL	chr8_76847465_76847743	4.76978272	12.3604809	1.7104E-06	0.039665	CTCF	LD
chr8	77251725	77251862	DEL	chr8_76847465_76847743	4.76978272	12.3604809	1.7104E-06	0.039665	CTCF	LD
chr14	84061806	84061980	DEL	chr14_84163439_84164478	1.96514861	12.3773896	1.6927E-06	0.039665	CTCF	LD
chr9	45198258	45198313	DEL	chr9_45107574_45107895	3.50124856	12.384523	1.6852E-06	0.039665	CTCF	LD
chr10	26537391	26537392	INS	chr10_26294333_26294612	2.83944688	12.3849997	1.6847E-06	0.039665	CTCF	LD

chr10	26532663	26532664	INS	chr10_26294333_26294612	2.83944688	12.3849997	1.6847E-06	0.039665	CTCF	LD
chr10	26730983	26730984	INS	chr10_26294333_26294612	2.83944688	12.3849997	1.6847E-06	0.039665	CTCF	LD
chr16	71402924	71402925	INS	chr16_71719519_71719753	3.6450335	12.4512243	1.6174E-06	0.039665	CTCF	LD
chr5	77947535	77947536	INS	chr5_78147610_78148916	5.03644375	12.4518126	1.6168E-06	0.039665	CTCF	LD
chr1	136502899	136502900	INS	chr1_136685122_136685498	2.23188028	12.3598474	1.7111E-06	0.039665	CTCF	LD
chr9	45349713	45349714	INS	chr9_45107574_45107895	3.50124856	12.384523	1.6852E-06	0.039665	CTCF	LD
chr9	45344547	45344548	INS	chr9_45107574_45107895	3.50124856	12.384523	1.6852E-06	0.039665	CTCF	LD
chr9	45553704	45553705	INS	chr9_45107574_45107895	3.50124856	12.384523	1.6852E-06	0.039665	CTCF	LD
chr7	12207884	12208262	DEL	chr7_12098528_12099667	1.98539261	12.3159555	1.7582E-06	0.04004904	CTCF	LD
chr7	12174888	12175052	DEL	chr7_12098528_12099667	1.98539261	12.3159555	1.7582E-06	0.04004904	CTCF	LD
chr7	12216967	12217132	DEL	chr7_12098528_12099667	1.98539261	12.3159555	1.7582E-06	0.04004904	CTCF	LD
chr7	12223683	12223736	DEL	chr7_12098528_12099667	1.98539261	12.3159555	1.7582E-06	0.04004904	CTCF	LD
chr17	12493145	12493928	DEL	chr17_12392582_12392889	2.83511317	12.2804391	1.7975E-06	0.0403971	CTCF	LD

chr2	41134517	41134687	DEL	chr2_41435487_41436304	4.55041806	12.2742705	1.8044E-06	0.0403971	CTCF	LD
chr17	12163846	12163847	INS	chr17_12392582_12392889	2.83511317	12.2804391	1.7975E-06	0.0403971	CTCF	LD
chr14	111917899	111918341	DEL	chr14_111821316_111821680	3.45014039	12.2404364	1.8428E-06	0.04106783	CTCF	LD
chr7	22807665	22807798	DEL	chr7_23147107_23148358	4.08320938	12.2132356	1.8744E-06	0.04106783	CTCF	LD
chr3	122108024	122108025	INS	chr3_122300897_122301195	4.23781188	12.207277	1.8814E-06	0.04106783	CTCF	LD
chr3	122132109	122132110	INS	chr3_122300897_122301195	4.23781188	12.207277	1.8814E-06	0.04106783	CTCF	LD
chr3	122780110	122780111	INS	chr3_122300897_122301195	4.23781188	12.207277	1.8814E-06	0.04106783	CTCF	LD
chr14	88672713	88672714	INS	chr14_88827597_88827846	3.24808311	12.1669158	1.9295E-06	0.04177094	CTCF	LD
chr14	89198219	89198220	INS	chr14_88827597_88827846	3.24808311	12.1669158	1.9295E-06	0.04177094	CTCF	LD
chr13	122537580	122537733	DEL	chr13_122436828_122437128	2.91366654	12.1193533	1.988E-06	0.04286086	CTCF	LD
chr3	58398188	58398189	INS	chr3_58464219_58466776	5.2357905	12.1049406	2.0062E-06	0.04289857	CTCF	LD
chr3	58568874	58568875	INS	chr3_58464219_58466776	5.2357905	12.1049406	2.0062E-06	0.04289857	CTCF	LD
chr4	82754668	82754736	DEL	chr4_82535398_82535724	4.43996778	12.0591817	2.065E-06	0.04327246	CTCF	LD

chr4	82764608	82764823	DEL	chr4_82535398_82535724	4.43996778	12.0591817	2.065E-06	0.04327246	CTCF	LD
chr4	82768569	82772207	DEL	chr4_82535398_82535724	4.43996778	12.0591817	2.065E-06	0.04327246	CTCF	LD
chr4	82746501	82746502	INS	chr4_82535398_82535724	4.43996778	12.0591817	2.065E-06	0.04327246	CTCF	LD
chr4	82847635	82847636	INS	chr4_82535398_82535724	4.43996778	12.0591817	2.065E-06	0.04327246	CTCF	LD
chr3	36143175	36143233	DEL	chr3_36207832_36208144	3.65041236	12.0084046	2.1325E-06	0.04400881	CTCF	LD
chr3	36214624	36214697	DEL	chr3_36207832_36208144	3.65041236	12.0084046	2.1325E-06	0.04400881	CTCF	LD
chr5	27420044	27420045	INS	chr5_27648808_27649137	4.54147417	12.001298	2.1421E-06	0.04400881	CTCF	LD
chr5	27656326	27656327	INS	chr5_27648808_27649137	4.54147417	12.001298	2.1421E-06	0.04400881	CTCF	LD
chr3	35749396	35749397	INS	chr3_36207832_36208144	3.65041236	12.0084046	2.1325E-06	0.04400881	CTCF	LD
chr11	76149766	76149826	DEL	chr11_75931000_75931281	2.09110288	11.985158	2.1642E-06	0.04402214	CTCF	LD
chr13	66138719	66138720	INS	chr13_66452346_66452719	2.98110347	11.9864387	2.1624E-06	0.04402214	CTCF	LD
chr12	7294070	7294071	INS	chr12_7179823_7180228	1.83265188	11.9824153	2.168E-06	0.04402214	CTCF	LD
chr16	69071	69365	DEL	chr16_287812_288702	2.63177044	11.9436522	2.2221E-06	0.04404519	CTCF	LD

chr14	117867506	117867553	DEL	chr14_117875651_117876165	3.22279306	11.9507971	2.212E-06	0.04404519	CTCF	LD
chr14	118068262	118068312	DEL	chr14_117875651_117876165	3.22279306	11.9507971	2.212E-06	0.04404519	CTCF	LD
chr5	34299335	34301890	DEL	chr5_34776305_34776565	2.38616736	11.8817315	2.3119E-06	0.04404519	CTCF	LD
chr16	737540	737541	INS	chr16_287812_288702	2.63177044	11.9436522	2.2221E-06	0.04404519	CTCF	LD
chr8	5271999	5272000	INS	chr8_5079932_5080243	6.69385344	11.8870446	2.304E-06	0.04404519	CTCF	LD
chr8	5439795	5439796	INS	chr8_5079932_5080243	3.34692672	11.8870446	2.304E-06	0.04404519	CTCF	LD
chr14	117785190	117785191	INS	chr14_117875651_117876165	3.22279306	11.9507971	2.212E-06	0.04404519	CTCF	LD
chr8	5603965	5603966	INS	chr8_5079932_5080243	3.34692672	11.8870446	2.304E-06	0.04404519	CTCF	LD
chr1	245208551	245208552	INS	chr1_244978880_244979324	2.67099661	11.920299	2.2555E-06	0.04404519	CTCF	LD
chr1	245427375	245427376	INS	chr1_244978880_244979324	2.67099661	11.920299	2.2555E-06	0.04404519	CTCF	LD
NW_018084953.1	39122	39123	INS	NW_018085270.1_76729_77113	9.71155972	11.8759666	2.3204E-06	0.04404519	CTCF	LD
NW_018085005.1	61148	61149	INS	chr6_30274494_30274895	2.56094975	11.8883558	2.3021E-06	0.04404519	CTCF	LD
chr5	34861379	34861380	INS	chr5_34776305_34776565	2.38616736	11.8817315	2.3119E-06	0.04404519	CTCF	LD

chr13	184326545	184326546	INS	chr13_184085528_184086930	6.44827882	11.8610752	2.3427E-06	0.04417809	CTCF	LD
chr16	35716659	35716660	INS	chr16_36049817_36051297	7.42233778	11.8528625	2.3551E-06	0.04417809	CTCF	LD
chr15	26335551	26335734	DEL	chr15_26147336_26147607	3.82386	11.8231874	2.4004E-06	0.04459458	CTCF	LD
chr15	26398704	26398896	DEL	chr15_26147336_26147607	3.82386	11.8231874	2.4004E-06	0.04459458	CTCF	LD
chr13	55038517	55038517	BND	chr13_54537621_54537977	3.52114133	11.7465695	2.5222E-06	0.04463951	CTCF	LD
chr16	68565023	68565278	DEL	chr16_68115867_68116136	1.84650269	11.7829957	2.4634E-06	0.04463951	CTCF	LD
chr11	70463376	70463649	DEL	chr11_70637244_70637637	2.70335667	11.7525553	2.5124E-06	0.04463951	CTCF	LD
chr13	36079095	36079096	INS	chr13_36052237_36052470	4.73759063	11.7547866	2.5088E-06	0.04463951	CTCF	LD
chr11	70461795	70461796	INS	chr11_70637244_70637637	2.70335667	11.7525553	2.5124E-06	0.04463951	CTCF	LD
chr11	70507912	70507913	INS	chr11_70637244_70637637	2.70335667	11.7525553	2.5124E-06	0.04463951	CTCF	LD
chr11	70777753	70777754	INS	chr11_70637244_70637637	2.70335667	11.7525553	2.5124E-06	0.04463951	CTCF	LD
chr11	70833263	70833264	INS	chr11_70637244_70637637	2.70335667	11.7525553	2.5124E-06	0.04463951	CTCF	LD
chr11	70886805	70886806	INS	chr11_70637244_70637637	2.70335667	11.7525553	2.5124E-06	0.04463951	CTCF	LD

chr13	55038501	55038502	INS	chr13_54537621_54537977	3.52114133	11.7465695	2.5222E-06	0.04463951	CTCF	LD
chr11	75424096	75424097	INS	chr11_75292673_75292940	1.88569869	11.7849179	2.4604E-06	0.04463951	CTCF	LD
chr10	65307964	65307965	INS	chr10_65322744_65323103	5.26384222	11.8077464	2.4244E-06	0.04463951	CTCF	LD
chr9	135949111	135949112	INS	chr9_135871387_135871686	1.62889444	11.7740556	2.4777E-06	0.04463951	CTCF	LD
chr16	31582028	31582501	DEL	chr16_31290416_31290671	3.34930576	11.7226463	2.5616E-06	0.04518429	CTCF	LD
chr10	22624255	22624347	DEL	chr10_22888116_22888465	3.67070861	11.6765379	2.6395E-06	0.04563653	CTCF	LD
chr14	24037273	24042908	DEL	chr14_23634380_23634693	5.47163228	11.6899077	2.6166E-06	0.04563653	CTCF	LD
chr10	22633531	22633532	INS	chr10_22888116_22888465	3.67070861	11.6765379	2.6395E-06	0.04563653	CTCF	LD
chr10	23252935	23252936	INS	chr10_22888116_22888465	3.67070861	11.6765379	2.6395E-06	0.04563653	CTCF	LD
chr14	23717163	23717164	INS	chr14_23634380_23634693	5.47163228	11.6899077	2.6166E-06	0.04563653	CTCF	LD
chr1	119348941	119348942	INS	chr1_119508594_119509058	3.37852472	11.6163848	2.7451E-06	0.04715198	CTCF	LD
chr1	119432649	119432650	INS	chr1_119508594_119509058	3.37852472	11.6163848	2.7451E-06	0.04715198	CTCF	LD
chr1	217966818	217966819	INS	chr1_217519159_217519537	3.46459711	11.60944	2.7576E-06	0.04721187	CTCF	LD

chr5	70440565	70440844	DEL	chr5_70327983_70328223	2.77047389	11.580738	2.8099E-06	0.04764083	CTCF	LD
chr5	69839061	69839062	INS	chr5_70327983_70328223	2.77047389	11.580738	2.8099E-06	0.04764083	CTCF	LD
chr5	70443439	70443440	INS	chr5_70327983_70328223	2.77047389	11.580738	2.8099E-06	0.04764083	CTCF	LD
chr12	56127419	56127520	DEL	chr12_56039689_56039969	4.03826728	11.357982	3.2561E-06	0.04779106	CTCF	LD
chr14	129222574	129222638	DEL	chr14_129660665_129661482	-2.2302875	-11.395545	3.1756E-06	0.04779106	CTCF	LD
chr14	129437482	129437770	DEL	chr14_129660665_129661482	-2.2302875	-11.395545	3.1756E-06	0.04779106	CTCF	LD
chr14	129468677	129468941	DEL	chr14_129660665_129661482	-2.2302875	-11.395545	3.1756E-06	0.04779106	CTCF	LD
chr14	129584699	129585001	DEL	chr14_129660665_129661482	-2.2302875	-11.395545	3.1756E-06	0.04779106	CTCF	LD
chr14	129848177	129848503	DEL	chr14_129660665_129661482	-2.2302875	-11.395545	3.1756E-06	0.04779106	CTCF	LD
chr14	129931390	129931675	DEL	chr14_129660665_129661482	-2.2302875	-11.395545	3.1756E-06	0.04779106	CTCF	LD
chr1	6536879	6536959	DEL	chr1_6962916_6963833	2.19731556	11.328507	3.3208E-06	0.04779106	CTCF	LD
chr2	147090163	147090219	DEL	chr2_146728367_146728814	2.89227231	11.5086246	2.9464E-06	0.04779106	CTCF	LD
chr16	16394291	16395679	DEL	chr16_16400638_16400947	2.74430479	11.3533808	3.2661E-06	0.04779106	CTCF	LD

chr8	21801807	21801862	DEL	chr8_21681632_21681862	3.10035272	11.3990441	3.1682E-06	0.04779106	CTCF	LD
chr8	21935839	21935965	DEL	chr8_21681632_21681862	3.10035272	11.3990441	3.1682E-06	0.04779106	CTCF	LD
chr5	20663070	20663256	DEL	NW_018084989.1_48288_48545	4.31778164	11.4299833	3.1037E-06	0.04779106	CTCF	LD
chr5	31176745	31176838	DEL	chr5_31347611_31347897	2.88154969	11.4950285	2.973E-06	0.04779106	CTCF	LD
chr13	6917895	6918181	DEL	chr13_7271523_7271811	1.55082019	11.3521603	3.2687E-06	0.04779106	CTCF	LD
chr13	6921263	6921560	DEL	chr13_7271523_7271811	1.55082019	11.3521603	3.2687E-06	0.04779106	CTCF	LD
chr8	48812422	48812599	DEL	chr8_48711350_48711779	2.66579132	11.4151062	3.1345E-06	0.04779106	CTCF	LD
chr7	35518765	35518952	DEL	chr7_35579181_35579563	3.98911183	11.4816857	2.9993E-06	0.04779106	CTCF	LD
chr16	47312992	47313551	DUP	chr16_47476594_47478494	11.8262713	11.3245315	3.3297E-06	0.04779106	CTCF	LD
chr16	47467984	47483275	DUP	chr16_47476594_47478494	11.8262713	11.3245315	3.3297E-06	0.04779106	CTCF	LD
chr12	55551710	55551711	INS	chr12_56039689_56039969	4.03826728	11.357982	3.2561E-06	0.04779106	CTCF	LD
chr12	55709314	55709315	INS	chr12_56039689_56039969	4.03826728	11.357982	3.2561E-06	0.04779106	CTCF	LD
chr14	129583021	129583022	INS	chr14_129660665_129661482	-2.2302875	-11.395545	3.1756E-06	0.04779106	CTCF	LD

chr14	129640944	129640945	INS	chr14_129660665_129661482	-2.2302875	-11.395545	3.1756E-06	0.04779106	CTCF	LD
chr14	129729439	129729440	INS	chr14_129660665_129661482	-2.2302875	-11.395545	3.1756E-06	0.04779106	CTCF	LD
chr14	129839166	129839167	INS	chr14_129660665_129661482	-2.2302875	-11.395545	3.1756E-06	0.04779106	CTCF	LD
chr1	6563425	6563426	INS	chr1_6962916_6963833	2.19731556	11.328507	3.3208E-06	0.04779106	CTCF	LD
chr4	120182505	120182506	INS	chr4_120573807_120574058	3.62491811	11.4531055	3.0565E-06	0.04779106	CTCF	LD
chr1	6775811	6775812	INS	chr1_6962916_6963833	2.19731556	11.328507	3.3208E-06	0.04779106	CTCF	LD
chr1	7079894	7079895	INS	chr1_6962916_6963833	2.19731556	11.328507	3.3208E-06	0.04779106	CTCF	LD
chr1	7418800	7418801	INS	chr1_6962916_6963833	2.19731556	11.328507	3.3208E-06	0.04779106	CTCF	LD
chr1	148336394	148336395	INS	chr1_148713224_148713502	2.66476989	11.5632027	2.8425E-06	0.04779106	CTCF	LD
chr13	159887182	159887183	INS	chr13_159837478_159837787	3.52638264	11.4366385	3.0901E-06	0.04779106	CTCF	LD
chr13	160071096	160071097	INS	chr13_159837478_159837787	3.52638264	11.4366385	3.0901E-06	0.04779106	CTCF	LD
chr6	14361557	14361558	INS	chr6_13946079_13946487	1.69650869	11.4843226	2.9941E-06	0.04779106	CTCF	LD
chr8	21736483	21736484	INS	chr8_21681632_21681862	3.10035272	11.3990441	3.1682E-06	0.04779106	CTCF	LD

chr16	16781998	16781999	INS	chr16_16400638_16400947	2.74430479	11.3533808	3.2661E-06	0.04779106	CTCF	LD
chr8	21916355	21916356	INS	chr8_21681632_21681862	3.10035272	11.3990441	3.1682E-06	0.04779106	CTCF	LD
chr16	16860865	16860866	INS	chr16_16400638_16400947	2.74430479	11.3533808	3.2661E-06	0.04779106	CTCF	LD
chr6	31891706	31891707	INS	chr6_32280825_32281118	3.45410856	11.3690591	3.2321E-06	0.04779106	CTCF	LD
chr6	32175004	32175005	INS	chr6_32280825_32281118	3.45410856	11.3690591	3.2321E-06	0.04779106	CTCF	LD
chr6	32259536	32259537	INS	chr6_32280825_32281118	3.45410856	11.3690591	3.2321E-06	0.04779106	CTCF	LD
chr6	32259536	32259537	INS	chr6_32751273_32751634	3.40146382	11.4608162	3.041E-06	0.04779106	CTCF	LD
chr13	7012357	7012358	INS	chr13_7271523_7271811	1.55082019	11.3521603	3.2687E-06	0.04779106	CTCF	LD
chr13	7031264	7031265	INS	chr13_7271523_7271811	1.55082019	11.3521603	3.2687E-06	0.04779106	CTCF	LD
chr7	24970011	24970012	INS	chr7_24598943_24599823	14.7125192	11.5494941	2.8682E-06	0.04779106	CTCF	LD
chr7	25032770	25032771	INS	chr7_24598943_24599823	14.7125192	11.5494941	2.8682E-06	0.04779106	CTCF	LD
chr8	48533511	48533512	INS	chr8_48711350_48711779	2.66579132	11.4151062	3.1345E-06	0.04779106	CTCF	LD
chr16	47117172	47117173	INS	chr16_47476594_47478494	5.91313563	11.3245315	3.3297E-06	0.04779106	CTCF	LD

chr8	48812012	48812013	INS	chr8_48711350_48711779	2.66579132	11.4151062	3.1345E-06	0.04779106	CTCF	LD
chr8	48824710	48824711	INS	chr8_48711350_48711779	2.66579132	11.4151062	3.1345E-06	0.04779106	CTCF	LD
chr16	47313537	47313538	INS	chr16_47476594_47478494	5.91313563	11.3245315	3.3297E-06	0.04779106	CTCF	LD
chr16	47334985	47334986	INS	chr16_47476594_47478494	5.91313563	11.3245315	3.3297E-06	0.04779106	CTCF	LD
chr7	35519348	35519349	INS	chr7_35579181_35579563	3.98911183	11.4816857	2.9993E-06	0.04779106	CTCF	LD
chr1	158720691	158720692	INS	chr1_158477990_158478421	1.9893525	11.2874853	3.4133E-06	0.04859281	CTCF	LD
chr1	158838444	158838445	INS	chr1_158477990_158478421	3.978705	11.2874853	3.4133E-06	0.04859281	CTCF	LD
chr1	158969836	158969837	INS	chr1_158477990_158478421	1.9893525	11.2874853	3.4133E-06	0.04859281	CTCF	LD
chr14	103055983	103056147	DEL	chr14_102719675_102720037	1.09743889	11.2624129	3.4713E-06	0.04862528	CTCF	LD
chr14	103073243	103073467	DEL	chr14_102719675_102720037	1.09743889	11.2624129	3.4713E-06	0.04862528	CTCF	LD
chr14	102840297	102840298	INS	chr14_102719675_102720037	1.09743889	11.2624129	3.4713E-06	0.04862528	CTCF	LD
chr14	102885821	102885822	INS	chr14_102719675_102720037	1.09743889	11.2624129	3.4713E-06	0.04862528	CTCF	LD
chr14	103053703	103053704	INS	chr14_102719675_102720037	1.09743889	11.2624129	3.4713E-06	0.04862528	CTCF	LD

chr17	12373991	12374065	DEL	chr17_12014650_12014999	3.66128856	11.2319436	3.5432E-06	0.04910772	CTCF	LD
chr17	12014421	12014422	INS	chr17_12014650_12014999	3.66128856	11.2319436	3.5432E-06	0.04910772	CTCF	LD
chr17	12245151	12245152	INS	chr17_12014650_12014999	3.66128856	11.2319436	3.5432E-06	0.04910772	CTCF	LD
NW_018085257.1	1716781	1716781	BND	chr1_262924432_262925036	4.62662825	11.2003936	3.6195E-06	0.04917887	CTCF	LD
chr7	61789011	61789098	DEL	chr7_61335933_61336219	4.33403172	10.9956447	4.1613E-06	0.04917887	CTCF	LD
chr4	4230844	4230916	DEL	chr4_4639665_4639989	6.06739194	11.0850446	3.9143E-06	0.04917887	CTCF	LD
chr6	125469742	125469967	DEL	chr6_125554733_125555021	4.30137708	11.1412032	3.7675E-06	0.04917887	CTCF	LD
chr6	125737861	125738418	DEL	chr6_125554733_125555021	4.30137708	11.1412032	3.7675E-06	0.04917887	CTCF	LD
chr15	123018021	123018109	DEL	chr15_123229053_123229417	4.07355078	11.1133427	3.8396E-06	0.04917887	CTCF	LD
chr2	107664242	107664409	DEL	chr2_107813882_107814339	1.91009261	11.1077896	3.8541E-06	0.04917887	CTCF	LD
chr8	129177935	129178413	DEL	chr8_129135684_129136132	2.45877594	11.0641304	3.9706E-06	0.04917887	CTCF	LD
chr6	7807449	7807502	DEL	chr6_8100927_8101478	2.32774514	10.9815312	4.2018E-06	0.04917887	CTCF	LD
chr18	14423748	14424051	DEL	chr18_14694778_14696518	5.1004325	10.9609362	4.2618E-06	0.04917887	CTCF	LD

chr18	14497053	14497366	DEL	chr18_14694778_14696518	5.1004325	10.9609362	4.2618E-06	0.04917887	CTCF	LD
chr18	14706980	14707650	DEL	chr18_14694778_14696518	5.1004325	10.9609362	4.2618E-06	0.04917887	CTCF	LD
chr18	14714382	14714696	DEL	chr18_14694778_14696518	5.1004325	10.9609362	4.2618E-06	0.04917887	CTCF	LD
chr18	14713376	14713653	DEL	chr18_14694778_14696518	5.1004325	10.9609362	4.2618E-06	0.04917887	CTCF	LD
chr18	15139566	15139867	DEL	chr18_14694778_14696518	5.1004325	10.9609362	4.2618E-06	0.04917887	CTCF	LD
chr2	27163221	27163500	DEL	chr2_27257872_27258645	7.64309097	11.168968	3.6973E-06	0.04917887	CTCF	LD
chr2	27570340	27570686	DEL	chr2_27257872_27258645	7.64309097	11.168968	3.6973E-06	0.04917887	CTCF	LD
chr12	36525565	36525616	DEL	chr12_36164406_36164781	2.18549011	10.9716272	4.2306E-06	0.04917887	CTCF	LD
chr12	36637329	36637394	DEL	chr12_36164406_36164781	2.18549011	10.9716272	4.2306E-06	0.04917887	CTCF	LD
chr7	24769188	24769497	DEL	chr7_25192757_25193511	2.01960833	10.9970472	4.1573E-06	0.04917887	CTCF	LD
chr5	34593028	34595257	DEL	chr5_35076796_35077215	3.77019563	10.997364	4.1564E-06	0.04917887	CTCF	LD
chr7	25172350	25173022	DEL	chr7_25192757_25193511	2.01960833	10.9970472	4.1573E-06	0.04917887	CTCF	LD
chr5	35066345	35066560	DEL	chr5_35076796_35077215	3.77019563	10.997364	4.1564E-06	0.04917887	CTCF	LD

chr5	35266906	35267072	DEL	chr5_35076796_35077215	3.77019563	10.997364	4.1564E-06	0.04917887	CTCF	LD
chr3	2563073	2563141	DEL	chr3_2923151_2923445	3.00534167	10.9998171	4.1494E-06	0.04917887	CTCF	LD
chr3	2540247	2540521	DEL	chr3_2923151_2923445	3.00534167	10.9998171	4.1494E-06	0.04917887	CTCF	LD
chr3	2597019	2597242	DEL	chr3_2923151_2923445	3.00534167	10.9998171	4.1494E-06	0.04917887	CTCF	LD
chr8	61833151	61833152	INS	chr8_61738140_61738470	2.92933506	11.1444956	3.7591E-06	0.04917887	CTCF	LD
chr6	83999580	83999581	INS	chr6_84371228_84372256	11.5805667	10.9630057	4.2557E-06	0.04917887	CTCF	LD
chr6	84222676	84222677	INS	chr6_84371228_84372256	11.5805667	10.9630057	4.2557E-06	0.04917887	CTCF	LD
chr6	84217291	84217292	INS	chr6_84371228_84372256	11.5805667	10.9630057	4.2557E-06	0.04917887	CTCF	LD
chr6	84191619	84191620	INS	chr6_84371228_84372256	11.5805667	10.9630057	4.2557E-06	0.04917887	CTCF	LD
chr6	84204760	84204761	INS	chr6_84371228_84372256	11.5805667	10.9630057	4.2557E-06	0.04917887	CTCF	LD
chr6	84682861	84682862	INS	chr6_84371228_84372256	11.5805667	10.9630057	4.2557E-06	0.04917887	CTCF	LD
chr7	61177243	61177244	INS	chr7_61335933_61336219	4.33403172	10.9956447	4.1613E-06	0.04917887	CTCF	LD
chr11	65170035	65170036	INS	chr11_65647224_65647505	4.86724597	11.0868619	3.9094E-06	0.04917887	CTCF	LD

chr4	4314440	4314441	INS	chr4_4639665_4639989	6.06739194	11.0850446	3.9143E-06	0.04917887	CTCF	LD
chr13	64851525	64851526	INS	chr13_64454900_64455145	4.64958639	11.2058516	3.6062E-06	0.04917887	CTCF	LD
chr6	125095591	125095592	INS	chr6_125554733_125555021	4.30137708	11.1412032	3.7675E-06	0.04917887	CTCF	LD
chr6	125584865	125584866	INS	chr6_125554733_125555021	4.30137708	11.1412032	3.7675E-06	0.04917887	CTCF	LD
chr6	125720437	125720438	INS	chr6_125554733_125555021	4.30137708	11.1412032	3.7675E-06	0.04917887	CTCF	LD
chr6	125798995	125798996	INS	chr6_125554733_125555021	4.30137708	11.1412032	3.7675E-06	0.04917887	CTCF	LD
chr15	122780520	122780521	INS	chr15_123229053_123229417	4.07355078	11.1133427	3.8396E-06	0.04917887	CTCF	LD
chr15	122775984	122775985	INS	chr15_123229053_123229417	4.07355078	11.1133427	3.8396E-06	0.04917887	CTCF	LD
chr15	123227257	123227258	INS	chr15_123229053_123229417	4.07355078	11.1133427	3.8396E-06	0.04917887	CTCF	LD
chr15	123616361	123616362	INS	chr15_123229053_123229417	4.07355078	11.1133427	3.8396E-06	0.04917887	CTCF	LD
chr7	93326138	93326139	INS	chr7_93350025_93351953	3.10604344	11.2004103	3.6195E-06	0.04917887	CTCF	LD
chr8	129026947	129026948	INS	chr8_129135684_129136132	2.45877594	11.0641304	3.9706E-06	0.04917887	CTCF	LD
chr8	129134268	129134269	INS	chr8_129135684_129136132	2.45877594	11.0641304	3.9706E-06	0.04917887	CTCF	LD

chr8	129349953	129349954	INS	chr8_129135684_129136132	2.45877594	11.0641304	3.9706E-06	0.04917887	CTCF	LD
chr6	8046631	8046632	INS	chr6_8100927_8101478	2.32774514	10.9815312	4.2018E-06	0.04917887	CTCF	LD
chr6	8565893	8565894	INS	chr6_8100927_8101478	2.32774514	10.9815312	4.2018E-06	0.04917887	CTCF	LD
chr6	14361557	14361558	INS	chr6_14135169_14135420	1.35798944	11.0942371	3.8898E-06	0.04917887	CTCF	LD
chr2	26883946	26883947	INS	chr2_27257872_27258645	7.64309097	11.168968	3.6973E-06	0.04917887	CTCF	LD
chr2	27143051	27143052	INS	chr2_27257872_27258645	7.64309097	11.168968	3.6973E-06	0.04917887	CTCF	LD
chr2	27377526	27377527	INS	chr2_27257872_27258645	7.64309097	11.168968	3.6973E-06	0.04917887	CTCF	LD
chr2	27725953	27725954	INS	chr2_27257872_27258645	7.64309097	11.168968	3.6973E-06	0.04917887	CTCF	LD
chr2	27763493	27763494	INS	chr2_27257872_27258645	7.64309097	11.168968	3.6973E-06	0.04917887	CTCF	LD
chr12	35880829	35880830	INS	chr12_36164406_36164781	2.18549011	10.9716272	4.2306E-06	0.04917887	CTCF	LD
chr12	36247623	36247624	INS	chr12_36164406_36164781	2.18549011	10.9716272	4.2306E-06	0.04917887	CTCF	LD
chr12	36244034	36244035	INS	chr12_36164406_36164781	2.18549011	10.9716272	4.2306E-06	0.04917887	CTCF	LD
chr12	36271321	36271322	INS	chr12_36164406_36164781	2.18549011	10.9716272	4.2306E-06	0.04917887	CTCF	LD

chr12	36509954	36509955	INS	chr12_36164406_36164781	2.18549011	10.9716272	4.2306E-06	0.04917887	CTCF	LD
chr5	34615878	34615879	INS	chr5_35076796_35077215	3.77019563	10.997364	4.1564E-06	0.04917887	CTCF	LD
chr5	34942195	34942196	INS	chr5_35076796_35077215	3.77019563	10.997364	4.1564E-06	0.04917887	CTCF	LD
chr5	35066445	35066446	INS	chr5_35076796_35077215	3.77019563	10.997364	4.1564E-06	0.04917887	CTCF	LD
chr5	35335671	35335672	INS	chr5_35076796_35077215	3.77019563	10.997364	4.1564E-06	0.04917887	CTCF	LD
chr3	2823792	2823793	INS	chr3_2923151_2923445	3.00534167	10.9998171	4.1494E-06	0.04917887	CTCF	LD
chr3	2827690	2827691	INS	chr3_2923151_2923445	3.00534167	10.9998171	4.1494E-06	0.04917887	CTCF	LD
chr3	2895439	2895440	INS	chr3_2923151_2923445	3.00534167	10.9998171	4.1494E-06	0.04917887	CTCF	LD
chr15	50635946	50636039	DEL	chr15_50308923_50309467	3.15152181	10.9564062	4.2751E-06	0.04918555	CTCF	LD
chr17	18865727	18865808	DEL	chr17_18902511_18902821	2.769324	10.9321477	4.3473E-06	0.04951065	CTCF	LD
chr17	18819836	18819837	INS	chr17_18902511_18902821	2.769324	10.9321477	4.3473E-06	0.04951065	CTCF	LD
chr17	18910519	18910520	INS	chr17_18902511_18902821	2.769324	10.9321477	4.3473E-06	0.04951065	CTCF	LD
chr17	19103728	19103729	INS	chr17_18902511_18902821	2.769324	10.9321477	4.3473E-06	0.04951065	CTCF	LD

chr7	2297614	2297763	DEL	chr7_2276196_2276868	6.64534883	51.8961701	2.1062E-11	2.2068E-05	CTCF	LD
chr7	2042143	2042144	INS	chr7_2276196_2276868	6.64534883	51.8961701	2.1062E-11	2.2068E-05	CTCF	LD
chr7	2162579	2162580	INS	chr7_2276196_2276868	6.64534883	51.8961701	2.1062E-11	2.2068E-05	CTCF	LD
chr7	2279026	2279027	INS	chr7_2276196_2276868	6.64534883	51.8961701	2.1062E-11	2.2068E-05	CTCF	LD
chr7	2297614	2297763	DEL	chr7_2276134_2277019	6.72353244	31.4495266	1.1369E-09	0.00013876	H3K27ac	LD
chr7	1980313	1980314	INS	chr7_2276134_2277019	6.72353244	31.4495266	1.1369E-09	0.00013876	H3K27ac	LD
chr7	2042143	2042144	INS	chr7_2276134_2277019	6.72353244	31.4495266	1.1369E-09	0.00013876	H3K27ac	LD
chr7	2162579	2162580	INS	chr7_2276134_2277019	6.72353244	31.4495266	1.1369E-09	0.00013876	H3K27ac	LD
chr7	2279026	2279027	INS	chr7_2276134_2277019	6.72353244	31.4495266	1.1369E-09	0.00013876	H3K27ac	LD
chr3	125499714	125499807	DEL	chr3_125944493_125945385	5.09108711	30.0044747	1.6516E-09	0.00017918	H3K27ac	LD
chr3	125500735	125500977	DEL	chr3_125944493_125945385	5.09108711	30.0044747	1.6516E-09	0.00017918	H3K27ac	LD
chr3	125625191	125625192	INS	chr3_125944493_125945385	5.09108711	30.0044747	1.6516E-09	0.00017918	H3K27ac	LD
chr3	125637733	125637734	INS	chr3_125944493_125945385	5.09108711	30.0044747	1.6516E-09	0.00017918	H3K27ac	LD

chr3	125802095	125802096	INS	chr3_125944493_125945385	5.09108711	30.0044747	1.6516E-09	0.00017918	H3K27ac	LD
chr3	126060760	126060761	INS	chr3_125944493_125945385	5.09108711	30.0044747	1.6516E-09	0.00017918	H3K27ac	LD
chr7	9438300	9438436	DEL	chr7_9866559_9867297	3.28566333	29.5581859	1.8601E-09	0.00019119	H3K27ac	LD
chr7	9751355	9751407	DEL	chr7_9866559_9867297	3.28566333	29.5581859	1.8601E-09	0.00019119	H3K27ac	LD
chr7	9891111	9891112	INS	chr7_9866559_9867297	3.28566333	29.5581859	1.8601E-09	0.00019119	H3K27ac	LD
chr8	121599857	121599858	INS	chr8_122037174_122038288	2.53353134	29.3757864	1.9538E-09	0.00019162	H3K27ac	LD
chr2	141818450	141818451	INS	chr2_141360008_141362181	8.16935528	29.3595028	1.9624E-09	0.00019162	H3K27ac	LD
chr6	134069272	134069273	INS	chr6_133890347_133891072	2.96045567	27.7301798	3.0861E-09	0.0002964	H3K27ac	LD
chr17	10024732	10024733	INS	chr17_9660611_9661363	0.87291122	27.3985871	3.3948E-09	0.00032079	H3K27ac	LD
chr7	116344914	116344915	INS	chr7_116444722_116445712	3.36431206	27.0025754	3.8099E-09	0.00032349	H3K27ac	LD
chr7	116359034	116359035	INS	chr7_116444722_116445712	3.36431206	27.0025754	3.8099E-09	0.00032349	H3K27ac	LD
chr7	116744972	116744973	INS	chr7_116444722_116445712	3.36431206	27.0025754	3.8099E-09	0.00032349	H3K27ac	LD
chr3	30964455	30964456	INS	chr3_30959032_30960457	2.76281728	27.0897046	3.7139E-09	0.00032349	H3K27ac	LD

chr3	30978161	30978162	INS	chr3_30959032_30960457	2.76281728	27.0897046	3.7139E-09	0.00032349	H3K27ac	LD
chr3	30980916	30980917	INS	chr3_30959032_30960457	2.76281728	27.0897046	3.7139E-09	0.00032349	H3K27ac	LD
chr13	180504790	180505075	DEL	chr13_180891887_180892987	3.09632931	25.9732306	5.1829E-09	0.00033006	H3K27ac	LD
chr13	180730292	180730346	DEL	chr13_180891887_180892987	3.09632931	25.9732306	5.1829E-09	0.00033006	H3K27ac	LD
chr1	242737904	242738840	DEL	chr1_242865565_242866290	6.78123068	26.110683	4.9709E-09	0.00033006	H3K27ac	LD
chr1	243236545	243239086	DEL	chr1_242865565_242866290	6.78123068	26.110683	4.9709E-09	0.00033006	H3K27ac	LD
chr9	8588494	8589789	DEL	chr9_8437804_8439007	6.91097	26.3287071	4.6541E-09	0.00033006	H3K27ac	LD
chr9	8905074	8905473	DEL	chr9_8437804_8439007	6.91097	26.3287071	4.6541E-09	0.00033006	H3K27ac	LD
chr5	72889345	72889346	INS	chr5_72845252_72845511	2.57811251	26.0706386	5.0316E-09	0.00033006	H3K27ac	LD
chr5	73320470	73320471	INS	chr5_72845252_72845511	2.57811251	26.0706386	5.0316E-09	0.00033006	H3K27ac	LD
chr10	61020139	61020140	INS	chr10_61019745_61020615	3.20324561	26.2220556	4.8061E-09	0.00033006	H3K27ac	LD
chr10	61028443	61028444	INS	chr10_61019745_61020615	3.20324561	26.2220556	4.8061E-09	0.00033006	H3K27ac	LD
chr13	180967033	180967034	INS	chr13_180891887_180892987	3.09632931	25.9732306	5.1829E-09	0.00033006	H3K27ac	LD

chr13	181112644	181112645	INS	chr13_180891887_180892987	3.09632931	25.9732306	5.1829E-09	0.00033006	H3K27ac	LD
chr1	242506220	242506221	INS	chr1_242865565_242866290	6.78123068	26.110683	4.9709E-09	0.00033006	H3K27ac	LD
chr1	242482359	242482360	INS	chr1_242865565_242866290	6.78123068	26.110683	4.9709E-09	0.00033006	H3K27ac	LD
chr1	242521458	242521459	INS	chr1_242865565_242866290	6.78123068	26.110683	4.9709E-09	0.00033006	H3K27ac	LD
chr1	242866604	242866605	INS	chr1_242865565_242866290	6.78123068	26.110683	4.9709E-09	0.00033006	H3K27ac	LD
chr1	243249032	243249033	INS	chr1_242865565_242866290	6.78123068	26.110683	4.9709E-09	0.00033006	H3K27ac	LD
chr9	8092921	8092922	INS	chr9_8437804_8439007	6.91097	26.3287071	4.6541E-09	0.00033006	H3K27ac	LD
chr9	8666806	8666807	INS	chr9_8437804_8439007	6.91097	26.3287071	4.6541E-09	0.00033006	H3K27ac	LD
chr9	8866106	8866107	INS	chr9_8437804_8439007	6.91097	26.3287071	4.6541E-09	0.00033006	H3K27ac	LD
chr9	8865150	8865151	INS	chr9_8437804_8439007	6.91097	26.3287071	4.6541E-09	0.00033006	H3K27ac	LD
chr6	151260873	151260874	INS	chr6_151272666_151273246	2.54817254	25.8171009	5.4363E-09	0.00034247	H3K27ac	LD
chr17	59934477	59934606	DEL	chr17_60070060_60072111	3.99061675	25.2235231	6.5348E-09	0.00038315	H3K27ac	LD
chr17	60029359	60029487	DEL	chr17_60070060_60072111	3.99061675	25.2235231	6.5348E-09	0.00038315	H3K27ac	LD

chr17	59938267	59938268	INS	chr17_60070060_60072111	3.99061675	25.2235231	6.5348E-09	0.00038315	H3K27ac	LD
chr17	60527683	60527684	INS	chr17_60070060_60072111	3.99061675	25.2235231	6.5348E-09	0.00038315	H3K27ac	LD
chr6	151260873	151260874	INS	chr6_151273508_151273963	3.08465033	25.2963568	6.3874E-09	0.00038315	H3K27ac	LD
chr7	11239143	11239144	INS	chr7_11722951_11723347	14.3467006	25.126531	6.737E-09	0.00039079	H3K27ac	LD
chr9	33837090	33837090	BND	chr9_33837052_33837911	2.61492972	24.3256632	8.7037E-09	0.00047215	H3K27ac	LD
chr9	33526849	33527066	DEL	chr9_33837052_33837911	2.61492972	24.3256632	8.7037E-09	0.00047215	H3K27ac	LD
chr9	33684729	33685050	DEL	chr9_33837052_33837911	2.61492972	24.3256632	8.7037E-09	0.00047215	H3K27ac	LD
chr9	33557955	33557956	INS	chr9_33837052_33837911	2.61492972	24.3256632	8.7037E-09	0.00047215	H3K27ac	LD
chr9	33850463	33850464	INS	chr9_33837052_33837911	2.61492972	24.3256632	8.7037E-09	0.00047215	H3K27ac	LD
chr9	33837071	33837072	INS	chr9_33837052_33837911	2.61492972	24.3256632	8.7037E-09	0.00047215	H3K27ac	LD
chr9	34100911	34100912	INS	chr9_33837052_33837911	2.61492972	24.3256632	8.7037E-09	0.00047215	H3K27ac	LD
chr2	1729822	1729823	INS	chr2_1484061_1485178	5.77827617	23.6844125	1.0749E-08	0.00057776	H3K27ac	LD
chr12	5287156	5287157	INS	chr12_5291251_5292659	2.07784169	22.4079247	1.6645E-08	0.0008707	H3K27ac	LD

chr12	5647558	5647559	INS	chr12_5291251_5292659	2.07784169	22.4079247	1.6645E-08	0.0008707	H3K27ac	LD
chr9	43934464	43934465	INS	chr9_44133408_44134082	3.84843019	22.3776324	1.6824E-08	0.00087225	H3K27ac	LD
chr13	189546379	189548076	DEL	chr13_189449662_189450677	2.65463867	21.8628661	2.0212E-08	0.0009616	H3K27ac	LD
chr7	32496108	32496225	DEL	chr7_32931392_32932760	2.42336125	21.7993384	2.0681E-08	0.0009616	H3K27ac	LD
chr13	188983904	188983905	INS	chr13_189449662_189450677	2.65463867	21.8628661	2.0212E-08	0.0009616	H3K27ac	LD
chr13	189143589	189143590	INS	chr13_189449662_189450677	2.65463867	21.8628661	2.0212E-08	0.0009616	H3K27ac	LD
chr13	189346991	189346992	INS	chr13_189449662_189450677	2.65463867	21.8628661	2.0212E-08	0.0009616	H3K27ac	LD
chr13	189680325	189680326	INS	chr13_189449662_189450677	2.65463867	21.8628661	2.0212E-08	0.0009616	H3K27ac	LD
chr13	189873403	189873404	INS	chr13_189449662_189450677	2.65463867	21.8628661	2.0212E-08	0.0009616	H3K27ac	LD
chr13	189922350	189922351	INS	chr13_189449662_189450677	5.30927734	21.8628661	2.0212E-08	0.0009616	H3K27ac	LD
chr6	19872529	19872530	INS	chr6_19788532_19790046	2.8147545	21.8188147	2.0536E-08	0.0009616	H3K27ac	LD
chr6	19947161	19947162	INS	chr6_19788532_19790046	2.8147545	21.8188147	2.0536E-08	0.0009616	H3K27ac	LD
chr6	20042223	20042224	INS	chr6_19788532_19790046	2.8147545	21.8188147	2.0536E-08	0.0009616	H3K27ac	LD

chr6	20043093	20043094	INS	chr6_19788532_19790046	2.8147545	21.8188147	2.0536E-08	0.0009616	H3K27ac	LD
chr6	20274349	20274350	INS	chr6_19788532_19790046	2.8147545	21.8188147	2.0536E-08	0.0009616	H3K27ac	LD
chr12	58500972	58501269	DEL	chr12_58729207_58730627	5.48067125	21.2365964	2.5412E-08	0.0011365	H3K27ac	LD
chr12	58528114	58528166	DEL	chr12_58729207_58730627	10.9613425	21.2365964	2.5412E-08	0.0011365	H3K27ac	LD
chr12	58508126	58508127	INS	chr12_58729207_58730627	5.48067125	21.2365964	2.5412E-08	0.0011365	H3K27ac	LD
chr12	58762838	58762839	INS	chr12_58729207_58730627	5.48067125	21.2365964	2.5412E-08	0.0011365	H3K27ac	LD
chr12	58508127	58534400	INV	chr12_58729207_58730627	10.9613425	21.2365964	2.5412E-08	0.0011365	H3K27ac	LD
chr15	81711469	81711890	DEL	chr15_81434118_81435263	2.62242094	21.0298521	2.7447E-08	0.00120904	H3K27ac	LD
chr15	81755305	81755306	INS	chr15_81434118_81435263	2.62242094	21.0298521	2.7447E-08	0.00120904	H3K27ac	LD
chr4	63605907	63605908	INS	chr4_63671353_63672173	2.00100417	20.7995849	2.9932E-08	0.0012894	H3K27ac	LD
chr4	63730806	63730807	INS	chr4_63671353_63672173	2.00100417	20.7995849	2.9932E-08	0.0012894	H3K27ac	LD
chr4	63781904	63781905	INS	chr4_63671353_63672173	2.00100417	20.7995849	2.9932E-08	0.0012894	H3K27ac	LD
chr6	5625414	5625474	DEL	chr6_5686417_5686910	4.42920889	20.2488053	3.6966E-08	0.00147663	H3K27ac	LD

chr6	5843921	5843983	DEL	chr6_5686417_5686910	4.42920889	20.2488053	3.6966E-08	0.00147663	H3K27ac	LD
chr11	3527785	3527786	INS	chr11_3619671_3620543	2.64558838	20.3754981	3.5197E-08	0.00147663	H3K27ac	LD
chr11	3613718	3613719	INS	chr11_3619671_3620543	2.64558838	20.3754981	3.5197E-08	0.00147663	H3K27ac	LD
chr6	5418221	5418222	INS	chr6_5686417_5686910	4.42920889	20.2488053	3.6966E-08	0.00147663	H3K27ac	LD
chr6	5413829	5413830	INS	chr6_5686417_5686910	4.42920889	20.2488053	3.6966E-08	0.00147663	H3K27ac	LD
chr18	33851164	33851165	INS	chr18_34326468_34326874	4.20007522	20.2255128	3.7302E-08	0.00147663	H3K27ac	LD
chr18	33948532	33948533	INS	chr18_34326468_34326874	4.20007522	20.2255128	3.7302E-08	0.00147663	H3K27ac	LD
chr18	33990090	33990091	INS	chr18_34326468_34326874	4.20007522	20.2255128	3.7302E-08	0.00147663	H3K27ac	LD
chr18	34103862	34103863	INS	chr18_34326468_34326874	4.20007522	20.2255128	3.7302E-08	0.00147663	H3K27ac	LD
chr18	34220782	34220783	INS	chr18_34326468_34326874	4.20007522	20.2255128	3.7302E-08	0.00147663	H3K27ac	LD
chr7	2711131	2711378	DEL	chr7_2673559_2674193	1.6722055	19.9479552	4.1582E-08	0.00157171	H3K27ac	LD
chr7	3168350	3168981	DEL	chr7_2673559_2674193	1.6722055	19.9479552	4.1582E-08	0.00157171	H3K27ac	LD
chr7	2214332	2214333	INS	chr7_2673559_2674193	1.6722055	19.9479552	4.1582E-08	0.00157171	H3K27ac	LD

chr7	2449976	2449977	INS	chr7_2673559_2674193	1.6722055	19.9479552	4.1582E-08	0.00157171	H3K27ac	LD
chr7	2675915	2675916	INS	chr7_2673559_2674193	1.6722055	19.9479552	4.1582E-08	0.00157171	H3K27ac	LD
chr7	2689666	2689667	INS	chr7_2673559_2674193	1.6722055	19.9479552	4.1582E-08	0.00157171	H3K27ac	LD
chr7	2691233	2691234	INS	chr7_2673559_2674193	1.6722055	19.9479552	4.1582E-08	0.00157171	H3K27ac	LD
chr12	7721606	7722514	DEL	chr12_7681720_7682727	2.70039913	19.8011167	4.4067E-08	0.00164443	H3K27ac	LD
chr12	7708090	7708091	INS	chr12_7681720_7682727	2.70039913	19.8011167	4.4067E-08	0.00164443	H3K27ac	LD
chr18	34696682	34696683	INS	chr18_34325367_34326045	2.86137131	19.7370306	4.5204E-08	0.00167616	H3K27ac	LD
chr2	135773551	135773675	DEL	chr2_136193170_136194237	3.79786238	19.447194	5.0773E-08	0.00184757	H3K27ac	LD
chr2	136156115	136156116	INS	chr2_136193170_136194237	3.79786238	19.447194	5.0773E-08	0.00184757	H3K27ac	LD
chr2	136266529	136266530	INS	chr2_136193170_136194237	3.79786238	19.447194	5.0773E-08	0.00184757	H3K27ac	LD
chr14	135359580	135359581	INS	chr14_135144837_135145520	3.41236094	19.2381958	5.5267E-08	0.00198643	H3K27ac	LD
chr8	41223208	41783661	DUP	chr8_41402038_41403489	23.6634665	14.2467895	5.7535E-08	0.00078348	H3K27ac	LD
chr2	1774590	1775895	DEL	chr2_1935121_1936427	7.45918411	18.9744598	6.1588E-08	0.00220014	H3K27ac	LD

chr5	32170275	32170332	DEL	chr5_32158206_32158926	3.76928819	18.9183555	6.3035E-08	0.00223819	H3K27ac	LD
chr13	71409434	71409435	INS	chr13_71579688_71580785	2.08483856	18.6402778	7.0795E-08	0.00249858	H3K27ac	LD
chr4	78071426	78071706	DEL	chr4_78076288_78077048	2.82078628	18.3455233	8.0212E-08	0.00252654	H3K27ac	LD
chr4	78193552	78194123	DEL	chr4_78076288_78077048	2.82078628	18.3455233	8.0212E-08	0.00252654	H3K27ac	LD
chr15	106432793	106433088	DEL	chr15_106382487_106383530	1.67644613	18.3623353	7.9639E-08	0.00252654	H3K27ac	LD
chr15	106453705	106453996	DEL	chr15_106382487_106383530	1.67644613	18.3623353	7.9639E-08	0.00252654	H3K27ac	LD
chr15	106458083	106459004	DEL	chr15_106382487_106383530	1.67644613	18.3623353	7.9639E-08	0.00252654	H3K27ac	LD
chr15	106729613	106729668	DEL	chr15_106382487_106383530	1.67644613	18.3623353	7.9639E-08	0.00252654	H3K27ac	LD
chr4	77773017	77773018	INS	chr4_78076288_78077048	2.82078628	18.3455233	8.0212E-08	0.00252654	H3K27ac	LD
chr15	106112983	106112984	INS	chr15_106382487_106383530	1.67644613	18.3623353	7.9639E-08	0.00252654	H3K27ac	LD
chr15	106153895	106153896	INS	chr15_106382487_106383530	1.67644613	18.3623353	7.9639E-08	0.00252654	H3K27ac	LD
chr15	106370456	106370457	INS	chr15_106382487_106383530	1.67644613	18.3623353	7.9639E-08	0.00252654	H3K27ac	LD
chr15	106441350	106441351	INS	chr15_106382487_106383530	1.67644613	18.3623353	7.9639E-08	0.00252654	H3K27ac	LD

chr15	106644078	106644079	INS	chr15_106382487_106383530	1.67644613	18.3623353	7.9639E-08	0.00252654	H3K27ac	LD
chr15	106626875	106626876	INS	chr15_106382487_106383530	1.67644613	18.3623353	7.9639E-08	0.00252654	H3K27ac	LD
chr12	19713772	19713773	INS	chr12_19713381_19714949	-3.399332	-18.393163	7.8599E-08	0.00252654	H3K27ac	LD
chr18	33851164	33851165	INS	chr18_34325367_34326045	2.83967956	18.4919954	7.5368E-08	0.00252654	H3K27ac	LD
chr18	33948532	33948533	INS	chr18_34325367_34326045	2.83967956	18.4919954	7.5368E-08	0.00252654	H3K27ac	LD
chr18	33990090	33990091	INS	chr18_34325367_34326045	2.83967956	18.4919954	7.5368E-08	0.00252654	H3K27ac	LD
chr18	34103862	34103863	INS	chr18_34325367_34326045	2.83967956	18.4919954	7.5368E-08	0.00252654	H3K27ac	LD
chr18	34220782	34220783	INS	chr18_34325367_34326045	2.83967956	18.4919954	7.5368E-08	0.00252654	H3K27ac	LD
chr7	3035972	3035973	INS	chr7_2633092_2633727	6.71252413	18.4206783	7.7684E-08	0.00252654	H3K27ac	LD
chr13	84436877	84436926	DEL	chr13_84771848_84772437	2.17605829	18.1687915	8.6529E-08	0.00253473	H3K27ac	LD
chr15	134543780	134543841	DEL	chr15_134559561_134559783	1.96564383	18.1993679	8.5398E-08	0.00253473	H3K27ac	LD
chr15	134557163	134557759	DEL	chr15_134559561_134559783	1.96564383	18.1993679	8.5398E-08	0.00253473	H3K27ac	LD
chr7	7138092	7138148	DEL	chr7_7498807_7499483	2.62789811	18.2312152	8.4237E-08	0.00253473	H3K27ac	LD

chr7	7150239	7150309	DEL	chr7_7498807_7499483	2.62789811	18.2312152	8.4237E-08	0.00253473	H3K27ac	LD
chr7	7393187	7393626	DEL	chr7_7498807_7499483	2.62789811	18.2312152	8.4237E-08	0.00253473	H3K27ac	LD
chr13	191426332	191426451	DEL	chr13_190984878_190985146	2.02507044	18.1707773	8.6455E-08	0.00253473	H3K27ac	LD
chr13	84945487	84945488	INS	chr13_84771848_84772437	2.17605829	18.1687915	8.6529E-08	0.00253473	H3K27ac	LD
chr13	84953448	84953449	INS	chr13_84771848_84772437	2.17605829	18.1687915	8.6529E-08	0.00253473	H3K27ac	LD
chr13	84948141	84948142	INS	chr13_84771848_84772437	2.17605829	18.1687915	8.6529E-08	0.00253473	H3K27ac	LD
chr1	131246835	131246836	INS	chr1_131391870_131392190	2.06709428	18.2184208	8.4701E-08	0.00253473	H3K27ac	LD
chr13	190551633	190551634	INS	chr13_190984878_190985146	2.02507044	18.1707773	8.6455E-08	0.00253473	H3K27ac	LD
chr13	191377863	191377864	INS	chr13_190984878_190985146	2.02507044	18.1707773	8.6455E-08	0.00253473	H3K27ac	LD
chr13	191349111	191349112	INS	chr13_190984878_190985146	2.02507044	18.1707773	8.6455E-08	0.00253473	H3K27ac	LD
chr18	46260011	46260205	DEL	chr18_46141784_46142107	1.78566	17.8494404	9.9412E-08	0.00285501	H3K27ac	LD
chr18	46080304	46080305	INS	chr18_46141784_46142107	1.78566	17.8494404	9.9412E-08	0.00285501	H3K27ac	LD
chr18	46212934	46212935	INS	chr18_46141784_46142107	1.78566	17.8494404	9.9412E-08	0.00285501	H3K27ac	LD

chr18	46531849	46531850	INS	chr18_46141784_46142107	1.78566	17.8494404	9.9412E-08	0.00285501	H3K27ac	LD
chr11	2277813	2278481	DEL	chr11_2238238_2238532	2.23198888	17.7992147	1.0163E-07	0.00287635	H3K27ac	LD
chr12	47048652	47048962	DEL	chr12_47387457_47389780	4.1634175	17.8057755	1.0134E-07	0.00287635	H3K27ac	LD
chr11	2278490	2278491	INS	chr11_2238238_2238532	2.23198888	17.7992147	1.0163E-07	0.00287635	H3K27ac	LD
chr4	78071426	78071706	DEL	chr4_78440064_78440343	1.47108389	17.5652793	1.1271E-07	0.00309002	H3K27ac	LD
chr4	78193552	78194123	DEL	chr4_78440064_78440343	1.47108389	17.5652793	1.1271E-07	0.00309002	H3K27ac	LD
chr5	91273146	91273676	DEL	chr5_91274322_91275534	2.48538065	17.5824901	1.1185E-07	0.00309002	H3K27ac	LD
chr5	90989883	90989884	INS	chr5_91274322_91275534	2.48538065	17.5824901	1.1185E-07	0.00309002	H3K27ac	LD
chr7	7397868	7397869	INS	chr7_7498807_7499483	2.64182163	17.5515525	1.134E-07	0.00309002	H3K27ac	LD
chr7	7556180	7556181	INS	chr7_7498807_7499483	2.64182163	17.5515525	1.134E-07	0.00309002	H3K27ac	LD
chr7	7542379	7542380	INS	chr7_7498807_7499483	2.64182163	17.5515525	1.134E-07	0.00309002	H3K27ac	LD
chr13	170214702	170214703	INS	chr13_169918142_169920188	2.71406289	17.3639	1.2334E-07	0.00334528	H3K27ac	LD
chr12	55037406	55037407	INS	chr12_54785475_54785770	2.01673425	17.2521708	1.2972E-07	0.0035021	H3K27ac	LD

chr2	117023543	117024038	DEL	chr2_117510937_117512352	2.58599589	17.1819582	1.3391E-07	0.00352882	H3K27ac	LD
chr2	117478046	117478188	DEL	chr2_117510937_117512352	2.58599589	17.1819582	1.3391E-07	0.00352882	H3K27ac	LD
chr2	117524108	117524170	DEL	chr2_117510937_117512352	2.58599589	17.1819582	1.3391E-07	0.00352882	H3K27ac	LD
chr2	143084053	143084526	DEL	chr2_143050210_143050566	2.16829122	17.1459481	1.3613E-07	0.00352882	H3K27ac	LD
chr1	71487847	71487848	INS	chr1_71487843_71488591	2.2458335	17.1860726	1.3366E-07	0.00352882	H3K27ac	LD
chr2	142562359	142562360	INS	chr2_143050210_143050566	2.16829122	17.1459481	1.3613E-07	0.00352882	H3K27ac	LD
chr2	142646589	142646590	INS	chr2_143050210_143050566	2.16829122	17.1459481	1.3613E-07	0.00352882	H3K27ac	LD
chr2	142760125	142760126	INS	chr2_143050210_143050566	2.16829122	17.1459481	1.3613E-07	0.00352882	H3K27ac	LD
chr2	142789505	142789506	INS	chr2_143050210_143050566	2.16829122	17.1459481	1.3613E-07	0.00352882	H3K27ac	LD
chr5	10731300	10731362	DEL	chr5_10849605_10850320	1.81660544	17.019655	1.4421E-07	0.00356499	H3K27ac	LD
chr5	11240434	11240561	DEL	chr5_10849605_10850320	1.81660544	17.019655	1.4421E-07	0.00356499	H3K27ac	LD
chr5	11244725	11244784	DEL	chr5_10849605_10850320	1.81660544	17.019655	1.4421E-07	0.00356499	H3K27ac	LD
chr4	14348040	14348321	DEL	chr4_14772731_14773580	7.0711835	17.0425578	1.4271E-07	0.00356499	H3K27ac	LD

chr4	14482085	14482401	DEL	chr4_14772731_14773580	7.0711835	17.0425578	1.4271E-07	0.00356499	H3K27ac	LD
chr4	14519833	14520121	DEL	chr4_14772731_14773580	7.0711835	17.0425578	1.4271E-07	0.00356499	H3K27ac	LD
chr4	14676696	14676807	DEL	chr4_14772731_14773580	7.0711835	17.0425578	1.4271E-07	0.00356499	H3K27ac	LD
chr4	15111522	15111752	DEL	chr4_14772731_14773580	7.0711835	17.0425578	1.4271E-07	0.00356499	H3K27ac	LD
chr5	10757999	10758000	INS	chr5_10849605_10850320	1.81660544	17.019655	1.4421E-07	0.00356499	H3K27ac	LD
chr4	14350891	14350892	INS	chr4_14772731_14773580	7.0711835	17.0425578	1.4271E-07	0.00356499	H3K27ac	LD
chr4	15108323	15108324	INS	chr4_14772731_14773580	7.0711835	17.0425578	1.4271E-07	0.00356499	H3K27ac	LD
chr4	94601354	94601685	DEL	chr4_94503441_94504545	3.00215488	16.9388298	1.4967E-07	0.00356744	H3K27ac	LD
chr14	37365520	37365786	DEL	chr14_36884906_36885323	1.52975061	16.947228	1.491E-07	0.00356744	H3K27ac	LD
chr4	94521048	94521049	INS	chr4_94503441_94504545	3.00215488	16.9388298	1.4967E-07	0.00356744	H3K27ac	LD
chr1	239084795	239084796	INS	chr1_239483661_239484025	4.68246889	16.9244355	1.5067E-07	0.00356744	H3K27ac	LD
chr16	6899596	6899597	INS	chr16_6822494_6823505	3.08819925	16.9108054	1.5162E-07	0.00356744	H3K27ac	LD
chr14	36645033	36645034	INS	chr14_36884906_36885323	1.52975061	16.947228	1.491E-07	0.00356744	H3K27ac	LD

chr14	37364956	37364957	INS	chr14_36884906_36885323	1.52975061	16.947228	1.491E-07	0.00356744	H3K27ac	LD
chr11	2277813	2278481	DEL	chr11_1949838_1950531	1.772107	16.8473251	1.5614E-07	0.00364081	H3K27ac	LD
chr1	127731232	127731233	INS	chr1_128202771_128204133	1.83220566	16.8408744	1.566E-07	0.00364081	H3K27ac	LD
chr11	2278490	2278491	INS	chr11_1949838_1950531	1.772107	16.8473251	1.5614E-07	0.00364081	H3K27ac	LD
chr10	32457875	32457876	INS	chr10_32694297_32694995	1.93575072	16.7745594	1.615E-07	0.00373978	H3K27ac	LD
chr8	6590538	6592307	DEL	chr8_6759286_6759545	1.44072406	16.6527253	1.7095E-07	0.00385199	H3K27ac	LD
chr8	7011822	7012705	DEL	chr8_6759286_6759545	1.44072406	16.6527253	1.7095E-07	0.00385199	H3K27ac	LD
chr8	6411586	6411587	INS	chr8_6759286_6759545	1.44072406	16.6527253	1.7095E-07	0.00385199	H3K27ac	LD
chr8	6550499	6550500	INS	chr8_6759286_6759545	1.44072406	16.6527253	1.7095E-07	0.00385199	H3K27ac	LD
chr8	6615803	6615804	INS	chr8_6759286_6759545	1.44072406	16.6527253	1.7095E-07	0.00385199	H3K27ac	LD
chr8	6622512	6622513	INS	chr8_6759286_6759545	1.44072406	16.6527253	1.7095E-07	0.00385199	H3K27ac	LD
chr8	6850546	6850547	INS	chr8_6759286_6759545	1.44072406	16.6527253	1.7095E-07	0.00385199	H3K27ac	LD
chr1	21122825	21122826	INS	chr1_21522228_21522558	-0.4823868	-16.612663	1.7419E-07	0.00390998	H3K27ac	LD

chr4	67006719	67006897	DEL	chr4_66985494_66985792	4.51756983	16.4431586	1.8868E-07	0.00412756	H3K27ac	LD
chr14	115562345	115562543	DEL	chr14_115187260_115188557	3.1916275	16.4338732	1.8952E-07	0.00412756	H3K27ac	LD
chr14	125120527	125120617	DEL	chr14_125136192_125136511	1.57618012	16.4726225	1.8607E-07	0.00412756	H3K27ac	LD
chr4	67009095	67009096	INS	chr4_66985494_66985792	4.51756983	16.4431586	1.8868E-07	0.00412756	H3K27ac	LD
chr14	115142175	115142176	INS	chr14_115187260_115188557	3.1916275	16.4338732	1.8952E-07	0.00412756	H3K27ac	LD
chr14	115398866	115398867	INS	chr14_115187260_115188557	3.1916275	16.4338732	1.8952E-07	0.00412756	H3K27ac	LD
chr14	125128665	125128666	INS	chr14_125136192_125136511	1.57618012	16.4726225	1.8607E-07	0.00412756	H3K27ac	LD
chr14	7440427	7440428	INS	chr14_7205953_7206502	9.27717	16.4787035	1.8554E-07	0.00412756	H3K27ac	LD
chr12	7979174	7979227	DEL	chr12_7924550_7925396	2.45671094	16.3641922	1.959E-07	0.00421945	H3K27ac	LD
chr12	8162476	8162529	DEL	chr12_7924550_7925396	2.45671094	16.3641922	1.959E-07	0.00421945	H3K27ac	LD
chr12	7896595	7896596	INS	chr12_7924550_7925396	2.45671094	16.3641922	1.959E-07	0.00421945	H3K27ac	LD
chr11	17440582	17440907	DEL	chr11_17081178_17081662	2.70403433	16.2449651	2.0738E-07	0.00438308	H3K27ac	LD
chr8	15493133	15493134	INS	chr8_15615311_15615914	1.6443598	16.2239673	2.0948E-07	0.00438308	H3K27ac	LD

chr8	15597660	15597661	INS	chr8_15615311_15615914	1.6443598	16.2239673	2.0948E-07	0.00438308	H3K27ac	LD
chr3	31124993	31124994	INS	chr3_30648472_30649340	2.44528013	16.1517402	2.1688E-07	0.00452188	H3K27ac	LD
chr11	47767910	47767911	INS	chr11_48183714_48185305	2.58972	16.0194541	2.3122E-07	0.00476989	H3K27ac	LD
chr11	48366990	48366991	INS	chr11_48183714_48185305	2.58972	16.0194541	2.3122E-07	0.00476989	H3K27ac	LD
chr11	48339259	48339260	INS	chr11_48183714_48185305	2.58972	16.0194541	2.3122E-07	0.00476989	H3K27ac	LD
chr11	9632489	9632564	DEL	chr11_9715886_9716269	3.45697144	15.8715207	2.4853E-07	0.00500359	H3K27ac	LD
chr11	10136750	10137029	DEL	chr11_9715886_9716269	3.45697144	15.8715207	2.4853E-07	0.00500359	H3K27ac	LD
chr11	9256939	9256940	INS	chr11_9715886_9716269	3.45697144	15.8715207	2.4853E-07	0.00500359	H3K27ac	LD
chr11	9634289	9634290	INS	chr11_9715886_9716269	3.45697144	15.8715207	2.4853E-07	0.00500359	H3K27ac	LD
chr11	9669823	9669824	INS	chr11_9715886_9716269	3.45697144	15.8715207	2.4853E-07	0.00500359	H3K27ac	LD
chr11	9940233	9940234	INS	chr11_9715886_9716269	3.45697144	15.8715207	2.4853E-07	0.00500359	H3K27ac	LD
chr11	9938346	9938347	INS	chr11_9715886_9716269	3.45697144	15.8715207	2.4853E-07	0.00500359	H3K27ac	LD
chr2	44976461	44976760	DEL	chr2_45155902_45157402	2.80523044	15.7964992	2.5786E-07	0.00512101	H3K27ac	LD

chr5	81070244	81070297	DEL	chr5_81227865_81228783	2.86291882	15.6618153	2.7561E-07	0.00541842	H3K27ac	LD
chr5	80955339	80955340	INS	chr5_81227865_81228783	2.86291882	15.6618153	2.7561E-07	0.00541842	H3K27ac	LD
chr5	81223761	81223762	INS	chr5_81227865_81228783	2.86291882	15.6618153	2.7561E-07	0.00541842	H3K27ac	LD
chr3	4370374	4370651	DEL	chr3_4558073_4559841	1.93137188	15.6410763	2.7846E-07	0.00544884	H3K27ac	LD
chr7	31382032	31382138	DEL	chr7_31229639_31229920	13.1092228	15.6237099	2.8087E-07	0.00544884	H3K27ac	LD
chr7	31405446	31407603	DEL	chr7_31229639_31229920	13.1092228	15.6237099	2.8087E-07	0.00544884	H3K27ac	LD
chr7	31549153	31549154	INS	chr7_31229639_31229920	13.1092228	15.6237099	2.8087E-07	0.00544884	H3K27ac	LD
chr18	34069885	34069935	DEL	chr18_34326468_34326874	4.19901975	15.6126235	2.8243E-07	0.0054609	H3K27ac	LD
chr5	64299488	64299489	INS	chr5_64708338_64708926	2.56496611	15.5958257	2.848E-07	0.00547066	H3K27ac	LD
chr15	135242606	135242607	INS	chr15_135585904_135586658	0.92976905	15.5698346	2.8852E-07	0.00552391	H3K27ac	LD
chr5	5099445	5099526	DEL	chr5_5088311_5089276	2.74428567	15.4931759	2.9979E-07	0.00572114	H3K27ac	LD
chr10	26869029	26875189	DEL	chr10_26931686_26932033	3.19964306	15.4542735	3.0571E-07	0.0057775	H3K27ac	LD
chr10	26882956	26882957	INS	chr10_26931686_26932033	3.19964306	15.4542735	3.0571E-07	0.0057775	H3K27ac	LD

chr10	27005863	27005864	INS	chr10_26931686_26932033	3.19964306	15.4542735	3.0571E-07	0.0057775	H3K27ac	LD
chr14	125145189	125145500	DEL	chr14_125176644_125177209	-4.5467637	-15.329218	3.2561E-07	0.00581602	H3K27ac	LD
chr3	11378193	11378455	DEL	chr3_11377751_11378152	2.65959902	15.3399908	3.2384E-07	0.00581602	H3K27ac	LD
chr16	48789493	48789560	DEL	chr16_48723716_48724651	10.9859494	15.3590111	3.2074E-07	0.00581602	H3K27ac	LD
chr14	125286621	125286622	INS	chr14_125176644_125177209	-4.5467637	-15.329218	3.2561E-07	0.00581602	H3K27ac	LD
chr14	125346834	125346835	INS	chr14_125176644_125177209	-4.5467637	-15.329218	3.2561E-07	0.00581602	H3K27ac	LD
chr14	125449978	125449979	INS	chr14_125176644_125177209	-4.5467637	-15.329218	3.2561E-07	0.00581602	H3K27ac	LD
chr14	125461758	125461759	INS	chr14_125176644_125177209	-4.5467637	-15.329218	3.2561E-07	0.00581602	H3K27ac	LD
chr14	125661993	125661994	INS	chr14_125176644_125177209	-4.5467637	-15.329218	3.2561E-07	0.00581602	H3K27ac	LD
chr16	48274765	48274766	INS	chr16_48723716_48724651	10.9859494	15.3590111	3.2074E-07	0.00581602	H3K27ac	LD
chr16	48295586	48295587	INS	chr16_48723716_48724651	10.9859494	15.3590111	3.2074E-07	0.00581602	H3K27ac	LD
chr16	48632665	48632666	INS	chr16_48723716_48724651	21.9718989	15.3590111	3.2074E-07	0.00581602	H3K27ac	LD
chr1	142151990	142152063	DEL	chr1_142412359_142413520	-2.6185723	-15.280706	3.3372E-07	0.00582076	H3K27ac	LD

chr17	4670603	4670798	DEL	chr17_5011012_5012416	2.01100556	15.2851192	3.3298E-07	0.00582076	H3K27ac	LD
chr17	4877572	4877764	DEL	chr17_5011012_5012416	2.01100556	15.2851192	3.3298E-07	0.00582076	H3K27ac	LD
chr17	4871260	4871548	DEL	chr17_5011012_5012416	2.01100556	15.2851192	3.3298E-07	0.00582076	H3K27ac	LD
chr2	25460761	25461018	DEL	chr2_25610349_25611358	1.49405406	15.2684197	3.3581E-07	0.00582076	H3K27ac	LD
chr2	25471776	25472926	DEL	chr2_25610349_25611358	1.49405406	15.2684197	3.3581E-07	0.00582076	H3K27ac	LD
chr17	51125293	51125294	INS	chr17_51168119_51168917	1.4916685	15.2700187	3.3554E-07	0.00582076	H3K27ac	LD
chr17	4813268	4813269	INS	chr17_5011012_5012416	2.01100556	15.2851192	3.3298E-07	0.00582076	H3K27ac	LD
chr17	5000144	5000145	INS	chr17_5011012_5012416	2.01100556	15.2851192	3.3298E-07	0.00582076	H3K27ac	LD
chr4	67430927	67431504	DEL	chr4_67231801_67232929	1.87796094	15.1407046	3.5843E-07	0.0060867	H3K27ac	LD
chr4	67441371	67442086	DEL	chr4_67231801_67232929	1.87796094	15.1407046	3.5843E-07	0.0060867	H3K27ac	LD
chr4	67352065	67352066	INS	chr4_67231801_67232929	1.87796094	15.1407046	3.5843E-07	0.0060867	H3K27ac	LD
chr4	67430930	67431664	INV	chr4_67231801_67232929	3.75592188	15.1407046	3.5843E-07	0.0060867	H3K27ac	LD
chr15	24523283	24523969	DEL	chr15_24931902_24932617	4.94846012	15.0407012	3.7733E-07	0.00628028	H3K27ac	LD

chr15	24999539	24999853	DEL	chr15_24931902_24932617	4.94846012	15.0407012	3.7733E-07	0.00628028	H3K27ac	LD
chr15	25078539	25078622	DEL	chr15_24931902_24932617	4.94846012	15.0407012	3.7733E-07	0.00628028	H3K27ac	LD
chr15	24622118	24622119	INS	chr15_24931902_24932617	9.89692024	15.0407012	3.7733E-07	0.00628028	H3K27ac	LD
chr15	25004746	25004747	INS	chr15_24931902_24932617	4.94846012	15.0407012	3.7733E-07	0.00628028	H3K27ac	LD
chr15	25110309	25110310	INS	chr15_24931902_24932617	4.94846012	15.0407012	3.7733E-07	0.00628028	H3K27ac	LD
chr15	25083729	25083730	INS	chr15_24931902_24932617	4.94846012	15.0407012	3.7733E-07	0.00628028	H3K27ac	LD
chr15	84696254	84696310	DEL	chr15_84718527_84719582	3.04296042	14.9951694	3.8631E-07	0.00628677	H3K27ac	LD
chr15	84754885	84754886	INS	chr15_84718527_84719582	3.04296042	14.9951694	3.8631E-07	0.00628677	H3K27ac	LD
chr15	85010449	85010450	INS	chr15_84718527_84719582	3.04296042	14.9951694	3.8631E-07	0.00628677	H3K27ac	LD
chr15	85018787	85018788	INS	chr15_84718527_84719582	3.04296042	14.9951694	3.8631E-07	0.00628677	H3K27ac	LD
chr3	92943265	92943345	DEL	chr3_93438075_93439074	3.01502761	14.9268191	4.0023E-07	0.00642918	H3K27ac	LD
chr3	92945049	92945231	DEL	chr3_93438075_93439074	3.01502761	14.9268191	4.0023E-07	0.00642918	H3K27ac	LD
chr3	93120380	93120381	INS	chr3_93438075_93439074	3.01502761	14.9268191	4.0023E-07	0.00642918	H3K27ac	LD

chr1	9608620	9608621	INS	chr1_9336111_9336861	1.53601525	14.9253164	4.0054E-07	0.00642918	H3K27ac	LD
chr10	58404235	58404236	INS	chr10_57922405_57922781	2.75446583	14.8880669	4.0838E-07	0.00653699	H3K27ac	LD
chr4	109047137	109047138	INS	chr4_109526558_109526826	2.27104833	14.7976565	4.2811E-07	0.00677879	H3K27ac	LD
chr4	109035259	109035260	INS	chr4_109526558_109526826	2.27104833	14.7976565	4.2811E-07	0.00677879	H3K27ac	LD
chr4	109061859	109061860	INS	chr4_109526558_109526826	2.27104833	14.7976565	4.2811E-07	0.00677879	H3K27ac	LD
chr4	109643075	109643076	INS	chr4_109526558_109526826	2.27104833	14.7976565	4.2811E-07	0.00677879	H3K27ac	LD
chr12	28200222	28200594	DEL	chr12_27988133_27988408	1.22015205	14.7295792	4.4368E-07	0.00700632	H3K27ac	LD
chr2	89638956	89639208	DEL	chr2_89746653_89747015	-1.6911848	-14.681542	4.5504E-07	0.00710915	H3K27ac	LD
chr2	89644034	89644362	DEL	chr2_89746653_89747015	1.69118483	14.6815416	4.5504E-07	0.00710915	H3K27ac	LD
chr7	14064483	14064548	DEL	chr7_14052362_14053317	2.23764494	14.6911626	4.5274E-07	0.00710915	H3K27ac	LD
chr7	13766495	13766496	INS	chr7_14052362_14053317	2.23764494	14.6911626	4.5274E-07	0.00710915	H3K27ac	LD
chr7	1748745	1748798	DEL	chr7_1579016_1579712	1.53460706	14.6405066	4.6501E-07	0.00711312	H3K27ac	LD
chr7	1929798	1929851	DEL	chr7_1579016_1579712	1.53460706	14.6405066	4.6501E-07	0.00711312	H3K27ac	LD

chr12	55037406	55037407	INS	chr12_55215069_55218039	-4.5888225	-14.659941	4.6026E-07	0.00711312	H3K27ac	LD
chr7	1523263	1523264	INS	chr7_1579016_1579712	1.53460706	14.6405066	4.6501E-07	0.00711312	H3K27ac	LD
chr7	1569174	1569175	INS	chr7_1579016_1579712	1.53460706	14.6405066	4.6501E-07	0.00711312	H3K27ac	LD
chr7	1811529	1811530	INS	chr7_1579016_1579712	1.53460706	14.6405066	4.6501E-07	0.00711312	H3K27ac	LD
chr7	1813759	1813760	INS	chr7_1579016_1579712	1.53460706	14.6405066	4.6501E-07	0.00711312	H3K27ac	LD
chr7	1949395	1949396	INS	chr7_1579016_1579712	1.53460706	14.6405066	4.6501E-07	0.00711312	H3K27ac	LD
chr16	74523566	74523662	DEL	chr16_74705152_74705550	8.356072	14.4531802	5.1373E-07	0.00718822	H3K27ac	LD
chr16	74810512	74810663	DEL	chr16_74705152_74705550	8.356072	14.4531802	5.1373E-07	0.00718822	H3K27ac	LD
chr13	116139907	116141499	DEL	chr13_115922759_115923077	2.21586019	14.5848981	4.7891E-07	0.00718822	H3K27ac	LD
chr13	189546379	189548076	DEL	chr13_189451395_189452168	1.82681933	14.5482049	4.8834E-07	0.00718822	H3K27ac	LD
chr5	10762104	10762578	DEL	chr5_10619114_10619873	1.20615813	14.5897519	4.7768E-07	0.00718822	H3K27ac	LD
chr16	47085242	47085536	DEL	chr16_47469085_47469466	2.58119756	14.5531284	4.8706E-07	0.00718822	H3K27ac	LD
chr16	54786690	54786780	DEL	chr16_54932108_54932375	1.93016784	14.4474234	5.1532E-07	0.00718822	H3K27ac	LD

chr16	54981565	54981875	DEL	chr16_54932108_54932375	1.93016784	14.4474234	5.1532E-07	0.00718822	H3K27ac	LD
chr16	74290155	74290156	INS	chr16_74705152_74705550	8.356072	14.4531802	5.1373E-07	0.00718822	H3K27ac	LD
chr16	74683234	74683235	INS	chr16_74705152_74705550	8.356072	14.4531802	5.1373E-07	0.00718822	H3K27ac	LD
chr16	75007017	75007018	INS	chr16_74705152_74705550	8.356072	14.4531802	5.1373E-07	0.00718822	H3K27ac	LD
chr13	188983904	188983905	INS	chr13_189451395_189452168	1.82681933	14.5482049	4.8834E-07	0.00718822	H3K27ac	LD
chr13	189143589	189143590	INS	chr13_189451395_189452168	1.82681933	14.5482049	4.8834E-07	0.00718822	H3K27ac	LD
chr13	189346991	189346992	INS	chr13_189451395_189452168	1.82681933	14.5482049	4.8834E-07	0.00718822	H3K27ac	LD
chr13	189680325	189680326	INS	chr13_189451395_189452168	1.82681933	14.5482049	4.8834E-07	0.00718822	H3K27ac	LD
chr13	189873403	189873404	INS	chr13_189451395_189452168	1.82681933	14.5482049	4.8834E-07	0.00718822	H3K27ac	LD
chr13	189922350	189922351	INS	chr13_189451395_189452168	3.65363867	14.5482049	4.8834E-07	0.00718822	H3K27ac	LD
chr2	834088	834089	INS	chr2_840199_841616	1.63582813	14.4692692	5.0933E-07	0.00718822	H3K27ac	LD
chr2	1053697	1053698	INS	chr2_840199_841616	1.63582813	14.4692692	5.0933E-07	0.00718822	H3K27ac	LD
chr2	1056976	1056977	INS	chr2_840199_841616	1.63582813	14.4692692	5.0933E-07	0.00718822	H3K27ac	LD

chr10	11698833	11698834	INS	chr10_11786783_11787216	1.67383794	14.5069184	4.9919E-07	0.00718822	H3K27ac	LD
chr10	11979215	11979216	INS	chr10_11786783_11787216	1.67383794	14.5069184	4.9919E-07	0.00718822	H3K27ac	LD
chr10	12030922	12030923	INS	chr10_11786783_11787216	1.67383794	14.5069184	4.9919E-07	0.00718822	H3K27ac	LD
chr10	12138054	12138055	INS	chr10_11786783_11787216	1.67383794	14.5069184	4.9919E-07	0.00718822	H3K27ac	LD
chr18	34696682	34696683	INS	chr18_34326468_34326874	4.18846456	14.503406	5.0013E-07	0.00718822	H3K27ac	LD
chr2	40784695	40784696	INS	chr2_41028383_41029590	2.23427111	14.5125984	4.9768E-07	0.00718822	H3K27ac	LD
chr16	47001071	47001072	INS	chr16_47469085_47469466	2.58119756	14.5531284	4.8706E-07	0.00718822	H3K27ac	LD
chr16	46999984	46999985	INS	chr16_47469085_47469466	2.58119756	14.5531284	4.8706E-07	0.00718822	H3K27ac	LD
chr16	47271630	47271631	INS	chr16_47469085_47469466	2.58119756	14.5531284	4.8706E-07	0.00718822	H3K27ac	LD
chr16	47282696	47282697	INS	chr16_47469085_47469466	2.58119756	14.5531284	4.8706E-07	0.00718822	H3K27ac	LD
chr16	55213946	55213947	INS	chr16_54932108_54932375	1.93016784	14.4474234	5.1532E-07	0.00718822	H3K27ac	LD
chr16	55265908	55265909	INS	chr16_54932108_54932375	1.93016784	14.4474234	5.1532E-07	0.00718822	H3K27ac	LD
chr16	55310724	55310725	INS	chr16_54932108_54932375	1.93016784	14.4474234	5.1532E-07	0.00718822	H3K27ac	LD

chr16	47000000	47000179	INV	chr16_47469085_47469466	2.58119756	14.5531284	4.8706E-07	0.00718822	H3K27ac	LD
chr11	2673974	2674042	DEL	chr11_2255707_2256779	2.965855	14.4275746	5.2082E-07	0.00723063	H3K27ac	LD
chr11	2693751	2693752	INS	chr11_2255707_2256779	2.965855	14.4275746	5.2082E-07	0.00723063	H3K27ac	LD
chr13	182332570	182332852	DEL	chr13_182328584_182329540	2.11627067	14.3688376	5.3751E-07	0.00739228	H3K27ac	LD
chr13	182330997	182331266	DEL	chr13_182328584_182329540	2.11627067	14.3688376	5.3751E-07	0.00739228	H3K27ac	LD
chr13	182586200	182587822	DEL	chr13_182328584_182329540	2.11627067	14.3688376	5.3751E-07	0.00739228	H3K27ac	LD
chr18	34069885	34069935	DEL	chr18_34325367_34326045	2.83613763	14.3633988	5.3909E-07	0.00739658	H3K27ac	LD
chr4	117014994	117015169	DEL	chr4_116921925_116922138	3.34337701	14.307749	5.5551E-07	0.00748091	H3K27ac	LD
chr4	117186122	117187428	DEL	chr4_116921925_116922138	3.34337701	14.307749	5.5551E-07	0.00748091	H3K27ac	LD
chr3	15621336	15621619	DEL	chr3_15191649_15192888	3.47555328	14.3145975	5.5346E-07	0.00748091	H3K27ac	LD
chr17	46835516	46835517	INS	chr17_47273496_47274654	1.838171	14.2994617	5.58E-07	0.00748091	H3K27ac	LD
chr17	47258838	47258839	INS	chr17_47273496_47274654	1.838171	14.2994617	5.58E-07	0.00748091	H3K27ac	LD
chr4	117126167	117126168	INS	chr4_116921925_116922138	3.34337701	14.307749	5.5551E-07	0.00748091	H3K27ac	LD
chr3	15115804	15115805	INS	chr3_15191649_15192888	3.47555328	14.3145975	5.5346E-07	0.00748091	H3K27ac	LD

chr3	15379269	15379270	INS	chr3_15191649_15192888	3.47555328	14.3145975	5.5346E-07	0.00748091	H3K27ac	LD
chr3	15599373	15599374	INS	chr3_15191649_15192888	3.47555328	14.3145975	5.5346E-07	0.00748091	H3K27ac	LD
chr1	216967651	216967651	BND	chr1_217214748_217214981	2.08563322	14.2590337	5.7035E-07	0.00750719	H3K27ac	LD
chr1	216967636	216967636	BND	chr1_217214748_217214981	2.08563322	14.2590337	5.7035E-07	0.00750719	H3K27ac	LD
chr1	216985279	216985397	DEL	chr1_217214748_217214981	2.08563322	14.2590337	5.7035E-07	0.00750719	H3K27ac	LD
chr1	6949592	6949593	INS	chr1_7434396_7434940	3.37379	14.2388569	5.7662E-07	0.00750719	H3K27ac	LD
chr3	442575	442576	INS	chr3_469415_471401	-2.1037524	-14.24304	5.7532E-07	0.00750719	H3K27ac	LD
chr3	471195	471196	INS	chr3_469415_471401	-2.1037524	-14.24304	5.7532E-07	0.00750719	H3K27ac	LD
chr1	216942632	216942633	INS	chr1_217214748_217214981	2.08563322	14.2590337	5.7035E-07	0.00750719	H3K27ac	LD
chr1	216963578	216963579	INS	chr1_217214748_217214981	2.08563322	14.2590337	5.7035E-07	0.00750719	H3K27ac	LD
chr1	216984174	216984175	INS	chr1_217214748_217214981	2.08563322	14.2590337	5.7035E-07	0.00750719	H3K27ac	LD
chr1	217009500	217009501	INS	chr1_217214748_217214981	2.08563322	14.2590337	5.7035E-07	0.00750719	H3K27ac	LD
chr1	217019959	217019960	INS	chr1_217214748_217214981	2.08563322	14.2590337	5.7035E-07	0.00750719	H3K27ac	LD

chr7	58330896	58331194	DEL	chr7_58712349_58713389	2.1784985	14.1501764	6.0513E-07	0.00775771	H3K27ac	LD
chr7	58583768	58584076	DEL	chr7_58712349_58713389	2.1784985	14.1501764	6.0513E-07	0.00775771	H3K27ac	LD
chr3	58398188	58398189	INS	chr3_58476418_58478422	4.19337125	14.1682839	5.9919E-07	0.00775771	H3K27ac	LD
chr3	58568874	58568875	INS	chr3_58476418_58478422	4.19337125	14.1682839	5.9919E-07	0.00775771	H3K27ac	LD
chr7	59023418	59023419	INS	chr7_58712349_58713389	2.1784985	14.1501764	6.0513E-07	0.00775771	H3K27ac	LD
chr7	59052293	59052294	INS	chr7_58712349_58713389	2.1784985	14.1501764	6.0513E-07	0.00775771	H3K27ac	LD
chr7	59193679	59193680	INS	chr7_58712349_58713389	2.1784985	14.1501764	6.0513E-07	0.00775771	H3K27ac	LD
chr15	24523283	24523969	DEL	chr15_24933593_24934000	2.49395674	14.1148614	6.1692E-07	0.00778955	H3K27ac	LD
chr15	24999539	24999853	DEL	chr15_24933593_24934000	2.49395674	14.1148614	6.1692E-07	0.00778955	H3K27ac	LD
chr15	25078539	25078622	DEL	chr15_24933593_24934000	2.49395674	14.1148614	6.1692E-07	0.00778955	H3K27ac	LD
chr15	24622118	24622119	INS	chr15_24933593_24934000	4.98791347	14.1148614	6.1692E-07	0.00778955	H3K27ac	LD
chr15	25004746	25004747	INS	chr15_24933593_24934000	2.49395674	14.1148614	6.1692E-07	0.00778955	H3K27ac	LD
chr15	25110309	25110310	INS	chr15_24933593_24934000	2.49395674	14.1148614	6.1692E-07	0.00778955	H3K27ac	LD

chr15	25083729	25083730	INS	chr15_24933593_24934000	2.49395674	14.1148614	6.1692E-07	0.00778955	H3K27ac	LD
chr2	3928648	3928814	DEL	chr2_4268879_4269091	1.55938939	14.0395338	6.4295E-07	0.00796363	H3K27ac	LD
chr2	3940139	3940433	DEL	chr2_4268879_4269091	1.55938939	14.0395338	6.4295E-07	0.00796363	H3K27ac	LD
chr3	14641313	14641370	DEL	chr3_15099247_15099527	4.76639767	14.0469516	6.4033E-07	0.00796363	H3K27ac	LD
chr3	14689089	14689376	DEL	chr3_15099247_15099527	4.76639767	14.0469516	6.4033E-07	0.00796363	H3K27ac	LD
chr3	15010037	15010323	DEL	chr3_15099247_15099527	4.76639767	14.0469516	6.4033E-07	0.00796363	H3K27ac	LD
chr3	15469022	15469137	DEL	chr3_15099247_15099527	4.76639767	14.0469516	6.4033E-07	0.00796363	H3K27ac	LD
chr3	14718791	14718792	INS	chr3_15099247_15099527	4.76639767	14.0469516	6.4033E-07	0.00796363	H3K27ac	LD
chr3	15494259	15494260	INS	chr3_15099247_15099527	4.76639767	14.0469516	6.4033E-07	0.00796363	H3K27ac	LD
chr1	20905538	20905598	DEL	chr1_20698296_20699103	2.07647688	13.9753561	6.6609E-07	0.00819826	H3K27ac	LD
chr1	21055246	21055396	DEL	chr1_20698296_20699103	2.07647688	13.9753561	6.6609E-07	0.00819826	H3K27ac	LD
chr1	21181171	21181249	DEL	chr1_20698296_20699103	2.07647688	13.9753561	6.6609E-07	0.00819826	H3K27ac	LD
chr1	187218142	187218143	INS	chr1_187485249_187485717	2.53562167	13.9494281	6.757E-07	0.00821305	H3K27ac	LD

chr1	187167328	187167329	INS	chr1_187485249_187485717	2.53562167	13.9494281	6.757E-07	0.00821305	H3K27ac	LD
chr1	187242929	187242930	INS	chr1_187485249_187485717	2.53562167	13.9494281	6.757E-07	0.00821305	H3K27ac	LD
chr1	187229573	187229574	INS	chr1_187485249_187485717	2.53562167	13.9494281	6.757E-07	0.00821305	H3K27ac	LD
chr8	3096324	3096325	INS	chr8_3245706_3245975	2.8446245	13.940261	6.7914E-07	0.00823772	H3K27ac	LD
chr11	72303060	72303178	DEL	chr11_72592429_72593268	1.86906322	13.7755384	7.4433E-07	0.00893605	H3K27ac	LD
chr11	72462361	72462460	DEL	chr11_72592429_72593268	1.86906322	13.7755384	7.4433E-07	0.00893605	H3K27ac	LD
chr14	88590639	88590640	INS	chr14_88587546_88587879	1.49925163	13.7771184	7.4368E-07	0.00893605	H3K27ac	LD
chr11	72317663	72317664	INS	chr11_72592429_72593268	1.86906322	13.7755384	7.4433E-07	0.00893605	H3K27ac	LD
chr15	30642959	30643060	DEL	chr15_30790841_30791321	1.31551444	13.7514182	7.5446E-07	0.00900224	H3K27ac	LD
chr15	30556894	30556895	INS	chr15_30790841_30791321	1.31551444	13.7514182	7.5446E-07	0.00900224	H3K27ac	LD
chr6	75388468	75388592	DEL	chr6_75137616_75138675	2.11759236	13.7387275	7.5984E-07	0.00901147	H3K27ac	LD
chr6	75495858	75496098	DEL	chr6_75137616_75138675	2.11759236	13.7387275	7.5984E-07	0.00901147	H3K27ac	LD
chr6	75216435	75216436	INS	chr6_75137616_75138675	2.11759236	13.7387275	7.5984E-07	0.00901147	H3K27ac	LD

chr14	88636891	88636892	INS	chr14_88683692_88684375	1.75863951	13.7319042	7.6276E-07	0.00902776	H3K27ac	LD
chr11	23634908	23634969	DEL	chr11_23985570_23986554	4.18532183	13.7162428	7.695E-07	0.00905264	H3K27ac	LD
chr11	23753392	23753393	INS	chr11_23985570_23986554	4.18532183	13.7162428	7.695E-07	0.00905264	H3K27ac	LD
chr11	24232286	24232287	INS	chr11_23985570_23986554	4.18532183	13.7162428	7.695E-07	0.00905264	H3K27ac	LD
chr8	110099584	110099916	DEL	chr8_110162060_110163195	4.64884789	13.6905035	7.8071E-07	0.00907527	H3K27ac	LD
chr8	110121017	110121085	DEL	chr8_110162060_110163195	4.64884789	13.6905035	7.8071E-07	0.00907527	H3K27ac	LD
chr8	110137183	110137476	DEL	chr8_110162060_110163195	4.64884789	13.6905035	7.8071E-07	0.00907527	H3K27ac	LD
chr8	5441995	5442279	DEL	chr8_5896674_5897344	1.69768589	13.7065455	7.737E-07	0.00907527	H3K27ac	LD
chr8	110102416	110102417	INS	chr8_110162060_110163195	4.64884789	13.6905035	7.8071E-07	0.00907527	H3K27ac	LD
chr8	110138787	110138788	INS	chr8_110162060_110163195	4.64884789	13.6905035	7.8071E-07	0.00907527	H3K27ac	LD
chr3	105893306	105893307	INS	chr3_106105716_106107413	0.97368681	13.6614581	7.9359E-07	0.00920673	H3K27ac	LD
chr15	119511808	119511859	DEL	chr15_119822535_119823098	1.74348331	13.6228987	8.1106E-07	0.00939078	H3K27ac	LD
chr9	129125776	129125830	DEL	chr9_129475893_129476304	1.5670541	13.5960186	8.2349E-07	0.00944143	H3K27ac	LD

chr2	13122273	13123683	DEL	chr2_13382603_13383898	14.8808043	13.5987307	8.2223E-07	0.00944143	H3K27ac	LD
chr9	129047885	129047886	INS	chr9_129475893_129476304	1.5670541	13.5960186	8.2349E-07	0.00944143	H3K27ac	LD
chr9	129079184	129079185	INS	chr9_129475893_129476304	1.5670541	13.5960186	8.2349E-07	0.00944143	H3K27ac	LD
chr9	129664547	129664548	INS	chr9_129475893_129476304	1.5670541	13.5960186	8.2349E-07	0.00944143	H3K27ac	LD
chr17	1011872	1011873	INS	chr17_1421580_1422050	2.261929	13.5796138	8.3119E-07	0.00949246	H3K27ac	LD
chr12	7979174	7979227	DEL	chr12_7945218_7945736	2.10826694	13.5637227	8.3871E-07	0.00952275	H3K27ac	LD
chr12	8162476	8162529	DEL	chr12_7945218_7945736	2.10826694	13.5637227	8.3871E-07	0.00952275	H3K27ac	LD
chr12	7896595	7896596	INS	chr12_7945218_7945736	2.10826694	13.5637227	8.3871E-07	0.00952275	H3K27ac	LD
chr18	33851164	33851165	INS	chr18_34311782_34312069	2.49952434	13.5387026	8.5072E-07	0.00956639	H3K27ac	LD
chr18	33948532	33948533	INS	chr18_34311782_34312069	2.49952434	13.5387026	8.5072E-07	0.00956639	H3K27ac	LD
chr18	33990090	33990091	INS	chr18_34311782_34312069	2.49952434	13.5387026	8.5072E-07	0.00956639	H3K27ac	LD
chr18	34103862	34103863	INS	chr18_34311782_34312069	2.49952434	13.5387026	8.5072E-07	0.00956639	H3K27ac	LD
chr18	34220782	34220783	INS	chr18_34311782_34312069	2.49952434	13.5387026	8.5072E-07	0.00956639	H3K27ac	LD

chr4	88317944	88318007	DEL	chr4_87923873_87924310	1.75819026	13.5264394	8.5668E-07	0.00959652	H3K27ac	LD
chr4	88337959	88338106	DEL	chr4_87923873_87924310	1.75819026	13.5264394	8.5668E-07	0.00959652	H3K27ac	LD
chr17	34591038	34592762	DEL	chr17_34825480_34826324	1.74289428	13.4084114	9.1645E-07	0.00981474	H3K27ac	LD
chr17	34915436	34915882	DEL	chr17_34825480_34826324	1.74289428	13.4084114	9.1645E-07	0.00981474	H3K27ac	LD
chr10	41963621	41964380	DEL	chr10_41815229_41816213	3.32899678	13.3913782	9.2545E-07	0.00981474	H3K27ac	LD
chr10	41995107	41995383	DEL	chr10_41815229_41816213	3.32899678	13.3913782	9.2545E-07	0.00981474	H3K27ac	LD
chr9	125280155	125280417	DEL	chr9_125544653_125545340	1.67148994	13.3491804	9.4819E-07	0.00981474	H3K27ac	LD
chr9	125584654	125584775	DEL	chr9_125544653_125545340	1.67148994	13.3491804	9.4819E-07	0.00981474	H3K27ac	LD
chr9	125789171	125789412	DEL	chr9_125544653_125545340	1.67148994	13.3491804	9.4819E-07	0.00981474	H3K27ac	LD
chr9	125836902	125837209	DEL	chr9_125544653_125545340	1.67148994	13.3491804	9.4819E-07	0.00981474	H3K27ac	LD
chr9	125942923	125943223	DEL	chr9_125544653_125545340	1.67148994	13.3491804	9.4819E-07	0.00981474	H3K27ac	LD
chr13	132036951	132037281	DEL	chr13_132445012_132446017	3.56103656	13.3507095	9.4736E-07	0.00981474	H3K27ac	LD
chr13	132066536	132066810	DEL	chr13_132445012_132446017	3.56103656	13.3507095	9.4736E-07	0.00981474	H3K27ac	LD

chr13	132535062	132535113	DEL	chr13_132445012_132446017	3.56103656	13.3507095	9.4736E-07	0.00981474	H3K27ac	LD
chr11	9967285	9967597	DEL	chr11_10317711_10318297	1.25418717	13.4119831	9.1457E-07	0.00981474	H3K27ac	LD
chr11	10416866	10417139	DEL	chr11_10317711_10318297	1.25418717	13.4119831	9.1457E-07	0.00981474	H3K27ac	LD
chr11	20834491	20834677	DEL	chr11_20766346_20767806	4.60334956	13.3888386	9.268E-07	0.00981474	H3K27ac	LD
chr11	20890182	20890256	DEL	chr11_20766346_20767806	4.60334956	13.3888386	9.268E-07	0.00981474	H3K27ac	LD
chr17	35145526	35145527	INS	chr17_34825480_34826324	1.74289428	13.4084114	9.1645E-07	0.00981474	H3K27ac	LD
chr10	42068222	42068223	INS	chr10_41815229_41816213	3.32899678	13.3913782	9.2545E-07	0.00981474	H3K27ac	LD
chr7	76590782	76590783	INS	chr7_76837928_76838581	3.26135641	13.3981268	9.2187E-07	0.00981474	H3K27ac	LD
chr7	76834294	76834295	INS	chr7_76837928_76838581	3.26135641	13.3981268	9.2187E-07	0.00981474	H3K27ac	LD
chr4	115398584	115398585	INS	chr4_115749480_115750289	3.48678668	13.3962883	9.2285E-07	0.00981474	H3K27ac	LD
chr4	115748918	115748919	INS	chr4_115749480_115750289	3.48678668	13.3962883	9.2285E-07	0.00981474	H3K27ac	LD
chr4	116067522	116067523	INS	chr4_115749480_115750289	3.48678668	13.3962883	9.2285E-07	0.00981474	H3K27ac	LD
chr9	125837762	125837763	INS	chr9_125544653_125545340	1.67148994	13.3491804	9.4819E-07	0.00981474	H3K27ac	LD

chr13	132054614	132054615	INS	chr13_132445012_132446017	3.56103656	13.3507095	9.4736E-07	0.00981474	H3K27ac	LD
chr11	9838526	9838527	INS	chr11_10317711_10318297	1.25418717	13.4119831	9.1457E-07	0.00981474	H3K27ac	LD
chr11	9914358	9914359	INS	chr11_10317711_10318297	1.25418717	13.4119831	9.1457E-07	0.00981474	H3K27ac	LD
chr10	6879148	6879149	INS	chr10_7262880_7263289	2.70818522	13.3523944	9.4644E-07	0.00981474	H3K27ac	LD
chr10	7207068	7207069	INS	chr10_7262880_7263289	2.70818522	13.3523944	9.4644E-07	0.00981474	H3K27ac	LD
chr10	7600673	7600674	INS	chr10_7262880_7263289	2.70818522	13.3523944	9.4644E-07	0.00981474	H3K27ac	LD
chr10	7629370	7629371	INS	chr10_7262880_7263289	2.70818522	13.3523944	9.4644E-07	0.00981474	H3K27ac	LD
chr10	7669859	7669860	INS	chr10_7262880_7263289	2.70818522	13.3523944	9.4644E-07	0.00981474	H3K27ac	LD
chr11	20793186	20793187	INS	chr11_20766346_20767806	4.60334956	13.3888386	9.268E-07	0.00981474	H3K27ac	LD
chr11	20806124	20806125	INS	chr11_20766346_20767806	4.60334956	13.3888386	9.268E-07	0.00981474	H3K27ac	LD
chr11	20980379	20980380	INS	chr11_20766346_20767806	4.60334956	13.3888386	9.268E-07	0.00981474	H3K27ac	LD
chr11	21120982	21120983	INS	chr11_20766346_20767806	4.60334956	13.3888386	9.268E-07	0.00981474	H3K27ac	LD
chr12	30983916	30983917	INS	chr12_31458504_31459093	1.1262795	13.4388949	9.0058E-07	0.00981474	H3K27ac	LD

chr15	58978965	58979020	DEL	chr15_58921700_58922333	3.42876174	13.324196	9.6195E-07	0.00988644	H3K27ac	LD
chr15	59000603	59000710	DEL	chr15_58921700_58922333	3.42876174	13.324196	9.6195E-07	0.00988644	H3K27ac	LD
chr15	59271408	59271409	INS	chr15_58921700_58922333	3.42876174	13.324196	9.6195E-07	0.00988644	H3K27ac	LD
chr14	125749696	125752831	DEL	chr14_125771862_125772898	5.10072061	13.2909429	9.8061E-07	0.00988825	H3K27ac	LD
chr14	125820966	125821859	DEL	chr14_125771862_125772898	5.10072061	13.2909429	9.8061E-07	0.00988825	H3K27ac	LD
chr14	126230173	126231813	DEL	chr14_125771862_125772898	5.10072061	13.2909429	9.8061E-07	0.00988825	H3K27ac	LD
chr14	125367373	125367374	INS	chr14_125771862_125772898	5.10072061	13.2909429	9.8061E-07	0.00988825	H3K27ac	LD
chr14	125535532	125535533	INS	chr14_125771862_125772898	5.10072061	13.2909429	9.8061E-07	0.00988825	H3K27ac	LD
chr14	125942900	125942901	INS	chr14_125771862_125772898	5.10072061	13.2909429	9.8061E-07	0.00988825	H3K27ac	LD
chr14	125949805	125949806	INS	chr14_125771862_125772898	5.10072061	13.2909429	9.8061E-07	0.00988825	H3K27ac	LD
chr14	126015483	126015484	INS	chr14_125771862_125772898	5.10072061	13.2909429	9.8061E-07	0.00988825	H3K27ac	LD
chr14	126098777	126098778	INS	chr14_125771862_125772898	5.10072061	13.2909429	9.8061E-07	0.00988825	H3K27ac	LD
NW_018085198.1	197171	197172	INS	NW_018085198.1_187081_187432	1.3697216	13.2773753	9.8834E-07	0.00991499	H3K27ac	LD

chr12	6462498	6462557	DEL	chr12_6714191_6717361	-6.0158563	-13.261291	9.9759E-07	0.0099907	H3K27ac	LD
chr7	2117751	2117809	DEL	chr7_2369205_2370407	2.42453817	13.2327916	1.0142E-06	0.01007119	H3K27ac	LD
chr7	2791373	2791620	DEL	chr7_2369205_2370407	2.42453817	13.2327916	1.0142E-06	0.01007119	H3K27ac	LD
chr7	2788509	2789192	DEL	chr7_2369205_2370407	2.42453817	13.2327916	1.0142E-06	0.01007119	H3K27ac	LD
chr7	2789599	2789874	DEL	chr7_2369205_2370407	2.42453817	13.2327916	1.0142E-06	0.01007119	H3K27ac	LD
chr7	2504066	2504067	INS	chr7_2369205_2370407	4.84907633	13.2327916	1.0142E-06	0.01007119	H3K27ac	LD
chr16	71784615	71784616	INS	chr16_71744709_71746050	3.518538	13.2261797	1.0181E-06	0.01009284	H3K27ac	LD
chr12	53685136	53685196	DEL	chr12_54033604_54034260	1.68176667	13.163598	1.0559E-06	0.01037962	H3K27ac	LD
chr10	1500716	1500824	DEL	chr10_1560783_1561197	2.45941775	13.1713213	1.0512E-06	0.01037962	H3K27ac	LD
chr12	54476335	54476336	INS	chr12_54033604_54034260	1.68176667	13.163598	1.0559E-06	0.01037962	H3K27ac	LD
chr9	6090430	6090503	DEL	chr9_6291389_6292761	2.14336188	13.137221	1.0723E-06	0.01038626	H3K27ac	LD
chr6	2026281	2026387	DEL	chr6_1869187_1871024	2.72218875	13.1467407	1.0664E-06	0.01038626	H3K27ac	LD
chr17	27720043	27720100	DEL	chr17_28095829_28096407	1.80867728	13.1171812	1.085E-06	0.01038626	H3K27ac	LD

chr17	27939863	27940054	DEL	chr17_28095829_28096407	1.80867728	13.1171812	1.085E-06	0.01038626	H3K27ac	LD
chr17	28088613	28088912	DEL	chr17_28095829_28096407	1.80867728	13.1171812	1.085E-06	0.01038626	H3K27ac	LD
chr17	28220512	28221747	DEL	chr17_28095829_28096407	1.80867728	13.1171812	1.085E-06	0.01038626	H3K27ac	LD
chr12	52376638	52376639	INS	chr12_52319003_52319428	1.61607839	13.1317366	1.0758E-06	0.01038626	H3K27ac	LD
chr12	52388734	52388735	INS	chr12_52319003_52319428	1.61607839	13.1317366	1.0758E-06	0.01038626	H3K27ac	LD
chr6	2100183	2100184	INS	chr6_1869187_1871024	2.72218875	13.1467407	1.0664E-06	0.01038626	H3K27ac	LD
chr17	27884515	27884516	INS	chr17_28095829_28096407	1.80867728	13.1171812	1.085E-06	0.01038626	H3K27ac	LD
chr17	27935599	27935600	INS	chr17_28095829_28096407	1.80867728	13.1171812	1.085E-06	0.01038626	H3K27ac	LD
chr17	28202530	28202531	INS	chr17_28095829_28096407	1.80867728	13.1171812	1.085E-06	0.01038626	H3K27ac	LD
chr17	28206265	28206266	INS	chr17_28095829_28096407	1.80867728	13.1171812	1.085E-06	0.01038626	H3K27ac	LD
chr17	28223429	28223430	INS	chr17_28095829_28096407	1.80867728	13.1171812	1.085E-06	0.01038626	H3K27ac	LD
chr17	28242286	28242287	INS	chr17_28095829_28096407	1.80867728	13.1171812	1.085E-06	0.01038626	H3K27ac	LD
chr18	10891516	10891754	DEL	chr18_10539306_10539654	4.26182872	13.0808553	1.1083E-06	0.01059251	H3K27ac	LD

chr15	96128036	96128354	DEL	chr15_95938037_95938739	1.23878013	13.0698958	1.1155E-06	0.01064353	H3K27ac	LD
chr6	34268109	34268110	INS	chr6_34518167_34518416	5.20833683	13.052761	1.1268E-06	0.01073379	H3K27ac	LD
chr5	17802326	17802327	INS	chr5_17450478_17451795	1.86389611	13.0419292	1.134E-06	0.01078488	H3K27ac	LD
chr16	68565023	68565278	DEL	chr16_68855371_68856371	0.97664188	12.9875298	1.1709E-06	0.0110825	H3K27ac	LD
chr16	68618958	68618959	INS	chr16_68855371_68856371	0.97664188	12.9875298	1.1709E-06	0.0110825	H3K27ac	LD
chr6	157567642	157567643	INS	chr6_157934483_157935633	6.36174944	12.9881465	1.1705E-06	0.0110825	H3K27ac	LD
chr9	119769210	119769279	DEL	chr9_119600576_119601637	1.67632097	12.9120332	1.2245E-06	0.01149657	H3K27ac	LD
chr9	119873028	119873122	DEL	chr9_119600576_119601637	1.67632097	12.9120332	1.2245E-06	0.01149657	H3K27ac	LD
chr9	119109160	119109161	INS	chr9_119600576_119601637	1.67632097	12.9120332	1.2245E-06	0.01149657	H3K27ac	LD
chr9	119479470	119479471	INS	chr9_119600576_119601637	1.67632097	12.9120332	1.2245E-06	0.01149657	H3K27ac	LD
chr9	119849464	119849465	INS	chr9_119600576_119601637	1.67632097	12.9120332	1.2245E-06	0.01149657	H3K27ac	LD
chr1	92555635	92555712	DEL	chr1_92351210_92352712	3.49190167	12.8644913	1.2596E-06	0.01150832	H3K27ac	LD
chr3	72340306	72342127	DEL	chr3_72716402_72717450	1.92696594	12.818941	1.2943E-06	0.01150832	H3K27ac	LD

chr2	126691403	126691567	DEL	chr2_126940177_126940729	1.49126944	12.8373101	1.2802E-06	0.01150832	H3K27ac	LD
chr2	141249576	141249649	DEL	chr2_141352405_141352878	4.33343944	12.8162187	1.2965E-06	0.01150832	H3K27ac	LD
chr2	141321676	141321776	DEL	chr2_141352405_141352878	4.33343944	12.8162187	1.2965E-06	0.01150832	H3K27ac	LD
chr2	141601296	141601910	DEL	chr2_141352405_141352878	4.33343944	12.8162187	1.2965E-06	0.01150832	H3K27ac	LD
chr1	219380135	219380437	DEL	chr1_219776847_219777794	1.04378611	12.8963135	1.236E-06	0.01150832	H3K27ac	LD
chr1	886373	886458	DEL	chr1_1166514_1167365	2.561447	12.8194092	1.294E-06	0.01150832	H3K27ac	LD
chr1	1172385	1172452	DEL	chr1_1166514_1167365	2.561447	12.8194092	1.294E-06	0.01150832	H3K27ac	LD
chr1	1302436	1302629	DEL	chr1_1166514_1167365	2.561447	12.8194092	1.294E-06	0.01150832	H3K27ac	LD
chr17	29879817	29880195	DEL	chr17_30328580_30328866	2.09465244	12.8860506	1.2436E-06	0.01150832	H3K27ac	LD
chr17	29969011	29969075	DEL	chr17_30328580_30328866	2.09465244	12.8860506	1.2436E-06	0.01150832	H3K27ac	LD
chr17	30263351	30263551	DEL	chr17_30328580_30328866	2.09465244	12.8860506	1.2436E-06	0.01150832	H3K27ac	LD
chr3	72405295	72405296	INS	chr3_72716402_72717450	1.92696594	12.818941	1.2943E-06	0.01150832	H3K27ac	LD
chr3	72515419	72515420	INS	chr3_72716402_72717450	1.92696594	12.818941	1.2943E-06	0.01150832	H3K27ac	LD

chr3	72668727	72668728	INS	chr3_72716402_72717450	1.92696594	12.818941	1.2943E-06	0.01150832	H3K27ac	LD
chr3	72699698	72699699	INS	chr3_72716402_72717450	1.92696594	12.818941	1.2943E-06	0.01150832	H3K27ac	LD
chr9	105600279	105600280	INS	chr9_105891012_105891696	1.1678305	12.8576889	1.2647E-06	0.01150832	H3K27ac	LD
chr9	106148475	106148476	INS	chr9_105891012_105891696	1.1678305	12.8576889	1.2647E-06	0.01150832	H3K27ac	LD
chr9	106287395	106287396	INS	chr9_105891012_105891696	1.1678305	12.8576889	1.2647E-06	0.01150832	H3K27ac	LD
chr8	130828410	130828411	INS	chr8_130823060_130823624	1.81749367	12.8408984	1.2775E-06	0.01150832	H3K27ac	LD
chr8	131006043	131006044	INS	chr8_130823060_130823624	1.81749367	12.8408984	1.2775E-06	0.01150832	H3K27ac	LD
chr8	131009776	131009777	INS	chr8_130823060_130823624	1.81749367	12.8408984	1.2775E-06	0.01150832	H3K27ac	LD
chr2	126770672	126770673	INS	chr2_126940177_126940729	1.49126944	12.8373101	1.2802E-06	0.01150832	H3K27ac	LD
chr2	127027611	127027612	INS	chr2_126940177_126940729	1.49126944	12.8373101	1.2802E-06	0.01150832	H3K27ac	LD
chr2	127207319	127207320	INS	chr2_126940177_126940729	1.49126944	12.8373101	1.2802E-06	0.01150832	H3K27ac	LD
chr2	141383621	141383622	INS	chr2_141352405_141352878	4.33343944	12.8162187	1.2965E-06	0.01150832	H3K27ac	LD
chr1	219548018	219548019	INS	chr1_219776847_219777794	1.04378611	12.8963135	1.236E-06	0.01150832	H3K27ac	LD

chr1	982463	982464	INS	chr1_1166514_1167365	2.561447	12.8194092	1.294E-06	0.01150832	H3K27ac	LD
chr1	1013579	1013580	INS	chr1_1166514_1167365	2.561447	12.8194092	1.294E-06	0.01150832	H3K27ac	LD
chr1	1290914	1290915	INS	chr1_1166514_1167365	2.561447	12.8194092	1.294E-06	0.01150832	H3K27ac	LD
chr5	18273921	18273922	INS	chr5_18141340_18142187	2.01591517	12.8424686	1.2763E-06	0.01150832	H3K27ac	LD
chr7	58330896	58331194	DEL	chr7_58584834_58585748	1.2607133	12.8009418	1.3084E-06	0.01152159	H3K27ac	LD
chr7	58583768	58584076	DEL	chr7_58584834_58585748	1.2607133	12.8009418	1.3084E-06	0.01152159	H3K27ac	LD
chr7	58129054	58129055	INS	chr7_58584834_58585748	1.2607133	12.8009418	1.3084E-06	0.01152159	H3K27ac	LD
chr7	59023418	59023419	INS	chr7_58584834_58585748	1.2607133	12.8009418	1.3084E-06	0.01152159	H3K27ac	LD
chr7	59052293	59052294	INS	chr7_58584834_58585748	1.2607133	12.8009418	1.3084E-06	0.01152159	H3K27ac	LD
chr12	53685136	53685196	DEL	chr12_53884719_53885330	2.44925039	12.7788327	1.3258E-06	0.01162796	H3K27ac	LD
chr13	206353933	206353934	INS	chr13_206397942_206398653	8.76633444	12.7759169	1.3281E-06	0.0116309	H3K27ac	LD
chr18	2641232	2641233	INS	chr18_2614256_2615010	2.03068488	12.7539064	1.3458E-06	0.011768	H3K27ac	LD
chr13	151215608	151215954	DEL	chr13_150993460_150993795	2.34347458	12.6782402	1.4086E-06	0.01226193	H3K27ac	LD

chr13	150789823	150789824	INS	chr13_150993460_150993795	2.34347458	12.6782402	1.4086E-06	0.01226193	H3K27ac	LD
chr13	151313128	151313129	INS	chr13_150993460_150993795	2.34347458	12.6782402	1.4086E-06	0.01226193	H3K27ac	LD
chr3	126194038	126194103	DEL	chr3_126398451_126400623	6.84306537	12.6736312	1.4125E-06	0.01227787	H3K27ac	LD
chr9	48385337	48385338	INS	chr9_48502693_48503121	1.33570611	12.6192448	1.4598E-06	0.01265135	H3K27ac	LD
chr9	48331266	48331267	INS	chr9_48502693_48503121	1.33570611	12.6192448	1.4598E-06	0.01265135	H3K27ac	LD
chr2	114552256	114552257	INS	chr2_114565269_114565911	2.2499275	12.6049686	1.4725E-06	0.0127426	H3K27ac	LD
chr12	6036037	6036110	DEL	chr12_5731654_5732797	2.12610833	12.5707414	1.5034E-06	0.01287741	H3K27ac	LD
chr12	5241797	5241798	INS	chr12_5731654_5732797	2.12610833	12.5707414	1.5034E-06	0.01287741	H3K27ac	LD
chr12	5276005	5276006	INS	chr12_5731654_5732797	2.12610833	12.5707414	1.5034E-06	0.01287741	H3K27ac	LD
chr12	5276914	5276915	INS	chr12_5731654_5732797	2.12610833	12.5707414	1.5034E-06	0.01287741	H3K27ac	LD
chr12	5477121	5477122	INS	chr12_5731654_5732797	2.12610833	12.5707414	1.5034E-06	0.01287741	H3K27ac	LD
chr13	115611953	115611954	INS	chr13_115922759_115923077	2.18850389	12.5765821	1.4981E-06	0.01287741	H3K27ac	LD
chr13	116380029	116380030	INS	chr13_115922759_115923077	2.18850389	12.5765821	1.4981E-06	0.01287741	H3K27ac	LD

chr12	43929416	43929729	DEL	chr12_44027751_44028103	1.34895613	12.5663919	1.5074E-06	0.01289271	H3K27ac	LD
chr15	4903241	4905069	DEL	chr15_4485136_4485589	1.52403481	12.557942	1.5152E-06	0.01292152	H3K27ac	LD
chr7	66387015	66387090	DEL	chr7_66384244_66384720	3.613033	12.5465898	1.5257E-06	0.01297348	H3K27ac	LD
chr7	66694642	66694831	DEL	chr7_66384244_66384720	3.613033	12.5465898	1.5257E-06	0.01297348	H3K27ac	LD
chr14	11134315	11134397	DEL	chr14_11478414_11479002	1.60274372	12.513766	1.5566E-06	0.01314075	H3K27ac	LD
chr14	11125158	11126042	DEL	chr14_11478414_11479002	1.60274372	12.513766	1.5566E-06	0.01314075	H3K27ac	LD
chr14	11242835	11243130	DEL	chr14_11478414_11479002	1.60274372	12.513766	1.5566E-06	0.01314075	H3K27ac	LD
chr14	11990550	11990759	DEL	chr14_11478414_11479002	1.60274372	12.513766	1.5566E-06	0.01314075	H3K27ac	LD
chr14	11244352	11244353	INS	chr14_11478414_11479002	1.60274372	12.513766	1.5566E-06	0.01314075	H3K27ac	LD
chr1	215020902	215021225	DEL	chr1_215404079_215405141	1.41325617	12.5012774	1.5686E-06	0.01318065	H3K27ac	LD
chr15	2611505	2611613	DEL	chr15_2594365_2595432	2.40124219	12.4947374	1.5748E-06	0.01318065	H3K27ac	LD
chr17	14055601	14055844	DEL	chr17_13726591_13727144	0.95736712	12.5007307	1.5691E-06	0.01318065	H3K27ac	LD
chr7	3776714	3776715	INS	chr7_4132649_4132900	1.71079736	12.4965934	1.5731E-06	0.01318065	H3K27ac	LD

chr7	3801047	3801048	INS	chr7_4132649_4132900	1.71079736	12.4965934	1.5731E-06	0.01318065	H3K27ac	LD
chr7	3837785	3837786	INS	chr7_4132649_4132900	1.71079736	12.4965934	1.5731E-06	0.01318065	H3K27ac	LD
chr12	3547922	3549334	DEL	chr12_3399639_3401140	5.71615222	12.4845763	1.5847E-06	0.01320633	H3K27ac	LD
chr12	3329084	3329085	INS	chr12_3399639_3401140	2.85807611	12.4845763	1.5847E-06	0.01320633	H3K27ac	LD
chr12	3384719	3384720	INS	chr12_3399639_3401140	2.85807611	12.4845763	1.5847E-06	0.01320633	H3K27ac	LD
chr9	28219462	28219523	DEL	chr9_28546302_28546848	1.61865833	12.4696641	1.5992E-06	0.0132148	H3K27ac	LD
chr9	28230497	28230565	DEL	chr9_28546302_28546848	1.61865833	12.4696641	1.5992E-06	0.0132148	H3K27ac	LD
chr9	28073753	28073754	INS	chr9_28546302_28546848	1.61865833	12.4696641	1.5992E-06	0.0132148	H3K27ac	LD
chr9	28054498	28054499	INS	chr9_28546302_28546848	1.61865833	12.4696641	1.5992E-06	0.0132148	H3K27ac	LD
chr9	28502390	28502391	INS	chr9_28546302_28546848	1.61865833	12.4696641	1.5992E-06	0.0132148	H3K27ac	LD
chr9	28919619	28919620	INS	chr9_28546302_28546848	1.61865833	12.4696641	1.5992E-06	0.0132148	H3K27ac	LD
chr15	96128036	96128354	DEL	chr15_96035048_96035785	0.87838575	12.454077	1.6146E-06	0.01332302	H3K27ac	LD
chr16	71784615	71784616	INS	chr16_71746684_71747249	4.10684231	12.4336391	1.635E-06	0.0134724	H3K27ac	LD

chr12	54857433	54857714	DEL	chr12_55209441_55210267	6.012902	12.3976812	1.6716E-06	0.01365874	H3K27ac	LD
chr12	55102291	55102670	DEL	chr12_55209441_55210267	6.012902	12.3976812	1.6716E-06	0.01365874	H3K27ac	LD
chr12	55111149	55111479	DEL	chr12_55209441_55210267	6.012902	12.3976812	1.6716E-06	0.01365874	H3K27ac	LD
chr12	55570531	55570840	DEL	chr12_55209441_55210267	-6.012902	-12.397681	1.6716E-06	0.01365874	H3K27ac	LD
chr12	55139758	55139759	INS	chr12_55209441_55210267	6.012902	12.3976812	1.6716E-06	0.01365874	H3K27ac	LD
chr12	55199996	55199997	INS	chr12_55209441_55210267	6.012902	12.3976812	1.6716E-06	0.01365874	H3K27ac	LD
chr1	10190799	10190800	INS	chr1_9847446_9848614	1.69785944	12.3602768	1.7106E-06	0.01395836	H3K27ac	LD
chr5	28872208	28872280	DEL	chr5_28538399_28539486	3.01686714	12.3579171	1.7131E-06	0.0139593	H3K27ac	LD
chr11	23747292	23747293	INS	chr11_23483525_23484473	2.62161555	12.3522765	1.7191E-06	0.01398862	H3K27ac	LD
chr4	111441903	111442229	DEL	chr4_111826864_111827082	3.31423611	12.3109097	1.7638E-06	0.01402071	H3K27ac	LD
chr4	111440474	111440855	DEL	chr4_111826864_111827082	3.31423611	12.3109097	1.7638E-06	0.01402071	H3K27ac	LD
chr4	111455017	111455259	DEL	chr4_111826864_111827082	3.31423611	12.3109097	1.7638E-06	0.01402071	H3K27ac	LD
chr4	111475575	111475859	DEL	chr4_111826864_111827082	3.31423611	12.3109097	1.7638E-06	0.01402071	H3K27ac	LD

chr4	111513800	111514088	DEL	chr4_111826864_111827082	3.31423611	12.3109097	1.7638E-06	0.01402071	H3K27ac	LD
chr4	111524360	111524672	DEL	chr4_111826864_111827082	3.31423611	12.3109097	1.7638E-06	0.01402071	H3K27ac	LD
chr4	111574757	111575066	DEL	chr4_111826864_111827082	3.31423611	12.3109097	1.7638E-06	0.01402071	H3K27ac	LD
chr17	47404088	47404089	INS	chr17_47482022_47482166	-2.028293	-12.314054	1.7603E-06	0.01402071	H3K27ac	LD
chr4	111451939	111451940	INS	chr4_111826864_111827082	3.31423611	12.3109097	1.7638E-06	0.01402071	H3K27ac	LD
chr4	111508830	111508831	INS	chr4_111826864_111827082	3.31423611	12.3109097	1.7638E-06	0.01402071	H3K27ac	LD
chr4	111821059	111821060	INS	chr4_111826864_111827082	3.31423611	12.3109097	1.7638E-06	0.01402071	H3K27ac	LD
chr4	112256584	112256585	INS	chr4_111826864_111827082	3.31423611	12.3109097	1.7638E-06	0.01402071	H3K27ac	LD
chr1	14779865	14779866	INS	chr1_14806919_14807230	1.989445	12.3185355	1.7554E-06	0.01402071	H3K27ac	LD
chr1	15269290	15269291	INS	chr1_14806919_14807230	1.989445	12.3185355	1.7554E-06	0.01402071	H3K27ac	LD
chr6	20415488	20415489	INS	chr6_20296471_20297988	-2.4822049	-12.343722	1.7283E-06	0.01402071	H3K27ac	LD
chr4	36831205	36831394	DEL	chr4_36710023_36710843	1.10681736	12.2563047	1.8247E-06	0.01434913	H3K27ac	LD
chr16	66236976	66236977	INS	chr16_66065363_66065692	0.94501414	12.2585598	1.8221E-06	0.01434913	H3K27ac	LD

chr4	36372060	36372061	INS	chr4_36710023_36710843	1.10681736	12.2563047	1.8247E-06	0.01434913	H3K27ac	LD
chr4	36731812	36731813	INS	chr4_36710023_36710843	1.10681736	12.2563047	1.8247E-06	0.01434913	H3K27ac	LD
chr4	36791518	36791519	INS	chr4_36710023_36710843	1.10681736	12.2563047	1.8247E-06	0.01434913	H3K27ac	LD
chr4	36935001	36935002	INS	chr4_36710023_36710843	1.10681736	12.2563047	1.8247E-06	0.01434913	H3K27ac	LD
chr4	36946085	36946086	INS	chr4_36710023_36710843	1.10681736	12.2563047	1.8247E-06	0.01434913	H3K27ac	LD
chr16	53709679	53709680	INS	chr16_53792371_53793203	3.73671144	12.2183554	1.8684E-06	0.01467321	H3K27ac	LD
chr13	180241586	180241587	INS	chr13_180207601_180208739	5.47735833	12.2051859	1.8838E-06	0.01475494	H3K27ac	LD
chr13	180415133	180415134	INS	chr13_180207601_180208739	5.47735833	12.2051859	1.8838E-06	0.01475494	H3K27ac	LD
chr7	50865308	50865372	DEL	chr7_51156073_51156853	2.00315786	12.1962573	1.8944E-06	0.01477831	H3K27ac	LD
chr7	50863408	50863562	DEL	chr7_51156073_51156853	2.00315786	12.1962573	1.8944E-06	0.01477831	H3K27ac	LD
chr7	51284613	51284664	DEL	chr7_51156073_51156853	2.00315786	12.1962573	1.8944E-06	0.01477831	H3K27ac	LD
chr11	67775143	67775447	DEL	chr11_67947411_67950093	-4.652568	-12.170277	1.9255E-06	0.0149412	H3K27ac	LD
chr11	67909408	67909409	INS	chr11_67947411_67950093	-4.652568	-12.170277	1.9255E-06	0.0149412	H3K27ac	LD

chr11	68004883	68004884	INS	chr11_67947411_67950093	-4.652568	-12.170277	1.9255E-06	0.0149412	H3K27ac	LD
NW_018084937.1	28498	29545	DEL	chr5_63771231_63772948	2.7229	12.1156414	1.9927E-06	0.01508682	H3K27ac	LD
chr1	207204292	207204347	DEL	chr1_207581150_207581985	3.52853403	12.1385908	1.9641E-06	0.01508682	H3K27ac	LD
chr1	207901544	207901687	DEL	chr1_207581150_207581985	3.52853403	12.1385908	1.9641E-06	0.01508682	H3K27ac	LD
chr1	886373	886458	DEL	chr1_1172498_1173690	5.29592317	12.1152812	1.9932E-06	0.01508682	H3K27ac	LD
chr1	1172385	1172452	DEL	chr1_1172498_1173690	5.29592317	12.1152812	1.9932E-06	0.01508682	H3K27ac	LD
chr1	1302436	1302629	DEL	chr1_1172498_1173690	5.29592317	12.1152812	1.9932E-06	0.01508682	H3K27ac	LD
chr9	49603919	49604484	DEL	chr9_49271859_49272298	1.72854939	12.1294362	1.9755E-06	0.01508682	H3K27ac	LD
chr1	207274400	207274401	INS	chr1_207581150_207581985	3.52853403	12.1385908	1.9641E-06	0.01508682	H3K27ac	LD
chr1	207426007	207426008	INS	chr1_207581150_207581985	3.52853403	12.1385908	1.9641E-06	0.01508682	H3K27ac	LD
chr1	207485699	207485700	INS	chr1_207581150_207581985	3.52853403	12.1385908	1.9641E-06	0.01508682	H3K27ac	LD
chr1	207565295	207565296	INS	chr1_207581150_207581985	3.52853403	12.1385908	1.9641E-06	0.01508682	H3K27ac	LD
chr1	982463	982464	INS	chr1_1172498_1173690	5.29592317	12.1152812	1.9932E-06	0.01508682	H3K27ac	LD

chr1	1013579	1013580	INS	chr1_1172498_1173690	5.29592317	12.1152812	1.9932E-06	0.01508682	H3K27ac	LD
chr1	1290914	1290915	INS	chr1_1172498_1173690	5.29592317	12.1152812	1.9932E-06	0.01508682	H3K27ac	LD
chr9	49726106	49726107	INS	chr9_49271859_49272298	1.72854939	12.1294362	1.9755E-06	0.01508682	H3K27ac	LD
chr17	21498766	21498767	INS	chr17_21625204_21626206	0.91206963	12.098898	2.0138E-06	0.01522366	H3K27ac	LD
chr8	56358452	56358453	INS	chr8_55963693_55963971	2.37120278	12.0858279	2.0305E-06	0.01529048	H3K27ac	LD
chr8	56380864	56380865	INS	chr8_55963693_55963971	2.37120278	12.0858279	2.0305E-06	0.01529048	H3K27ac	LD
chr8	56441303	56441304	INS	chr8_55963693_55963971	2.37120278	12.0858279	2.0305E-06	0.01529048	H3K27ac	LD
chr3	19371667	19373346	DEL	chr3_19414639_19415095	2.01834763	12.0547121	2.0708E-06	0.01543529	H3K27ac	LD
chr3	19428165	19428403	DEL	chr3_19414639_19415095	2.01834763	12.0547121	2.0708E-06	0.01543529	H3K27ac	LD
chr3	19525043	19525113	DEL	chr3_19414639_19415095	2.01834763	12.0547121	2.0708E-06	0.01543529	H3K27ac	LD
chr3	19844376	19846158	DEL	chr3_19414639_19415095	2.01834763	12.0547121	2.0708E-06	0.01543529	H3K27ac	LD
chr3	19852323	19853848	DEL	chr3_19414639_19415095	2.01834763	12.0547121	2.0708E-06	0.01543529	H3K27ac	LD
chr3	19847896	19852299	DEL	chr3_19414639_19415095	2.01834763	12.0547121	2.0708E-06	0.01543529	H3K27ac	LD

chr3	19443399	19443400	INS	chr3_19414639_19415095	2.01834763	12.0547121	2.0708E-06	0.01543529	H3K27ac	LD
chr3	19830871	19830872	INS	chr3_19414639_19415095	2.01834763	12.0547121	2.0708E-06	0.01543529	H3K27ac	LD
chr1	195531349	195531350	INS	chr1_196024908_196025211	1.89914944	12.0499866	2.077E-06	0.0154422	H3K27ac	LD
chr1	195994817	195994818	INS	chr1_196024908_196025211	1.89914944	12.0499866	2.077E-06	0.0154422	H3K27ac	LD
chr9	7913352	7913353	INS	chr9_8385994_8386207	7.17418063	12.0448773	2.0837E-06	0.01547257	H3K27ac	LD
chr15	32494876	32494877	INS	chr15_32768847_32769875	1.19542222	12.035581	2.096E-06	0.01552456	H3K27ac	LD
chr15	32657325	32657326	INS	chr15_32768847_32769875	1.19542222	12.035581	2.096E-06	0.01552456	H3K27ac	LD
chr8	74819683	74819985	DEL	chr8_75248354_75249095	1.90736156	12.0127163	2.1266E-06	0.01557401	H3K27ac	LD
chr8	75220455	75220537	DEL	chr8_75248354_75249095	1.90736156	12.0127163	2.1266E-06	0.01557401	H3K27ac	LD
chr8	75638466	75638558	DEL	chr8_75248354_75249095	1.90736156	12.0127163	2.1266E-06	0.01557401	H3K27ac	LD
chr8	75747445	75747582	DEL	chr8_75248354_75249095	1.90736156	12.0127163	2.1266E-06	0.01557401	H3K27ac	LD
chr8	75160841	75160842	INS	chr8_75248354_75249095	1.90736156	12.0127163	2.1266E-06	0.01557401	H3K27ac	LD
chr8	75404636	75404637	INS	chr8_75248354_75249095	1.90736156	12.0127163	2.1266E-06	0.01557401	H3K27ac	LD

chr8	75397932	75397933	INS	chr8_75248354_75249095	1.90736156	12.0127163	2.1266E-06	0.01557401	H3K27ac	LD
chr8	75703189	75703190	INS	chr8_75248354_75249095	1.90736156	12.0127163	2.1266E-06	0.01557401	H3K27ac	LD
chr8	75725415	75725416	INS	chr8_75248354_75249095	1.90736156	12.0127163	2.1266E-06	0.01557401	H3K27ac	LD
chr3	3637794	3637861	DEL	chr3_3530708_3530968	2.579925	11.9748379	2.1784E-06	0.01571891	H3K27ac	LD
chr13	10725409	10731409	DEL	chr13_10586223_10587607	2.78260381	11.9747073	2.1786E-06	0.01571891	H3K27ac	LD
chr13	10813053	10813336	DEL	chr13_10586223_10587607	2.78260381	11.9747073	2.1786E-06	0.01571891	H3K27ac	LD
chr13	10820210	10820368	DEL	chr13_10586223_10587607	2.78260381	11.9747073	2.1786E-06	0.01571891	H3K27ac	LD
chr13	10172354	10172355	INS	chr13_10586223_10587607	2.78260381	11.9747073	2.1786E-06	0.01571891	H3K27ac	LD
chr13	10344533	10344534	INS	chr13_10586223_10587607	2.78260381	11.9747073	2.1786E-06	0.01571891	H3K27ac	LD
chr13	10578694	10578695	INS	chr13_10586223_10587607	2.78260381	11.9747073	2.1786E-06	0.01571891	H3K27ac	LD
chr13	10709196	10709197	INS	chr13_10586223_10587607	2.78260381	11.9747073	2.1786E-06	0.01571891	H3K27ac	LD
chr13	10830239	10830240	INS	chr13_10586223_10587607	2.78260381	11.9747073	2.1786E-06	0.01571891	H3K27ac	LD
chr13	10825319	10825320	INS	chr13_10586223_10587607	2.78260381	11.9747073	2.1786E-06	0.01571891	H3K27ac	LD

chr8	3603610	3603759	DEL	chr8_3777219_3777652	2.21545188	11.9635522	2.1941E-06	0.01579202	H3K27ac	LD
chr8	3263315	3263316	INS	chr8_3777219_3777652	2.21545188	11.9635522	2.1941E-06	0.01579202	H3K27ac	LD
chr7	114967669	114967846	DEL	chr7_115356273_115357947	2.66429278	11.937976	2.2302E-06	0.0158184	H3K27ac	LD
chr7	115538968	115539244	DEL	chr7_115356273_115357947	2.66429278	11.937976	2.2302E-06	0.0158184	H3K27ac	LD
chr7	2711131	2711378	DEL	chr7_2735086_2736332	2.14740794	11.9412184	2.2256E-06	0.0158184	H3K27ac	LD
chr7	3168350	3168981	DEL	chr7_2735086_2736332	2.14740794	11.9412184	2.2256E-06	0.0158184	H3K27ac	LD
chr7	3226757	3226758	INS	chr7_2735086_2736332	2.14740794	11.9412184	2.2256E-06	0.0158184	H3K27ac	LD
chr7	115107150	115107151	INS	chr7_115356273_115357947	2.66429278	11.937976	2.2302E-06	0.0158184	H3K27ac	LD
chr7	115285940	115285941	INS	chr7_115356273_115357947	2.66429278	11.937976	2.2302E-06	0.0158184	H3K27ac	LD
chr7	115489273	115489274	INS	chr7_115356273_115357947	2.66429278	11.937976	2.2302E-06	0.0158184	H3K27ac	LD
chr7	2449976	2449977	INS	chr7_2735086_2736332	2.14740794	11.9412184	2.2256E-06	0.0158184	H3K27ac	LD
chr7	2675915	2675916	INS	chr7_2735086_2736332	2.14740794	11.9412184	2.2256E-06	0.0158184	H3K27ac	LD
chr7	2689666	2689667	INS	chr7_2735086_2736332	2.14740794	11.9412184	2.2256E-06	0.0158184	H3K27ac	LD

chr7	2691233	2691234	INS	chr7_2735086_2736332	2.14740794	11.9412184	2.2256E-06	0.0158184	H3K27ac	LD
chr9	33837090	33837090	BND	chr9_34022143_34022479	1.40108239	11.9103726	2.2699E-06	0.01596447	H3K27ac	LD
chr9	33526849	33527066	DEL	chr9_34022143_34022479	1.40108239	11.9103726	2.2699E-06	0.01596447	H3K27ac	LD
chr9	33684729	33685050	DEL	chr9_34022143_34022479	1.40108239	11.9103726	2.2699E-06	0.01596447	H3K27ac	LD
chr9	33557955	33557956	INS	chr9_34022143_34022479	1.40108239	11.9103726	2.2699E-06	0.01596447	H3K27ac	LD
chr9	33850463	33850464	INS	chr9_34022143_34022479	1.40108239	11.9103726	2.2699E-06	0.01596447	H3K27ac	LD
chr9	33837071	33837072	INS	chr9_34022143_34022479	1.40108239	11.9103726	2.2699E-06	0.01596447	H3K27ac	LD
chr9	34100911	34100912	INS	chr9_34022143_34022479	1.40108239	11.9103726	2.2699E-06	0.01596447	H3K27ac	LD
chr14	78570952	78571034	DEL	chr14_78612168_78612645	1.05878257	11.904951	2.2778E-06	0.0160007	H3K27ac	LD
chr12	59679085	59679086	INS	chr12_59448829_59449504	1.02252519	11.902297	2.2816E-06	0.0160087	H3K27ac	LD
chr2	5817838	5817839	INS	chr2_5880633_5882714	2.8698431	11.8959092	2.291E-06	0.01605504	H3K27ac	LD
NW_018084968.1	746147	746148	INS	NW_018084968.1_612320_613280	1.27809238	11.8753549	2.3213E-06	0.01622901	H3K27ac	LD
NW_018084968.1	257909	257910	INS	NW_018084968.1_612320_613280	1.27809238	11.8753549	2.3213E-06	0.01622901	H3K27ac	LD

chr14	13913272	13913554	DEL	chr14_13883261_13883661	1.302781	11.8635794	2.3389E-06	0.01631311	H3K27ac	LD
chr14	13525256	13525257	INS	chr14_13883261_13883661	1.302781	11.8635794	2.3389E-06	0.01631311	H3K27ac	LD
chr14	58052902	58052950	DEL	chr14_58467355_58468256	4.92613674	11.8597738	2.3447E-06	0.01631416	H3K27ac	LD
chr14	58454749	58454750	INS	chr14_58467355_58468256	4.92613674	11.8597738	2.3447E-06	0.01631416	H3K27ac	LD
chr7	18308073	18308163	DEL	chr7_18491247_18491681	1.54572417	11.8391095	2.376E-06	0.01634544	H3K27ac	LD
chr7	18319529	18319691	DEL	chr7_18491247_18491681	1.54572417	11.8391095	2.376E-06	0.01634544	H3K27ac	LD
chr7	18550275	18550411	DEL	chr7_18491247_18491681	1.54572417	11.8391095	2.376E-06	0.01634544	H3K27ac	LD
chr17	10875809	10876185	DEL	chr17_10650910_10651347	1.56491264	11.8329455	2.3854E-06	0.01634544	H3K27ac	LD
chr4	105698990	105698991	INS	chr4_106021571_106021813	1.90350778	11.8442808	2.3681E-06	0.01634544	H3K27ac	LD
chr4	105882402	105882403	INS	chr4_106021571_106021813	1.90350778	11.8442808	2.3681E-06	0.01634544	H3K27ac	LD
chr7	18028398	18028399	INS	chr7_18491247_18491681	1.54572417	11.8391095	2.376E-06	0.01634544	H3K27ac	LD
chr7	18269194	18269195	INS	chr7_18491247_18491681	1.54572417	11.8391095	2.376E-06	0.01634544	H3K27ac	LD
chr7	18550631	18550632	INS	chr7_18491247_18491681	1.54572417	11.8391095	2.376E-06	0.01634544	H3K27ac	LD

chr7	18924781	18924782	INS	chr7_18491247_18491681	1.54572417	11.8391095	2.376E-06	0.01634544	H3K27ac	LD
chr17	10152382	10152383	INS	chr17_10650910_10651347	1.56491264	11.8329455	2.3854E-06	0.01634544	H3K27ac	LD
chr17	10532876	10532877	INS	chr17_10650910_10651347	1.56491264	11.8329455	2.3854E-06	0.01634544	H3K27ac	LD
chr17	10827591	10827592	INS	chr17_10650910_10651347	1.56491264	11.8329455	2.3854E-06	0.01634544	H3K27ac	LD
chr12	23061742	23061800	DEL	chr12_23239883_23240602	4.96434375	11.8165112	2.4108E-06	0.01639272	H3K27ac	LD
chr12	23165657	23165954	DEL	chr12_23239883_23240602	4.96434375	11.8165112	2.4108E-06	0.01639272	H3K27ac	LD
chr9	41449637	41449712	DEL	chr9_41799862_41800535	3.97119222	11.8139881	2.4147E-06	0.01639272	H3K27ac	LD
chr9	42227260	42227548	DEL	chr9_41799862_41800535	3.97119222	11.8139881	2.4147E-06	0.01639272	H3K27ac	LD
chr12	23054397	23054398	INS	chr12_23239883_23240602	4.96434375	11.8165112	2.4108E-06	0.01639272	H3K27ac	LD
chr9	41669652	41669653	INS	chr9_41799862_41800535	3.97119222	11.8139881	2.4147E-06	0.01639272	H3K27ac	LD
chr9	42088027	42088028	INS	chr9_41799862_41800535	3.97119222	11.8139881	2.4147E-06	0.01639272	H3K27ac	LD
chr9	42255940	42255941	INS	chr9_41799862_41800535	3.97119222	11.8139881	2.4147E-06	0.01639272	H3K27ac	LD
chr7	65405657	65405729	DEL	chr7_65645765_65646933	2.54734342	11.7740779	2.4777E-06	0.01675585	H3K27ac	LD

chr7	65648286	65648589	DEL	chr7_65645765_65646933	2.54734342	11.7740779	2.4777E-06	0.01675585	H3K27ac	LD
chr7	65853868	65853869	INS	chr7_65645765_65646933	2.54734342	11.7740779	2.4777E-06	0.01675585	H3K27ac	LD
chr3	96447744	96447745	INS	chr3_96340371_96340770	0.98250269	11.7728564	2.4796E-06	0.01675585	H3K27ac	LD
chr15	39186278	39186367	DEL	chr15_38789933_38792084	2.90650625	11.7416137	2.5303E-06	0.01699991	H3K27ac	LD
chr15	39262089	39262390	DEL	chr15_38789933_38792084	5.8130125	11.7416137	2.5303E-06	0.01699991	H3K27ac	LD
chr15	38493706	38493707	INS	chr15_38789933_38792084	2.90650625	11.7416137	2.5303E-06	0.01699991	H3K27ac	LD
chr15	39010272	39010273	INS	chr15_38789933_38792084	2.90650625	11.7416137	2.5303E-06	0.01699991	H3K27ac	LD
chr15	39049411	39049412	INS	chr15_38789933_38792084	2.90650625	11.7416137	2.5303E-06	0.01699991	H3K27ac	LD
chr5	85274611	85274727	DEL	chr5_85108083_85109405	6.87662756	11.7254604	2.5569E-06	0.01706141	H3K27ac	LD
chr5	85296090	85296218	DEL	chr5_85108083_85109405	6.87662756	11.7254604	2.5569E-06	0.01706141	H3K27ac	LD
chr10	61779310	61779311	INS	chr10_61708540_61709660	1.7894275	11.7284792	2.5519E-06	0.01706141	H3K27ac	LD
chr5	84827328	84827329	INS	chr5_85108083_85109405	6.87662756	11.7254604	2.5569E-06	0.01706141	H3K27ac	LD
chr5	85207330	85207331	INS	chr5_85108083_85109405	6.87662756	11.7254604	2.5569E-06	0.01706141	H3K27ac	LD

chr5	85259779	85259780	INS	chr5_85108083_85109405	6.87662756	11.7254604	2.5569E-06	0.01706141	H3K27ac	LD
chr4	61881845	61881846	INS	chr4_61780323_61781226	3.98534478	11.7153998	2.5736E-06	0.01711465	H3K27ac	LD
chr4	61936751	61936752	INS	chr4_61780323_61781226	3.98534478	11.7153998	2.5736E-06	0.01711465	H3K27ac	LD
chr4	61975829	61975830	INS	chr4_61780323_61781226	3.98534478	11.7153998	2.5736E-06	0.01711465	H3K27ac	LD
chr13	194980929	194980984	DEL	chr13_194838105_194838421	1.20511817	11.6854752	2.6242E-06	0.0172742	H3K27ac	LD
chr13	195027685	195027968	DEL	chr13_194838105_194838421	1.20511817	11.6854752	2.6242E-06	0.0172742	H3K27ac	LD
chr13	195022724	195023105	DEL	chr13_194838105_194838421	1.20511817	11.6854752	2.6242E-06	0.0172742	H3K27ac	LD
chr13	194390440	194390441	INS	chr13_194838105_194838421	1.20511817	11.6854752	2.6242E-06	0.0172742	H3K27ac	LD
chr13	194410999	194411000	INS	chr13_194838105_194838421	1.20511817	11.6854752	2.6242E-06	0.0172742	H3K27ac	LD
chr13	194608290	194608291	INS	chr13_194838105_194838421	1.20511817	11.6854752	2.6242E-06	0.0172742	H3K27ac	LD
chr13	195028720	195028721	INS	chr13_194838105_194838421	1.20511817	11.6854752	2.6242E-06	0.0172742	H3K27ac	LD
chr13	195162856	195162857	INS	chr13_194838105_194838421	1.20511817	11.6854752	2.6242E-06	0.0172742	H3K27ac	LD
chr13	195274387	195274388	INS	chr13_194838105_194838421	1.20511817	11.6854752	2.6242E-06	0.0172742	H3K27ac	LD

chr15	55615201	55615439	DEL	chr15_55991649_55992123	4.20967444	11.6065905	2.7628E-06	0.01792474	H3K27ac	LD
chr15	4658867	4658949	DEL	chr15_4485136_4485589	1.50842261	11.6101203	2.7564E-06	0.01792474	H3K27ac	LD
chr15	55675502	55675503	INS	chr15_55991649_55992123	4.20967444	11.6065905	2.7628E-06	0.01792474	H3K27ac	LD
chr15	55718732	55718733	INS	chr15_55991649_55992123	4.20967444	11.6065905	2.7628E-06	0.01792474	H3K27ac	LD
chr15	55705087	55705088	INS	chr15_55991649_55992123	4.20967444	11.6065905	2.7628E-06	0.01792474	H3K27ac	LD
chr15	55896369	55896370	INS	chr15_55991649_55992123	4.20967444	11.6065905	2.7628E-06	0.01792474	H3K27ac	LD
chr2	118112356	118112357	INS	chr2_117677828_117678098	3.41368167	11.6192379	2.74E-06	0.01792474	H3K27ac	LD
chr15	4819492	4819493	INS	chr15_4485136_4485589	1.50842261	11.6101203	2.7564E-06	0.01792474	H3K27ac	LD
chr15	4856174	4856175	INS	chr15_4485136_4485589	1.50842261	11.6101203	2.7564E-06	0.01792474	H3K27ac	LD
chr15	4871621	4871622	INS	chr15_4485136_4485589	1.50842261	11.6101203	2.7564E-06	0.01792474	H3K27ac	LD
chr7	11254222	11254223	INS	chr7_11615847_11616828	5.95031333	11.6110168	2.7548E-06	0.01792474	H3K27ac	LD
chr6	42800274	42800275	INS	chr6_42802501_42803183	1.71203594	11.6148953	2.7478E-06	0.01792474	H3K27ac	LD
chr1	96621352	96621662	DEL	chr1_96566339_96567337	2.10077063	11.5644229	2.8402E-06	0.01824528	H3K27ac	LD

chr7	76590782	76590783	INS	chr7_76832442_76833936	2.03854472	11.5662754	2.8367E-06	0.01824528	H3K27ac	LD
chr7	76834294	76834295	INS	chr7_76832442_76833936	2.03854472	11.5662754	2.8367E-06	0.01824528	H3K27ac	LD
chr1	96580710	96580711	INS	chr1_96566339_96567337	2.10077063	11.5644229	2.8402E-06	0.01824528	H3K27ac	LD
chr1	96606317	96606318	INS	chr1_96566339_96567337	2.10077063	11.5644229	2.8402E-06	0.01824528	H3K27ac	LD
chr1	96658311	96658312	INS	chr1_96566339_96567337	2.10077063	11.5644229	2.8402E-06	0.01824528	H3K27ac	LD
chr1	96661658	96661659	INS	chr1_96566339_96567337	2.10077063	11.5644229	2.8402E-06	0.01824528	H3K27ac	LD
chr18	34696682	34696683	INS	chr18_34311782_34312069	2.49589863	11.5698812	2.83E-06	0.01824528	H3K27ac	LD
chr3	125236510	125237128	DEL	chr3_125216864_125217498	1.85903083	11.5389229	2.8882E-06	0.01835916	H3K27ac	LD
chr3	125534678	125534905	DEL	chr3_125216864_125217498	1.85903083	11.5389229	2.8882E-06	0.01835916	H3K27ac	LD
chr3	124835193	124835194	INS	chr3_125216864_125217498	1.85903083	11.5389229	2.8882E-06	0.01835916	H3K27ac	LD
chr3	125038991	125038992	INS	chr3_125216864_125217498	1.85903083	11.5389229	2.8882E-06	0.01835916	H3K27ac	LD
chr3	125045994	125045995	INS	chr3_125216864_125217498	1.85903083	11.5389229	2.8882E-06	0.01835916	H3K27ac	LD
chr18	25537822	25537823	INS	chr18_25712644_25712892	1.94621139	11.5383646	2.8893E-06	0.01835916	H3K27ac	LD

chr18	25671236	25671237	INS	chr18_25712644_25712892	1.94621139	11.5383646	2.8893E-06	0.01835916	H3K27ac	LD
chr18	25673649	25673650	INS	chr18_25712644_25712892	1.94621139	11.5383646	2.8893E-06	0.01835916	H3K27ac	LD
chr18	26211189	26211190	INS	chr18_25712644_25712892	1.94621139	11.5383646	2.8893E-06	0.01835916	H3K27ac	LD
chr7	4760866	4760937	DEL	chr7_4481677_4482191	5.89887333	11.5192713	2.9258E-06	0.01849114	H3K27ac	LD
chr7	4182425	4182426	INS	chr7_4481677_4482191	5.89887333	11.5192713	2.9258E-06	0.01849114	H3K27ac	LD
chr7	4450972	4450973	INS	chr7_4481677_4482191	5.89887333	11.5192713	2.9258E-06	0.01849114	H3K27ac	LD
chr7	4749568	4749569	INS	chr7_4481677_4482191	5.89887333	11.5192713	2.9258E-06	0.01849114	H3K27ac	LD
chr7	4843286	4843287	INS	chr7_4481677_4482191	5.89887333	11.5192713	2.9258E-06	0.01849114	H3K27ac	LD
chr1	180343252	180343334	DEL	chr1_179847723_179847936	3.05798539	11.517337	2.9295E-06	0.01849477	H3K27ac	LD
chr4	110150133	110150134	INS	chr4_110497559_110498348	3.58902639	11.5005034	2.9622E-06	0.01864096	H3K27ac	LD
chr4	110850791	110850792	INS	chr4_110497559_110498348	3.58902639	11.5005034	2.9622E-06	0.01864096	H3K27ac	LD
chr2	140753430	140753794	DEL	chr2_140936989_140937799	2.93405251	11.4898865	2.9831E-06	0.0186718	H3K27ac	LD
chr2	140904741	140904791	DEL	chr2_140936989_140937799	2.93405251	11.4898865	2.9831E-06	0.0186718	H3K27ac	LD

chr2	140512036	140512037	INS	chr2_140936989_140937799	2.93405251	11.4898865	2.9831E-06	0.0186718	H3K27ac	LD
chr18	8118179	8118180	INS	chr18_8480431_8480709	1.30457017	11.4822759	2.9981E-06	0.01874588	H3K27ac	LD
chr1	20603079	20603080	INS	chr1_21090980_21091343	2.03824208	11.4721755	3.0182E-06	0.01879125	H3K27ac	LD
chr5	79364639	79364640	INS	chr5_79840277_79840680	1.42407361	11.4656012	3.0313E-06	0.01885312	H3K27ac	LD
chr2	12789310	12789615	DEL	chr2_13025518_13026636	5.60408958	11.4489274	3.065E-06	0.01902202	H3K27ac	LD
chr2	12747947	12747948	INS	chr2_13025518_13026636	5.60408958	11.4489274	3.065E-06	0.01902202	H3K27ac	LD
chr12	59606113	59606287	DEL	chr12_60042781_60044571	2.36776361	11.4203877	3.1236E-06	0.01915889	H3K27ac	LD
chr15	84696254	84696310	DEL	chr15_84727782_84729284	3.38163889	11.4131799	3.1386E-06	0.01915889	H3K27ac	LD
chr1	6592901	6593070	DEL	chr1_6415780_6416164	1.18203125	11.4191125	3.1262E-06	0.01915889	H3K27ac	LD
chr17	19088317	19088675	DEL	chr17_19441902_19442118	2.10759493	11.4065333	3.1525E-06	0.01915889	H3K27ac	LD
chr17	19253258	19253310	DEL	chr17_19441902_19442118	2.10759493	11.4065333	3.1525E-06	0.01915889	H3K27ac	LD
chr17	19661088	19661373	DEL	chr17_19441902_19442118	2.10759493	11.4065333	3.1525E-06	0.01915889	H3K27ac	LD
chr12	59801168	59801169	INS	chr12_60042781_60044571	2.36776361	11.4203877	3.1236E-06	0.01915889	H3K27ac	LD

chr12	60251195	60251196	INS	chr12_60042781_60044571	2.36776361	11.4203877	3.1236E-06	0.01915889	H3K27ac	LD
chr15	84754885	84754886	INS	chr15_84727782_84729284	3.38163889	11.4131799	3.1386E-06	0.01915889	H3K27ac	LD
chr15	85010449	85010450	INS	chr15_84727782_84729284	3.38163889	11.4131799	3.1386E-06	0.01915889	H3K27ac	LD
chr15	85018787	85018788	INS	chr15_84727782_84729284	3.38163889	11.4131799	3.1386E-06	0.01915889	H3K27ac	LD
chr1	6741584	6741585	INS	chr1_6415780_6416164	1.18203125	11.4191125	3.1262E-06	0.01915889	H3K27ac	LD
chr1	6770469	6770470	INS	chr1_6415780_6416164	1.18203125	11.4191125	3.1262E-06	0.01915889	H3K27ac	LD
chr17	18942470	18942471	INS	chr17_19441902_19442118	2.10759493	11.4065333	3.1525E-06	0.01915889	H3K27ac	LD
chr17	18962708	18962709	INS	chr17_19441902_19442118	2.10759493	11.4065333	3.1525E-06	0.01915889	H3K27ac	LD
chr14	88590639	88590640	INS	chr14_88683692_88684375	1.75347164	11.3880682	3.1914E-06	0.01937558	H3K27ac	LD
chr14	115562345	115562543	DEL	chr14_115247071_115247376	3.62210069	11.370835	3.2283E-06	0.01937841	H3K27ac	LD
chr2	104267619	104268726	DEL	chr2_103771675_103772539	2.94190794	11.3719849	3.2258E-06	0.01937841	H3K27ac	LD
chr5	92100018	92100334	DEL	chr5_91832899_91833409	1.62316061	11.3715095	3.2268E-06	0.01937841	H3K27ac	LD
chr14	115142175	115142176	INS	chr14_115247071_115247376	3.62210069	11.370835	3.2283E-06	0.01937841	H3K27ac	LD

chr14	115398866	115398867	INS	chr14_115247071_115247376	3.62210069	11.370835	3.2283E-06	0.01937841	H3K27ac	LD
chr14	115735538	115735539	INS	chr14_115247071_115247376	3.62210069	11.370835	3.2283E-06	0.01937841	H3K27ac	LD
chr2	103438650	103438651	INS	chr2_103771675_103772539	2.94190794	11.3719849	3.2258E-06	0.01937841	H3K27ac	LD
chr2	103924277	103924278	INS	chr2_103771675_103772539	2.94190794	11.3719849	3.2258E-06	0.01937841	H3K27ac	LD
chr2	104125001	104125002	INS	chr2_103771675_103772539	2.94190794	11.3719849	3.2258E-06	0.01937841	H3K27ac	LD
chr5	91835969	91835970	INS	chr5_91832899_91833409	1.62316061	11.3715095	3.2268E-06	0.01937841	H3K27ac	LD
chr5	91932889	91932890	INS	chr5_91832899_91833409	1.62316061	11.3715095	3.2268E-06	0.01937841	H3K27ac	LD
chr14	89723334	89723446	DEL	chr14_89700036_89700265	1.86174756	11.367577	3.2353E-06	0.01940067	H3K27ac	LD
chr13	79451200	79451201	INS	chr13_79316483_79316902	1.62661091	11.3633581	3.2444E-06	0.01943544	H3K27ac	LD
chr1	259902147	259902203	DEL	chr1_260096335_260096579	1.45933576	11.358415	3.2551E-06	0.01947972	H3K27ac	LD
chr4	124372035	124372036	INS	chr4_124307842_124308589	4.55038813	11.3426272	3.2896E-06	0.01964601	H3K27ac	LD
chr15	134149340	134149410	DEL	chr15_134555736_134556101	3.2835	11.3247869	3.3291E-06	0.0197535	H3K27ac	LD
chr7	111524207	111524498	DEL	chr7_111820856_111821187	1.4861035	11.3160232	3.3487E-06	0.0197535	H3K27ac	LD

chr7	111839272	111840072	DEL	chr7_111820856_111821187	1.4861035	11.3160232	3.3487E-06	0.0197535	H3K27ac	LD
chr7	111994681	111995773	DEL	chr7_111820856_111821187	1.4861035	11.3160232	3.3487E-06	0.0197535	H3K27ac	LD
chr7	112222236	112222670	DEL	chr7_111820856_111821187	1.4861035	11.3160232	3.3487E-06	0.0197535	H3K27ac	LD
chr1	10984687	10984748	DEL	chr1_10927518_10927906	2.92724042	11.3132849	3.3548E-06	0.0197535	H3K27ac	LD
chr15	134336590	134336591	INS	chr15_134555736_134556101	1.64175	11.3247869	3.3291E-06	0.0197535	H3K27ac	LD
chr15	134532036	134532037	INS	chr15_134555736_134556101	1.64175	11.3247869	3.3291E-06	0.0197535	H3K27ac	LD
chr7	111605619	111605620	INS	chr7_111820856_111821187	1.4861035	11.3160232	3.3487E-06	0.0197535	H3K27ac	LD
chr7	111879070	111879071	INS	chr7_111820856_111821187	1.4861035	11.3160232	3.3487E-06	0.0197535	H3K27ac	LD
chr7	111985757	111985758	INS	chr7_111820856_111821187	1.4861035	11.3160232	3.3487E-06	0.0197535	H3K27ac	LD
chr7	112221707	112221708	INS	chr7_111820856_111821187	1.4861035	11.3160232	3.3487E-06	0.0197535	H3K27ac	LD
chr1	11012921	11012922	INS	chr1_10927518_10927906	2.92724042	11.3132849	3.3548E-06	0.0197535	H3K27ac	LD
chr1	11077077	11077078	INS	chr1_10927518_10927906	2.92724042	11.3132849	3.3548E-06	0.0197535	H3K27ac	LD
chr6	9694079	9694376	DEL	chr6_9460477_9461109	3.45174872	11.2548507	3.489E-06	0.02050244	H3K27ac	LD

chr6	9821185	9821186	INS	chr6_9460477_9461109	6.90349744	11.2548507	3.489E-06	0.02050244	H3K27ac	LD
chr3	11378193	11378455	DEL	chr3_11378488_11379270	2.844926	11.2473802	3.5066E-06	0.02058513	H3K27ac	LD
chr9	11987399	11987400	INS	chr9_11741966_11742269	1.27958993	11.2407725	3.5222E-06	0.02061495	H3K27ac	LD
chr9	12086715	12086716	INS	chr9_11741966_11742269	1.27958993	11.2407725	3.5222E-06	0.02061495	H3K27ac	LD
chr9	12106144	12106145	INS	chr9_11741966_11742269	1.27958993	11.2407725	3.5222E-06	0.02061495	H3K27ac	LD
chr9	63760656	63760798	DEL	chr9_64179114_64179970	2.02506111	11.2340146	3.5383E-06	0.02064714	H3K27ac	LD
chr9	64490860	64490946	DEL	chr9_64179114_64179970	2.02506111	11.2340146	3.5383E-06	0.02064714	H3K27ac	LD
chr9	64449324	64449325	INS	chr9_64179114_64179970	2.02506111	11.2340146	3.5383E-06	0.02064714	H3K27ac	LD
chr15	30642959	30643060	DEL	chr15_30795509_30796018	2.71387646	11.227558	3.5537E-06	0.02067542	H3K27ac	LD
chr15	30556894	30556895	INS	chr15_30795509_30796018	2.71387646	11.227558	3.5537E-06	0.02067542	H3K27ac	LD
chr5	5099445	5099526	DEL	chr5_5090321_5091319	1.85687792	11.2098724	3.5964E-06	0.0207652	H3K27ac	LD
chr6	19898577	19898647	DEL	chr6_19784943_19785432	1.9547024	11.2064323	3.6048E-06	0.0207652	H3K27ac	LD
chr1	37835585	37837012	DEL	chr1_37841881_37842551	1.54962769	11.2153849	3.583E-06	0.0207652	H3K27ac	LD

chr8	3603610	3603759	DEL	chr8_3770961_3771357	5.85203944	11.2050375	3.6082E-06	0.0207652	H3K27ac	LD
chr2	86747882	86747883	INS	chr2_87210232_87212048	1.764895	11.2117899	3.5917E-06	0.0207652	H3K27ac	LD
chr6	19299914	19299915	INS	chr6_19784943_19785432	1.9547024	11.2064323	3.6048E-06	0.0207652	H3K27ac	LD
chr6	19379500	19379501	INS	chr6_19784943_19785432	1.9547024	11.2064323	3.6048E-06	0.0207652	H3K27ac	LD
chr6	19716835	19716836	INS	chr6_19784943_19785432	1.9547024	11.2064323	3.6048E-06	0.0207652	H3K27ac	LD
chr1	37705345	37705346	INS	chr1_37841881_37842551	1.54962769	11.2153849	3.583E-06	0.0207652	H3K27ac	LD
chr1	37857767	37857768	INS	chr1_37841881_37842551	1.54962769	11.2153849	3.583E-06	0.0207652	H3K27ac	LD
chr8	3263315	3263316	INS	chr8_3770961_3771357	5.85203944	11.2050375	3.6082E-06	0.0207652	H3K27ac	LD
chr5	80241318	80241389	DEL	chr5_80634194_80634519	-1.7325965	-11.196517	3.629E-06	0.02080334	H3K27ac	LD
chr5	80874654	80874655	INS	chr5_80634194_80634519	-1.7325965	-11.196517	3.629E-06	0.02080334	H3K27ac	LD
chr5	80938515	80938516	INS	chr5_80634194_80634519	-1.7325965	-11.196517	3.629E-06	0.02080334	H3K27ac	LD
chr5	81092685	81092686	INS	chr5_80634194_80634519	-1.7325965	-11.196517	3.629E-06	0.02080334	H3K27ac	LD
chr4	103458500	103459374	DEL	chr4_103920841_103921822	1.84631778	11.1727071	3.6879E-06	0.02105874	H3K27ac	LD

chr4	103695022	103695133	DEL	chr4_103920841_103921822	1.84631778	11.1727071	3.6879E-06	0.02105874	H3K27ac	LD
chr4	103501647	103501648	INS	chr4_103920841_103921822	1.84631778	11.1727071	3.6879E-06	0.02105874	H3K27ac	LD
chr4	103857178	103857179	INS	chr4_103920841_103921822	1.84631778	11.1727071	3.6879E-06	0.02105874	H3K27ac	LD
chr8	124427809	124427810	INS	chr8_124871594_124872262	1.979052	11.1650577	3.7071E-06	0.02114753	H3K27ac	LD
chr10	10765079	10765286	DEL	chr10_11196723_11197211	1.95305346	11.1418937	3.7658E-06	0.02136726	H3K27ac	LD
chr10	10771994	10772255	DEL	chr10_11196723_11197211	1.95305346	11.1418937	3.7658E-06	0.02136726	H3K27ac	LD
chr10	10978479	10978748	DEL	chr10_11196723_11197211	1.95305346	11.1418937	3.7658E-06	0.02136726	H3K27ac	LD
chr15	119487532	119487533	INS	chr15_119822535_119823098	1.71521572	11.1398034	3.7711E-06	0.02136726	H3K27ac	LD
chr15	119495570	119495571	INS	chr15_119822535_119823098	1.71521572	11.1398034	3.7711E-06	0.02136726	H3K27ac	LD
chr10	11035110	11035111	INS	chr10_11196723_11197211	1.95305346	11.1418937	3.7658E-06	0.02136726	H3K27ac	LD
chr16	19642676	19642951	DEL	chr16_19647242_19647536	1.47024764	11.1238187	3.8123E-06	0.0215797	H3K27ac	LD
chr18	47229386	47229387	INS	chr18_46838267_46839346	4.0932465	11.1198478	3.8226E-06	0.02159631	H3K27ac	LD
chr8	38517457	38517523	DEL	chr8_38788229_38788677	1.21808862	11.1147122	3.836E-06	0.02160937	H3K27ac	LD

chr8	38831867	38832154	DEL	chr8_38788229_38788677	1.21808862	11.1147122	3.836E-06	0.02160937	H3K27ac	LD
chr8	38843802	38843803	INS	chr8_38788229_38788677	1.21808862	11.1147122	3.836E-06	0.02160937	H3K27ac	LD
chr2	72561458	72561555	DEL	chr2_72486234_72486488	3.16794444	11.1073222	3.8553E-06	0.02164172	H3K27ac	LD
chr2	82073775	82074792	DEL	chr2_81877948_81878286	1.64271007	11.0683712	3.9591E-06	0.02164172	H3K27ac	LD
chr2	82093517	82093790	DEL	chr2_81877948_81878286	1.64271007	11.0683712	3.9591E-06	0.02164172	H3K27ac	LD
chr2	82264697	82264743	DEL	chr2_81877948_81878286	1.64271007	11.0683712	3.9591E-06	0.02164172	H3K27ac	LD
chr14	129222574	129222638	DEL	chr14_129660619_129661491	-1.6612806	-11.080367	3.9268E-06	0.02164172	H3K27ac	LD
chr14	129437482	129437770	DEL	chr14_129660619_129661491	-1.6612806	-11.080367	3.9268E-06	0.02164172	H3K27ac	LD
chr14	129468677	129468941	DEL	chr14_129660619_129661491	-1.6612806	-11.080367	3.9268E-06	0.02164172	H3K27ac	LD
chr14	129584699	129585001	DEL	chr14_129660619_129661491	-1.6612806	-11.080367	3.9268E-06	0.02164172	H3K27ac	LD
chr14	129848177	129848503	DEL	chr14_129660619_129661491	-1.6612806	-11.080367	3.9268E-06	0.02164172	H3K27ac	LD
chr14	129931390	129931675	DEL	chr14_129660619_129661491	-1.6612806	-11.080367	3.9268E-06	0.02164172	H3K27ac	LD
chr3	124635148	124635652	DEL	chr3_125022430_125023202	2.12125	11.0680552	3.9599E-06	0.02164172	H3K27ac	LD

chr3	125228734	125228815	DEL	chr3_125022430_125023202	2.12125	11.0680552	3.9599E-06	0.02164172	H3K27ac	LD
chr6	13619990	13620298	DEL	chr6_13617936_13618305	2.40305222	11.0934705	3.8919E-06	0.02164172	H3K27ac	LD
chr6	13720716	13720794	DEL	chr6_13617936_13618305	2.40305222	11.0934705	3.8919E-06	0.02164172	H3K27ac	LD
chr2	81746841	81746842	INS	chr2_81877948_81878286	1.64271007	11.0683712	3.9591E-06	0.02164172	H3K27ac	LD
chr2	81721996	81721997	INS	chr2_81877948_81878286	1.64271007	11.0683712	3.9591E-06	0.02164172	H3K27ac	LD
chr2	81792419	81792420	INS	chr2_81877948_81878286	1.64271007	11.0683712	3.9591E-06	0.02164172	H3K27ac	LD
chr2	82318909	82318910	INS	chr2_81877948_81878286	1.64271007	11.0683712	3.9591E-06	0.02164172	H3K27ac	LD
chr14	129583021	129583022	INS	chr14_129660619_129661491	-1.6612806	-11.080367	3.9268E-06	0.02164172	H3K27ac	LD
chr14	129640944	129640945	INS	chr14_129660619_129661491	-1.6612806	-11.080367	3.9268E-06	0.02164172	H3K27ac	LD
chr14	129729439	129729440	INS	chr14_129660619_129661491	-1.6612806	-11.080367	3.9268E-06	0.02164172	H3K27ac	LD
chr14	129839166	129839167	INS	chr14_129660619_129661491	-1.6612806	-11.080367	3.9268E-06	0.02164172	H3K27ac	LD
chr3	124695342	124695343	INS	chr3_125022430_125023202	2.12125	11.0680552	3.9599E-06	0.02164172	H3K27ac	LD
chr3	125289244	125289245	INS	chr3_125022430_125023202	2.12125	11.0680552	3.9599E-06	0.02164172	H3K27ac	LD

chr3	125457742	125457743	INS	chr3_125022430_125023202	2.12125	11.0680552	3.9599E-06	0.02164172	H3K27ac	LD
chr1	161864361	161864362	INS	chr1_161509032_161509315	1.90996771	11.1055559	3.86E-06	0.02164172	H3K27ac	LD
chr6	13263776	13263777	INS	chr6_13617936_13618305	2.40305222	11.0934705	3.8919E-06	0.02164172	H3K27ac	LD
chr6	13604336	13604337	INS	chr6_13617936_13618305	2.40305222	11.0934705	3.8919E-06	0.02164172	H3K27ac	LD
chr6	14094327	14094328	INS	chr6_13617936_13618305	2.40305222	11.0934705	3.8919E-06	0.02164172	H3K27ac	LD
chr1	15531394	15531395	INS	chr1_15909964_15910433	5.80059206	11.0908501	3.8988E-06	0.02164172	H3K27ac	LD
chr13	28833746	28833747	INS	chr13_28963911_28964315	1.53287589	11.0290966	4.0669E-06	0.02216411	H3K27ac	LD
chr13	29034487	29034488	INS	chr13_28963911_28964315	1.53287589	11.0290966	4.0669E-06	0.02216411	H3K27ac	LD
chr2	130547527	130547528	INS	chr2_131031292_131031831	1.83508889	11.0251198	4.078E-06	0.02220393	H3K27ac	LD
chr3	127423963	127424132	DEL	chr3_127639516_127639809	2.03910965	11.0164988	4.1021E-06	0.02221761	H3K27ac	LD
chr3	127710800	127710882	DEL	chr3_127639516_127639809	2.03910965	11.0164988	4.1021E-06	0.02221761	H3K27ac	LD
chr3	127596527	127596528	INS	chr3_127639516_127639809	2.03910965	11.0164988	4.1021E-06	0.02221761	H3K27ac	LD
chr6	27274566	27274567	INS	chr6_27230942_27232110	1.75525822	11.0161091	4.1032E-06	0.02221761	H3K27ac	LD

chr6	27701019	27701020	INS	chr6_27230942_27232110	1.75525822	11.0161091	4.1032E-06	0.02221761	H3K27ac	LD
chr1	229979667	229979668	INS	chr1_230372146_230372508	1.59473044	11.0054622	4.1333E-06	0.02235982	H3K27ac	LD
chr13	3873862	3874135	DEL	chr13_4036227_4037624	3.64587	11.0008175	4.1465E-06	0.02238988	H3K27ac	LD
chr13	4348440	4348441	INS	chr13_4036227_4037624	3.64587	11.0008175	4.1465E-06	0.02238988	H3K27ac	LD
chr13	99464779	99464780	INS	chr13_99768790_99769026	4.73146438	10.9757496	4.2186E-06	0.02271382	H3K27ac	LD
chr13	99680159	99680160	INS	chr13_99768790_99769026	4.73146438	10.9757496	4.2186E-06	0.02271382	H3K27ac	LD
chr13	99867851	99867852	INS	chr13_99768790_99769026	4.73146438	10.9757496	4.2186E-06	0.02271382	H3K27ac	LD
chr7	116344914	116344915	INS	chr7_116446240_116446574	1.92901259	10.9705669	4.2336E-06	0.02271382	H3K27ac	LD
chr7	116359034	116359035	INS	chr7_116446240_116446574	1.92901259	10.9705669	4.2336E-06	0.02271382	H3K27ac	LD
chr7	116744972	116744973	INS	chr7_116446240_116446574	1.92901259	10.9705669	4.2336E-06	0.02271382	H3K27ac	LD
chr9	10681070	10681071	INS	chr9_10761234_10761540	3.84867	10.9672512	4.2433E-06	0.02274489	H3K27ac	LD
chr10	66701041	66701195	DEL	chr10_67073410_67074042	1.19534713	10.9513304	4.2901E-06	0.02293935	H3K27ac	LD
chr7	13162656	13162657	INS	chr7_13562991_13563293	2.44307389	10.9509149	4.2913E-06	0.02293935	H3K27ac	LD

chr7	13566904	13566905	INS	chr7_13562991_13563293	2.44307389	10.9509149	4.2913E-06	0.02293935	H3K27ac	LD
chr1	109083685	109083972	DEL	chr1_109239923_109240440	3.8120275	10.9413	4.3199E-06	0.02302893	H3K27ac	LD
chr1	109435022	109435023	INS	chr1_109239923_109240440	7.624055	10.9413	4.3199E-06	0.02302893	H3K27ac	LD
chr1	109576824	109576825	INS	chr1_109239923_109240440	3.8120275	10.9413	4.3199E-06	0.02302893	H3K27ac	LD
chr7	67736522	67736838	DEL	chr7_67701059_67701727	1.05979256	10.9339278	4.3419E-06	0.02305455	H3K27ac	LD
chr7	67999370	67999371	INS	chr7_67701059_67701727	2.11958513	10.9339278	4.3419E-06	0.02305455	H3K27ac	LD
chr6	136318572	136318573	INS	chr6_136316137_136316943	2.64539838	10.9304897	4.3522E-06	0.02305455	H3K27ac	LD
chr6	136434543	136434544	INS	chr6_136316137_136316943	2.64539838	10.9304897	4.3522E-06	0.02305455	H3K27ac	LD
chr1	6949592	6949593	INS	chr1_7440675_7441851	3.9573288	10.9323023	4.3468E-06	0.02305455	H3K27ac	LD
chr9	49906249	49906250	INS	chr9_50341231_50341831	1.75463413	10.9230365	4.3747E-06	0.02315261	H3K27ac	LD
chr18	34069885	34069935	DEL	chr18_34311782_34312069	2.48714488	10.9079748	4.4205E-06	0.02337385	H3K27ac	LD
chr17	30690234	30692559	DEL	chr17_30839903_30840345	3.59194333	10.8977592	4.4519E-06	0.02340035	H3K27ac	LD
chr10	56993758	56993759	INS	chr10_56943169_56943531	1.74348	10.8972377	4.4535E-06	0.02340035	H3K27ac	LD

chr10	57279843	57279844	INS	chr10_56943169_56943531	1.74348	10.8972377	4.4535E-06	0.02340035	H3K27ac	LD
chr17	30390842	30390843	INS	chr17_30839903_30840345	3.59194333	10.8977592	4.4519E-06	0.02340035	H3K27ac	LD
chr17	30427782	30427783	INS	chr17_30839903_30840345	3.59194333	10.8977592	4.4519E-06	0.02340035	H3K27ac	LD
chr17	30783559	30783560	INS	chr17_30839903_30840345	3.59194333	10.8977592	4.4519E-06	0.02340035	H3K27ac	LD
chr17	30935522	30935523	INS	chr17_30839903_30840345	3.59194333	10.8977592	4.4519E-06	0.02340035	H3K27ac	LD
chr10	23082230	23082379	DEL	chr10_23440846_23441470	1.69845035	10.8844056	4.4932E-06	0.02352489	H3K27ac	LD
chr10	23867366	23867655	DEL	chr10_23440846_23441470	1.69845035	10.8844056	4.4932E-06	0.02352489	H3K27ac	LD
chr10	23041302	23041303	INS	chr10_23440846_23441470	1.69845035	10.8844056	4.4932E-06	0.02352489	H3K27ac	LD
chr10	23904012	23904013	INS	chr10_23440846_23441470	1.69845035	10.8844056	4.4932E-06	0.02352489	H3K27ac	LD
chr6	5105612	5106347	DEL	chr6_5501683_5501957	1.68624486	10.8787412	4.5109E-06	0.02359637	H3K27ac	LD
chr13	51402263	51404525	DEL	chr13_51250091_51251343	1.42030417	10.8733379	4.5279E-06	0.02364033	H3K27ac	LD
chr1	247700564	247701336	DEL	chr1_248051338_248052213	19.5974294	10.8669053	4.5481E-06	0.02370626	H3K27ac	LD
chr5	91273146	91273676	DEL	chr5_91288331_91289584	1.95749743	10.8591249	4.5727E-06	0.02377122	H3K27ac	LD

chr5	90989883	90989884	INS	chr5_91288331_91289584	1.95749743	10.8591249	4.5727E-06	0.02377122	H3K27ac	LD
chr9	31833412	31833413	INS	chr9_31782715_31783070	3.22265528	10.8563488	4.5816E-06	0.02377491	H3K27ac	LD
chr11	19406781	19407000	DEL	chr11_19676705_19678228	4.04929744	10.839592	4.6353E-06	0.02388424	H3K27ac	LD
chr11	19462267	19462569	DEL	chr11_19676705_19678228	4.04929744	10.839592	4.6353E-06	0.02388424	H3K27ac	LD
chr11	19672334	19674473	DEL	chr11_19676705_19678228	4.04929744	10.839592	4.6353E-06	0.02388424	H3K27ac	LD
chr16	20823648	20823756	DEL	chr16_20815149_20815411	1.59723483	10.8459314	4.6149E-06	0.02388424	H3K27ac	LD
chr14	128682025	128682026	INS	chr14_129125129_129125395	2.19284517	10.8430352	4.6242E-06	0.02388424	H3K27ac	LD
chr16	20843292	20843293	INS	chr16_20815149_20815411	1.59723483	10.8459314	4.6149E-06	0.02388424	H3K27ac	LD
chr1	238389994	238390425	DEL	chr1_238139134_238140812	3.12623389	10.8225449	4.6906E-06	0.02406354	H3K27ac	LD
chr1	238310209	238310210	INS	chr1_238139134_238140812	3.12623389	10.8225449	4.6906E-06	0.02406354	H3K27ac	LD
chr1	238436395	238436396	INS	chr1_238139134_238140812	3.12623389	10.8225449	4.6906E-06	0.02406354	H3K27ac	LD
chr1	238446846	238446847	INS	chr1_238139134_238140812	3.12623389	10.8225449	4.6906E-06	0.02406354	H3K27ac	LD
chr4	36531513	36531910	DEL	chr4_36472393_36472711	1.33152275	10.8169997	4.7087E-06	0.02407238	H3K27ac	LD

chr4	36605924	36606245	DEL	chr4_36472393_36472711	1.33152275	10.8169997	4.7087E-06	0.02407238	H3K27ac	LD
chr4	36668600	36668852	DEL	chr4_36472393_36472711	2.6630455	10.8169997	4.7087E-06	0.02407238	H3K27ac	LD
chr4	36858905	36859249	DEL	chr4_36472393_36472711	1.33152275	10.8169997	4.7087E-06	0.02407238	H3K27ac	LD
chr7	8422028	8422029	INS	chr7_8628799_8629034	1.31837555	10.7948956	4.7819E-06	0.02442512	H3K27ac	LD
chr9	137126166	137126259	DEL	chr9_137171211_137172433	2.99858688	10.7551309	4.9168E-06	0.02443225	H3K27ac	LD
chr12	11994757	11995100	DEL	chr12_12445267_12445594	1.85955383	10.760155	4.8995E-06	0.02443225	H3K27ac	LD
chr1	886373	886458	DEL	chr1_854931_857957	5.60272944	10.7753834	4.8476E-06	0.02443225	H3K27ac	LD
chr12	12458313	12458836	DEL	chr12_12445267_12445594	1.85955383	10.760155	4.8995E-06	0.02443225	H3K27ac	LD
chr12	12768861	12768914	DEL	chr12_12445267_12445594	1.85955383	10.760155	4.8995E-06	0.02443225	H3K27ac	LD
chr1	1172385	1172452	DEL	chr1_854931_857957	5.60272944	10.7753834	4.8476E-06	0.02443225	H3K27ac	LD
chr1	1302436	1302629	DEL	chr1_854931_857957	5.60272944	10.7753834	4.8476E-06	0.02443225	H3K27ac	LD
chr11	24133091	24133292	DEL	chr11_24084731_24084989	2.28641222	10.7555792	4.9152E-06	0.02443225	H3K27ac	LD
chr7	39483090	39483091	INS	chr7_38998380_38998813	2.8822725	10.7880043	4.805E-06	0.02443225	H3K27ac	LD

chr15	77765155	77765156	INS	chr15_77964884_77965734	1.77678272	10.7816528	4.8264E-06	0.02443225	H3K27ac	LD
chr15	78026257	78026258	INS	chr15_77964884_77965734	1.77678272	10.7816528	4.8264E-06	0.02443225	H3K27ac	LD
chr1	471693	471694	INS	chr1_854931_857957	5.60272944	10.7753834	4.8476E-06	0.02443225	H3K27ac	LD
chr1	525720	525721	INS	chr1_854931_857957	5.60272944	10.7753834	4.8476E-06	0.02443225	H3K27ac	LD
chr1	534087	534088	INS	chr1_854931_857957	5.60272944	10.7753834	4.8476E-06	0.02443225	H3K27ac	LD
chr2	134145029	134145030	INS	chr2_134219102_134219977	3.71498563	10.7589101	4.9038E-06	0.02443225	H3K27ac	LD
chr2	134931943	134931944	INS	chr2_134706969_134707672	3.71808444	10.7635605	4.8878E-06	0.02443225	H3K27ac	LD
chr2	135188887	135188888	INS	chr2_134706969_134707672	3.71808444	10.7635605	4.8878E-06	0.02443225	H3K27ac	LD
chr2	141064640	141064641	INS	chr2_140733074_140733344	1.33813683	10.7726682	4.8568E-06	0.02443225	H3K27ac	LD
chr1	982463	982464	INS	chr1_854931_857957	5.60272944	10.7753834	4.8476E-06	0.02443225	H3K27ac	LD
chr1	1013579	1013580	INS	chr1_854931_857957	5.60272944	10.7753834	4.8476E-06	0.02443225	H3K27ac	LD
chr10	10325801	10325802	INS	chr10_10642456_10642677	1.41528938	10.7569385	4.9105E-06	0.02443225	H3K27ac	LD
chr10	10457462	10457463	INS	chr10_10642456_10642677	1.41528938	10.7569385	4.9105E-06	0.02443225	H3K27ac	LD

chr10	10925462	10925463	INS	chr10_10642456_10642677	1.41528938	10.7569385	4.9105E-06	0.02443225	H3K27ac	LD
chr1	1290914	1290915	INS	chr1_854931_857957	5.60272944	10.7753834	4.8476E-06	0.02443225	H3K27ac	LD
chr11	23955908	23955909	INS	chr11_24084731_24084989	2.28641222	10.7555792	4.9152E-06	0.02443225	H3K27ac	LD
chr11	24087156	24087157	INS	chr11_24084731_24084989	2.28641222	10.7555792	4.9152E-06	0.02443225	H3K27ac	LD
chr11	24060599	24060600	INS	chr11_24084731_24084989	2.28641222	10.7555792	4.9152E-06	0.02443225	H3K27ac	LD
chr11	24131246	24131247	INS	chr11_24084731_24084989	2.28641222	10.7555792	4.9152E-06	0.02443225	H3K27ac	LD
chr11	24583307	24583308	INS	chr11_24084731_24084989	2.28641222	10.7555792	4.9152E-06	0.02443225	H3K27ac	LD
chr12	1531071	1532287	DEL	chr12_1751133_1751741	1.57541617	10.737945	4.9764E-06	0.02443817	H3K27ac	LD
chr12	1532334	1532725	DEL	chr12_1751133_1751741	1.57541617	10.737945	4.9764E-06	0.02443817	H3K27ac	LD
chr8	131361394	131361395	INS	chr8_131588945_131589510	0.8638375	10.7484287	4.9399E-06	0.02443817	H3K27ac	LD
chr8	131516165	131516166	INS	chr8_131588945_131589510	0.8638375	10.7484287	4.9399E-06	0.02443817	H3K27ac	LD
chr12	1808085	1808086	INS	chr12_1751133_1751741	1.57541617	10.737945	4.9764E-06	0.02443817	H3K27ac	LD
chr12	1835366	1835367	INS	chr12_1751133_1751741	1.57541617	10.737945	4.9764E-06	0.02443817	H3K27ac	LD

chr12	1964906	1964907	INS	chr12_1751133_1751741	1.57541617	10.737945	4.9764E-06	0.02443817	H3K27ac	LD
chr12	2084059	2084060	INS	chr12_1751133_1751741	1.57541617	10.737945	4.9764E-06	0.02443817	H3K27ac	LD
chr12	2081731	2081732	INS	chr12_1751133_1751741	1.57541617	10.737945	4.9764E-06	0.02443817	H3K27ac	LD
chr12	2137275	2137276	INS	chr12_1751133_1751741	1.57541617	10.737945	4.9764E-06	0.02443817	H3K27ac	LD
chr15	30055088	30055089	INS	chr15_29713242_29714783	1.73693071	10.751947	4.9277E-06	0.02443817	H3K27ac	LD
chr15	30056980	30056981	INS	chr15_29713242_29714783	1.73693071	10.751947	4.9277E-06	0.02443817	H3K27ac	LD
chr17	58794419	58794543	DEL	chr17_59018723_59019721	2.07768278	10.726267	5.0173E-06	0.02461865	H3K27ac	LD
chr12	20206314	20209844	DUP	chr12_19775625_19776060	1.972358	10.7174412	5.0485E-06	0.02473031	H3K27ac	LD
chr12	20214723	20214724	INS	chr12_19775625_19776060	0.986179	10.7174412	5.0485E-06	0.02473031	H3K27ac	LD
chr11	2741070	2741252	DEL	chr11_2477394_2478536	2.70465549	10.7045039	5.0946E-06	0.02476987	H3K27ac	LD
chr11	2743815	2743868	DEL	chr11_2477394_2478536	2.70465549	10.7045039	5.0946E-06	0.02476987	H3K27ac	LD
chr11	2789307	2789469	DEL	chr11_2477394_2478536	2.70465549	10.7045039	5.0946E-06	0.02476987	H3K27ac	LD
chr3	86725525	86725731	DEL	chr3_86887322_86888374	1.44697083	10.7113377	5.0702E-06	0.02476987	H3K27ac	LD

chr11	2744435	2744436	INS	chr11_2477394_2478536	2.70465549	10.7045039	5.0946E-06	0.02476987	H3K27ac	LD
chr14	88636891	88636892	INS	chr14_88587546_88587879	1.47061367	10.7088761	5.079E-06	0.02476987	H3K27ac	LD
chr9	71133790	71133791	INS	chr9_71018331_71019713	1.11278144	10.7136321	5.062E-06	0.02476987	H3K27ac	LD
chr18	2885255	2885256	INS	chr18_2906045_2907447	6.80330308	10.7123915	5.0665E-06	0.02476987	H3K27ac	LD
chr11	1999473	1999474	INS	chr11_2477394_2478536	2.70465549	10.7045039	5.0946E-06	0.02476987	H3K27ac	LD
chr13	169024992	169025297	DEL	chr13_168809200_168810283	1.75795679	10.6845577	5.1667E-06	0.02507852	H3K27ac	LD
chr6	108419730	108419731	INS	chr6_108084220_108084409	0.82131306	10.6809788	5.1797E-06	0.02510025	H3K27ac	LD
chr5	99855656	99855890	DEL	chr5_100314390_100314618	1.95424267	10.6460172	5.3091E-06	0.02512466	H3K27ac	LD
chr5	100614304	100614375	DEL	chr5_100314390_100314618	1.95424267	10.6460172	5.3091E-06	0.02512466	H3K27ac	LD
chr5	100714252	100714312	DEL	chr5_100314390_100314618	1.95424267	10.6460172	5.3091E-06	0.02512466	H3K27ac	LD
chr4	14899095	14899166	DEL	chr4_15348022_15348653	2.04496	10.6473349	5.3042E-06	0.02512466	H3K27ac	LD
chr6	34921532	34921591	DEL	chr6_34584476_34585634	1.50045438	10.6635256	5.2439E-06	0.02512466	H3K27ac	LD
chr3	58398188	58398189	INS	chr3_58464360_58466789	10.4691806	10.6699262	5.2202E-06	0.02512466	H3K27ac	LD

chr3	58568874	58568875	INS	chr3_58464360_58466789	10.4691806	10.6699262	5.2202E-06	0.02512466	H3K27ac	LD
chr14	128828752	128828753	INS	chr14_129244349_129245092	2.68337	10.6521958	5.286E-06	0.02512466	H3K27ac	LD
chr14	128948366	128948367	INS	chr14_129244349_129245092	2.68337	10.6521958	5.286E-06	0.02512466	H3K27ac	LD
chr14	129165237	129165238	INS	chr14_129244349_129245092	2.68337	10.6521958	5.286E-06	0.02512466	H3K27ac	LD
chr14	129603938	129603939	INS	chr14_129244349_129245092	2.68337	10.6521958	5.286E-06	0.02512466	H3K27ac	LD
chr14	129692670	129692671	INS	chr14_129244349_129245092	2.68337	10.6521958	5.286E-06	0.02512466	H3K27ac	LD
chr1	118721934	118721935	INS	chr1_119188612_119188928	1.23255961	10.6571154	5.2677E-06	0.02512466	H3K27ac	LD
chr5	100023791	100023792	INS	chr5_100314390_100314618	1.95424267	10.6460172	5.3091E-06	0.02512466	H3K27ac	LD
chr5	100445243	100445244	INS	chr5_100314390_100314618	1.95424267	10.6460172	5.3091E-06	0.02512466	H3K27ac	LD
chr5	100573601	100573602	INS	chr5_100314390_100314618	1.95424267	10.6460172	5.3091E-06	0.02512466	H3K27ac	LD
chr5	100729228	100729229	INS	chr5_100314390_100314618	1.95424267	10.6460172	5.3091E-06	0.02512466	H3K27ac	LD
chr1	7105148	7105149	INS	chr1_7434396_7434940	3.5240836	10.6572927	5.267E-06	0.02512466	H3K27ac	LD
chr2	12000791	12000792	INS	chr2_12209035_12209641	1.36697	10.6758221	5.1986E-06	0.02512466	H3K27ac	LD

chr4	14953147	14953148	INS	chr4_15348022_15348653	2.04496	10.6473349	5.3042E-06	0.02512466	H3K27ac	LD
chr4	15194340	15194341	INS	chr4_15348022_15348653	2.04496	10.6473349	5.3042E-06	0.02512466	H3K27ac	LD
chr4	15350202	15350203	INS	chr4_15348022_15348653	2.04496	10.6473349	5.3042E-06	0.02512466	H3K27ac	LD
chr4	15629726	15629727	INS	chr4_15348022_15348653	2.04496	10.6473349	5.3042E-06	0.02512466	H3K27ac	LD
chr13	2519134	2519135	INS	chr13_2552198_2553053	6.61996429	10.6678941	5.2277E-06	0.02512466	H3K27ac	LD
chr12	43436422	43436423	INS	chr12_43398347_43398567	1.74443625	10.6682105	5.2266E-06	0.02512466	H3K27ac	LD
chr12	43502490	43502491	INS	chr12_43398347_43398567	1.74443625	10.6682105	5.2266E-06	0.02512466	H3K27ac	LD
chr13	12418289	12418290	INS	chr13_12489789_12491563	1.51170844	10.6498372	5.2948E-06	0.02512466	H3K27ac	LD
chr18	50193859	50194151	DEL	chr18_50608211_50610169	3.07299119	10.6279506	5.3774E-06	0.02540675	H3K27ac	LD
chr18	51033942	51033990	DEL	chr18_50608211_50610169	3.07299119	10.6279506	5.3774E-06	0.02540675	H3K27ac	LD
chr12	54857433	54857714	DEL	chr12_55115398_55116851	1.94837017	10.6073438	5.4565E-06	0.0255333	H3K27ac	LD
chr12	55102291	55102670	DEL	chr12_55115398_55116851	1.94837017	10.6073438	5.4565E-06	0.0255333	H3K27ac	LD
chr12	55111149	55111479	DEL	chr12_55115398_55116851	1.94837017	10.6073438	5.4565E-06	0.0255333	H3K27ac	LD

chr12	55570531	55570840	DEL	chr12_55115398_55116851	-1.9483702	-10.607344	5.4565E-06	0.0255333	H3K27ac	LD
chr11	4246983	4247422	DEL	chr11_4129679_4130468	2.56197878	10.6115725	5.4402E-06	0.0255333	H3K27ac	LD
chr12	54670370	54670371	INS	chr12_55115398_55116851	1.94837017	10.6073438	5.4565E-06	0.0255333	H3K27ac	LD
chr12	55139758	55139759	INS	chr12_55115398_55116851	1.94837017	10.6073438	5.4565E-06	0.0255333	H3K27ac	LD
chr12	55199996	55199997	INS	chr12_55115398_55116851	1.94837017	10.6073438	5.4565E-06	0.0255333	H3K27ac	LD
chr13	43266048	43266049	INS	chr13_43720399_43721087	3.72852667	10.6079804	5.454E-06	0.0255333	H3K27ac	LD
chr13	44057976	44057977	INS	chr13_43720399_43721087	3.72852667	10.6079804	5.454E-06	0.0255333	H3K27ac	LD
chr2	91435998	91435999	INS	chr2_91121287_91121558	1.78200077	10.6174754	5.4174E-06	0.0255333	H3K27ac	LD
chr17	21498766	21498767	INS	chr17_21969587_21969882	0.819854	10.6060427	5.4615E-06	0.02553647	H3K27ac	LD
chr13	79451200	79451201	INS	chr13_79164437_79165416	1.94059097	10.5853066	5.5425E-06	0.02589443	H3K27ac	LD
chr1	6020780	6020853	DEL	chr1_6414120_6415081	11.8584075	10.5823251	5.5543E-06	0.02590802	H3K27ac	LD
chr16	17987087	17987137	DEL	chr16_17991572_17992229	1.09666875	10.5760964	5.5789E-06	0.0259816	H3K27ac	LD
chr16	17905524	17905525	INS	chr16_17991572_17992229	1.09666875	10.5760964	5.5789E-06	0.0259816	H3K27ac	LD

chr6	120350354	120351088	DEL	chr6_120353022_120353376	0.899638	10.5652542	5.6221E-06	0.02606585	H3K27ac	LD
chr13	29533959	29533960	INS	chr13_29302243_29302866	1.99800672	10.5637436	5.6281E-06	0.02606585	H3K27ac	LD
chr13	29616720	29616721	INS	chr13_29302243_29302866	1.99800672	10.5637436	5.6281E-06	0.02606585	H3K27ac	LD
chr10	56993758	56993759	INS	chr10_57177213_57177628	2.04307678	10.567892	5.6115E-06	0.02606585	H3K27ac	LD
chr10	57279843	57279844	INS	chr10_57177213_57177628	2.04307678	10.567892	5.6115E-06	0.02606585	H3K27ac	LD
chr6	119856492	119856493	INS	chr6_120353022_120353376	0.899638	10.5652542	5.6221E-06	0.02606585	H3K27ac	LD
chr18	42634318	42634319	INS	chr18_42312002_42312672	2.06931619	10.5668027	5.6159E-06	0.02606585	H3K27ac	LD
chr16	20902920	20903040	DEL	chr16_20472212_20472632	0.80991545	10.5538881	5.6678E-06	0.02622865	H3K27ac	LD
chr2	5423095	5423096	INS	chr2_5101944_5105908	-7.1594875	-10.552007	5.6754E-06	0.02624309	H3K27ac	LD
chr2	141249576	141249649	DEL	chr2_141098332_141098884	2.33863056	10.5441602	5.7072E-06	0.02630975	H3K27ac	LD
chr2	141321676	141321776	DEL	chr2_141098332_141098884	2.33863056	10.5441602	5.7072E-06	0.02630975	H3K27ac	LD
chr2	141383621	141383622	INS	chr2_141098332_141098884	2.33863056	10.5441602	5.7072E-06	0.02630975	H3K27ac	LD
chr1	179587624	179587625	INS	chr1_179847723_179847936	3.06027481	10.5440235	5.7077E-06	0.02630975	H3K27ac	LD

chr7	65359418	65359419	INS	chr7_65727245_65728062	1.77888111	10.5388298	5.7289E-06	0.02636587	H3K27ac	LD
chr7	65580280	65580281	INS	chr7_65727245_65728062	1.77888111	10.5388298	5.7289E-06	0.02636587	H3K27ac	LD
chr14	20240682	20240742	DEL	chr14_20150328_20150802	2.64375128	10.535898	5.7409E-06	0.02637962	H3K27ac	LD
chr14	20235664	20235665	INS	chr14_20150328_20150802	2.64375128	10.535898	5.7409E-06	0.02637962	H3K27ac	LD
NW_018084937.1	28498	29545	DEL	chr5_63764893_63766054	2.82920375	10.5322837	5.7557E-06	0.026427	H3K27ac	LD
chr10	50867019	50867161	DEL	chr10_51120451_51121645	4.20341117	10.5122583	5.8386E-06	0.02666137	H3K27ac	LD
chr10	50873614	50873789	DEL	chr10_51120451_51121645	4.20341117	10.5122583	5.8386E-06	0.02666137	H3K27ac	LD
chr10	50683636	50683637	INS	chr10_51120451_51121645	4.20341117	10.5122583	5.8386E-06	0.02666137	H3K27ac	LD
chr10	50951655	50951656	INS	chr10_51120451_51121645	4.20341117	10.5122583	5.8386E-06	0.02666137	H3K27ac	LD
chr10	51021953	51021954	INS	chr10_51120451_51121645	4.20341117	10.5122583	5.8386E-06	0.02666137	H3K27ac	LD
chr10	51413283	51413284	INS	chr10_51120451_51121645	4.20341117	10.5122583	5.8386E-06	0.02666137	H3K27ac	LD
chr11	14435183	14435184	INS	chr11_14536222_14536561	0.82312319	10.5098967	5.8485E-06	0.02666485	H3K27ac	LD
chr11	14829396	14829397	INS	chr11_14536222_14536561	0.82312319	10.5098967	5.8485E-06	0.02666485	H3K27ac	LD

chr7	56183774	56183775	INS	chr7_56175749_56177123	2.60390333	10.5068636	5.8612E-06	0.02668121	H3K27ac	LD
chr7	56186592	56186593	INS	chr7_56175749_56177123	2.60390333	10.5068636	5.8612E-06	0.02668121	H3K27ac	LD
chr8	18880041	18880042	INS	chr8_19143142_19145712	-1.770965	-10.505099	5.8686E-06	0.02669416	H3K27ac	LD
chr12	7979174	7979227	DEL	chr12_7939182_7940558	3.35199056	10.4988316	5.895E-06	0.02674708	H3K27ac	LD
chr12	8162476	8162529	DEL	chr12_7939182_7940558	3.35199056	10.4988316	5.895E-06	0.02674708	H3K27ac	LD
chr12	7896595	7896596	INS	chr12_7939182_7940558	3.35199056	10.4988316	5.895E-06	0.02674708	H3K27ac	LD
chr11	4246983	4247422	DEL	chr11_4022098_4023386	2.18125589	10.4920455	5.9237E-06	0.02683728	H3K27ac	LD
chr3	4434846	4434847	INS	chr3_4628571_4628849	3.87652444	10.4900525	5.9321E-06	0.02683728	H3K27ac	LD
chr3	4659389	4659390	INS	chr3_4628571_4628849	3.87652444	10.4900525	5.9321E-06	0.02683728	H3K27ac	LD
chr7	61253426	61253427	INS	chr7_61513265_61513496	1.73136986	10.4821836	5.9657E-06	0.02696815	H3K27ac	LD
chr4	124164425	124164426	INS	chr4_124245882_124246934	-7.4599663	-10.47649	5.99E-06	0.02705754	H3K27ac	LD
chr1	253745096	253745097	INS	chr1_253385419_253385983	-0.655785	-10.471222	6.0127E-06	0.02713901	H3K27ac	LD
chr6	2346889	2347451	DEL	chr6_2361387_2363007	3.503457	10.4699725	6.0181E-06	0.02714244	H3K27ac	LD

chr10	40717164	40717238	DEL	chr10_41089461_41090074	3.33867507	10.4612666	6.0558E-06	0.02724959	H3K27ac	LD
chr10	40809878	40809879	INS	chr10_41089461_41090074	3.33867507	10.4612666	6.0558E-06	0.02724959	H3K27ac	LD
chr10	41213531	41213532	INS	chr10_41089461_41090074	3.33867507	10.4612666	6.0558E-06	0.02724959	H3K27ac	LD
chr1	269987980	269988126	DEL	chr1_269863670_269864131	1.35813542	10.4441011	6.1309E-06	0.02725275	H3K27ac	LD
chr17	278264	278594	DEL	chr17_267467_267690	2.09468308	10.4461779	6.1218E-06	0.02725275	H3K27ac	LD
chr17	288618	289415	DEL	chr17_267467_267690	2.09468308	10.4461779	6.1218E-06	0.02725275	H3K27ac	LD
chr17	372138	372238	DEL	chr17_267467_267690	2.09468308	10.4461779	6.1218E-06	0.02725275	H3K27ac	LD
chr16	66240211	66240212	INS	chr16_66000522_66000892	1.46810719	10.4493924	6.1077E-06	0.02725275	H3K27ac	LD
chr4	114933561	114933562	INS	chr4_115256574_115257458	-2.441463	-10.448045	6.1136E-06	0.02725275	H3K27ac	LD
chr4	115456306	115456307	INS	chr4_115256574_115257458	-2.441463	-10.448045	6.1136E-06	0.02725275	H3K27ac	LD
chr1	270259197	270259198	INS	chr1_269863670_269864131	1.35813542	10.4441011	6.1309E-06	0.02725275	H3K27ac	LD
chr1	270281077	270281078	INS	chr1_269863670_269864131	1.35813542	10.4441011	6.1309E-06	0.02725275	H3K27ac	LD
chr1	270339320	270339321	INS	chr1_269863670_269864131	1.35813542	10.4441011	6.1309E-06	0.02725275	H3K27ac	LD

chr17	97847	97848	INS	chr17_267467_267690	2.09468308	10.4461779	6.1218E-06	0.02725275	H3K27ac	LD
chr17	620961	620962	INS	chr17_267467_267690	2.09468308	10.4461779	6.1218E-06	0.02725275	H3K27ac	LD
chr16	17912364	17912365	INS	chr16_18127501_18127954	4.11670542	10.4552853	6.0819E-06	0.02725275	H3K27ac	LD
chr16	18285021	18285022	INS	chr16_18127501_18127954	4.11670542	10.4552853	6.0819E-06	0.02725275	H3K27ac	LD
chr12	3432719	3432720	INS	chr12_3277794_3278480	5.87586905	10.4546765	6.0845E-06	0.02725275	H3K27ac	LD
chr6	88954960	88954961	INS	chr6_89280173_89280856	1.85759958	10.4377489	6.159E-06	0.02733603	H3K27ac	LD
chr14	10149416	10149527	DEL	chr14_10168626_10169430	1.02852215	10.4290244	6.1978E-06	0.02742505	H3K27ac	LD
chr14	10131173	10131174	INS	chr14_10168626_10169430	1.02852215	10.4290244	6.1978E-06	0.02742505	H3K27ac	LD
chr14	10129333	10129334	INS	chr14_10168626_10169430	1.02852215	10.4290244	6.1978E-06	0.02742505	H3K27ac	LD
chr9	47562153	47562154	INS	chr9_47271183_47271483	0.69218263	10.4183933	6.2454E-06	0.02761494	H3K27ac	LD
chr12	25169812	25169813	INS	chr12_25408037_25408310	2.5404885	10.4161151	6.2557E-06	0.02763944	H3K27ac	LD
chr2	13122273	13123683	DEL	chr2_13384201_13385635	15.9525571	10.4145677	6.2627E-06	0.02764942	H3K27ac	LD
chr16	20675738	20675794	DEL	chr16_20356605_20357386	2.88442181	10.4043991	6.3087E-06	0.02781094	H3K27ac	LD

chr16	20301355	20301356	INS	chr16_20356605_20357386	2.88442181	10.4043991	6.3087E-06	0.02781094	H3K27ac	LD
chr15	45526492	45526543	DEL	chr15_45612893_45613580	4.16828222	10.4009128	6.3246E-06	0.02786001	H3K27ac	LD
chr7	74926005	74926006	INS	chr7_75414111_75414727	1.44874132	10.396803	6.3434E-06	0.02790079	H3K27ac	LD
chr1	10919281	10919282	INS	chr1_10929237_10929625	2.95317792	10.3870887	6.3881E-06	0.02805504	H3K27ac	LD
chr1	11069376	11069377	INS	chr1_10929237_10929625	2.95317792	10.3870887	6.3881E-06	0.02805504	H3K27ac	LD
chr2	13902150	13903219	DEL	NW_018085198.1_57657_57918	1.45653507	10.3832815	6.4056E-06	0.02809019	H3K27ac	LD
chr2	13933100	13933101	INS	NW_018085198.1_57657_57918	1.45653507	10.3832815	6.4056E-06	0.02809019	H3K27ac	LD
chr15	134332375	134332376	INS	chr15_134461098_134461502	0.71031338	10.3745062	6.4464E-06	0.02824778	H3K27ac	LD
chr17	59934477	59934606	DEL	chr17_60073371_60074878	5.09957569	10.3630708	6.5E-06	0.02834786	H3K27ac	LD
chr17	60029359	60029487	DEL	chr17_60073371_60074878	5.09957569	10.3630708	6.5E-06	0.02834786	H3K27ac	LD
chr5	644041	644122	DEL	chr5_545140_545767	1.27497928	10.3528989	6.548E-06	0.02834786	H3K27ac	LD
chr5	715869	715934	DEL	chr5_545140_545767	1.27497928	10.3528989	6.548E-06	0.02834786	H3K27ac	LD
chr5	754078	754137	DEL	chr5_545140_545767	1.27497928	10.3528989	6.548E-06	0.02834786	H3K27ac	LD
chr5	880646	880761	DEL	chr5_545140_545767	1.27497928	10.3528989	6.548E-06	0.02834786	H3K27ac	LD

chr16	17987087	17987137	DEL	chr16_18124469_18124719	2.22283125	10.3511423	6.5563E-06	0.02834786	H3K27ac	LD
chr17	59938267	59938268	INS	chr17_60073371_60074878	5.09957569	10.3630708	6.5E-06	0.02834786	H3K27ac	LD
chr17	60527683	60527684	INS	chr17_60073371_60074878	5.09957569	10.3630708	6.5E-06	0.02834786	H3K27ac	LD
chr5	824013	824014	INS	chr5_545140_545767	1.27497928	10.3528989	6.548E-06	0.02834786	H3K27ac	LD
chr5	830516	830517	INS	chr5_545140_545767	1.27497928	10.3528989	6.548E-06	0.02834786	H3K27ac	LD
chr5	884567	884568	INS	chr5_545140_545767	1.27497928	10.3528989	6.548E-06	0.02834786	H3K27ac	LD
chr16	17905524	17905525	INS	chr16_18124469_18124719	2.22283125	10.3511423	6.5563E-06	0.02834786	H3K27ac	LD
chr5	141020	141021	INS	chr5_545140_545767	1.27497928	10.3528989	6.548E-06	0.02834786	H3K27ac	LD
chr12	45568310	45568311	INS	chr12_46011556_46012306	4.20491111	10.360742	6.5109E-06	0.02834786	H3K27ac	LD
chr12	45980286	45980287	INS	chr12_46011556_46012306	4.20491111	10.360742	6.5109E-06	0.02834786	H3K27ac	LD
chr12	46165389	46165390	INS	chr12_46011556_46012306	4.20491111	10.360742	6.5109E-06	0.02834786	H3K27ac	LD
chr14	9444357	9444674	DEL	chr14_9760627_9762557	0.51840963	10.3481899	6.5704E-06	0.02838762	H3K27ac	LD
chr16	66107808	66108145	DEL	chr16_65760806_65761703	4.57245917	10.3378588	6.6198E-06	0.02851691	H3K27ac	LD
chr16	66110091	66110373	DEL	chr16_65760806_65761703	4.57245917	10.3378588	6.6198E-06	0.02851691	H3K27ac	LD

chr16	66222486	66222773	DEL	chr16_65760806_65761703	4.57245917	10.3378588	6.6198E-06	0.02851691	H3K27ac	LD
chr16	66087773	66087774	INS	chr16_65760806_65761703	4.57245917	10.3378588	6.6198E-06	0.02851691	H3K27ac	LD
chr18	2051651	2051931	DEL	chr18_1562726_1563403	3.36156613	10.3302208	6.6566E-06	0.02865431	H3K27ac	LD
chr17	44561489	44561489	BND	chr17_44667125_44669089	3.34244122	10.3141616	6.7347E-06	0.02877525	H3K27ac	LD
chr17	44852403	44852471	DEL	chr17_44667125_44669089	3.34244122	10.3141616	6.7347E-06	0.02877525	H3K27ac	LD
chr11	65431751	65432072	DEL	chr11_65254837_65255735	-1.97079	-10.289861	6.8548E-06	0.02877525	H3K27ac	LD
chr4	117978801	117978916	DEL	chr4_117730726_117732750	4.57694611	10.2929326	6.8395E-06	0.02877525	H3K27ac	LD
chr4	118001729	118001880	DEL	chr4_117730726_117732750	4.57694611	10.2929326	6.8395E-06	0.02877525	H3K27ac	LD
chr4	117999909	118000198	DEL	chr4_117730726_117732750	4.57694611	10.2929326	6.8395E-06	0.02877525	H3K27ac	LD
chr9	6306511	6306775	DEL	chr9_6156749_6157547	3.72378089	10.2912539	6.8478E-06	0.02877525	H3K27ac	LD
chr2	24460194	24460474	DEL	chr2_24953134_24953465	1.7651441	10.2885189	6.8615E-06	0.02877525	H3K27ac	LD
chr9	6501231	6502083	DUP	chr9_6156749_6157547	3.72378089	10.2912539	6.8478E-06	0.02877525	H3K27ac	LD
chr17	44289228	44289229	INS	chr17_44667125_44669089	3.34244122	10.3141616	6.7347E-06	0.02877525	H3K27ac	LD

chr17	44561498	44561499	INS	chr17_44667125_44669089	3.34244122	10.3141616	6.7347E-06	0.02877525	H3K27ac	LD
chr17	44951386	44951387	INS	chr17_44667125_44669089	3.34244122	10.3141616	6.7347E-06	0.02877525	H3K27ac	LD
chr17	45103545	45103546	INS	chr17_44667125_44669089	3.34244122	10.3141616	6.7347E-06	0.02877525	H3K27ac	LD
chr11	65260979	65260980	INS	chr11_65254837_65255735	-1.97079	-10.289861	6.8548E-06	0.02877525	H3K27ac	LD
chr11	65412580	65412581	INS	chr11_65254837_65255735	-1.97079	-10.289861	6.8548E-06	0.02877525	H3K27ac	LD
chr2	86987685	86987686	INS	chr2_87380539_87382578	2.69404222	10.2974025	6.8172E-06	0.02877525	H3K27ac	LD
chr4	117473079	117473080	INS	chr4_117730726_117732750	4.57694611	10.2929326	6.8395E-06	0.02877525	H3K27ac	LD
chr4	117746392	117746393	INS	chr4_117730726_117732750	4.57694611	10.2929326	6.8395E-06	0.02877525	H3K27ac	LD
chr4	117945046	117945047	INS	chr4_117730726_117732750	4.57694611	10.2929326	6.8395E-06	0.02877525	H3K27ac	LD
chr4	118002624	118002625	INS	chr4_117730726_117732750	4.57694611	10.2929326	6.8395E-06	0.02877525	H3K27ac	LD
chr4	118005675	118005676	INS	chr4_117730726_117732750	4.57694611	10.2929326	6.8395E-06	0.02877525	H3K27ac	LD
chr4	118083331	118083332	INS	chr4_117730726_117732750	4.57694611	10.2929326	6.8395E-06	0.02877525	H3K27ac	LD
chr4	118131010	118131011	INS	chr4_117730726_117732750	4.57694611	10.2929326	6.8395E-06	0.02877525	H3K27ac	LD

chr4	127211452	127211453	INS	chr4_127312643_127313508	1.14709572	10.2985949	6.8113E-06	0.02877525	H3K27ac	LD
chr4	127347628	127347629	INS	chr4_127312643_127313508	2.29419144	10.2985949	6.8113E-06	0.02877525	H3K27ac	LD
chr9	6040023	6040024	INS	chr9_6156749_6157547	3.72378089	10.2912539	6.8478E-06	0.02877525	H3K27ac	LD
chr13	202667554	202667555	INS	chr13_202936662_202937118	2.70287035	10.3171574	6.72E-06	0.02877525	H3K27ac	LD
chr13	202669454	202669455	INS	chr13_202936662_202937118	2.70287035	10.3171574	6.72E-06	0.02877525	H3K27ac	LD
chr7	19170696	19170697	INS	chr7_19470551_19471683	1.88668439	10.2888148	6.86E-06	0.02877525	H3K27ac	LD
chr17	5748712	5748713	INS	chr17_5259143_5259347	0.89793281	10.2920848	6.8437E-06	0.02877525	H3K27ac	LD
chr2	24871327	24871328	INS	chr2_24953134_24953465	1.7651441	10.2885189	6.8615E-06	0.02877525	H3K27ac	LD
chr2	25298951	25298952	INS	chr2_24953134_24953465	1.7651441	10.2885189	6.8615E-06	0.02877525	H3K27ac	LD
chr4	2479119	2479120	INS	chr4_2902742_2903543	3.30986211	10.3231131	6.691E-06	0.02877525	H3K27ac	LD
chr2	135773551	135773675	DEL	chr2_136195666_136196323	3.067874	10.2847594	6.8803E-06	0.02879233	H3K27ac	LD
chr2	136156115	136156116	INS	chr2_136195666_136196323	3.067874	10.2847594	6.8803E-06	0.02879233	H3K27ac	LD
chr2	136266529	136266530	INS	chr2_136195666_136196323	3.067874	10.2847594	6.8803E-06	0.02879233	H3K27ac	LD
chr9	3215951	3215952	INS	chr9_3519811_3520498	0.85891538	10.2829137	6.8895E-06	0.0288105	H3K27ac	LD

chr3	115022507	115022695	DEL	chr3_115213910_115214324	1.34799233	10.2817171	6.8956E-06	0.02881506	H3K27ac	LD
chr15	31033405	31033489	DEL	chr15_31299952_31300857	6.09779644	10.2640958	6.9847E-06	0.02894006	H3K27ac	LD
chr15	31232754	31232836	DEL	chr15_31299952_31300857	6.09779644	10.2640958	6.9847E-06	0.02894006	H3K27ac	LD
chr15	31226637	31226949	DEL	chr15_31299952_31300857	6.09779644	10.2640958	6.9847E-06	0.02894006	H3K27ac	LD
chr15	31323509	31323687	DEL	chr15_31299952_31300857	6.09779644	10.2640958	6.9847E-06	0.02894006	H3K27ac	LD
chr5	64473687	64473688	INS	chr5_64434217_64435025	11.1766285	10.2701207	6.9541E-06	0.02894006	H3K27ac	LD
chr3	19844357	19844358	INS	chr3_19402635_19404115	2.98897594	10.2723043	6.943E-06	0.02894006	H3K27ac	LD
chr15	31032850	31032851	INS	chr15_31299952_31300857	6.09779644	10.2640958	6.9847E-06	0.02894006	H3K27ac	LD
chr15	31116638	31116639	INS	chr15_31299952_31300857	6.09779644	10.2640958	6.9847E-06	0.02894006	H3K27ac	LD
chr15	31238478	31238479	INS	chr15_31299952_31300857	6.09779644	10.2640958	6.9847E-06	0.02894006	H3K27ac	LD
chr15	31269036	31269037	INS	chr15_31299952_31300857	6.09779644	10.2640958	6.9847E-06	0.02894006	H3K27ac	LD
chr15	31324555	31324556	INS	chr15_31299952_31300857	6.09779644	10.2640958	6.9847E-06	0.02894006	H3K27ac	LD
chr16	32823106	32823107	INS	chr16_32917163_32917809	0.69552425	10.2580731	7.0155E-06	0.02900604	H3K27ac	LD

chr16	33037165	33037166	INS	chr16_32917163_32917809	0.69552425	10.2580731	7.0155E-06	0.02900604	H3K27ac	LD
chr9	66859088	66859530	DEL	chr9_67036126_67036348	2.3113655	10.2454266	7.0806E-06	0.02914989	H3K27ac	LD
chr9	67446707	67446810	DEL	chr9_67036126_67036348	2.3113655	10.2454266	7.0806E-06	0.02914989	H3K27ac	LD
chr5	10762104	10762578	DEL	chr5_10628942_10629393	1.37493425	10.2445577	7.0851E-06	0.02914989	H3K27ac	LD
chr9	66803205	66803206	INS	chr9_67036126_67036348	2.3113655	10.2454266	7.0806E-06	0.02914989	H3K27ac	LD
chr9	67181333	67181334	INS	chr9_67036126_67036348	2.3113655	10.2454266	7.0806E-06	0.02914989	H3K27ac	LD
chr9	67224531	67224532	INS	chr9_67036126_67036348	2.3113655	10.2454266	7.0806E-06	0.02914989	H3K27ac	LD
chr10	60114996	60114997	INS	chr10_60510856_60511386	1.07258375	10.2461726	7.0768E-06	0.02914989	H3K27ac	LD
chr8	132533215	132533216	INS	chr8_132878692_132879055	1.9168046	10.2256409	7.1839E-06	0.02953541	H3K27ac	LD
chr1	33412027	33412313	DEL	chr1_33470183_33470902	1.92807956	10.2174687	7.227E-06	0.02967109	H3K27ac	LD
chr1	33413400	33413541	DEL	chr1_33470183_33470902	1.92807956	10.2174687	7.227E-06	0.02967109	H3K27ac	LD
chr17	29879817	29880195	DEL	chr17_30070477_30070968	1.38791	10.2142069	7.2443E-06	0.02967971	H3K27ac	LD
chr17	29969011	29969075	DEL	chr17_30070477_30070968	1.38791	10.2142069	7.2443E-06	0.02967971	H3K27ac	LD

chr17	30263351	30263551	DEL	chr17_30070477_30070968	1.38791	10.2142069	7.2443E-06	0.02967971	H3K27ac	LD
chr14	24037273	24042908	DEL	chr14_23927471_23927847	1.76479111	10.2003516	7.3183E-06	0.02978233	H3K27ac	LD
chr1	51169511	51170322	DEL	chr1_51150092_51150813	2.27719088	10.2048868	7.294E-06	0.02978233	H3K27ac	LD
chr14	134566976	134566977	INS	chr14_134925102_134925326	1.85120875	10.1996654	7.322E-06	0.02978233	H3K27ac	LD
chr14	135230867	135230868	INS	chr14_134925102_134925326	1.85120875	10.1996654	7.322E-06	0.02978233	H3K27ac	LD
chr1	250222484	250222485	INS	chr1_250401971_250402211	1.07014549	10.1990528	7.3253E-06	0.02978233	H3K27ac	LD
chr1	250527567	250527568	INS	chr1_250401971_250402211	1.07014549	10.1990528	7.3253E-06	0.02978233	H3K27ac	LD
chr14	23717163	23717164	INS	chr14_23927471_23927847	1.76479111	10.2003516	7.3183E-06	0.02978233	H3K27ac	LD
chr14	24190259	24190260	INS	chr14_23927471_23927847	1.76479111	10.2003516	7.3183E-06	0.02978233	H3K27ac	LD
chr14	24301411	24301412	INS	chr14_23927471_23927847	1.76479111	10.2003516	7.3183E-06	0.02978233	H3K27ac	LD
chr14	24303900	24303901	INS	chr14_23927471_23927847	1.76479111	10.2003516	7.3183E-06	0.02978233	H3K27ac	LD
chr7	66387015	66387090	DEL	chr7_66381328_66382334	1.843591	10.1920711	7.3629E-06	0.02989388	H3K27ac	LD
chr7	66694642	66694831	DEL	chr7_66381328_66382334	1.843591	10.1920711	7.3629E-06	0.02989388	H3K27ac	LD

chr9	496224	496225	INS	chr9_775761_776400	1.13161867	10.187735	7.3864E-06	0.02992702	H3K27ac	LD
chr9	586549	586550	INS	chr9_775761_776400	1.13161867	10.187735	7.3864E-06	0.02992702	H3K27ac	LD
chr9	637694	637695	INS	chr9_775761_776400	2.26323733	10.187735	7.3864E-06	0.02992702	H3K27ac	LD
chr6	16656983	16656984	INS	chr6_17128149_17129505	3.38870694	10.1752082	7.4547E-06	0.03014131	H3K27ac	LD
chr6	16897321	16897322	INS	chr6_17128149_17129505	3.38870694	10.1752082	7.4547E-06	0.03014131	H3K27ac	LD
chr6	17135580	17135581	INS	chr6_17128149_17129505	3.38870694	10.1752082	7.4547E-06	0.03014131	H3K27ac	LD
chr4	1378429	1378430	INS	chr4_1290339_1290567	1.49738583	10.1741441	7.4606E-06	0.0301441	H3K27ac	LD
chr13	69008345	69008346	INS	chr13_68746675_68747370	2.57518594	10.1668106	7.5009E-06	0.03028629	H3K27ac	LD
chr13	36262159	36262160	INS	chr13_35962206_35962464	2.06462056	10.1609137	7.5336E-06	0.0303547	H3K27ac	LD
chr15	101078193	101078194	INS	chr15_101272261_101272639	2.62425288	10.1600062	7.5386E-06	0.0303547	H3K27ac	LD
chr15	101462879	101462880	INS	chr15_101272261_101272639	2.62425288	10.1600062	7.5386E-06	0.0303547	H3K27ac	LD
chr5	14310373	14311153	DEL	chr5_13823717_13824765	1.86864286	10.1562454	7.5595E-06	0.03041798	H3K27ac	LD
chr14	89723334	89723446	DEL	chr14_89707225_89708048	1.22362044	10.1473144	7.6094E-06	0.03055411	H3K27ac	LD

chr17	52647390	52647877	DEL	chr17_52923486_52923969	2.13504117	10.1421897	7.6382E-06	0.03055411	H3K27ac	LD
chr17	52648126	52648292	DEL	chr17_52923486_52923969	2.13504117	10.1421897	7.6382E-06	0.03055411	H3K27ac	LD
chr2	13071290	13071358	DEL	chr2_13186693_13187515	1.61943611	10.139053	7.6559E-06	0.03055411	H3K27ac	LD
chr12	59619448	59619449	INS	chr12_59787795_59788021	2.24279294	10.1435211	7.6307E-06	0.03055411	H3K27ac	LD
chr12	59757950	59757951	INS	chr12_59787795_59788021	2.24279294	10.1435211	7.6307E-06	0.03055411	H3K27ac	LD
chr17	54714175	54714176	INS	chr17_54603365_54603662	1.55463167	10.1408243	7.6459E-06	0.03055411	H3K27ac	LD
chr17	54807252	54807253	INS	chr17_54603365_54603662	1.55463167	10.1408243	7.6459E-06	0.03055411	H3K27ac	LD
chr2	135110908	135110909	INS	chr2_134706969_134707672	3.72601438	10.1395541	7.6531E-06	0.03055411	H3K27ac	LD
chr2	13069331	13069332	INS	chr2_13186693_13187515	1.61943611	10.139053	7.6559E-06	0.03055411	H3K27ac	LD
chr4	20296091	20296092	INS	chr4_20562406_20563326	1.19210444	10.1406718	7.6468E-06	0.03055411	H3K27ac	LD
chr4	20913966	20913967	INS	chr4_20562406_20563326	1.19210444	10.1406718	7.6468E-06	0.03055411	H3K27ac	LD
chr1	5682318	5682618	DEL	chr1_5732250_5732894	1.31563122	10.1268436	7.7252E-06	0.03066359	H3K27ac	LD
chr1	6138925	6143057	DEL	chr1_5732250_5732894	1.31563122	10.1268436	7.7252E-06	0.03066359	H3K27ac	LD

chr1	5262246	5262247	INS	chr1_5732250_5732894	1.31563122	10.1268436	7.7252E-06	0.03066359	H3K27ac	LD
chr1	5549345	5549346	INS	chr1_5732250_5732894	1.31563122	10.1268436	7.7252E-06	0.03066359	H3K27ac	LD
chr1	5573097	5573098	INS	chr1_5732250_5732894	1.31563122	10.1268436	7.7252E-06	0.03066359	H3K27ac	LD
chr11	63118567	63118881	DEL	chr11_63041609_63042129	1.60976938	10.1153549	7.7911E-06	0.03088313	H3K27ac	LD
chr11	63122698	63122756	DEL	chr11_63041609_63042129	1.60976938	10.1153549	7.7911E-06	0.03088313	H3K27ac	LD
chr10	17726267	17726701	DEL	chr10_18212545_18213560	1.29406726	10.1082589	7.8321E-06	0.03089926	H3K27ac	LD
chr10	18435856	18437494	DEL	chr10_18212545_18213560	1.29406726	10.1082589	7.8321E-06	0.03089926	H3K27ac	LD
chr10	17824032	17824033	INS	chr10_18212545_18213560	1.29406726	10.1082589	7.8321E-06	0.03089926	H3K27ac	LD
chr10	17822893	17822894	INS	chr10_18212545_18213560	1.29406726	10.1082589	7.8321E-06	0.03089926	H3K27ac	LD
chr10	17827052	17827053	INS	chr10_18212545_18213560	1.29406726	10.1082589	7.8321E-06	0.03089926	H3K27ac	LD
chr10	17920107	17920108	INS	chr10_18212545_18213560	1.29406726	10.1082589	7.8321E-06	0.03089926	H3K27ac	LD
chr10	18315872	18315873	INS	chr10_18212545_18213560	1.29406726	10.1082589	7.8321E-06	0.03089926	H3K27ac	LD
chr10	33056452	33056453	INS	chr10_33120710_33121117	2.13967722	10.1019763	7.8686E-06	0.03098063	H3K27ac	LD

chr10	32981267	32981268	INS	chr10_33120710_33121117	2.13967722	10.1019763	7.8686E-06	0.03098063	H3K27ac	LD
chr3	113818128	113818129	INS	chr3_113430215_113430465	1.71656963	10.1036538	7.8588E-06	0.03098063	H3K27ac	LD
chr1	130666453	130666454	INS	chr1_130858522_130858773	1.58203472	10.0907425	7.9343E-06	0.0312184	H3K27ac	LD
chr2	83266705	83266840	DEL	chr2_83219651_83222036	2.36219042	10.0748392	8.0284E-06	0.03152509	H3K27ac	LD
chr2	82897544	82897545	INS	chr2_83219651_83222036	2.36219042	10.0748392	8.0284E-06	0.03152509	H3K27ac	LD
chr2	83655251	83655252	INS	chr2_83219651_83222036	2.36219042	10.0748392	8.0284E-06	0.03152509	H3K27ac	LD
chr11	1101793	1102070	DEL	chr11_878415_878666	0.92740669	10.069045	8.063E-06	0.0316397	H3K27ac	LD
chr3	86725525	86725731	DEL	chr3_86744056_86744327	3.74357014	10.0560546	8.1411E-06	0.03184199	H3K27ac	LD
chr8	71855913	71855914	INS	chr8_72182932_72183399	1.25525533	10.0550618	8.1471E-06	0.03184199	H3K27ac	LD
chr8	71972811	71972812	INS	chr8_72182932_72183399	1.25525533	10.0550618	8.1471E-06	0.03184199	H3K27ac	LD
chr8	72206652	72206653	INS	chr8_72182932_72183399	1.25525533	10.0550618	8.1471E-06	0.03184199	H3K27ac	LD
chr11	58571454	58571455	INS	chr11_59051909_59052347	1.55100567	10.0466319	8.1983E-06	0.0319144	H3K27ac	LD
chr11	58645300	58645301	INS	chr11_59051909_59052347	1.55100567	10.0466319	8.1983E-06	0.0319144	H3K27ac	LD

chr11	58708093	58708094	INS	chr11_59051909_59052347	1.55100567	10.0466319	8.1983E-06	0.0319144	H3K27ac	LD
chr11	58913266	58913267	INS	chr11_59051909_59052347	1.55100567	10.0466319	8.1983E-06	0.0319144	H3K27ac	LD
chr11	59303245	59303246	INS	chr11_59051909_59052347	1.55100567	10.0466319	8.1983E-06	0.0319144	H3K27ac	LD
chr13	129600877	129600878	INS	chr13_129936552_129937600	1.93129728	10.047274	8.1944E-06	0.0319144	H3K27ac	LD
chr3	14180558	14180820	DEL	chr3_14613957_14614841	0.98347856	10.0420306	8.2264E-06	0.03198131	H3K27ac	LD
chr12	38148281	38148334	DEL	chr12_38625426_38627052	0.56749875	10.0394127	8.2425E-06	0.03202241	H3K27ac	LD
chr1	272134874	272135176	DEL	chr1_272167725_272168249	3.35538167	10.0300084	8.3004E-06	0.03211956	H3K27ac	LD
chr1	272014785	272014786	INS	chr1_272167725_272168249	3.35538167	10.0300084	8.3004E-06	0.03211956	H3K27ac	LD
chr1	272127592	272127593	INS	chr1_272167725_272168249	3.35538167	10.0300084	8.3004E-06	0.03211956	H3K27ac	LD
chr1	272240079	272240080	INS	chr1_272167725_272168249	3.35538167	10.0300084	8.3004E-06	0.03211956	H3K27ac	LD
chr1	272410850	272410851	INS	chr1_272167725_272168249	3.35538167	10.0300084	8.3004E-06	0.03211956	H3K27ac	LD
chr1	272586414	272586415	INS	chr1_272167725_272168249	3.35538167	10.0300084	8.3004E-06	0.03211956	H3K27ac	LD
chr3	19844572	19853848	DEL	chr3_19414639_19415095	2.49286185	10.0246581	8.3335E-06	0.0321569	H3K27ac	LD

chr3	19846188	19847874	DEL	chr3_19414639_19415095	2.49286185	10.0246581	8.3335E-06	0.0321569	H3K27ac	LD
chr3	23585264	23585539	DEL	chr3_23917718_23918377	4.72228939	10.022256	8.3484E-06	0.0321569	H3K27ac	LD
chr3	23753861	23754307	DEL	chr3_23917718_23918377	4.72228939	10.022256	8.3484E-06	0.0321569	H3K27ac	LD
chr3	23971745	23972018	DEL	chr3_23917718_23918377	4.72228939	10.022256	8.3484E-06	0.0321569	H3K27ac	LD
chr3	23555285	23555286	INS	chr3_23917718_23918377	4.72228939	10.022256	8.3484E-06	0.0321569	H3K27ac	LD
chr3	23624988	23624989	INS	chr3_23917718_23918377	4.72228939	10.022256	8.3484E-06	0.0321569	H3K27ac	LD
chr17	51214823	51214891	DEL	chr17_51281332_51282148	1.51473444	10.0192995	8.3669E-06	0.03217704	H3K27ac	LD
chr3	102796923	102797184	DEL	chr3_102494423_102494987	2.07328736	10.0178919	8.3756E-06	0.03217704	H3K27ac	LD
chr14	23249058	23249104	DEL	chr14_23055882_23056224	0.94118625	10.0185659	8.3714E-06	0.03217704	H3K27ac	LD
chr17	51175340	51175341	INS	chr17_51281332_51282148	1.51473444	10.0192995	8.3669E-06	0.03217704	H3K27ac	LD
chr15	128681291	128681292	INS	chr15_128443987_128445011	2.23886333	10.0098987	8.4257E-06	0.0323482	H3K27ac	LD
chr13	117655524	117655525	INS	chr13_117672731_117673022	0.78400642	10.0088267	8.4324E-06	0.03235287	H3K27ac	LD
chr16	47580501	47580502	INS	chr16_47720793_47721778	0.46008221	10.007373	8.4416E-06	0.03236678	H3K27ac	LD

chr18	28474595	28474809	DEL	chr18_28475719_28475944	1.26089819	9.99303972	8.5324E-06	0.03267224	H3K27ac	LD
chr17	49961229	49962027	DEL	chr17_50075353_50076132	1.30477051	9.97384641	8.6557E-06	0.03293441	H3K27ac	LD
chr3	112451069	112451465	DEL	chr3_112649444_112650414	1.05349161	9.97363704	8.6571E-06	0.03293441	H3K27ac	LD
chr3	112852046	112852120	DEL	chr3_112649444_112650414	1.05349161	9.97363704	8.6571E-06	0.03293441	H3K27ac	LD
chr18	54184198	54184199	INS	chr18_54254701_54256098	8.73005347	9.97849915	8.6257E-06	0.03293441	H3K27ac	LD
chr7	100831415	100831416	INS	chr7_100395749_100397123	4.40823333	9.97718207	8.6342E-06	0.03293441	H3K27ac	LD
chr3	112196001	112196002	INS	chr3_112649444_112650414	1.05349161	9.97363704	8.6571E-06	0.03293441	H3K27ac	LD
chr3	112705032	112705033	INS	chr3_112649444_112650414	1.05349161	9.97363704	8.6571E-06	0.03293441	H3K27ac	LD
chr3	113042638	113042639	INS	chr3_112649444_112650414	1.05349161	9.97363704	8.6571E-06	0.03293441	H3K27ac	LD
chr3	113086561	113086562	INS	chr3_112649444_112650414	1.05349161	9.97363704	8.6571E-06	0.03293441	H3K27ac	LD
chr1	142751907	142759959	DEL	chr1_142499959_142501303	1.85172896	9.96371838	8.7216E-06	0.03315834	H3K27ac	LD
chr14	61916302	61917084	DEL	chr14_61971938_61972352	1.27531778	9.95490085	8.7794E-06	0.03317405	H3K27ac	LD
chr6	70706484	70707220	DEL	chr6_71051648_71051965	0.91404206	9.95799398	8.7591E-06	0.03317405	H3K27ac	LD

chr6	71495253	71495599	DEL	chr6_71051648_71051965	0.91404206	9.95799398	8.7591E-06	0.03317405	H3K27ac	LD
chr6	71543796	71543865	DEL	chr6_71051648_71051965	0.91404206	9.95799398	8.7591E-06	0.03317405	H3K27ac	LD
chr3	57981305	57981372	DEL	chr3_57968667_57968912	2.99509983	9.95445308	8.7824E-06	0.03317405	H3K27ac	LD
chr14	61560028	61560029	INS	chr14_61971938_61972352	1.27531778	9.95490085	8.7794E-06	0.03317405	H3K27ac	LD
chr14	62448342	62448343	INS	chr14_61971938_61972352	1.27531778	9.95490085	8.7794E-06	0.03317405	H3K27ac	LD
chr6	71337295	71337296	INS	chr6_71051648_71051965	0.91404206	9.95799398	8.7591E-06	0.03317405	H3K27ac	LD
chr1	77522824	77522825	INS	chr1_77804048_77804458	1.67930593	9.95548853	8.7756E-06	0.03317405	H3K27ac	LD
chr3	57723299	57723300	INS	chr3_57968667_57968912	2.99509983	9.95445308	8.7824E-06	0.03317405	H3K27ac	LD
chr6	126701519	126701519	BND	chr6_126430499_126431304	2.20836994	9.93845399	8.8884E-06	0.03337919	H3K27ac	LD
chr11	28918853	28918853	BND	chr11_28727167_28727549	2.48394194	9.93681365	8.8994E-06	0.03337919	H3K27ac	LD
chr11	28519733	28519802	DEL	chr11_28727167_28727549	2.48394194	9.93681365	8.8994E-06	0.03337919	H3K27ac	LD
chr3	73851375	73851376	INS	chr3_74220024_74221096	1.51041889	9.9436113	8.8541E-06	0.03337919	H3K27ac	LD
chr13	79327300	79327301	INS	chr13_79286715_79287085	1.22366979	9.9373585	8.8957E-06	0.03337919	H3K27ac	LD

chr6	126701519	126701520	INS	chr6_126430499_126431304	2.20836994	9.93845399	8.8884E-06	0.03337919	H3K27ac	LD
chr11	28564349	28564350	INS	chr11_28727167_28727549	2.48394194	9.93681365	8.8994E-06	0.03337919	H3K27ac	LD
chr11	28706855	28706856	INS	chr11_28727167_28727549	2.48394194	9.93681365	8.8994E-06	0.03337919	H3K27ac	LD
chr11	28737320	28737321	INS	chr11_28727167_28727549	2.48394194	9.93681365	8.8994E-06	0.03337919	H3K27ac	LD
chr11	28850784	28850785	INS	chr11_28727167_28727549	2.48394194	9.93681365	8.8994E-06	0.03337919	H3K27ac	LD
chr11	28918838	28918839	INS	chr11_28727167_28727549	2.48394194	9.93681365	8.8994E-06	0.03337919	H3K27ac	LD
chr12	576934	576935	INS	chr12_577886_578226	2.58832994	9.9340612	8.9178E-06	0.03342684	H3K27ac	LD
chr14	125145189	125145500	DEL	chr14_125158247_125159911	-5.69599	-9.9079598	9.0945E-06	0.0335112	H3K27ac	LD
chr9	113402496	113402804	DEL	chr9_113755012_113756003	4.66723403	9.91847915	9.0228E-06	0.0335112	H3K27ac	LD
chr6	18892358	18893075	DEL	chr6_18889940_18890658	2.10979839	9.90292193	9.129E-06	0.0335112	H3K27ac	LD
chr18	13404305	13404531	DEL	chr18_13628273_13628688	2.051004	9.90398734	9.1217E-06	0.0335112	H3K27ac	LD
chr18	13707566	13707873	DEL	chr18_13628273_13628688	2.051004	9.90398734	9.1217E-06	0.0335112	H3K27ac	LD
chr1	50934752	50934983	DEL	chr1_50967474_50968103	1.32277681	9.92887291	8.9526E-06	0.0335112	H3K27ac	LD

chr13	50463131	50463132	INS	chr13_50767791_50768016	1.63741428	9.9157608	9.0413E-06	0.0335112	H3K27ac	LD
chr14	125286621	125286622	INS	chr14_125158247_125159911	-5.69599	-9.9079598	9.0945E-06	0.0335112	H3K27ac	LD
chr14	125346834	125346835	INS	chr14_125158247_125159911	-5.69599	-9.9079598	9.0945E-06	0.0335112	H3K27ac	LD
chr14	125449978	125449979	INS	chr14_125158247_125159911	-5.69599	-9.9079598	9.0945E-06	0.0335112	H3K27ac	LD
chr14	125461758	125461759	INS	chr14_125158247_125159911	-5.69599	-9.9079598	9.0945E-06	0.0335112	H3K27ac	LD
chr14	125661993	125661994	INS	chr14_125158247_125159911	-5.69599	-9.9079598	9.0945E-06	0.0335112	H3K27ac	LD
chr9	113718505	113718506	INS	chr9_113755012_113756003	4.66723403	9.91847915	9.0228E-06	0.0335112	H3K27ac	LD
chr9	113807086	113807087	INS	chr9_113755012_113756003	4.66723403	9.91847915	9.0228E-06	0.0335112	H3K27ac	LD
chr9	114086853	114086854	INS	chr9_113755012_113756003	4.66723403	9.91847915	9.0228E-06	0.0335112	H3K27ac	LD
chr9	114151564	114151565	INS	chr9_113755012_113756003	4.66723403	9.91847915	9.0228E-06	0.0335112	H3K27ac	LD
chr1	6949592	6949593	INS	chr1_7411849_7413820	5.307906	9.9232406	8.9906E-06	0.0335112	H3K27ac	LD
chr6	19291472	19291473	INS	chr6_18889940_18890658	2.10979839	9.90292193	9.129E-06	0.0335112	H3K27ac	LD
chr18	13320728	13320729	INS	chr18_13628273_13628688	2.051004	9.90398734	9.1217E-06	0.0335112	H3K27ac	LD

chr18	13470269	13470270	INS	chr18_13628273_13628688	2.051004	9.90398734	9.1217E-06	0.0335112	H3K27ac	LD
chr18	13832932	13832933	INS	chr18_13628273_13628688	2.051004	9.90398734	9.1217E-06	0.0335112	H3K27ac	LD
chr11	21890279	21890280	INS	chr11_22071765_22072467	0.8350735	9.908039	9.0939E-06	0.0335112	H3K27ac	LD
chr1	50931319	50931320	INS	chr1_50967474_50968103	1.32277681	9.92887291	8.9526E-06	0.0335112	H3K27ac	LD
chr17	46495345	46495346	INS	chr17_46621853_46622524	16.0784112	9.89870764	9.1581E-06	0.03357565	H3K27ac	LD
chr15	136234708	136235011	DEL	chr15_136185913_136186591	4.12590938	9.89206122	9.204E-06	0.0337231	H3K27ac	LD
chr3	23941095	23941096	INS	chr3_24195179_24195686	1.33026875	9.88688081	9.24E-06	0.03383389	H3K27ac	LD
chr14	99251435	99251436	INS	chr14_99379319_99379515	-1.38222	-9.8835509	9.2633E-06	0.03387661	H3K27ac	LD
chr14	99287018	99287019	INS	chr14_99379319_99379515	-1.38222	-9.8835509	9.2633E-06	0.03387661	H3K27ac	LD
chr7	8684021	8684669	DEL	chr7_8628799_8629034	1.30209931	9.87668571	9.3114E-06	0.03392546	H3K27ac	LD
chr7	8399769	8399770	INS	chr7_8628799_8629034	1.30209931	9.87668571	9.3114E-06	0.03392546	H3K27ac	LD
chr7	8495036	8495037	INS	chr7_8628799_8629034	1.30209931	9.87668571	9.3114E-06	0.03392546	H3K27ac	LD
chr7	8683557	8683558	INS	chr7_8628799_8629034	1.30209931	9.87668571	9.3114E-06	0.03392546	H3K27ac	LD

chr4	18741704	18741705	INS	chr4_18650430_18650723	1.79862667	9.87800103	9.3021E-06	0.03392546	H3K27ac	LD
chr8	104776404	104776404	BND	chr8_104962907_104963475	2.28257917	9.86129692	9.4202E-06	0.03397688	H3K27ac	LD
chr6	69269480	69269618	DEL	chr6_69056182_69056741	4.40604778	9.86409956	9.4003E-06	0.03397688	H3K27ac	LD
chr8	104741823	104751060	DEL	chr8_104962907_104963475	2.28257917	9.86129692	9.4202E-06	0.03397688	H3K27ac	LD
chr10	17075042	17076319	DEL	chr10_17520090_17523039	1.74461833	9.84943361	9.5051E-06	0.03397688	H3K27ac	LD
chr10	17429299	17429637	DEL	chr10_17520090_17523039	1.74461833	9.84943361	9.5051E-06	0.03397688	H3K27ac	LD
chr10	17509680	17510141	DEL	chr10_17520090_17523039	1.74461833	9.84943361	9.5051E-06	0.03397688	H3K27ac	LD
chr10	17510688	17510754	DEL	chr10_17520090_17523039	1.74461833	9.84943361	9.5051E-06	0.03397688	H3K27ac	LD
chr10	17540777	17541013	DEL	chr10_17520090_17523039	1.74461833	9.84943361	9.5051E-06	0.03397688	H3K27ac	LD
chr10	17920212	17920426	DEL	chr10_17520090_17523039	1.74461833	9.84943361	9.5051E-06	0.03397688	H3K27ac	LD
chr4	99920033	99920034	INS	chr4_99549144_99549618	3.85307894	9.86713554	9.3788E-06	0.03397688	H3K27ac	LD
chr8	104776389	104776390	INS	chr8_104962907_104963475	2.28257917	9.86129692	9.4202E-06	0.03397688	H3K27ac	LD
chr8	105277849	105277850	INS	chr8_104962907_104963475	2.28257917	9.86129692	9.4202E-06	0.03397688	H3K27ac	LD

chr5	11169916	11169917	INS	chr5_11563505_11564015	1.83426708	9.8602129	9.4279E-06	0.03397688	H3K27ac	LD
chr5	11257494	11257495	INS	chr5_11563505_11564015	1.83426708	9.8602129	9.4279E-06	0.03397688	H3K27ac	LD
chr5	11323246	11323247	INS	chr5_11563505_11564015	1.83426708	9.8602129	9.4279E-06	0.03397688	H3K27ac	LD
chr5	11471186	11471187	INS	chr5_11563505_11564015	1.83426708	9.8602129	9.4279E-06	0.03397688	H3K27ac	LD
chr4	18227611	18227612	INS	chr4_18226681_18227505	1.37845744	9.84940877	9.5053E-06	0.03397688	H3K27ac	LD
chr10	17201316	17201317	INS	chr10_17520090_17523039	1.74461833	9.84943361	9.5051E-06	0.03397688	H3K27ac	LD
chr10	17354643	17354644	INS	chr10_17520090_17523039	1.74461833	9.84943361	9.5051E-06	0.03397688	H3K27ac	LD
chr10	17552279	17552280	INS	chr10_17520090_17523039	1.74461833	9.84943361	9.5051E-06	0.03397688	H3K27ac	LD
chr10	17579706	17579707	INS	chr10_17520090_17523039	1.74461833	9.84943361	9.5051E-06	0.03397688	H3K27ac	LD
chr10	17568625	17568626	INS	chr10_17520090_17523039	1.74461833	9.84943361	9.5051E-06	0.03397688	H3K27ac	LD
chr10	17683188	17683189	INS	chr10_17520090_17523039	1.74461833	9.84943361	9.5051E-06	0.03397688	H3K27ac	LD
chr10	18007463	18007464	INS	chr10_17520090_17523039	1.74461833	9.84943361	9.5051E-06	0.03397688	H3K27ac	LD
chr6	64685134	64685203	DEL	chr6_64423668_64424438	1.3996112	9.84635498	9.5273E-06	0.0340347	H3K27ac	LD

chr10	29219307	29219985	DEL	chr10_29278893_29279670	1.45247764	9.82878102	9.6549E-06	0.0341225	H3K27ac	LD
chr10	29252782	29252847	DEL	chr10_29278893_29279670	1.45247764	9.82878102	9.6549E-06	0.0341225	H3K27ac	LD
chr10	29236831	29237132	DEL	chr10_29278893_29279670	1.45247764	9.82878102	9.6549E-06	0.0341225	H3K27ac	LD
chr10	29305994	29306282	DEL	chr10_29278893_29279670	1.45247764	9.82878102	9.6549E-06	0.0341225	H3K27ac	LD
chr10	29323450	29323504	DEL	chr10_29278893_29279670	1.45247764	9.82878102	9.6549E-06	0.0341225	H3K27ac	LD
chr10	29331858	29331910	DEL	chr10_29278893_29279670	1.45247764	9.82878102	9.6549E-06	0.0341225	H3K27ac	LD
chr4	55463561	55464047	DEL	chr4_55584686_55585424	1.0877216	9.83574496	9.6041E-06	0.0341225	H3K27ac	LD
chr4	55859601	55859602	INS	chr4_55584686_55585424	1.0877216	9.83574496	9.6041E-06	0.0341225	H3K27ac	LD
chr10	29254940	29254941	INS	chr10_29278893_29279670	1.45247764	9.82878102	9.6549E-06	0.0341225	H3K27ac	LD
chr10	29249561	29249562	INS	chr10_29278893_29279670	1.45247764	9.82878102	9.6549E-06	0.0341225	H3K27ac	LD
chr10	29264527	29264528	INS	chr10_29278893_29279670	1.45247764	9.82878102	9.6549E-06	0.0341225	H3K27ac	LD
chr10	29237740	29237741	INS	chr10_29278893_29279670	1.45247764	9.82878102	9.6549E-06	0.0341225	H3K27ac	LD
chr10	29317490	29317491	INS	chr10_29278893_29279670	1.45247764	9.82878102	9.6549E-06	0.0341225	H3K27ac	LD

chr10	29302512	29302513	INS	chr10_29278893_29279670	1.45247764	9.82878102	9.6549E-06	0.0341225	H3K27ac	LD
chr10	29359175	29359176	INS	chr10_29278893_29279670	1.45247764	9.82878102	9.6549E-06	0.0341225	H3K27ac	LD
chr2	95963526	95963527	INS	chr2_96395705_96396093	1.08388681	9.82853817	9.6567E-06	0.0341225	H3K27ac	LD
chr18	757952	757953	INS	chr18_744155_745115	-3.3039159	-9.8410655	9.5655E-06	0.0341225	H3K27ac	LD
chr13	11442530	11442581	DEL	chr13_11315397_11316587	1.157287	9.82751149	9.6642E-06	0.03412847	H3K27ac	LD
chr1	14952680	14952855	DEL	chr1_15366712_15367414	1.93013928	9.81582597	9.7502E-06	0.03428756	H3K27ac	LD
chr1	15077830	15078730	DEL	chr1_15366712_15367414	1.93013928	9.81582597	9.7502E-06	0.03428756	H3K27ac	LD
chr1	15370506	15370807	DEL	chr1_15366712_15367414	1.93013928	9.81582597	9.7502E-06	0.03428756	H3K27ac	LD
chr9	6523301	6523302	INS	chr9_6156749_6157547	3.73405913	9.81800581	9.7341E-06	0.03428756	H3K27ac	LD
chr1	15531394	15531395	INS	chr1_15366712_15367414	1.93013928	9.81582597	9.7502E-06	0.03428756	H3K27ac	LD
chr2	137512073	137512139	DEL	chr2_137559674_137560216	2.17813944	9.80895449	9.8012E-06	0.03436371	H3K27ac	LD
chr17	34934745	34934746	INS	chr17_35282032_35282489	2.70855611	9.80967085	9.7959E-06	0.03436371	H3K27ac	LD
chr2	137261298	137261299	INS	chr2_137559674_137560216	2.17813944	9.80895449	9.8012E-06	0.03436371	H3K27ac	LD

chr2	137378464	137378465	INS	chr2_137559674_137560216	2.17813944	9.80895449	9.8012E-06	0.03436371	H3K27ac	LD
chr16	68565023	68565278	DEL	chr16_69018777_69019787	0.77905771	9.79994597	9.8685E-06	0.03448381	H3K27ac	LD
chr8	6221001	6221106	DEL	chr8_6419379_6420175	3.50279875	9.80192006	9.8537E-06	0.03448381	H3K27ac	LD
chr3	12824777	12825047	DEL	chr3_13134190_13134625	2.12907733	9.78947705	9.9473E-06	0.03448381	H3K27ac	LD
chr3	17853270	17853392	DEL	chr3_17531269_17532026	0.80161483	9.79856053	9.8789E-06	0.03448381	H3K27ac	LD
chr18	37017706	37017755	DEL	chr18_36885986_36886893	2.30264256	9.7925987	9.9237E-06	0.03448381	H3K27ac	LD
chr16	68618958	68618959	INS	chr16_69018777_69019787	0.77905771	9.79994597	9.8685E-06	0.03448381	H3K27ac	LD
chr8	109887415	109887416	INS	chr8_109779641_109781515	0.91507286	9.78551507	9.9773E-06	0.03448381	H3K27ac	LD
chr8	110047140	110047141	INS	chr8_109779641_109781515	0.91507286	9.78551507	9.9773E-06	0.03448381	H3K27ac	LD
chr8	110239854	110239855	INS	chr8_109779641_109781515	0.91507286	9.78551507	9.9773E-06	0.03448381	H3K27ac	LD
chr8	6501622	6501623	INS	chr8_6419379_6420175	3.50279875	9.80192006	9.8537E-06	0.03448381	H3K27ac	LD
chr3	123091607	123091608	INS	chr3_123150619_123151006	1.09568076	9.7975635	9.8863E-06	0.03448381	H3K27ac	LD
chr3	126321732	126321733	INS	chr3_126716302_126716619	1.17166333	9.78404797	9.9885E-06	0.03448381	H3K27ac	LD

chr3	126643110	126643111	INS	chr3_126716302_126716619	1.17166333	9.78404797	9.9885E-06	0.03448381	H3K27ac	LD
chr3	126720278	126720279	INS	chr3_126716302_126716619	1.17166333	9.78404797	9.9885E-06	0.03448381	H3K27ac	LD
chr3	126758422	126758423	INS	chr3_126716302_126716619	1.17166333	9.78404797	9.9885E-06	0.03448381	H3K27ac	LD
chr3	126822583	126822584	INS	chr3_126716302_126716619	1.17166333	9.78404797	9.9885E-06	0.03448381	H3K27ac	LD
chr3	126911164	126911165	INS	chr3_126716302_126716619	1.17166333	9.78404797	9.9885E-06	0.03448381	H3K27ac	LD
chr3	127057703	127057704	INS	chr3_126716302_126716619	1.17166333	9.78404797	9.9885E-06	0.03448381	H3K27ac	LD
chr3	12748581	12748582	INS	chr3_13134190_13134625	2.12907733	9.78947705	9.9473E-06	0.03448381	H3K27ac	LD
chr3	12747472	12747473	INS	chr3_13134190_13134625	2.12907733	9.78947705	9.9473E-06	0.03448381	H3K27ac	LD
chr3	12818799	12818800	INS	chr3_13134190_13134625	2.12907733	9.78947705	9.9473E-06	0.03448381	H3K27ac	LD
chr3	17841136	17841137	INS	chr3_17531269_17532026	0.80161483	9.79856053	9.8789E-06	0.03448381	H3K27ac	LD
chr18	37018401	37018402	INS	chr18_36885986_36886893	2.30264256	9.7925987	9.9237E-06	0.03448381	H3K27ac	LD
chr6	95017206	95017634	DEL	chr6_94690580_94692287	6.15988883	39.7383721	1.7686E-10	4.8375E-05	H3K27ac	LD
chr14	133145647	133145877	DEL	chr14_133212600_133213414	2.52230192	36.6259413	3.3854E-10	4.8375E-05	H3K27ac	LD

chr14	133203448	133203517	DEL	chr14_133212600_133213414	5.04460384	36.6259413	3.3854E-10	4.8375E-05	H3K27ac	LD
chr14	133232275	133232392	DEL	chr14_133212600_133213414	2.52230192	36.6259413	3.3854E-10	4.8375E-05	H3K27ac	LD
chr14	133315198	133315368	DEL	chr14_133212600_133213414	2.52230192	36.6259413	3.3854E-10	4.8375E-05	H3K27ac	LD
chr14	133432892	133433053	DEL	chr14_133212600_133213414	2.52230192	36.6259413	3.3854E-10	4.8375E-05	H3K27ac	LD
chr11	17440582	17440907	DEL	chr11_17082509_17083688	3.89683144	39.046811	2.034E-10	4.8375E-05	H3K27ac	LD
chr16	22011137	22011266	DEL	chr16_22396976_22397849	6.28641311	41.1215251	1.3467E-10	4.8375E-05	H3K27ac	LD
chr6	94726778	94726779	INS	chr6_94690580_94692287	6.15988883	39.7383721	1.7686E-10	4.8375E-05	H3K27ac	LD
chr6	94760548	94760549	INS	chr6_94690580_94692287	6.15988883	39.7383721	1.7686E-10	4.8375E-05	H3K27ac	LD
chr6	94769331	94769332	INS	chr6_94690580_94692287	6.15988883	39.7383721	1.7686E-10	4.8375E-05	H3K27ac	LD
chr6	94996088	94996089	INS	chr6_94690580_94692287	6.15988883	39.7383721	1.7686E-10	4.8375E-05	H3K27ac	LD
chr6	95112403	95112404	INS	chr6_94690580_94692287	6.15988883	39.7383721	1.7686E-10	4.8375E-05	H3K27ac	LD
chr14	133104665	133104666	INS	chr14_133212600_133213414	2.52230192	36.6259413	3.3854E-10	4.8375E-05	H3K27ac	LD
chr14	133202898	133202899	INS	chr14_133212600_133213414	2.52230192	36.6259413	3.3854E-10	4.8375E-05	H3K27ac	LD

chr14	133285066	133285067	INS	chr14_133212600_133213414	2.52230192	36.6259413	3.3854E-10	4.8375E-05	H3K27ac	LD
chr14	133519983	133519984	INS	chr14_133212600_133213414	2.52230192	36.6259413	3.3854E-10	4.8375E-05	H3K27ac	LD
chr14	133623385	133623386	INS	chr14_133212600_133213414	2.52230192	36.6259413	3.3854E-10	4.8375E-05	H3K27ac	LD
chr6	27274566	27274567	INS	chr6_27208993_27209785	3.33107828	37.4098417	2.8603E-10	4.8375E-05	H3K27ac	LD
chr6	27701019	27701020	INS	chr6_27208993_27209785	3.33107828	37.4098417	2.8603E-10	4.8375E-05	H3K27ac	LD
chr16	22618676	22618677	INS	chr16_22396976_22397849	6.28641311	41.1215251	1.3467E-10	4.8375E-05	H3K27ac	LD
chr16	22682232	22682233	INS	chr16_22396976_22397849	6.28641311	41.1215251	1.3467E-10	4.8375E-05	H3K27ac	LD
chr16	53709679	53709680	INS	chr16_53775728_53776603	2.76376189	37.0598023	3.0826E-10	4.8375E-05	H3K27ac	LD
chr12	55169082	55170053	DEL	chr12_55493955_55494974	6.13817578	54.9729107	1.3301E-11	7.7928E-06	H3K27ac	LD
chr12	55616959	55617051	DEL	chr12_55493955_55494974	6.13817578	54.9729107	1.3301E-11	7.7928E-06	H3K27ac	LD
chr15	24951939	24956849	DEL	chr15_25447691_25449944	7.20137017	55.9101073	1.1622E-11	7.7928E-06	H3K27ac	LD
chr15	25320801	25321010	DEL	chr15_25447691_25449944	7.20137017	55.9101073	1.1622E-11	7.7928E-06	H3K27ac	LD
chr6	36644597	36644737	DEL	chr6_36300027_36300298	2.16220372	66.1233922	3.0445E-12	7.7928E-06	H3K27ac	LD

chr12	55609745	55609746	INS	chr12_55493955_55494974	6.13817578	54.9729107	1.3301E-11	7.7928E-06	H3K27ac	LD
chr12	55832663	55832664	INS	chr12_55493955_55494974	6.13817578	54.9729107	1.3301E-11	7.7928E-06	H3K27ac	LD
chr15	25284197	25284198	INS	chr15_25447691_25449944	7.20137017	55.9101073	1.1622E-11	7.7928E-06	H3K27ac	LD
chr17	56132480	56132481	INS	chr17_55970834_55971768	5.97621517	34.1144891	5.9566E-10	8.1157E-05	H3K27ac	LD
chr17	56243816	56243817	INS	chr17_55970834_55971768	5.97621517	34.1144891	5.9566E-10	8.1157E-05	H3K27ac	LD
chr1	269801124	269807111	DUP	chr1_269804204_269806833	9.22622125	33.4993908	6.8836E-10	0.0008209	H3K27me3	LD
chr4	2071948	2072223	DEL	chr4_2175144_2175983	5.13522778	25.9415349	5.2333E-09	0.00208031	H3K27me3	LD
chr9	132210042	132210043	INS	chr9_132210367_132210791	6.10971611	27.2198825	3.5755E-09	0.00208031	H3K27me3	LD
chr7	23981736	23981737	INS	chr7_23921764_23922313	4.83109056	27.8408606	2.9902E-09	0.00208031	H3K27me3	LD
chr4	2349812	2349813	INS	chr4_2175144_2175983	5.13522778	25.9415349	5.2333E-09	0.00208031	H3K27me3	LD
chr11	2929403	2929403	BND	chr11_2928489_2929202	6.78734222	22.5598602	1.5781E-08	0.00313663	H3K27me3	LD
chr11	2796284	2796586	DEL	chr11_2928489_2929202	6.78734222	22.5598602	1.5781E-08	0.00313663	H3K27me3	LD
chr11	3028105	3028695	DEL	chr11_2928489_2929202	6.78734222	22.5598602	1.5781E-08	0.00313663	H3K27me3	LD

chr16	69008886	69008887	INS	chr16_68806077_68806388	2.70515706	22.7606661	1.4716E-08	0.00313663	H3K27me3	LD
chr11	2929402	2929403	INS	chr11_2928489_2929202	6.78734222	22.5598602	1.5781E-08	0.00313663	H3K27me3	LD
chr3	44371	44372	INS	chr3_57841_58609	-5.8526716	-23.192782	1.2686E-08	0.00313663	H3K27me3	LD
chr4	90810925	90810925	BND	chr4_90809640_90810615	1.364504	20.6968571	3.1121E-08	0.00494838	H3K27me3	LD
chr4	90684032	90684033	INS	chr4_90809640_90810615	1.364504	20.6968571	3.1121E-08	0.00494838	H3K27me3	LD
chr11	5099557	5099558	INS	chr11_5097744_5098237	4.71829228	19.6799976	4.6243E-08	0.00689338	H3K27me3	LD
chr15	25387199	25387199	BND	chr15_25365600_25365895	4.73328133	18.2464903	8.3686E-08	0.00998899	H3K27me3	LD
chr11	6394079	6395867	DEL	chr11_6309366_6310521	4.66350805	18.3414694	8.0351E-08	0.00998899	H3K27me3	LD
chr14	10688812	10688813	INS	chr14_10689038_10689664	3.63201878	18.1483676	8.7295E-08	0.00998899	H3K27me3	LD
chr11	5099557	5099558	INS	chr11_5097035_5097357	4.16843778	17.7607106	1.0336E-07	0.01120607	H3K27me3	LD
chr1	132677507	132677507	BND	chr1_132677751_132679461	4.30627567	15.2185715	3.4444E-07	0.01298009	H3K27me3	LD
chr3	33308009	33308009	BND	chr3_33306743_33307786	6.47371746	15.3601865	3.2055E-07	0.01298009	H3K27me3	LD
chr4	81337064	81337359	DEL	chr4_81241599_81241919	-2.787101	-16.063146	2.2637E-07	0.01298009	H3K27me3	LD

chr4	81408593	81408868	DEL	chr4_81241599_81241919	-2.787101	-16.063146	2.2637E-07	0.01298009	H3K27me3	LD
chr1	142151990	142152063	DEL	chr1_142244315_142245218	-2.5295462	-14.985326	3.8828E-07	0.01298009	H3K27me3	LD
chr1	142151990	142152063	DEL	chr1_142269881_142270883	-3.6593336	-14.909199	4.0391E-07	0.01298009	H3K27me3	LD
chr1	238888107	238889097	DEL	chr1_239022818_239023973	4.84746794	14.8707784	4.1207E-07	0.01298009	H3K27me3	LD
chr6	13937994	13945828	DEL	chr6_13944392_13946691	4.47626475	14.8987641	4.0611E-07	0.01298009	H3K27me3	LD
chr6	13980303	13980496	DEL	chr6_13944392_13946691	4.47626475	14.8987641	4.0611E-07	0.01298009	H3K27me3	LD
chr6	14034868	14035150	DEL	chr6_13944392_13946691	4.47626475	14.8987641	4.0611E-07	0.01298009	H3K27me3	LD
chr1	15964303	15964597	DEL	chr1_16056729_16059254	-9.212584	-15.652902	2.7683E-07	0.01298009	H3K27me3	LD
chr2	45579565	45579883	DEL	chr2_45605974_45607953	4.80331938	14.5875665	4.7823E-07	0.01298009	H3K27me3	LD
chr2	45586949	45587222	DEL	chr2_45605974_45607953	4.80331938	14.5875665	4.7823E-07	0.01298009	H3K27me3	LD
chr2	45588150	45589844	DEL	chr2_45605974_45607953	4.80331938	14.5875665	4.7823E-07	0.01298009	H3K27me3	LD
chr2	45595976	45596276	DEL	chr2_45605974_45607953	4.80331938	14.5875665	4.7823E-07	0.01298009	H3K27me3	LD
chr2	45588150	45588287	DEL	chr2_45605974_45607953	4.80331938	14.5875665	4.7823E-07	0.01298009	H3K27me3	LD

chr2	45605878	45608297	DUP	chr2_45605974_45607953	9.60663875	14.5875665	4.7823E-07	0.01298009	H3K27me3	LD
chr13	24271694	24271695	INS	chr13_24266285_24266611	5.49030378	17.1245619	1.3746E-07	0.01298009	H3K27me3	LD
chr4	71085769	71085770	INS	chr4_71281328_71282111	1.73894433	15.1579311	3.5528E-07	0.01298009	H3K27me3	LD
chr4	71282290	71282291	INS	chr4_71281328_71282111	1.73894433	15.1579311	3.5528E-07	0.01298009	H3K27me3	LD
chr16	72923737	72923738	INS	chr16_72727722_72728076	4.41633167	14.5636074	4.8435E-07	0.01298009	H3K27me3	LD
chr17	47642135	47642136	INS	chr17_47639773_47640798	8.207445	15.2000751	3.4771E-07	0.01298009	H3K27me3	LD
chr17	47680666	47680667	INS	chr17_47639773_47640798	8.207445	15.2000751	3.4771E-07	0.01298009	H3K27me3	LD
chr10	50363015	50363016	INS	chr10_50369940_50370170	2.71085544	15.9773683	2.36E-07	0.01298009	H3K27me3	LD
chr2	82423296	82423297	INS	chr2_82422326_82422857	5.18242	16.8830369	1.5358E-07	0.01298009	H3K27me3	LD
chr4	105442602	105442603	INS	chr4_105442780_105443939	5.60076	14.6319751	4.6711E-07	0.01298009	H3K27me3	LD
chr13	76699009	76699010	INS	chr13_76696699_76698783	4.99743656	14.6981655	4.5107E-07	0.01298009	H3K27me3	LD
chr4	109047137	109047138	INS	chr4_109035359_109035809	6.31223028	14.9319083	3.9918E-07	0.01298009	H3K27me3	LD
chr4	109035259	109035260	INS	chr4_109035359_109035809	6.31223028	14.9319083	3.9918E-07	0.01298009	H3K27me3	LD

chr4	109061859	109061860	INS	chr4_109035359_109035809	6.31223028	14.9319083	3.9918E-07	0.01298009	H3K27me3	LD
chr13	80115878	80115879	INS	chr13_80116155_80116805	2.383458	16.2958005	2.0239E-07	0.01298009	H3K27me3	LD
chr1	132677507	132677508	INS	chr1_132677751_132679461	2.15313783	15.2185715	3.4444E-07	0.01298009	H3K27me3	LD
chr1	9280049	9280050	INS	chr1_9385234_9385760	1.90771643	15.7608883	2.6242E-07	0.01298009	H3K27me3	LD
chr1	9367431	9367432	INS	chr1_9385234_9385760	1.90771643	15.7608883	2.6242E-07	0.01298009	H3K27me3	LD
chr1	9397187	9397188	INS	chr1_9385234_9385760	1.90771643	15.7608883	2.6242E-07	0.01298009	H3K27me3	LD
chr1	238851630	238851631	INS	chr1_239022818_239023973	4.84746794	14.8707784	4.1207E-07	0.01298009	H3K27me3	LD
chr1	239084795	239084796	INS	chr1_239022818_239023973	4.84746794	14.8707784	4.1207E-07	0.01298009	H3K27me3	LD
chr1	254272045	254272046	INS	chr1_254352112_254352895	0.60705167	14.6314903	4.6723E-07	0.01298009	H3K27me3	LD
chr6	13937706	13937707	INS	chr6_13944392_13946691	4.47626475	14.8987641	4.0611E-07	0.01298009	H3K27me3	LD
chr6	13967501	13967502	INS	chr6_13944392_13946691	4.47626475	14.8987641	4.0611E-07	0.01298009	H3K27me3	LD
chr6	14102684	14102685	INS	chr6_13944392_13946691	4.47626475	14.8987641	4.0611E-07	0.01298009	H3K27me3	LD
chr1	16154637	16154638	INS	chr1_16056729_16059254	-9.212584	-15.652902	2.7683E-07	0.01298009	H3K27me3	LD

chr6	19291472	19291473	INS	chr6_19292622_19293041	4.70352389	14.5894652	4.7775E-07	0.01298009	H3K27me3	LD
chr15	19813940	19813941	INS	chr15_19813072_19813653	2.49156561	15.7013726	2.7026E-07	0.01298009	H3K27me3	LD
chr15	19912917	19912918	INS	chr15_19813072_19813653	2.49156561	15.7013726	2.7026E-07	0.01298009	H3K27me3	LD
chr7	24288655	24288656	INS	chr7_24335570_24336528	-6.096075	-15.239864	3.4073E-07	0.01298009	H3K27me3	LD
chr14	19017454	19017455	INS	chr14_19155485_19156682	1.78522146	14.566239	4.8368E-07	0.01298009	H3K27me3	LD
chr14	19020953	19020954	INS	chr14_19155485_19156682	1.78522146	14.566239	4.8368E-07	0.01298009	H3K27me3	LD
chr14	19035700	19035701	INS	chr14_19155485_19156682	1.78522146	14.566239	4.8368E-07	0.01298009	H3K27me3	LD
chr14	19156856	19156857	INS	chr14_19155485_19156682	1.78522146	14.566239	4.8368E-07	0.01298009	H3K27me3	LD
chr3	33433527	33433528	INS	chr3_33306743_33307786	6.47371746	15.3601865	3.2055E-07	0.01298009	H3K27me3	LD
chr2	45555215	45555216	INS	chr2_45605974_45607953	4.80331938	14.5875665	4.7823E-07	0.01298009	H3K27me3	LD
chr2	45548726	45548727	INS	chr2_45605974_45607953	4.80331938	14.5875665	4.7823E-07	0.01298009	H3K27me3	LD
chr2	45570410	45570411	INS	chr2_45605974_45607953	4.80331938	14.5875665	4.7823E-07	0.01298009	H3K27me3	LD
chr2	45565638	45565639	INS	chr2_45605974_45607953	4.80331938	14.5875665	4.7823E-07	0.01298009	H3K27me3	LD

chr2	45591258	45591259	INS	chr2_45605974_45607953	4.80331938	14.5875665	4.7823E-07	0.01298009	H3K27me3	LD
chr2	45650548	45650549	INS	chr2_45605974_45607953	4.80331938	14.5875665	4.7823E-07	0.01298009	H3K27me3	LD
chr2	45616793	45616794	INS	chr2_45605974_45607953	4.80331938	14.5875665	4.7823E-07	0.01298009	H3K27me3	LD
chr2	45717532	45717533	INS	chr2_45605974_45607953	4.80331938	14.5875665	4.7823E-07	0.01298009	H3K27me3	LD
chr2	45721112	45721113	INS	chr2_45605974_45607953	4.80331938	14.5875665	4.7823E-07	0.01298009	H3K27me3	LD
chr2	45719661	45719662	INS	chr2_45605974_45607953	4.80331938	14.5875665	4.7823E-07	0.01298009	H3K27me3	LD
chr2	45802510	45802511	INS	chr2_45605974_45607953	4.80331938	14.5875665	4.7823E-07	0.01298009	H3K27me3	LD
chr14	31961311	31961446	DEL	chr14_31807851_31808253	3.65092588	14.4269539	5.21E-07	0.01380695	H3K27me3	LD
chr13	199753408	199753489	DEL	chr13_199646840_199647328	-3.032489	-14.241464	5.7581E-07	0.01476726	H3K27me3	LD
chr6	4054046	4054047	INS	chr6_4201794_4202493	1.86096556	14.2424396	5.755E-07	0.01476726	H3K27me3	LD
chr13	199506572	199506573	INS	chr13_199646840_199647328	-3.032489	-14.241464	5.7581E-07	0.01476726	H3K27me3	LD
chr11	6309245	6309245	BND	chr11_6311074_6311994	6.28672625	14.1410466	6.0816E-07	0.01477676	H3K27me3	LD
chr11	6395911	6397205	DEL	chr11_6311074_6311994	6.28672625	14.1410466	6.0816E-07	0.01477676	H3K27me3	LD

chr11	6398467	6398694	DEL	chr11_6311074_6311994	6.28672625	14.1410466	6.0816E-07	0.01477676	H3K27me3	LD
chr11	6397225	6398198	DEL	chr11_6311074_6311994	6.28672625	14.1410466	6.0816E-07	0.01477676	H3K27me3	LD
chr6	66346085	66346086	INS	chr6_66346285_66346888	3.47142757	14.1071123	6.1955E-07	0.01477676	H3K27me3	LD
chr11	6412411	6412412	INS	chr11_6311074_6311994	3.14336313	14.1410466	6.0816E-07	0.01477676	H3K27me3	LD
chr4	108822503	108822504	INS	chr4_108842307_108843459	2.88377372	13.9443228	6.7761E-07	0.01569092	H3K27me3	LD
chr4	108843551	108843552	INS	chr4_108842307_108843459	2.88377372	13.9443228	6.7761E-07	0.01569092	H3K27me3	LD
chr4	106284611	106284611	BND	chr4_106284860_106286344	3.87636625	13.7483235	7.5577E-07	0.01637593	H3K27me3	LD
chr4	106284588	106284588	BND	chr4_106284860_106286344	7.7527325	13.7483235	7.5577E-07	0.01637593	H3K27me3	LD
chr16	70688055	70689562	DEL	chr16_70617899_70618647	3.22852083	13.7174229	7.6899E-07	0.01637593	H3K27me3	LD
chr16	70515771	70515772	INS	chr16_70617899_70618647	3.22852083	13.7174229	7.6899E-07	0.01637593	H3K27me3	LD
chr16	70638770	70638771	INS	chr16_70617899_70618647	3.22852083	13.7174229	7.6899E-07	0.01637593	H3K27me3	LD
chr16	70618746	70618747	INS	chr16_70617899_70618647	3.22852083	13.7174229	7.6899E-07	0.01637593	H3K27me3	LD
chr16	70797627	70797628	INS	chr16_70617899_70618647	3.22852083	13.7174229	7.6899E-07	0.01637593	H3K27me3	LD

chr4	106284588	106284589	INS	chr4_106284860_106286344	3.87636625	13.7483235	7.5577E-07	0.01637593	H3K27me3	LD
chr14	54500481	54500482	INS	chr14_54500640_54501721	3.23792111	13.7986713	7.3477E-07	0.01637593	H3K27me3	LD
chr10	47001649	47001650	INS	chr10_47001953_47002689	5.70625989	13.569563	8.3594E-07	0.01764417	H3K27me3	LD
chr6	38008682	38008683	INS	chr6_38009235_38010535	2.73588278	13.5169991	8.6129E-07	0.01801988	H3K27me3	LD
chr9	45354914	45354915	INS	chr9_45403011_45403275	2.25972375	13.4726783	8.8334E-07	0.0183205	H3K27me3	LD
chr15	50886728	50886729	INS	chr15_50885795_50886089	2.60898875	13.4383742	9.0084E-07	0.0185224	H3K27me3	LD
chr13	72837751	72837752	INS	chr13_72839050_72840140	7.12738111	13.3141851	9.6753E-07	0.01972346	H3K27me3	LD
chr9	58416151	58416152	INS	chr9_58416275_58418068	3.94046319	13.2548172	1.0013E-06	0.02023985	H3K27me3	LD
chr4	81337064	81337359	DEL	chr4_81237051_81237853	-2.47056	-13.214679	1.025E-06	0.02037185	H3K27me3	LD
chr4	81408593	81408868	DEL	chr4_81237051_81237853	-2.47056	-13.214679	1.025E-06	0.02037185	H3K27me3	LD
chr17	50166005	50166335	DEL	chr17_50331030_50331320	-7.46489	-13.126757	1.0789E-06	0.02075211	H3K27me3	LD
chr17	50180153	50181003	DEL	chr17_50331030_50331320	-7.46489	-13.126757	1.0789E-06	0.02075211	H3K27me3	LD
chr17	50472659	50472804	DEL	chr17_50331030_50331320	-7.46489	-13.126757	1.0789E-06	0.02075211	H3K27me3	LD

chr17	50408467	50408468	INS	chr17_50331030_50331320	-7.46489	-13.126757	1.0789E-06	0.02075211	H3K27me3	LD
chr18	34057770	34060287	DEL	chr18_33935906_33936309	1.93098767	13.0833602	1.1067E-06	0.02111631	H3K27me3	LD
chr17	28302133	28302133	BND	chr17_28302235_28303999	8.1716775	12.772065	1.3312E-06	0.02130904	H3K27me3	LD
chr18	49127000	49127110	DEL	chr18_49261616_49262017	4.72772667	12.8541531	1.2674E-06	0.02130904	H3K27me3	LD
chr18	49357122	49357300	DEL	chr18_49261616_49262017	2.36386333	12.8541531	1.2674E-06	0.02130904	H3K27me3	LD
chr17	50166005	50166335	DEL	chr17_50323827_50325964	-3.283424	-12.776814	1.3274E-06	0.02130904	H3K27me3	LD
chr17	50180153	50181003	DEL	chr17_50323827_50325964	-3.283424	-12.776814	1.3274E-06	0.02130904	H3K27me3	LD
chr17	50472659	50472804	DEL	chr17_50323827_50325964	-3.283424	-12.776814	1.3274E-06	0.02130904	H3K27me3	LD
chr13	71054424	71054836	DEL	chr13_71093463_71094538	1.73617063	12.8280265	1.2873E-06	0.02130904	H3K27me3	LD
chr13	71241078	71244292	DEL	chr13_71093463_71094538	1.73617063	12.8280265	1.2873E-06	0.02130904	H3K27me3	LD
chr4	107359450	107359576	DEL	chr4_107272366_107273944	3.20299872	12.9862938	1.1718E-06	0.02130904	H3K27me3	LD
chr4	107390606	107391056	DEL	chr4_107272366_107273944	3.20299872	12.9862938	1.1718E-06	0.02130904	H3K27me3	LD
chr4	107397494	107397785	DEL	chr4_107272366_107273944	3.20299872	12.9862938	1.1718E-06	0.02130904	H3K27me3	LD

chr18	40880195	40880481	DEL	chr18_40957758_40957982	2.703268	12.9236967	1.216E-06	0.02130904	H3K27me3	LD
chr18	41072233	41072511	DEL	chr18_40957758_40957982	2.703268	12.9236967	1.216E-06	0.02130904	H3K27me3	LD
chr18	49125075	49125076	INS	chr18_49261616_49262017	2.36386333	12.8541531	1.2674E-06	0.02130904	H3K27me3	LD
chr18	49355645	49355646	INS	chr18_49261616_49262017	2.36386333	12.8541531	1.2674E-06	0.02130904	H3K27me3	LD
chr16	71040729	71040730	INS	chr16_71213211_71213617	2.31043833	13.0366683	1.1375E-06	0.02130904	H3K27me3	LD
chr17	50408467	50408468	INS	chr17_50323827_50325964	-3.283424	-12.776814	1.3274E-06	0.02130904	H3K27me3	LD
chr4	107274417	107274418	INS	chr4_107272366_107273944	3.20299872	12.9862938	1.1718E-06	0.02130904	H3K27me3	LD
chr1	247300514	247300515	INS	chr1_247362487_247362819	1.58599639	12.7813632	1.3238E-06	0.02130904	H3K27me3	LD
chr1	247398375	247398376	INS	chr1_247362487_247362819	1.58599639	12.7813632	1.3238E-06	0.02130904	H3K27me3	LD
chr18	40908577	40908578	INS	chr18_40957758_40957982	2.703268	12.9236967	1.216E-06	0.02130904	H3K27me3	LD
chr18	40977394	40977395	INS	chr18_40957758_40957982	2.703268	12.9236967	1.216E-06	0.02130904	H3K27me3	LD
chr18	41156958	41156959	INS	chr18_40957758_40957982	2.703268	12.9236967	1.216E-06	0.02130904	H3K27me3	LD
chr1	185066229	185066230	INS	chr1_185143906_185144574	3.35935709	12.6681749	1.4172E-06	0.02238441	H3K27me3	LD

chr1	185143689	185143690	INS	chr1_185143906_185144574	3.35935709	12.6681749	1.4172E-06	0.02238441	H3K27me3	LD
chr9	2350690	2350691	INS	chr9_2340898_2342155	4.75519583	12.5894423	1.4864E-06	0.02332424	H3K27me3	LD
chr7	24288655	24288656	INS	chr7_24286019_24287260	-3.977191	-12.424783	1.6439E-06	0.02562692	H3K27me3	LD
chr2	82318676	82318676	BND	chr2_82318119_82318534	3.96160006	12.2934572	1.783E-06	0.02657853	H3K27me3	LD
chr13	125128469	125128469	BND	chr13_125128691_125129161	3.69079378	12.3023498	1.7732E-06	0.02657853	H3K27me3	LD
chr3	46455694	46455980	DEL	chr3_46431353_46432085	-3.0430988	-12.33689	1.7356E-06	0.02657853	H3K27me3	LD
chr3	46470801	46471101	DEL	chr3_46431353_46432085	-3.0430988	-12.33689	1.7356E-06	0.02657853	H3K27me3	LD
chr2	82167441	82167442	INS	chr2_82318119_82318534	3.96160006	12.2934572	1.783E-06	0.02657853	H3K27me3	LD
chr2	82318659	82318660	INS	chr2_82318119_82318534	3.96160006	12.2934572	1.783E-06	0.02657853	H3K27me3	LD
chr13	125128456	125128457	INS	chr13_125128691_125129161	3.69079378	12.3023498	1.7732E-06	0.02657853	H3K27me3	LD
chr11	21494430	21494494	DEL	chr11_21619886_21620799	2.46517086	12.2196894	1.8668E-06	0.02712964	H3K27me3	LD
chr12	59690569	59690570	INS	chr12_59856316_59856636	2.182954	12.2290544	1.8559E-06	0.02712964	H3K27me3	LD
chr12	59889842	59889843	INS	chr12_59856316_59856636	2.182954	12.2290544	1.8559E-06	0.02712964	H3K27me3	LD

chr12	60052560	60052561	INS	chr12_59856316_59856636	2.182954	12.2290544	1.8559E-06	0.02712964	H3K27me3	LD
chr5	17504108	17504109	INS	chr5_17626250_17626607	8.71527417	12.2111465	1.8768E-06	0.02712964	H3K27me3	LD
chr8	71892131	71892401	DEL	chr8_72090937_72091342	2.00834208	12.0302712	2.1031E-06	0.03021731	H3K27me3	LD
chr12	52348757	52348924	DEL	chr12_52330475_52332814	2.96217133	11.9332542	2.2369E-06	0.03090897	H3K27me3	LD
chr12	52210954	52210955	INS	chr12_52330475_52332814	2.96217133	11.9332542	2.2369E-06	0.03090897	H3K27me3	LD
chr12	52324809	52324810	INS	chr12_52330475_52332814	2.96217133	11.9332542	2.2369E-06	0.03090897	H3K27me3	LD
chr12	52350329	52350330	INS	chr12_52330475_52332814	2.96217133	11.9332542	2.2369E-06	0.03090897	H3K27me3	LD
chr12	52347807	52347808	INS	chr12_52330475_52332814	2.96217133	11.9332542	2.2369E-06	0.03090897	H3K27me3	LD
chr12	52461241	52461242	INS	chr12_52330475_52332814	2.96217133	11.9332542	2.2369E-06	0.03090897	H3K27me3	LD
chr17	44561489	44561489	BND	chr17_44561686_44562597	4.17859167	11.8860449	2.3055E-06	0.03142174	H3K27me3	LD
chr17	44561498	44561499	INS	chr17_44561686_44562597	4.17859167	11.8860449	2.3055E-06	0.03142174	H3K27me3	LD
chr3	124727270	124727270	BND	chr3_124725615_124726138	7.36692178	11.7909511	2.4508E-06	0.03283968	H3K27me3	LD
chr1	43639844	43640181	DEL	chr1_43745129_43745564	1.2478375	11.7984111	2.4391E-06	0.03283968	H3K27me3	LD

chr3	124727270	124727271	INS	chr3_124725615_124726138	3.68346089	11.7909511	2.4508E-06	0.03283968	H3K27me3	LD
chr1	142751907	142759959	DEL	chr1_142750988_142751939	1.74791891	11.7447928	2.5251E-06	0.03310524	H3K27me3	LD
chr7	1433281	1434163	DEL	chr7_1432656_1433918	-4.0857213	-11.74411	2.5262E-06	0.03310524	H3K27me3	LD
chr7	1440336	1440395	DEL	chr7_1432656_1433918	-4.0857213	-11.74411	2.5262E-06	0.03310524	H3K27me3	LD
chr9	5955363	5956957	DEL	chr9_6018977_6019646	6.11094813	11.7342605	2.5423E-06	0.03313509	H3K27me3	LD
chr16	75146139	75146618	DEL	chr16_75089153_75089496	3.99688506	11.5827422	2.8063E-06	0.03454023	H3K27me3	LD
chr16	75235277	75235368	DEL	chr16_75089153_75089496	3.99688506	11.5827422	2.8063E-06	0.03454023	H3K27me3	LD
chr18	6349297	6349617	DEL	chr18_6516574_6517159	-3.0237514	-11.527065	2.9108E-06	0.03454023	H3K27me3	LD
chr18	6470402	6470695	DEL	chr18_6516574_6517159	-3.0237514	-11.527065	2.9108E-06	0.03454023	H3K27me3	LD
chr18	6525440	6525737	DEL	chr18_6516574_6517159	-3.0237514	-11.527065	2.9108E-06	0.03454023	H3K27me3	LD
chr18	6526716	6526997	DEL	chr18_6516574_6517159	-3.0237514	-11.527065	2.9108E-06	0.03454023	H3K27me3	LD
chr18	6548010	6548361	DEL	chr18_6516574_6517159	-3.0237514	-11.527065	2.9108E-06	0.03454023	H3K27me3	LD
chr18	6546699	6546880	DEL	chr18_6516574_6517159	-3.0237514	-11.527065	2.9108E-06	0.03454023	H3K27me3	LD

chr16	74962820	74962821	INS	chr16_75089153_75089496	3.99688506	11.5827422	2.8063E-06	0.03454023	H3K27me3	LD
chr16	75003347	75003348	INS	chr16_75089153_75089496	3.99688506	11.5827422	2.8063E-06	0.03454023	H3K27me3	LD
chr16	75088963	75088964	INS	chr16_75089153_75089496	3.99688506	11.5827422	2.8063E-06	0.03454023	H3K27me3	LD
chr16	75096866	75096867	INS	chr16_75089153_75089496	3.99688506	11.5827422	2.8063E-06	0.03454023	H3K27me3	LD
chr16	75237113	75237114	INS	chr16_75089153_75089496	3.99688506	11.5827422	2.8063E-06	0.03454023	H3K27me3	LD
chr18	6413826	6413827	INS	chr18_6516574_6517159	-3.0237514	-11.527065	2.9108E-06	0.03454023	H3K27me3	LD
chr18	6548591	6548592	INS	chr18_6516574_6517159	-3.0237514	-11.527065	2.9108E-06	0.03454023	H3K27me3	LD
chr18	6527255	6527256	INS	chr18_6516574_6517159	-3.0237514	-11.527065	2.9108E-06	0.03454023	H3K27me3	LD
chr18	6594513	6594514	INS	chr18_6516574_6517159	-3.0237514	-11.527065	2.9108E-06	0.03454023	H3K27me3	LD
chr1	126438599	126438600	INS	chr1_126437587_126438535	3.11795	11.5157025	2.9327E-06	0.03462748	H3K27me3	LD
NW_018084968.1	1487343	1487344	INS	NW_018084968.1_1647276_1647552	2.69083222	11.485145	2.9924E-06	0.0349864	H3K27me3	LD
chr16	72404872	72404924	DEL	chr16_72402000_72402474	3.38424194	11.4485637	3.0657E-06	0.0356046	H3K27me3	LD
chr5	88001236	88001698	DEL	chr5_88121966_88122358	1.68617672	11.3899795	3.1874E-06	0.0356046	H3K27me3	LD

chr12	11149783	11150064	DEL	chr12_11164504_11165342	2.87236588	11.390562	3.1861E-06	0.0356046	H3K27me3	LD
chr12	11155526	11155823	DEL	chr12_11164504_11165342	2.87236588	11.390562	3.1861E-06	0.0356046	H3K27me3	LD
chr12	11163882	11164386	DEL	chr12_11164504_11165342	2.87236588	11.390562	3.1861E-06	0.0356046	H3K27me3	LD
chr12	11176768	11176815	DEL	chr12_11164504_11165342	2.87236588	11.390562	3.1861E-06	0.0356046	H3K27me3	LD
chr5	88002684	88002685	INS	chr5_88121966_88122358	1.68617672	11.3899795	3.1874E-06	0.0356046	H3K27me3	LD
chr12	11241417	11241418	INS	chr12_11164504_11165342	2.87236588	11.390562	3.1861E-06	0.0356046	H3K27me3	LD
chr1	271070524	271070525	INS	chr1_271039298_271039708	2.28815833	11.422202	3.1198E-06	0.0356046	H3K27me3	LD
chr2	86013658	86014869	DEL	chr2_86156542_86157033	2.72129063	11.3513553	3.2705E-06	0.03594673	H3K27me3	LD
chr9	5924259	5925901	DEL	chr9_6018977_6019646	6.06189944	11.3576608	3.2568E-06	0.03594673	H3K27me3	LD
chr17	47374149	47374256	DEL	chr17_47179106_47179470	2.32303472	11.2403972	3.5231E-06	0.03836979	H3K27me3	LD
chr17	47281163	47281164	INS	chr17_47179106_47179470	2.32303472	11.2403972	3.5231E-06	0.03836979	H3K27me3	LD
chr8	12757116	12757117	INS	chr8_12686840_12687121	2.0095534	11.1501365	3.7448E-06	0.04059835	H3K27me3	LD
chr15	17393203	17393204	INS	chr15_17403140_17403479	1.43721206	11.1377718	3.7763E-06	0.04075525	H3K27me3	LD

chr5	5848912	5848913	INS	chr5_5842790_5843143	1.977255	11.0532332	4.0002E-06	0.0427846	H3K27me3	LD
chr5	5949172	5949173	INS	chr5_5842790_5843143	1.977255	11.0532332	4.0002E-06	0.0427846	H3K27me3	LD
chr12	6156017	6156192	DEL	chr12_6265602_6265902	2.94028174	11.030382	4.0633E-06	0.04324567	H3K27me3	LD
chr1	255589665	255589952	DEL	chr1_255684931_255685243	2.19467556	11.0180599	4.0978E-06	0.04324567	H3K27me3	LD
chr1	255667140	255667141	INS	chr1_255684931_255685243	2.19467556	11.0180599	4.0978E-06	0.04324567	H3K27me3	LD
chr14	85270599	85270600	INS	chr14_85270003_85270406	3.05460977	10.9800304	4.2062E-06	0.04419433	H3K27me3	LD
chr2	117287618	117288492	DEL	chr2_117311555_117311854	2.29527439	10.9460685	4.3057E-06	0.04445681	H3K27me3	LD
chr2	117318933	117319252	DEL	chr2_117311555_117311854	2.29527439	10.9460685	4.3057E-06	0.04445681	H3K27me3	LD
chr2	117134521	117134522	INS	chr2_117311555_117311854	2.29527439	10.9460685	4.3057E-06	0.04445681	H3K27me3	LD
chr2	117310807	117310808	INS	chr2_117311555_117311854	2.29527439	10.9460685	4.3057E-06	0.04445681	H3K27me3	LD
chr1	146611148	146611149	INS	chr1_146490851_146491256	1.62909306	10.910387	4.4131E-06	0.04517485	H3K27me3	LD
chr1	146634883	146634884	INS	chr1_146490851_146491256	1.62909306	10.910387	4.4131E-06	0.04517485	H3K27me3	LD
chr10	15126572	15126630	DEL	chr10_15027297_15027548	2.07462965	10.8498555	4.6023E-06	0.04631604	H3K27me3	LD

chr7	81123949	81123950	INS	chr7_81033061_81033353	1.19278243	10.8558685	4.5831E-06	0.04631604	H3K27me3	LD
chr10	15223813	15223814	INS	chr10_15027297_15027548	2.07462965	10.8498555	4.6023E-06	0.04631604	H3K27me3	LD
chr6	164202716	164202875	DEL	chr6_164372022_164372409	2.43735708	10.8129663	4.722E-06	0.04692678	H3K27me3	LD
chr6	164209308	164209595	DEL	chr6_164372022_164372409	2.43735708	10.8129663	4.722E-06	0.04692678	H3K27me3	LD
chr17	50293185	50293288	DEL	chr17_50209815_50210865	-5.7083438	-10.794481	4.7833E-06	0.04714307	H3K27me3	LD
chr17	50296865	50296866	INS	chr17_50209815_50210865	-5.7083438	-10.794481	4.7833E-06	0.04714307	H3K27me3	LD
chr6	128624985	128624986	INS	chr6_128579101_128580055	1.58616894	10.7807367	4.8295E-06	0.04740203	H3K27me3	LD
chr13	81616400	81616672	DEL	chr13_81687199_81687972	-1.7506675	-10.70627	5.0883E-06	0.04783809	H3K27me3	LD
chr13	81624645	81624975	DEL	chr13_81687199_81687972	-1.7506675	-10.70627	5.0883E-06	0.04783809	H3K27me3	LD
chr13	81703158	81703229	DEL	chr13_81687199_81687972	-1.7506675	-10.70627	5.0883E-06	0.04783809	H3K27me3	LD
chr8	29017260	29017557	DEL	chr8_29071776_29072298	1.15787931	10.7170629	5.0498E-06	0.04783809	H3K27me3	LD
chr12	39468817	39477240	DEL	chr12_39465446_39465763	2.2669475	10.7357041	4.9842E-06	0.04783809	H3K27me3	LD
chr12	39626204	39626506	DEL	chr12_39465446_39465763	2.2669475	10.7357041	4.9842E-06	0.04783809	H3K27me3	LD

chr12	39651505	39651565	DEL	chr12_39465446_39465763	2.2669475	10.7357041	4.9842E-06	0.04783809	H3K27me3	LD
chr13	47053331	47053332	INS	chr13_47137251_47139592	-1.463282	-10.733886	4.9905E-06	0.04783809	H3K27me3	LD
chr13	81620142	81620143	INS	chr13_81687199_81687972	-1.7506675	-10.70627	5.0883E-06	0.04783809	H3K27me3	LD
chr13	81643667	81643668	INS	chr13_81687199_81687972	-1.7506675	-10.70627	5.0883E-06	0.04783809	H3K27me3	LD
chr4	17723413	17723414	INS	chr4_17724392_17724770	1.66924531	10.6933937	5.1346E-06	0.04783809	H3K27me3	LD
chr12	60251195	60251196	INS	chr12_60288162_60288702	4.07984444	10.637311	5.3419E-06	0.04957567	H3K27me3	LD
chr10	4781600	4781601	INS	chr10_4588090_4588811	2.01616771	32.2841317	9.2331E-10	0.00045753	H3K4me1	LD
chr6	27274566	27274567	INS	chr6_27207938_27208927	3.953636	31.2409114	1.1986E-09	0.00050908	H3K4me1	LD
chr17	49961229	49962027	DEL	chr17_50074512_50075131	1.57075688	28.7961986	2.2884E-09	0.00075597	H3K4me1	LD
chr1	8139905	8139906	INS	chr1_8056736_8057121	5.78574733	26.0652929	5.0398E-09	0.00149842	H3K4me1	LD
chr16	67210384	67211872	DEL	chr16_67166852_67167336	1.25004161	21.546311	2.2673E-08	0.00306446	H3K4me1	LD
chr12	55616959	55617051	DEL	chr12_55493879_55495119	3.04392446	21.4878489	2.3164E-08	0.00306446	H3K4me1	LD
chr11	72462361	72462460	DEL	chr11_72591914_72593566	2.11559433	20.2653965	3.6729E-08	0.00306446	H3K4me1	LD

chr3	11378193	11378455	DEL	chr3_11376505_11378056	3.42559198	22.0310653	1.9027E-08	0.00306446	H3K4me1	LD
chr1	16101216	16101427	DEL	chr1_16052953_16054413	2.16467333	20.239145	3.7105E-08	0.00306446	H3K4me1	LD
chr3	33375700	33377288	DEL	chr3_33326900_33328307	1.51028878	21.5438522	2.2694E-08	0.00306446	H3K4me1	LD
chr3	33374425	33375258	DEL	chr3_33326900_33328307	1.51028878	21.5438522	2.2694E-08	0.00306446	H3K4me1	LD
chr3	33378973	33379109	DEL	chr3_33326900_33328307	1.51028878	21.5438522	2.2694E-08	0.00306446	H3K4me1	LD
chr16	47272116	47272167	DEL	chr16_47292388_47293170	2.79418438	20.8702041	2.9144E-08	0.00306446	H3K4me1	LD
chr16	47282373	47282731	DEL	chr16_47292388_47293170	2.79418438	20.8702041	2.9144E-08	0.00306446	H3K4me1	LD
chr12	55609745	55609746	INS	chr12_55493879_55495119	3.04392446	21.4878489	2.3164E-08	0.00306446	H3K4me1	LD
chr16	69079940	69079941	INS	chr16_69078602_69080730	3.00776134	20.4588315	3.4085E-08	0.00306446	H3K4me1	LD
chr16	69208366	69208367	INS	chr16_69078602_69080730	3.00776134	20.4588315	3.4085E-08	0.00306446	H3K4me1	LD
chr3	58398188	58398189	INS	chr3_58469995_58471918	6.91112063	20.391972	3.4974E-08	0.00306446	H3K4me1	LD
chr3	58568874	58568875	INS	chr3_58469995_58471918	6.91112063	20.391972	3.4974E-08	0.00306446	H3K4me1	LD
chr6	94726778	94726779	INS	chr6_94665987_94666918	1.57129719	21.4473806	2.3511E-08	0.00306446	H3K4me1	LD

chr6	94760548	94760549	INS	chr6_94665987_94666918	1.57129719	21.4473806	2.3511E-08	0.00306446	H3K4me1	LD
chr6	94769331	94769332	INS	chr6_94665987_94666918	1.57129719	21.4473806	2.3511E-08	0.00306446	H3K4me1	LD
chr5	1341172	1341173	INS	chr5_1346805_1347750	1.90299631	20.6279637	3.1948E-08	0.00306446	H3K4me1	LD
chr9	11987399	11987400	INS	chr9_12099590_12100622	1.5892985	20.5143426	3.3366E-08	0.00306446	H3K4me1	LD
chr9	12086715	12086716	INS	chr9_12099590_12100622	1.5892985	20.5143426	3.3366E-08	0.00306446	H3K4me1	LD
chr9	12106144	12106145	INS	chr9_12099590_12100622	1.5892985	20.5143426	3.3366E-08	0.00306446	H3K4me1	LD
chr9	12260665	12260666	INS	chr9_12099590_12100622	1.5892985	20.5143426	3.3366E-08	0.00306446	H3K4me1	LD
chr1	15993713	15993714	INS	chr1_16052953_16054413	2.16467333	20.239145	3.7105E-08	0.00306446	H3K4me1	LD
chr1	16056082	16056083	INS	chr1_16052953_16054413	2.16467333	20.239145	3.7105E-08	0.00306446	H3K4me1	LD
chr15	123686711	123686857	DEL	chr15_123756041_123756261	1.94278944	20.1093113	3.9031E-08	0.0030871	H3K4me1	LD
chr11	357926	358211	DEL	chr11_364308_366545	1.01561813	20.0816027	3.9456E-08	0.0030871	H3K4me1	LD
chr15	55790209	55790210	INS	chr15_55790577_55791230	1.20435042	19.6459806	4.6876E-08	0.00357358	H3K4me1	LD
chr1	19718812	19718990	DEL	chr1_19689998_19691069	2.26059138	19.4534799	5.0644E-08	0.00358508	H3K4me1	LD

chr1	19660178	19660179	INS	chr1_19689998_19691069	2.26059138	19.4534799	5.0644E-08	0.00358508	H3K4me1	LD
chr1	19603931	19603932	INS	chr1_19689998_19691069	2.26059138	19.4534799	5.0644E-08	0.00358508	H3K4me1	LD
chr13	200736890	200736891	INS	chr13_200873245_200874136	1.63743825	19.3129242	5.361E-08	0.00362257	H3K4me1	LD
chr13	201014236	201014237	INS	chr13_200873245_200874136	1.63743825	19.3129242	5.361E-08	0.00362257	H3K4me1	LD
chr9	8342843	8342844	INS	chr9_8363263_8363974	3.05257467	18.8293926	6.5409E-08	0.00413769	H3K4me1	LD
chr9	8492517	8492518	INS	chr9_8363263_8363974	3.05257467	18.8293926	6.5409E-08	0.00413769	H3K4me1	LD
chr8	20174411	20175309	DEL	chr8_20167704_20169850	3.85535142	18.7597067	6.7338E-08	0.00417099	H3K4me1	LD
chr7	19255752	19255941	DEL	chr7_19299010_19299952	1.1871655	18.4041083	7.8234E-08	0.00456084	H3K4me1	LD
chr7	19165586	19165587	INS	chr7_19299010_19299952	1.1871655	18.4041083	7.8234E-08	0.00456084	H3K4me1	LD
chr7	19481961	19481962	INS	chr7_19299010_19299952	1.1871655	18.4041083	7.8234E-08	0.00456084	H3K4me1	LD
chr3	1265693	1265743	DEL	chr3_1422015_1422908	2.90207278	17.9448492	9.5351E-08	0.00534896	H3K4me1	LD
chr3	1401799	1401856	DEL	chr3_1422015_1422908	2.90207278	17.9448492	9.5351E-08	0.00534896	H3K4me1	LD
chr17	50472659	50472804	DEL	chr17_50659235_50659858	-1.2952085	-17.776501	1.0265E-07	0.00535423	H3K4me1	LD

chr17	50562308	50562386	DEL	chr17_50659235_50659858	-1.2952085	-17.776501	1.0265E-07	0.00535423	H3K4me1	LD
chr17	50560674	50560985	DEL	chr17_50659235_50659858	-1.2952085	-17.776501	1.0265E-07	0.00535423	H3K4me1	LD
chr17	50659510	50659511	INS	chr17_50659235_50659858	-1.2952085	-17.776501	1.0265E-07	0.00535423	H3K4me1	LD
chr14	109703811	109704122	DEL	chr14_109737889_109738957	1.49647367	17.6094624	1.1051E-07	0.00544005	H3K4me1	LD
chr15	123686711	123686857	DEL	chr15_123754472_123755054	2.6881615	17.6535119	1.0838E-07	0.00544005	H3K4me1	LD
chr14	109603030	109603031	INS	chr14_109737889_109738957	1.49647367	17.6094624	1.1051E-07	0.00544005	H3K4me1	LD
chr13	121330881	121330882	INS	chr13_121328978_121330882	-0.9216142	-17.55066	1.1344E-07	0.00544005	H3K4me1	LD
chr13	121380583	121380584	INS	chr13_121328978_121330882	-0.9216142	-17.55066	1.1344E-07	0.00544005	H3K4me1	LD
chr6	19872529	19872530	INS	chr6_19788920_19789534	2.46533872	17.4511472	1.186E-07	0.00550957	H3K4me1	LD
chr6	19947161	19947162	INS	chr6_19788920_19789534	2.46533872	17.4511472	1.186E-07	0.00550957	H3K4me1	LD
chr3	71011959	71012025	DEL	chr3_71062705_71063640	1.84245892	17.3307614	1.2519E-07	0.00559758	H3K4me1	LD
chr7	67736522	67736838	DEL	chr7_67700889_67701770	2.89737794	17.281743	1.2799E-07	0.00559758	H3K4me1	LD
chr8	31901932	31901933	INS	chr8_32068784_32070081	1.8144743	17.2811893	1.2802E-07	0.00559758	H3K4me1	LD

chr8	32069597	32069598	INS	chr8_32068784_32070081	-1.8144743	-17.281189	1.2802E-07	0.00559758	H3K4me1	LD
chr11	24527694	24527745	DEL	chr11_24608301_24609019	1.76114876	16.9768433	1.4708E-07	0.00607344	H3K4me1	LD
chr11	24790094	24790150	DEL	chr11_24608301_24609019	1.76114876	16.9768433	1.4708E-07	0.00607344	H3K4me1	LD
chr11	24683681	24683682	INS	chr11_24608301_24609019	1.76114876	16.9768433	1.4708E-07	0.00607344	H3K4me1	LD
chr7	78409570	78409571	INS	chr7_78538513_78540058	1.60974775	16.6052339	1.748E-07	0.00711916	H3K4me1	LD
chr1	8139905	8139906	INS	chr1_8059193_8060146	4.56679389	16.4559161	1.8755E-07	0.00753529	H3K4me1	LD
chr7	39583205	39583484	DEL	chr7_39561765_39562107	1.79073239	16.3035783	2.0164E-07	0.00778449	H3K4me1	LD
chr7	39605565	39606394	DEL	chr7_39561765_39562107	1.79073239	16.3035783	2.0164E-07	0.00778449	H3K4me1	LD
chr3	73117863	73118163	DEL	chr3_73127753_73129831	-2.0752455	-16.147631	2.1731E-07	0.00778449	H3K4me1	LD
chr10	63563084	63563693	DEL	chr10_63527279_63527944	0.95152511	16.1713117	2.1485E-07	0.00778449	H3K4me1	LD
chr10	63563897	63565513	DEL	chr10_63527279_63527944	0.95152511	16.1713117	2.1485E-07	0.00778449	H3K4me1	LD
chr10	63565396	63565595	DEL	chr10_63527279_63527944	1.90305022	16.1713117	2.1485E-07	0.00778449	H3K4me1	LD
chr7	39554312	39554313	INS	chr7_39561765_39562107	1.79073239	16.3035783	2.0164E-07	0.00778449	H3K4me1	LD

chr10	63649261	63649262	INS	chr10_63527279_63527944	0.95152511	16.1713117	2.1485E-07	0.00778449	H3K4me1	LD
chr10	63713642	63713643	INS	chr10_63527279_63527944	0.95152511	16.1713117	2.1485E-07	0.00778449	H3K4me1	LD
chr4	68534750	68534973	DEL	chr4_68543932_68544543	2.87313583	16.0164415	2.3156E-07	0.00809964	H3K4me1	LD
chr4	68536364	68536365	INS	chr4_68543932_68544543	2.87313583	16.0164415	2.3156E-07	0.00809964	H3K4me1	LD
chr9	112245184	112245340	DEL	chr9_112246131_112246765	1.00445305	15.9912324	2.3442E-07	0.00810419	H3K4me1	LD
chr10	45589385	45589632	DEL	chr10_45391663_45392249	1.45068467	15.8207683	2.548E-07	0.00823432	H3K4me1	LD
chr8	31966170	31966514	DEL	chr8_31992415_31992754	1.58572875	15.8434218	2.5198E-07	0.00823432	H3K4me1	LD
chr8	31976589	31976918	DEL	chr8_31992415_31992754	1.58572875	15.8434218	2.5198E-07	0.00823432	H3K4me1	LD
chr10	45496181	45496182	INS	chr10_45391663_45392249	1.45068467	15.8207683	2.548E-07	0.00823432	H3K4me1	LD
chr8	31807892	31807893	INS	chr8_31992415_31992754	1.58572875	15.8434218	2.5198E-07	0.00823432	H3K4me1	LD
chr8	31905621	31905622	INS	chr8_31992415_31992754	1.58572875	15.8434218	2.5198E-07	0.00823432	H3K4me1	LD
chr15	25320801	25321010	DEL	chr15_25447757_25450423	2.68531167	15.7458858	2.6437E-07	0.00827397	H3K4me1	LD
chr16	52083168	52083464	DEL	chr16_52197794_52199096	1.492586	15.7627504	2.6218E-07	0.00827397	H3K4me1	LD

chr15	25284197	25284198	INS	chr15_25447757_25450423	2.68531167	15.7458858	2.6437E-07	0.00827397	H3K4me1	LD
chr13	196074562	196074853	DEL	chr13_196220876_196221219	2.69561563	15.671038	2.7435E-07	0.00840915	H3K4me1	LD
chr13	196377926	196377927	INS	chr13_196220876_196221219	2.69561563	15.671038	2.7435E-07	0.00840915	H3K4me1	LD
chr2	38662702	38663906	DEL	chr2_38816095_38816441	1.49123928	15.551754	2.9113E-07	0.00874331	H3K4me1	LD
chr2	38702334	38702406	DEL	chr2_38816095_38816441	1.49123928	15.551754	2.9113E-07	0.00874331	H3K4me1	LD
chr7	58583768	58584076	DEL	chr7_58711124_58713732	3.1706287	15.4428007	3.0747E-07	0.00905543	H3K4me1	LD
chr12	13104722	13104723	INS	chr12_13191830_13193116	2.69245317	15.4418767	3.0762E-07	0.00905543	H3K4me1	LD
chr7	19255752	19255941	DEL	chr7_19300713_19301090	1.93418629	15.3565915	3.2113E-07	0.00918065	H3K4me1	LD
chr7	19165586	19165587	INS	chr7_19300713_19301090	1.93418629	15.3565915	3.2113E-07	0.00918065	H3K4me1	LD
chr7	19481961	19481962	INS	chr7_19300713_19301090	1.93418629	15.3565915	3.2113E-07	0.00918065	H3K4me1	LD
chr3	92200089	92200090	INS	chr3_92274459_92274879	1.834023	15.2648512	3.3642E-07	0.00943627	H3K4me1	LD
chr15	72137567	72137568	INS	chr15_72165525_72166032	2.45272831	15.0685058	3.7197E-07	0.01033571	H3K4me1	LD
chr9	134460817	134460818	INS	chr9_134457217_134458604	2.02122719	14.918024	4.0206E-07	0.01106859	H3K4me1	LD

chr12	55102291	55102670	DEL	chr12_55109709_55110900	1.06994208	14.7389302	4.415E-07	0.01112423	H3K4me1	LD
chr12	55111149	55111479	DEL	chr12_55109709_55110900	1.06994208	14.7389302	4.415E-07	0.01112423	H3K4me1	LD
chr12	55139758	55139759	INS	chr12_55109709_55110900	1.06994208	14.7389302	4.415E-07	0.01112423	H3K4me1	LD
chr12	55199996	55199997	INS	chr12_55109709_55110900	1.06994208	14.7389302	4.415E-07	0.01112423	H3K4me1	LD
chr8	65352151	65352152	INS	chr8_65397960_65398364	1.42255025	14.8337296	4.2011E-07	0.01112423	H3K4me1	LD
chr8	65362018	65362019	INS	chr8_65397960_65398364	1.42255025	14.8337296	4.2011E-07	0.01112423	H3K4me1	LD
chr8	65431085	65431086	INS	chr8_65397960_65398364	1.42255025	14.8337296	4.2011E-07	0.01112423	H3K4me1	LD
chr2	76434615	76434616	INS	chr2_76564501_76566045	-1.2362938	-14.773462	4.3357E-07	0.01112423	H3K4me1	LD
chr2	76431520	76431521	INS	chr2_76564501_76566045	-1.2362938	-14.773462	4.3357E-07	0.01112423	H3K4me1	LD
chr11	71543065	71543065	BND	chr11_71488868_71490435	1.50438183	14.4351726	5.1871E-07	0.01118227	H3K4me1	LD
chr7	65648286	65648589	DEL	chr7_65647311_65647956	2.2561645	14.533417	4.9219E-07	0.01118227	H3K4me1	LD
chr13	195524615	195525560	DEL	chr13_195613900_195614372	1.37960881	14.3939388	5.3031E-07	0.01118227	H3K4me1	LD
chr10	34107229	34107230	INS	chr10_33959473_33960785	2.74128361	14.5081599	4.9886E-07	0.01118227	H3K4me1	LD

chr16	71784615	71784616	INS	chr16_71746874_71747350	4.74988481	14.4727077	5.0839E-07	0.01118227	H3K4me1	LD
chr11	71467065	71467066	INS	chr11_71488868_71490435	1.50438183	14.4351726	5.1871E-07	0.01118227	H3K4me1	LD
chr11	71543074	71543075	INS	chr11_71488868_71490435	1.50438183	14.4351726	5.1871E-07	0.01118227	H3K4me1	LD
chr5	91957112	91957113	INS	chr5_92117085_92118151	1.68160254	14.4880033	5.0426E-07	0.01118227	H3K4me1	LD
chr7	102537386	102537387	INS	chr7_102559980_102561602	2.41897892	14.6007196	4.7491E-07	0.01118227	H3K4me1	LD
chr13	195425233	195425234	INS	chr13_195613900_195614372	1.37960881	14.3939388	5.3031E-07	0.01118227	H3K4me1	LD
chr13	195492082	195492083	INS	chr13_195613900_195614372	1.37960881	14.3939388	5.3031E-07	0.01118227	H3K4me1	LD
chr13	195532827	195532828	INS	chr13_195613900_195614372	1.37960881	14.3939388	5.3031E-07	0.01118227	H3K4me1	LD
chr13	195687925	195687926	INS	chr13_195613900_195614372	1.37960881	14.3939388	5.3031E-07	0.01118227	H3K4me1	LD
chr4	38216363	38216364	INS	chr4_38209531_38211112	-0.8220374	-14.566498	4.8361E-07	0.01118227	H3K4me1	LD
chr13	31599528	31599529	INS	chr13_31599037_31600633	-1.2906016	-14.315332	5.5324E-07	0.01150267	H3K4me1	LD
chr15	128656264	128656265	INS	chr15_128630955_128631403	0.85996952	14.315582	5.5317E-07	0.01150267	H3K4me1	LD
chr5	91273146	91273676	DEL	chr5_91274261_91275687	1.90370826	14.2672151	5.6783E-07	0.01172392	H3K4me1	LD

chr7	77729016	77729017	INS	chr7_77652412_77653126	1.87741083	14.1964448	5.9007E-07	0.01193448	H3K4me1	LD
chr7	77697182	77697183	INS	chr7_77652412_77653126	1.87741083	14.1964448	5.9007E-07	0.01193448	H3K4me1	LD
chr2	4682489	4682545	DEL	chr2_4544881_4546444	4.21898456	14.0354606	6.4439E-07	0.01206204	H3K4me1	LD
chr2	4696179	4696361	DEL	chr2_4544881_4546444	4.21898456	14.0354606	6.4439E-07	0.01206204	H3K4me1	LD
chr5	70720777	70721047	DEL	chr5_70760432_70761012	2.01886972	14.0507067	6.3901E-07	0.01206204	H3K4me1	LD
chr14	125749696	125752831	DEL	chr14_125766127_125766770	1.3278015	14.0754476	6.3039E-07	0.01206204	H3K4me1	LD
chr14	125820966	125821859	DEL	chr14_125766127_125766770	1.3278015	14.0754476	6.3039E-07	0.01206204	H3K4me1	LD
chr8	121420487	121421126	DEL	chr8_121279783_121281324	-1.3914361	-13.91244	6.8968E-07	0.01206204	H3K4me1	LD
chr8	121424274	121424553	DEL	chr8_121279783_121281324	-1.3914361	-13.91244	6.8968E-07	0.01206204	H3K4me1	LD
chr14	133145647	133145877	DEL	chr14_133212379_133213439	1.98439378	13.9209057	6.8645E-07	0.01206204	H3K4me1	LD
chr14	133203448	133203517	DEL	chr14_133212379_133213439	3.96878756	13.9209057	6.8645E-07	0.01206204	H3K4me1	LD
chr14	133232275	133232392	DEL	chr14_133212379_133213439	1.98439378	13.9209057	6.8645E-07	0.01206204	H3K4me1	LD
chr14	133315198	133315368	DEL	chr14_133212379_133213439	1.98439378	13.9209057	6.8645E-07	0.01206204	H3K4me1	LD

chr2	4411235	4411236	INS	chr2_4544881_4546444	4.21898456	14.0354606	6.4439E-07	0.01206204	H3K4me1	LD
chr2	4544282	4544283	INS	chr2_4544881_4546444	4.21898456	14.0354606	6.4439E-07	0.01206204	H3K4me1	LD
chr2	4581207	4581208	INS	chr2_4544881_4546444	4.21898456	14.0354606	6.4439E-07	0.01206204	H3K4me1	LD
chr4	80062482	80062483	INS	chr4_79985334_79986402	1.2318529	13.9429581	6.7812E-07	0.01206204	H3K4me1	LD
chr8	121224053	121224054	INS	chr8_121279783_121281324	-1.3914361	-13.91244	6.8968E-07	0.01206204	H3K4me1	LD
chr14	125942900	125942901	INS	chr14_125766127_125766770	1.3278015	14.0754476	6.3039E-07	0.01206204	H3K4me1	LD
chr14	125949805	125949806	INS	chr14_125766127_125766770	1.3278015	14.0754476	6.3039E-07	0.01206204	H3K4me1	LD
chr8	121422264	121422265	INS	chr8_121279783_121281324	-1.3914361	-13.91244	6.8968E-07	0.01206204	H3K4me1	LD
chr14	133104665	133104666	INS	chr14_133212379_133213439	1.98439378	13.9209057	6.8645E-07	0.01206204	H3K4me1	LD
chr14	133202898	133202899	INS	chr14_133212379_133213439	1.98439378	13.9209057	6.8645E-07	0.01206204	H3K4me1	LD
chr14	133285066	133285067	INS	chr14_133212379_133213439	1.98439378	13.9209057	6.8645E-07	0.01206204	H3K4me1	LD
chr3	11378193	11378455	DEL	chr3_11378537_11379431	3.59072764	13.8608546	7.0973E-07	0.01229356	H3K4me1	LD
chr8	1451974	1451975	INS	chr8_1396325_1396683	2.66404992	13.8571587	7.1119E-07	0.01229356	H3K4me1	LD

chr9	119784323	119784390	DEL	chr9_119707403_119709150	1.61315125	13.7932766	7.3699E-07	0.01259303	H3K4me1	LD
chr2	103924277	103924278	INS	chr2_103770411_103771451	2.23215356	13.7061422	7.7388E-07	0.01314783	H3K4me1	LD
chr14	78560469	78560807	DEL	chr14_78582820_78583769	1.62389589	13.6571076	7.9554E-07	0.01343915	H3K4me1	LD
chr13	28833746	28833747	INS	chr13_28964597_28965052	3.64355472	13.6331819	8.0636E-07	0.01346884	H3K4me1	LD
chr13	29034487	29034488	INS	chr13_28964597_28965052	3.64355472	13.6331819	8.0636E-07	0.01346884	H3K4me1	LD
chr11	12454581	12454582	INS	chr11_12650150_12651903	1.60080708	13.593891	8.2449E-07	0.01361858	H3K4me1	LD
chr11	12803087	12803088	INS	chr11_12650150_12651903	1.60080708	13.593891	8.2449E-07	0.01361858	H3K4me1	LD
chr5	15365771	15366055	DEL	chr5_15374871_15376315	1.36265098	13.5339594	8.5302E-07	0.01393503	H3K4me1	LD
chr5	15273778	15273779	INS	chr5_15374871_15376315	1.36265098	13.5339594	8.5302E-07	0.01393503	H3K4me1	LD
chr2	3928648	3928814	DEL	chr2_3822084_3822396	1.54275903	13.3383386	9.5414E-07	0.01430946	H3K4me1	LD
chr2	3940139	3940433	DEL	chr2_3822084_3822396	1.54275903	13.3383386	9.5414E-07	0.01430946	H3K4me1	LD
chr7	64386545	64386814	DEL	chr7_64530515_64530934	-2.2245995	-13.360356	9.4211E-07	0.01430946	H3K4me1	LD
chr7	64388635	64388951	DEL	chr7_64530515_64530934	-2.2245995	-13.360356	9.4211E-07	0.01430946	H3K4me1	LD

chr7	64531142	64531452	DEL	chr7_64530515_64530934	-2.2245995	-13.360356	9.4211E-07	0.01430946	H3K4me1	LD
chr7	64675851	64676009	DEL	chr7_64530515_64530934	2.2245995	13.3603565	9.4211E-07	0.01430946	H3K4me1	LD
chr15	106150004	106150302	DEL	chr15_106069940_106071783	1.32212881	13.3762026	9.3356E-07	0.01430946	H3K4me1	LD
chr1	16101216	16101427	DEL	chr1_16059297_16060139	1.93288172	13.3382771	9.5417E-07	0.01430946	H3K4me1	LD
chr11	24007198	24007277	DEL	chr11_23984132_23986598	2.01904431	13.4004191	9.2066E-07	0.01430946	H3K4me1	LD
chr7	64693046	64693047	INS	chr7_64530515_64530934	2.2245995	13.3603565	9.4211E-07	0.01430946	H3K4me1	LD
chr7	64676529	64676530	INS	chr7_64530515_64530934	2.2245995	13.3603565	9.4211E-07	0.01430946	H3K4me1	LD
chr15	106084958	106084959	INS	chr15_106069940_106071783	1.32212881	13.3762026	9.3356E-07	0.01430946	H3K4me1	LD
chr1	15993713	15993714	INS	chr1_16059297_16060139	1.93288172	13.3382771	9.5417E-07	0.01430946	H3K4me1	LD
chr1	16056082	16056083	INS	chr1_16059297_16060139	1.93288172	13.3382771	9.5417E-07	0.01430946	H3K4me1	LD
chr9	26785601	26785602	INS	chr9_26685227_26686128	1.13218524	13.4398368	9.0009E-07	0.01430946	H3K4me1	LD
chr9	26794968	26794969	INS	chr9_26685227_26686128	1.13218524	13.4398368	9.0009E-07	0.01430946	H3K4me1	LD
chr6	2421642	2421643	INS	chr6_2271631_2273152	0.99628742	13.3317693	9.5776E-07	0.01430946	H3K4me1	LD

chr10	41706454	41706455	INS	chr10_41895352_41896643	1.45706722	13.3206899	9.639E-07	0.01432922	H3K4me1	LD
chr2	148849256	148849257	INS	chr2_148704845_148705782	1.89333094	13.2946838	9.7849E-07	0.01447378	H3K4me1	LD
chr1	7022841	7022842	INS	chr1_6919198_6919738	2.42759283	13.2364635	1.0121E-06	0.01467828	H3K4me1	LD
chr1	7023848	7023849	INS	chr1_6919198_6919738	2.42759283	13.2364635	1.0121E-06	0.01467828	H3K4me1	LD
chr4	11602056	11602225	DEL	chr4_11775624_11777297	1.42138242	13.1947102	1.0369E-06	0.01486814	H3K4me1	LD
NW_018084968.1	771072	771073	INS	NW_018084968.1_639544_640815	1.81663339	13.1811543	1.0452E-06	0.01486814	H3K4me1	LD
NW_018084968.1	752152	752153	INS	NW_018084968.1_639544_640815	1.81663339	13.1811543	1.0452E-06	0.01486814	H3K4me1	LD
NW_018084968.1	584050	584051	INS	NW_018084968.1_639544_640815	1.81663339	13.1811543	1.0452E-06	0.01486814	H3K4me1	LD
chr3	69706401	69706402	INS	chr3_69832712_69834821	2.06144238	13.1557999	1.0607E-06	0.01494666	H3K4me1	LD
chr5	10936266	10936267	INS	chr5_11007763_11008618	1.18126722	13.1416463	1.0695E-06	0.01499967	H3K4me1	LD
chr6	146564199	146564199	BND	chr6_146410162_146412225	4.11403611	13.1052373	1.0926E-06	0.01502977	H3K4me1	LD
chr6	137195264	137195347	DEL	chr6_137168398_137169625	1.11863733	13.0672761	1.1172E-06	0.01502977	H3K4me1	LD
chr6	137253415	137256323	DEL	chr6_137168398_137169625	1.11863733	13.0672761	1.1172E-06	0.01502977	H3K4me1	LD

chr6	137250829	137250945	DEL	chr6_137168398_137169625	1.11863733	13.0672761	1.1172E-06	0.01502977	H3K4me1	LD
chr5	65609792	65609793	INS	chr5_65670545_65670935	1.26877894	13.1034745	1.0937E-06	0.01502977	H3K4me1	LD
chr6	137252280	137252281	INS	chr6_137168398_137169625	1.11863733	13.0672761	1.1172E-06	0.01502977	H3K4me1	LD
chr6	137266730	137266731	INS	chr6_137168398_137169625	1.11863733	13.0672761	1.1172E-06	0.01502977	H3K4me1	LD
chr6	146268523	146268524	INS	chr6_146410162_146412225	2.05701806	13.1052373	1.0926E-06	0.01502977	H3K4me1	LD
chr6	146564203	146564204	INS	chr6_146410162_146412225	2.05701806	13.1052373	1.0926E-06	0.01502977	H3K4me1	LD
chr5	19305696	19305895	DEL	chr5_19407888_19408655	3.78230056	13.006064	1.1582E-06	0.01525722	H3K4me1	LD
chr7	116344914	116344915	INS	chr7_116444712_116446266	1.733198	13.0037758	1.1597E-06	0.01525722	H3K4me1	LD
chr7	116359034	116359035	INS	chr7_116444712_116446266	1.733198	13.0037758	1.1597E-06	0.01525722	H3K4me1	LD
chr5	19551761	19551762	INS	chr5_19407888_19408655	7.56460111	13.006064	1.1582E-06	0.01525722	H3K4me1	LD
chr5	50760528	50760593	DEL	chr5_50580132_50581189	1.11816056	12.9940754	1.1664E-06	0.01526477	H3K4me1	LD
chr15	30642959	30643060	DEL	chr15_30795511_30796816	1.7238875	12.9732441	1.1809E-06	0.01526477	H3K4me1	LD
chr3	96447744	96447745	INS	chr3_96340739_96341058	1.10918363	12.9796937	1.1764E-06	0.01526477	H3K4me1	LD

chr13	195844665	195844783	DEL	chr13_195998648_196000441	3.37840293	12.9129188	1.2238E-06	0.01568406	H3K4me1	LD
chr6	133358116	133358117	INS	chr6_133508012_133508653	1.41789378	12.91854	1.2198E-06	0.01568406	H3K4me1	LD
chr15	121393670	121393832	DEL	chr15_121334750_121335031	3.12969567	12.8779067	1.2496E-06	0.01574265	H3K4me1	LD
chr14	126234262	126234449	DEL	chr14_126197819_126198922	1.74386378	12.8802119	1.2479E-06	0.01574265	H3K4me1	LD
chr15	121344691	121344692	INS	chr15_121334750_121335031	3.12969567	12.8779067	1.2496E-06	0.01574265	H3K4me1	LD
chr7	102537386	102537387	INS	chr7_102552433_102553176	2.0879815	12.879986	1.248E-06	0.01574265	H3K4me1	LD
chr5	72889345	72889346	INS	chr5_72845104_72845529	1.47777567	12.8307288	1.2853E-06	0.01612362	H3K4me1	LD
chr3	117629846	117630456	DEL	chr3_117758238_117760471	-2.7303254	-12.750253	1.3488E-06	0.01684908	H3K4me1	LD
chr5	4038027	4038112	DEL	chr5_4156213_4157192	4.48452556	12.6251076	1.4546E-06	0.01702659	H3K4me1	LD
chr5	4058472	4058562	DEL	chr5_4156213_4157192	4.48452556	12.6251076	1.4546E-06	0.01702659	H3K4me1	LD
chr9	126502338	126503142	DEL	chr9_126499353_126499695	1.29206391	12.6347007	1.4462E-06	0.01702659	H3K4me1	LD
chr3	18305990	18306041	DEL	chr3_18113989_18114331	1.81148931	12.6847546	1.403E-06	0.01702659	H3K4me1	LD
chr7	46835451	46841912	DUP	chr7_46838400_46839725	3.29836125	12.6304788	1.4499E-06	0.01702659	H3K4me1	LD

chr7	46900509	46900510	INS	chr7_46838400_46839725	1.64918063	12.6304788	1.4499E-06	0.01702659	H3K4me1	LD
chr5	3971434	3971435	INS	chr5_4156213_4157192	4.48452556	12.6251076	1.4546E-06	0.01702659	H3K4me1	LD
chr6	80007449	80007450	INS	chr6_80082379_80083809	2.00986333	12.6554221	1.4281E-06	0.01702659	H3K4me1	LD
chr5	4045157	4045158	INS	chr5_4156213_4157192	4.48452556	12.6251076	1.4546E-06	0.01702659	H3K4me1	LD
chr9	126359771	126359772	INS	chr9_126499353_126499695	1.29206391	12.6347007	1.4462E-06	0.01702659	H3K4me1	LD
chr1	164346099	164346100	INS	chr1_164170457_164170775	-0.923565	-12.653901	1.4294E-06	0.01702659	H3K4me1	LD
chr13	28833746	28833747	INS	chr13_28963122_28964317	3.90709739	12.5383871	1.5334E-06	0.01780865	H3K4me1	LD
chr13	29034487	29034488	INS	chr13_28963122_28964317	3.90709739	12.5383871	1.5334E-06	0.01780865	H3K4me1	LD
chr11	20834491	20834677	DEL	chr11_20766373_20767808	1.85032	12.4917062	1.5778E-06	0.01783641	H3K4me1	LD
chr11	20890182	20890256	DEL	chr11_20766373_20767808	1.85032	12.4917062	1.5778E-06	0.01783641	H3K4me1	LD
chr3	33375700	33377288	DEL	chr3_33430693_33431976	1.20790924	12.5057906	1.5642E-06	0.01783641	H3K4me1	LD
chr3	33374425	33375258	DEL	chr3_33430693_33431976	1.20790924	12.5057906	1.5642E-06	0.01783641	H3K4me1	LD
chr3	33378973	33379109	DEL	chr3_33430693_33431976	1.20790924	12.5057906	1.5642E-06	0.01783641	H3K4me1	LD

chr11	20793186	20793187	INS	chr11_20766373_20767808	1.85032	12.4917062	1.5778E-06	0.01783641	H3K4me1	LD
chr11	20806124	20806125	INS	chr11_20766373_20767808	1.85032	12.4917062	1.5778E-06	0.01783641	H3K4me1	LD
chr7	7558253	7558254	INS	chr7_7530896_7532013	1.21195906	12.4569082	1.6118E-06	0.01815199	H3K4me1	LD
chr6	137195264	137195347	DEL	chr6_137117100_137118637	2.39992756	12.4103405	1.6586E-06	0.01833208	H3K4me1	LD
chr6	137253415	137256323	DEL	chr6_137117100_137118637	2.39992756	12.4103405	1.6586E-06	0.01833208	H3K4me1	LD
chr6	137250829	137250945	DEL	chr6_137117100_137118637	2.39992756	12.4103405	1.6586E-06	0.01833208	H3K4me1	LD
chr6	137252280	137252281	INS	chr6_137117100_137118637	2.39992756	12.4103405	1.6586E-06	0.01833208	H3K4me1	LD
chr6	137266730	137266731	INS	chr6_137117100_137118637	2.39992756	12.4103405	1.6586E-06	0.01833208	H3K4me1	LD
chr7	120319759	120319760	INS	chr7_120438990_120440092	1.15681569	12.3919283	1.6775E-06	0.01840447	H3K4me1	LD
chr7	120606954	120606955	INS	chr7_120438990_120440092	1.15681569	12.3919283	1.6775E-06	0.01840447	H3K4me1	LD
chr16	71784615	71784616	INS	chr16_71744995_71746141	3.73554	12.3737126	1.6965E-06	0.01854413	H3K4me1	LD
chr14	56839649	56839838	DEL	chr14_56700378_56700915	2.89498789	12.2840247	1.7935E-06	0.01925005	H3K4me1	LD
chr14	56829778	56830093	DEL	chr14_56700378_56700915	2.89498789	12.2840247	1.7935E-06	0.01925005	H3K4me1	LD

chr14	56769952	56769953	INS	chr14_56700378_56700915	2.89498789	12.2840247	1.7935E-06	0.01925005	H3K4me1	LD
chr14	56701908	56701909	INS	chr14_56700378_56700915	2.89498789	12.2840247	1.7935E-06	0.01925005	H3K4me1	LD
chr14	56809512	56809513	INS	chr14_56700378_56700915	2.89498789	12.2840247	1.7935E-06	0.01925005	H3K4me1	LD
chr7	102537386	102537387	INS	chr7_102542106_102543396	1.42405842	12.2728811	1.8059E-06	0.01931421	H3K4me1	LD
chr8	16653262	16654448	DEL	chr8_16671358_16671708	2.35220556	12.1457955	1.9553E-06	0.02066985	H3K4me1	LD
chr8	16714877	16715175	DEL	chr8_16671358_16671708	2.35220556	12.1457955	1.9553E-06	0.02066985	H3K4me1	LD
NW_018084968.1	2102704	2102705	INS	chr5_11853072_11854640	0.86524306	12.1415461	1.9605E-06	0.02066985	H3K4me1	LD
chr8	16725962	16725963	INS	chr8_16671358_16671708	2.35220556	12.1457955	1.9553E-06	0.02066985	H3K4me1	LD
chr1	6592901	6593070	DEL	chr1_6414041_6415024	3.62037194	12.1095666	2.0003E-06	0.02101538	H3K4me1	LD
chr3	573316	573380	DEL	chr3_399380_400048	2.03779389	12.0600939	2.0638E-06	0.02145436	H3K4me1	LD
chr7	97841067	97841068	INS	chr7_97813068_97813953	-3.8235329	-12.068818	2.0524E-06	0.02145436	H3K4me1	LD
chr3	491138	491139	INS	chr3_399380_400048	2.03779389	12.0600939	2.0638E-06	0.02145436	H3K4me1	LD
chr7	1748745	1748798	DEL	chr7_1579168_1579973	1.53449444	12.0175492	2.1201E-06	0.02181139	H3K4me1	LD

chr7	1523263	1523264	INS	chr7_1579168_1579973	1.53449444	12.0175492	2.1201E-06	0.02181139	H3K4me1	LD
chr7	1569174	1569175	INS	chr7_1579168_1579973	1.53449444	12.0175492	2.1201E-06	0.02181139	H3K4me1	LD
chr7	102537386	102537387	INS	chr7_102551149_102552237	1.38063458	11.9799673	2.1713E-06	0.02218474	H3K4me1	LD
chr3	117876512	117876513	INS	chr3_117888846_117890268	-1.5313142	-11.983728	2.1661E-06	0.02218474	H3K4me1	LD
chr4	65388280	65388281	INS	chr4_65527313_65528085	-1.6776087	-11.939689	2.2278E-06	0.02246143	H3K4me1	LD
chr4	65459247	65459248	INS	chr4_65527313_65528085	-1.6776087	-11.939689	2.2278E-06	0.02246143	H3K4me1	LD
chr4	65567274	65567275	INS	chr4_65527313_65528085	-1.6776087	-11.939689	2.2278E-06	0.02246143	H3K4me1	LD
chr12	59157782	59157783	INS	chr12_59358857_59359765	2.69124625	11.9390778	2.2286E-06	0.02246143	H3K4me1	LD
chr3	30964455	30964456	INS	chr3_30958813_30959687	2.16223839	11.9151245	2.263E-06	0.02257803	H3K4me1	LD
chr3	30978161	30978162	INS	chr3_30958813_30959687	2.16223839	11.9151245	2.263E-06	0.02257803	H3K4me1	LD
chr3	30980916	30980917	INS	chr3_30958813_30959687	2.16223839	11.9151245	2.263E-06	0.02257803	H3K4me1	LD
chr1	95392761	95392762	INS	chr1_95195607_95195953	1.23621153	11.8901179	2.2995E-06	0.02278919	H3K4me1	LD
chr1	43479695	43479899	DEL	chr1_43556057_43557074	2.87407902	11.8234387	2.4001E-06	0.0236284	H3K4me1	LD

chr1	43564232	43564328	DEL	chr1_43556057_43557074	2.87407902	11.8234387	2.4001E-06	0.0236284	H3K4me1	LD
chr4	109819693	109819901	DEL	chr4_109918182_109919309	1.96992931	11.6388619	2.7051E-06	0.02376755	H3K4me1	LD
chr4	109828442	109828656	DEL	chr4_109918182_109919309	1.96992931	11.6388619	2.7051E-06	0.02376755	H3K4me1	LD
chr4	109835679	109835820	DEL	chr4_109918182_109919309	1.96992931	11.6388619	2.7051E-06	0.02376755	H3K4me1	LD
chr9	107134288	107134477	DEL	chr9_107107369_107107742	1.84676403	11.6136992	2.7499E-06	0.02376755	H3K4me1	LD
chr3	113239926	113240286	DEL	chr3_113381070_113381549	2.01798867	11.6630611	2.6627E-06	0.02376755	H3K4me1	LD
chr3	113355860	113356126	DEL	chr3_113381070_113381549	2.01798867	11.6630611	2.6627E-06	0.02376755	H3K4me1	LD
chr3	113521159	113521232	DEL	chr3_113381070_113381549	2.01798867	11.6630611	2.6627E-06	0.02376755	H3K4me1	LD
chr6	161176492	161176837	DEL	chr6_161278203_161280636	-1.2261325	-11.708045	2.5859E-06	0.02376755	H3K4me1	LD
chr6	161244806	161245098	DEL	chr6_161278203_161280636	-1.2261325	-11.708045	2.5859E-06	0.02376755	H3K4me1	LD
chr6	161289014	161289306	DEL	chr6_161278203_161280636	-1.2261325	-11.708045	2.5859E-06	0.02376755	H3K4me1	LD
chr6	161303468	161303767	DEL	chr6_161278203_161280636	-1.2261325	-11.708045	2.5859E-06	0.02376755	H3K4me1	LD
chr6	161466406	161466700	DEL	chr6_161278203_161280636	-1.2261325	-11.708045	2.5859E-06	0.02376755	H3K4me1	LD

chr1	219380135	219380437	DEL	chr1_219318572_219319370	2.39704289	11.7686233	2.4864E-06	0.02376755	H3K4me1	LD
chr1	253854234	253854351	DEL	chr1_254023618_254024517	1.56688063	11.6141823	2.7491E-06	0.02376755	H3K4me1	LD
chr1	253897430	253897502	DEL	chr1_254023618_254024517	1.56688063	11.6141823	2.7491E-06	0.02376755	H3K4me1	LD
chr1	254215556	254215640	DEL	chr1_254023618_254024517	1.56688063	11.6141823	2.7491E-06	0.02376755	H3K4me1	LD
chr6	12920907	12921007	DEL	chr6_12891430_12891698	2.70011953	11.6754846	2.6413E-06	0.02376755	H3K4me1	LD
chr6	18892358	18893075	DEL	chr6_18889811_18890719	4.52307444	11.7477287	2.5203E-06	0.02376755	H3K4me1	LD
chr15	46166044	46166319	DEL	chr15_46184008_46184417	-1.3409833	-11.670711	2.6495E-06	0.02376755	H3K4me1	LD
chr13	10725409	10731409	DEL	chr13_10672335_10673388	1.75860656	11.684196	2.6263E-06	0.02376755	H3K4me1	LD
chr13	10813053	10813336	DEL	chr13_10672335_10673388	1.75860656	11.684196	2.6263E-06	0.02376755	H3K4me1	LD
chr13	10820210	10820368	DEL	chr13_10672335_10673388	1.75860656	11.684196	2.6263E-06	0.02376755	H3K4me1	LD
chr14	79615098	79615099	INS	chr14_79685549_79685902	1.70379104	11.7683244	2.4869E-06	0.02376755	H3K4me1	LD
chr14	79605943	79605944	INS	chr14_79685549_79685902	1.70379104	11.7683244	2.4869E-06	0.02376755	H3K4me1	LD
chr7	76689077	76689078	INS	chr7_76838014_76838841	1.2442675	11.7026316	2.5951E-06	0.02376755	H3K4me1	LD

chr4	109736435	109736436	INS	chr4_109918182_109919309	1.96992931	11.6388619	2.7051E-06	0.02376755	H3K4me1	LD
chr4	109830894	109830895	INS	chr4_109918182_109919309	1.96992931	11.6388619	2.7051E-06	0.02376755	H3K4me1	LD
chr9	107195740	107195741	INS	chr9_107107369_107107742	1.84676403	11.6136992	2.7499E-06	0.02376755	H3K4me1	LD
chr6	161239401	161239402	INS	chr6_161278203_161280636	-1.2261325	-11.708045	2.5859E-06	0.02376755	H3K4me1	LD
chr6	161240038	161240039	INS	chr6_161278203_161280636	-1.2261325	-11.708045	2.5859E-06	0.02376755	H3K4me1	LD
chr6	161319824	161319825	INS	chr6_161278203_161280636	-1.2261325	-11.708045	2.5859E-06	0.02376755	H3K4me1	LD
chr1	253841796	253841797	INS	chr1_254023618_254024517	1.56688063	11.6141823	2.7491E-06	0.02376755	H3K4me1	LD
chr1	253838467	253838468	INS	chr1_254023618_254024517	1.56688063	11.6141823	2.7491E-06	0.02376755	H3K4me1	LD
chr1	253846080	253846081	INS	chr1_254023618_254024517	1.56688063	11.6141823	2.7491E-06	0.02376755	H3K4me1	LD
chr1	253921721	253921722	INS	chr1_254023618_254024517	1.56688063	11.6141823	2.7491E-06	0.02376755	H3K4me1	LD
chr1	253989206	253989207	INS	chr1_254023618_254024517	1.56688063	11.6141823	2.7491E-06	0.02376755	H3K4me1	LD
chr13	10578694	10578695	INS	chr13_10672335_10673388	1.75860656	11.684196	2.6263E-06	0.02376755	H3K4me1	LD
chr13	10709196	10709197	INS	chr13_10672335_10673388	1.75860656	11.684196	2.6263E-06	0.02376755	H3K4me1	LD

chr13	10830239	10830240	INS	chr13_10672335_10673388	1.75860656	11.684196	2.6263E-06	0.02376755	H3K4me1	LD
chr13	10825319	10825320	INS	chr13_10672335_10673388	1.75860656	11.684196	2.6263E-06	0.02376755	H3K4me1	LD
chr14	26841280	26841281	INS	chr14_26650250_26651462	1.32996617	11.5841316	2.8037E-06	0.02416199	H3K4me1	LD
chr13	70081032	70081032	BND	chr13_70029590_70030976	7.355585	11.5300638	2.9051E-06	0.02464302	H3K4me1	LD
chr3	11286561	11286845	DEL	chr3_11213259_11214124	2.03141556	11.527887	2.9092E-06	0.02464302	H3K4me1	LD
chr3	11408791	11409068	DEL	chr3_11213259_11214124	2.03141556	11.527887	2.9092E-06	0.02464302	H3K4me1	LD
chr13	70081007	70081008	INS	chr13_70029590_70030976	3.6777925	11.5300638	2.9051E-06	0.02464302	H3K4me1	LD
chr3	11314854	11314855	INS	chr3_11213259_11214124	2.03141556	11.527887	2.9092E-06	0.02464302	H3K4me1	LD
chr18	26478102	26478223	DEL	chr18_26624833_26625359	1.27188925	11.5019344	2.9594E-06	0.02499698	H3K4me1	LD
chr6	87318960	87319219	DEL	chr6_87162806_87163148	1.15339528	11.4830421	2.9966E-06	0.02509686	H3K4me1	LD
chr3	107954698	107954778	DEL	chr3_108006778_108007144	4.81099549	11.4872195	2.9883E-06	0.02509686	H3K4me1	LD
chr13	198062980	198062981	INS	chr13_197864672_197865169	1.18530506	11.4864376	2.9899E-06	0.02509686	H3K4me1	LD
chr17	60879191	60879455	DEL	chr17_61043563_61044111	1.15424656	11.4251667	3.1137E-06	0.02567205	H3K4me1	LD

chr2	136627706	136627999	DEL	chr2_136494942_136495389	2.43489714	11.4214001	3.1215E-06	0.02567205	H3K4me1	LD
chr16	54786690	54786780	DEL	chr16_54902424_54902822	1.20751756	11.4148221	3.1351E-06	0.02567205	H3K4me1	LD
chr16	54981565	54981875	DEL	chr16_54902424_54902822	1.20751756	11.4148221	3.1351E-06	0.02567205	H3K4me1	LD
chr15	80795841	80795842	INS	chr15_80879780_80880160	1.12466	11.4158195	3.1331E-06	0.02567205	H3K4me1	LD
chr2	88883463	88883464	INS	chr2_88883002_88883493	2.56833083	11.4069354	3.1516E-06	0.02567205	H3K4me1	LD
chr11	4671239	4671240	INS	chr11_4553364_4555331	0.40491575	11.4088053	3.1477E-06	0.02567205	H3K4me1	LD
chr17	5710462	5710463	INS	chr17_5747262_5747632	3.45344567	11.4310672	3.1015E-06	0.02567205	H3K4me1	LD
chr17	5798124	5798125	INS	chr17_5747262_5747632	3.45344567	11.4310672	3.1015E-06	0.02567205	H3K4me1	LD
chr17	5821316	5821317	INS	chr17_5747262_5747632	3.45344567	11.4310672	3.1015E-06	0.02567205	H3K4me1	LD
chr8	75508547	75508741	DEL	chr8_75481149_75481604	2.87754594	11.2617955	3.4728E-06	0.02667278	H3K4me1	LD
chr17	51250361	51250654	DEL	chr17_51441033_51442801	2.3403899	11.3293169	3.319E-06	0.02667278	H3K4me1	LD
chr17	51436675	51436975	DEL	chr17_51441033_51442801	2.3403899	11.3293169	3.319E-06	0.02667278	H3K4me1	LD
chr8	110099584	110099916	DEL	chr8_110161625_110163159	1.22396656	11.25858	3.4803E-06	0.02667278	H3K4me1	LD

chr8	110121017	110121085	DEL	chr8_110161625_110163159	1.22396656	11.25858	3.4803E-06	0.02667278	H3K4me1	LD
chr8	110137183	110137476	DEL	chr8_110161625_110163159	1.22396656	11.25858	3.4803E-06	0.02667278	H3K4me1	LD
chr3	15469022	15469137	DEL	chr3_15349112_15349380	1.28033761	11.2721927	3.4486E-06	0.02667278	H3K4me1	LD
chr8	18038594	18038651	DEL	chr8_18110391_18110758	1.19329178	11.2734302	3.4457E-06	0.02667278	H3K4me1	LD
chr3	26994432	26995024	DEL	chr3_26996137_26996833	1.5673585	11.2522681	3.4951E-06	0.02667278	H3K4me1	LD
chr3	26997405	26997674	DEL	chr3_26996137_26996833	1.5673585	11.2522681	3.4951E-06	0.02667278	H3K4me1	LD
chr3	27141356	27149021	DEL	chr3_26996137_26996833	1.5673585	11.2522681	3.4951E-06	0.02667278	H3K4me1	LD
chr8	75610670	75610671	INS	chr8_75481149_75481604	2.87754594	11.2617955	3.4728E-06	0.02667278	H3K4me1	LD
chr7	54854964	54854965	INS	chr7_54793390_54795112	2.97840048	11.2431121	3.5167E-06	0.02667278	H3K4me1	LD
chr7	54833578	54833579	INS	chr7_54793390_54795112	2.97840048	11.2431121	3.5167E-06	0.02667278	H3K4me1	LD
chr8	110102416	110102417	INS	chr8_110161625_110163159	1.22396656	11.25858	3.4803E-06	0.02667278	H3K4me1	LD
chr8	110138787	110138788	INS	chr8_110161625_110163159	1.22396656	11.25858	3.4803E-06	0.02667278	H3K4me1	LD
chr9	126510647	126510648	INS	chr9_126499353_126499695	1.2761125	11.2472721	3.5069E-06	0.02667278	H3K4me1	LD

chr9	126634135	126634136	INS	chr9_126499353_126499695	1.2761125	11.2472721	3.5069E-06	0.02667278	H3K4me1	LD
chr6	162919830	162919831	INS	chr6_162788854_162790248	0.79544788	11.2800098	3.4305E-06	0.02667278	H3K4me1	LD
chr3	15494259	15494260	INS	chr3_15349112_15349380	1.28033761	11.2721927	3.4486E-06	0.02667278	H3K4me1	LD
chr8	18134916	18134917	INS	chr8_18110391_18110758	1.19329178	11.2734302	3.4457E-06	0.02667278	H3K4me1	LD
chr8	18249002	18249003	INS	chr8_18110391_18110758	1.19329178	11.2734302	3.4457E-06	0.02667278	H3K4me1	LD
chr8	18272276	18272277	INS	chr8_18110391_18110758	1.19329178	11.2734302	3.4457E-06	0.02667278	H3K4me1	LD
chr8	22858958	22858959	INS	chr8_22821204_22822139	1.24204596	11.2850433	3.4189E-06	0.02667278	H3K4me1	LD
chr1	35043395	35043484	DEL	chr1_34869846_34870933	1.29151633	11.131747	3.7918E-06	0.02729718	H3K4me1	LD
chr17	30271672	30271880	DEL	chr17_30329430_30329675	2.19069097	11.134605	3.7845E-06	0.02729718	H3K4me1	LD
chr16	47312992	47313551	DUP	chr16_47476398_47477754	4.40735238	11.1488816	3.748E-06	0.02729718	H3K4me1	LD
chr16	47467984	47483275	DUP	chr16_47476398_47477754	4.40735238	11.1488816	3.748E-06	0.02729718	H3K4me1	LD
chr13	84945487	84945488	INS	chr13_84771555_84772624	1.61562644	11.1644933	3.7085E-06	0.02729718	H3K4me1	LD
chr13	84953448	84953449	INS	chr13_84771555_84772624	1.61562644	11.1644933	3.7085E-06	0.02729718	H3K4me1	LD

chr13	84948141	84948142	INS	chr13_84771555_84772624	1.61562644	11.1644933	3.7085E-06	0.02729718	H3K4me1	LD
chr2	144768721	144768722	INS	chr2_144822982_144825539	1.42206979	11.1638555	3.7101E-06	0.02729718	H3K4me1	LD
chr2	144827456	144827457	INS	chr2_144822982_144825539	1.42206979	11.1638555	3.7101E-06	0.02729718	H3K4me1	LD
chr14	10096782	10096783	INS	chr14_10205615_10206418	2.85239667	11.146685	3.7536E-06	0.02729718	H3K4me1	LD
chr1	34707070	34707071	INS	chr1_34869846_34870933	1.29151633	11.131747	3.7918E-06	0.02729718	H3K4me1	LD
chr1	35012975	35012976	INS	chr1_34869846_34870933	2.58303267	11.131747	3.7918E-06	0.02729718	H3K4me1	LD
chr1	35004944	35004945	INS	chr1_34869846_34870933	1.29151633	11.131747	3.7918E-06	0.02729718	H3K4me1	LD
chr16	47313537	47313538	INS	chr16_47476398_47477754	2.20367619	11.1488816	3.748E-06	0.02729718	H3K4me1	LD
chr16	47334985	47334986	INS	chr16_47476398_47477754	2.20367619	11.1488816	3.748E-06	0.02729718	H3K4me1	LD
chr7	29425953	29425954	INS	chr7_29527234_29527547	2.18923986	11.1831687	3.6619E-06	0.02729718	H3K4me1	LD
chr17	30317906	30317907	INS	chr17_30329430_30329675	2.19069097	11.134605	3.7845E-06	0.02729718	H3K4me1	LD
chr3	19371667	19373346	DEL	chr3_19414343_19415104	1.54450874	11.1085501	3.8521E-06	0.02733409	H3K4me1	LD
chr3	19428165	19428403	DEL	chr3_19414343_19415104	1.54450874	11.1085501	3.8521E-06	0.02733409	H3K4me1	LD

chr3	19525043	19525113	DEL	chr3_19414343_19415104	1.54450874	11.1085501	3.8521E-06	0.02733409	H3K4me1	LD
chr17	14626451	14626551	DEL	chr17_14717861_14719946	1.89077505	11.12124	3.819E-06	0.02733409	H3K4me1	LD
chr7	11487794	11487795	INS	chr7_11619076_11619508	-1.1137429	-11.116355	3.8317E-06	0.02733409	H3K4me1	LD
chr3	19443399	19443400	INS	chr3_19414343_19415104	1.54450874	11.1085501	3.8521E-06	0.02733409	H3K4me1	LD
chr12	53725374	53726137	DEL	chr12_53726247_53726647	2.74558172	11.0793587	3.9295E-06	0.02750962	H3K4me1	LD
chr12	53729524	53729993	DEL	chr12_53726247_53726647	2.74558172	11.0793587	3.9295E-06	0.02750962	H3K4me1	LD
chr18	9041706	9041775	DEL	chr18_9230122_9230447	2.91200072	11.0748503	3.9416E-06	0.02750962	H3K4me1	LD
chr18	9211301	9211387	DEL	chr18_9230122_9230447	2.91200072	11.0748503	3.9416E-06	0.02750962	H3K4me1	LD
chr18	9311351	9311531	DEL	chr18_9230122_9230447	2.91200072	11.0748503	3.9416E-06	0.02750962	H3K4me1	LD
chr10	33272619	33272620	INS	chr10_33397451_33397758	3.51579778	11.0929581	3.8932E-06	0.02750962	H3K4me1	LD
chr18	9260222	9260223	INS	chr18_9230122_9230447	2.91200072	11.0748503	3.9416E-06	0.02750962	H3K4me1	LD
chr9	33837090	33837090	BND	chr9_34035572_34036026	1.90651794	11.0491608	4.0114E-06	0.02767295	H3K4me1	LD
chr4	59734526	59734856	DEL	chr4_59707887_59708224	2.23546319	11.0491066	4.0116E-06	0.02767295	H3K4me1	LD

chr9	33850463	33850464	INS	chr9_34035572_34036026	1.90651794	11.0491608	4.0114E-06	0.02767295	H3K4me1	LD
chr9	33837071	33837072	INS	chr9_34035572_34036026	1.90651794	11.0491608	4.0114E-06	0.02767295	H3K4me1	LD
chr9	34100911	34100912	INS	chr9_34035572_34036026	1.90651794	11.0491608	4.0114E-06	0.02767295	H3K4me1	LD
chr15	74531184	74531476	DEL	chr15_74487914_74489864	-2.5449173	-11.024987	4.0783E-06	0.02781292	H3K4me1	LD
chr15	74536841	74537202	DEL	chr15_74487914_74489864	-2.5449173	-11.024987	4.0783E-06	0.02781292	H3K4me1	LD
chr6	82040130	82040190	DEL	chr6_82053099_82053528	1.22924338	11.0148861	4.1067E-06	0.02781292	H3K4me1	LD
chr6	82127312	82127589	DEL	chr6_82053099_82053528	1.22924338	11.0148861	4.1067E-06	0.02781292	H3K4me1	LD
chr15	74498443	74498444	INS	chr15_74487914_74489864	-2.5449173	-11.024987	4.0783E-06	0.02781292	H3K4me1	LD
chr18	25537822	25537823	INS	chr18_25671484_25672834	1.77180306	11.0181104	4.0976E-06	0.02781292	H3K4me1	LD
chr18	25671236	25671237	INS	chr18_25671484_25672834	1.77180306	11.0181104	4.0976E-06	0.02781292	H3K4me1	LD
chr18	25673649	25673650	INS	chr18_25671484_25672834	1.77180306	11.0181104	4.0976E-06	0.02781292	H3K4me1	LD
chr18	33336460	33336690	DEL	chr18_33457800_33458126	1.18573238	11.0031428	4.1399E-06	0.0279742	H3K4me1	LD
chr6	103190798	103190799	INS	chr6_103130248_103132461	0.75764792	10.9981999	4.154E-06	0.02800565	H3K4me1	LD

chr12	52850676	52851043	DEL	chr12_53038304_53040542	2.41985419	10.8727754	4.5296E-06	0.0285475	H3K4me1	LD
chr2	136396380	136396695	DEL	chr2_136494942_136495389	2.39359813	10.8657437	4.5518E-06	0.0285475	H3K4me1	LD
chr2	136500656	136500961	DEL	chr2_136494942_136495389	2.39359813	10.8657437	4.5518E-06	0.0285475	H3K4me1	LD
chr13	197888482	197888614	DEL	chr13_197864672_197865169	1.18874356	10.8714315	4.5338E-06	0.0285475	H3K4me1	LD
chr3	573316	573380	DEL	chr3_498562_500166	4.01944278	10.8586603	4.5742E-06	0.0285475	H3K4me1	LD
chr9	9360919	9361222	DEL	chr9_9493220_9493751	1.86778319	10.9395014	4.3253E-06	0.0285475	H3K4me1	LD
chr7	13960177	13960316	DEL	chr7_13883227_13883726	1.61061875	10.9632233	4.2551E-06	0.0285475	H3K4me1	LD
chr8	31966170	31966514	DEL	chr8_32002868_32003686	0.98687317	10.9225129	4.3763E-06	0.0285475	H3K4me1	LD
chr8	31976589	31976918	DEL	chr8_32002868_32003686	0.98687317	10.9225129	4.3763E-06	0.0285475	H3K4me1	LD
chr4	35732092	35732608	DEL	chr4_35553998_35554306	1.27680618	10.9537953	4.2828E-06	0.0285475	H3K4me1	LD
chr12	46004695	46004746	DEL	chr12_45950040_45951227	1.86920139	10.848777	4.6057E-06	0.0285475	H3K4me1	LD
chr12	46115051	46115346	DEL	chr12_45950040_45951227	1.86920139	10.848777	4.6057E-06	0.0285475	H3K4me1	LD
chr3	58398188	58398189	INS	chr3_58464207_58466014	4.5562825	10.8624347	4.5622E-06	0.0285475	H3K4me1	LD

chr3	58398188	58398189	INS	chr3_58476439_58478432	5.19253125	10.8798445	4.5075E-06	0.0285475	H3K4me1	LD
chr3	58568874	58568875	INS	chr3_58464207_58466014	4.5562825	10.8624347	4.5622E-06	0.0285475	H3K4me1	LD
chr3	58568874	58568875	INS	chr3_58476439_58478432	5.19253125	10.8798445	4.5075E-06	0.0285475	H3K4me1	LD
chr10	41213531	41213532	INS	chr10_41089246_41090254	3.08904917	10.8624168	4.5623E-06	0.0285475	H3K4me1	LD
chr3	491138	491139	INS	chr3_498562_500166	4.01944278	10.8586603	4.5742E-06	0.0285475	H3K4me1	LD
chr6	9714587	9714588	INS	chr6_9822645_9823545	0.82163433	10.8650607	4.5539E-06	0.0285475	H3K4me1	LD
chr6	9804876	9804877	INS	chr6_9822645_9823545	0.82163433	10.8650607	4.5539E-06	0.0285475	H3K4me1	LD
chr6	9903275	9903276	INS	chr6_9822645_9823545	0.82163433	10.8650607	4.5539E-06	0.0285475	H3K4me1	LD
chr6	9994568	9994569	INS	chr6_9822645_9823545	0.82163433	10.8650607	4.5539E-06	0.0285475	H3K4me1	LD
chr3	678590	678591	INS	chr3_498562_500166	4.01944278	10.8586603	4.5742E-06	0.0285475	H3K4me1	LD
chr9	9360292	9360293	INS	chr9_9493220_9493751	1.86778319	10.9395014	4.3253E-06	0.0285475	H3K4me1	LD
chr7	13707925	13707926	INS	chr7_13883227_13883726	1.61061875	10.9632233	4.2551E-06	0.0285475	H3K4me1	LD
chr8	31807892	31807893	INS	chr8_32002868_32003686	0.98687317	10.9225129	4.3763E-06	0.0285475	H3K4me1	LD

chr8	31905621	31905622	INS	chr8_32002868_32003686	0.98687317	10.9225129	4.3763E-06	0.0285475	H3K4me1	LD
chr4	35512559	35512560	INS	chr4_35553998_35554306	1.27680618	10.9537953	4.2828E-06	0.0285475	H3K4me1	LD
chr12	45832290	45832291	INS	chr12_45950040_45951227	1.86920139	10.848777	4.6057E-06	0.0285475	H3K4me1	LD
chr12	45926011	45926012	INS	chr12_45950040_45951227	1.86920139	10.848777	4.6057E-06	0.0285475	H3K4me1	LD
chr12	45987188	45987189	INS	chr12_45950040_45951227	1.86920139	10.848777	4.6057E-06	0.0285475	H3K4me1	LD
chr12	46004130	46004131	INS	chr12_45950040_45951227	1.86920139	10.848777	4.6057E-06	0.0285475	H3K4me1	LD
chr12	46129611	46129612	INS	chr12_45950040_45951227	1.86920139	10.848777	4.6057E-06	0.0285475	H3K4me1	LD
chr12	46117954	46117955	INS	chr12_45950040_45951227	1.86920139	10.848777	4.6057E-06	0.0285475	H3K4me1	LD
chr12	46150805	46150806	INS	chr12_45950040_45951227	1.86920139	10.848777	4.6057E-06	0.0285475	H3K4me1	LD
chr6	128327579	128327580	INS	chr6_128226028_128227104	1.24943833	10.8310647	4.6628E-06	0.02876234	H3K4me1	LD
chr6	13550582	13550725	DEL	chr6_13557271_13558305	-1.5656325	-10.802322	4.7572E-06	0.02922306	H3K4me1	LD
chr8	11076147	11076208	DEL	chr8_10997171_10997601	1.7103325	10.7765516	4.8436E-06	0.02944963	H3K4me1	LD
chr5	52631389	52631390	INS	chr5_52452566_52453486	1.58804375	10.7787571	4.8361E-06	0.02944963	H3K4me1	LD

chr14	129377996	129377997	INS	chr14_129183076_129184065	1.67116688	10.7845382	4.8166E-06	0.02944963	H3K4me1	LD
chr8	11083526	11083527	INS	chr8_10997171_10997601	1.7103325	10.7765516	4.8436E-06	0.02944963	H3K4me1	LD
chr8	11102963	11102964	INS	chr8_10997171_10997601	1.7103325	10.7765516	4.8436E-06	0.02944963	H3K4me1	LD
chr7	80335135	80335136	INS	chr7_80334683_80337389	1.12983458	10.7430035	4.9587E-06	0.03008808	H3K4me1	LD
chr13	197442220	197442520	DEL	chr13_197422507_197423261	2.05939275	10.7344552	4.9885E-06	0.0300997	H3K4me1	LD
chr14	100856308	100856309	INS	chr14_100963113_100963437	2.27062269	10.730864	5.0011E-06	0.0300997	H3K4me1	LD
chr9	9227917	9227918	INS	chr9_9243684_9244061	2.47765167	10.7321985	4.9965E-06	0.0300997	H3K4me1	LD
chr14	56643463	56643734	DEL	chr14_56730689_56731255	3.17908483	10.6904519	5.1453E-06	0.0303801	H3K4me1	LD
chr14	56853561	56853708	DEL	chr14_56730689_56731255	3.17908483	10.6904519	5.1453E-06	0.0303801	H3K4me1	LD
chr14	56882611	56882899	DEL	chr14_56730689_56731255	3.17908483	10.6904519	5.1453E-06	0.0303801	H3K4me1	LD
chr15	79152878	79153151	DEL	chr15_79085954_79086537	-2.28009	-10.695581	5.1267E-06	0.0303801	H3K4me1	LD
chr1	222385368	222385369	INS	chr1_222291552_222292011	1.11115083	10.7092579	5.0776E-06	0.0303801	H3K4me1	LD
chr1	222421258	222421259	INS	chr1_222291552_222292011	1.11115083	10.7092579	5.0776E-06	0.0303801	H3K4me1	LD

chr12	48007548	48007549	INS	chr12_48197087_48198191	2.82863683	10.6891715	5.1499E-06	0.0303801	H3K4me1	LD
chr14	56741899	56742202	INV	chr14_56730689_56731255	3.17908483	10.6904519	5.1453E-06	0.0303801	H3K4me1	LD
chr6	65359957	65361570	DEL	chr6_65324703_65324993	3.26692938	10.6406675	5.3292E-06	0.03125199	H3K4me1	LD
chr6	65319617	65319618	INS	chr6_65324703_65324993	3.26692938	10.6406675	5.3292E-06	0.03125199	H3K4me1	LD
chr1	252785703	252785939	DEL	chr1_252651338_252652071	1.26363125	10.6291082	5.373E-06	0.03144658	H3K4me1	LD
chr8	94100835	94100836	INS	chr8_94055021_94056101	1.31036956	10.6100802	5.4459E-06	0.03156021	H3K4me1	LD
chr8	94119429	94119430	INS	chr8_94055021_94056101	1.31036956	10.6100802	5.4459E-06	0.03156021	H3K4me1	LD
chr8	94207293	94207294	INS	chr8_94055021_94056101	1.31036956	10.6100802	5.4459E-06	0.03156021	H3K4me1	LD
chr2	73761636	73761637	INS	chr2_73750193_73750539	2.60521181	10.6074429	5.4561E-06	0.03156021	H3K4me1	LD
chr9	33837090	33837090	BND	chr9_33969040_33969471	1.89226989	10.556014	5.6592E-06	0.03189647	H3K4me1	LD
chr8	130917917	130918239	DEL	chr8_131115487_131115866	0.92160857	10.5487328	5.6886E-06	0.03189647	H3K4me1	LD
chr2	138323185	138323744	DEL	chr2_138437379_138438245	0.86409394	10.5521612	5.6747E-06	0.03189647	H3K4me1	LD
chr6	12938852	12939187	DEL	chr6_12891430_12891698	2.66678472	10.546761	5.6966E-06	0.03189647	H3K4me1	LD

chr6	13061421	13062487	DEL	chr6_12891430_12891698	2.66678472	10.546761	5.6966E-06	0.03189647	H3K4me1	LD
chr7	13213175	13213446	DEL	chr7_13213486_13213750	2.53766833	10.5619412	5.6354E-06	0.03189647	H3K4me1	LD
chr14	88590639	88590640	INS	chr14_88683819_88684365	1.98160444	10.5729588	5.5914E-06	0.03189647	H3K4me1	LD
chr2	103924277	103924278	INS	chr2_103771720_103772641	2.10762556	10.5555232	5.6612E-06	0.03189647	H3K4me1	LD
chr13	115451161	115451162	INS	chr13_115642559_115643656	-1.2038808	-10.551107	5.679E-06	0.03189647	H3K4me1	LD
chr13	115520818	115520819	INS	chr13_115642559_115643656	-1.2038808	-10.551107	5.679E-06	0.03189647	H3K4me1	LD
chr5	99166744	99166745	INS	chr5_99349577_99350534	3.54066211	10.5677776	5.612E-06	0.03189647	H3K4me1	LD
chr5	99436413	99436414	INS	chr5_99349577_99350534	3.54066211	10.5677776	5.612E-06	0.03189647	H3K4me1	LD
chr2	138443640	138443641	INS	chr2_138437379_138438245	0.86409394	10.5521612	5.6747E-06	0.03189647	H3K4me1	LD
chr6	12848141	12848142	INS	chr6_12891430_12891698	2.66678472	10.546761	5.6966E-06	0.03189647	H3K4me1	LD
chr9	33850463	33850464	INS	chr9_33969040_33969471	1.89226989	10.556014	5.6592E-06	0.03189647	H3K4me1	LD
chr9	33837071	33837072	INS	chr9_33969040_33969471	1.89226989	10.556014	5.6592E-06	0.03189647	H3K4me1	LD
chr9	34100911	34100912	INS	chr9_33969040_33969471	1.89226989	10.556014	5.6592E-06	0.03189647	H3K4me1	LD

chr18	1485021	1485305	DEL	chr18_1510332_1511375	2.1132261	10.5237421	5.7909E-06	0.03218213	H3K4me1	LD
chr18	1429075	1429076	INS	chr18_1510332_1511375	2.1132261	10.5237421	5.7909E-06	0.03218213	H3K4me1	LD
chr18	1516790	1516791	INS	chr18_1510332_1511375	2.1132261	10.5237421	5.7909E-06	0.03218213	H3K4me1	LD
chr7	10079855	10079856	INS	chr7_9913754_9914713	1.05586778	10.5291699	5.7685E-06	0.03218213	H3K4me1	LD
chr9	9536267	9536324	DEL	chr9_9442765_9443440	1.06548194	10.5042941	5.872E-06	0.0322706	H3K4me1	LD
chr11	20807885	20808203	DEL	chr11_20836160_20838113	-0.7578713	-10.509757	5.8491E-06	0.0322706	H3K4me1	LD
chr11	20818444	20818534	DEL	chr11_20836160_20838113	-0.7578713	-10.509757	5.8491E-06	0.0322706	H3K4me1	LD
chr9	9298631	9298632	INS	chr9_9442765_9443440	1.06548194	10.5042941	5.872E-06	0.0322706	H3K4me1	LD
chr11	20837129	20837130	INS	chr11_20836160_20838113	-0.7578713	-10.509757	5.8491E-06	0.0322706	H3K4me1	LD
chr4	106676759	106677125	DEL	chr4_106679028_106680593	-2.8860508	-10.464485	6.0418E-06	0.03283996	H3K4me1	LD
chr4	106825723	106826005	DEL	chr4_106679028_106680593	-2.8860508	-10.464485	6.0418E-06	0.03283996	H3K4me1	LD
chr4	106523776	106523777	INS	chr4_106679028_106680593	-2.8860508	-10.464485	6.0418E-06	0.03283996	H3K4me1	LD
chr4	106582801	106582802	INS	chr4_106679028_106680593	-2.8860508	-10.464485	6.0418E-06	0.03283996	H3K4me1	LD

chr4	106827664	106827665	INS	chr4_106679028_106680593	-2.8860508	-10.464485	6.0418E-06	0.03283996	H3K4me1	LD
chr4	106822604	106822605	INS	chr4_106679028_106680593	-2.8860508	-10.464485	6.0418E-06	0.03283996	H3K4me1	LD
chr9	22858092	22860520	DEL	chr9_22908016_22908663	0.960052	10.4531196	6.0913E-06	0.03297707	H3K4me1	LD
chr3	25337721	25337722	INS	chr3_25263195_25264113	0.5746745	10.4510647	6.1003E-06	0.03297707	H3K4me1	LD
chr1	247388434	247388489	DEL	chr1_247469878_247470244	1.72278738	10.4329608	6.1803E-06	0.03334846	H3K4me1	LD
chr14	88636891	88636892	INS	chr14_88683819_88684365	1.96724417	10.4085792	6.2898E-06	0.03375546	H3K4me1	LD
chr1	183743486	183743562	DEL	chr1_183890945_183892076	-3.7587067	-10.396679	6.344E-06	0.03386309	H3K4me1	LD
chr1	183770544	183770604	DEL	chr1_183890945_183892076	-3.7587067	-10.396679	6.344E-06	0.03386309	H3K4me1	LD
chr1	183767037	183767038	INS	chr1_183890945_183892076	-3.7587067	-10.396679	6.344E-06	0.03386309	H3K4me1	LD
chr6	120071840	120071912	DEL	chr6_120040630_120042443	1.84609125	10.3625255	6.5025E-06	0.03452342	H3K4me1	LD
chr6	120073364	120073365	INS	chr6_120040630_120042443	1.84609125	10.3625255	6.5025E-06	0.03452342	H3K4me1	LD
chr15	24194165	24194166	INS	chr15_24123125_24123829	0.62698864	10.3536874	6.5443E-06	0.03468312	H3K4me1	LD
chr6	40585397	40585505	DEL	chr6_40702964_40703326	2.28167272	10.3305121	6.6552E-06	0.03496324	H3K4me1	LD

chr13	26196598	26196599	INS	chr13_26051708_26052381	1.55736813	10.3348838	6.6341E-06	0.03496324	H3K4me1	LD
chr3	122076066	122076067	INS	chr3_121991422_121993307	2.12166092	10.3303545	6.6559E-06	0.03496324	H3K4me1	LD
chr6	40591206	40591207	INS	chr6_40702964_40703326	2.28167272	10.3305121	6.6552E-06	0.03496324	H3K4me1	LD
chr8	41290153	41294443	DUP	chr8_41421827_41422298	2.18937938	10.3019787	6.7946E-06	0.03550343	H3K4me1	LD
chr8	41416313	41416314	INS	chr8_41421827_41422298	1.09468969	10.3019787	6.7946E-06	0.03550343	H3K4me1	LD
chr1	161767593	161767875	DEL	chr1_161890618_161892665	-1.377524	-10.288046	6.8638E-06	0.03555293	H3K4me1	LD
chr1	161884771	161884994	DEL	chr1_161890618_161892665	-1.377524	-10.288046	6.8638E-06	0.03555293	H3K4me1	LD
chr1	161907656	161907944	DEL	chr1_161890618_161892665	-1.377524	-10.288046	6.8638E-06	0.03555293	H3K4me1	LD
chr1	161883786	161883787	INS	chr1_161890618_161892665	-1.377524	-10.288046	6.8638E-06	0.03555293	H3K4me1	LD
chr1	162056924	162056925	INS	chr1_161890618_161892665	-1.377524	-10.288046	6.8638E-06	0.03555293	H3K4me1	LD
chr3	69976151	69976444	DEL	chr3_69980706_69981722	-1.1691477	-10.271594	6.9466E-06	0.03579475	H3K4me1	LD
chr3	70156089	70156090	INS	chr3_69980706_69981722	1.1691477	10.2715937	6.9466E-06	0.03579475	H3K4me1	LD
chr3	70174879	70174880	INS	chr3_69980706_69981722	1.1691477	10.2715937	6.9466E-06	0.03579475	H3K4me1	LD

chr16	47936485	47936815	DEL	chr16_48076940_48078363	-0.7835283	-10.262836	6.9912E-06	0.03583787	H3K4me1	LD
chr16	48068970	48069256	DEL	chr16_48076940_48078363	-0.7835283	-10.262836	6.9912E-06	0.03583787	H3K4me1	LD
chr9	33837090	33837090	BND	chr9_34000115_34000878	1.51275386	10.25246	7.0443E-06	0.03586313	H3K4me1	LD
chr9	33850463	33850464	INS	chr9_34000115_34000878	1.51275386	10.25246	7.0443E-06	0.03586313	H3K4me1	LD
chr9	33837071	33837072	INS	chr9_34000115_34000878	1.51275386	10.25246	7.0443E-06	0.03586313	H3K4me1	LD
chr9	34100911	34100912	INS	chr9_34000115_34000878	1.51275386	10.25246	7.0443E-06	0.03586313	H3K4me1	LD
chr7	53058253	53058254	INS	chr7_52980607_52981864	2.17655222	10.2459756	7.0778E-06	0.03597188	H3K4me1	LD
chr3	109064266	109064324	DEL	chr3_109010351_109010829	1.26028767	10.2388358	7.1149E-06	0.03603696	H3K4me1	LD
chr3	108976577	108976578	INS	chr3_109010351_109010829	1.26028767	10.2388358	7.1149E-06	0.03603696	H3K4me1	LD
chr2	32905739	32906017	DEL	chr2_32877205_32877910	1.66163208	10.2198849	7.2142E-06	0.03633059	H3K4me1	LD
chr2	33067581	33067880	DEL	chr2_32877205_32877910	1.66163208	10.2198849	7.2142E-06	0.03633059	H3K4me1	LD
chr9	92920761	92920762	INS	chr9_92952543_92953769	1.08498486	10.2184735	7.2217E-06	0.03633059	H3K4me1	LD
chr9	92869167	92869168	INS	chr9_92952543_92953769	1.08498486	10.2184735	7.2217E-06	0.03633059	H3K4me1	LD

chr14	125145189	125145500	DEL	chr14_125148067_125149975	-1.2515192	-10.181874	7.4183E-06	0.03667775	H3K4me1	LD
chr13	189546379	189548076	DEL	chr13_189438382_189439804	1.68828539	10.1781287	7.4387E-06	0.03667775	H3K4me1	LD
chr14	125286621	125286622	INS	chr14_125148067_125149975	-1.2515192	-10.181874	7.4183E-06	0.03667775	H3K4me1	LD
chr14	125346834	125346835	INS	chr14_125148067_125149975	-1.2515192	-10.181874	7.4183E-06	0.03667775	H3K4me1	LD
chr13	189346991	189346992	INS	chr13_189438382_189439804	1.68828539	10.1781287	7.4387E-06	0.03667775	H3K4me1	LD
chr15	25387134	25387135	INS	chr15_25475527_25475867	3.49096444	10.1825015	7.4149E-06	0.03667775	H3K4me1	LD
chr15	25421751	25421752	INS	chr15_25475527_25475867	3.49096444	10.1825015	7.4149E-06	0.03667775	H3K4me1	LD
chr15	25420664	25420665	INS	chr15_25475527_25475867	3.49096444	10.1825015	7.4149E-06	0.03667775	H3K4me1	LD
chr1	149783484	149784071	DEL	chr1_149768995_149770623	1.01809358	10.1703669	7.4813E-06	0.03670511	H3K4me1	LD
chr1	149833711	149834359	DEL	chr1_149768995_149770623	1.01809358	10.1703669	7.4813E-06	0.03670511	H3K4me1	LD
chr1	149785119	149785120	INS	chr1_149768995_149770623	1.01809358	10.1703669	7.4813E-06	0.03670511	H3K4me1	LD
chr11	7762352	7762631	DEL	chr11_7749155_7749514	1.51160639	10.148034	7.6054E-06	0.03717497	H3K4me1	LD
chr9	64300462	64300463	INS	chr9_64396066_64397029	2.74181476	10.1463905	7.6146E-06	0.03717497	H3K4me1	LD

chr5	91273146	91273676	DEL	chr5_91283512_91284030	0.94230331	10.1414471	7.6424E-06	0.03724956	H3K4me1	LD
chr8	17539888	17540200	DEL	chr8_17553552_17555509	1.8542671	10.1214919	7.7558E-06	0.03767878	H3K4me1	LD
chr16	53709679	53709680	INS	chr16_53766165_53766816	2.21604189	10.1137999	7.8E-06	0.03783177	H3K4me1	LD
chr14	931118	931440	DEL	chr14_1088471_1088814	2.91510479	10.0998665	7.8809E-06	0.03785322	H3K4me1	LD
chr14	1106319	1106588	DEL	chr14_1088471_1088814	2.91510479	10.0998665	7.8809E-06	0.03785322	H3K4me1	LD
chr14	1208295	1208350	DEL	chr14_1088471_1088814	2.91510479	10.0998665	7.8809E-06	0.03785322	H3K4me1	LD
chr8	6045852	6045853	INS	chr8_5935590_5935876	1.48819972	10.1026117	7.8649E-06	0.03785322	H3K4me1	LD
chr14	930357	930358	INS	chr14_1088471_1088814	2.91510479	10.0998665	7.8809E-06	0.03785322	H3K4me1	LD
chr11	2741070	2741252	DEL	chr11_2700057_2702956	3.19357789	10.0900607	7.9383E-06	0.03788427	H3K4me1	LD
chr11	2743815	2743868	DEL	chr11_2700057_2702956	3.19357789	10.0900607	7.9383E-06	0.03788427	H3K4me1	LD
chr11	2789307	2789469	DEL	chr11_2700057_2702956	3.19357789	10.0900607	7.9383E-06	0.03788427	H3K4me1	LD
chr11	2744435	2744436	INS	chr11_2700057_2702956	3.19357789	10.0900607	7.9383E-06	0.03788427	H3K4me1	LD
chr5	4038027	4038112	DEL	chr5_4158013_4158850	6.19752	10.074698	8.0292E-06	0.03797828	H3K4me1	LD

chr5	4058472	4058562	DEL	chr5_4158013_4158850	6.19752	10.074698	8.0292E-06	0.03797828	H3K4me1	LD
chr5	3971434	3971435	INS	chr5_4158013_4158850	6.19752	10.074698	8.0292E-06	0.03797828	H3K4me1	LD
chr5	4045157	4045158	INS	chr5_4158013_4158850	6.19752	10.074698	8.0292E-06	0.03797828	H3K4me1	LD
chr13	206027311	206027312	INS	chr13_205969935_205970380	1.50843215	10.0696356	8.0594E-06	0.03803501	H3K4me1	LD
chr1	119489297	119489369	DEL	chr1_119300112_119300865	1.67624611	10.0631834	8.0981E-06	0.03803649	H3K4me1	LD
chr1	119480076	119480077	INS	chr1_119300112_119300865	1.67624611	10.0631834	8.0981E-06	0.03803649	H3K4me1	LD
chr8	41416313	41416314	INS	chr8_41557092_41557439	2.61292828	10.067317	8.0733E-06	0.03803649	H3K4me1	LD
chr2	26626352	26626353	INS	chr2_26620979_26621994	1.81015956	10.0548665	8.1483E-06	0.03821183	H3K4me1	LD
chr3	11251232	11251521	DEL	chr3_11431947_11432998	-1.7457429	-10.020493	8.3594E-06	0.03851149	H3K4me1	LD
chr3	11437860	11437929	DEL	chr3_11431947_11432998	-1.7457429	-10.020493	8.3594E-06	0.03851149	H3K4me1	LD
chr3	11519155	11519443	DEL	chr3_11431947_11432998	1.74574292	10.020493	8.3594E-06	0.03851149	H3K4me1	LD
chr11	14399365	14399421	DEL	chr11_14456933_14457369	3.147742	10.019175	8.3676E-06	0.03851149	H3K4me1	LD
chr6	28764369	28764445	DEL	chr6_28932529_28933665	1.61622674	10.0209311	8.3567E-06	0.03851149	H3K4me1	LD

chr6	28774216	28774514	DEL	chr6_28932529_28933665	1.61622674	10.0209311	8.3567E-06	0.03851149	H3K4me1	LD
chr14	88636891	88636892	INS	chr14_88684836_88685370	1.62910076	10.0315428	8.2909E-06	0.03851149	H3K4me1	LD
chr3	11291733	11291734	INS	chr3_11431947_11432998	-1.7457429	-10.020493	8.3594E-06	0.03851149	H3K4me1	LD
chr3	11294221	11294222	INS	chr3_11431947_11432998	-1.7457429	-10.020493	8.3594E-06	0.03851149	H3K4me1	LD
chr3	11296129	11296130	INS	chr3_11431947_11432998	1.74574292	10.020493	8.3594E-06	0.03851149	H3K4me1	LD
chr3	11349372	11349373	INS	chr3_11431947_11432998	-1.7457429	-10.020493	8.3594E-06	0.03851149	H3K4me1	LD
chr3	11436934	11436935	INS	chr3_11431947_11432998	-1.7457429	-10.020493	8.3594E-06	0.03851149	H3K4me1	LD
chr8	71798333	71800854	DEL	chr8_71719599_71720047	1.34020785	9.99306283	8.5323E-06	0.03857286	H3K4me1	LD
chr13	205429659	205429709	DEL	chr13_205443796_205444421	0.95717631	10.0093398	8.4292E-06	0.03857286	H3K4me1	LD
chr13	205442803	205443212	DEL	chr13_205443796_205444421	0.95717631	10.0093398	8.4292E-06	0.03857286	H3K4me1	LD
chr12	14105502	14105645	DEL	chr12_14262813_14263232	1.54466111	9.99593651	8.514E-06	0.03857286	H3K4me1	LD
chr8	71766736	71766737	INS	chr8_71719599_71720047	1.34020785	9.99306283	8.5323E-06	0.03857286	H3K4me1	LD
chr8	71703616	71703617	INS	chr8_71719599_71720047	1.34020785	9.99306283	8.5323E-06	0.03857286	H3K4me1	LD

chr13	205298141	205298142	INS	chr13_205443796_205444421	0.95717631	10.0093398	8.4292E-06	0.03857286	H3K4me1	LD
chr16	6747193	6747194	INS	chr16_6724448_6724712	1.64917756	9.98831488	8.5626E-06	0.03857286	H3K4me1	LD
chr12	14107505	14107506	INS	chr12_14262813_14263232	1.54466111	9.99593651	8.514E-06	0.03857286	H3K4me1	LD
chr12	14410915	14410916	INS	chr12_14262813_14263232	1.54466111	9.99593651	8.514E-06	0.03857286	H3K4me1	LD
chr6	27203017	27203018	INS	chr6_27230692_27231934	1.5208619	10.0056935	8.4522E-06	0.03857286	H3K4me1	LD
chr5	72614918	72614982	DEL	chr5_72758856_72759390	1.69874261	9.92357384	8.9883E-06	0.03870218	H3K4me1	LD
chr3	86103630	86103932	DEL	chr3_86248717_86249044	-3.5450469	-9.8980418	9.1626E-06	0.03870218	H3K4me1	LD
chr12	6156017	6156192	DEL	chr12_6291592_6291928	2.09879722	9.94246332	8.8617E-06	0.03870218	H3K4me1	LD
chr14	133145647	133145877	DEL	chr14_133207607_133208234	1.42813756	9.90995916	9.0808E-06	0.03870218	H3K4me1	LD
chr14	133203448	133203517	DEL	chr14_133207607_133208234	2.85627511	9.90995916	9.0808E-06	0.03870218	H3K4me1	LD
chr14	133232275	133232392	DEL	chr14_133207607_133208234	1.42813756	9.90995916	9.0808E-06	0.03870218	H3K4me1	LD
chr14	133315198	133315368	DEL	chr14_133207607_133208234	1.42813756	9.90995916	9.0808E-06	0.03870218	H3K4me1	LD
chr9	137024426	137025185	DEL	chr9_137032349_137032765	2.3933925	9.95176313	8.8001E-06	0.03870218	H3K4me1	LD

chr1	203028304	203028480	DEL	chr1_203090936_203091812	0.89954461	9.95913491	8.7516E-06	0.03870218	H3K4me1	LD
chr5	17142665	17142986	DEL	chr5_17270905_17271883	-1.830682	-9.8940719	9.1901E-06	0.03870218	H3K4me1	LD
chr5	17200810	17201086	DEL	chr5_17270905_17271883	-1.830682	-9.8940719	9.1901E-06	0.03870218	H3K4me1	LD
chr5	17204281	17204579	DEL	chr5_17270905_17271883	-1.830682	-9.8940719	9.1901E-06	0.03870218	H3K4me1	LD
chr5	17232664	17232847	DEL	chr5_17270905_17271883	-1.830682	-9.8940719	9.1901E-06	0.03870218	H3K4me1	LD
chr5	17251521	17251804	DEL	chr5_17270905_17271883	-1.830682	-9.8940719	9.1901E-06	0.03870218	H3K4me1	LD
chr14	10773637	10773919	DEL	chr14_10694182_10695009	0.25923183	9.9311267	8.9374E-06	0.03870218	H3K4me1	LD
chr5	4826056	4826057	INS	chr5_4935252_4935563	2.41622183	9.90147532	9.139E-06	0.03870218	H3K4me1	LD
chr5	5034276	5034277	INS	chr5_4935252_4935563	2.41622183	9.90147532	9.139E-06	0.03870218	H3K4me1	LD
chr5	5108214	5108215	INS	chr5_4935252_4935563	2.41622183	9.90147532	9.139E-06	0.03870218	H3K4me1	LD
chr7	98000589	98000590	INS	chr7_97923702_97924143	2.27937681	9.89603949	9.1765E-06	0.03870218	H3K4me1	LD
chr14	133104665	133104666	INS	chr14_133207607_133208234	1.42813756	9.90995916	9.0808E-06	0.03870218	H3K4me1	LD
chr14	133202898	133202899	INS	chr14_133207607_133208234	1.42813756	9.90995916	9.0808E-06	0.03870218	H3K4me1	LD

chr14	133285066	133285067	INS	chr14_133207607_133208234	1.42813756	9.90995916	9.0808E-06	0.03870218	H3K4me1	LD
chr1	202967698	202967699	INS	chr1_203090936_203091812	0.89954461	9.95913491	8.7516E-06	0.03870218	H3K4me1	LD
chr9	7901393	7901394	INS	chr9_7789110_7790613	1.12069238	9.89889445	9.1568E-06	0.03870218	H3K4me1	LD
chr8	15493133	15493134	INS	chr8_15615624_15615984	1.79389178	9.9193877	9.0166E-06	0.03870218	H3K4me1	LD
chr8	15597660	15597661	INS	chr8_15615624_15615984	1.79389178	9.9193877	9.0166E-06	0.03870218	H3K4me1	LD
chr5	17224329	17224330	INS	chr5_17270905_17271883	-1.830682	-9.8940719	9.1901E-06	0.03870218	H3K4me1	LD
chr5	17252206	17252207	INS	chr5_17270905_17271883	-1.830682	-9.8940719	9.1901E-06	0.03870218	H3K4me1	LD
chr14	10550131	10550132	INS	chr14_10694182_10695009	0.25923183	9.9311267	8.9374E-06	0.03870218	H3K4me1	LD
chr14	10572445	10572446	INS	chr14_10694182_10695009	0.25923183	9.9311267	8.9374E-06	0.03870218	H3K4me1	LD
chr14	10655238	10655239	INS	chr14_10694182_10695009	0.25923183	9.9311267	8.9374E-06	0.03870218	H3K4me1	LD
chr14	10660859	10660860	INS	chr14_10694182_10695009	0.25923183	9.9311267	8.9374E-06	0.03870218	H3K4me1	LD
chr3	21513737	21513738	INS	chr3_21513115_21513392	1.92388269	9.89764446	9.1654E-06	0.03870218	H3K4me1	LD
chr16	47173433	47173434	INS	chr16_47185739_47187138	-0.7096326	-9.8968943	9.1706E-06	0.03870218	H3K4me1	LD

chr16	47363536	47363537	INS	chr16_47185739_47187138	-0.7096326	-9.8968943	9.1706E-06	0.03870218	H3K4me1	LD
chr17	25249608	25249609	INS	chr17_25229215_25231128	1.27875322	9.98042463	8.6133E-06	0.03870218	H3K4me1	LD
chr17	38326916	38327205	DEL	chr17_38318836_38319623	-3.6123302	-9.887043	9.2389E-06	0.03879786	H3K4me1	LD
chr17	38326847	38326848	INS	chr17_38318836_38319623	-3.6123302	-9.887043	9.2389E-06	0.03879786	H3K4me1	LD
chr10	45499810	45499811	INS	chr10_45400242_45401868	1.58890067	9.86213653	9.4142E-06	0.03947837	H3K4me1	LD
chr6	104426384	104426586	DEL	chr6_104434728_104435983	1.29257639	9.85149887	9.4903E-06	0.03957387	H3K4me1	LD
chr6	104421064	104421065	INS	chr6_104434728_104435983	1.29257639	9.85149887	9.4903E-06	0.03957387	H3K4me1	LD
chr6	104417617	104417618	INS	chr6_104434728_104435983	1.29257639	9.85149887	9.4903E-06	0.03957387	H3K4me1	LD
chr6	104527556	104527557	INS	chr6_104434728_104435983	1.29257639	9.85149887	9.4903E-06	0.03957387	H3K4me1	LD
chr1	129371173	129371223	DEL	chr1_129354352_129355233	1.26768883	9.8448545	9.5381E-06	0.03969338	H3K4me1	LD
chr14	138982100	138982101	INS	chr14_139085186_139086136	0.62150317	9.84381074	9.5456E-06	0.03969338	H3K4me1	LD
chr1	115293954	115294242	DEL	chr1_115168930_115170102	1.27963071	9.84116367	9.5648E-06	0.03971745	H3K4me1	LD
chr8	93985389	93992791	DEL	chr8_94055021_94056101	1.31138907	9.83140589	9.6357E-06	0.03984509	H3K4me1	LD

chr12	10666077	10666078	INS	chr12_10585791_10586090	1.73927174	9.83358937	9.6198E-06	0.03984509	H3K4me1	LD
chr1	21122825	21122826	INS	chr1_21000677_21001125	0.781824	9.83321791	9.6225E-06	0.03984509	H3K4me1	LD
chr1	12226999	12227273	DEL	chr1_12389210_12390056	1.98142948	9.82882656	9.6546E-06	0.0398676	H3K4me1	LD
chr13	182944973	182945094	DEL	chr13_182815086_182815452	1.85990368	9.80452903	9.8342E-06	0.04032924	H3K4me1	LD
chr13	183001493	183003696	DEL	chr13_182815086_182815452	1.85990368	9.80452903	9.8342E-06	0.04032924	H3K4me1	LD
NW_018085100.1	119755	119948	DEL	NW_018085100.1_118934_119372	1.22233167	9.80898611	9.8009E-06	0.04032924	H3K4me1	LD
chr8	41416313	41416314	INS	chr8_41545507_41547375	2.35998438	9.80919476	9.7994E-06	0.04032924	H3K4me1	LD
chr1	248192803	248194778	DEL	chr1_248235894_248237756	-1.707275	-9.7883361	9.9559E-06	0.04054897	H3K4me1	LD
chr3	33375700	33377288	DEL	chr3_33313917_33314650	0.99491983	9.79018371	9.942E-06	0.04054897	H3K4me1	LD
chr3	33374425	33375258	DEL	chr3_33313917_33314650	0.99491983	9.79018371	9.942E-06	0.04054897	H3K4me1	LD
chr3	33378973	33379109	DEL	chr3_33313917_33314650	0.99491983	9.79018371	9.942E-06	0.04054897	H3K4me1	LD
chr1	248238996	248238997	INS	chr1_248235894_248237756	-1.707275	-9.7883361	9.9559E-06	0.04054897	H3K4me1	LD
chr3	109306300	109308118	DEL	chr3_109136388_109136887	3.833798	46.1674428	5.3539E-11	3.6663E-05	H3K4me1	LD

chr6	27274566	27274567	INS	chr6_27209199_27209863	3.77917006	45.3572125	6.1657E-11	3.6663E-05	H3K4me1	LD
chr2	1774590	1775895	DEL	chr2_1935172_1936585	9.32618656	34.1479153	5.9104E-10	0.00019302	H3K4me3	LD
chr6	27072003	27072310	DEL	chr6_27508806_27509961	5.98760311	30.5118161	1.4458E-09	0.00019302	H3K4me3	LD
chr6	27156306	27156375	DEL	chr6_27508806_27509961	5.98760311	30.5118161	1.4458E-09	0.00019302	H3K4me3	LD
chr6	27614341	27614930	DEL	chr6_27508806_27509961	5.98760311	30.5118161	1.4458E-09	0.00019302	H3K4me3	LD
chr6	27671069	27671242	DEL	chr6_27508806_27509961	5.98760311	30.5118161	1.4458E-09	0.00019302	H3K4me3	LD
chr6	27821965	27822250	DEL	chr6_27508806_27509961	5.98760311	30.5118161	1.4458E-09	0.00019302	H3K4me3	LD
chr2	1729822	1729823	INS	chr2_1484061_1484352	15.4433808	31.4246309	1.144E-09	0.00019302	H3K4me3	LD
chr6	27499402	27499403	INS	chr6_27508806_27509961	5.98760311	30.5118161	1.4458E-09	0.00019302	H3K4me3	LD
chr2	1729822	1729823	INS	chr2_1482968_1483769	12.7412767	28.8210031	2.2728E-09	0.00028559	H3K4me3	LD
chr2	1729822	1729823	INS	chr2_1479124_1480952	19.9019393	28.4979694	2.4853E-09	0.00029494	H3K4me3	LD
chr14	115562345	115562543	DEL	chr14_115187519_115188851	9.31244833	24.4900921	8.2523E-09	0.00082151	H3K4me3	LD
chr14	115142175	115142176	INS	chr14_115187519_115188851	9.31244833	24.4900921	8.2523E-09	0.00082151	H3K4me3	LD

chr14	115398866	115398867	INS	chr14_115187519_115188851	9.31244833	24.4900921	8.2523E-09	0.00082151	H3K4me3	LD
chr6	42800274	42800275	INS	chr6_42802508_42803417	1.95218219	24.4129308	8.4608E-09	0.00082151	H3K4me3	LD
chr17	54592988	54593171	DEL	chr17_55018403_55019159	0.99713563	23.1107878	1.3045E-08	0.00093992	H3K4me3	LD
NW_018085246.1	75482	75532	DEL	NW_018085246.1_140783_142911	-14.509557	-21.976963	1.9399E-08	0.00093992	H3K4me3	LD
NW_018085246.1	224079	224366	DEL	NW_018085246.1_140783_142911	-14.509557	-21.976963	1.9399E-08	0.00093992	H3K4me3	LD
NW_018085246.1	294074	294137	DEL	NW_018085246.1_140783_142911	-14.509557	-21.976963	1.9399E-08	0.00093992	H3K4me3	LD
NW_018085246.1	328807	329087	DEL	NW_018085246.1_140783_142911	-14.509557	-21.976963	1.9399E-08	0.00093992	H3K4me3	LD
NW_018085246.1	357496	357556	DEL	NW_018085246.1_140783_142911	-14.509557	-21.976963	1.9399E-08	0.00093992	H3K4me3	LD
chr8	12408939	12409025	DEL	chr8_12667139_12669495	8.99481111	21.6288686	2.2001E-08	0.00093992	H3K4me3	LD
chr8	12774810	12775117	DEL	chr8_12667139_12669495	8.99481111	21.6288686	2.2001E-08	0.00093992	H3K4me3	LD
chr8	12777088	12777364	DEL	chr8_12667139_12669495	8.99481111	21.6288686	2.2001E-08	0.00093992	H3K4me3	LD
chr8	12793713	12793868	DEL	chr8_12667139_12669495	8.99481111	21.6288686	2.2001E-08	0.00093992	H3K4me3	LD
chr3	3665671	3665672	INS	chr3_4153523_4154780	2.1939706	22.9582018	1.3745E-08	0.00093992	H3K4me3	LD

chr6	80308875	80308876	INS	chr6_80467930_80468911	3.04381888	21.9649922	1.9483E-08	0.00093992	H3K4me3	LD
chr6	80313815	80313816	INS	chr6_80467930_80468911	3.04381888	21.9649922	1.9483E-08	0.00093992	H3K4me3	LD
chr3	3924276	3924277	INS	chr3_4153523_4154780	2.1939706	22.9582018	1.3745E-08	0.00093992	H3K4me3	LD
NW_018085246.1	305762	305763	INS	NW_018085246.1_140783_142911	-14.509557	-21.976963	1.9399E-08	0.00093992	H3K4me3	LD
NW_018085246.1	356177	356178	INS	NW_018085246.1_140783_142911	-14.509557	-21.976963	1.9399E-08	0.00093992	H3K4me3	LD
NW_018085246.1	351928	351929	INS	NW_018085246.1_140783_142911	-14.509557	-21.976963	1.9399E-08	0.00093992	H3K4me3	LD
chr9	7913352	7913353	INS	chr9_8386819_8387517	16.0768619	22.464193	1.6319E-08	0.00093992	H3K4me3	LD
chr8	12793025	12793026	INS	chr8_12667139_12669495	8.99481111	21.6288686	2.2001E-08	0.00093992	H3K4me3	LD
chr8	12795850	12795851	INS	chr8_12667139_12669495	8.99481111	21.6288686	2.2001E-08	0.00093992	H3K4me3	LD
chr8	12985884	12985885	INS	chr8_12667139_12669495	8.99481111	21.6288686	2.2001E-08	0.00093992	H3K4me3	LD
chr11	25436047	25436048	INS	chr11_25705439_25707227	-19.2356	-23.115	1.3027E-08	0.00093992	H3K4me3	LD
NW_018085211.1	29713	29714	INS	chr11_25705439_25707227	-19.2356	-23.115	1.3027E-08	0.00093992	H3K4me3	LD
chr12	55169082	55170053	DEL	chr12_55494112_55494635	1.99608744	21.0090545	2.7662E-08	0.00095304	H3K4me3	LD

chr12	55616959	55617051	DEL	chr12_55494112_55494635	1.99608744	21.0090545	2.7662E-08	0.00095304	H3K4me3	LD
chr5	88338289	88338498	DEL	chr5_88433616_88434442	2.25401867	21.0250279	2.7497E-08	0.00095304	H3K4me3	LD
chr11	20834491	20834677	DEL	chr11_20766421_20767759	4.93254778	21.1412934	2.6328E-08	0.00095304	H3K4me3	LD
chr11	20890182	20890256	DEL	chr11_20766421_20767759	4.93254778	21.1412934	2.6328E-08	0.00095304	H3K4me3	LD
chr12	55609745	55609746	INS	chr12_55494112_55494635	1.99608744	21.0090545	2.7662E-08	0.00095304	H3K4me3	LD
chr12	55832663	55832664	INS	chr12_55494112_55494635	1.99608744	21.0090545	2.7662E-08	0.00095304	H3K4me3	LD
chr11	20793186	20793187	INS	chr11_20766421_20767759	4.93254778	21.1412934	2.6328E-08	0.00095304	H3K4me3	LD
chr11	20806124	20806125	INS	chr11_20766421_20767759	4.93254778	21.1412934	2.6328E-08	0.00095304	H3K4me3	LD
chr11	20980379	20980380	INS	chr11_20766421_20767759	4.93254778	21.1412934	2.6328E-08	0.00095304	H3K4me3	LD
chr11	21120982	21120983	INS	chr11_20766421_20767759	4.93254778	21.1412934	2.6328E-08	0.00095304	H3K4me3	LD
chr13	8980723	8982396	DEL	chr13_8932616_8933134	2.31583756	20.4111866	3.4716E-08	0.00114089	H3K4me3	LD
chr13	9316777	9316778	INS	chr13_8932616_8933134	2.31583756	20.4111866	3.4716E-08	0.00114089	H3K4me3	LD
chr13	9401648	9401649	INS	chr13_8932616_8933134	2.31583756	20.4111866	3.4716E-08	0.00114089	H3K4me3	LD

chr1	6020780	6020853	DEL	chr1_6414266_6414955	2.81381212	20.0507459	3.9936E-08	0.00127325	H3K4me3	LD
chr1	142151990	142152063	DEL	chr1_142412344_142413972	-26.047844	-19.828773	4.3587E-08	0.00134937	H3K4me3	LD
chr17	49127291	49127292	INS	chr17_49015303_49015622	2.35993528	19.8388902	4.3412E-08	0.00134937	H3K4me3	LD
chr11	2929403	2929403	BND	chr11_3038646_3039770	3.38633755	19.321842	5.3416E-08	0.00143921	H3K4me3	LD
chr11	2796284	2796586	DEL	chr11_3038646_3039770	3.38633755	19.321842	5.3416E-08	0.00143921	H3K4me3	LD
chr11	3028105	3028695	DEL	chr11_3038646_3039770	3.38633755	19.321842	5.3416E-08	0.00143921	H3K4me3	LD
chr11	3465357	3469944	DEL	chr11_3038646_3039770	3.38633755	19.321842	5.3416E-08	0.00143921	H3K4me3	LD
chr3	126594383	126594671	DEL	chr3_126704427_126706065	5.43487694	19.2390494	5.5247E-08	0.00143921	H3K4me3	LD
chr3	126980653	126980722	DEL	chr3_126704427_126706065	5.43487694	19.2390494	5.5247E-08	0.00143921	H3K4me3	LD
chr11	2929402	2929403	INS	chr11_3038646_3039770	3.38633755	19.321842	5.3416E-08	0.00143921	H3K4me3	LD
chr7	95393408	95393409	INS	chr7_94941646_94942609	2.85888861	19.5453279	4.8805E-08	0.00143921	H3K4me3	LD
chr3	126224286	126224287	INS	chr3_126704427_126706065	5.43487694	19.2390494	5.5247E-08	0.00143921	H3K4me3	LD
chr3	126371813	126371814	INS	chr3_126704427_126706065	5.43487694	19.2390494	5.5247E-08	0.00143921	H3K4me3	LD

chr3	126522554	126522555	INS	chr3_126704427_126706065	5.43487694	19.2390494	5.5247E-08	0.00143921	H3K4me3	LD
chr3	127165473	127165474	INS	chr3_126704427_126706065	5.43487694	19.2390494	5.5247E-08	0.00143921	H3K4me3	LD
chr8	41223208	41783661	DUP	chr8_41401866_41403769	23.6634665	14.2467895	5.7535E-08	0.00078348	H3K4me3	LD
chr5	66369181	66369379	DEL	chr5_66662359_66662593	1.89426033	18.964264	6.1848E-08	0.0015632	H3K4me3	LD
chr11	4246983	4247422	DEL	chr11_4021939_4023418	10.6660864	18.9039889	6.3412E-08	0.0015632	H3K4me3	LD
chr12	55911158	55911159	INS	chr12_55477555_55478426	3.71429768	18.9288418	6.2762E-08	0.0015632	H3K4me3	LD
chr11	66710966	66710967	INS	chr11_67205398_67206830	3.73645733	18.8128369	6.5861E-08	0.0015632	H3K4me3	LD
chr11	66880425	66880426	INS	chr11_67205398_67206830	3.73645733	18.8128369	6.5861E-08	0.0015632	H3K4me3	LD
chr11	67148004	67148005	INS	chr11_67205398_67206830	3.73645733	18.8128369	6.5861E-08	0.0015632	H3K4me3	LD
chr11	67215377	67215378	INS	chr11_67205398_67206830	3.73645733	18.8128369	6.5861E-08	0.0015632	H3K4me3	LD
chr11	67652297	67652298	INS	chr11_67205398_67206830	3.73645733	18.8128369	6.5861E-08	0.0015632	H3K4me3	LD
chr1	6460049	6460050	INS	chr1_6395990_6397583	1.87106773	18.7340054	6.8066E-08	0.0015804	H3K4me3	LD
chr1	6643056	6643057	INS	chr1_6395990_6397583	1.87106773	18.7340054	6.8066E-08	0.0015804	H3K4me3	LD

chr13	31599528	31599529	INS	chr13_31916315_31917993	-13.7623	-18.584778	7.2469E-08	0.00166455	H3K4me3	LD
chr18	20667030	20667031	INS	chr18_20664654_20665040	2.10753833	17.9898224	9.3501E-08	0.00210243	H3K4me3	LD
chr10	47662570	47662644	DEL	chr10_47611286_47612241	2.46602863	17.8374098	9.9938E-08	0.00216352	H3K4me3	LD
chr12	13553928	13554224	DEL	chr12_13514758_13515771	2.16654594	17.7619403	1.0331E-07	0.00216352	H3K4me3	LD
chr15	133027548	133027549	INS	chr15_132761945_132763229	1.78317725	17.8209245	1.0066E-07	0.00216352	H3K4me3	LD
chr12	13566024	13566025	INS	chr12_13514758_13515771	2.16654594	17.7619403	1.0331E-07	0.00216352	H3K4me3	LD
chr5	100885004	100885004	BND	chr5_101254208_101255160	3.13977413	17.7017441	1.0609E-07	0.00220017	H3K4me3	LD
chr1	252539664	252539951	DEL	chr1_252943350_252944059	3.03121669	17.6345749	1.0929E-07	0.00224477	H3K4me3	LD
chr9	33802484	33802485	INS	chr9_34094643_34095402	2.70312483	17.6033587	1.1081E-07	0.0022544	H3K4me3	LD
chr15	25462007	25462315	DEL	chr15_25301210_25301736	4.82187678	17.3790508	1.225E-07	0.00230248	H3K4me3	LD
chr1	131675549	131675550	INS	chr1_132161477_132162532	4.31839106	17.4837505	1.1688E-07	0.00230248	H3K4me3	LD
chr17	46495345	46495346	INS	chr17_46622025_46622303	3.88132819	17.2332894	1.3083E-07	0.0023289	H3K4me3	LD
chr3	53936080	53936081	INS	chr3_53632549_53633181	1.80950183	17.1112404	1.383E-07	0.00242147	H3K4me3	LD

chr3	54034033	54034034	INS	chr3_53632549_53633181	1.80950183	17.1112404	1.383E-07	0.00242147	H3K4me3	LD
chr1	268489011	268489093	DEL	chr1_268689508_268692057	1.75663688	16.4947431	1.8413E-07	0.00300266	H3K4me3	LD
chr1	268975962	268976235	DEL	chr1_268689508_268692057	1.75663688	16.4947431	1.8413E-07	0.00300266	H3K4me3	LD
chr5	19085361	19085671	DEL	chr5_19450626_19451909	5.61968438	16.4001476	1.9257E-07	0.00300266	H3K4me3	LD
chr12	26953276	26953559	DEL	chr12_26823466_26824079	2.76043478	16.403446	1.9227E-07	0.00300266	H3K4me3	LD
chr12	27037542	27037834	DEL	chr12_26823466_26824079	2.76043478	16.403446	1.9227E-07	0.00300266	H3K4me3	LD
chr1	71487847	71487848	INS	chr1_71487839_71488572	4.42525389	16.4236739	1.9044E-07	0.00300266	H3K4me3	LD
chr5	19616645	19616646	INS	chr5_19450626_19451909	5.61968438	16.4001476	1.9257E-07	0.00300266	H3K4me3	LD
chr12	26368385	26368386	INS	chr12_26823466_26824079	2.76043478	16.403446	1.9227E-07	0.00300266	H3K4me3	LD
chr12	27009586	27009587	INS	chr12_26823466_26824079	2.76043478	16.403446	1.9227E-07	0.00300266	H3K4me3	LD
chr12	27012031	27012032	INS	chr12_26823466_26824079	2.76043478	16.403446	1.9227E-07	0.00300266	H3K4me3	LD
chr12	27207300	27207301	INS	chr12_26823466_26824079	2.76043478	16.403446	1.9227E-07	0.00300266	H3K4me3	LD
chr14	111014067	111062178	DUP	chr14_111047934_111048601	4.25183511	16.2379099	2.0808E-07	0.00317266	H3K4me3	LD

chr14	111449975	111449976	INS	chr14_111047934_111048601	2.12591756	16.2379099	2.0808E-07	0.00317266	H3K4me3	LD
chr1	8139905	8139906	INS	chr1_8060666_8061043	2.73617973	16.2245573	2.0942E-07	0.00317266	H3K4me3	LD
chr1	8382109	8382110	INS	chr1_8060666_8061043	2.73617973	16.2245573	2.0942E-07	0.00317266	H3K4me3	LD
chr3	68144528	68144998	DEL	chr3_68362355_68363621	1.31868856	16.1634073	2.1567E-07	0.00322164	H3K4me3	LD
chr3	68141059	68142597	INV	chr3_68362355_68363621	2.63737713	16.1634073	2.1567E-07	0.00322164	H3K4me3	LD
chr2	144634483	144634613	DEL	chr2_144610839_144611690	4.91528472	16.0607411	2.2664E-07	0.00329355	H3K4me3	LD
chr13	204597061	204597984	DEL	chr13_205091716_205092583	2.84492844	16.0164576	2.3156E-07	0.00329355	H3K4me3	LD
chr13	205241555	205241612	DEL	chr13_205091716_205092583	2.84492844	16.0164576	2.3156E-07	0.00329355	H3K4me3	LD
chr8	1497631	1497737	DEL	chr8_1379152_1379713	3.56669072	16.0053091	2.3282E-07	0.00329355	H3K4me3	LD
chr13	204893478	204893479	INS	chr13_205091716_205092583	2.84492844	16.0164576	2.3156E-07	0.00329355	H3K4me3	LD
chr13	205318354	205318355	INS	chr13_205091716_205092583	2.84492844	16.0164576	2.3156E-07	0.00329355	H3K4me3	LD
chr13	205483287	205483288	INS	chr13_205091716_205092583	2.84492844	16.0164576	2.3156E-07	0.00329355	H3K4me3	LD
chr8	1500407	1500408	INS	chr8_1379152_1379713	3.56669072	16.0053091	2.3282E-07	0.00329355	H3K4me3	LD

chr11	67775143	67775447	DEL	chr11_67947319_67949849	-3.814707	-15.643759	2.7809E-07	0.00330019	H3K4me3	LD
chr3	105682570	105682767	DEL	chr3_106154425_106154625	2.44549356	15.8250044	2.5427E-07	0.00330019	H3K4me3	LD
chr4	117978801	117978916	DEL	chr4_117731062_117732865	3.28164276	15.646426	2.7772E-07	0.00330019	H3K4me3	LD
chr4	118001729	118001880	DEL	chr4_117731062_117732865	3.28164276	15.646426	2.7772E-07	0.00330019	H3K4me3	LD
chr4	117999909	118000198	DEL	chr4_117731062_117732865	3.28164276	15.646426	2.7772E-07	0.00330019	H3K4me3	LD
chr13	204514042	204514128	DEL	chr13_204105579_204106477	2.08442261	15.826465	2.5409E-07	0.00330019	H3K4me3	LD
chr13	204597061	204597984	DEL	chr13_204105579_204106477	2.08442261	15.826465	2.5409E-07	0.00330019	H3K4me3	LD
chr11	9632489	9632564	DEL	chr11_9634632_9635174	2.34227306	15.847233	2.5151E-07	0.00330019	H3K4me3	LD
chr11	67909408	67909409	INS	chr11_67947319_67949849	-3.814707	-15.643759	2.7809E-07	0.00330019	H3K4me3	LD
chr11	68004883	68004884	INS	chr11_67947319_67949849	-3.814707	-15.643759	2.7809E-07	0.00330019	H3K4me3	LD
chr3	105713637	105713638	INS	chr3_106154425_106154625	2.44549356	15.8250044	2.5427E-07	0.00330019	H3K4me3	LD
chr3	106398685	106398686	INS	chr3_106154425_106154625	2.44549356	15.8250044	2.5427E-07	0.00330019	H3K4me3	LD
chr4	117473079	117473080	INS	chr4_117731062_117732865	3.28164276	15.646426	2.7772E-07	0.00330019	H3K4me3	LD

chr4	117746392	117746393	INS	chr4_117731062_117732865	3.28164276	15.646426	2.7772E-07	0.00330019	H3K4me3	LD
chr4	117945046	117945047	INS	chr4_117731062_117732865	3.28164276	15.646426	2.7772E-07	0.00330019	H3K4me3	LD
chr4	118002624	118002625	INS	chr4_117731062_117732865	3.28164276	15.646426	2.7772E-07	0.00330019	H3K4me3	LD
chr4	118005675	118005676	INS	chr4_117731062_117732865	3.28164276	15.646426	2.7772E-07	0.00330019	H3K4me3	LD
chr4	118083331	118083332	INS	chr4_117731062_117732865	3.28164276	15.646426	2.7772E-07	0.00330019	H3K4me3	LD
chr4	118131010	118131011	INS	chr4_117731062_117732865	3.28164276	15.646426	2.7772E-07	0.00330019	H3K4me3	LD
chr11	9141194	9141195	INS	chr11_9634632_9635174	2.34227306	15.847233	2.5151E-07	0.00330019	H3K4me3	LD
chr11	9212236	9212237	INS	chr11_9634632_9635174	2.34227306	15.847233	2.5151E-07	0.00330019	H3K4me3	LD
chr11	9256939	9256940	INS	chr11_9634632_9635174	2.34227306	15.847233	2.5151E-07	0.00330019	H3K4me3	LD
chr11	9634289	9634290	INS	chr11_9634632_9635174	2.34227306	15.847233	2.5151E-07	0.00330019	H3K4me3	LD
chr11	9669823	9669824	INS	chr11_9634632_9635174	2.34227306	15.847233	2.5151E-07	0.00330019	H3K4me3	LD
chr11	9940233	9940234	INS	chr11_9634632_9635174	2.34227306	15.847233	2.5151E-07	0.00330019	H3K4me3	LD
chr11	9938346	9938347	INS	chr11_9634632_9635174	2.34227306	15.847233	2.5151E-07	0.00330019	H3K4me3	LD

chr4	37860289	37860290	INS	chr4_37528775_37529510	2.18836701	15.8054512	2.5672E-07	0.00330019	H3K4me3	LD
chr4	110940984	110944411	DEL	chr4_111228529_111229549	2.33888388	15.4527754	3.0594E-07	0.00342156	H3K4me3	LD
chr4	111126400	111126653	DEL	chr4_111228529_111229549	2.33888388	15.4527754	3.0594E-07	0.00342156	H3K4me3	LD
chr4	111288062	111288371	DEL	chr4_111228529_111229549	2.33888388	15.4527754	3.0594E-07	0.00342156	H3K4me3	LD
chr4	111483287	111484159	DEL	chr4_111228529_111229549	2.33888388	15.4527754	3.0594E-07	0.00342156	H3K4me3	LD
chr14	112232488	112232489	INS	chr14_112053406_112054761	7.5865	15.5170776	2.9623E-07	0.00342156	H3K4me3	LD
chr4	110797388	110797389	INS	chr4_111228529_111229549	2.33888388	15.4527754	3.0594E-07	0.00342156	H3K4me3	LD
chr4	111005051	111005052	INS	chr4_111228529_111229549	2.33888388	15.4527754	3.0594E-07	0.00342156	H3K4me3	LD
chr4	111235724	111235725	INS	chr4_111228529_111229549	2.33888388	15.4527754	3.0594E-07	0.00342156	H3K4me3	LD
chr4	111287234	111287235	INS	chr4_111228529_111229549	2.33888388	15.4527754	3.0594E-07	0.00342156	H3K4me3	LD
chr4	111668628	111668629	INS	chr4_111228529_111229549	2.33888388	15.4527754	3.0594E-07	0.00342156	H3K4me3	LD
chr4	111688795	111688796	INS	chr4_111228529_111229549	2.33888388	15.4527754	3.0594E-07	0.00342156	H3K4me3	LD
chr5	73979409	73979468	DEL	chr5_73931974_73932610	1.18769725	15.2556411	3.38E-07	0.00361008	H3K4me3	LD

chr3	12997649	12997888	DEL	chr3_13007414_13008056	3.53650228	15.3047368	3.2968E-07	0.00361008	H3K4me3	LD
chr8	47128072	47128679	DEL	chr8_47468417_47469347	2.51859161	15.2748475	3.3472E-07	0.00361008	H3K4me3	LD
chr8	47452086	47452265	DEL	chr8_47468417_47469347	2.51859161	15.2748475	3.3472E-07	0.00361008	H3K4me3	LD
chr3	12956131	12956132	INS	chr3_13225162_13225846	1.75995126	15.2929997	3.3165E-07	0.00361008	H3K4me3	LD
chr3	13027216	13027217	INS	chr3_13007414_13008056	3.53650228	15.3047368	3.2968E-07	0.00361008	H3K4me3	LD
chr3	13085123	13085124	INS	chr3_13225162_13225846	1.75995126	15.2929997	3.3165E-07	0.00361008	H3K4me3	LD
chr1	18325395	18325396	INS	chr1_17853053_17853892	1.238495	15.2633752	3.3668E-07	0.00361008	H3K4me3	LD
chr8	47460588	47460589	INS	chr8_47468417_47469347	2.51859161	15.2748475	3.3472E-07	0.00361008	H3K4me3	LD
chr8	71578731	71578975	DEL	chr8_71193165_71193530	2.37141222	15.1850028	3.504E-07	0.00363345	H3K4me3	LD
chr8	70929158	70929159	INS	chr8_71193165_71193530	2.37141222	15.1850028	3.504E-07	0.00363345	H3K4me3	LD
chr8	71515478	71515479	INS	chr8_71193165_71193530	2.37141222	15.1850028	3.504E-07	0.00363345	H3K4me3	LD
chr11	3527785	3527786	INS	chr11_3616531_3618144	2.7173773	15.2025991	3.4726E-07	0.00363345	H3K4me3	LD
chr11	3613718	3613719	INS	chr11_3616531_3618144	2.7173773	15.2025991	3.4726E-07	0.00363345	H3K4me3	LD

chr7	88720496	88720497	INS	chr7_89011093_89011413	3.00917978	15.1980001	3.4808E-07	0.00363345	H3K4me3	LD
chr11	17440582	17440907	DEL	chr11_17082386_17083288	2.54280256	15.1148713	3.6321E-07	0.00374811	H3K4me3	LD
chr2	58199810	58199811	INS	chr2_58461096_58461263	2.97311375	15.0074765	3.8386E-07	0.00394215	H3K4me3	LD
chr17	21515237	21515556	DEL	chr17_21872839_21873735	2.20758762	14.9015747	4.0552E-07	0.00406683	H3K4me3	LD
chr17	21448572	21448573	INS	chr17_21872839_21873735	2.20758762	14.9015747	4.0552E-07	0.00406683	H3K4me3	LD
chr17	21556027	21556028	INS	chr17_21872839_21873735	2.20758762	14.9015747	4.0552E-07	0.00406683	H3K4me3	LD
chr17	21548699	21548700	INS	chr17_21872839_21873735	2.20758762	14.9015747	4.0552E-07	0.00406683	H3K4me3	LD
chr17	22195850	22195851	INS	chr17_21872839_21873735	2.20758762	14.9015747	4.0552E-07	0.00406683	H3K4me3	LD
chr5	17802326	17802327	INS	chr5_17537536_17538500	2.97811563	14.7984233	4.2794E-07	0.00427164	H3K4me3	LD
chr17	55375184	55375313	DEL	chr17_55506119_55506802	1.92834928	14.5674516	4.8337E-07	0.00473637	H3K4me3	LD
chr17	55836482	55836483	INS	chr17_55506119_55506802	1.92834928	14.5674516	4.8337E-07	0.00473637	H3K4me3	LD
chr17	55973153	55973154	INS	chr17_55506119_55506802	1.92834928	14.5674516	4.8337E-07	0.00473637	H3K4me3	LD
chr6	134939853	134939854	INS	chr6_135162040_135162902	2.58836344	14.5675099	4.8335E-07	0.00473637	H3K4me3	LD

chr7	53911834	53912138	DEL	chr7_53664965_53667873	17.0683656	14.5074116	4.9906E-07	0.00473803	H3K4me3	LD
chr2	24460194	24460474	DEL	chr2_24956613_24957128	1.58299429	14.5081679	4.9886E-07	0.00473803	H3K4me3	LD
chr7	53994934	53994935	INS	chr7_53664965_53667873	17.0683656	14.5074116	4.9906E-07	0.00473803	H3K4me3	LD
chr14	117074443	117074444	INS	chr14_117517610_117518127	2.51363031	14.5259611	4.9415E-07	0.00473803	H3K4me3	LD
chr2	24871327	24871328	INS	chr2_24956613_24957128	1.58299429	14.5081679	4.9886E-07	0.00473803	H3K4me3	LD
chr2	25298951	25298952	INS	chr2_24956613_24957128	1.58299429	14.5081679	4.9886E-07	0.00473803	H3K4me3	LD
chr7	3035972	3035973	INS	chr7_2633298_2633778	1.36979369	14.3737522	5.3609E-07	0.0050671	H3K4me3	LD
chr6	50020156	50020347	DEL	chr6_49926247_49927164	7.69226389	14.1794105	5.9556E-07	0.00555546	H3K4me3	LD
chr6	49635255	49635256	INS	chr6_49926247_49927164	7.69226389	14.1794105	5.9556E-07	0.00555546	H3K4me3	LD
chr2	30364157	30364158	INS	chr2_30172788_30173420	2.80973334	14.1113044	6.1813E-07	0.00574085	H3K4me3	LD
chr2	130926753	130926754	INS	chr2_131117870_131119340	-7.7728175	-14.098829	6.2236E-07	0.00575517	H3K4me3	LD
chr14	107411096	107411097	INS	chr14_107179603_107180060	2.09314645	14.0592714	6.3601E-07	0.00585603	H3K4me3	LD
chr1	235761892	235762268	DEL	chr1_235834587_235836017	-10.637151	-13.958257	6.7241E-07	0.00608623	H3K4me3	LD

chr1	236168236	236169024	DEL	chr1_235834587_235836017	-21.274302	-13.958257	6.7241E-07	0.00608623	H3K4me3	LD
chr1	235828159	235828160	INS	chr1_235834587_235836017	-10.637151	-13.958257	6.7241E-07	0.00608623	H3K4me3	LD
chr1	236173642	236173643	INS	chr1_235834587_235836017	-10.637151	-13.958257	6.7241E-07	0.00608623	H3K4me3	LD
chr16	42587498	42587661	DEL	chr16_43060477_43060886	2.51324896	13.8375283	7.1901E-07	0.00637299	H3K4me3	LD
chr5	52398267	52398268	INS	chr5_52378547_52378911	2.26524938	13.8499557	7.1405E-07	0.00637299	H3K4me3	LD
chr16	42582967	42582968	INS	chr16_43060477_43060886	2.51324896	13.8375283	7.1901E-07	0.00637299	H3K4me3	LD
chr16	42589034	42589035	INS	chr16_43060477_43060886	2.51324896	13.8375283	7.1901E-07	0.00637299	H3K4me3	LD
chr16	43379935	43379936	INS	chr16_43060477_43060886	2.51324896	13.8375283	7.1901E-07	0.00637299	H3K4me3	LD
chr11	17440582	17440907	DEL	chr11_17039419_17039686	4.73095972	13.7712505	7.4612E-07	0.00658599	H3K4me3	LD
chr5	85274611	85274727	DEL	chr5_85108451_85110049	4.09467575	13.6777964	7.8632E-07	0.0067457	H3K4me3	LD
chr5	85296090	85296218	DEL	chr5_85108451_85110049	4.09467575	13.6777964	7.8632E-07	0.0067457	H3K4me3	LD
chr5	84827328	84827329	INS	chr5_85108451_85110049	4.09467575	13.6777964	7.8632E-07	0.0067457	H3K4me3	LD
chr5	85207330	85207331	INS	chr5_85108451_85110049	4.09467575	13.6777964	7.8632E-07	0.0067457	H3K4me3	LD

chr5	85259779	85259780	INS	chr5_85108451_85110049	4.09467575	13.6777964	7.8632E-07	0.0067457	H3K4me3	LD
chr16	3634854	3634855	INS	chr16_3599516_3599841	1.61566317	13.7027059	7.7537E-07	0.0067457	H3K4me3	LD
chr16	3825549	3825550	INS	chr16_3599516_3599841	1.61566317	13.7027059	7.7537E-07	0.0067457	H3K4me3	LD
chr6	148411777	148412074	DEL	chr6_148304857_148305885	2.06828419	13.5900728	8.2627E-07	0.00692166	H3K4me3	LD
chr15	43758152	43759039	DEL	chr15_44028851_44029146	2.55592833	13.3705704	9.3659E-07	0.00754971	H3K4me3	LD
chr15	43941500	43944231	DEL	chr15_44028851_44029146	2.55592833	13.3705704	9.3659E-07	0.00754971	H3K4me3	LD
chr15	44190293	44190346	DEL	chr15_44028851_44029146	2.55592833	13.3705704	9.3659E-07	0.00754971	H3K4me3	LD
chr4	91016526	91016527	INS	chr4_91236022_91236332	1.96543125	13.3794501	9.3182E-07	0.00754971	H3K4me3	LD
chr1	164176481	164176482	INS	chr1_164019999_164020774	2.08223744	13.40242	9.196E-07	0.00754971	H3K4me3	LD
chr15	44084450	44084451	INS	chr15_44028851_44029146	2.55592833	13.3705704	9.3659E-07	0.00754971	H3K4me3	LD
chr15	44143523	44143524	INS	chr15_44028851_44029146	2.55592833	13.3705704	9.3659E-07	0.00754971	H3K4me3	LD
chr15	44266439	44266440	INS	chr15_44028851_44029146	2.55592833	13.3705704	9.3659E-07	0.00754971	H3K4me3	LD
chr15	44496162	44496163	INS	chr15_44028851_44029146	2.55592833	13.3705704	9.3659E-07	0.00754971	H3K4me3	LD

chr14	138112027	138112028	INS	chr14_137736694_137737099	1.17545275	13.34471	9.5064E-07	0.00763415	H3K4me3	LD
chr1	128942200	128942495	DEL	chr1_129023454_129024686	1.36991112	13.3009292	9.7497E-07	0.00774219	H3K4me3	LD
chr1	129016140	129016141	INS	chr1_129023454_129024686	1.36991112	13.3009292	9.7497E-07	0.00774219	H3K4me3	LD
chr1	129305511	129305512	INS	chr1_129023454_129024686	1.36991112	13.3009292	9.7497E-07	0.00774219	H3K4me3	LD
chr3	1628687	1628957	DEL	chr3_2089042_2091520	6.48358786	13.2280171	1.017E-06	0.00795799	H3K4me3	LD
chr3	2078437	2078519	DEL	chr3_2089042_2091520	6.48358786	13.2280171	1.017E-06	0.00795799	H3K4me3	LD
chr3	2039109	2039110	INS	chr3_2089042_2091520	6.48358786	13.2280171	1.017E-06	0.00795799	H3K4me3	LD
chr3	2082592	2082593	INS	chr3_2089042_2091520	6.48358786	13.2280171	1.017E-06	0.00795799	H3K4me3	LD
chr7	43650766	43650767	INS	chr7_43927334_43927530	3.49354694	13.1843085	1.0432E-06	0.00801616	H3K4me3	LD
chr7	43826592	43826593	INS	chr7_43927334_43927530	3.49354694	13.1843085	1.0432E-06	0.00801616	H3K4me3	LD
chr7	43838146	43838147	INS	chr7_43927334_43927530	3.49354694	13.1843085	1.0432E-06	0.00801616	H3K4me3	LD
chr7	43884036	43884037	INS	chr7_43927334_43927530	3.49354694	13.1843085	1.0432E-06	0.00801616	H3K4me3	LD
chr5	10124550	10124551	INS	chr5_10423347_10423674	2.64612139	13.1565072	1.0603E-06	0.00808902	H3K4me3	LD

chr5	10454542	10454543	INS	chr5_10423347_10423674	2.64612139	13.1565072	1.0603E-06	0.00808902	H3K4me3	LD
chr5	79364639	79364640	INS	chr5_79840231_79840961	1.95530322	13.1004067	1.0957E-06	0.00832916	H3K4me3	LD
chr18	50124205	50124386	DEL	chr18_50113585_50113870	1.45916806	12.9340802	1.2086E-06	0.00846644	H3K4me3	LD
chr18	50168174	50168479	DEL	chr18_50113585_50113870	1.45916806	12.9340802	1.2086E-06	0.00846644	H3K4me3	LD
chr6	71314426	71315566	DEL	chr6_71583836_71584302	1.70682681	12.91201	1.2245E-06	0.00846644	H3K4me3	LD
chr17	40742290	40742340	DEL	chr17_40627051_40627478	8.6792738	12.9480914	1.1986E-06	0.00846644	H3K4me3	LD
chr8	71578731	71578975	DEL	chr8_71099246_71099572	2.18195278	12.8860652	1.2435E-06	0.00846644	H3K4me3	LD
chr14	94841153	94841215	DEL	chr14_94921503_94922055	2.49091778	12.9952012	1.1656E-06	0.00846644	H3K4me3	LD
chr14	95236353	95236646	DEL	chr14_94921503_94922055	2.49091778	12.9952012	1.1656E-06	0.00846644	H3K4me3	LD
chr6	148423200	148423280	DEL	chr6_148856587_148858603	3.7660885	12.84868	1.2716E-06	0.00846644	H3K4me3	LD
chr6	148597915	148597994	DEL	chr6_148856587_148858603	3.7660885	12.84868	1.2716E-06	0.00846644	H3K4me3	LD
chr1	141074864	141074924	DEL	chr1_141458776_141459207	1.99012924	13.0036295	1.1598E-06	0.00846644	H3K4me3	LD
chr11	10775578	10775894	DEL	chr11_10884782_10885145	2.47541688	12.8577364	1.2647E-06	0.00846644	H3K4me3	LD

chr1	15652175	15652246	DEL	chr1_16031865_16032687	2.50047444	12.8931528	1.2383E-06	0.00846644	H3K4me3	LD
chr1	16101216	16101427	DEL	chr1_16031865_16032687	2.50047444	12.8931528	1.2383E-06	0.00846644	H3K4me3	LD
chr1	16453277	16453351	DEL	chr1_16031865_16032687	2.50047444	12.8931528	1.2383E-06	0.00846644	H3K4me3	LD
chr15	56712911	56712912	INS	chr15_57034670_57036035	6.91646484	13.0450298	1.1319E-06	0.00846644	H3K4me3	LD
chr18	50042739	50042740	INS	chr18_50113585_50113870	1.45916806	12.9340802	1.2086E-06	0.00846644	H3K4me3	LD
chr18	50206610	50206611	INS	chr18_50113585_50113870	1.45916806	12.9340802	1.2086E-06	0.00846644	H3K4me3	LD
chr18	50345775	50345776	INS	chr18_50113585_50113870	1.45916806	12.9340802	1.2086E-06	0.00846644	H3K4me3	LD
chr18	50545255	50545256	INS	chr18_50113585_50113870	1.45916806	12.9340802	1.2086E-06	0.00846644	H3K4me3	LD
chr8	70929158	70929159	INS	chr8_71099246_71099572	2.18195278	12.8860652	1.2435E-06	0.00846644	H3K4me3	LD
chr8	71515478	71515479	INS	chr8_71099246_71099572	2.18195278	12.8860652	1.2435E-06	0.00846644	H3K4me3	LD
chr5	64299488	64299489	INS	chr5_64349780_64350536	1.91139772	12.8723407	1.2537E-06	0.00846644	H3K4me3	LD
chr14	95238033	95238034	INS	chr14_94921503_94922055	2.49091778	12.9952012	1.1656E-06	0.00846644	H3K4me3	LD
chr13	122578230	122578231	INS	chr13_122998066_122998618	2.05519013	12.943983	1.2015E-06	0.00846644	H3K4me3	LD

chr13	123125052	123125053	INS	chr13_122998066_122998618	2.05519013	12.943983	1.2015E-06	0.00846644	H3K4me3	LD
chr6	148712317	148712318	INS	chr6_148856587_148858603	3.7660885	12.84868	1.2716E-06	0.00846644	H3K4me3	LD
chr6	148763290	148763291	INS	chr6_148856587_148858603	3.7660885	12.84868	1.2716E-06	0.00846644	H3K4me3	LD
chr6	148965946	148965947	INS	chr6_148856587_148858603	3.7660885	12.84868	1.2716E-06	0.00846644	H3K4me3	LD
chr1	141594721	141594722	INS	chr1_141458776_141459207	1.99012924	13.0036295	1.1598E-06	0.00846644	H3K4me3	LD
chr13	179241524	179241525	INS	chr13_179538210_179538476	2.38060729	12.9853042	1.1725E-06	0.00846644	H3K4me3	LD
chr11	10443396	10443397	INS	chr11_10884782_10885145	2.47541688	12.8577364	1.2647E-06	0.00846644	H3K4me3	LD
chr11	10472383	10472384	INS	chr11_10884782_10885145	2.47541688	12.8577364	1.2647E-06	0.00846644	H3K4me3	LD
chr11	11237291	11237292	INS	chr11_10884782_10885145	2.47541688	12.8577364	1.2647E-06	0.00846644	H3K4me3	LD
chr11	11272971	11272972	INS	chr11_10884782_10885145	2.47541688	12.8577364	1.2647E-06	0.00846644	H3K4me3	LD
chr11	11361553	11361554	INS	chr11_10884782_10885145	2.47541688	12.8577364	1.2647E-06	0.00846644	H3K4me3	LD
chr1	15993713	15993714	INS	chr1_16031865_16032687	2.50047444	12.8931528	1.2383E-06	0.00846644	H3K4me3	LD
chr1	16056082	16056083	INS	chr1_16031865_16032687	2.50047444	12.8931528	1.2383E-06	0.00846644	H3K4me3	LD

chr6	34749305	34749306	INS	chr6_35230324_35231815	2.22852706	12.8929034	1.2385E-06	0.00846644	H3K4me3	LD
chr17	14241034	14241035	INS	chr17_13870903_13871828	1.7606305	12.847741	1.2723E-06	0.00846644	H3K4me3	LD
chr6	6240949	6240950	INS	chr6_5977407_5978579	2.51603292	12.8316912	1.2845E-06	0.00852142	H3K4me3	LD
chr2	58590284	58590285	INS	chr2_58411633_58412260	8.3390999	12.8077686	1.303E-06	0.00859083	H3K4me3	LD
chr8	64360842	64360843	INS	chr8_64566913_64567179	2.61602271	12.5699317	1.5042E-06	0.00985621	H3K4me3	LD
chr1	229398592	229398593	INS	chr1_229628729_229628894	3.56595979	12.5010237	1.5688E-06	0.01018584	H3K4me3	LD
chr1	229396062	229396063	INS	chr1_229628729_229628894	3.56595979	12.5010237	1.5688E-06	0.01018584	H3K4me3	LD
chr12	4060292	4061493	DEL	chr12_4059172_4060175	2.43265403	12.4309722	1.6377E-06	0.01026329	H3K4me3	LD
chr12	4076277	4076579	DEL	chr12_4059172_4060175	2.43265403	12.4309722	1.6377E-06	0.01026329	H3K4me3	LD
chr8	73303889	73303947	DEL	chr8_73465657_73466287	1.98973535	12.2595358	1.821E-06	0.01026329	H3K4me3	LD
chr14	107267549	107267622	DEL	chr14_107179603_107180060	2.06730514	12.2307787	1.8539E-06	0.01026329	H3K4me3	LD
chr14	129222574	129222638	DEL	chr14_129660563_129661785	-10.617369	-12.210348	1.8778E-06	0.01026329	H3K4me3	LD
chr14	129437482	129437770	DEL	chr14_129660563_129661785	-10.617369	-12.210348	1.8778E-06	0.01026329	H3K4me3	LD

chr14	129468677	129468941	DEL	chr14_129660563_129661785	-10.617369	-12.210348	1.8778E-06	0.01026329	H3K4me3	LD
chr14	129584699	129585001	DEL	chr14_129660563_129661785	-10.617369	-12.210348	1.8778E-06	0.01026329	H3K4me3	LD
chr14	129848177	129848503	DEL	chr14_129660563_129661785	-10.617369	-12.210348	1.8778E-06	0.01026329	H3K4me3	LD
chr14	129931390	129931675	DEL	chr14_129660563_129661785	-10.617369	-12.210348	1.8778E-06	0.01026329	H3K4me3	LD
chr14	1783307	1786514	DEL	chr14_1547190_1547542	2.29468438	12.4122158	1.6567E-06	0.01026329	H3K4me3	LD
chr14	1963858	1964017	DEL	chr14_1547190_1547542	2.29468438	12.4122158	1.6567E-06	0.01026329	H3K4me3	LD
chr11	12123084	12123305	DEL	chr11_11914311_11914510	3.18613833	12.296291	1.7798E-06	0.01026329	H3K4me3	LD
chr6	37693615	37693803	DEL	chr6_37723752_37724778	4.39661762	12.3784346	1.6916E-06	0.01026329	H3K4me3	LD
chr6	37711707	37711926	DEL	chr6_37723752_37724778	4.39661762	12.3784346	1.6916E-06	0.01026329	H3K4me3	LD
chr6	37721546	37721853	DEL	chr6_37723752_37724778	4.39661762	12.3784346	1.6916E-06	0.01026329	H3K4me3	LD
chr2	30360643	30360813	DEL	chr2_30172788_30173420	2.77504528	12.2654192	1.8143E-06	0.01026329	H3K4me3	LD
chr6	37745188	37745521	DEL	chr6_37723752_37724778	4.39661762	12.3784346	1.6916E-06	0.01026329	H3K4me3	LD
chr12	3896436	3896437	INS	chr12_4059172_4060175	2.43265403	12.4309722	1.6377E-06	0.01026329	H3K4me3	LD

chr8	73024668	73024669	INS	chr8_73465657_73466287	1.98973535	12.2595358	1.821E-06	0.01026329	H3K4me3	LD
chr8	73149691	73149692	INS	chr8_73465657_73466287	1.98973535	12.2595358	1.821E-06	0.01026329	H3K4me3	LD
chr8	73233306	73233307	INS	chr8_73465657_73466287	1.98973535	12.2595358	1.821E-06	0.01026329	H3K4me3	LD
chr8	73388812	73388813	INS	chr8_73465657_73466287	1.98973535	12.2595358	1.821E-06	0.01026329	H3K4me3	LD
chr8	73941604	73941605	INS	chr8_73465657_73466287	1.98973535	12.2595358	1.821E-06	0.01026329	H3K4me3	LD
chr5	63589295	63589296	INS	chr5_63491281_63492728	3.49532011	12.3001063	1.7756E-06	0.01026329	H3K4me3	LD
chr14	107087508	107087509	INS	chr14_107179603_107180060	2.06730514	12.2307787	1.8539E-06	0.01026329	H3K4me3	LD
chr14	107200516	107200517	INS	chr14_107179603_107180060	2.06730514	12.2307787	1.8539E-06	0.01026329	H3K4me3	LD
chr14	107244970	107244971	INS	chr14_107179603_107180060	2.06730514	12.2307787	1.8539E-06	0.01026329	H3K4me3	LD
chr14	107246780	107246781	INS	chr14_107179603_107180060	2.06730514	12.2307787	1.8539E-06	0.01026329	H3K4me3	LD
chr14	107254120	107254121	INS	chr14_107179603_107180060	2.06730514	12.2307787	1.8539E-06	0.01026329	H3K4me3	LD
chr14	107283999	107284000	INS	chr14_107179603_107180060	2.06730514	12.2307787	1.8539E-06	0.01026329	H3K4me3	LD
chr14	107261348	107261349	INS	chr14_107179603_107180060	2.06730514	12.2307787	1.8539E-06	0.01026329	H3K4me3	LD

chr14	107582898	107582899	INS	chr14_107179603_107180060	2.06730514	12.2307787	1.8539E-06	0.01026329	H3K4me3	LD
chr14	129583021	129583022	INS	chr14_129660563_129661785	-10.617369	-12.210348	1.8778E-06	0.01026329	H3K4me3	LD
chr14	129640944	129640945	INS	chr14_129660563_129661785	-10.617369	-12.210348	1.8778E-06	0.01026329	H3K4me3	LD
chr14	129729439	129729440	INS	chr14_129660563_129661785	-10.617369	-12.210348	1.8778E-06	0.01026329	H3K4me3	LD
chr14	129839166	129839167	INS	chr14_129660563_129661785	-10.617369	-12.210348	1.8778E-06	0.01026329	H3K4me3	LD
chr14	1066643	1066644	INS	chr14_1547190_1547542	2.29468438	12.4122158	1.6567E-06	0.01026329	H3K4me3	LD
chr14	1803868	1803869	INS	chr14_1547190_1547542	2.29468438	12.4122158	1.6567E-06	0.01026329	H3K4me3	LD
chr14	1905186	1905187	INS	chr14_1547190_1547542	2.29468438	12.4122158	1.6567E-06	0.01026329	H3K4me3	LD
chr2	147422712	147422713	INS	chr2_147438874_147439177	1.97501789	12.2828682	1.7947E-06	0.01026329	H3K4me3	LD
chr2	834088	834089	INS	chr2_839970_841616	6.63986938	12.4000189	1.6692E-06	0.01026329	H3K4me3	LD
chr2	1053697	1053698	INS	chr2_839970_841616	6.63986938	12.4000189	1.6692E-06	0.01026329	H3K4me3	LD
chr2	1056976	1056977	INS	chr2_839970_841616	6.63986938	12.4000189	1.6692E-06	0.01026329	H3K4me3	LD
chr6	20054205	20054206	INS	chr6_19881829_19883115	0.85000167	12.2583364	1.8224E-06	0.01026329	H3K4me3	LD

chr6	20055729	20055730	INS	chr6_19881829_19883115	0.85000167	12.2583364	1.8224E-06	0.01026329	H3K4me3	LD
chr8	32089956	32089957	INS	chr8_32069235_32069786	1.61249578	12.2055365	1.8834E-06	0.01026329	H3K4me3	LD
chr8	32288931	32288932	INS	chr8_32069235_32069786	1.61249578	12.2055365	1.8834E-06	0.01026329	H3K4me3	LD
chr2	30288777	30288778	INS	chr2_30172788_30173420	2.77504528	12.2654192	1.8143E-06	0.01026329	H3K4me3	LD
chr2	30338030	30338031	INS	chr2_30172788_30173420	2.77504528	12.2654192	1.8143E-06	0.01026329	H3K4me3	LD
chr6	37775953	37775954	INS	chr6_37723752_37724778	4.39661762	12.3784346	1.6916E-06	0.01026329	H3K4me3	LD
chr6	37744198	37744199	INS	chr6_37723752_37724778	4.39661762	12.3784346	1.6916E-06	0.01026329	H3K4me3	LD
chr6	37813083	37813084	INS	chr6_37723752_37724778	4.39661762	12.3784346	1.6916E-06	0.01026329	H3K4me3	LD
chr5	101709943	101709944	INS	chr5_102128991_102129151	2.32980028	12.140061	1.9623E-06	0.01063903	H3K4me3	LD
chr5	101679073	101679074	INS	chr5_102128991_102129151	2.32980028	12.140061	1.9623E-06	0.01063903	H3K4me3	LD
chr11	21444756	21444757	INS	chr11_21549401_21550368	1.09152613	12.1358462	1.9675E-06	0.01064025	H3K4me3	LD
chr1	68952880	68952950	DEL	chr1_69406466_69406792	1.5608122	12.0878884	2.0279E-06	0.01091125	H3K4me3	LD
chr1	69627872	69627873	INS	chr1_69406466_69406792	1.5608122	12.0878884	2.0279E-06	0.01091125	H3K4me3	LD

chr3	121657515	121657516	INS	chr3_121457400_121458077	1.04481564	12.0698489	2.0511E-06	0.01100849	H3K4me3	LD
chr10	60462409	60462410	INS	chr10_60642092_60642703	1.43577731	12.0254031	2.1096E-06	0.01126588	H3K4me3	LD
chr15	71490383	71490672	DEL	chr15_71496456_71498375	-7.91906	-11.936576	2.2322E-06	0.01185046	H3K4me3	LD
chr16	9032909	9036475	DEL	chr16_9115560_9115889	1.70965533	11.9235939	2.2508E-06	0.01185046	H3K4me3	LD
chr16	9590659	9593049	DEL	chr16_9115560_9115889	1.70965533	11.9235939	2.2508E-06	0.01185046	H3K4me3	LD
chr15	71812217	71812218	INS	chr15_71496456_71498375	-7.91906	-11.936576	2.2322E-06	0.01185046	H3K4me3	LD
chr16	74409740	74409741	INS	chr16_74529029_74529187	1.33836569	11.9256507	2.2478E-06	0.01185046	H3K4me3	LD
chr13	205648607	205648608	INS	chr13_205524675_205525288	1.9783515	11.9148128	2.2634E-06	0.01185046	H3K4me3	LD
chr13	205801345	205801346	INS	chr13_205524675_205525288	1.9783515	11.9148128	2.2634E-06	0.01185046	H3K4me3	LD
chr1	18325395	18325396	INS	chr1_17979465_17980258	0.79229288	11.916166	2.2615E-06	0.01185046	H3K4me3	LD
chr3	121657515	121657516	INS	chr3_121736156_121737413	1.77256688	11.8865404	2.3048E-06	0.01203728	H3K4me3	LD
chr18	1666142	1666195	DEL	chr18_2000659_2001646	1.27121189	11.8209443	2.4039E-06	0.01208249	H3K4me3	LD
chr18	1978810	1979096	DEL	chr18_2000659_2001646	1.27121189	11.8209443	2.4039E-06	0.01208249	H3K4me3	LD

chr18	2475075	2475694	DEL	chr18_2000659_2001646	1.27121189	11.8209443	2.4039E-06	0.01208249	H3K4me3	LD
chr11	15394609	15394913	DEL	chr11_15897358_15897666	2.29427042	11.8295991	2.3906E-06	0.01208249	H3K4me3	LD
chr11	16253211	16253371	DEL	chr11_15897358_15897666	2.29427042	11.8295991	2.3906E-06	0.01208249	H3K4me3	LD
chr11	16300589	16300773	DEL	chr11_15897358_15897666	2.29427042	11.8295991	2.3906E-06	0.01208249	H3K4me3	LD
chr11	16351503	16352329	DEL	chr11_15897358_15897666	2.29427042	11.8295991	2.3906E-06	0.01208249	H3K4me3	LD
chr8	120532861	120532862	INS	chr8_120346853_120348294	4.02406594	11.8663235	2.3348E-06	0.01208249	H3K4me3	LD
chr8	120818907	120818908	INS	chr8_120346853_120348294	4.02406594	11.8663235	2.3348E-06	0.01208249	H3K4me3	LD
chr1	248000066	248000067	INS	chr1_247804032_247804813	1.91596944	11.8761318	2.3202E-06	0.01208249	H3K4me3	LD
chr18	1953063	1953064	INS	chr18_2000659_2001646	1.27121189	11.8209443	2.4039E-06	0.01208249	H3K4me3	LD
chr11	15813356	15813357	INS	chr11_15897358_15897666	2.29427042	11.8295991	2.3906E-06	0.01208249	H3K4me3	LD
chr11	16041318	16041319	INS	chr11_15897358_15897666	2.29427042	11.8295991	2.3906E-06	0.01208249	H3K4me3	LD
chr11	16263489	16263490	INS	chr11_15897358_15897666	2.29427042	11.8295991	2.3906E-06	0.01208249	H3K4me3	LD
chr11	16253742	16253743	INS	chr11_15897358_15897666	2.29427042	11.8295991	2.3906E-06	0.01208249	H3K4me3	LD

chr11	16299574	16299575	INS	chr11_15897358_15897666	2.29427042	11.8295991	2.3906E-06	0.01208249	H3K4me3	LD
chr17	59934477	59934606	DEL	chr17_60073313_60074534	4.56442026	11.7728091	2.4797E-06	0.0123002	H3K4me3	LD
chr17	60029359	60029487	DEL	chr17_60073313_60074534	4.56442026	11.7728091	2.4797E-06	0.0123002	H3K4me3	LD
chr3	111527701	111527810	DEL	chr3_111387990_111388771	1.29292756	11.7607892	2.4991E-06	0.0123002	H3K4me3	LD
chr17	59938267	59938268	INS	chr17_60073313_60074534	4.56442026	11.7728091	2.4797E-06	0.0123002	H3K4me3	LD
chr17	60527683	60527684	INS	chr17_60073313_60074534	4.56442026	11.7728091	2.4797E-06	0.0123002	H3K4me3	LD
chr3	111367076	111367077	INS	chr3_111387990_111388771	1.29292756	11.7607892	2.4991E-06	0.0123002	H3K4me3	LD
chr3	111528491	111528492	INS	chr3_111387990_111388771	1.29292756	11.7607892	2.4991E-06	0.0123002	H3K4me3	LD
chr3	111846153	111846154	INS	chr3_111387990_111388771	1.29292756	11.7607892	2.4991E-06	0.0123002	H3K4me3	LD
chr6	40281913	40281914	INS	chr6_40500731_40501893	3.68704	11.7419351	2.5297E-06	0.01242259	H3K4me3	LD
chr1	15652175	15652246	DEL	chr1_16054604_16055049	1.86431861	11.7090165	2.5843E-06	0.01248964	H3K4me3	LD
chr1	16101216	16101427	DEL	chr1_16054604_16055049	1.86431861	11.7090165	2.5843E-06	0.01248964	H3K4me3	LD
chr1	16453277	16453351	DEL	chr1_16054604_16055049	1.86431861	11.7090165	2.5843E-06	0.01248964	H3K4me3	LD

chr5	63589295	63589296	INS	NW_018085127.1_42134_42312	2.16263278	11.7159399	2.5727E-06	0.01248964	H3K4me3	LD
chr1	15993713	15993714	INS	chr1_16054604_16055049	1.86431861	11.7090165	2.5843E-06	0.01248964	H3K4me3	LD
chr1	16056082	16056083	INS	chr1_16054604_16055049	1.86431861	11.7090165	2.5843E-06	0.01248964	H3K4me3	LD
chr1	215312842	215313273	DEL	chr1_215070180_215070918	2.70703924	11.6786524	2.6358E-06	0.01263698	H3K4me3	LD
chr18	4368868	4369014	DEL	chr18_4147903_4149061	1.48908433	11.653214	2.6799E-06	0.01263698	H3K4me3	LD
chr18	4413501	4413648	DEL	chr18_4147903_4149061	1.48908433	11.653214	2.6799E-06	0.01263698	H3K4me3	LD
chr18	4563236	4563521	DEL	chr18_4147903_4149061	1.48908433	11.653214	2.6799E-06	0.01263698	H3K4me3	LD
chr18	4593933	4594363	DEL	chr18_4147903_4149061	1.48908433	11.653214	2.6799E-06	0.01263698	H3K4me3	LD
chr18	4605216	4605284	DEL	chr18_4147903_4149061	1.48908433	11.653214	2.6799E-06	0.01263698	H3K4me3	LD
chr1	214774237	214782083	DUP	chr1_215070180_215070918	5.41407847	11.6786524	2.6358E-06	0.01263698	H3K4me3	LD
chr18	3958585	3958586	INS	chr18_4147903_4149061	1.48908433	11.653214	2.6799E-06	0.01263698	H3K4me3	LD
chr18	4590937	4590938	INS	chr18_4147903_4149061	1.48908433	11.653214	2.6799E-06	0.01263698	H3K4me3	LD
chr18	4592819	4592820	INS	chr18_4147903_4149061	1.48908433	11.653214	2.6799E-06	0.01263698	H3K4me3	LD

chr3	15224561	15224562	INS	chr3_15574499_15574761	2.90963311	11.6813005	2.6313E-06	0.01263698	H3K4me3	LD
chr2	43740155	43740156	INS	chr2_44049916_44050523	1.93237161	11.6037734	2.7679E-06	0.0130231	H3K4me3	LD
chr14	138517099	138517348	DEL	chr14_138852715_138854050	3.72392188	11.5964075	2.7812E-06	0.01302867	H3K4me3	LD
chr14	139199874	139199875	INS	chr14_138852715_138854050	3.72392188	11.5964075	2.7812E-06	0.01302867	H3K4me3	LD
chr4	77067623	77067624	INS	chr4_77144767_77145601	2.08428678	11.5570605	2.854E-06	0.01334007	H3K4me3	LD
chr16	3289419	3289420	INS	chr16_3454239_3455082	0.91860844	11.5399731	2.8862E-06	0.01346131	H3K4me3	LD
chr10	60462409	60462410	INS	chr10_60719971_60720297	1.13466981	11.529059	2.907E-06	0.01352881	H3K4me3	LD
chr18	8249322	8249394	DEL	chr18_8643110_8643339	2.54218328	11.5002088	2.9628E-06	0.01375857	H3K4me3	LD
chr6	163400141	163400189	DEL	chr6_163885649_163885914	2.31784819	11.4659076	3.0307E-06	0.01398279	H3K4me3	LD
chr6	164185952	164185953	INS	chr6_163885649_163885914	2.31784819	11.4659076	3.0307E-06	0.01398279	H3K4me3	LD
chr6	164249585	164249586	INS	chr6_163885649_163885914	2.31784819	11.4659076	3.0307E-06	0.01398279	H3K4me3	LD
chr7	84531436	84531437	INS	chr7_84566852_84567140	2.32352813	11.4241579	3.1158E-06	0.0143441	H3K4me3	LD
chr7	49297061	49299027	DEL	chr7_49381320_49382434	3.30549006	11.388018	3.1915E-06	0.01442765	H3K4me3	LD

chr7	49586613	49586664	DEL	chr7_49381320_49382434	3.30549006	11.388018	3.1915E-06	0.01442765	H3K4me3	LD
chr7	49272999	49273000	INS	chr7_49381320_49382434	3.30549006	11.388018	3.1915E-06	0.01442765	H3K4me3	LD
chr7	49421027	49421028	INS	chr7_49381320_49382434	3.30549006	11.388018	3.1915E-06	0.01442765	H3K4me3	LD
chr7	49587314	49587315	INS	chr7_49381320_49382434	3.30549006	11.388018	3.1915E-06	0.01442765	H3K4me3	LD
chr7	49615926	49615927	INS	chr7_49381320_49382434	3.30549006	11.388018	3.1915E-06	0.01442765	H3K4me3	LD
chr5	87629624	87629625	INS	chr5_87629140_87629869	2.29482574	11.3910978	3.185E-06	0.01442765	H3K4me3	LD
chr3	13581410	13581411	INS	chr3_13225162_13225846	1.74783392	11.3865255	3.1947E-06	0.01442765	H3K4me3	LD
chr4	111529407	111529590	DEL	chr4_111976802_111977083	2.77897285	11.3135464	3.3542E-06	0.01447487	H3K4me3	LD
chr4	112216586	112216758	DEL	chr4_111976802_111977083	2.77897285	11.3135464	3.3542E-06	0.01447487	H3K4me3	LD
chr8	131890907	131891196	DEL	chr8_132201628_132202675	2.76645674	11.3229307	3.3332E-06	0.01447487	H3K4me3	LD
chr8	132029950	132030057	DEL	chr8_132201628_132202675	2.76645674	11.3229307	3.3332E-06	0.01447487	H3K4me3	LD
chr8	132051363	132051858	DEL	chr8_132201628_132202675	2.76645674	11.3229307	3.3332E-06	0.01447487	H3K4me3	LD
chr8	132293696	132294084	DEL	chr8_132201628_132202675	2.76645674	11.3229307	3.3332E-06	0.01447487	H3K4me3	LD

chr6	148423200	148423280	DEL	chr6_148143300_148143833	1.85655567	11.3341031	3.3084E-06	0.01447487	H3K4me3	LD
chr6	148597915	148597994	DEL	chr6_148143300_148143833	1.85655567	11.3341031	3.3084E-06	0.01447487	H3K4me3	LD
chr18	54184198	54184199	INS	chr18_54516899_54517555	1.15154646	11.3684461	3.2334E-06	0.01447487	H3K4me3	LD
chr4	111482116	111482117	INS	chr4_111976802_111977083	2.77897285	11.3135464	3.3542E-06	0.01447487	H3K4me3	LD
chr4	111524672	111524673	INS	chr4_111976802_111977083	2.77897285	11.3135464	3.3542E-06	0.01447487	H3K4me3	LD
chr4	111876285	111876286	INS	chr4_111976802_111977083	2.77897285	11.3135464	3.3542E-06	0.01447487	H3K4me3	LD
chr4	111869970	111869971	INS	chr4_111976802_111977083	2.77897285	11.3135464	3.3542E-06	0.01447487	H3K4me3	LD
chr4	112447773	112447774	INS	chr4_111976802_111977083	2.77897285	11.3135464	3.3542E-06	0.01447487	H3K4me3	LD
chr8	131721765	131721766	INS	chr8_132201628_132202675	2.76645674	11.3229307	3.3332E-06	0.01447487	H3K4me3	LD
chr6	147640087	147640088	INS	chr6_148143300_148143833	1.85655567	11.3341031	3.3084E-06	0.01447487	H3K4me3	LD
chr8	132178459	132178460	INS	chr8_132201628_132202675	5.53291347	11.3229307	3.3332E-06	0.01447487	H3K4me3	LD
chr8	132241515	132241516	INS	chr8_132201628_132202675	2.76645674	11.3229307	3.3332E-06	0.01447487	H3K4me3	LD
chr18	12006248	12006249	INS	chr18_11800936_11801712	4.04602965	11.3538306	3.2651E-06	0.01447487	H3K4me3	LD

chr18	11955677	11955678	INS	chr18_11800936_11801712	4.04602965	11.3538306	3.2651E-06	0.01447487	H3K4me3	LD
NW_018085335.1	106693	107184	DEL	chr14_6162414_6162736	2.78649338	11.3005792	3.3835E-06	0.01447641	H3K4me3	LD
chr10	11698833	11698834	INS	chr10_11786853_11787264	2.6123705	11.2954084	3.3952E-06	0.01447641	H3K4me3	LD
chr10	11979215	11979216	INS	chr10_11786853_11787264	2.6123705	11.2954084	3.3952E-06	0.01447641	H3K4me3	LD
chr10	12030922	12030923	INS	chr10_11786853_11787264	2.6123705	11.2954084	3.3952E-06	0.01447641	H3K4me3	LD
chr10	12138054	12138055	INS	chr10_11786853_11787264	2.6123705	11.2954084	3.3952E-06	0.01447641	H3K4me3	LD
chr17	38233034	38233084	DEL	chr17_38568961_38569630	1.60880062	11.2791901	3.4324E-06	0.0144902	H3K4me3	LD
chr13	3393952	3394105	DEL	chr13_3627204_3627832	1.95362675	11.2802919	3.4299E-06	0.0144902	H3K4me3	LD
chr13	3221377	3221378	INS	chr13_3627204_3627832	1.95362675	11.2802919	3.4299E-06	0.0144902	H3K4me3	LD
chr13	3699315	3699316	INS	chr13_3627204_3627832	1.95362675	11.2802919	3.4299E-06	0.0144902	H3K4me3	LD
chr13	3846459	3846460	INS	chr13_3627204_3627832	1.95362675	11.2802919	3.4299E-06	0.0144902	H3K4me3	LD
chr11	3066164	3066218	DEL	chr11_2731692_2732202	1.61505872	11.2593612	3.4784E-06	0.01459802	H3K4me3	LD
chr7	71105016	71105161	DEL	chr7_70652882_70653320	2.27684264	11.2603905	3.476E-06	0.01459802	H3K4me3	LD

chr11	2739831	2739832	INS	chr11_2731692_2732202	1.61505872	11.2593612	3.4784E-06	0.01459802	H3K4me3	LD
chr8	78887071	78887072	INS	chr8_79156378_79157231	1.36226183	11.2417399	3.5199E-06	0.01474321	H3K4me3	LD
chr7	53574936	53574986	DEL	chr7_53664965_53667873	16.9675563	11.223232	3.5641E-06	0.01489905	H3K4me3	LD
chr16	35086569	35086570	INS	chr16_34780444_34781965	2.352342	11.2054739	3.6071E-06	0.01504924	H3K4me3	LD
chr10	58147556	58149393	DEL	chr10_57650514_57651260	2.14183944	11.1882458	3.6493E-06	0.01519582	H3K4me3	LD
chr14	138112027	138112028	INS	chr14_137934665_137935434	1.76545769	11.1501061	3.7449E-06	0.01556318	H3K4me3	LD
chr18	1506997	1507665	DEL	chr18_1454198_1454799	4.06817444	11.1239719	3.8119E-06	0.01561419	H3K4me3	LD
chr15	24951939	24956849	DEL	chr15_25448171_25449048	1.96013767	11.0946685	3.8887E-06	0.01561419	H3K4me3	LD
chr15	25320801	25321010	DEL	chr15_25448171_25449048	1.96013767	11.0946685	3.8887E-06	0.01561419	H3K4me3	LD
chr7	36163869	36164028	DEL	chr7_35905908_35906188	2.84463264	11.1015181	3.8706E-06	0.01561419	H3K4me3	LD
chr16	65632961	65632962	INS	chr16_65793427_65794802	2.329879	11.1098225	3.8488E-06	0.01561419	H3K4me3	LD
chr16	65989179	65989180	INS	chr16_65793427_65794802	2.329879	11.1098225	3.8488E-06	0.01561419	H3K4me3	LD
chr16	66224473	66224474	INS	chr16_65793427_65794802	2.329879	11.1098225	3.8488E-06	0.01561419	H3K4me3	LD

chr16	66235346	66235347	INS	chr16_65793427_65794802	2.329879	11.1098225	3.8488E-06	0.01561419	H3K4me3	LD
chr18	1236632	1236633	INS	chr18_1454198_1454799	4.06817444	11.1239719	3.8119E-06	0.01561419	H3K4me3	LD
chr11	12326595	12326596	INS	chr11_12309128_12310276	4.80445286	11.0949306	3.888E-06	0.01561419	H3K4me3	LD
chr11	12382644	12382645	INS	chr11_12309128_12310276	4.80445286	11.0949306	3.888E-06	0.01561419	H3K4me3	LD
chr15	25284197	25284198	INS	chr15_25448171_25449048	1.96013767	11.0946685	3.8887E-06	0.01561419	H3K4me3	LD
chr2	48005027	48005028	INS	chr2_48043807_48044037	2.16350243	11.0986307	3.8782E-06	0.01561419	H3K4me3	LD
chr2	48280369	48280370	INS	chr2_48043807_48044037	2.16350243	11.0986307	3.8782E-06	0.01561419	H3K4me3	LD
chr7	36107995	36107996	INS	chr7_35905908_35906188	2.84463264	11.1015181	3.8706E-06	0.01561419	H3K4me3	LD
chr9	126359771	126359772	INS	chr9_125913550_125914075	2.00600431	11.0779726	3.9332E-06	0.01573382	H3K4me3	LD
chr4	57988408	57988530	DEL	chr4_58407828_58408514	1.86981644	11.0724558	3.9481E-06	0.01576365	H3K4me3	LD
chr14	45315997	45316248	DEL	chr14_44933977_44934493	3.28204609	11.059008	3.9845E-06	0.01587942	H3K4me3	LD
chr16	63132855	63133143	DEL	chr16_63175374_63177235	-13.135905	-10.990294	4.1766E-06	0.01634018	H3K4me3	LD
chr16	63127527	63127578	DEL	chr16_63175374_63177235	-26.271811	-10.990294	4.1766E-06	0.01634018	H3K4me3	LD

chr13	39261622	39261912	DEL	chr13_39686312_39687246	4.15998333	10.9967682	4.1581E-06	0.01634018	H3K4me3	LD
chr13	39728296	39728605	DEL	chr13_39686312_39687246	4.15998333	10.9967682	4.1581E-06	0.01634018	H3K4me3	LD
chr16	63077068	63077069	INS	chr16_63175374_63177235	-13.135905	-10.990294	4.1766E-06	0.01634018	H3K4me3	LD
chr16	63150440	63150441	INS	chr16_63175374_63177235	-13.135905	-10.990294	4.1766E-06	0.01634018	H3K4me3	LD
chr16	63206352	63206353	INS	chr16_63175374_63177235	-13.135905	-10.990294	4.1766E-06	0.01634018	H3K4me3	LD
chr13	39186600	39186601	INS	chr13_39686312_39687246	4.15998333	10.9967682	4.1581E-06	0.01634018	H3K4me3	LD
chr13	39448923	39448924	INS	chr13_39686312_39687246	4.15998333	10.9967682	4.1581E-06	0.01634018	H3K4me3	LD
chr18	20796323	20796324	INS	chr18_20664654_20665040	2.10415062	11.0016009	4.1443E-06	0.01634018	H3K4me3	LD
chr16	27724811	27727100	DEL	chr16_27780635_27782299	7.11899311	10.9514629	4.2897E-06	0.01675209	H3K4me3	LD
chr9	67166023	67166449	DEL	chr9_67201354_67202451	4.28149188	10.9321952	4.3471E-06	0.01691441	H3K4me3	LD
chr9	66810574	66810575	INS	chr9_67201354_67202451	4.28149188	10.9321952	4.3471E-06	0.01691441	H3K4me3	LD
chr1	104001593	104001594	INS	chr1_103936801_103937138	1.56993546	10.9175106	4.3915E-06	0.0170558	H3K4me3	LD
chr16	20400935	20401009	DEL	chr16_20822345_20823251	1.45401032	10.8950467	4.4602E-06	0.0171669	H3K4me3	LD

NW_018084840.1	43092	43394	DEL	chr4_28089393_28091423	1.6301985	10.9016408	4.4399E-06	0.0171669	H3K4me3	LD
chr16	21178648	21178705	DEL	chr16_20822345_20823251	1.45401032	10.8950467	4.4602E-06	0.0171669	H3K4me3	LD
chr4	28191395	28191396	INS	chr4_28089393_28091423	1.6301985	10.9016408	4.4399E-06	0.0171669	H3K4me3	LD
chr4	63621352	63621437	DEL	chr4_63961060_63962433	7.379565	10.868774	4.5422E-06	0.0173013	H3K4me3	LD
chr16	29833932	29834012	DEL	chr16_30218500_30219942	3.00331658	10.8657207	4.5519E-06	0.0173013	H3K4me3	LD
chr16	30514654	30514888	DEL	chr16_30218500_30219942	6.00663315	10.8657207	4.5519E-06	0.0173013	H3K4me3	LD
chr16	30584564	30584639	DEL	chr16_30218500_30219942	3.00331658	10.8657207	4.5519E-06	0.0173013	H3K4me3	LD
chr16	30555564	30555565	INS	chr16_30218500_30219942	3.00331658	10.8657207	4.5519E-06	0.0173013	H3K4me3	LD
chr16	30707657	30707658	INS	chr16_30218500_30219942	3.00331658	10.8657207	4.5519E-06	0.0173013	H3K4me3	LD
chr5	40186785	40186834	DEL	chr5_40182038_40182663	6.28385078	10.8339592	4.6535E-06	0.01759358	H3K4me3	LD
chr5	40194281	40194330	DEL	chr5_40182038_40182663	6.28385078	10.8339592	4.6535E-06	0.01759358	H3K4me3	LD
chr5	40181920	40181921	INS	chr5_40182038_40182663	6.28385078	10.8339592	4.6535E-06	0.01759358	H3K4me3	LD
chr9	122734148	122734375	DEL	chr9_123090705_123091361	1.79957417	10.8084891	4.7368E-06	0.0176894	H3K4me3	LD

chr9	123336879	123339793	DEL	chr9_123090705_123091361	1.79957417	10.8084891	4.7368E-06	0.0176894	H3K4me3	LD
chr1	86136311	86136312	INS	chr1_85827813_85828315	1.9845191	10.8107047	4.7295E-06	0.0176894	H3K4me3	LD
chr1	86279573	86279574	INS	chr1_85827813_85828315	1.9845191	10.8107047	4.7295E-06	0.0176894	H3K4me3	LD
chr9	123493024	123493025	INS	chr9_123090705_123091361	1.79957417	10.8084891	4.7368E-06	0.0176894	H3K4me3	LD
chr11	74726871	74726969	DEL	chr11_74546021_74546825	3.9685025	10.7803344	4.8308E-06	0.0180091	H3K4me3	LD
chr6	13274225	13274389	DEL	chr6_13194769_13195059	2.59184689	10.7699094	4.8662E-06	0.01801512	H3K4me3	LD
chr6	12762882	12762883	INS	chr6_13194769_13195059	2.59184689	10.7699094	4.8662E-06	0.01801512	H3K4me3	LD
chr6	13129139	13129140	INS	chr6_13194769_13195059	2.59184689	10.7699094	4.8662E-06	0.01801512	H3K4me3	LD
chr6	13569108	13569109	INS	chr6_13194769_13195059	2.59184689	10.7699094	4.8662E-06	0.01801512	H3K4me3	LD
chr2	148119463	148119776	DEL	chr2_147833894_147834342	1.75571661	10.7450676	4.9516E-06	0.01823647	H3K4me3	LD
chr2	147422712	147422713	INS	chr2_147833894_147834342	1.75571661	10.7450676	4.9516E-06	0.01823647	H3K4me3	LD
chr2	148121124	148121125	INS	chr2_147833894_147834342	1.75571661	10.7450676	4.9516E-06	0.01823647	H3K4me3	LD
chr15	84696254	84696310	DEL	chr15_84718776_84719181	1.60786396	10.7222111	5.0316E-06	0.01824815	H3K4me3	LD

chr7	49255224	49255225	INS	chr7_49687462_49688766	0.948443	10.722552	5.0304E-06	0.01824815	H3K4me3	LD
chr15	84754885	84754886	INS	chr15_84718776_84719181	1.60786396	10.7222111	5.0316E-06	0.01824815	H3K4me3	LD
chr15	85010449	85010450	INS	chr15_84718776_84719181	1.60786396	10.7222111	5.0316E-06	0.01824815	H3K4me3	LD
chr15	85018787	85018788	INS	chr15_84718776_84719181	1.60786396	10.7222111	5.0316E-06	0.01824815	H3K4me3	LD
chr14	88636891	88636892	INS	chr14_88586115_88586861	1.6635665	10.7161202	5.0532E-06	0.01829535	H3K4me3	LD
chr17	54592988	54593171	DEL	chr17_54996045_54997575	2.1521755	10.6884223	5.1526E-06	0.01831033	H3K4me3	LD
chr13	199906400	199906697	DEL	chr13_200324319_200325867	-4.17713	-10.698467	5.1163E-06	0.01831033	H3K4me3	LD
chr13	199907445	199907745	DEL	chr13_200324319_200325867	-4.17713	-10.698467	5.1163E-06	0.01831033	H3K4me3	LD
chr13	200148906	200149019	DEL	chr13_200324319_200325867	-4.17713	-10.698467	5.1163E-06	0.01831033	H3K4me3	LD
chr13	200516284	200516570	DEL	chr13_200324319_200325867	-4.17713	-10.698467	5.1163E-06	0.01831033	H3K4me3	LD
chr8	27185255	27185594	DEL	chr8_26942975_26943677	2.20200368	10.688231	5.1533E-06	0.01831033	H3K4me3	LD
chr14	52219869	52221464	DEL	chr14_51732467_51733016	49.7140181	10.6937829	5.1332E-06	0.01831033	H3K4me3	LD
chr15	118551012	118551013	INS	chr15_119032999_119033287	2.31584833	10.6893886	5.1491E-06	0.01831033	H3K4me3	LD

chr13	200301517	200301518	INS	chr13_200324319_200325867	-4.17713	-10.698467	5.1163E-06	0.01831033	H3K4me3	LD
chr13	200390948	200390949	INS	chr13_200324319_200325867	-4.17713	-10.698467	5.1163E-06	0.01831033	H3K4me3	LD
chr8	26842431	26842432	INS	chr8_26942975_26943677	2.20200368	10.688231	5.1533E-06	0.01831033	H3K4me3	LD
chr14	13417219	13417220	INS	chr14_13587041_13587828	1.0707739	10.6863394	5.1602E-06	0.01831033	H3K4me3	LD
chr4	71017558	71017841	DEL	chr4_70619606_70620995	1.25931131	10.6756629	5.1992E-06	0.01841803	H3K4me3	LD
chr7	3696530	3696531	INS	chr7_3370018_3370621	1.49017556	10.6720768	5.2123E-06	0.0184341	H3K4me3	LD
chr3	51987462	51987736	DEL	chr3_52410452_52411274	2.94038589	10.6356248	5.3483E-06	0.01845655	H3K4me3	LD
chr3	52101617	52101893	DEL	chr3_52410452_52411274	2.94038589	10.6356248	5.3483E-06	0.01845655	H3K4me3	LD
chr8	71746910	71746993	DEL	chr8_71700655_71701726	3.06917828	10.6524255	5.2851E-06	0.01845655	H3K4me3	LD
chr5	6613062	6613356	DEL	chr5_7009870_7011841	-4.274536	-10.638188	5.3386E-06	0.01845655	H3K4me3	LD
chr5	6614524	6614875	DEL	chr5_7009870_7011841	-4.274536	-10.638188	5.3386E-06	0.01845655	H3K4me3	LD
chr5	6674834	6675144	DEL	chr5_7009870_7011841	4.274536	10.6381877	5.3386E-06	0.01845655	H3K4me3	LD
chr6	12848652	12849549	DEL	chr6_13089094_13089353	0.88708194	10.6427053	5.3216E-06	0.01845655	H3K4me3	LD

chr3	52016762	52016763	INS	chr3_52410452_52411274	2.94038589	10.6356248	5.3483E-06	0.01845655	H3K4me3	LD
chr3	52012571	52012572	INS	chr3_52410452_52411274	2.94038589	10.6356248	5.3483E-06	0.01845655	H3K4me3	LD
chr8	71812768	71812769	INS	chr8_71700655_71701726	3.06917828	10.6524255	5.2851E-06	0.01845655	H3K4me3	LD
chr5	6611859	6611860	INS	chr5_7009870_7011841	-4.274536	-10.638188	5.3386E-06	0.01845655	H3K4me3	LD
chr5	6616163	6616164	INS	chr5_7009870_7011841	-4.274536	-10.638188	5.3386E-06	0.01845655	H3K4me3	LD
chr5	6620824	6620825	INS	chr5_7009870_7011841	-4.274536	-10.638188	5.3386E-06	0.01845655	H3K4me3	LD
chr5	6784220	6784221	INS	chr5_7009870_7011841	4.274536	10.6381877	5.3386E-06	0.01845655	H3K4me3	LD
chr6	12922694	12922695	INS	chr6_13089094_13089353	0.88708194	10.6427053	5.3216E-06	0.01845655	H3K4me3	LD
chr4	83871126	83871409	DEL	chr4_84164975_84165789	-3.403304	-10.613118	5.4342E-06	0.01869265	H3K4me3	LD
chr4	83871648	83871649	INS	chr4_84164975_84165789	-3.403304	-10.613118	5.4342E-06	0.01869265	H3K4me3	LD
chr18	1511526	1511527	INS	chr18_1968836_1969840	5.3097031	10.5992407	5.4879E-06	0.01884716	H3K4me3	LD
NW_018085246.1	328807	329087	DEL	chr1_142256550_142257340	-4.666614	-10.57019	5.6024E-06	0.01887231	H3K4me3	LD
NW_018085246.1	357496	357556	DEL	chr1_142256550_142257340	-4.666614	-10.57019	5.6024E-06	0.01887231	H3K4me3	LD

chr1	142151990	142152063	DEL	chr1_142256550_142257340	-4.666614	-10.57019	5.6024E-06	0.01887231	H3K4me3	LD
chr14	13185975	13186245	DEL	chr14_12905926_12906096	3.03875347	10.5472586	5.6946E-06	0.01887231	H3K4me3	LD
chr12	36458676	36458996	DEL	chr12_36181096_36182190	2.12797613	10.5311693	5.7603E-06	0.01887231	H3K4me3	LD
chr7	2711131	2711378	DEL	chr7_2735240_2736177	3.31505783	10.5633402	5.6297E-06	0.01887231	H3K4me3	LD
chr7	3168350	3168981	DEL	chr7_2735240_2736177	3.31505783	10.5633402	5.6297E-06	0.01887231	H3K4me3	LD
chr7	3226757	3226758	INS	chr7_2735240_2736177	3.31505783	10.5633402	5.6297E-06	0.01887231	H3K4me3	LD
chr4	99792968	99792969	INS	chr4_100093426_100094239	3.14634056	10.5395254	5.7261E-06	0.01887231	H3K4me3	LD
chr4	100136603	100136604	INS	chr4_100093426_100094239	3.14634056	10.5395254	5.7261E-06	0.01887231	H3K4me3	LD
chr4	100168005	100168006	INS	chr4_100093426_100094239	3.14634056	10.5395254	5.7261E-06	0.01887231	H3K4me3	LD
chr4	100245877	100245878	INS	chr4_100093426_100094239	3.14634056	10.5395254	5.7261E-06	0.01887231	H3K4me3	LD
chr4	100217377	100217378	INS	chr4_100093426_100094239	3.14634056	10.5395254	5.7261E-06	0.01887231	H3K4me3	LD
chr4	100225175	100225176	INS	chr4_100093426_100094239	3.14634056	10.5395254	5.7261E-06	0.01887231	H3K4me3	LD
chr1	119077652	119077653	INS	chr1_119363920_119364745	1.91470622	10.5607587	5.6401E-06	0.01887231	H3K4me3	LD

chr1	119166234	119166235	INS	chr1_119363920_119364745	1.91470622	10.5607587	5.6401E-06	0.01887231	H3K4me3	LD
NW_018085246.1	305762	305763	INS	chr1_142256550_142257340	-4.666614	-10.57019	5.6024E-06	0.01887231	H3K4me3	LD
NW_018085246.1	356177	356178	INS	chr1_142256550_142257340	-4.666614	-10.57019	5.6024E-06	0.01887231	H3K4me3	LD
NW_018085246.1	351928	351929	INS	chr1_142256550_142257340	-4.666614	-10.57019	5.6024E-06	0.01887231	H3K4me3	LD
chr7	2449976	2449977	INS	chr7_2735240_2736177	3.31505783	10.5633402	5.6297E-06	0.01887231	H3K4me3	LD
chr12	35897790	35897791	INS	chr12_36181096_36182190	2.12797613	10.5311693	5.7603E-06	0.01887231	H3K4me3	LD
chr12	36000771	36000772	INS	chr12_36181096_36182190	2.12797613	10.5311693	5.7603E-06	0.01887231	H3K4me3	LD
chr12	36223197	36223198	INS	chr12_36181096_36182190	2.12797613	10.5311693	5.7603E-06	0.01887231	H3K4me3	LD
chr12	36594744	36594745	INS	chr12_36181096_36182190	2.12797613	10.5311693	5.7603E-06	0.01887231	H3K4me3	LD
chr7	2675915	2675916	INS	chr7_2735240_2736177	3.31505783	10.5633402	5.6297E-06	0.01887231	H3K4me3	LD
chr7	2689666	2689667	INS	chr7_2735240_2736177	3.31505783	10.5633402	5.6297E-06	0.01887231	H3K4me3	LD
chr7	2691233	2691234	INS	chr7_2735240_2736177	3.31505783	10.5633402	5.6297E-06	0.01887231	H3K4me3	LD
chr7	35078467	35078468	INS	chr7_35095388_35096472	4.8333525	10.5203583	5.8049E-06	0.0189894	H3K4me3	LD

chr17	54592988	54593171	DEL	chr17_54998311_54999269	1.16749594	10.4729981	6.0051E-06	0.01907289	H3K4me3	LD
chr6	5625414	5625474	DEL	chr6_5792066_5792346	1.99292728	10.475265	5.9953E-06	0.01907289	H3K4me3	LD
chr6	5843921	5843983	DEL	chr6_5792066_5792346	1.99292728	10.475265	5.9953E-06	0.01907289	H3K4me3	LD
chr1	33121969	33122174	DEL	chr1_32996315_32996963	2.0851465	10.4942163	5.9145E-06	0.01907289	H3K4me3	LD
chr1	33122896	33123201	DEL	chr1_32996315_32996963	2.0851465	10.4942163	5.9145E-06	0.01907289	H3K4me3	LD
chr6	5418221	5418222	INS	chr6_5792066_5792346	1.99292728	10.475265	5.9953E-06	0.01907289	H3K4me3	LD
chr6	5413829	5413830	INS	chr6_5792066_5792346	1.99292728	10.475265	5.9953E-06	0.01907289	H3K4me3	LD
chr3	5439336	5439337	INS	chr3_5878044_5878512	1.6177085	10.4809246	5.971E-06	0.01907289	H3K4me3	LD
chr3	5588332	5588333	INS	chr3_5878044_5878512	1.6177085	10.4809246	5.971E-06	0.01907289	H3K4me3	LD
chr3	5764964	5764965	INS	chr3_5878044_5878512	1.6177085	10.4809246	5.971E-06	0.01907289	H3K4me3	LD
chr16	3289419	3289420	INS	chr16_3650432_3652132	1.15180625	10.5066429	5.8621E-06	0.01907289	H3K4me3	LD
chr3	6097384	6097385	INS	chr3_5878044_5878512	1.6177085	10.4809246	5.971E-06	0.01907289	H3K4me3	LD
chr1	22259473	22259474	INS	chr1_22239286_22243047	10.8675056	10.4638155	6.0447E-06	0.01907289	H3K4me3	LD

chr1	22380588	22380589	INS	chr1_22239286_22243047	10.8675056	10.4638155	6.0447E-06	0.01907289	H3K4me3	LD
chr1	22600789	22600790	INS	chr1_22239286_22243047	10.8675056	10.4638155	6.0447E-06	0.01907289	H3K4me3	LD
chr1	22646067	22646068	INS	chr1_22239286_22243047	10.8675056	10.4638155	6.0447E-06	0.01907289	H3K4me3	LD
chr1	32549279	32549280	INS	chr1_32996315_32996963	2.0851465	10.4942163	5.9145E-06	0.01907289	H3K4me3	LD
chr1	32658817	32658818	INS	chr1_32996315_32996963	2.0851465	10.4942163	5.9145E-06	0.01907289	H3K4me3	LD
chr1	32661405	32661406	INS	chr1_32996315_32996963	2.0851465	10.4942163	5.9145E-06	0.01907289	H3K4me3	LD
chr1	33083035	33083036	INS	chr1_32996315_32996963	2.0851465	10.4942163	5.9145E-06	0.01907289	H3K4me3	LD
chr15	29088150	29088151	INS	chr15_29478731_29479113	1.26186144	10.5055307	5.8668E-06	0.01907289	H3K4me3	LD
chr9	13483314	13483744	DEL	chr9_13330626_13331391	2.22298302	10.4542433	6.0864E-06	0.01917609	H3K4me3	LD
chr1	214153603	214153604	INS	chr1_213969466_213970161	1.26370498	10.4402308	6.148E-06	0.01934163	H3K4me3	LD
chr4	57988408	57988530	DEL	chr4_58351209_58352491	4.08679368	10.429902	6.1939E-06	0.01945723	H3K4me3	LD
chr4	91016526	91016527	INS	chr4_90846163_90846533	2.10418264	10.4114371	6.2768E-06	0.01968876	H3K4me3	LD
chr6	4092036	4092316	DEL	chr6_4188996_4189355	2.61018356	10.3728533	6.4541E-06	0.020016	H3K4me3	LD

chr6	4198614	4199094	DEL	chr6_4188996_4189355	2.61018356	10.3728533	6.4541E-06	0.020016	H3K4me3	LD
chr5	101550457	101550590	DEL	chr5_101545590_101546707	6.22285056	10.3684223	6.4748E-06	0.020016	H3K4me3	LD
chr6	4227113	4227114	INS	chr6_4188996_4189355	2.61018356	10.3728533	6.4541E-06	0.020016	H3K4me3	LD
chr6	4376608	4376609	INS	chr6_4188996_4189355	2.61018356	10.3728533	6.4541E-06	0.020016	H3K4me3	LD
chr5	101115789	101115790	INS	chr5_101545590_101546707	6.22285056	10.3684223	6.4748E-06	0.020016	H3K4me3	LD
chr5	101261658	101261659	INS	chr5_101545590_101546707	6.22285056	10.3684223	6.4748E-06	0.020016	H3K4me3	LD
chr5	101331136	101331137	INS	chr5_101545590_101546707	6.22285056	10.3684223	6.4748E-06	0.020016	H3K4me3	LD
chr5	101709943	101709944	INS	chr5_101545590_101546707	6.22285056	10.3684223	6.4748E-06	0.020016	H3K4me3	LD
chr5	101679073	101679074	INS	chr5_101545590_101546707	6.22285056	10.3684223	6.4748E-06	0.020016	H3K4me3	LD
chr13	54655193	54655483	DEL	chr13_54901875_54905216	4.50431875	10.3323355	6.6464E-06	0.02016783	H3K4me3	LD
chr3	121594348	121594420	DEL	chr3_121464128_121464765	1.18792822	10.3299884	6.6577E-06	0.02016783	H3K4me3	LD
chr2	143084053	143084526	DEL	chr2_143050198_143051065	4.94214389	10.3189703	6.7112E-06	0.02016783	H3K4me3	LD
chr10	13684885	13685195	DEL	chr10_13371287_13371825	2.00753389	10.31092	6.7505E-06	0.02016783	H3K4me3	LD

chr10	19087717	19087785	DEL	chr10_18842646_18843234	2.00376861	10.3476504	6.5729E-06	0.02016783	H3K4me3	LD
chr7	2117751	2117809	DEL	chr7_2369099_2370536	5.35987689	10.3313611	6.6511E-06	0.02016783	H3K4me3	LD
chr7	2791373	2791620	DEL	chr7_2369099_2370536	5.35987689	10.3313611	6.6511E-06	0.02016783	H3K4me3	LD
chr7	2788509	2789192	DEL	chr7_2369099_2370536	5.35987689	10.3313611	6.6511E-06	0.02016783	H3K4me3	LD
chr7	2789599	2789874	DEL	chr7_2369099_2370536	5.35987689	10.3313611	6.6511E-06	0.02016783	H3K4me3	LD
chr10	60462409	60462410	INS	chr10_60435675_60436652	1.75780856	10.3333615	6.6414E-06	0.02016783	H3K4me3	LD
chr1	6460049	6460050	INS	chr1_6406769_6407508	1.1095525	10.3150266	6.7304E-06	0.02016783	H3K4me3	LD
chr1	6643056	6643057	INS	chr1_6406769_6407508	1.1095525	10.3150266	6.7304E-06	0.02016783	H3K4me3	LD
chr3	121653617	121653618	INS	chr3_121464128_121464765	1.18792822	10.3299884	6.6577E-06	0.02016783	H3K4me3	LD
chr2	142562359	142562360	INS	chr2_143050198_143051065	4.94214389	10.3189703	6.7112E-06	0.02016783	H3K4me3	LD
chr2	142646589	142646590	INS	chr2_143050198_143051065	4.94214389	10.3189703	6.7112E-06	0.02016783	H3K4me3	LD
chr2	142760125	142760126	INS	chr2_143050198_143051065	4.94214389	10.3189703	6.7112E-06	0.02016783	H3K4me3	LD
chr2	142789505	142789506	INS	chr2_143050198_143051065	4.94214389	10.3189703	6.7112E-06	0.02016783	H3K4me3	LD

chr10	13108532	13108533	INS	chr10_13371287_13371825	2.00753389	10.31092	6.7505E-06	0.02016783	H3K4me3	LD
chr10	13215015	13215016	INS	chr10_13371287_13371825	2.00753389	10.31092	6.7505E-06	0.02016783	H3K4me3	LD
chr10	13793488	13793489	INS	chr10_13371287_13371825	2.00753389	10.31092	6.7505E-06	0.02016783	H3K4me3	LD
chr10	13782646	13782647	INS	chr10_13371287_13371825	2.00753389	10.31092	6.7505E-06	0.02016783	H3K4me3	LD
chr10	18367158	18367159	INS	chr10_18842646_18843234	2.00376861	10.3476504	6.5729E-06	0.02016783	H3K4me3	LD
chr7	2504066	2504067	INS	chr7_2369099_2370536	10.7197538	10.3313611	6.6511E-06	0.02016783	H3K4me3	LD
chr11	2481928	2481929	INS	chr11_2441295_2442879	5.256726	10.3498206	6.5626E-06	0.02016783	H3K4me3	LD
chr13	200308197	200308267	DEL	chr13_199946477_199946902	2.38617505	10.2926058	6.8411E-06	0.0204098	H3K4me3	LD
chr14	6432857	6434651	DEL	chr14_6162414_6162736	1.37604611	10.2646213	6.9821E-06	0.02062739	H3K4me3	LD
chr15	43941500	43944231	DEL	chr15_44432101_44432696	1.13955868	10.2533924	7.0395E-06	0.02062739	H3K4me3	LD
chr15	44190293	44190346	DEL	chr15_44432101_44432696	1.13955868	10.2533924	7.0395E-06	0.02062739	H3K4me3	LD
chr14	1066643	1066644	INS	chr14_1241867_1242515	1.64891706	10.259988	7.0057E-06	0.02062739	H3K4me3	LD
chr14	6579866	6579867	INS	chr14_6162414_6162736	1.37604611	10.2646213	6.9821E-06	0.02062739	H3K4me3	LD

chr15	44084450	44084451	INS	chr15_44432101_44432696	1.13955868	10.2533924	7.0395E-06	0.02062739	H3K4me3	LD
chr15	44143523	44143524	INS	chr15_44432101_44432696	1.13955868	10.2533924	7.0395E-06	0.02062739	H3K4me3	LD
chr15	44266439	44266440	INS	chr15_44432101_44432696	1.13955868	10.2533924	7.0395E-06	0.02062739	H3K4me3	LD
chr15	44496162	44496163	INS	chr15_44432101_44432696	1.13955868	10.2533924	7.0395E-06	0.02062739	H3K4me3	LD
chr15	44663667	44663668	INS	chr15_44432101_44432696	1.13955868	10.2533924	7.0395E-06	0.02062739	H3K4me3	LD
chr15	44667712	44667713	INS	chr15_44432101_44432696	1.13955868	10.2533924	7.0395E-06	0.02062739	H3K4me3	LD
chr15	44686781	44686782	INS	chr15_44432101_44432696	1.13955868	10.2533924	7.0395E-06	0.02062739	H3K4me3	LD
chr1	242026508	242027197	DEL	chr1_242160491_242161560	-7.6696612	-10.245781	7.0788E-06	0.02065743	H3K4me3	LD
chr1	242024379	242024630	DEL	chr1_242160491_242161560	-15.339322	-10.245781	7.0788E-06	0.02065743	H3K4me3	LD
chr1	242024630	242024631	INS	chr1_242160491_242161560	-15.339322	-10.245781	7.0788E-06	0.02065743	H3K4me3	LD
chr11	53786233	53786578	DEL	chr11_54210081_54211104	0.95580079	10.2298294	7.1619E-06	0.02087136	H3K4me3	LD
chr8	490687	490736	DEL	chr8_383068_385283	6.50088667	10.2030522	7.3038E-06	0.0211982	H3K4me3	LD
chr8	31981	32088	DEL	chr8_383068_385283	6.50088667	10.2030522	7.3038E-06	0.0211982	H3K4me3	LD

chr8	734923	734924	INS	chr8_383068_385283	6.50088667	10.2030522	7.3038E-06	0.0211982	H3K4me3	LD
chr11	59781261	59781459	DEL	chr11_59463984_59464832	0.89285044	10.161448	7.5306E-06	0.02167967	H3K4me3	LD
chr11	59790861	59790929	DEL	chr11_59463984_59464832	0.89285044	10.161448	7.5306E-06	0.02167967	H3K4me3	LD
chr3	68144528	68144998	DEL	chr3_68353856_68354168	1.05973069	10.162926	7.5224E-06	0.02167967	H3K4me3	LD
chr11	59770922	59770923	INS	chr11_59463984_59464832	0.89285044	10.161448	7.5306E-06	0.02167967	H3K4me3	LD
chr11	59748035	59748036	INS	chr11_59463984_59464832	0.89285044	10.161448	7.5306E-06	0.02167967	H3K4me3	LD
chr3	68141059	68142597	INV	chr3_68353856_68354168	2.11946138	10.162926	7.5224E-06	0.02167967	H3K4me3	LD
chr8	76032867	76032868	INS	chr8_75997054_75997272	1.78378083	10.1570664	7.5549E-06	0.02172046	H3K4me3	LD
chr8	32756409	32756486	DEL	chr8_32727304_32728051	1.92565544	10.1499478	7.5947E-06	0.02177607	H3K4me3	LD
chr8	32288931	32288932	INS	chr8_32727304_32728051	1.92565544	10.1499478	7.5947E-06	0.02177607	H3K4me3	LD
chr7	4162446	4162447	INS	chr7_4272618_4275454	4.47163	10.1421022	7.6387E-06	0.02181454	H3K4me3	LD
chr7	4189808	4189809	INS	chr7_4272618_4275454	4.47163	10.1421022	7.6387E-06	0.02181454	H3K4me3	LD
chr7	4190063	4190064	INS	chr7_4272618_4275454	4.47163	10.1421022	7.6387E-06	0.02181454	H3K4me3	LD

chr13	193909294	193909602	DEL	chr13_193437609_193438741	1.65963406	10.1101126	7.8213E-06	0.02223123	H3K4me3	LD
chr11	3460350	3460351	INS	chr11_3579777_3580016	2.57844206	10.1056704	7.8471E-06	0.02223123	H3K4me3	LD
chr11	3604129	3604130	INS	chr11_3579777_3580016	2.57844206	10.1056704	7.8471E-06	0.02223123	H3K4me3	LD
chr11	4064913	4064914	INS	chr11_3579777_3580016	2.57844206	10.1056704	7.8471E-06	0.02223123	H3K4me3	LD
chr13	193861641	193861642	INS	chr13_193437609_193438741	1.65963406	10.1101126	7.8213E-06	0.02223123	H3K4me3	LD
chr13	38039742	38039743	INS	chr13_38438198_38439517	3.44170625	10.081485	7.9889E-06	0.02251357	H3K4me3	LD
chr13	38355520	38355521	INS	chr13_38438198_38439517	3.44170625	10.081485	7.9889E-06	0.02251357	H3K4me3	LD
chr13	38813993	38813994	INS	chr13_38438198_38439517	3.44170625	10.081485	7.9889E-06	0.02251357	H3K4me3	LD
chr13	38906914	38906915	INS	chr13_38438198_38439517	3.44170625	10.081485	7.9889E-06	0.02251357	H3K4me3	LD
chr13	55040368	55040369	INS	chr13_55183169_55183559	1.28729019	10.0503298	8.1758E-06	0.02300998	H3K4me3	LD
NW_018085018.1	35702	35703	INS	NW_018085018.1_216455_217272	1.259417	10.034406	8.2732E-06	0.02316615	H3K4me3	LD
chr4	28812028	28812029	INS	chr4_28380564_28380880	1.78783215	10.0341706	8.2747E-06	0.02316615	H3K4me3	LD
chr4	28813807	28813808	INS	chr4_28380564_28380880	1.78783215	10.0341706	8.2747E-06	0.02316615	H3K4me3	LD

chr17	54711956	54712169	DEL	chr17_54529566_54529890	1.40724493	10.0002205	8.4868E-06	0.02355274	H3K4me3	LD
chr8	79882314	79882315	INS	chr8_80053841_80054162	1.60972104	10.0005283	8.4848E-06	0.02355274	H3K4me3	LD
chr8	80528371	80528372	INS	chr8_80053841_80054162	1.60972104	10.0005283	8.4848E-06	0.02355274	H3K4me3	LD
chr17	54549155	54549156	INS	chr17_54529566_54529890	1.40724493	10.0002205	8.4868E-06	0.02355274	H3K4me3	LD
chr17	54556056	54556057	INS	chr17_54529566_54529890	1.40724493	10.0002205	8.4868E-06	0.02355274	H3K4me3	LD
chr12	5187233	5187291	DEL	chr12_5430169_5430980	4.3900986	9.9785786	8.6252E-06	0.02371225	H3K4me3	LD
chr12	5367059	5367358	DEL	chr12_5430169_5430980	-2.1950493	-9.9785786	8.6252E-06	0.02371225	H3K4me3	LD
chr12	5492732	5492733	INS	chr12_5430169_5430980	-2.1950493	-9.9785786	8.6252E-06	0.02371225	H3K4me3	LD
chr12	5697365	5697366	INS	chr12_5430169_5430980	2.1950493	9.9785786	8.6252E-06	0.02371225	H3K4me3	LD
chr12	5909334	5909335	INS	chr12_5430169_5430980	-2.1950493	-9.9785786	8.6252E-06	0.02371225	H3K4me3	LD
chr1	103658379	103658467	DEL	chr1_103936801_103937138	1.55055354	9.97152464	8.6708E-06	0.02377653	H3K4me3	LD
chr1	104235030	104235313	DEL	chr1_103936801_103937138	1.55055354	9.97152464	8.6708E-06	0.02377653	H3K4me3	LD
chr8	131078419	131078420	INS	chr8_131557248_131557616	1.57349572	9.96677924	8.7017E-06	0.02383055	H3K4me3	LD

chr16	7039542	7039543	INS	chr16_6765532_6767555	3.23795901	9.94809563	8.8243E-06	0.02408833	H3K4me3	LD
chr1	147729521	147729702	DEL	chr1_147933595_147934205	2.17930847	9.93004215	8.9447E-06	0.02434021	H3K4me3	LD
chr1	147950260	147950261	INS	chr1_147933595_147934205	2.17930847	9.93004215	8.9447E-06	0.02434021	H3K4me3	LD
chr2	144634483	144634613	DEL	chr2_144611952_144612289	2.17621922	9.91939756	9.0166E-06	0.02445689	H3K4me3	LD
chr4	115398584	115398585	INS	chr4_115749600_115750289	2.24176956	9.91523245	9.0449E-06	0.02445689	H3K4me3	LD
chr4	115748918	115748919	INS	chr4_115749600_115750289	2.24176956	9.91523245	9.0449E-06	0.02445689	H3K4me3	LD
chr4	116067522	116067523	INS	chr4_115749600_115750289	2.24176956	9.91523245	9.0449E-06	0.02445689	H3K4me3	LD
chr5	78783594	78784412	DEL	chr5_78380507_78380810	2.6145184	9.87381958	9.3315E-06	0.02448159	H3K4me3	LD
chr15	136860874	136861184	DEL	chr15_137358671_137359767	1.99809944	9.883588	9.263E-06	0.02448159	H3K4me3	LD
chr15	137204907	137205043	DEL	chr15_137358671_137359767	1.99809944	9.883588	9.263E-06	0.02448159	H3K4me3	LD
chr15	137450333	137450387	DEL	chr15_137358671_137359767	1.99809944	9.883588	9.263E-06	0.02448159	H3K4me3	LD
chr15	137451755	137452011	DEL	chr15_137358671_137359767	1.99809944	9.883588	9.263E-06	0.02448159	H3K4me3	LD
chr1	254315710	254315799	DEL	chr1_254219472_254220261	1.86242913	9.90026188	9.1473E-06	0.02448159	H3K4me3	LD

chr9	40081249	40081365	DEL	chr9_40231452_40232551	2.75025156	9.86282083	9.4094E-06	0.02448159	H3K4me3	LD
chr18	45631495	45631808	DEL	chr18_45808631_45809108	2.24226542	9.86962074	9.3612E-06	0.02448159	H3K4me3	LD
chr15	137203151	137203235	DUP	chr15_137358671_137359767	3.99619889	9.883588	9.263E-06	0.02448159	H3K4me3	LD
chr7	42807695	42807696	INS	chr7_43027210_43028365	2.6172245	9.86022571	9.4278E-06	0.02448159	H3K4me3	LD
chr14	89220844	89220845	INS	chr14_89642286_89643095	2.22713794	9.88291061	9.2677E-06	0.02448159	H3K4me3	LD
chr5	78067627	78067628	INS	chr5_78380507_78380810	2.6145184	9.87381958	9.3315E-06	0.02448159	H3K4me3	LD
chr5	78857324	78857325	INS	chr5_78380507_78380810	2.6145184	9.87381958	9.3315E-06	0.02448159	H3K4me3	LD
chr15	137112525	137112526	INS	chr15_137358671_137359767	1.99809944	9.883588	9.263E-06	0.02448159	H3K4me3	LD
chr15	137450875	137450876	INS	chr15_137358671_137359767	1.99809944	9.883588	9.263E-06	0.02448159	H3K4me3	LD
chr15	137452933	137452934	INS	chr15_137358671_137359767	1.99809944	9.883588	9.263E-06	0.02448159	H3K4me3	LD
chr1	254536054	254536055	INS	chr1_254219472_254220261	1.86242913	9.90026188	9.1473E-06	0.02448159	H3K4me3	LD
chr18	29055855	29055856	INS	chr18_29140491_29141080	1.50084389	9.86288257	9.4089E-06	0.02448159	H3K4me3	LD
chr8	48542628	48542629	INS	chr8_48510687_48511204	2.02312806	9.85800898	9.4437E-06	0.02448159	H3K4me3	LD

chr8	48759493	48759494	INS	chr8_48510687_48511204	2.02312806	9.85800898	9.4437E-06	0.02448159	H3K4me3	LD
chr9	39894790	39894791	INS	chr9_40231452_40232551	2.75025156	9.86282083	9.4094E-06	0.02448159	H3K4me3	LD
chr9	39900020	39900021	INS	chr9_40231452_40232551	2.75025156	9.86282083	9.4094E-06	0.02448159	H3K4me3	LD
chr12	43436422	43436423	INS	chr12_43907446_43907817	2.06979589	9.86856181	9.3687E-06	0.02448159	H3K4me3	LD
chr12	43502490	43502491	INS	chr12_43907446_43907817	2.06979589	9.86856181	9.3687E-06	0.02448159	H3K4me3	LD
chr9	40421085	40421086	INS	chr9_40231452_40232551	2.75025156	9.86282083	9.4094E-06	0.02448159	H3K4me3	LD
chr9	40693059	40693060	INS	chr9_40231452_40232551	2.75025156	9.86282083	9.4094E-06	0.02448159	H3K4me3	LD
chr12	44053247	44053248	INS	chr12_43907446_43907817	2.06979589	9.86856181	9.3687E-06	0.02448159	H3K4me3	LD
chr12	44189089	44189090	INS	chr12_43907446_43907817	2.06979589	9.86856181	9.3687E-06	0.02448159	H3K4me3	LD
chr12	44198578	44198579	INS	chr12_43907446_43907817	2.06979589	9.86856181	9.3687E-06	0.02448159	H3K4me3	LD
chr18	45354096	45354097	INS	chr18_45808631_45809108	2.24226542	9.86962074	9.3612E-06	0.02448159	H3K4me3	LD
chr18	45606412	45606413	INS	chr18_45808631_45809108	2.24226542	9.86962074	9.3612E-06	0.02448159	H3K4me3	LD
chr1	114488786	114488787	INS	chr1_114926134_114926619	1.57673257	9.84558478	9.5328E-06	0.02462307	H3K4me3	LD

chr1	114620312	114620313	INS	chr1_114926134_114926619	1.57673257	9.84558478	9.5328E-06	0.02462307	H3K4me3	LD
chr1	114614128	114614129	INS	chr1_114926134_114926619	1.57673257	9.84558478	9.5328E-06	0.02462307	H3K4me3	LD
chr13	93308770	93308771	INS	chr13_93356222_93356835	0.89876944	9.83357248	9.6199E-06	0.02481802	H3K4me3	LD
chr13	110464456	110464515	DEL	chr13_110308305_110308671	2.41998133	9.82017384	9.7181E-06	0.02501093	H3K4me3	LD
chr2	141818450	141818451	INS	chr2_141361338_141362140	9.67291139	50.1077388	2.7862E-11	1.4879E-05	H3K4me3	LD
chr10	41995107	41995383	DEL	chr10_41815120_41816223	7.60900911	66.2709947	2.9908E-12	2.1296E-06	H3K4me3	LD
chr10	42068222	42068223	INS	chr10_41815120_41816223	7.60900911	66.2709947	2.9908E-12	2.1296E-06	H3K4me3	LD
